# Suction use in ureterorenoscopy: A systematic review and meta‐analysis of comparative studies

**DOI:** 10.1002/bco2.408

**Published:** 2024-07-08

**Authors:** Lazaros Tzelves, Robert Geraghty, Patrick Juliebø‐Jones, Yuhong Yuan, Konstantinos Kapriniotis, Daniele Castellani, Vineet Gauhar, Andreas Skolarikos, Bhaskar Somani

**Affiliations:** ^1^ Second Department of Urology, Sismanogleio Hospital National and Kapodistrian University of Athens Athens Greece; ^2^ Young Academic Urologists (YAU), Urolithiasis Group European Association of Urology (EAU) Arnhem Netherlands; ^3^ Department of Urology, Freeman Hospital Newcastle‐upon‐Tyne UK; ^4^ Institute of Genetic Medicine Newcastle University Newcastle‐upon‐Tyne UK; ^5^ Department of Urology Haukeland University Hospital Bergen Norway; ^6^ Department of Clinical Medicine University of Bergen Bergen Norway; ^7^ Department of Medicine London Health Science Centre London Ontario Canada; ^8^ Department of Medicine, Health Sciences Centre McMaster University Hamilton Ontario Canada; ^9^ Department of Urology Whipps cross University Hospital London UK; ^10^ Urology Division Azienda Ospedaliero‐Universitaria delle Marche Ancona Italy; ^11^ Ng Teng Fong General Hospital (NUHS) Singapore; ^12^ Department of Urology University of Southampton Southampton UK

**Keywords:** endourology, retrograde intrarenal surgery (RIRS), suction, ureteroscopy (URS), urolithiasis

## Abstract

**Objectives:**

Ureterorenoscopy is seeing a bloom of technological advances, one of which is incorporating suction. The objective of this study is to systematically review existing literature regarding suction use in rigid and flexible ureterorenoscopy and perform meta‐analysis of studies comparing suction versus no suction ureteroscopy or mini percutaneous nephrolithotomy (PCNL).

**Methods:**

A literature search was performed (November 2023) in MEDLINE, Embase and Cochrane CENTRAL. Study protocol was registered at PROSPERO (CRD42023482360). Comparative studies (observational and randomized) were eligible for inclusion if they compared suction versus no suction group and reported at least one primary outcome of interest (stone‐free or complication rate).

**Results:**

Sixteen studies (5 randomized and 11 observational), analysing 1086 and 1109 patients in standard and suction groups, respectively, were included. Final stone‐free rates (SFRs), overall and infectious complications and length of hospital stay exhibited significant improvement when suction was used. When mini‐PCNL was compared with flexible ureterorenoscopy with suction, no differences were found in terms of stone‐free and infectious complications rates.

**Conclusions:**

Ureterorenoscopy is a commonly performed endoscopic procedure for urolithiasis treatment, the success of which is defined by SFRs and complication rates. Application of suction via ureteral access sheaths, ureteral catheters or scopes may provide improved SFRs, reduced overall and infectious complication rates, along with a reduction in length of hospital stay. Further randomized studies are needed to validate these findings and standardize indications and protocols.

## 1 INTRODUCTION

The increasing urolithiasis incidence has led to a bloom of technological advances in the field of endourology; from adoption of the miniaturized, digital and single‐use flexible scopes to high‐power and more efficient laser fibres and machines,^1^, ^2^ all of them led to an increase in utilization of endoscopic procedures such as percutaneous nephrolithotomy (PCNL) and ureteroscopy (URS)/retrograde intrarenal surgery (RIRS). European Association of Urology Guidelines on Urolithiasis set the clear indication for proper management selection according to stone size, composition and location, with RIRS being a suitable choice for stones up to 2 cm in size in most cases, while the increased efficiency seen with the implementation of new technologies is anticipated to expand these indications.^3^ Despite the wide adoption of this technique, RIRS does not come without cost both for patients and healthcare systems; up to 22% of patients may have residual fragments, and infectious complications are not uncommon, while life‐threatening sepsis may be seen in 1.3% of cases.^4^

Stone‐free rates (SFRs) are related to surgical expertise, appropriate patient selection, surgical technique (stone dusting, fragmentation and pop‐dusting) and available surgical equipment.^5^ There is a scientific debate according to proper size cut‐off to define clinically insignificantly residual fragments (CIRFs), but studies have shown that even stone particles 2–4 mm can lead to recurrence, while those measured >4 mm are related to re‐intervention rates in a significant proportion of patients.^6^ Therefore, surgeons are frequently relying on manual extraction of fragments using baskets and/or forceps, which can lead to increased operating time and potentially complications. Infectious complications have been clearly associated with infected urine, increased operating time and raised intrarenal temperature or pressure, which can lead to pyelovenous and pyelolymphatic backflow of urine into systemic circulation.^7^ Suction has been initially used in endoscopic stone management for more than 25 years during PCNL, but the emergence of new devices applying suction through patented ureteroscopes, ureteral access sheaths (UAS) and ureteral catheters led to application of suction in URS/RIRS.^8^

The aim of this systematic review and meta‐analysis is to appraise existing literature on suction use during URS/RIRS and provide pooled estimates for its safety and effectiveness.

## 2 MATERIALS AND METHODS

This systematic review and meta‐analysis were conducted according to the Preferred Reporting Items for Systematic Reviews and Meta‐Analyses (PRISMA) statement.^9^

### 2.1 Data sources and searches

Two authors (L.T. and P.J.) independently performed the literature search in MEDLINE, Embase and Cochrane Central Register of Controlled Trials (CENTRAL) (via Ovid) from inception to 10 November 2023, using the following search algorithm as provided in Appendix S1 (control vocabulary and text words were searched using terms related to ureterorenoscopy and suction use). Conference abstracts and case reports were excluded in the search. Duplicate studies were removed, while reference lists of included studies were also screened. In case of disagreement between the two authors, a third author (R.G.) was advised to reach consensus. The protocol for the systematic review/meta‐analysis was registered in PROSPERO (CRD42023482360).

### 2.2 Eligibility criteria, data extraction and outcome of interest

Only English clinical papers were accepted (Table 1). The PICOS model (Patient Intervention Comparison Outcome Study type) was used to frame and answer to the clinical question:

P: adult patients with ureteral/kidney stones;
I: URS/RIRS using suction;
C: URS/RIRS not using suction or PCNL;
O: primary: complications and SFR; secondary: operative time (OR time), stone fragmentation time, lasering time, fluoroscopy time, length of hospital stay (LOS), auxiliary procedures, readmission rates, cost, post‐procedural pain scores and quality of life indicators
S: prospective and retrospective comparative studies.

**TABLE 1:** Baseline patient and study characteristics.

Author/year	Study design	Control arm					Experimental arm					Details about suction
		Definition	Sample size	Age (± SD)	BMI (± SD)	Energy used for lithotripsy	Definition	Sample size	Age (± SD)	BMI (± SD)	Energy used for lithotripsy	
Sur 2022	RCT	Flexible URS	8	37	23.4	Ho:YAG lithotripsy (0.6–1.0 J, 6–12 Hz), 270 μm fibre	Flexible URS with suction	9	42	22.3	Ho:YAG lithotripsy (0.6–1.0 J, 6–12 Hz), 270 μm fibre	SURE procedure using the C‐VAC aspiration system, a custom aspiration catheter used to navigate all areas of collecting system under fluoroscopy. The device has a steering control dial to allow deflection and guidance into collecting system, allowing irrigation and aspiration via connection to a vacuum port with suction set at 150–200 mmHg. Fragments up to 2.5 mm can be removed (inner aspiration channel 7.5 Fr)
Tang 2023	RCT	Mini‐PCNL	87	51.3 (8.2)	22.6 (3.2)	Ho:YAG lithotripsy (15–20 W), 200 or 365 μm fibre	Flexible URS with suction	86	52.7 (9.3)	23.1 (3.9)	Ho:YAG lithotripsy (0.8–1.0 J, 15–20 Hz), 200 μm fibre	Novel disposable vacuum‐assisted ureteral access sheath (V‐UAS, Y‐type) consisting from an expansion tube, tube connector, sheath tube and operating handle
Chen 2019	Retrospective	Mini‐PCNL	45	39.4 (17.9)	27.3 (4.4)	Ho:YAG lithotripsy (2.0 J, 20 Hz)	Flexible URS with suction	44	45.7 (11.9)	28.2 (8.2)	Ho:YAG lithotripsy (0.8 J, 30 Hz)	An intelligent irrigation and suctioning pressure monitoring platform with the integrated pressure‐measuring flexible ureteral access sheath with pressure sensing and suctioning function through pressure feedback control technology
Du 2019	RCT	Semi‐rigid URS	60	47 (15.7)	NR	Ho:YAG lithotripsy (0.6–0.8 J, 25–30 Hz), 550 μm fibre	Semi‐rigid URS with suction	62	47.4 (13.2)	NR	Ho:YAG lithotripsy (0.6–0.8 J, 25–30 Hz), 550 μm fibre	Patented perfusion and suctioning platform consisting of a main control unit, perfusion and suctioning device and pressure feedback device. Platform allows setting perfusion flow rate, control pressure, alarming pressure and maximum pressure level. Ureteral access sheath inner diameter is 12 Fr and outer diameter 14 Fr with a length of 30–45 cm. A pressure sensor lies at the front and two connection channels at the back end, which are connected to the pressure monitoring, feedback device and negative pressure suctioning device. If the pressure of the operation exceeds the alarming pressure, platform gives alarm, while if it exceeds the maximum pressure level the platform stops perfusion automatically
Lai 2020	Retrospective	Mini‐PCNL	56	49.6 (12.2)	25.3 (4.1)	Ho:YAG lithotripsy or pneumatic lithotripter	Flexible URS with suction	28	45.2 (10.4)	25 (3.5)	Ho:YAG lithotripsy (1.0–1.5 J, 15–20 Hz), 200 μm fibre	Novel vacuum‐assisted ureteral access sheath (V‐UAS, ClearPetra) with an oblique drainage tube connected to a negative pressure aspirator
Zhu 2018	Retrospective	Flexible URS	165	51.7 (15.8)	23.1 (3.4)	Ho:YAG lithotripsy (12–16 W for ureter, 12–20 W for kidney, 14–20 Hz), 200 μm fibre	Flexible URS with suction	165	53.9 (13.4)	22.9 (2.6)	Ho:YAG lithotripsy (12–16 W for ureter, 12–20 W for kidney, 14–20 Hz), 200 μm fibre	Suction system included a modified ureteral access sheath and a vacuum device, connected at the back end of the sheath. An elastic rubber film with a hole on the tail end of the UAS enhanced the efficiency of suction by providing an airproof. Additionally, on the back end of the UAS, another channel covered by a red cap worked as an air door to regulate the negative pressure. Perfusion flow and negative pressure were set at 60–140 ml/min and 3–8 kPa, while to maintain satisfactory suctioning effect, the pressure was dynamically regulated by manually twisting the red cap on the tail end of the sheath. When lithotripsy was completed, the flexible ureteroscope was detracted, a 5F ureteral catheter was inserted into the UAS, its tip was placed in the ureteropelvic junction, and the tail end of the ureteral catheter was injected with saline to create artificial water circulation. The flow of artificial water circulation and the negative pressure were set to approximately 180 ml/min and 5 kPa, respectively, and were maintained for 20–40 s
Zhang 2021	Retrospective	Semi‐rigid URS	50	54.3 (11.6)	21.8 (3.4)	Ho:YAG lithotripsy, 200 μm fibre	Semi‐rigid URS with suction	56	53.8 (12.1)	20.8 (3.4)	Ho:YAG lithotripsy, 200 μm fibre	The new vacuum suction ureteroscope consisted of a standard ureteroscope (9.8F), a lithotripsy endoscope (6F), a standard semi‐rigid ureteroscopic access sheath (13F) and a vacuum suction device
Zhang 2021	Retrospective	Flexible URS	54	55.2 (10.2)	21.9 (3.5)	Ho:YAG lithotripsy, 200 μm fibre	Semi‐rigid URS with suction	56	53.8 (12.1)	20.8 (3.4)	Ho:YAG lithotripsy, 200 μm fibre	The new vacuum suction ureteroscope consisted of a standard ureteroscope (9.8F), a lithotripsy endoscope (6F), a standard semi‐rigid ureteroscopic access sheath (13F) and a vacuum suction device
Lechevallier 2003	RCT	Semi‐rigid URS	14	NR	NR	Pneumatic lithotripter	Semi‐rigid URS with suction	11	NR	NR	Pneumatic lithotripter	Automated electronically controlled irrigation/suction system, consisting of an irrigation roller pump with a pressure control that supplies continuous irrigation to the ureteroscope and a suction roller pump with a constant flow rate that removes liquid and stone particles from the cavity. Pressure was set at 150 cm H20 (110.3 mmHg) and suction at 200 ml/min
Huang 2023	Retrospective	Flexible URS	103	54.7 (10.7)	26.5 (4.9)	Ho:YAG lithotripsy (1.2 J, 20 Hz)	Flexible URS with suction	103	54.5 (11)	26.3 (4.2)	Ho:YAG lithotripsy (1.2 J, 20 Hz)	Vacuum‐assisted ureteral access sheath (FV‐UAS) was used. Continuous suction was applied through the entire process and after stones were pulverized the intrarenal end of the FV‐UAS sheath was guided into the calyces to suck out the fragments. Vacuum pressure was set at 100–300 cmH20, while intrarenal vacuum pressure was set according to surgeon. Irrigation rate was set at 65‐75 ml
Wu 2022	Retrospective	Semi‐rigid URS	82	44.9 (12.7)	24.6 (2.8)	Ho:YAG lithotripsy (0.3–0.8 J, 15–30 Hz), 200 μm fibre	Semi‐rigid URS with suction	76	48.5 (12.4)	23.9 (2)	Ho:YAG lithotripsy (0.3–0.8 J, 15–30 Hz), 200 μm fibre	The vacuum suction device is composed of a 5F ureteral catheter and a tee joint, which can be assembled into a semi‐rigid ureteroscope. The ureteral catheter is linked to the vacuum aspirator
Ding 2023	Retrospective	Flexible URS	61	55.7 (13.1)	24 (2.6)	Ho:YAG lithotripsy	Flexible URS with suction	138	57.6 (13.7)	24.6 (3.2)	Ho:YAG lithotripsy	Omnidirectional (flexible) ureteral access sheath (OD UAS) is supported by a metal wire coil to prevent collapse under pressure, the intraluminal channel is coated with Teflon (polyetrafluoroethylene) and the outside layer is coated with hydrophilic polyvinylpyrrolidone. The suction port comprises a nozzle which connects to vacuum suction, a suction switch and a watertight valve. The top 3 mm of the sheath is without a metal wire coil and is soft, while the flexible portion of the sheath is 10 cm. The suction was continuously performed with a pressure around −25 kPa, while the suction intensity could be modulated by tuning the suction switch from weak to strong
Zhai 2023	Retrospective	Semi‐rigid URS	60	46.2 (6.9)	26.2 (4.1)	Ho:YAG lithotripsy (0.6–1.0 J, 20–30 Hz), 275 μm fibre	Semi‐rigid URS with suction	60	47.2 (6.5)	25 (3.4)	Ho:YAG lithotripsy (0.6–1.0 J, 20–30 Hz), 275 μm fibre	Use of a ureteral access sheath with negative pressure suctioning
Zhai 2023	Retrospective	Semi‐rigid URS	60	46.2 (6.9)	26.2 (4.1)	Ho:YAG lithotripsy (0.6–1.0 J, 20–30 Hz), 275 μm fibre	Semi‐rigid URS with suction	60	45.7 (4.5)	24.9 (3.1)	Ho:YAG lithotripsy (0.6–1.0 J, 20–30 Hz), 275 μm fibre	Use of a negative pressure suctioning integrated semi‐rigid ureteroscope
Qian 2022	Retrospective	Flexible URS	81	50 (10.6)	24.1 (3.4)	Ho:YAG lithotripsy (12–20 W, 14–20 Hz), 200 μm fibre	Flexible URS with suction	81	50 (11.3)	24 (3)	Ho:YAG lithotripsy (12–20 W, 14–20 Hz), 200 μm fibre	Suctioning access sheath which was connected with a negative pressure pump whose pressure was maintained at 0.01 Mpa and perfusion flow was set at 50–150 ml/min
Zhang 2022	RCT	Flexible URS	30	55.7 (10.8)	25.5 (2.9)	Ho:YAG lithotripsy	Semi‐rigid URS with suction (combined with flexible standard URS)	30	53.5 (12.9)	25.2 (3.2)	Ho:YAG lithotripsy	The Soton ureteroscope was used. It comprises of 5 main components: a standard ureteroscope, a console ureteroscope, a rigid ureteral access sheath, a switch for adjusting the negative pressure and irrigation/suctioning platgorm. Perfusion exists in pulsed and continuous modes easily switched, while setting range for suction negative pressure is −25 to −4 kPa
Deng 2022	Retrospective	Mini‐PCNL	70	47.4 (7.8)	19.8 (5.7)	Ho:YAG lithotripsy (2.0 J, 25 Hz)	Flexible URS with suction	57	51.9 (10.9)	20.3 (4.4)	Ho:YAG lithotripsy (0.8 J, 20 Hz), 200 μm fibre	Patented ureteral access sheath (12/14 Fr) with pressure measuring suctioning. Perfusion flow rate was set at 50–150 ml/min, renal pressure control value at −15 to 5 mmHg, renal pressure warning value at 20 mmHg and renal pressure maximum value at 30 mmHg
AlSmadi 2019	Retrospective	Semi‐rigid URS	60	48.2 (11)	24 (1.7)	Ho:YAG or pneumatic lithotripsy	Semi‐rigid URS with suction	43	51.9 (13.2)	22.8 (1.5)	Ho:YAG or pneumatic lithotripsy	The modified ureteral access sheath used was a sheath with an additional channel at the proximal end, allowing the sheath to be connected to the suction machine. The sheath consist of a straight distal and proximal bifurcated segment. The distal segment accommodated a semi‐rigid ureteroscope. The suction was connected to the sheath and suction was set in continuous mode at 150–200 mmHg, while flow was adjusted to 60–80 ml/min

Abbreviations: BMI, body mass index (kg/m^2^); Ho:YAG, Holmium‐Yttrium‐Aluminium‐Garnet; mini‐PCNL, mini percutaneous nephrolithotripsy; NR, not reported; RCT, randomized controlled trial; SD, standard deviation; SFR, stone‐free rate; URS, ureteroscopy.

Studies including less than 10 patients, those with patients with urinary diversions, ureteral re‐implantation, previous ureteric strictures or urologic malignancy were excluded. Any mode of stone fragmentation was considered eligible (laser, mechanical, ultrasound, extraction using baskets or forceps or combination of these methods). Immediate SFR was defined according to study definition but ranged between 1 and 5 days postoperatively (Table 2), and final SFR ranged between 4 and 12 weeks (Table 2). Secondary outcomes were operative time (OR time), stone fragmentation time, lasering time, fluoroscopy time, length of hospital stay (LOS), auxiliary procedures, readmission rates, cost, post‐procedural pain scores and quality of life indicators.

**TABLE 2:** Stone characteristics and stone‐free rate definition.

Author/Year	Control arm						Experimental arm						SFR Definition	SFR assessment
	Definition	Sample size	Stone size mm (± SD)	Stone surface mm^2^ (± SD)	HU (± SD)	Stone location (number)	Definition	Sample size	Stone size mm (± SD)	Stone surface mm^2^ (± SD)	HU (± SD)	Stone location (number)		
Sur 2022	Flexible URS	8	NR	NR	926	Ureter 0 Renal 5	Flexible URS with suction	9	NR	NR	786	Ureter 0 Renal 7	No residual fragments	CT at 1 day and 4 weeks
Tang 2023	Mini‐PCNL	87	15 (5)	NR	NR	Upper ureter 87 Renal 0	Flexible URS with suction	86	16 (4)	NR	NR	Upper ureter 86 Renal 0	Fragments ≤2 mm	CT for radiolucent stones and X‐ray KUB for radiopaque stones at 1 day (immediate), 2 weeks and 4 weeks (final)
Chen 2019	Mini‐PCNL	45	NR	NR	NR	Ureter 0 Upper calyx 16 Middle calyx 16 Lower calyx 13	Flexible URS with suction	44	NR	NR	NR	Ureter 0 Upper calyx 17 Middle calyx 18 Lower calyx 11	Fragments <3 mm	X‐ray KUB at 4 weeks
Du 2019	Semi‐rigid URS	60	21.4 (3.6)	NR	985 (227)	Upper ureter 20 Mid ureter 13 Distal ureter 27 Renal 0	Semi‐rigid URS with suction	62	21.9 (4.9)	NR	1023 (215)	Upper ureter 21 Mid ureter 15 Distal ureter 26 Renal 0	Fragments ≤4 mm	X‐ray KUB at 4 weeks
Lai 2020	Mini‐PCNL	56	38.2 (5.4)	729 (83.7)	845 (240)	Ureter 0 Renal pelvis 56 Upper calyx 22 Middle calyx 28 Lower calyx 22	Flexible URS with suction	28	35.3 (6.3)	676.1 (42.2)	894 (232)	Ureter 0 Renal pelvis 28 Upper calyx 13 Middle calyx 15 Lower calyx 11	No residual fragments	CT at 1 day and 12 weeks
Zhu 2018	Flexible URS	165	17.4 (4.7)	NR	1023 (175)	Ureter NR Renal pelvis 23 Upper calyx 18 Middle calyx 35 Lower calyx 42	Flexible URS with suction	165	18.2 (5.2)	NR	1049 (196)	Ureter NR Renal pelvis 29 Upper calyx 14 Middle calyx 27 Lower calyx 40	Fragments <2 mm	X‐ray KUB at 1 day and X‐ray or CT KUB at 4 weeks
Zhang 2021	Semi‐rigid URS	50	12.5 (4.9)	NR	665 (310)	Upper ureter 50 Renal 0	Semi‐rigid URS with suction	56	13.9 (4.7)	NR	709 (344)	Upper ureter 56 Renal 0	No residual fragments	CT at 3–5 days and 4 weeks
Zhang 2021	Flexible URS	54	12.7 (5.5)	NR	684 (376)	Upper ureter 54 Renal 0	Semi‐rigid URS with suction	56	13.9 (4.7)	NR	709 (344)	Upper ureter 56 Renal 0	No residual fragments	CT at 3–5 days and 4 weeks
Lechevallier 2003	Semi‐rigid URS	14	NR	NR	NR	NR	Semi‐rigid URS with suction	11	NR	NR	NR	NR	NR	XR KUB at the end of procedure
Huang 2023	Flexible URS	103	17 (5)	NR	NR	NR	Flexible URS with suction	103	17 (6)	NR	NR	NR	Fragments <3 mm	CT at 1 day and 4 weeks
Wu 2022	Semi‐rigid URS	82	NR	157 (35)	916 (81)	Upper ureter 82 Renal 0	Semi‐rigid URS with suction	76	NR	165 (33)	938 (85)	Upper ureter 76 Renal 0	No residual fragments	X‐ray KUB at 1 day and 4 weeks
Ding 2023	Flexible URS	61	13.4 (5.2)	NR	715 (341)	Upper ureter 13 Renal pelvis 35 Upper calyx 2 Middle calyx 5 Lower calyx 29	Flexible URS with suction	138	13 (6.9)	NR	752 (429)	Upper ureter 26 Renal pelvis 98 Upper calyx 3 Middle calyx 11 Lower calyx 64	Fragments <2 mm	CT at 4 weeks
Zhai 2023	Semi‐rigid URS	60	NR	132 (25)	912 (53)	Upper ureter 13 Mid ureter 17 Distal ureter 30 Renal 0	Semi‐rigid URS with suction	60	NR	137 (27)	907 (64)	Upper ureter 12 Mid ureter 18 Distal ureter 30 Renal 0	Fragments <4 mm	4 weeks
Zhai 2023	Semi‐rigid URS	60	NR	132 (25)	912 (53)	Upper ureter 13 Mid ureter 17 Distal ureter 30 Renal 0	Semi‐rigid URS with suction	60	NR	136 (25)	897 (94)	Upper ureter 12 Mid ureter 28 Distal ureter 20 Renal 0	Fragments <4 mm	4 weeks
Qian 2022	Flexible URS	81	20 (4.5)	NR	NR	Ureter 0 Renal 81	Flexible URS with suction	81	19.7 (4.5)	NR	NR	Ureter 0 Renal 0	Fragments <4 mm	X‐ray KUB at 1 day and X‐ray/CT KUB at 4 weeks
Zhang 2022	Flexible URS	30	18.5 (5.6)	NR	NR	Ureter 0 Renal 30	Semi‐rigid URS with suction (combined with fURS)	30	18.2 (5.3)	NR	NR	Ureter 0 Renal 30	Fragments ≤3 mm	X‐ray KUB at 1 week
Deng 2022	Mini‐PCNL	70	24.9 (7.9)	NR	NR	Ureter 0 Renal 70	Flexible URS with suction	57	23.1 (6.5)	NR	NR	Ureter 0 Renal 57	Fragments <2 mm	CT at 4 and 12 weeks
AlSmadi 2019	Semi‐rigid URS	60	14.9 (1.8)	NR	777 (120)	Upper ureter 60 Renal 0	Semi‐rigid URS with suction	43	12.7 (1.2)	NR	896 (70)	Upper ureter 43 Renal 0	Fragments ≤3 mm	X‐ray KUB at 1–2 days and CT at 4 weeks

Abbreviations: fURS, flexible ureteroscopy; KUB, kidneys‐ureters‐bladder; mini‐PCNL, mini percutaneous nephrolithotripsy; NR, not reported; RCT, randomized controlled trial; URS, ureteroscopy.

Data extraction was performed using a preset Excel sheet for baseline study and patient characteristics (year, centre, inclusion/exclusion criteria, age, body mass index‐BMI, sample size, stone size/surface/volume and technical characteristics regarding the operation and suction technique) and primary and secondary outcomes. Two authors (L.T. and P.J.) independently extracted data, and in case of disagreement between the two authors, a third author (R.G.) was advised to reach consensus.

### 2.3 Risk of bias assessment

Two authors (L.T. and P.J.) assessed the risk of bias independently using the Cochrane risk of bias 2 for randomized controlled trials (RCTs) and the risk of bias for non‐randomized trials tool (ROBINS‐I).^10^, ^11^ In case of disagreement between the two authors, a third author (R.G.) was advised to reach consensus.

### 2.4 Publication bias assessment

Publication bias was assessed after visual inspection of Funnel plots for outcomes on which at least 10 studies were reporting results.

### 2.5 Certainty of evidence

The Grading of Recommendation Assessment, Development and Evaluation (GRADE) tool was used to evaluate each of the outcomes for certainty of evidence.^12^ The levels of evidence were *very low*, *low*, *moderate* or *high*; each evidence certainty was ranked as *high* for RCTs and *low* for observational studies initially, while after evaluating limitations of risk of bias, imprecision, inconsistency, indirectness and publication bias, the level of certainty was downrated.^12^

### 2.6 Statistical analysis

Statistical analysis was performed in R (R statistical software; R Foundation for Statistical Computing, Vienna, Austria, version 4.3.1). Meta‐analyses were performed using the meta and metafor packages, with plots made using these along with the forestplot package. For continuous variables, the mean difference or standardized mean difference was used with the corresponding 95% confidence intervals (CIs). Relative risks (RRs) were used to estimate binary outcomes with corresponding 95% CIs. For missing data, no imputation was performed. A priori, a fixed effects model was used in case of low heterogeneity (I^2^ < 50%) and random effects model for high heterogeneity (I^2^ > 50%). Heterogeneity between studies was assessed using the Chi‐squared Q test and I^2^ statistics. If I^2^ > 50% and/or Chi‐squared < 0.10, substantial heterogeneity was considered. Formulas by Sterne et al.^10^, ^11^ were used to transform medians and interquartile ranges to means and standard deviations when necessary. Subanalyses for study design (RCT only) and type of intervention (semirigid URS, flexible URS and PCNL) were also conducted. For all outcomes, heterogeneity was assessed using I^2^, tau^2^ and Cochran's Q. We present risk ratios, according to the random or fixed effects model as above. Publication bias was assessed via visual inspection of Funnel plots. In analyses with *n* > 2 studies, we performed trim and fill analyses to statistically assess for publication bias. Adjusted values for the trim and fill analysis are presented along with the calculated number of missing studies. We present forest and funnel plots, along with heterogeneity statistics (I^2^, Cochran's Q and Tau^2^) if the number of studies included was >2. All analyses are available in Appendix S2, which details the full code.

## 3 RESULTS

**FIGURE 1:**
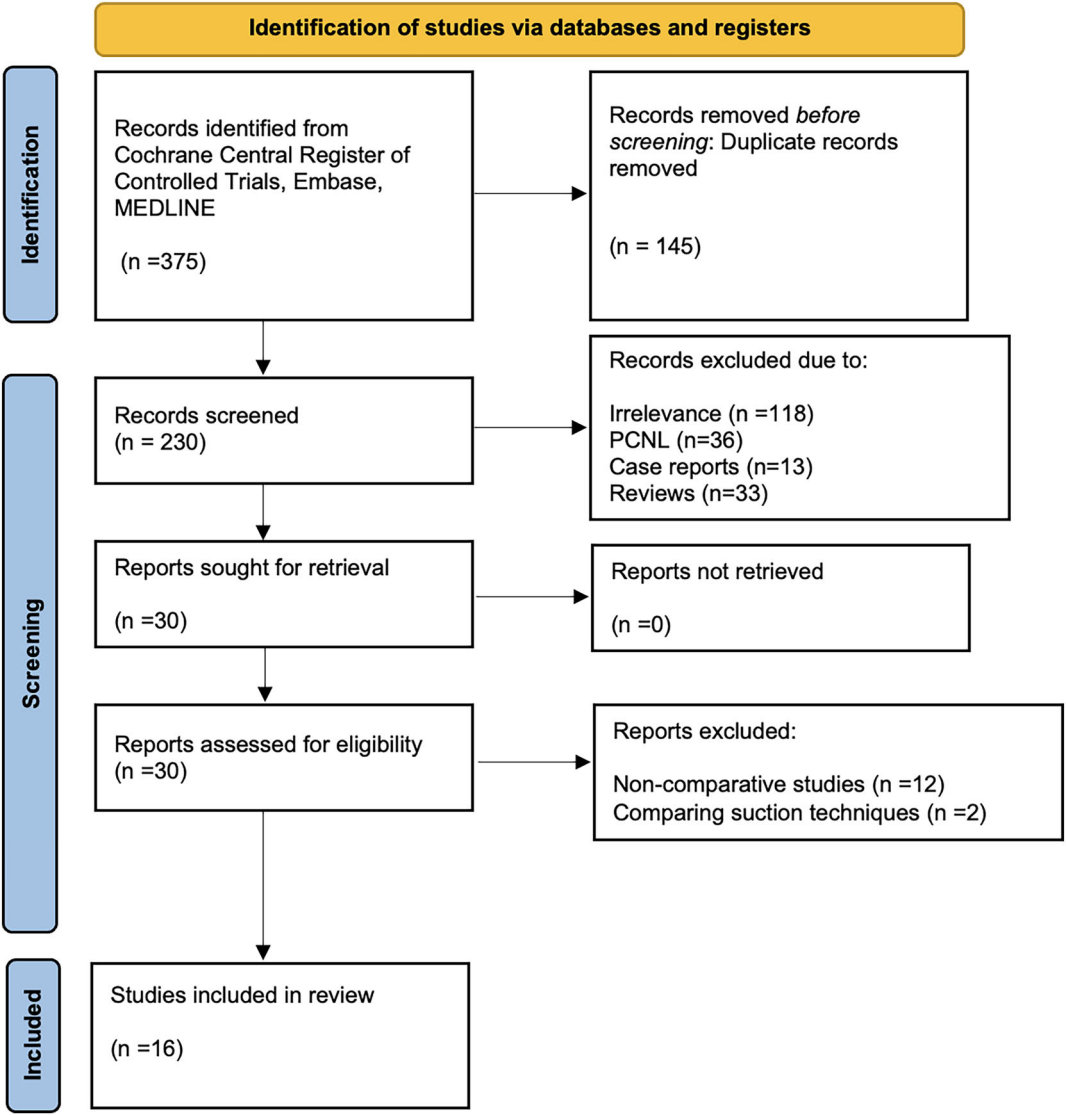
PRISMA flow diagram.

Literature search in 3 databases revealed 230 studies after removal of 145 duplicates. After initial screening by title and abstract, 118 were excluded due to irrelevance, 36 due to analysing PCNL only data, while 13 case reports and 33 reviews were also excluded, leaving 30 studies to be screened by reading the full text. Twelve further studies were excluded due to non‐comparative design and two because they were comparing two different suction techniques. Finally, 16 comparative studies (5 RCTs^13^, ^14^, ^15^, ^16^, ^17^ and 11 observational^18^, ^19^, ^20^, ^21^, ^22^, ^23^, ^24^, ^25^, ^26^, ^27^, ^28^) were included in qualitative and quantitative analysis, including 1086 patients in control and 1109 patients in suction groups (2 of the studies reported 3 groups,^21^, ^25^ thus in total 18 groups of comparison were extracted). Figure 1 shows the PRISMA flow diagram for study selection. The risk of bias assessment for all studies can be found in Tables S1 and S2, while certainty of evidence for all outcomes based on GRADE system in Table S3. Publication bias assessed by visual inspection of Funnel plots revealed high risk for all outcomes. All analyses are detailed in Appendix S2 including heterogeneity statistics.

### 3.1 Overall comparative analysis

**FIGURE 2:**
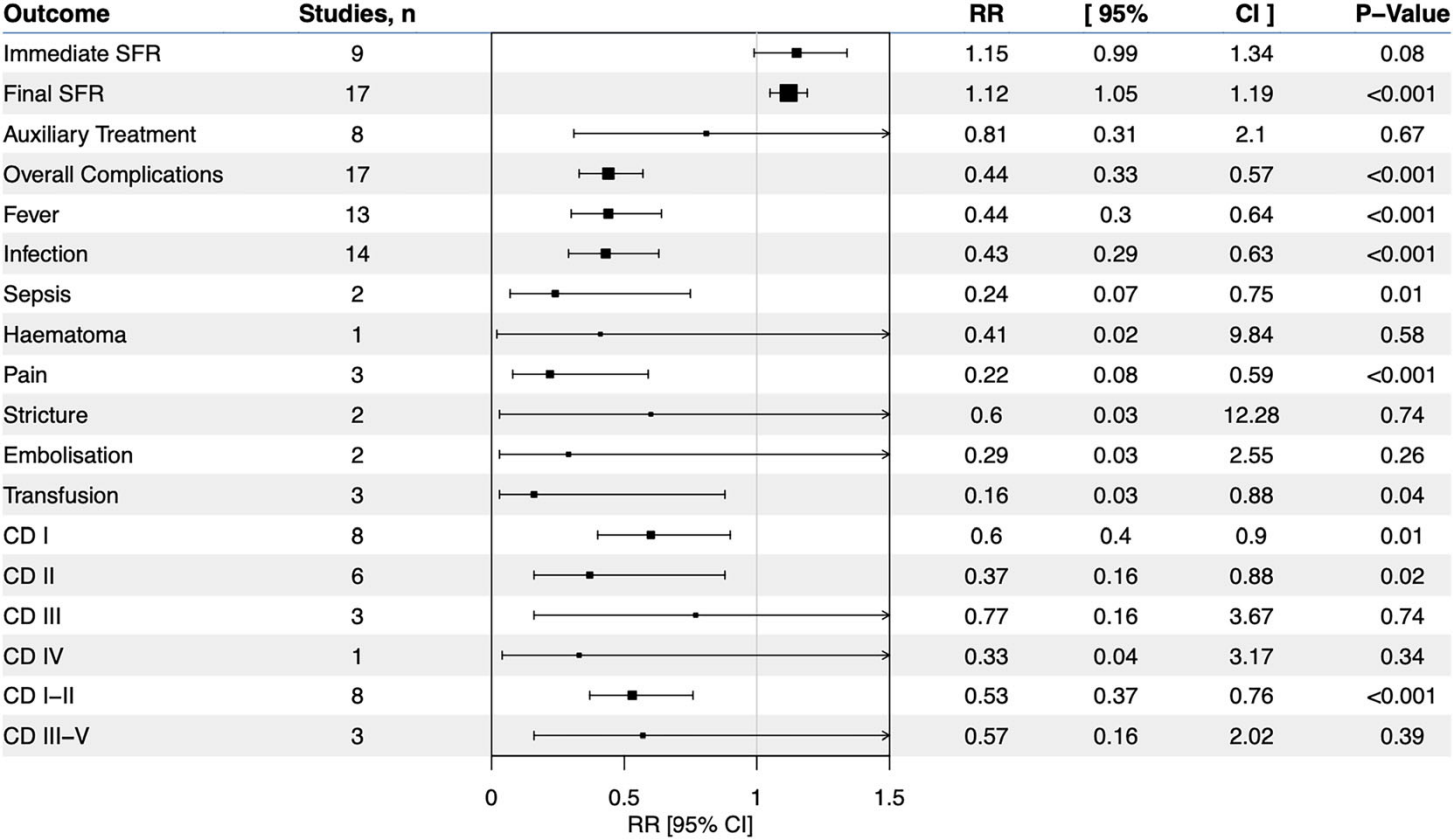
Forest plot for overall comparative analysis of binary outcomes.

Pooled analysis of nine studies revealed a non‐significant difference on immediate SFR between suction and non‐suction groups (RR = 1.15, 95% C.I.: 0.99–1.34, p = 0.08), while analysis of 17 study arms on final SFR showed a significant improvement in favour of suction (RR = 1.12, 95% C.I.: 1.05–1.19, p < 0.001). Auxiliary treatment did not differ significantly. Overall complications as analysed in 17 study arms were significantly lower in suction group (RR = 0.44, 95% C.I.: 0.33–0.57, p < 0.001). Fever (RR = 0.44, 95% C.I.: 0.3–0.64, *p* < 0.001), infections (RR = 0.43, 95% C.I.: 0.29–0.63, *p* < 0.001), sepsis (RR = 0.24, 95% C.I.: 0.07–0.75, *p* = 0.01), pain (RR = 0.22, 95% C.I.: 0.08–0.59, *p* < 0.001) and transfusion rates (RR = 0.16, 95% C.I.: 0.03–0.88, *p* = 0.04) were significantly lower in suction group, while no significant differences were detected regarding ureteral stricture formation and embolization. When using Clavien‐Dindo classification, grades I (RR = 0.6, 95% C.I.:0.4–0.9, *p* = 0.01) and II (RR = 0.37, 95% C.I.: 0.16–0.88, *p* = 0.02) were significantly lower in suction group (for combined grades I and II, RR = 0.53, 95% C.I.: 0.37–0.76, *p* < 0.001), while grades III and IV did not differ significantly. Summary forest plots are shown in Figure 2.

Operative time and pain when reported as VAS scores did not differ significantly between the two groups, but LOS was significantly shorter by 1.09 days in suction group (mean difference—1.09, 95% C.I.: −1.9 to −0.28, *p* = 0.01). Summary forest plots are shown in Figure S1. See Appendix S2, section 4, for individual outcome forest plots and heterogeneity statistics.

### 3.2 Sensitivity analysis on RCTs only

Pooled analysis of five RCTs revealed no statistically significant difference in both immediate and final SFR, auxiliary treatments, sepsis, stricture formation and Clavien‐Dindo grade I or II complications, although only a single RCT reported results for auxiliary treatments, sepsis and stricture formation. Overall complications (RR = 0.2, 95% C.I.: 0.09–0.41, *p* < 0.001), infections (RR = 0.22, 95% C.I.: 0.08–0.61, *p* < 0.001) and fever (RR = 0.22, 95% C.I.: 0.07–0.63, *p* < 0.001) were significantly lower the suction group. OR time and LOS did not differ significantly between groups. One study analysing pain and VAS score showed results in favour of suction group (RR = 0.16, 95% C.I.: 0.04–0.67, *p* = 0.01 and MD = −0.34, 95% C.I.: −0.65 to −0.03, *p* = 0.03). Summary forest plots are shown in Figures S2 and S3. See Appendix S2, section 6, for individual outcome forest plots and heterogeneity statistics.

### 3.3 Subgroup analysis on semi‐rigid URS only

Pooled analysis of studies comparing suction versus no suction in semi‐rigid URS showed significantly improved final (RR = 1.16, 95% C.I.: 1.07–1.25, *p* < 0.001) but not immediate SFR in favour of suction group, while rates of auxiliary treatment were also similar. Overall complications (RR = 0.45, 95% C.I.: 0.25–0.83, *p* = 0.01), fever (RR = 0.29, 95% C.I.: 0.12–0.72, *p* = 0.01) and infection (RR = 0.29, 95% C.I.: 0.12–0.72, *p* = 0.01) were significantly lower in suction group, but pain (one study), ureteral stricture formation (one study) and Clavien‐Dindo grades I and II complications were similar between the two groups. LOS was significantly lower in suction group by 0.29 days (MD = −0.29, 95% C.I.: −0.55 to −0.03, *p* = 0.03), but OR time was similar. Summary forest plots are shown in Figures S4 and S5. See Appendix S2, section 8, for individual outcome forest plots and heterogeneity statistics.

### 3.4 Subgroup analysis on flexible URS/RIRS only

Pooled analysis of studies comparing suction versus no suction in flexible URS/RIRS revealed significantly improved final SFR (RR = 1.2, 95% C.I.: 1.1–1.3, *p* < 0.001) but not immediate SFR or auxiliary treatments (one study), in favour of suction group. Overall complications (RR = 0.44, 95% C.I.: 0.29–0.67, *p* < 0.001), fever (RR = 0.38, 95% C.I.: 0.23–0.65, *p* < 0.001), infections (RR = 0.38, 95% C.I.: 0.23–0.64, *p* < 0.001), sepsis at one study (RR = 0.27, 95% C.I.: 0.08–0.96, *p* = 0.04), Clavien II (RR = 0.28, 95% C.I.: 0.09–0.88, *p* = 0.03) and I‐II (RR = 0.48, 95% C.I.: 0.29–0.8, *p* < 0.001) were significantly lower in suction group, while ureteral stricture formation (one study), Clavien‐Dindo I, III, IV and III‐IV (one study) did not differ significantly between the two groups. OR time and LOS did not differ significantly, while VAS score was lower in suction group, as reported in one study. Summary forest plots are shown in Figures S6 and S7. See Appendix S2, section 10, for individual outcome forest plots and heterogeneity statistics.

### 3.5 Subgroup analysis on mini‐PCNL versus flexible URS/RIRS

Pooled analysis comparing suction in flexible URS/RIRS versus mini‐PCNL revealed no significant differences in both immediate and final SFR, auxiliary treatments, infectious complications (infections, fever and sepsis), hematoma formation (one study), embolization and Clavien‐Dindo I, II, III and IV. However, overall complications (RR = 0.42, 95% C.I.: 0.21–0.81, *p* = 0.01), pain (RR = 0.21, 95% C.I.: 0.07–0.6, *p* < 0.001) and transfusion rates (RR = 0.16, 95% C.I.: 0.03–0.88, *p* = 0.04) were significantly lower in flexible URS/RIRS with the use of suction. OR time did not differ significantly between the two groups, while LOS was significantly shorter by 2.89 days in flexible URS/RIRS group (MD = −2.89, 95% C.I.: −3.55 to −2.23, *p* < 0.001). VAS score in one study was also significantly lower in flexible URS/RIRS group. Summary forest plots are shown in Figures S8 and S9. See Appendix S2, section 12, for individual outcome forest plots and heterogeneity statistics.

## 4 DISCUSSION

The two outcomes synthesizing the success of URS/RIRS are SFR and complication rates; suction application seems to be associated with both increased SFRs (mainly final) and substantially reduced complication rates, especially those related to infections. More specifically, final SFR was improved by 12% in suction group, while infectious complications (sepsis, infection and fever) were reduced by 56–76% in suction group when all studies were analysed. In RCTs subgroup analysis, infectious complication rates were also decreased, but SFRs and sepsis rates were similar between suction and non‐suction groups. In further subgroup analysis accordingly to specific types of treatment, in semi‐rigid and flexible URS/ RIRS, final SFRs were improved by 16–20% in suction groups, despite immediate SFRs being similar. Also, in both subanalyses, infectious and overall complications were significantly reduced in suction groups. However, when mini‐PCNL was compared to suction‐aided flexible URS/ RIRS, no significant differences were detected regarding SFRs, auxiliary treatments and infectious complications; reduced rates of overall complications, pain and transfusion rates were seen in suction URS/ RIRS. These findings are probably explained due to suction effectiveness in reducing intrarenal pressure, which can rise during URS/ RIRS from irrigation fluids and lead to pyelovenous and pyelolymphatic backflow and entrance of pathogenic bacteria in systemic circulation.^29^ High‐power Holmium and Thulium Fibre laser (TFL) have enhanced the dusting mode of stone disintegration, but vision can be obscured from this ‘cloud of dust’, thus increasing operative time and risk for injuries; when suction is applied, several reports support that vision is not compromised, accounting partially for the reported reduced operative times.^29^ Nevertheless, our pooled analysis did not reveal any significant improvement in terms of operative time in any of the groups. The absence of observed significant differences between mini‐PCNL and suction aided URS/RIRS can potentially be explained by the inherent suction component of mini‐PCNL technique itself, due to the ‘Venturi effect’ generated by the dynamic fluid property at the tip of the nephroscope.^30^

The first reported RCT evaluating suction in RIRS was by Lechevallier et al.,^16^ 20 years ago, who showed that OR time was significantly reduced, while SFR was higher in suction group (92% vs. 69%, *p* = 0.048). Du et al.^15^ and Chen et al.^18^ designed further RCTs to assess suction via a patented perfusion and suction platform in ureteric and renal stones, respectively. The system was connected to a patented ureteral access sheath and was able to maintain a low/negative intrapelvic pressure (5 to −15 mm Hg) with perfusion set at 50–150 ml/min and concluded that its application is effective and safe. A new semi‐rigid ureterorenoscope, the Soton ureterorenoscope, was used by Zhang et al.^17^ in comparison with standard ureteroscopy; authors reported improved vision and appropriate control of intrapelvic pressure, leading to reduction of operative time. Aspiration systems have been described also via the scope used for lithotripsy, the so‐called direct in‐scope suction (DISS) technique, as described in the original study.^31^ The great advantage of DISS is that the system comprises of a reproducible idea on every ureteroscope. Specifically, two simple three‐way stoppers are attached and allow connection to irrigation and suction tubes according to surgeons' preference during surgery.^31^ In the study by Gauhar et al.,^31^ the DISS technique compared to a traditional suction access sheath led to reduction of LOS and similar residual fragments rates in the cost of increased operative time. Besides ureteral access sheaths and ureteroscopes, ureteral catheters can also serve as a mean of suction application during URS/RIRS. In the study by Wu et al.,^23^ a modified 5Fr ureteral catheter was connected via a T‐shaped joint to a vacuum system and then introduced into a semi‐rigid ureterorenoscope. This system was tested in patients with impacted ureteral stones in comparison with conventional URS and showed that operative time was significantly lower (38.2 min vs. 46.7 min, *P* < 0.001), fever rates lower (3.9% vs. 14.6%, *p* = 0.022) and higher early SFR (88.2% vs. 72%, *p* = 0.011).^23^ In our pooled analysis, immediate SFR was similar, final SFR higher, operative time similar and infectious complications significantly reduced in the group of suction‐aided, semi‐rigid ureteroscopy. An innovative system was described in the pilot study by Sur et al.,^13^ who used the steerable ureteroscopic renal evacuation catheter (SURE), which was connected to a ureteral access sheath after completing lithotripsy and was guided by fluoroscopy to all calyces in order to apply suction and remove fragmented stone particles. Authors detected a final SFR that was significantly higher in the SURE group compared to basket extraction group (100% vs. 75%, *p* = 0.20), although number of patients was small.^13^

Flexible URS/RIRS is an already costly procedure and adding another technological innovation may reasonably increase the associated costs. Not many studies reported the monetary burden of this technology, but in two studies, Du et al.^15^ and Zhang et al.^21^ did not show any increased costs; on the contrary, Zhang et al.^21^ reported that suction group was associated with significantly lower costs (2622.6 US dollars) compared to rigid URS (2883.6 US dollars) and flexible RIRS (3724.4 US dollars). This was attributed to skipping the use of baskets and forceps and to the reusable design of suction equipment. The trend observed on reduced LOS in suction group may also contribute to cost reduction. Another important limitation of this equipment is its availability since many of the described systems are patented and may not be easy to be acquired. Some of these systems need additional use of fluoroscopy, which may add more hazardous exposure to both patients and operating room staff.^32^, ^33^

This study is not devoid of limitations. First of all, both observational and randomized studies were collected due to paucity of data derived from RCTs; however, subgroup and sensitivity analysis was also performed for RCTs showing similar results excluding the SFRs and specific types of complications. Existing RCTs analyse small sizes and different stone location/types of suction; thus, we have chosen to include also overall analysis including observational studies. Suction and irrigation settings were also widely variable, contributing to the heterogeneity of the results among studies, while definition and assessment (timepoint and examination used) of SFRs and complications were also variable. Endourological equipment is continuously enriched, and endourologists are blessed and cursed at the same time to have a vast number of choices regarding every step of ureteroscopy; to name some, guidewires, stents, ureteroscopes, access sheaths, laser types and settings, graspers and baskets are only the main categories. Adding to this complexity, several suction devices are already available and tested: semirigid ureteroscopes with incorporated suction, ureteral access sheaths with suction, which can be steerable or not, several suction techniques such as direct‐in‐scope‐technique or the flexible and navigable ureteral suction sheath (FANS). The comparative studies found in literature were heterogeneous regarding suction type, stone type/size and location, technique used for comparison (miniPCNL or non‐suction ureteroscopy), pressure used for suction and irrigation. In order to be able to incorporate suction technology for specific indications, we certainly need sounder and more robust comparative RCTs for specific patient populations and specific suction technology. Despite these limitations, this is the first systematic review with a meta‐analysis on suction use for URS/RIRS and may serve as the basis for designing proper clinical trials to define the indications, protocols, safety and effectiveness of these systems, since paucity of existing data prevents us from comparing which suction mechanism has the best possible potential to improve RIRS in future.

## 5 CONCLUSIONS

Application of suction via ureteral access sheaths, ureteral catheters or scopes may provide improved SFRs, reduced overall and infectious complication rates, along with a reduction in length of hospital stay. Further randomized studies are needed to validate these findings and standardize indications and protocols.

## AUTHOR CONTRIBUTIONS

**Lazaros Tzelves**: Conception/design of the work; acquisition/analysis/interpretation of data; drafting the manuscript. **Robert Geraghty**: Acquisition/analysis/interpretation of data; critically reviewing the manuscript for important intellectual content. **Patrick Juliebø‐Jones**: acquisition/analysis/interpretation of data; critically reviewing the manuscript for important intellectual content. **Yuhong Yuan**: Acquisition/analysis/interpretation of data; critically reviewing the manuscript for important intellectual content. **Konstantinos Kapriniotis**: Acquisition/analysis/interpretation of data; critically reviewing the manuscript for important intellectual content. **Daniele Castellani**: Acquisition/analysis/interpretation of data; critically reviewing the manuscript for important intellectual content. **Vineet Gauhar**: Acquisition/analysis/interpretation of data; critically reviewing the manuscript for important intellectual content. **Andreas Skolarikos**: Acquisition/analysis/interpretation of data; critically reviewing the manuscript for important intellectual content. **Bhaskar Somani**: Conception/design of the work; acquisition/analysis/interpretation of data; critically reviewing the manuscript for important intellectual content.

## CONFLICT OF INTEREST STATEMENT

L. Tzelves is an associate member of EAU Guidelines Panel on Urolithiasis, a YAU Member in Endourology Group and a member of ESU Working Group on PCNL. R. Geraghty is an associate member of EAU Guidelines Panel on Urolithiasis. P. Juliebø‐Jones is a YAU Member in Endourology Group. Y. Yuan is a member of EAU Guidelines Methods Office. G. Vineet is a clinical and education research consultant for PUSEN, BIORAD, CLEARPETRA, INNOVEX, ROCAMED and BD and a member of European Section of Urolithiasis (EULIS), EAU Guidelines Dissemination committee; a board member of endourology academy; and a board member of kidney stone academy. A. Skolarikos is a member of EAU Guidelines panel on Urolithiasis and European Section of Urolithiasis (EULIS). B. Somani is a member of EAU Guidelines panel on Urolithiasis and European Section of Urolithiasis (EULIS).

## Supporting information


**Appendix S1.** Supporting Information

**Appendix S2.** Supporting Information

**Table S1.** Risk of bias for randomized controlled trials (Risk of Bias 2 tool)
**Table S2**. Risk of bias for non‐randomized comparative trials (ROBINS‐I tool)

**Table S3.** Certainty of the evidence for each outcomes based on the GRADE approach

**Figure S1.** Forest plot for overall comparative analysis of continuous outcomes

**Figure S2.** Forest plot for analysis of binary outcomes in RCTs

**Figure S3.** Forest plot for analysis of continuous outcomes in RCTs

**Figure S4.** Forest plot for analysis of binary outcomes in semi‐rigid URS

**Figure S5.** Forest plot for analysis of continuous outcomes in semi‐rigid URS

**Figure S6.** Forest plot for analysis of binary outcomes in flexible URS/RIRS

**Figure S7.** Forest plot for analysis of continuous outcomes in flexible URS/RIRS

**Figure S8.** Forest plot for analysis of binary outcomes in flexible URS/RIRS vs mini‐PCNL

**Figure S9.** Forest plot for analysis of continuous outcomes in flexible URS/RIRS vs mini‐PCNL
