# Supplementary material for: Suction use in ureterorenoscopy: A systematic review and meta‐analysis of comparative studies
Source: BJUI Compass. 2024 Jul 8;5(10):895–912. doi: 10.1002/bco2.408 (PMC11479806; doi:10.1002/bco2.408)
Supplement: Supplementary file 1 — Appendix S1. Supporting Information [file BCO2-5-895-s002.docx]

**Database: EBM Reviews - Cochrane Central Register of Controlled Trials <October 2023>, Embase <1974 to 2023 November 10>, OVID Medline Epub Ahead of Print, In-Process & Other Non-Indexed Citations, Ovid MEDLINE(R) Daily and Ovid MEDLINE(R) 1946 to Present**
**Search Strategy:**
**1**  Ureteroscopy/ (17306)
**2**  (Ureteroscop* or Ureterorenoscop* or pyeloureteroscop* or ureteropyeloscop*).tw,kw,kf. (22668)
**3**  (URS or fURS).tw,kw,kf. (7749)
**4**  or/1-3 (29584)
**5**  Nephrostomy, Percutaneous/ use ppez,cctr (5458)
**6**  percutaneous nephrolithotomy/ or percutaneous nephrolithotripsy/ use oemezd (11428)
**7**  (percutaneous adj (nephrolithotripsy or nephro-lithotripsy or nephrolithotomy)).tw,kw,kf. (16151)
**8**  (PCNL or PNL).tw,kw. (13509)
**9**  ((retrograde intrarenal or retrograde Intra-Renal) adj3 (surger* or surgical or endoscop*)).tw,kw,kf. (3030)
**10**  RIRS.tw,kw,kf. (2838)
**11**  or/5-10 (25650)
**12**  suction/ (27171)
**13**  (suction or suck*).tw,kw,kf. (100895)
**14**  vacuum/ (24945)
**15**  vacuum.tw,kw,kf. (94385)
**16**  or/12-15 (207672)
**17**  4 and 16 (220)
**18**  11 and 16 (468)
**19**  17 or 18 (582)
**20**  limit 19 to english language (562)
**21**  conference abstract.pt. (4945357)
**22**  20 not 21 (387)
**23**  case report/ or case report.ti. (3278734)
**24**  22 not 23 (375)
**25**  remove duplicates from 24 (230)
