# Supplementary material for: Suction use in ureterorenoscopy: A systematic review and meta‐analysis of comparative studies
Source: BJUI Compass. 2024 Jul 8;5(10):895–912. doi: 10.1002/bco2.408 (PMC11479806; doi:10.1002/bco2.408)

Suction in Ureteroscopy

R Geraghty

2024-01-18

Contents

1 Setup **17**
1.1 Data Encoding . . . 19
1.2 Make new columns combining CD . . . 26

2 Data Exploration **27**

3 Demographic information **36**
3.1 Number of Patients . . . 36
3.2 Number of Surgeons . . . 37
3.3 Number of Male Patients . . . 38
3.4 Comparisons - type of intervention . . . 39
3.5 Age . . . 40
3.6 BMI . . . 41
3.7 HU . . . 42
3.8 Size Metrics . . . 43
3.8.1 Size (mm) - missing data removed . . . 43
3.8.2 Area (mmˆ2) - missing data removed . . . 44
3.8.3 Volume (mmˆ3) - missing data removed . . . 45
3.9 Stone Location . . . 46
3.9.1 Distal Ureter . . . 46
3.9.2 Mid Ureter . . . 47
3.9.3 Upper Ureter . . . 48
3.9.4 Renal Pelvis . . . 49
3.9.5 Lower Calyx . . . 50
3.9.6 middle Calyx . . . 51
3.9.7 Upper Calyx . . . 52

4 Overall Meta-Analysis Outcomes **53**
4.1 Immediate SFR . . . 53
4.1.1 Meta-analysis . . . 53
4.1.2 Forest plot . . . 54
4.1.3 Trim and Fill . . . 55
4.1.4 Funnel plot . . . 56
4.1.5 Baujat . . . 57
4.1.6 Leave one out . . . 58
4.2 Final SFR . . . 59
4.2.1 Meta-analysis . . . 59
4.2.2 Forest plot . . . 60
4.2.3 Trim and Fill . . . 61
4.2.4 Funnel plot . . . 63
4.2.5 Baujat . . . 64

4.2.6 Leave one out . . . 65
4.3 OR time . . . 66
4.3.1 Meta-analysis . . . 66
4.3.2 Forest plot . . . 67
4.3.3 Trim and Fill . . . 68
4.3.4 Funnel plot . . . 69
4.3.5 Baujat . . . 70
4.3.6 Leave one out . . . 71
4.4 Auxiliary Treatments . . . 72
4.4.1 Meta-analysis . . . 72
4.4.2 Forest plot . . . 73
4.4.3 Trim and Fill . . . 74
4.4.4 Funnel plot . . . 75
4.4.5 Baujat . . . 76
4.4.6 Leave one out . . . 77
4.5 VAS . . . 78
4.5.1 Meta-analysis . . . 78
4.5.2 Forest plot . . . 79
4.5.3 Trim and Fill . . . 80
4.5.4 Funnel plot . . . 81
4.5.5 Baujat . . . 82
4.5.6 Leave one out . . . 83
4.6 LoS . . . 84
4.6.1 Meta-analysis . . . 84
4.6.2 Forest plot . . . 85
4.6.3 Trim and Fill . . . 86
4.6.4 Funnel plot . . . 87
4.6.5 Baujat . . . 88
4.6.6 Leave one out . . . 89
4.7 Complications - All . . . 90
4.7.1 Meta-analysis . . . 90
4.7.2 Forest plot . . . 91
4.7.3 Trim and Fill . . . 92
4.7.4 Funnel plot . . . 93
4.7.5 Baujat . . . 94
4.7.6 Leave one out . . . 95
4.8 Fever . . . 96
4.8.1 Meta-analysis . . . 96
4.8.2 Forest plot . . . 97
4.8.3 Trim and Fill . . . 98
4.8.4 Funnel plot . . . 99
4.8.5 Baujat . . . 100
4.8.6 Leave one out . . . 101
4.9 Infections . . . 102
4.9.1 Meta-analysis . . . 102
4.9.2 Forest plot . . . 103
4.9.3 Trim and Fill . . . 104
4.9.4 Funnel plot . . . 105
4.9.5 Baujat . . . 106
4.9.6 Leave one out . . . 107
4.10 Sepsis . . . 108
4.10.1 Meta-analysis . . . 108
4.10.2 Forest plot . . . 109
4.10.3 Trim and Fill . . . 110

4.10.4 Funnel plot . . . 111
4.10.5 Baujat . . . 112
4.10.6 Leave one out . . . 113
4.11 Abscess . . . 114
4.11.1 Meta-analysis . . . 114
4.11.2 Forest plot . . . 115
4.11.3 Trim and Fill . . . 116
4.11.4 Funnel plot . . . 117
4.11.5 Baujat . . . 118
4.11.6 Leave one out . . . 119
4.12 Haematoma . . . 120
4.12.1 Meta-analysis . . . 120
4.12.2 Forest plot . . . 121
4.12.3 Trim and Fill . . . 122
4.12.4 Funnel plot . . . 123
4.12.5 Baujat . . . 124
4.12.6 Leave one out . . . 125
4.13 Pain . . . 126
4.13.1 Meta-analysis . . . 126
4.13.2 Forest plot . . . 127
4.13.3 Trim and Fill . . . 128
4.13.4 Funnel plot . . . 129
4.13.5 Baujat . . . 130
4.13.6 Leave one out . . . 131
4.14 Stricture . . . 132
4.14.1 Meta-analysis . . . 132
4.14.2 Forest plot . . . 133
4.14.3 Trim and Fill . . . 134
4.14.4 Funnel plot . . . 135
4.14.5 Baujat . . . 136
4.14.6 Leave one out . . . 137
4.15 Embolisation required . . . 138
4.15.1 Meta-analysis . . . 138
4.15.2 Forest plot . . . 139
4.15.3 Trim and Fill . . . 140
4.15.4 Funnel plot . . . 141
4.15.5 Baujat . . . 142
4.15.6 Leave one out . . . 143
4.16 Transfusion . . . 144
4.16.1 Meta-analysis . . . 144
4.16.2 Forest plot . . . 145
4.16.3 Trim and Fill . . . 146
4.16.4 Funnel plot . . . 147
4.16.5 Baujat . . . 148
4.16.6 Leave one out . . . 149
4.17 Clavien I . . . 150
4.17.1 Meta-analysis . . . 150
4.17.2 Forest plot . . . 151
4.17.3 Trim and Fill . . . 152
4.17.4 Funnel plot . . . 153
4.17.5 Baujat . . . 154
4.17.6 Leave one out . . . 155
4.18 Clavien II . . . 156
4.18.1 Meta-analysis . . . 156

4.18.2 Forest plot . . . 157
4.18.3 Trim and Fill . . . 158
4.18.4 Funnel plot . . . 159
4.18.5 Baujat . . . 160
4.18.6 Leave one out . . . 161
4.19 Clavien III . . . 162
4.19.1 Meta-analysis . . . 162
4.19.2 Forest plot . . . 163
4.19.3 Trim and Fill . . . 164
4.19.4 Funnel plot . . . 165
4.19.5 Baujat . . . 166
4.19.6 Leave one out . . . 167
4.20 Clavien IV . . . 168
4.20.1 Meta-analysis . . . 168
4.20.2 Forest plot . . . 169
4.20.3 Trim and Fill . . . 170
4.20.4 Funnel plot . . . 171
4.20.5 Baujat . . . 172
4.20.6 Leave one out . . . 173
4.21 Clavien V . . . 174
4.21.1 Meta-analysis . . . 174
4.21.2 Forest plot . . . 175
4.21.3 Trim and Fill . . . 176
4.21.4 Funnel plot . . . 177
4.21.5 Baujat . . . 178
4.21.6 Leave one out . . . 179
4.22 Clavien I-II . . . 180
4.22.1 Meta-analysis . . . 180
4.22.2 Forest plot . . . 181
4.22.3 Trim and Fill . . . 182
4.22.4 Funnel plot . . . 183
4.22.5 Baujat . . . 184
4.22.6 Leave one out . . . 185
4.23 Clavien III-V . . . 186
4.23.1 Meta-analysis . . . 186
4.23.2 Forest plot . . . 187
4.23.3 Trim and Fill . . . 188
4.23.4 Funnel plot . . . 189
4.23.5 Baujat . . . 190
4.23.6 Leave one out . . . 191

5 Overall Summary Forest plots **192**
5.1 Summary Table of number of studies for each outcome included in meta-analysis . . . 196
5.2 Summary Forest plot of Continuous outcomes . . . 197
5.2.1 Continuous Outcomes Table . . . 197
5.2.2 Continuous Forest plot . . . 198
5.3 Summary Forest plot of Binary outcomes . . . 199
5.3.1 Binary Outcomes Table . . . 200
5.3.2 Binary Forest plot - Meta-Analysis . . . 201

6 RCT only Meta-Analysis Outcomes **202**
6.1 Immediate SFR . . . 203
6.1.1 Meta-analysis . . . 203
6.1.2 Forest plot . . . 204

6.1.3 Trim and Fill . . . 205
6.1.4 Funnel plot . . . 206
6.1.5 Baujat . . . 207
6.1.6 Leave one out . . . 208
6.2 Final SFR . . . 209
6.2.1 Meta-analysis . . . 209
6.2.2 Forest plot . . . 210
6.2.3 Trim and Fill . . . 211
6.2.4 Funnel plot . . . 212
6.2.5 Baujat . . . 213
6.2.6 Leave one out . . . 214
6.3 OR time . . . 215
6.3.1 Meta-analysis . . . 215
6.3.2 Forest plot . . . 216
6.3.3 Trim and Fill . . . 217
6.3.4 Funnel plot . . . 218
6.3.5 Baujat . . . 219
6.3.6 Leave one out . . . 220
6.4 Auxiliary Treatments . . . 221
6.4.1 Meta-analysis . . . 221
6.4.2 Forest plot . . . 222
6.4.3 Trim and Fill . . . 223
6.4.4 Funnel plot . . . 224
6.4.5 Baujat . . . 225
6.4.6 Leave one out . . . 226
6.5 VAS . . . 227
6.5.1 Meta-analysis . . . 227
6.5.2 Forest plot . . . 228
6.5.3 Trim and Fill . . . 229
6.5.4 Funnel plot . . . 230
6.5.5 Baujat . . . 231
6.5.6 Leave one out . . . 232
6.6 LoS . . . 233
6.6.1 Meta-analysis . . . 233
6.6.2 Forest plot . . . 234
6.6.3 Trim and Fill . . . 235
6.6.4 Funnel plot . . . 236
6.6.5 Baujat . . . 237
6.6.6 Leave one out . . . 238
6.7 Complications - All . . . 239
6.7.1 Meta-analysis . . . 239
6.7.2 Forest plot . . . 240
6.7.3 Trim and Fill . . . 241
6.7.4 Funnel plot . . . 242
6.7.5 Baujat . . . 243
6.7.6 Leave one out . . . 244
6.8 Fever . . . 245
6.8.1 Meta-analysis . . . 245
6.8.2 Forest plot . . . 246
6.8.3 Trim and Fill . . . 247
6.8.4 Funnel plot . . . 248
6.8.5 Baujat . . . 249
6.8.6 Leave one out . . . 250
6.9 Infections . . . 251

6.9.1 Meta-analysis . . . 251
6.9.2 Forest plot . . . 252
6.9.3 Trim and Fill . . . 253
6.9.4 Funnel plot . . . 254
6.9.5 Baujat . . . 255
6.9.6 Leave one out . . . 256
6.10 Sepsis . . . 257
6.10.1 Meta-analysis . . . 257
6.10.2 Forest plot . . . 258
6.10.3 Trim and Fill . . . 259
6.10.4 Funnel plot . . . 260
6.10.5 Baujat . . . 261
6.10.6 Leave one out . . . 262
6.11 Abscess . . . 263
6.11.1 Meta-analysis . . . 263
6.11.2 Forest plot . . . 264
6.11.3 Trim and Fill . . . 265
6.11.4 Funnel plot . . . 266
6.11.5 Baujat . . . 267
6.11.6 Leave one out . . . 268
6.12 Haematoma . . . 269
6.12.1 Meta-analysis . . . 269
6.12.2 Forest plot . . . 270
6.12.3 Trim and Fill . . . 271
6.12.4 Funnel plot . . . 272
6.12.5 Baujat . . . 273
6.12.6 Leave one out . . . 274
6.13 Pain . . . 275
6.13.1 Meta-analysis . . . 275
6.13.2 Forest plot . . . 276
6.13.3 Trim and Fill . . . 277
6.13.4 Funnel plot . . . 278
6.13.5 Baujat . . . 279
6.13.6 Leave one out . . . 280
6.14 Stricture . . . 281
6.14.1 Meta-analysis . . . 281
6.14.2 Forest plot . . . 282
6.14.3 Trim and Fill . . . 283
6.14.4 Funnel plot . . . 284
6.14.5 Baujat . . . 285
6.14.6 Leave one out . . . 286
6.15 Embolisation required . . . 287
6.15.1 Meta-analysis . . . 287
6.15.2 Forest plot . . . 288
6.15.3 Trim and Fill . . . 289
6.15.4 Funnel plot . . . 290
6.15.5 Baujat . . . 291
6.15.6 Leave one out . . . 292
6.16 Transfusion . . . 293
6.16.1 Meta-analysis . . . 293
6.16.2 Forest plot . . . 294
6.16.3 Trim and Fill . . . 295
6.16.4 Funnel plot . . . 296
6.16.5 Baujat . . . 297

6.16.6 Leave one out . . . 298
6.17 Clavien I . . . 299
6.17.1 Meta-analysis . . . 299
6.17.2 Forest plot . . . 300
6.17.3 Trim and Fill . . . 301
6.17.4 Funnel plot . . . 302
6.17.5 Baujat . . . 303
6.17.6 Leave one out . . . 304
6.18 Clavien II . . . 305
6.18.1 Meta-analysis . . . 305
6.18.2 Forest plot . . . 306
6.18.3 Trim and Fill . . . 307
6.18.4 Funnel plot . . . 308
6.18.5 Baujat . . . 309
6.18.6 Leave one out . . . 310
6.19 Clavien III . . . 311
6.19.1 Meta-analysis . . . 311
6.19.2 Forest plot . . . 312
6.19.3 Trim and Fill . . . 313
6.19.4 Funnel plot . . . 314
6.19.5 Baujat . . . 315
6.19.6 Leave one out . . . 316
6.20 Clavien IV . . . 317
6.20.1 Meta-analysis . . . 317
6.20.2 Forest plot . . . 318
6.20.3 Trim and Fill . . . 319
6.20.4 Funnel plot . . . 320
6.20.5 Baujat . . . 321
6.20.6 Leave one out . . . 322
6.21 Clavien V . . . 323
6.21.1 Meta-analysis . . . 323
6.21.2 Forest plot . . . 324
6.21.3 Trim and Fill . . . 325
6.21.4 Funnel plot . . . 326
6.21.5 Baujat . . . 327
6.21.6 Leave one out . . . 328
6.22 Clavien I-II . . . 329
6.22.1 Meta-analysis . . . 329
6.22.2 Forest plot . . . 330
6.22.3 Trim and Fill . . . 331
6.22.4 Funnel plot . . . 332
6.22.5 Baujat . . . 333
6.22.6 Leave one out . . . 334
6.23 Clavien III-V . . . 335
6.23.1 Meta-analysis . . . 335
6.23.2 Forest plot . . . 336
6.23.3 Trim and Fill . . . 337
6.23.4 Funnel plot . . . 338
6.23.5 Baujat . . . 339
6.23.6 Leave one out . . . 340

7 RCT Only Summary Forest plots **341**
7.1 Summary Table of number of studies for each outcome included in meta-analysis . . . 345
7.2 Summary Forest plot of Continuous outcomes . . . 346

7.2.1 Continuous Outcomes Table . . . 346
7.2.2 Continuous Forest plot . . . 347
7.3 Summary Forest plot of Binary outcomes . . . 348
7.3.1 Binary Outcomes Table . . . 349
7.3.2 Binary Forest plot - Meta-Analysis . . . 350

8 Semi-rigid Only Meta-Analysis Outcomes 351
8.1 Immediate SFR . . . 352
8.1.1 Meta-analysis . . . 352
8.1.2 Forest plot . . . 353
8.1.3 Trim and Fill . . . 354
8.1.4 Funnel plot . . . 355
8.1.5 Baujat . . . 356
8.1.6 Leave one out . . . 357
8.2 Final SFR . . . 358
8.2.1 Meta-analysis . . . 358
8.2.2 Forest plot . . . 359
8.2.3 Trim and Fill . . . 360
8.2.4 Funnel plot . . . 361
8.2.5 Baujat . . . 362
8.2.6 Leave one out . . . 363
8.3 OR time . . . 364
8.3.1 Meta-analysis . . . 364
8.3.2 Forest plot . . . 365
8.3.3 Trim and Fill . . . 366
8.3.4 Funnel plot . . . 367
8.3.5 Baujat . . . 368
8.3.6 Leave one out . . . 369
8.4 Auxiliary Treatments . . . 370
8.4.1 Meta-analysis . . . 370
8.4.2 Forest plot . . . 371
8.4.3 Trim and Fill . . . 372
8.4.4 Funnel plot . . . 373
8.4.5 Baujat . . . 374
8.4.6 Leave one out . . . 375
8.5 VAS . . . 376
8.5.1 Meta-analysis . . . 376
8.5.2 Forest plot . . . 377
8.5.3 Trim and Fill . . . 378
8.5.4 Funnel plot . . . 379
8.5.5 Baujat . . . 380
8.5.6 Leave one out . . . 381
8.6 LoS . . . 382
8.6.1 Meta-analysis . . . 382
8.6.2 Forest plot . . . 383
8.6.3 Trim and Fill . . . 384
8.6.4 Funnel plot . . . 385
8.6.5 Baujat . . . 386
8.6.6 Leave one out . . . 387
8.7 Complications - All . . . 388
8.7.1 Meta-analysis . . . 388
8.7.2 Forest plot . . . 389
8.7.3 Trim and Fill . . . 390
8.7.4 Funnel plot . . . 391

8.7.5 Baujat 392
8.7.6 Leave one out 393
8.8 Fever 394
8.8.1 Meta-analysis 394
8.8.2 Forest plot 395
8.8.3 Trim and Fill 396
8.8.4 Funnel plot 397
8.8.5 Baujat 398
8.8.6 Leave one out 399
8.9 Infections 400
8.9.1 Meta-analysis 400
8.9.2 Forest plot 401
8.9.3 Trim and Fill 402
8.9.4 Funnel plot 403
8.9.5 Baujat 404
8.9.6 Leave one out 405
8.10 Sepsis 406
8.10.1 Meta-analysis 406
8.10.2 Forest plot 407
8.10.3 Trim and Fill 408
8.10.4 Funnel plot 409
8.10.5 Baujat 410
8.10.6 Leave one out 411
8.11 Abscess 412
8.11.1 Meta-analysis 412
8.11.2 Forest plot 413
8.11.3 Trim and Fill 414
8.11.4 Funnel plot 415
8.11.5 Baujat 416
8.11.6 Leave one out 417
8.12 Haematoma 418
8.12.1 Meta-analysis 418
8.12.2 Forest plot 419
8.12.3 Trim and Fill 420
8.12.4 Funnel plot 421
8.12.5 Baujat 422
8.12.6 Leave one out 423
8.13 Pain 424
8.13.1 Meta-analysis 424
8.13.2 Forest plot 425
8.13.3 Trim and Fill 426
8.13.4 Funnel plot 427
8.13.5 Baujat 428
8.13.6 Leave one out 429
8.14 Stricture 430
8.14.1 Meta-analysis 430
8.14.2 Forest plot 431
8.14.3 Trim and Fill 432
8.14.4 Funnel plot 433
8.14.5 Baujat 434
8.14.6 Leave one out 435
8.15 Embolisation required 436
8.15.1 Meta-analysis 436
8.15.2 Forest plot 437

8.15.3 Trim and Fill . . . 438
8.15.4 Funnel plot . . . 439
8.15.5 Baujat . . . 440
8.15.6 Leave one out . . . 441
8.16 Transfusion . . . 442
8.16.1 Meta-analysis . . . 442
8.16.2 Forest plot . . . 443
8.16.3 Trim and Fill . . . 444
8.16.4 Funnel plot . . . 445
8.16.5 Baujat . . . 446
8.16.6 Leave one out . . . 447
8.17 Clavien I . . . 448
8.17.1 Meta-analysis . . . 448
8.17.2 Forest plot . . . 449
8.17.3 Trim and Fill . . . 450
8.17.4 Funnel plot . . . 451
8.17.5 Baujat . . . 452
8.17.6 Leave one out . . . 453
8.18 Clavien II . . . 454
8.18.1 Meta-analysis . . . 454
8.18.2 Forest plot . . . 455
8.18.3 Trim and Fill . . . 456
8.18.4 Funnel plot . . . 457
8.18.5 Baujat . . . 458
8.18.6 Leave one out . . . 459
8.19 Clavien III . . . 460
8.19.1 Meta-analysis . . . 460
8.19.2 Forest plot . . . 461
8.19.3 Trim and Fill . . . 462
8.19.4 Funnel plot . . . 463
8.19.5 Baujat . . . 464
8.19.6 Leave one out . . . 465
8.20 Clavien IV . . . 466
8.20.1 Meta-analysis . . . 466
8.20.2 Forest plot . . . 467
8.20.3 Trim and Fill . . . 468
8.20.4 Funnel plot . . . 469
8.20.5 Baujat . . . 470
8.20.6 Leave one out . . . 471
8.21 Clavien V . . . 472
8.21.1 Meta-analysis . . . 472
8.21.2 Forest plot . . . 473
8.21.3 Trim and Fill . . . 474
8.21.4 Funnel plot . . . 475
8.21.5 Baujat . . . 476
8.21.6 Leave one out . . . 477
8.22 Clavien I-II . . . 478
8.22.1 Meta-analysis . . . 478
8.22.2 Forest plot . . . 479
8.22.3 Trim and Fill . . . 480
8.22.4 Funnel plot . . . 481
8.22.5 Baujat . . . 482
8.22.6 Leave one out . . . 483
8.23 Clavien III-V . . . 484

8.23.1 Meta-analysis . . . 484
8.23.2 Forest plot . . . 485
8.23.3 Trim and Fill . . . 486
8.23.4 Funnel plot . . . 487
8.23.5 Baujat . . . 488
8.23.6 Leave one out . . . 489

9 Semi-Rigid URS Only Summary Forest plots 490
9.1 Summary Table of number of studies for each outcome included in meta-analysis . . . 494
9.2 Summary Forest plot of Continuous outcomes . . . 495
9.2.1 Continuous Outcomes Table . . . 495
9.2.2 Continuous Forest plot . . . 496
9.3 Summary Forest plot of Binary outcomes . . . 497
9.3.1 Binary Outcomes Table . . . 498
9.3.2 Binary Forest plot - Meta-Analysis . . . 499

10 Flexi URS Only Meta-Analysis Outcomes 500
10.1 Immediate SFR . . . 501
10.1.1 Meta-analysis . . . 501
10.1.2 Forest plot . . . 502
10.1.3 Trim and Fill . . . 503
10.1.4 Funnel plot . . . 504
10.1.5 Baujat . . . 505
10.1.6 Leave one out . . . 506
10.2 Final SFR . . . 507
10.2.1 Meta-analysis . . . 507
10.2.2 Forest plot . . . 508
10.2.3 Trim and Fill . . . 509
10.2.4 Funnel plot . . . 510
10.2.5 Baujat . . . 511
10.2.6 Leave one out . . . 512
10.3 OR time . . . 513
10.3.1 Meta-analysis . . . 513
10.3.2 Forest plot . . . 514
10.3.3 Trim and Fill . . . 515
10.3.4 Funnel plot . . . 516
10.3.5 Baujat . . . 517
10.3.6 Leave one out . . . 518
10.4 Auxiliary Treatments . . . 519
10.4.1 Meta-analysis . . . 519
10.4.2 Forest plot . . . 520
10.4.3 Trim and Fill . . . 521
10.4.4 Funnel plot . . . 522
10.4.5 Baujat . . . 523
10.4.6 Leave one out . . . 524
10.5 VAS . . . 525
10.5.1 Meta-analysis . . . 525
10.5.2 Forest plot . . . 526
10.5.3 Trim and Fill . . . 527
10.5.4 Funnel plot . . . 528
10.5.5 Baujat . . . 529
10.5.6 Leave one out . . . 530
10.6 LoS . . . 531
10.6.1 Meta-analysis . . . 531

10.6.2 Forest plot 532
10.6.3 Trim and Fill 533
10.6.4 Funnel plot 534
10.6.5 Baujat 535
10.6.6 Leave one out 536
10.7 Complications - All 537
10.7.1 Meta-analysis 537
10.7.2 Forest plot 538
10.7.3 Trim and Fill 539
10.7.4 Funnel plot 540
10.7.5 Baujat 541
10.7.6 Leave one out 542
10.8 Fever 543
10.8.1 Meta-analysis 543
10.8.2 Forest plot 544
10.8.3 Trim and Fill 545
10.8.4 Funnel plot 546
10.8.5 Baujat 547
10.8.6 Leave one out 548
10.9 Infections 549
10.9.1 Meta-analysis 549
10.9.2 Forest plot 550
10.9.3 Trim and Fill 551
10.9.4 Funnel plot 552
10.9.5 Baujat 553
10.9.6 Leave one out 554
10.10 Sepsis 555
10.10.1 Meta-analysis 555
10.10.2 Forest plot 556
10.10.3 Trim and Fill 557
10.10.4 Funnel plot 558
10.10.5 Baujat 559
10.10.6 Leave one out 560
10.11 Abscess 561
10.11.1 Meta-analysis 561
10.11.2 Forest plot 562
10.11.3 Trim and Fill 563
10.11.4 Funnel plot 564
10.11.5 Baujat 565
10.11.6 Leave one out 566
10.12 Haematoma 567
10.12.1 Meta-analysis 567
10.12.2 Forest plot 568
10.12.3 Trim and Fill 569
10.12.4 Funnel plot 570
10.12.5 Baujat 571
10.12.6 Leave one out 572
10.13 Pain 573
10.13.1 Meta-analysis 573
10.13.2 Forest plot 574
10.13.3 Trim and Fill 575
10.13.4 Funnel plot 576
10.13.5 Baujat 577
10.13.6 Leave one out 578

10.14 Stricture . . . 579
10.14.1 Meta-analysis . . . 579
10.14.2 Forest plot . . . 580
10.14.3 Trim and Fill . . . 581
10.14.4 Funnel plot . . . 582
10.14.5 Baujat . . . 583
10.14.6 Leave one out . . . 584
10.15 Embolisation required . . . 585
10.15.1 Meta-analysis . . . 585
10.15.2 Forest plot . . . 586
10.15.3 Trim and Fill . . . 587
10.15.4 Funnel plot . . . 588
10.15.5 Baujat . . . 589
10.15.6 Leave one out . . . 590
10.16 Transfusion . . . 591
10.16.1 Meta-analysis . . . 591
10.16.2 Forest plot . . . 592
10.16.3 Trim and Fill . . . 593
10.16.4 Funnel plot . . . 594
10.16.5 Baujat . . . 595
10.16.6 Leave one out . . . 596
10.17 Clavien I . . . 597
10.17.1 Meta-analysis . . . 597
10.17.2 Forest plot . . . 598
10.17.3 Trim and Fill . . . 599
10.17.4 Funnel plot . . . 600
10.17.5 Baujat . . . 601
10.17.6 Leave one out . . . 602
10.18 Clavien II . . . 603
10.18.1 Meta-analysis . . . 603
10.18.2 Forest plot . . . 604
10.18.3 Trim and Fill . . . 605
10.18.4 Funnel plot . . . 606
10.18.5 Baujat . . . 607
10.18.6 Leave one out . . . 608
10.19 Clavien III . . . 609
10.19.1 Meta-analysis . . . 609
10.19.2 Forest plot . . . 610
10.19.3 Trim and Fill . . . 611
10.19.4 Funnel plot . . . 612
10.19.5 Baujat . . . 613
10.19.6 Leave one out . . . 614
10.20 Clavien IV . . . 615
10.20.1 Meta-analysis . . . 615
10.20.2 Forest plot . . . 616
10.20.3 Trim and Fill . . . 617
10.20.4 Funnel plot . . . 618
10.20.5 Baujat . . . 619
10.20.6 Leave one out . . . 620
10.21 Clavien V . . . 621
10.21.1 Meta-analysis . . . 621
10.21.2 Forest plot . . . 622
10.21.3 Trim and Fill . . . 623
10.21.4 Funnel plot . . . 624

10.21.5 Baujat . . . 625
10.21.6 Leave one out . . . 626
10.22 Clavien I-II . . . 627
10.22.1 Meta-analysis . . . 627
10.22.2 Forest plot . . . 628
10.22.3 Trim and Fill . . . 629
10.22.4 Funnel plot . . . 630
10.22.5 Baujat . . . 631
10.22.6 Leave one out . . . 632
10.23 Clavien III-V . . . 633
10.23.1 Meta-analysis . . . 633
10.23.2 Forest plot . . . 634
10.23.3 Trim and Fill . . . 635
10.23.4 Funnel plot . . . 636
10.23.5 Baujat . . . 637
10.23.6 Leave one out . . . 638

11 Flexi URS only Summary Forest plots 639
11.1 Summary Table of number of studies for each outcome included in meta-analysis . . . 643
11.2 Summary Forest plot of Continuous outcomes . . . 644
11.2.1 Continuous Outcomes Table . . . 644
11.2.2 Continuous Forest plot . . . 645
11.3 Summary Forest plot of Binary outcomes . . . 646
11.3.1 Binary Outcomes Table . . . 647
11.3.2 Binary Forest plot - Meta-Analysis . . . 648

12 Mini-PCNL vs Flexi URS with Suction Meta-Analysis Outcomes 649
12.1 Immediate SFR . . . 650
12.1.1 Meta-analysis . . . 650
12.1.2 Forest plot . . . 651
12.1.3 Trim and Fill . . . 652
12.1.4 Funnel plot . . . 653
12.1.5 Baujat . . . 654
12.1.6 Leave one out . . . 655
12.2 Final SFR . . . 656
12.2.1 Meta-analysis . . . 656
12.2.2 Forest plot . . . 657
12.2.3 Trim and Fill . . . 658
12.2.4 Funnel plot . . . 659
12.2.5 Baujat . . . 660
12.2.6 Leave one out . . . 661
12.3 OR time . . . 662
12.3.1 Meta-analysis . . . 662
12.3.2 Forest plot . . . 663
12.3.3 Trim and Fill . . . 664
12.3.4 Funnel plot . . . 665
12.3.5 Baujat . . . 666
12.3.6 Leave one out . . . 667
12.4 Auxiliary Treatments . . . 668
12.4.1 Meta-analysis . . . 668
12.4.2 Forest plot . . . 669
12.4.3 Trim and Fill . . . 670
12.4.4 Funnel plot . . . 671
12.4.5 Baujat . . . 672

12.4.6 Leave one out 673
12.5 VAS 674
12.5.1 Meta-analysis 674
12.5.2 Forest plot 675
12.5.3 Trim and Fill 676
12.5.4 Funnel plot 677
12.5.5 Baujat 678
12.5.6 Leave one out 679
12.6 LoS 680
12.6.1 Meta-analysis 680
12.6.2 Forest plot 681
12.6.3 Trim and Fill 682
12.6.4 Funnel plot 683
12.6.5 Baujat 684
12.6.6 Leave one out 685
12.7 Complications - All 686
12.7.1 Meta-analysis 686
12.7.2 Forest plot 687
12.7.3 Trim and Fill 688
12.7.4 Funnel plot 689
12.7.5 Baujat 690
12.7.6 Leave one out 691
12.8 Fever 692
12.8.1 Meta-analysis 692
12.8.2 Forest plot 693
12.8.3 Trim and Fill 694
12.8.4 Funnel plot 695
12.8.5 Baujat 696
12.8.6 Leave one out 697
12.9 Infections 698
12.9.1 Meta-analysis 698
12.9.2 Forest plot 699
12.9.3 Trim and Fill 700
12.9.4 Funnel plot 701
12.9.5 Baujat 702
12.9.6 Leave one out 703
12.10 Sepsis 704
12.10.1 Meta-analysis 704
12.10.2 Forest plot 705
12.10.3 Trim and Fill 706
12.10.4 Funnel plot 707
12.10.5 Baujat 708
12.10.6 Leave one out 709
12.11 Abscess 710
12.11.1 Meta-analysis 710
12.11.2 Forest plot 711
12.11.3 Trim and Fill 712
12.11.4 Funnel plot 713
12.11.5 Baujat 714
12.11.6 Leave one out 715
12.12 Haematoma 716
12.12.1 Meta-analysis 716
12.12.2 Forest plot 717
12.12.3 Trim and Fill 718

12.12.4 Funnel plot 719
12.12.5 Baujat 720
12.12.6 Leave one out 721
12.13 Pain 722
12.13.1 Meta-analysis 722
12.13.2 Forest plot 723
12.13.3 Trim and Fill 724
12.13.4 Funnel plot 725
12.13.5 Baujat 726
12.13.6 Leave one out 727
12.14 Stricture 728
12.14.1 Meta-analysis 728
12.14.2 Forest plot 729
12.14.3 Trim and Fill 730
12.14.4 Funnel plot 731
12.14.5 Baujat 732
12.14.6 Leave one out 733
12.15 Embolisation required 734
12.15.1 Meta-analysis 734
12.15.2 Forest plot 735
12.15.3 Trim and Fill 736
12.15.4 Funnel plot 737
12.15.5 Baujat 738
12.15.6 Leave one out 739
12.16 Transfusion 740
12.16.1 Meta-analysis 740
12.16.2 Forest plot 741
12.16.3 Trim and Fill 742
12.16.4 Funnel plot 743
12.16.5 Baujat 744
12.16.6 Leave one out 745
12.17 Clavien I 746
12.17.1 Meta-analysis 746
12.17.2 Forest plot 747
12.17.3 Trim and Fill 748
12.17.4 Funnel plot 749
12.17.5 Baujat 750
12.17.6 Leave one out 751
12.18 Clavien II 752
12.18.1 Meta-analysis 752
12.18.2 Forest plot 753
12.18.3 Trim and Fill 754
12.18.4 Funnel plot 755
12.18.5 Baujat 756
12.18.6 Leave one out 757
12.19 Clavien III 758
12.19.1 Meta-analysis 758
12.19.2 Forest plot 759
12.19.3 Trim and Fill 760
12.19.4 Funnel plot 761
12.19.5 Baujat 762
12.19.6 Leave one out 763
12.20 Clavien IV 764
12.20.1 Meta-analysis 764

12.20.2 Forest plot 765
12.20.3 Trim and Fill 766
12.20.4 Funnel plot 767
12.20.5 Baujat 768
12.20.6 Leave one out 769
12.21 Clavien V 770
12.21.1 Meta-analysis 770
12.21.2 Forest plot 771
12.21.3 Trim and Fill 772
12.21.4 Funnel plot 773
12.21.5 Baujat 774
12.21.6 Leave one out 775
12.22 Clavien I-II 776
12.22.1 Meta-analysis 776
12.22.2 Forest plot 777
12.22.3 Trim and Fill 778
12.22.4 Funnel plot 779
12.22.5 Baujat 780
12.22.6 Leave one out 781
12.23 Clavien III-V 782
12.23.1 Meta-analysis 782
12.23.2 Forest plot 783
12.23.3 Trim and Fill 784
12.23.4 Funnel plot 785
12.23.5 Baujat 786
12.23.6 Leave one out 787

13 Mini-PCNL vs Flexi URS Summary Forest plots **788**
13.1 Summary Table of number of studies for each outcome included in meta-analysis 792
13.2 Summary Forest plot of Continuous outcomes 793
13.2.1 Continuous Outcomes Table 793
13.2.2 Continuous Forest plot 794
13.3 Summary Forest plot of Binary outcomes 795
13.3.1 Binary Outcomes Table 796
13.3.2 Binary Forest plot - Meta-Analysis 797

1 Setup

```
knitr::opts_chunk$set(echo = TRUE)
library(tidyverse)
```

```
## -- Attaching core tidyverse packages ------------------------ tidyverse 2.0.0 --
## v dplyr     1.1.4     v readr     2.1.4
## v forcats   1.0.0     v stringr   1.5.1
## v ggplot2   3.4.4     v tibble    3.2.1
## v lubridate 1.9.2     v tidyr     1.3.0
## v purrr     1.0.2
## -- Conflicts ------------------------------------------ tidyverse_conflicts() --
## x dplyr::filter() masks stats::filter()
## x dplyr::lag()    masks stats::lag()
## i Use the conflicted package (<http://conflicted.r-lib.org/>) to force all conflicts to become error
```

```
library(gt)
library(gtExtras)
```

```r
library(meta)
```

```
## Loading 'meta' package (version 6.5-0).
## Type 'help(meta)' for a brief overview.
## Readers of 'Meta-Analysis with R (Use R!)' should install
## older version of 'meta' package: https://tinyurl.com/dt4y5drs
```

```r
library(metafor)
```

```
## Loading required package: Matrix
##
## Attaching package: 'Matrix'
##
## The following objects are masked from 'package:tidyr':
##
##     expand, pack, unpack
##
## Loading required package: metadat
## Loading required package: numDeriv
##
## Loading the 'metafor' package (version 4.2-0). For an
## introduction to the package please type: help(metafor)
```

```r
library(janitor)
```

```
##
## Attaching package: 'janitor'
##
## The following objects are masked from 'package:stats':
##
##     chisq.test, fisher.test
```

```r
library(DataExplorer)
library(forestplot)
```

```
## Loading required package: grid
## Loading required package: checkmate
## Loading required package: abind
```

```r
suction_data <- read.csv("~/Desktop/Somani/Suction/suction_urs.csv") %>% as_tibble() %>% janitor::clean_
```

1.1 Data Encoding

```
#Define Demographics
suction_data$design <- as.factor(suction_data$design)
suction_data$number_of_surgeons_control <- as.integer(suction_data$number_of_surgeons_control)
```

```
## Warning: NAs introduced by coercion
```

```
suction_data$number_of_surgeons_suction<- as.integer(suction_data$number_of_surgeons_suction)
```

```
## Warning: NAs introduced by coercion
```

```
suction_data$age_mean_control <- as.numeric(suction_data$age_mean_control)
```

```
## Warning: NAs introduced by coercion
```

```
suction_data$age_sd_control <- as.numeric(suction_data$age_sd_control)
```

```
## Warning: NAs introduced by coercion
```

```
suction_data$age_mean_suction <- as.numeric(suction_data$age_mean_suction)
```

```
## Warning: NAs introduced by coercion
```

```
suction_data$age_sd_suction <- as.numeric(suction_data$age_sd_suction)
```

```
## Warning: NAs introduced by coercion
```

```
suction_data$male_n_control <- as.integer(suction_data$male_n_control)
```

```
## Warning: NAs introduced by coercion
```

```
suction_data$male_n_suction <- as.integer(suction_data$male_n_suction)
```

```
## Warning: NAs introduced by coercion
```

```
suction_data$bmi_mean_control <- as.numeric(suction_data$bmi_mean_control)
```

```
## Warning: NAs introduced by coercion
```

```
suction_data$bmi_sd_control <- as.numeric(suction_data$bmi_sd_control)
```

```
## Warning: NAs introduced by coercion
```

```
suction_data$bmi_mean_suction <- as.numeric(suction_data$bmi_mean_suction)
```

```
## Warning: NAs introduced by coercion
```

```
suction_data$bmi_sd_suction <- as.numeric(suction_data$bmi_sd_suction)
```

```
## Warning: NAs introduced by coercion
```

```
suction_data$hu_mean_control <- as.numeric(suction_data$hu_mean_control)
```

```
## Warning: NAs introduced by coercion
```

```
suction_data$hu_sd_control <- as.numeric(suction_data$hu_sd_control)
```

```
## Warning: NAs introduced by coercion
```

```
suction_data$hu_mean_suction <- as.numeric(suction_data$hu_mean_suction)
```

```
## Warning: NAs introduced by coercion
```

```r
suction_data$hu_sd_suction <- as.numeric(suction_data$hu_sd_suction)
```

```
## Warning: NAs introduced by coercion
```

```r
suction_data$stone_size_mm_mean_control <- as.numeric(suction_data$stone_size_mm_mean_control)
```

```
## Warning: NAs introduced by coercion
```

```r
suction_data$stone_size_mm_sd_control <- as.numeric(suction_data$stone_size_mm_sd_control)
```

```
## Warning: NAs introduced by coercion
```

```r
suction_data$stone_size_mm_mean_suction <- as.numeric(suction_data$stone_size_mm_mean_suction)
```

```
## Warning: NAs introduced by coercion
```

```r
suction_data$stone_size_mm_sd_suction <- as.numeric(suction_data$stone_size_mm_sd_suction)
```

```
## Warning: NAs introduced by coercion
```

```r
suction_data$stone_surface_mm2_mean_control <- as.numeric(suction_data$stone_surface_mm2_mean_control)
```

```
## Warning: NAs introduced by coercion
```

```r
suction_data$stone_surface_mm2_sd_control <- as.numeric(suction_data$stone_surface_mm2_sd_control)
```

```
## Warning: NAs introduced by coercion
```

```r
suction_data$stone_surface_mm2_mean_suction <- as.numeric(suction_data$stone_surface_mm2_mean_suction)
```

```
## Warning: NAs introduced by coercion
```

```r
suction_data$stone_surface_mm2_sd_suction <- as.numeric(suction_data$stone_surface_mm2_sd_suction)
```

```
## Warning: NAs introduced by coercion
```

```r
suction_data$stone_burden_mm3_mean_control <- as.numeric(suction_data$stone_burden_mm3_mean_control)
```

```
## Warning: NAs introduced by coercion
```

```r
suction_data$stone_burden_mm3_sd_control <- as.numeric(suction_data$stone_burden_mm3_sd_control)
```

```
## Warning: NAs introduced by coercion
```

```r
suction_data$stone_burden_mm3_mean_suction <- as.numeric(suction_data$stone_burden_mm3_mean_suction)
```

```
## Warning: NAs introduced by coercion
```

```r
suction_data$stone_burden_mm3_sd_suction <- as.numeric(suction_data$stone_burden_mm3_sd_suction)
```

```
## Warning: NAs introduced by coercion
```

```r
suction_data$upper_ureter_n_control <- as.integer(suction_data$upper_ureter_n_control)
```

```
## Warning: NAs introduced by coercion
```

```r
suction_data$upper_ureter_n_suction <- as.integer(suction_data$upper_ureter_n_suction)
```

```
## Warning: NAs introduced by coercion
```

```r
suction_data$mid_ureter_n_control <- as.integer(suction_data$mid_ureter_n_control)
```

```
## Warning: NAs introduced by coercion
```

```r
suction_data$mid_ureter_n_suction <- as.integer(suction_data$mid_ureter_n_suction)
```

```
## Warning: NAs introduced by coercion
```

```r
suction_data$distal_ureter_n_control <- as.integer(suction_data$distal_ureter_n_control)
```

```
## Warning: NAs introduced by coercion
```

```r
suction_data$distal_ureter_n_suction <- as.integer(suction_data$distal_ureter_n_suction)
```

```
## Warning: NAs introduced by coercion
```

```r
suction_data$renal_pelvis_n_control <- as.integer(suction_data$renal_pelvis_n_control)
```

```
## Warning: NAs introduced by coercion
```

```r
suction_data$renal_pelvis_n_suction <- as.integer(suction_data$renal_pelvis_n_suction)
```

```
## Warning: NAs introduced by coercion
```

```r
suction_data$upper_calyx_n_control <- as.integer(suction_data$upper_calyx_n_control)
```

```
## Warning: NAs introduced by coercion
```

```r
suction_data$upper_calyx_n_suction <- as.integer(suction_data$upper_calyx_n_suction)
```

```
## Warning: NAs introduced by coercion
```

```r
suction_data$middle_calyx_n_control <- as.integer(suction_data$middle_calyx_n_control)
```

```
## Warning: NAs introduced by coercion
```

```r
suction_data$middle_calyx_n_suction <- as.integer(suction_data$middle_calyx_n_suction)
```

```
## Warning: NAs introduced by coercion
```

```r
suction_data$lower_calyx_n_control <- as.integer(suction_data$lower_calyx_n_control)
```

```
## Warning: NAs introduced by coercion
```

```r
suction_data$lower_calxy_n_suction <- as.integer(suction_data$lower_calxy_n_suction)
```

```
## Warning: NAs introduced by coercion
```

```r
# Define Outcomes
suction_data$sfr_immediate_n_control <- as.integer(suction_data$sfr_immediate_n_control)
```

```
## Warning: NAs introduced by coercion
```

```r
suction_data$sfr_immediate_n_suction <- as.integer(suction_data$sfr_immediate_n_suction)
```

```
## Warning: NAs introduced by coercion
```

```r
suction_data$sfr_final_n_control <- as.integer(suction_data$sfr_final_n_control)
```

```
## Warning: NAs introduced by coercion
```

```r
suction_data$sfr_final_n_suction <- as.integer(suction_data$sfr_final_n_suction)
```

```
## Warning: NAs introduced by coercion
```

```r
suction_data$auxillary_tx_n_control <- as.integer(suction_data$auxillary_tx_n_control)
```

```
## Warning: NAs introduced by coercion
```

```r
suction_data$auxillary_tx_n_suction <- as.integer(suction_data$auxillary_tx_n_suction)
```

```
## Warning: NAs introduced by coercion
```

```r
suction_data$or_time_min_mean_control <- as.numeric(suction_data$or_time_min_mean_control)
```

```
## Warning: NAs introduced by coercion
```

```r
suction_data$or_time_min_sd_control <- as.numeric(suction_data$or_time_min_sd_control)
```

```
## Warning: NAs introduced by coercion
```

```r
suction_data$or_time_min_mean_suction <- as.numeric(suction_data$or_time_min_mean_suction)
```

```
## Warning: NAs introduced by coercion
```

```r
suction_data$or_time_min_sd_suction <- as.numeric(suction_data$or_time_min_sd_suction)
```

```
## Warning: NAs introduced by coercion
```

```r
suction_data$fragmentation_time_min_mean_control <- as.numeric(suction_data$fragmentation_time_min_mean_
```

```
## Warning: NAs introduced by coercion
```

```r
suction_data$fragmentation_time_min_sd_control <- as.numeric(suction_data$fragmentation_time_min_sd_con
```

```
## Warning: NAs introduced by coercion
```

```r
suction_data$fragmentation_time_min_mean_suction <- as.numeric(suction_data$fragmentation_time_min_mean_
```

```
## Warning: NAs introduced by coercion
```

```r
suction_data$fragmentation_time_min_sd_suction <- as.numeric(suction_data$fragmentation_time_min_sd_suc
```

```
## Warning: NAs introduced by coercion
```

```r
suction_data$fluoroscopy_time_sec_mean_control <- as.numeric(suction_data$fluoroscopy_time_sec_mean_con
```

```
## Warning: NAs introduced by coercion
```

```r
suction_data$fluoroscopy_time_sec_sd_control <- as.numeric(suction_data$fluoroscopy_time_sec_sd_control
```

```
## Warning: NAs introduced by coercion
```

```r
suction_data$fluoroscopy_time_sec_mean_suction <- as.numeric(suction_data$fluoroscopy_time_sec_mean_suc
```

```
## Warning: NAs introduced by coercion
```

```r
suction_data$fluoroscopy_time_sec_sd_suction <- as.numeric(suction_data$fluoroscopy_time_sec_sd_suction
```

```
## Warning: NAs introduced by coercion
```

```r
suction_data$use_of_baskets_n_control <- as.integer(suction_data$use_of_baskets_n_control)
```

```
## Warning: NAs introduced by coercion
```

```r
suction_data$use_of_baskets_n_suction <- as.integer(suction_data$use_of_baskets_n_suction)
```

```
## Warning: NAs introduced by coercion
```

```r
suction_data$intrapelvic_pressure_mm_hg_mean_control <- as.numeric(suction_data$intrapelvic_pressure_mm_
```

```
## Warning: NAs introduced by coercion
```

```r
suction_data$intrapelvic_pressure_mm_hg_mean_suction <- as.numeric(suction_data$intrapelvic_pressure_mm_
```

```
## Warning: NAs introduced by coercion
```

```
suction_data$los_days_mean_control <- as.numeric(suction_data$los_days_mean_control)
```

```
## Warning: NAs introduced by coercion
```

```
suction_data$los_days_sd_control <- as.numeric(suction_data$los_days_sd_control)
```

```
## Warning: NAs introduced by coercion
```

```
suction_data$los_days_mean_suction <- as.numeric(suction_data$los_days_mean_suction)
```

```
## Warning: NAs introduced by coercion
```

```
suction_data$los_days_sd_suction <- as.numeric(suction_data$los_days_sd_suction)
```

```
## Warning: NAs introduced by coercion
```

```
suction_data$complications_postop_overall_n_control <- as.integer(suction_data$complications_postop_over
```

```
## Warning: NAs introduced by coercion
```

```
suction_data$complications_postop_overall_n_suction <- as.integer(suction_data$complications_postop_over
```

```
## Warning: NAs introduced by coercion
```

```
suction_data$infection_n_control <- as.integer(suction_data$infection_n_control)
```

```
## Warning: NAs introduced by coercion
```

```
suction_data$infection_n_suction <- as.integer(suction_data$infection_n_suction)
```

```
## Warning: NAs introduced by coercion
```

```
suction_data$fever_n_control <- as.integer(suction_data$fever_n_control)
```

```
## Warning: NAs introduced by coercion
```

```
suction_data$fever_n_suction <- as.integer(suction_data$fever_n_suction)
```

```
## Warning: NAs introduced by coercion
```

```
suction_data$abscess_n_control <- as.integer(suction_data$abscess_n_control)
```

```
## Warning: NAs introduced by coercion
```

```
suction_data$abscess_n_suction <- as.integer(suction_data$abscess_n_suction)
```

```
## Warning: NAs introduced by coercion
```

```
suction_data$sepsis_n_control <- as.integer(suction_data$sepsis_n_control)
```

```
## Warning: NAs introduced by coercion
```

```
suction_data$sepsis_n_suction <- as.integer(suction_data$sepsis_n_suction)
```

```
## Warning: NAs introduced by coercion
```

```
suction_data$pain_n_control <- as.integer(suction_data$pain_n_control)
```

```
## Warning: NAs introduced by coercion
```

```
suction_data$pain_n_suction <- as.integer(suction_data$pain_n_suction)
```

```
## Warning: NAs introduced by coercion
```

```
suction_data$hematoma_n_control <- as.integer(suction_data$hematoma_n_control)
```

```
## Warning: NAs introduced by coercion
```

```
suction_data$hematoma_n_suction <- as.integer(suction_data$hematoma_n_suction)
```

```
## Warning: NAs introduced by coercion
```

```
suction_data$transfusion_n_control <- as.integer(suction_data$transfusion_n_control)
```

```
## Warning: NAs introduced by coercion
```

```
suction_data$transfusion_n_suction <- as.integer(suction_data$transfusion_n_suction)
```

```
## Warning: NAs introduced by coercion
```

```
suction_data$embolism_ir_intervention_n_control <- as.integer(suction_data$embolism_ir_intervention_n_c
```

```
## Warning: NAs introduced by coercion
```

```
suction_data$embolism_ir_intervention_n_suction <- as.integer(suction_data$embolism_ir_intervention_n_su
```

```
## Warning: NAs introduced by coercion
```

```
suction_data$cost_us_dollars_mean_control <- as.numeric(suction_data$cost_us_dollars_mean_control)
```

```
## Warning: NAs introduced by coercion
```

```
suction_data$cost_us_dollars_sd_control <- as.numeric(suction_data$cost_us_dollars_sd_control)
```

```
## Warning: NAs introduced by coercion
```

```
suction_data$cost_us_dollars_mean_suction <- as.numeric(suction_data$cost_us_dollars_mean_suction)
```

```
## Warning: NAs introduced by coercion
```

```
suction_data$cost_us_dollars_sd_suction <- as.numeric(suction_data$cost_us_dollars_sd_suction)
```

```
## Warning: NAs introduced by coercion
```

```
suction_data$clavien_i_n_control <- as.integer(suction_data$clavien_i_n_control)
```

```
## Warning: NAs introduced by coercion
```

```
suction_data$clavien_ii_n_control <- as.integer(suction_data$clavien_ii_n_control)
```

```
## Warning: NAs introduced by coercion
```

```
suction_data$clavien_iii_n_control <- as.integer(suction_data$clavien_iii_n_control)
```

```
## Warning: NAs introduced by coercion
```

```
suction_data$clavien_iv_n_control <- as.integer(suction_data$clavien_iv_n_control)
```

```
## Warning: NAs introduced by coercion
```

```
suction_data$clavien_v_n_control <- as.integer(suction_data$clavien_v_n_control)
suction_data$clavien_i_n_suction <- as.integer(suction_data$clavien_i_n_suction)
```

```
## Warning: NAs introduced by coercion
```

```
suction_data$clavien_ii_n_suction <- as.integer(suction_data$clavien_ii_n_suction)
```

```
## Warning: NAs introduced by coercion
```

```r
suction_data$clavien_iii_n_suction <- as.integer(suction_data$clavien_iii_n_suction)
```

```
## Warning: NAs introduced by coercion
```

```r
suction_data$clavien_iv_n_suction <- as.integer(suction_data$clavien_iv_n_suction)
```

```
## Warning: NAs introduced by coercion
```

```r
suction_data$clavien_v_n_suction <- as.integer(suction_data$clavien_v_n_suction)
suction_data$death_n_control <- as.integer(suction_data$death_n_control)
```

```
## Warning: NAs introduced by coercion
```

```r
suction_data$death_n_suction <- as.integer(suction_data$death_n_suction)
```

```
## Warning: NAs introduced by coercion
```

```r
suction_data$vas_score_mean_suction <- as.numeric(suction_data$vas_score_mean_suction)
```

```
## Warning: NAs introduced by coercion
```

```r
suction_data$vas_score_sd_suction <-as.numeric(suction_data$vas_score_sd_suction)
```

```
## Warning: NAs introduced by coercion
```

```r
suction_data$vas_score_mean_control <- as.numeric(suction_data$vas_score_mean_control)
```

```
## Warning: NAs introduced by coercion
```

```r
suction_data$vas_score_sd_control <-as.numeric(suction_data$vas_score_sd_control)
```

```
## Warning: NAs introduced by coercion
```

```r
suction_data$stricture_n_control <- as.integer(suction_data$stricture_n_control)
```

```
## Warning: NAs introduced by coercion
```

```r
suction_data$stricture_n_suction <- as.integer(suction_data$stricture_n_suction)
```

```
## Warning: NAs introduced by coercion
```

1.2 Make new columns combining CD

```
suction_data <- suction_data %>% mutate(
  clav_i_ii_n_control = clavien_i_n_control + clavien_ii_n_control,
  clav_i_ii_n_suction = clavien_i_n_suction + clavien_ii_n_suction,
  clav_iii_v_n_control = clavien_iii_n_control + clavien_iv_n_control + clavien_v_n_control,
  clav_iii_v_n_suction = clavien_iii_n_suction + clavien_iv_n_suction + clavien_v_n_suction
)
```

2 Data Exploration

```
plot_missing(suction_data[1:10])
```

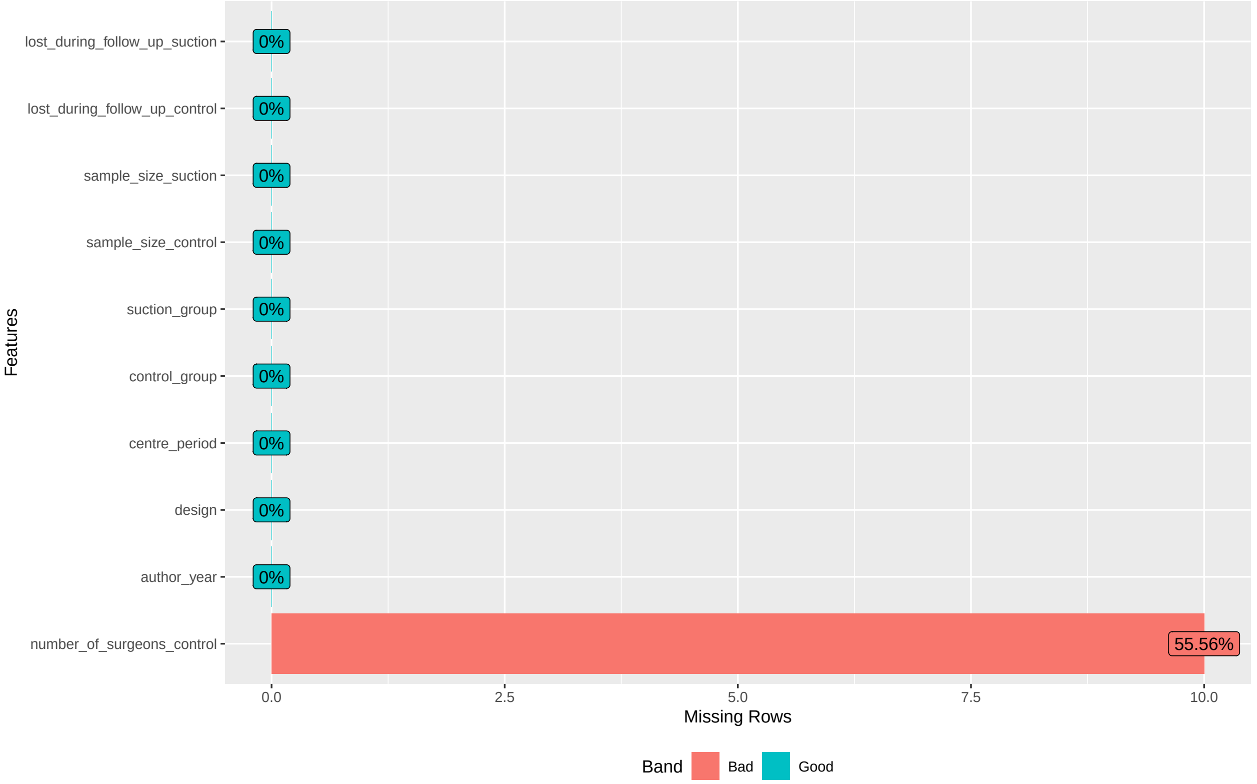


```
plot_missing(suction_data[11:20])
```

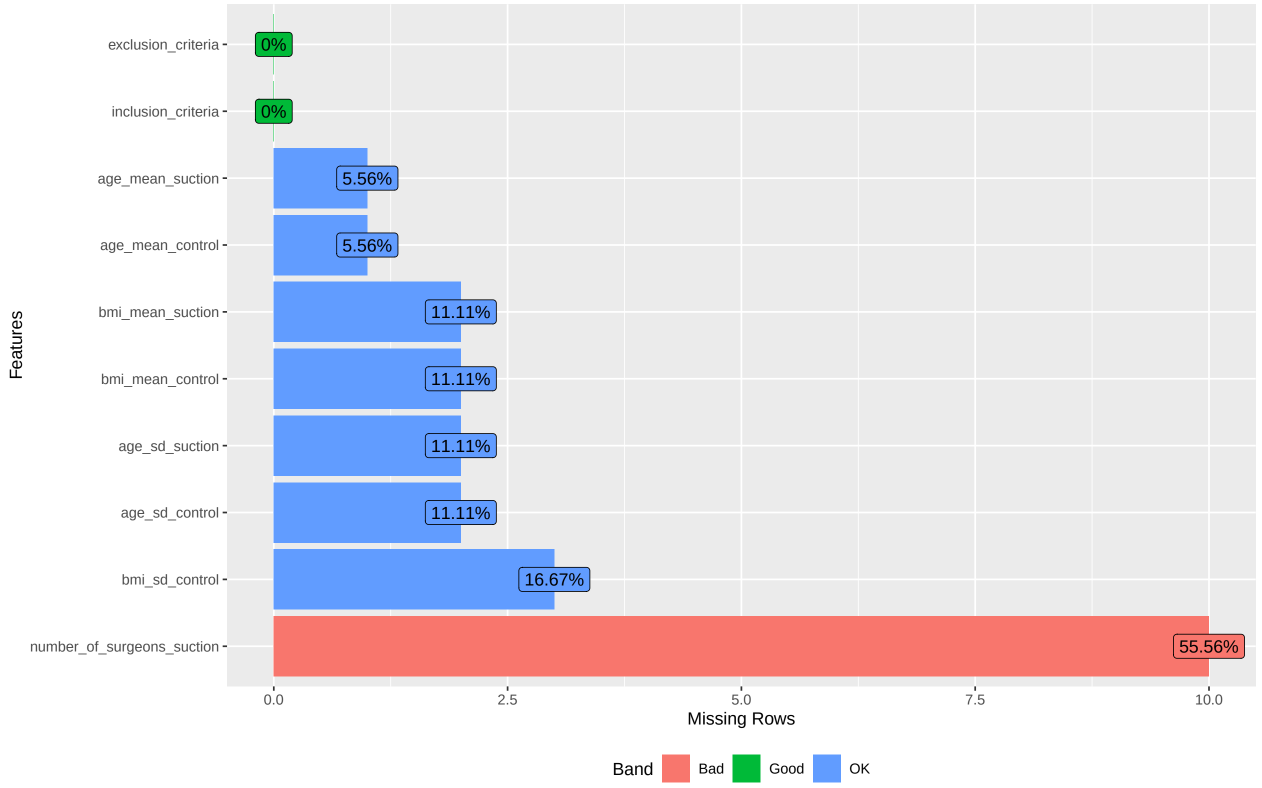


```r
plot_missing(suction_data[21:30])
```

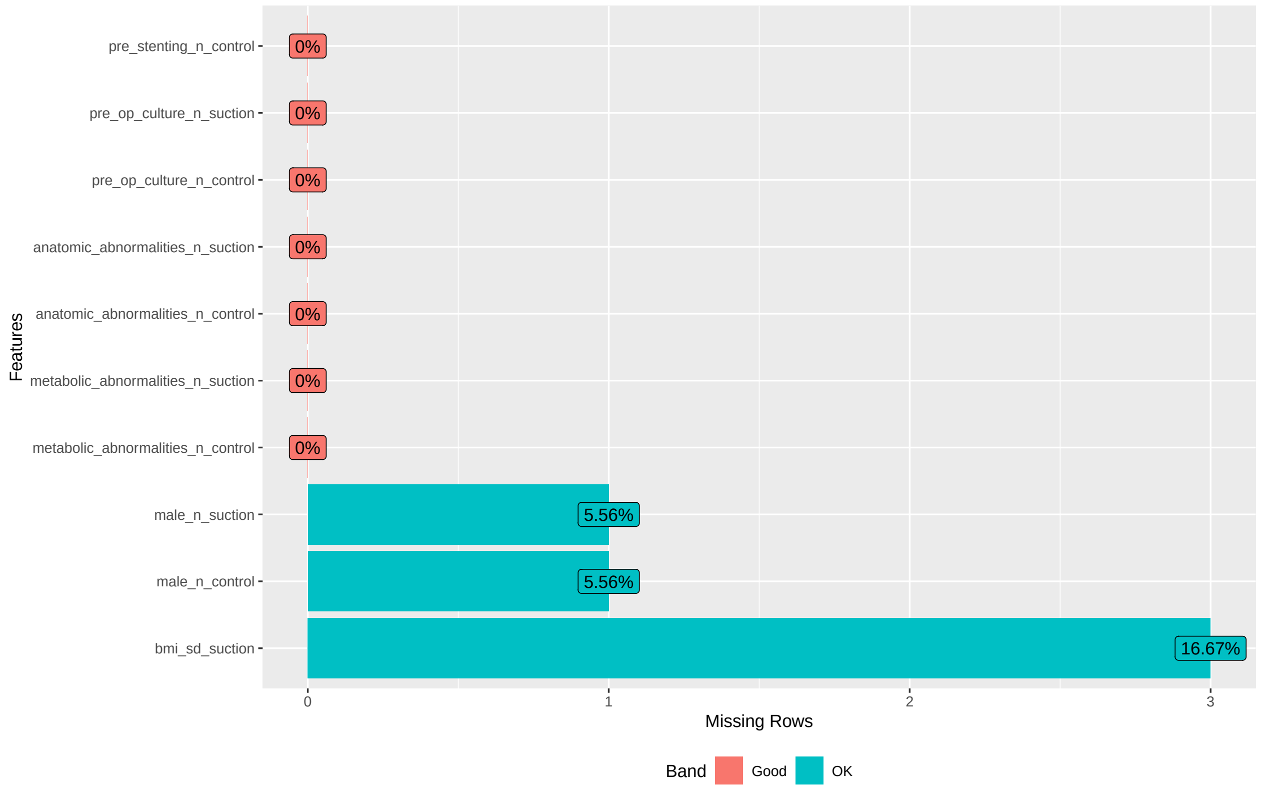

```r
plot_missing(suction_data[31:40])
```

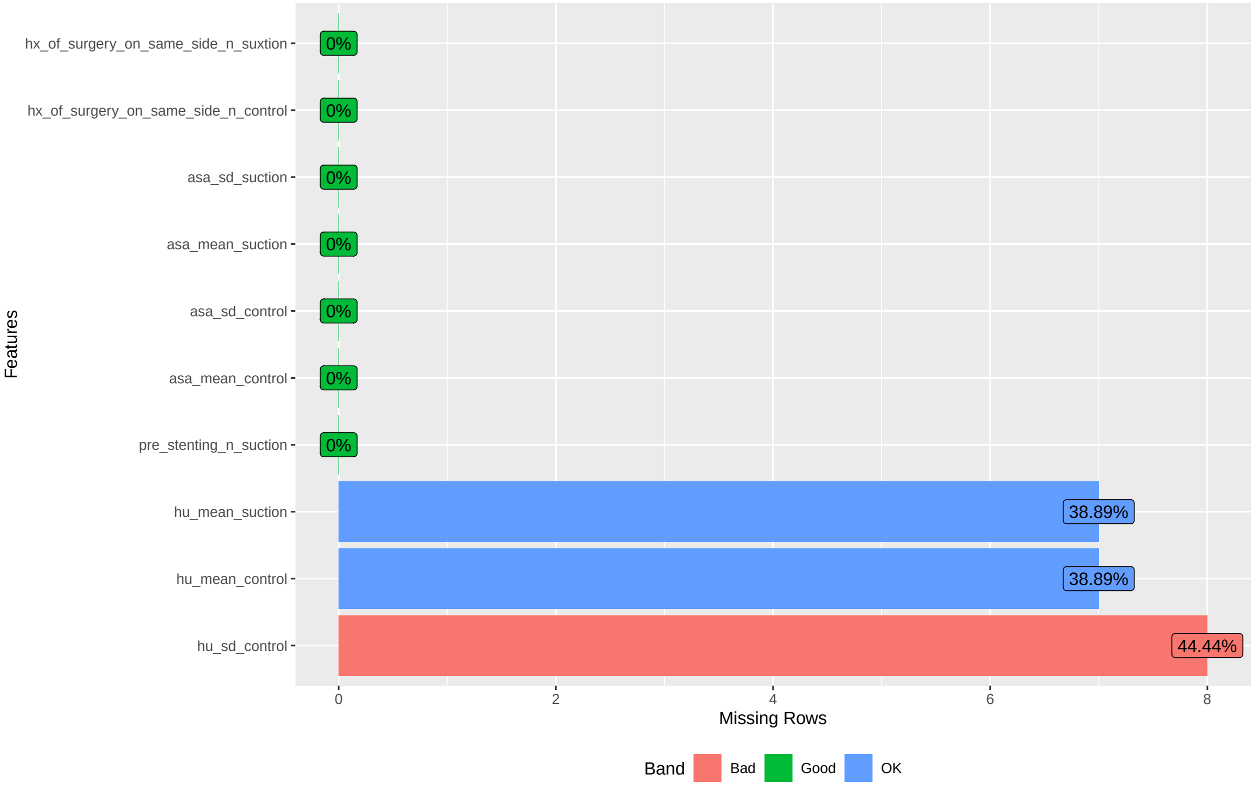


```r
plot_missing(suction_data[41:50])
```

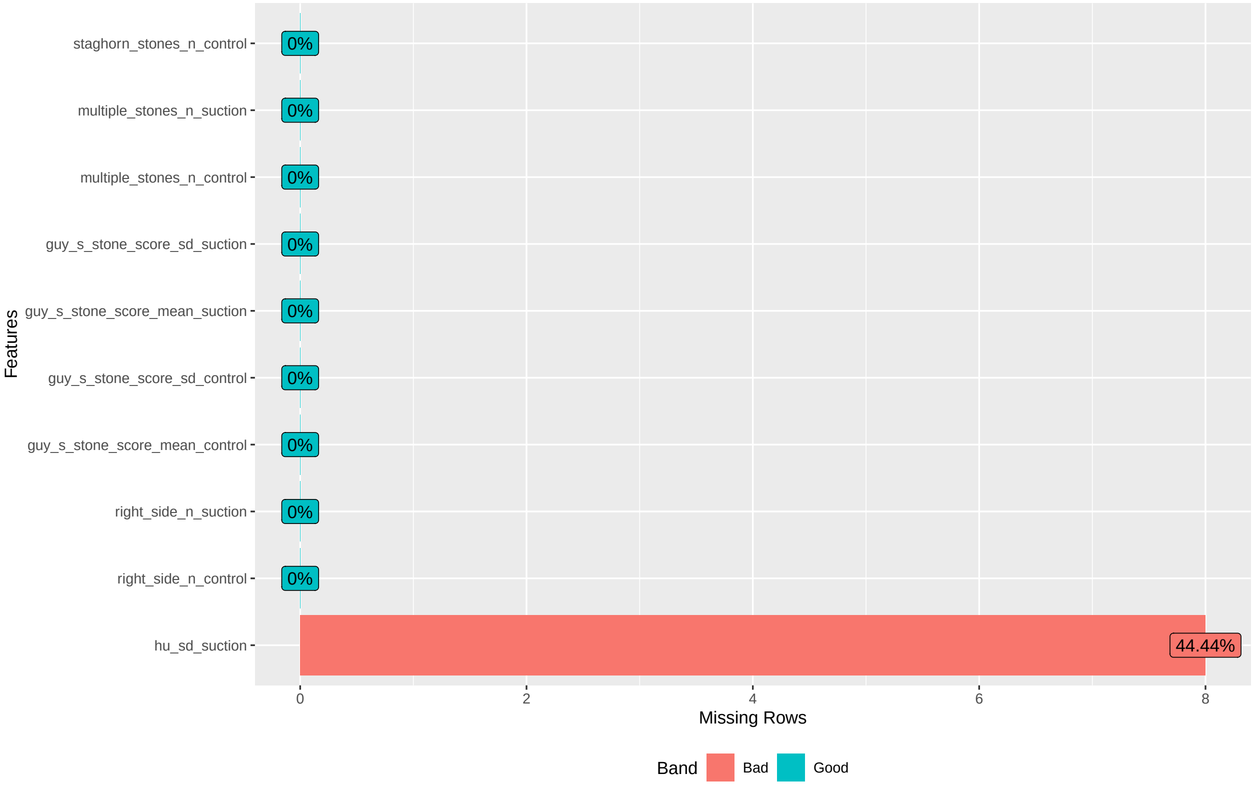

```r
plot_missing(suction_data[51:60])
```

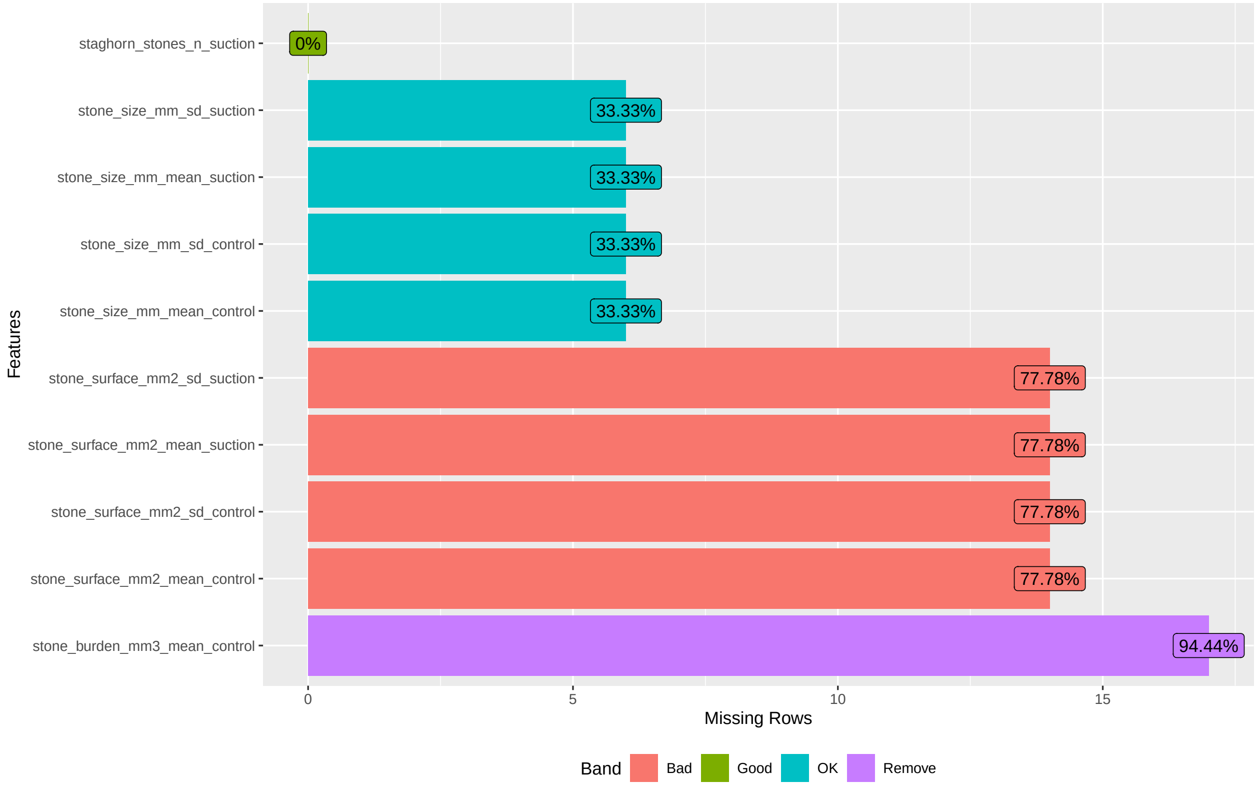


```r
plot_missing(suction_data[61:70])
```

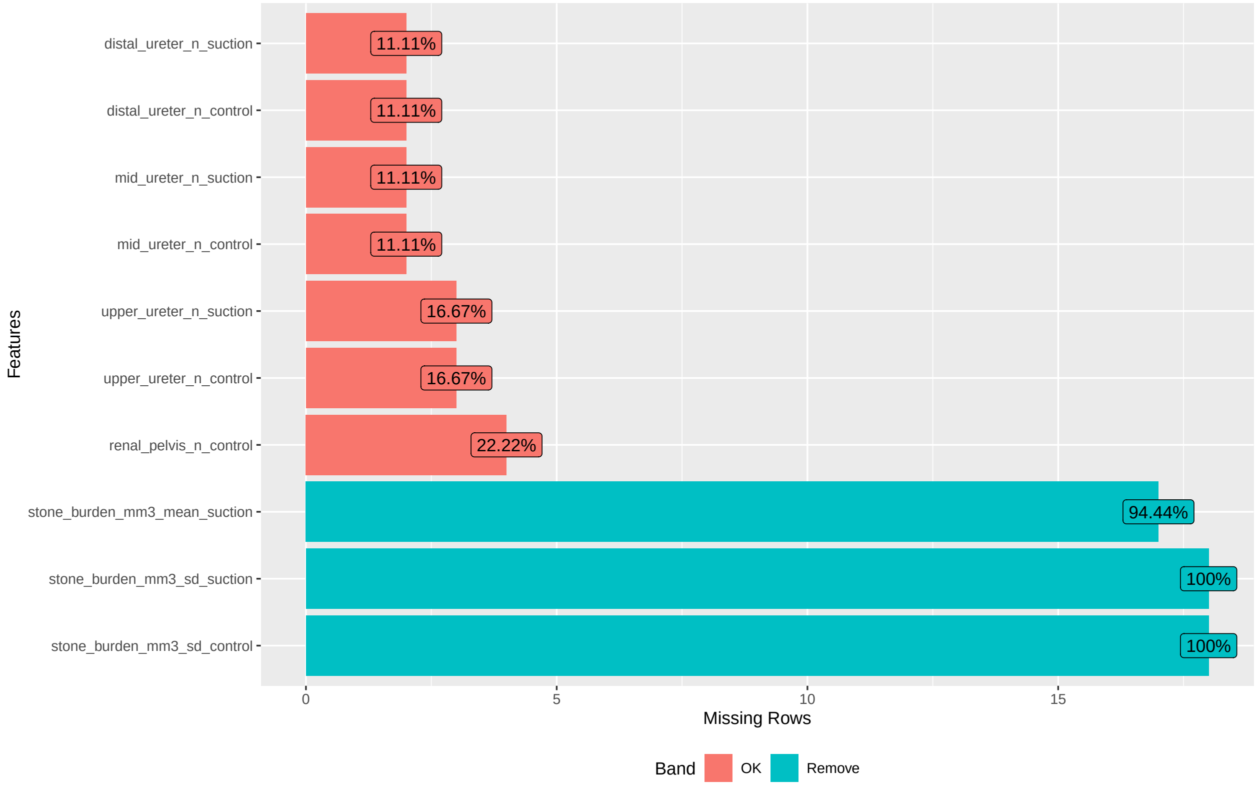

```r
plot_missing(suction_data[71:80])
```

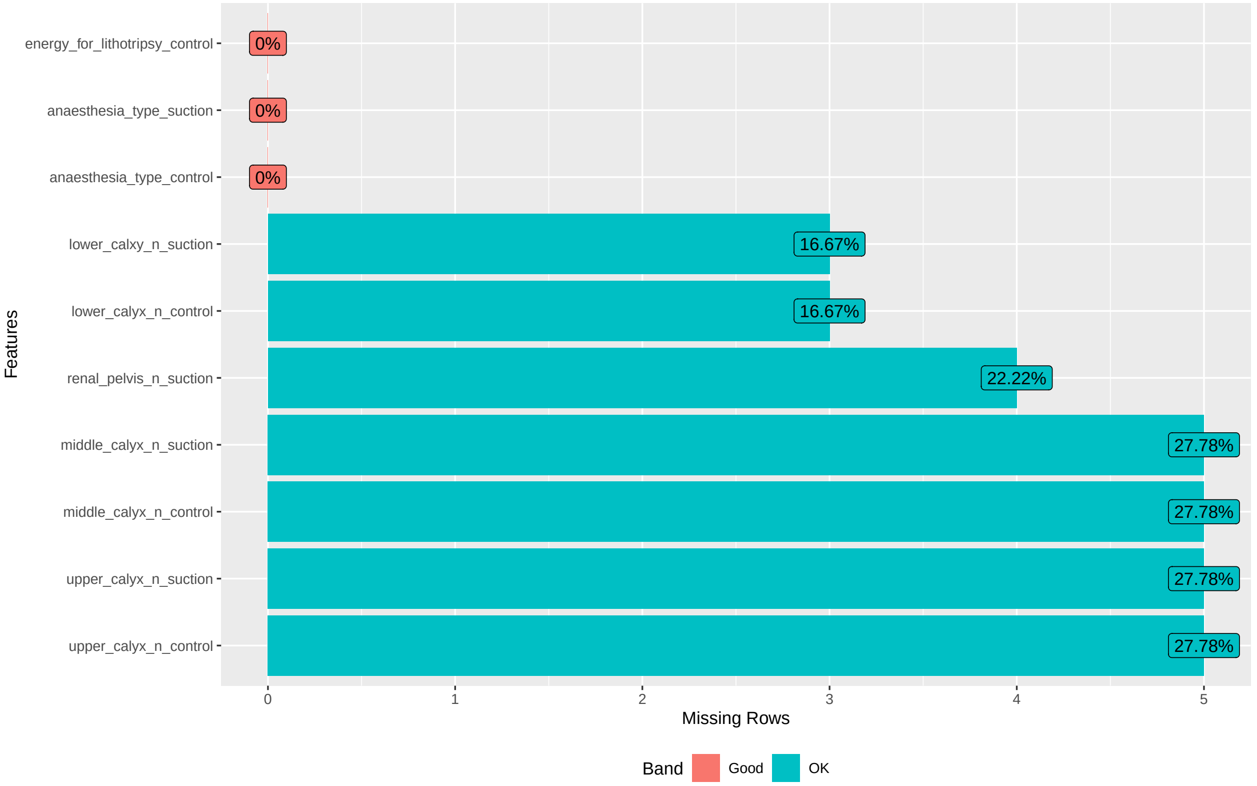


```r
plot_missing(suction_data[81:90])
```

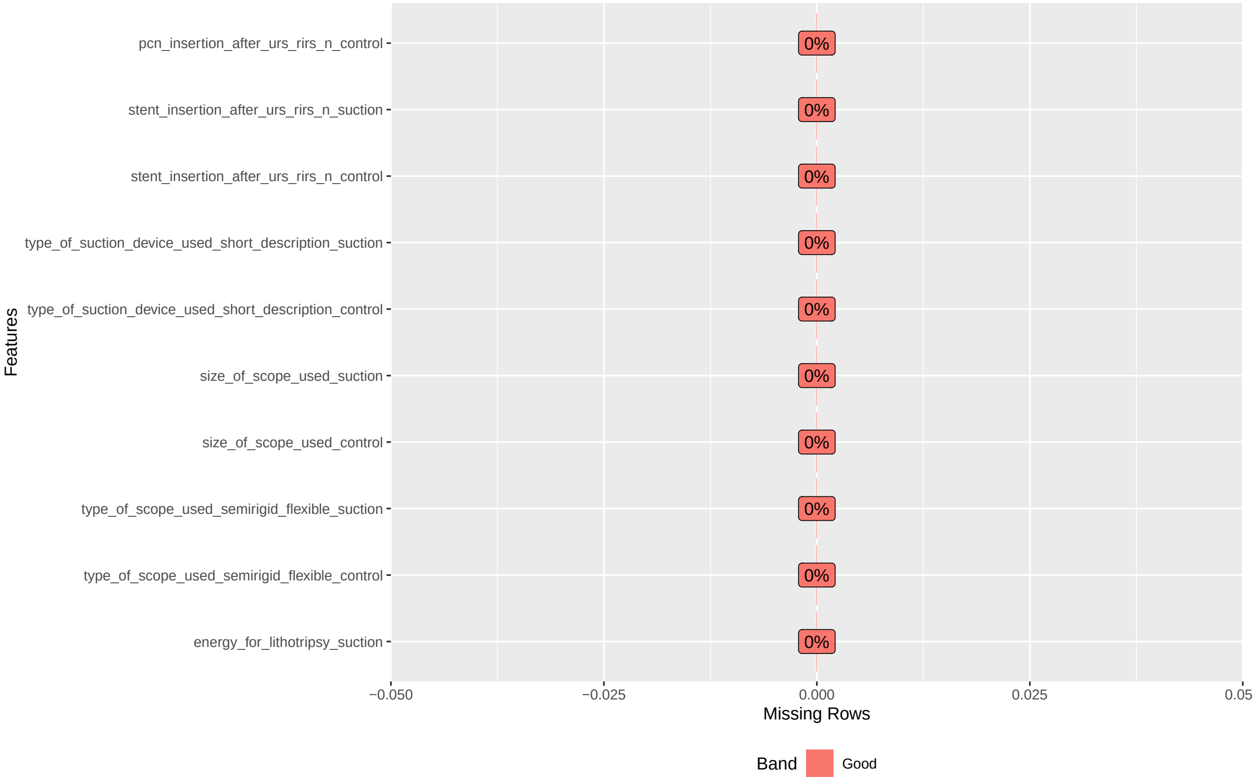

```r
plot_missing(suction_data[91:100])
```

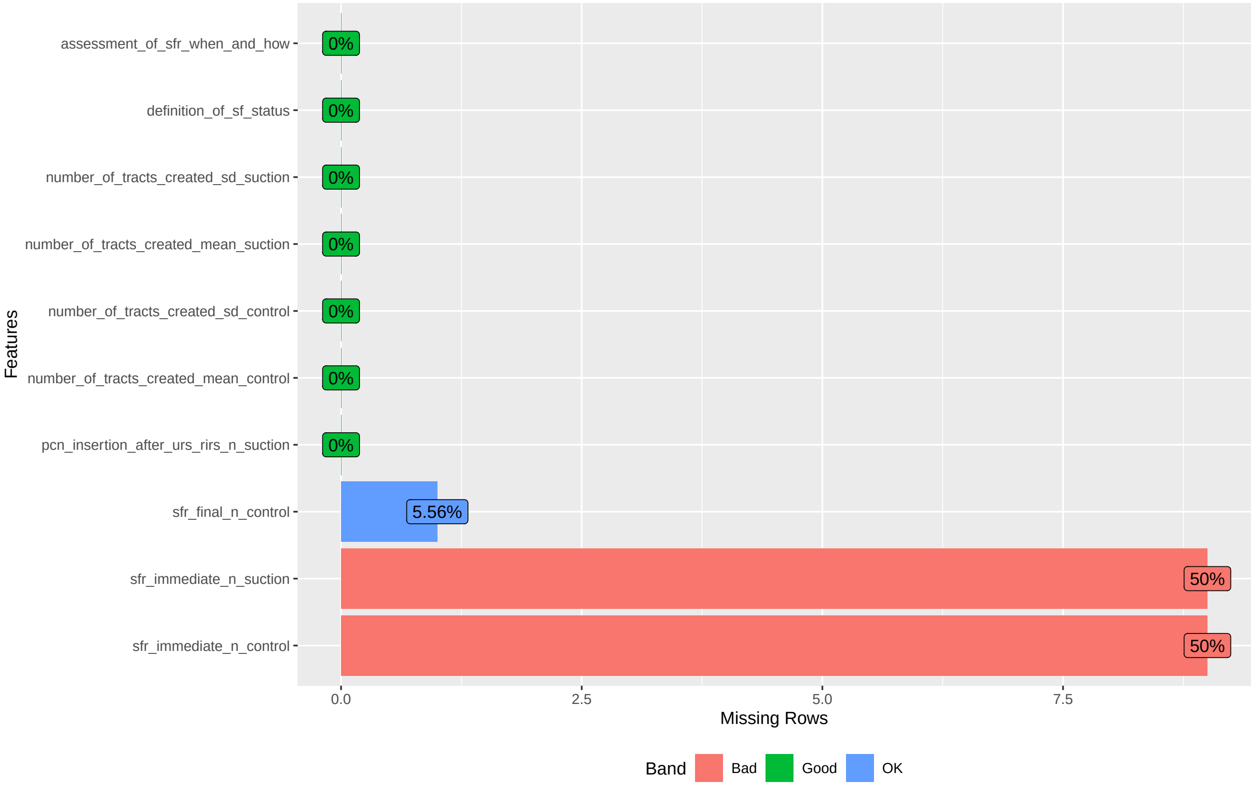


```r
plot_missing(suction_data[101:110])
```

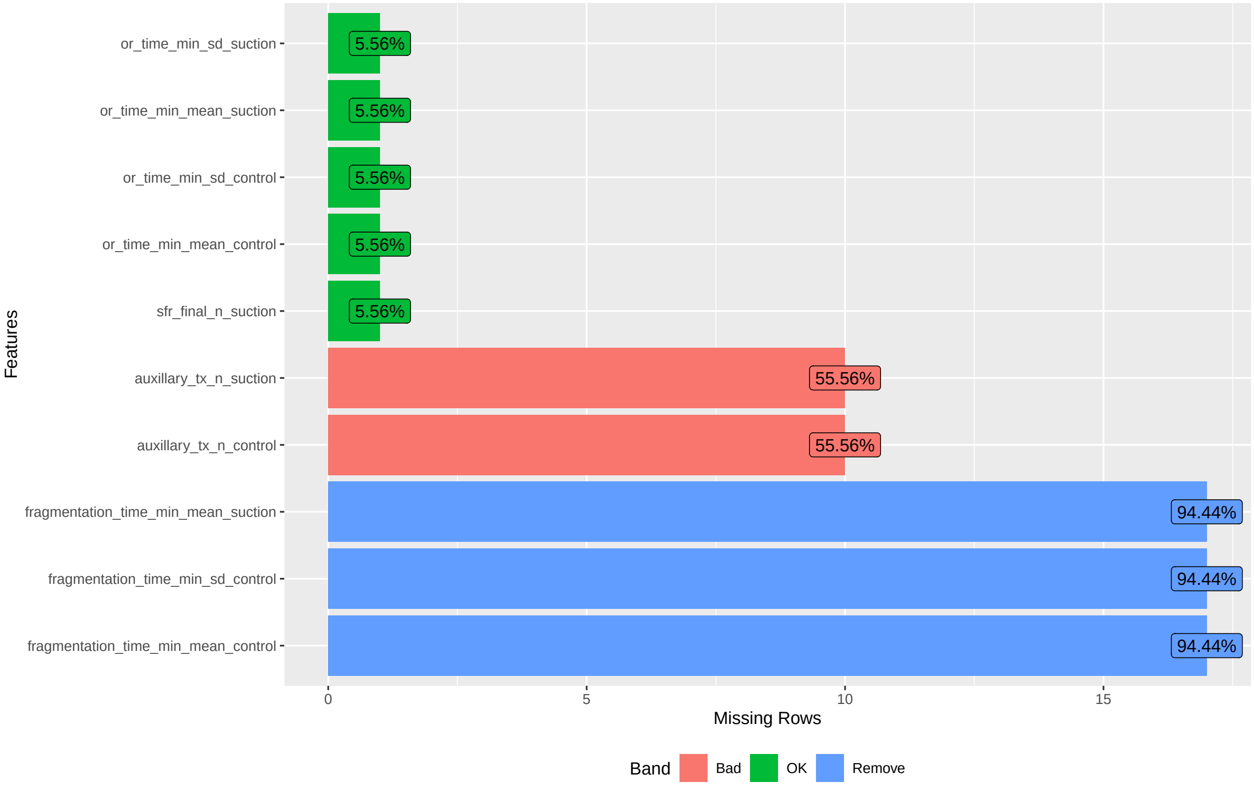

```r
plot_missing(suction_data[121:130])
```

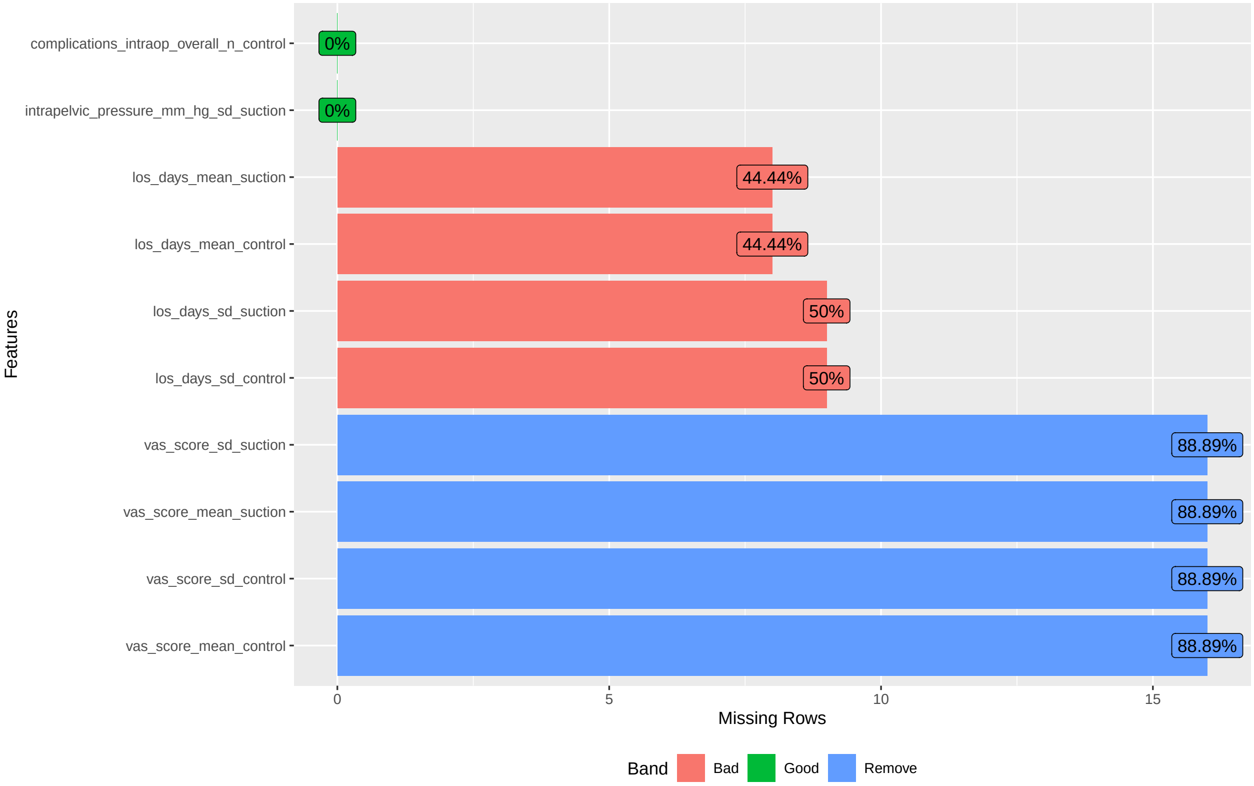


```r
plot_missing(suction_data[131:140])
```

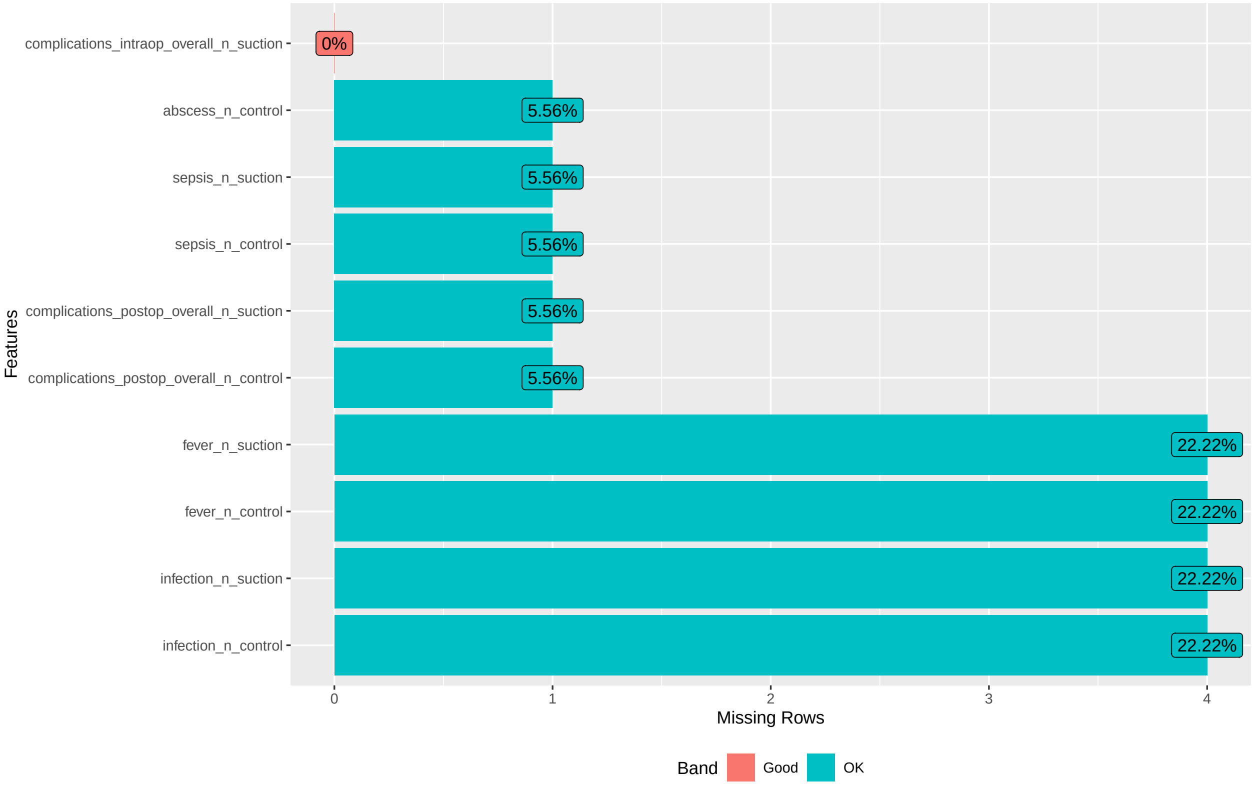

```r
plot_missing(suction_data[141:150])
```

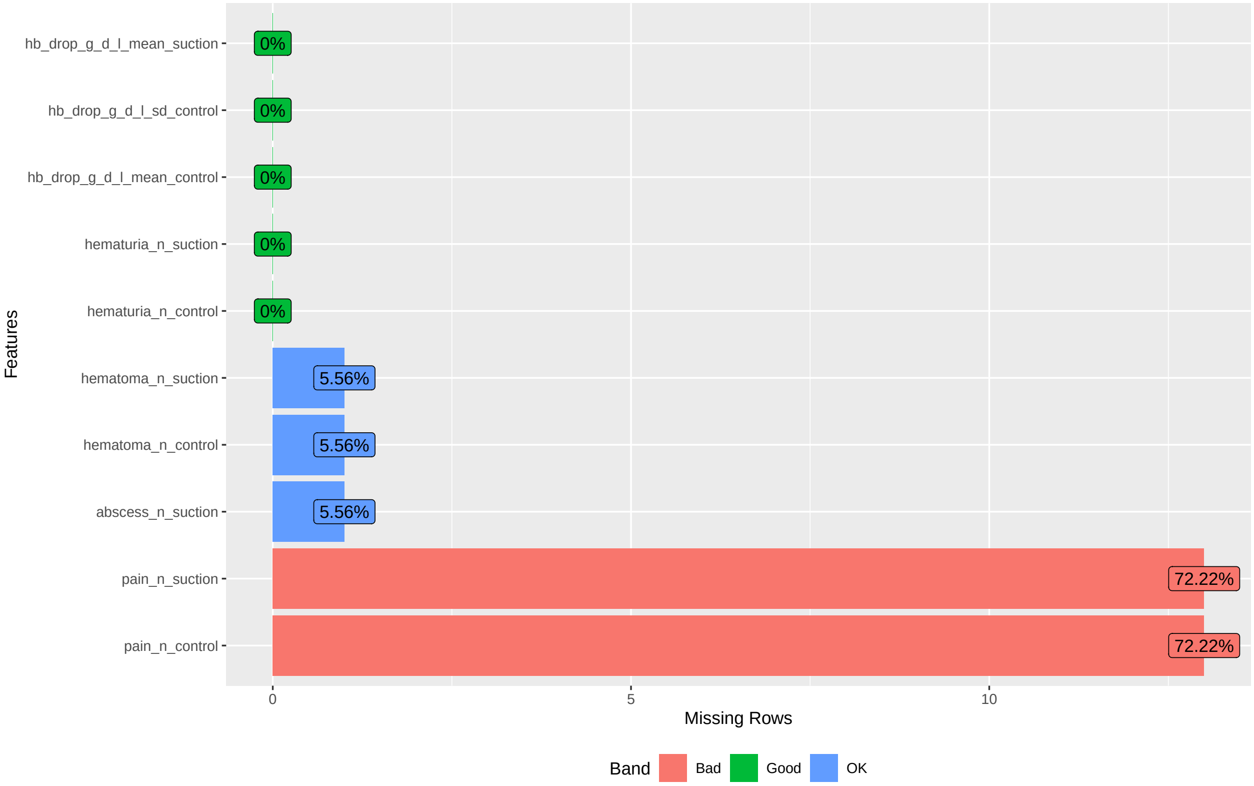


```r
plot_missing(suction_data[151:160])
```

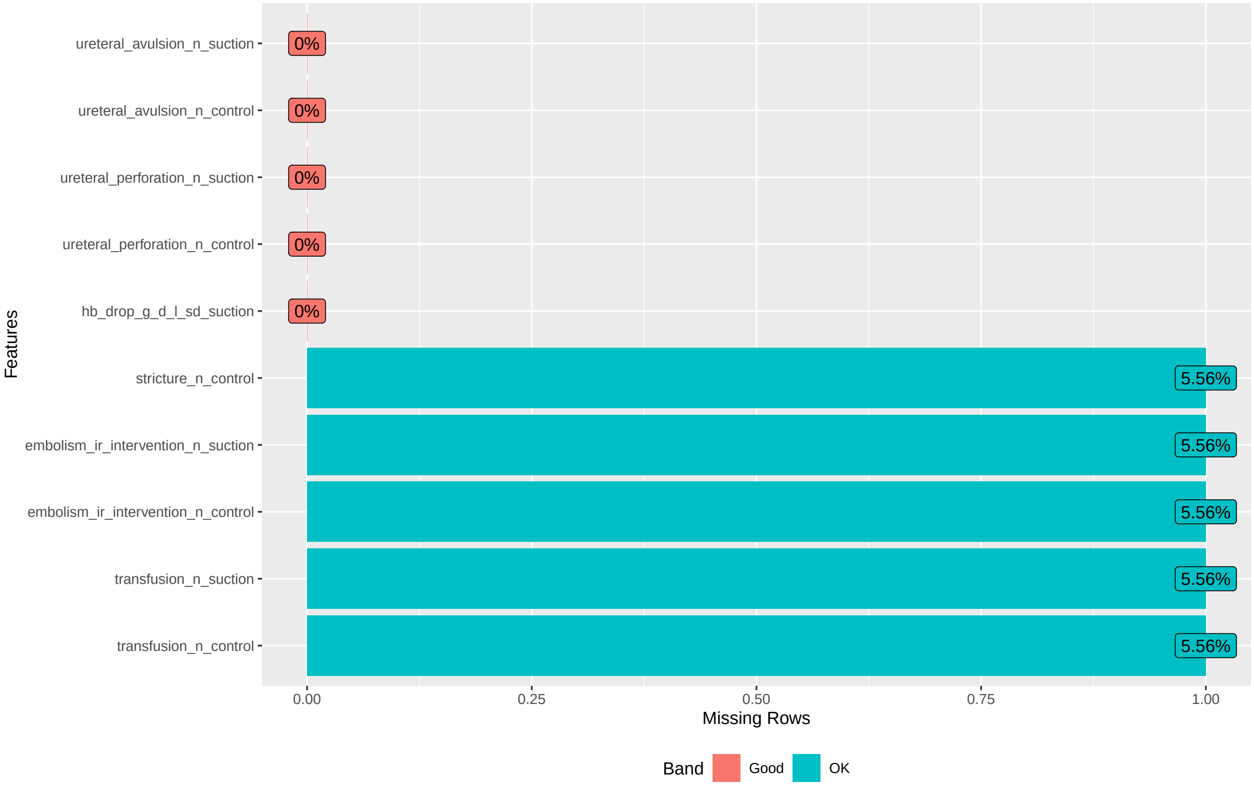

```r
plot_missing(suction_data[161:170])
```

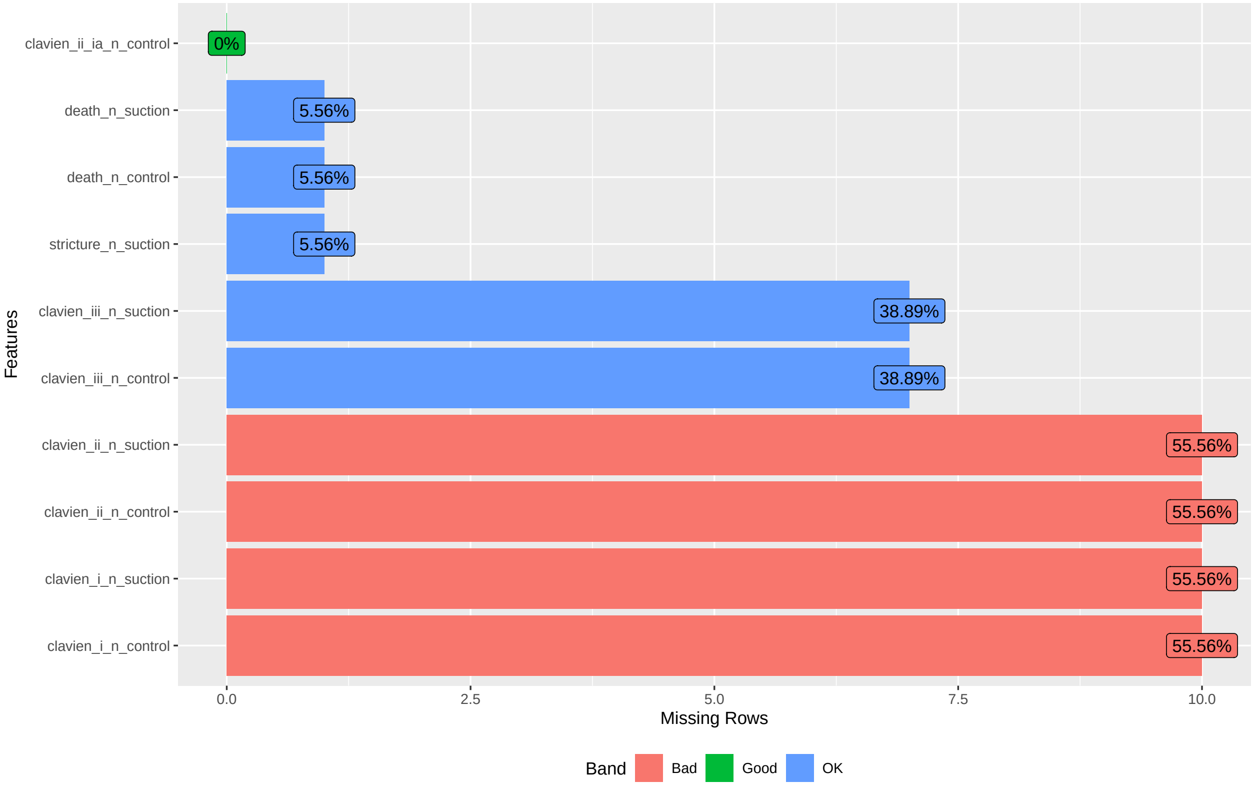


```r
plot_missing(suction_data[171:182])
```

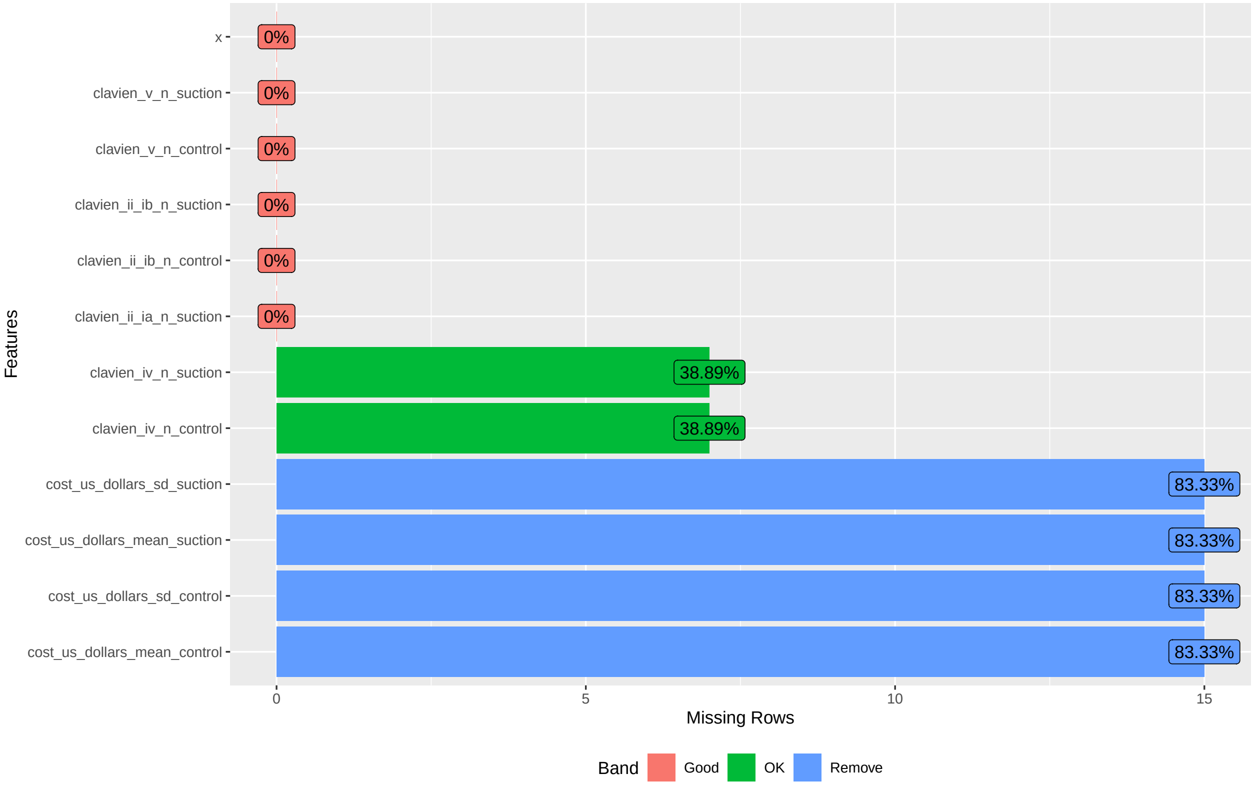

3 Demographic information

3.1 Number of Patients

```
total_n <- c(
  "Total",
  sum(suction_data$sample_size_control),
  sum(suction_data$sample_size_suction)
)

suction_data %>% subset(select = c(author_year,
                                   sample_size_control,
                                   sample_size_suction)) %>% rbind(total_n) %>% gt() %>% cols_label(
                                     author_year = "Study",
                                     sample_size_control = "Control, N",
                                     sample_size_suction = "Suction, N"
                                   )
```

Study	Control, N	Suction, N
Sur 2022	8	9
Tang 2023	87	86
Chen 2019	45	44
Du 2019	60	62
Lai 2020	56	28
Zhu 2018	165	165
Zhang 2021	50	56
Zhang 2021	54	56
Lechevallier 2003	14	11
Huang 2023	103	103
Wu 2022	82	76
Ding 2023	61	138
Zhai 2023	60	60
Zhai 2023	60	60
Qian 2022	81	81
Zhang 2022	30	30
Deng 2022	70	57
AlSmadi 2019	60	43
Total	1146	1165

3.2 Number of Surgeons

```
median_surgeons_n <- c(
  "Median",
  median(na.omit(suction_data$number_of_surgeons_control)),
  median(na.omit(suction_data$number_of_surgeons_suction))
)

suction_data %>% subset(select = c(author_year,
                                   number_of_surgeons_control,
                                   number_of_surgeons_suction)) %>% na.omit() %>% rbind(median_surgeons_
                                     author_year = "Study",
                                     number_of_surgeons_control = "Control, N",
                                     number_of_surgeons_suction = "Suction, N"
                                   )
```

Study	Control, N	Suction, N
Sur 2022	4	4
Tang 2023	1	1
Du 2019	1	1
Zhu 2018	3	3
Lechevallier 2003	1	1
Huang 2023	1	1
Zhai 2023	1	1
Zhai 2023	1	1
Median	1	1

3.3 Number of Male Patients

```
total_surgeons_n <- c(
  "Total",
  median(na.omit(suction_data$male_n_control)),
  median(na.omit(suction_data$male_n_suction))
)

suction_data %>% subset(select = c(author_year,
                                   male_n_control,
                                   male_n_suction)) %>% na.omit() %>% rbind(total_surgeons_n) %>% gt() %
                                     author_year = "Study",
                                     male_n_control = "Control, N",
                                     male_n_suction = "Suction, N"
                                   )
```

Study	Control, N	Suction, N
Sur 2022	5	8
Tang 2023	41	37
Chen 2019	23	21
Du 2019	36	37
Lai 2020	26	16
Zhu 2018	109	109
Zhang 2021	25	34
Zhang 2021	37	34
Huang 2023	68	71
Wu 2022	46	43
Ding 2023	39	89
Zhai 2023	32	34
Zhai 2023	32	35
Qian 2022	56	52
Zhang 2022	19	17
Deng 2022	47	32
AlSmadi 2019	24	18
Total	36	34

3.4 Comparisons - type of intervention

```
suction_data %>% subset(select = c(author_year,
                                   design,
                                   control_group,
                                   suction_group
                                   )) %>% gt()
```

author_year	design	control_group	suction_group
Sur 2022	RCT	Flexible URS	Flexible URS with suction
Tang 2023	RCT	mini-PCNL	Flexible URS with suction
Chen 2019	Retrospective	mini-PCNL	Flexible URS with suction
Du 2019	RCT	Semi-rigid URS	Semi-rigid URS with suction
Lai 2020	Retrospective	mini-PCNL	Flexible URS with suction
Zhu 2018	Retrospective	Flexible URS	Flexible URS with suction
Zhang 2021	Retrospective	Semi-rigid URS	Semi-rigid URS with suction
Zhang 2021	Retrospective	Flexible URS	Flexible URS with suction
Lechevallier 2003	RCT	Semi-rigid URS	Semi-rigid URS with suction
Huang 2023	Retrospective	Flexible URS	Flexible URS with suction
Wu 2022	Retrospective	Semi-rigid URS	Semi-rigid URS with suction
Ding 2023	Retrospective	Flexible URS	Flexible URS with suction
Zhai 2023	Retrospective	Semi-rigid URS	Semi-rigid URS with suction
Zhai 2023	Retrospective	Semi-rigid URS	Semi-rigid URS with suction
Qian 2022	Retrospective	Flexible URS	Flexible URS with suction
Zhang 2022	RCT	Flexible URS	Semi-rigid URS with suction (combined with flexible standard URS)
Deng 2022	Retrospective	mini-PCNL	Flexible URS with suction
AlSmadi 2019	Retrospective	Semi-rigid URS	Semi-rigid URS with suction

3.5 Age

```
suction_data %>% subset(select = c(author_year,
                                   age_mean_control,
                                   age_sd_control,
                                   age_mean_suction,
                                   age_sd_suction)) %>% gt() %>% cols_merge(columns = c(age_mean_contro
                                                                                        age_sd_control)
                                                                             pattern = "{1}±{2}") %>% co
                                                                                        age_sd_suction)
                                                                             pattern = "{1}±{2}") %>% co
```

Study	Control Age ± SD	Suction Age ± SD
Sur 2022	37.0±NA	42.0±NA
Tang 2023	51.3±8.2	52.7±9.3
Chen 2019	39.4±17.9	45.7±11.9
Du 2019	47.0±15.7	47.4±13.2
Lai 2020	49.6±12.2	45.2±10.4
Zhu 2018	51.7±15.8	53.9±13.4
Zhang 2021	54.3±11.6	53.8±12.1
Zhang 2021	55.2±10.2	53.8±12.1
Lechevallier 2003	NA±NA	NA±NA
Huang 2023	54.7±10.7	54.5±11.0
Wu 2022	44.9±12.7	48.5±12.4
Ding 2023	55.7±13.1	57.6±13.7
Zhai 2023	46.2±6.9	45.7±6.5
Zhai 2023	46.2±6.9	47.2±4.5
Qian 2022	50.0±10.6	50.0±11.3
Zhang 2022	55.7±10.8	53.5±12.9
Deng 2022	47.4±7.8	51.9±10.9
AlSmadi 2019	48.2±11.0	51.9±13.2

3.6 BMI

```
suction_data %>% subset(select = c(author_year,
                                   bmi_mean_control,
                                   bmi_sd_control,
                                   bmi_mean_suction,
                                   bmi_sd_suction)) %>% gt() %>% cols_merge(columns = c(bmi_mean_contro
                                                                                      bmi_sd_control)
                                                                           pattern = "{1}±{2}") %>% co
                                                                                      bmi_sd_suction)
                                                                           pattern = "{1}±{2}") %>% co
```

Study	Control BMI ± SD	Suction BMI ± SD
Sur 2022	23.4±NA	22.3±NA
Tang 2023	22.6±3.2	23.1±3.9
Chen 2019	27.3±4.4	28.2±8.2
Du 2019	NA±NA	NA±NA
Lai 2020	25.3±4.1	25.0±3.5
Zhu 2018	23.1±3.4	22.9±2.6
Zhang 2021	21.8±3.4	20.8±3.4
Zhang 2021	21.9±3.5	20.8±3.4
Lechevallier 2003	NA±NA	NA±NA
Huang 2023	26.5±4.9	26.3±4.2
Wu 2022	24.6±2.8	23.9±2.0
Ding 2023	24.0±2.6	24.6±3.2
Zhai 2023	26.2±4.1	25.0±3.4
Zhai 2023	26.2±4.1	24.9±3.1
Qian 2022	24.1±3.4	24.0±3.0
Zhang 2022	25.5±2.9	25.2±3.2
Deng 2022	19.8±5.7	20.3±4.4
AlSmadi 2019	24.0±1.7	22.8±1.5

3.7 HU

```
suction_data %>% subset(select = c(author_year,
                                   hu_mean_control,
                                   hu_sd_control,
                                   hu_mean_suction,
                                   hu_sd_suction)) %>% gt() %>% cols_merge(columns = c(hu_mean_control,
                                                                                        hu_sd_control),
                                                                                  pattern = "{1}±{2}") %>% co
                                                                                        hu_sd_suction),
                                                                                  pattern = "{1}±{2}") %>% co
```

Study	Control HU ± SD	Suction HU ± SD
Sur 2022	926.0±NA	786.0±NA
Tang 2023	NA±NA	NA±NA
Chen 2019	NA±NA	NA±NA
Du 2019	984.5±226.8	1022.8±215.3
Lai 2020	845.2±240.2	894.3±232.2
Zhu 2018	1023.0±175.0	1049.0±196.0
Zhang 2021	665.0±309.6	708.6±343.7
Zhang 2021	684.2±376.3	708.6±343.7
Lechevallier 2003	NA±NA	NA±NA
Huang 2023	NA±NA	NA±NA
Wu 2022	916.4±80.7	937.7±85.0
Ding 2023	715.3±340.8	751.6±429.3
Zhai 2023	912.3±52.9	906.5±63.5
Zhai 2023	912.3±52.9	897.1±94.2
Qian 2022	NA±NA	NA±NA
Zhang 2022	NA±NA	NA±NA
Deng 2022	NA±NA	NA±NA
AlSmadi 2019	776.5±120.1	895.6±70.4

3.8 Size Metrics

3.8.1 Size (mm) - missing data removed

```
suction_data %>% subset(select = c(author_year,
                                   stone_size_mm_mean_control,
                                   stone_size_mm_sd_control,
                                   stone_size_mm_mean_suction,
                                   stone_size_mm_sd_suction)) %>% drop_na(stone_size_mm_mean_control) %
                                                                                                      stone_size_mm_s
                                                                                          pattern = "{1}±{2}") %>% co
                                                                                                      stone_size_mm_s
                                                                                          pattern = "{1}±{2}") %>% co
```

Study	Control Size (mm) ± SD	Suction Size (mm) ± SD
Tang 2023	15.0±5.0	16.0±4.0
Du 2019	21.4±3.6	21.9±4.9
Lai 2020	38.2±5.4	35.3±6.3
Zhu 2018	17.4±4.7	18.2±5.2
Zhang 2021	12.5±4.9	13.9±4.7
Zhang 2021	12.7±5.5	13.9±4.7
Huang 2023	17.0±5.0	17.0±6.0
Ding 2023	13.4±5.2	13.0±6.9
Qian 2022	20.0±4.5	19.7±4.5
Zhang 2022	18.5±5.6	18.2±5.3
Deng 2022	24.9±7.9	23.1±6.5
AlSmadi 2019	14.9±1.8	12.7±1.2

3.8.2 Area (mm^2) - missing data removed

```
suction_data %>% subset(select = c(author_year,
                                   stone_surface_mm2_mean_control,
                                   stone_surface_mm2_sd_control,
                                   stone_surface_mm2_mean_suction,
                                   stone_surface_mm2_sd_suction)) %>% drop_na(stone_surface_mm2_mean_co
                                                                            stone_surface_m
                                                                    pattern = "{1}±{2}") %>% co
                                                                            stone_surface_m
                                                                    pattern = "{1}±{2}") %>% co
```

Study	Control Area (mm^2) ± SD	Suction Area (mm^2) ± SD
Lai 2020	729.0±83.7	676.1±42.2
Wu 2022	157.0±34.6	165.3±33.4
Zhai 2023	131.8±25.1	136.5±26.6
Zhai 2023	131.8±25.1	135.9±24.8

3.8.3 Volume (mm^3) - missing data removed

```
suction_data %>% subset(select = c(author_year,
                                   stone_burden_mm3_mean_control,
                                   stone_burden_mm3_sd_control,
                                   stone_burden_mm3_mean_suction,
                                   stone_burden_mm3_sd_suction)) %>% drop_na(stone_burden_mm3_mean_cont
                                                                               stone_burden_mm3
                                                                  pattern = "{1}±{2}") %>% co
                                                                               stone_burden_mm3
                                                                  pattern = "{1}±{2}") %>% co
```

Study	Control Volume (mm^3) ± SD	Suction Volume (mm^3) ± SD
Sur 2022	210±NA	267±NA

3.9 Stone Location

3.9.1 Distal Ureter

```
total_distal_n <- c(
  "Total",
  sum(na.omit(suction_data$distal_ureter_n_control)),
  sum(na.omit(suction_data$distal_ureter_n_suction))
)

suction_data %>% subset(select = c(author_year,
                                  distal_ureter_n_control,
                                  distal_ureter_n_suction)) %>% na.omit() %>% rbind(total_distal_n) %>
                                    author_year = "Study",
                                    distal_ureter_n_control = "Control, N",
                                    distal_ureter_n_suction = "Suction, N"
                                  )
```

Study	Control, N	Suction, N
Sur 2022	0	0
Tang 2023	0	0
Chen 2019	0	0
Du 2019	27	26
Lai 2020	0	0
Zhang 2021	0	0
Zhang 2021	0	0
Huang 2023	0	0
Wu 2022	0	0
Ding 2023	0	0
Zhai 2023	30	30
Zhai 2023	30	20
Qian 2022	0	0
Zhang 2022	0	0
Deng 2022	0	0
AlSmadi 2019	0	0
Total	87	76

3.9.2 Mid Ureter

```
total_mid_n <- c(
  "Total",
  sum(na.omit(suction_data$mid_ureter_n_control)),
  sum(na.omit(suction_data$mid_ureter_n_suction))
)

suction_data %>% subset(select = c(author_year,
                                   mid_ureter_n_control,
                                   mid_ureter_n_suction)) %>% na.omit() %>% rbind(total_mid_n) %>% gt()
                                     author_year = "Study",
                                     mid_ureter_n_control = "Control, N",
                                     mid_ureter_n_suction = "Suction, N"
                                   )
```

Study	Control, N	Suction, N
Sur 2022	0	0
Tang 2023	0	0
Chen 2019	0	0
Du 2019	13	15
Lai 2020	0	0
Zhang 2021	0	0
Zhang 2021	0	0
Huang 2023	0	0
Wu 2022	0	0
Ding 2023	0	0
Zhai 2023	17	18
Zhai 2023	17	28
Qian 2022	0	0
Zhang 2022	0	0
Deng 2022	0	0
AlSmadi 2019	0	0
Total	47	61

3.9.3 Upper Ureter

```r
total_upper_n <- c(
  "Total",
  sum(na.omit(suction_data$upper_ureter_n_control)),
  sum(na.omit(suction_data$upper_ureter_n_suction))
)

suction_data %>% subset(select = c(author_year,
                                   upper_ureter_n_control,
                                   upper_ureter_n_suction)) %>% na.omit() %>% rbind(total_upper_n) %>%
                                     author_year = "Study",
                                     upper_ureter_n_control = "Control, N",
                                     upper_ureter_n_suction = "Suction, N"
                                   )
```

Study	Control, N	Suction, N
Sur 2022	0	0
Tang 2023	87	86
Chen 2019	0	0
Du 2019	20	21
Lai 2020	0	0
Zhang 2021	50	56
Zhang 2021	54	56
Wu 2022	82	76
Ding 2023	13	26
Zhai 2023	13	12
Zhai 2023	13	12
Qian 2022	0	0
Zhang 2022	0	0
Deng 2022	0	0
AlSmadi 2019	60	43
Total	392	388

3.9.4 Renal Pelvis

```
total_renal_pelvis_n <- c(
  "Total",
  sum(na.omit(suction_data$renal_pelvis_n_control)),
  sum(na.omit(suction_data$renal_pelvis_n_suction))
)

suction_data %>% subset(select = c(author_year,
                                   renal_pelvis_n_control,
                                   renal_pelvis_n_suction)) %>% na.omit() %>% rbind(total_renal_pelvis_r
                                     author_year = "Study",
                                     renal_pelvis_n_control = "Control, N",
                                     renal_pelvis_n_suction = "Suction, N"
                                   )
```

Study	Control, N	Suction, N
Tang 2023	0	0
Chen 2019	0	0
Du 2019	0	0
Lai 2020	56	28
Zhu 2018	23	29
Zhang 2021	0	0
Zhang 2021	0	0
Wu 2022	0	0
Ding 2023	35	98
Zhai 2023	0	0
Zhai 2023	0	0
Qian 2022	0	0
Deng 2022	5	6
AlSmadi 2019	0	0
Total	119	161

3.9.5 Lower Calyx

```
total_lower_calyx_n <- c(
  "Total",
  sum(na.omit(suction_data$lower_calyx_n_control)),
  sum(na.omit(suction_data$lower_calxy_n_suction))
)

suction_data %>% subset(select = c(author_year,
                                   lower_calyx_n_control,
                                   lower_calxy_n_suction)) %>% na.omit() %>% rbind(total_lower_calyx_n)
                                     author_year = "Study",
                                     lower_calyx_n_control = "Control, N",
                                     lower_calxy_n_suction = "Suction, N"
                                   )
```

Study	Control, N	Suction, N
Sur 2022	5	7
Tang 2023	0	0
Chen 2019	13	11
Du 2019	0	0
Lai 2020	22	11
Zhu 2018	42	40
Zhang 2021	0	0
Zhang 2021	0	0
Wu 2022	0	0
Ding 2023	29	64
Zhai 2023	0	0
Zhai 2023	0	0
Qian 2022	25	34
Deng 2022	5	3
AlSmadi 2019	0	0
Total	141	170

3.9.6 middle Calyx

```
total_middle_calyx_n <- c(
  "Total",
  sum(na.omit(suction_data$middle_calyx_n_control)),
  sum(na.omit(suction_data$middle_calyx_n_suction))
)

suction_data %>% subset(select = c(author_year,
                                   middle_calyx_n_control,
                                   middle_calyx_n_suction)) %>% na.omit() %>% rbind(total_middle_calyx_
                                     author_year = "Study",
                                     middle_calyx_n_control = "Control, N",
                                     middle_calyx_n_suction = "Suction, N"
                                   )
```

Study	Control, N	Suction, N
Tang 2023	0	0
Chen 2019	16	18
Du 2019	0	0
Lai 2020	28	15
Zhu 2018	35	27
Zhang 2021	0	0
Zhang 2021	0	0
Wu 2022	0	0
Ding 2023	5	11
Zhai 2023	0	0
Zhai 2023	0	0
Deng 2022	7	4
AlSmadi 2019	0	0
Total	91	75

3.9.7 Upper Calyx

```
total_upper_calyx_n <- c(
  "Total",
  sum(na.omit(suction_data$upper_calyx_n_control)),
  sum(na.omit(suction_data$upper_calyx_n_suction))
)

suction_data %>% subset(select = c(author_year,
                                   upper_calyx_n_control,
                                   upper_calyx_n_suction)) %>% na.omit() %>% rbind(total_upper_calyx_n)
                                     author_year = "Study",
                                     upper_calyx_n_control = "Control, N",
                                     upper_calyx_n_suction = "Suction, N"
                                   )
```

Study	Control, N	Suction, N
Tang 2023	0	0
Chen 2019	16	17
Du 2019	0	0
Lai 2020	22	13
Zhu 2018	18	14
Zhang 2021	0	0
Zhang 2021	0	0
Wu 2022	0	0
Ding 2023	2	3
Zhai 2023	0	0
Zhai 2023	0	0
Deng 2022	14	15
AlSmadi 2019	0	0
Total	72	62

4 Overall Meta-Analysis Outcomes

4.1 Immediate SFR

4.1.1 Meta-analysis

```
immediate_sfr_metabin <- metabin(data = suction_data,
                          event.c = sfr_immediate_n_control,
                        n.c = sample_size_control,
                        event.e = sfr_immediate_n_suction,
                        n.e = sample_size_suction,
                        studlab = author_year)
immediate_sfr_metabin
```

```
## Number of studies: k = 9
## Number of observations: o = 2311
## Number of events: e = 979
##
##                          RR            95%-CI    z  p-value
## Common effect model  1.1581 [1.0834; 1.2380] 4.31 < 0.0001
## Random effects model 1.1478 [0.9854; 1.3370] 1.77   0.0765
##
## Quantifying heterogeneity:
##  tau^2 = 0.0365 [0.0088; 0.2318]; tau = 0.1910 [0.0939; 0.4814]
##  I^2 = 74.6% [50.9%; 86.9%]; H = 1.99 [1.43; 2.76]
##
## Test of heterogeneity:
##      Q d.f. p-value
##  31.55    8  0.0001
##
## Details on meta-analytical method:
## - Mantel-Haenszel method
## - Inverse variance method
## - Restricted maximum-likelihood estimator for tau^2
## - Q-Profile method for confidence interval of tau^2 and tau
```

4.1.2 Forest plot

```r
forest(immediate_sfr_metabin,
       sortvar = TE)
```

Study	Experimental Events	Experimental Total	Control Events	Control Total	RR	95%−CI	Weight (common)	Weight (random)
Lai 2020	14	28	41	56	0.68	[0.46; 1.02]	6.1%	7.7%
Tang 2023	63	86	75	87	0.85	[0.73; 0.99]	16.6%	14.2%
Zhu 2018	136	165	118	165	1.15	[1.02; 1.30]	26.3%	15.1%
Qian 2022	70	81	58	81	1.21	[1.03; 1.42]	12.9%	14.0%
AlSmadi 2019	40	43	46	60	1.21	[1.03; 1.43]	8.6%	14.0%
Wu 2022	67	76	59	82	1.23	[1.05; 1.44]	12.6%	14.1%
Zhang 2021	18	56	13	54	1.34	[0.73; 2.45]	2.9%	4.6%
Huang 2023	81	103	52	103	1.56	[1.25; 1.93]	11.6%	12.5%
Zhang 2021	18	56	10	50	1.61	[0.82; 3.15]	2.4%	3.9%
Sur 2022	.	9	.	8			0.0%	0.0%
Chen 2019	.	44	.	45			0.0%	0.0%
Du 2019	.	62	.	60			0.0%	0.0%
Lechevallier 2003	.	11	.	14			0.0%	0.0%
Ding 2023	.	138	.	61			0.0%	0.0%
Zhai 2023	.	60	.	60			0.0%	0.0%
Zhai 2023	.	60	.	60			0.0%	0.0%
Zhang 2022	.	30	.	30			0.0%	0.0%
Deng 2022	.	57	.	70			0.0%	0.0%
**Common effect model**		**1165**		**1146**	**1.16**	**[1.08; 1.24]**	**100.0%**	**--**
**Random effects model**					**1.15**	**[0.99; 1.34]**	**--**	**100.0%**

Heterogeneity: $I^2 = 75\%$, $\tau^2 = 0.0365$, $p < 0.01$

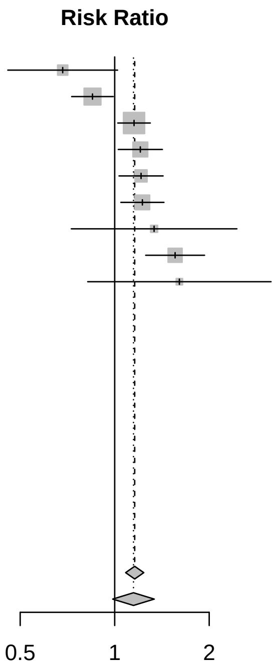

4.1.3 Trim and Fill

```r
trimfill(immediate_sfr_metabin)
```

```
## Warning in trimfill.meta(immediate_sfr_metabin): 9 observation(s) dropped due
## to missing values

## Number of studies: k = 11 (with 2 added studies)
## Number of observations: o = 1744
## Number of events: e = 1140
##
##                           RR           95%-CI    z p-value
## Random effects model 1.0855 [0.9289; 1.2685] 1.03  0.3019
##
## Quantifying heterogeneity:
##  tau^2 = 0.0471 [0.0138; 0.2196]; tau = 0.2170 [0.1173; 0.4686]
##  I^2 = 76.9% [58.8%; 87.1%]; H = 2.08 [1.56; 2.78]
##
## Test of heterogeneity:
##      Q d.f.  p-value
##  43.33   10 < 0.0001
##
## Details on meta-analytical method:
## - Inverse variance method
## - Restricted maximum-likelihood estimator for tau^2
## - Q-Profile method for confidence interval of tau^2 and tau
## - Trim-and-fill method to adjust for funnel plot asymmetry (L-estimator)
```

```r
forest(trimfill(immediate_sfr_metabin),
       sortvar = TE)
```

```
## Warning in trimfill.meta(immediate_sfr_metabin): 9 observation(s) dropped due
## to missing values
```

Study	logRR	SE(logRR)	Experimental Total	Control Total	Risk Ratio	RR	95%−CI	Weight
Lai 2020	−0.3814	0.2055	28	56		0.68	[0.46; 1.02]	7.1%
Filled: Zhang 2021	−0.2721	0.3431	56	50		0.76	[0.39; 1.49]	3.8%
Filled: Huang 2023	−0.2408	0.1103	103	103		0.79	[0.63; 0.98]	10.7%
Tang 2023	−0.1628	0.0780	86	87		0.85	[0.73; 0.99]	11.9%
Zhu 2018	0.1420	0.0609	165	165		1.15	[1.02; 1.30]	12.4%
Qian 2022	0.1881	0.0827	81	81		1.21	[1.03; 1.42]	11.7%
AlSmadi 2019	0.1934	0.0826	43	60		1.21	[1.03; 1.43]	11.7%
Wu 2022	0.2031	0.0808	76	82		1.23	[1.05; 1.44]	11.8%
Zhang 2021	0.2891	0.3100	56	54		1.34	[0.73; 2.45]	4.4%
Huang 2023	0.4432	0.1103	103	103		1.56	[1.25; 1.93]	10.7%
Zhang 2021	0.4745	0.3431	56	50		1.61	[0.82; 3.15]	3.8%
**Random effects model**			**853**	**891**		**1.09**	**[0.93; 1.27]**	**100.0%**

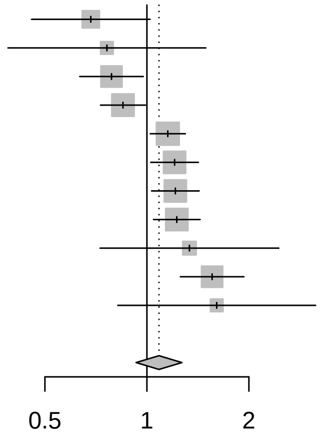

Heterogeneity: $I^2 = 77\%$, $\tau^2 = 0.0471$, $p < 0.01$

4.1.4 Funnel plot

```
funnel(trimfill(immediate_sfr_metabin))
```

```
## Warning in trimfill.meta(immediate_sfr_metabin): 9 observation(s) dropped due
## to missing values
```

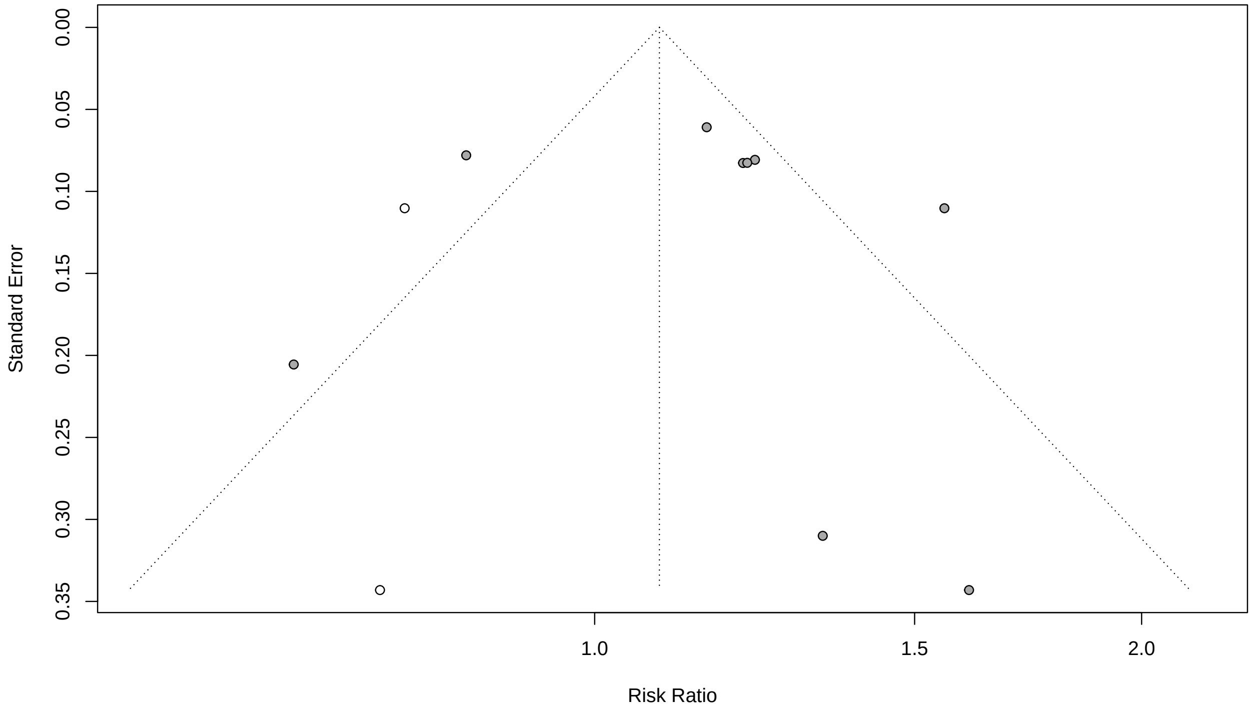

4.1.5 Baujat

```
baujat(immediate_sfr_metabin, pos = 1)
```

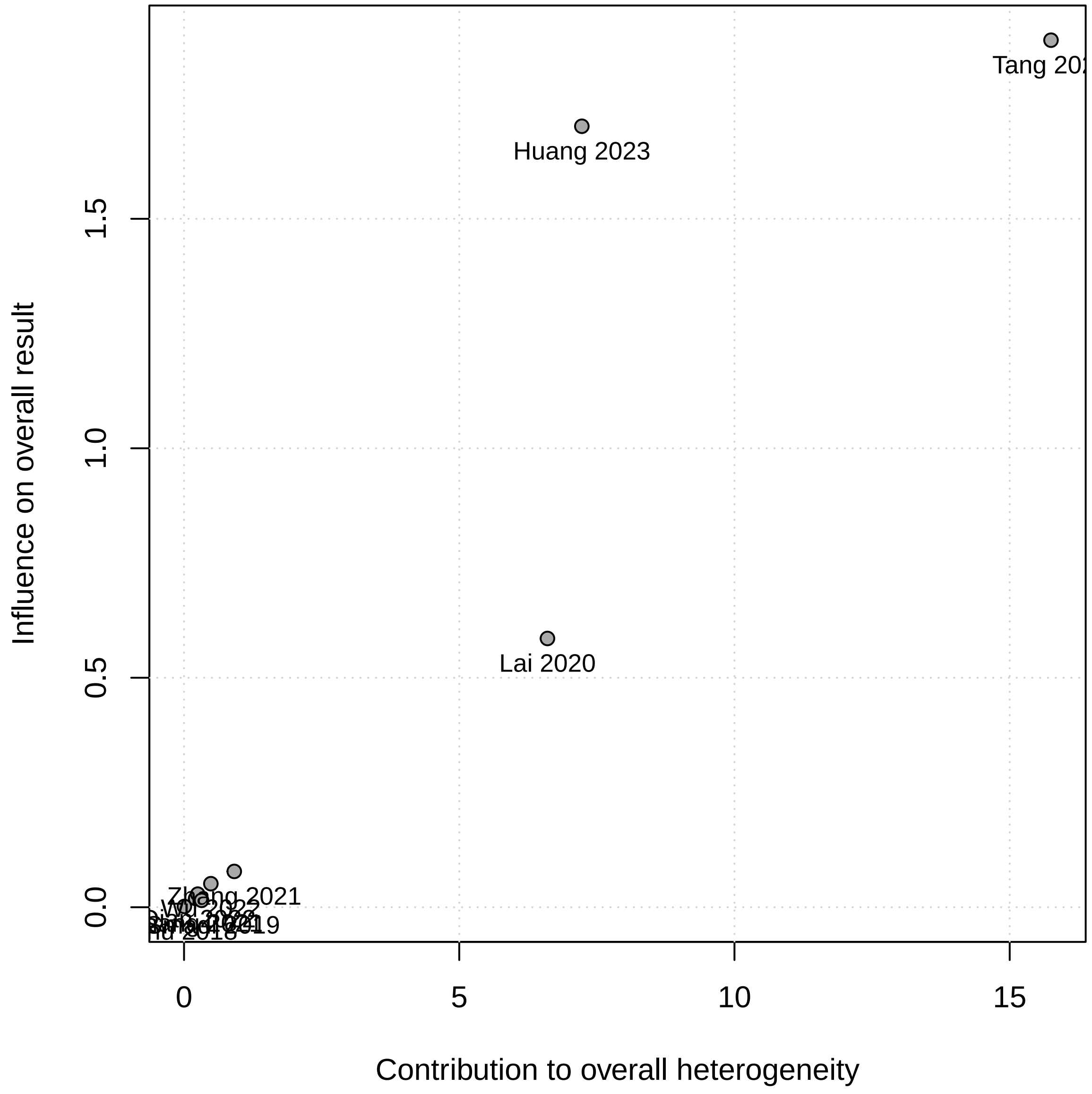

4.1.6 Leave one out

```r
metainf(immediate_sfr_metabin)
```

```
## Influential analysis (common effect model)
##
##                                 RR           95%-CI  p-value   tau^2     tau
## Omitting Sur 2022           1.1581 [1.0834; 1.2380] < 0.0001  0.0365  0.1910
## Omitting Tang 2023          1.2195 [1.1329; 1.3128] < 0.0001  0.0076  0.0873
## Omitting Chen 2019          1.1581 [1.0834; 1.2380] < 0.0001  0.0365  0.1910
## Omitting Du 2019            1.1581 [1.0834; 1.2380] < 0.0001  0.0365  0.1910
## Omitting Lai 2020           1.1889 [1.1116; 1.2716] < 0.0001  0.0258  0.1607
## Omitting Zhu 2018           1.1601 [1.0710; 1.2565]   0.0003  0.0482  0.2196
## Omitting Zhang 2021         1.1473 [1.0741; 1.2254] < 0.0001  0.0376  0.1938
## Omitting Zhang 2021         1.1527 [1.0791; 1.2313] < 0.0001  0.0396  0.1990
## Omitting Lechevallier 2003  1.1581 [1.0834; 1.2380] < 0.0001  0.0365  0.1910
## Omitting Huang 2023         1.1057 [1.0315; 1.1853]   0.0046  0.0246  0.1568
## Omitting Wu 2022            1.1484 [1.0676; 1.2353]   0.0002  0.0464  0.2153
## Omitting Ding 2023          1.1581 [1.0834; 1.2380] < 0.0001  0.0365  0.1910
## Omitting Zhai 2023          1.1581 [1.0834; 1.2380] < 0.0001  0.0365  0.1910
## Omitting Zhai 2023          1.1581 [1.0834; 1.2380] < 0.0001  0.0365  0.1910
## Omitting Qian 2022          1.1508 [1.0700; 1.2377]   0.0002  0.0468  0.2164
## Omitting Zhang 2022         1.1581 [1.0834; 1.2380] < 0.0001  0.0365  0.1910
## Omitting Deng 2022          1.1581 [1.0834; 1.2380] < 0.0001  0.0365  0.1910
## Omitting AlSmadi 2019       1.1529 [1.0734; 1.2383] < 0.0001  0.0467  0.2160
##
## Pooled estimate             1.1581 [1.0834; 1.2380] < 0.0001  0.0365  0.1910
##                                I^2
## Omitting Sur 2022            74.6%
## Omitting Tang 2023           51.5%
## Omitting Chen 2019           74.6%
## Omitting Du 2019             74.6%
## Omitting Lai 2020            72.2%
## Omitting Zhu 2018            77.8%
## Omitting Zhang 2021          77.1%
## Omitting Zhang 2021          77.6%
## Omitting Lechevallier 2003   74.6%
## Omitting Huang 2023          69.4%
## Omitting Wu 2022             77.2%
## Omitting Ding 2023           74.6%
## Omitting Zhai 2023           74.6%
## Omitting Zhai 2023           74.6%
## Omitting Qian 2022           77.4%
## Omitting Zhang 2022          74.6%
## Omitting Deng 2022           74.6%
## Omitting AlSmadi 2019        77.4%
##
## Pooled estimate              74.6%
##
## Details on meta-analytical method:
## - Mantel-Haenszel method
## - Restricted maximum-likelihood estimator for tau^2
```

4.2 Final SFR

4.2.1 Meta-analysis

```
sfr_metabin <- metabin(data = suction_data,
                         event.c = sfr_final_n_control,
                       n.c = sample_size_control,
                       event.e = sfr_final_n_suction,
                       n.e = sample_size_suction,
                       studlab = author_year)
sfr_metabin
```

```
## Number of studies: k = 17
## Number of observations: o = 2311
## Number of events: e = 2014
##
##                           RR           95%-CI    z  p-value
## Common effect model  1.1295 [1.0935; 1.1666] 7.38 < 0.0001
## Random effects model 1.1185 [1.0547; 1.1862] 3.74   0.0002
##
## Quantifying heterogeneity:
##  tau^2 = 0.0102 [0.0036; 0.0310]; tau = 0.1011 [0.0597; 0.1760]
##  I^2 = 71.3% [53.1%; 82.4%]; H = 1.87 [1.46; 2.38]
##
## Test of heterogeneity:
##      Q d.f.  p-value
##  55.71   16 < 0.0001
##
## Details on meta-analytical method:
## - Mantel-Haenszel method
## - Inverse variance method
## - Restricted maximum-likelihood estimator for tau^2
## - Q-Profile method for confidence interval of tau^2 and tau
## - Continuity correction of 0.5 in studies with zero cell frequencies
```

4.2.2 Forest plot

```r
forest(sfr_metabin,
       sortvar = TE)
```

Study	Experimental Events	Experimental Total	Control Events	Control Total	RR	95%−CI	Weight (common)	Weight (random)
Chen 2019	40	44	43	45	0.95	[0.85; 1.06]	4.6%	6.6%
Deng 2022	52	57	67	70	0.95	[0.87; 1.05]	6.5%	7.2%
Lai 2020	25	28	52	56	0.96	[0.83; 1.11]	3.8%	5.7%
Tang 2023	81	86	83	87	0.99	[0.92; 1.06]	8.9%	7.8%
Wu 2022	72	76	76	82	1.02	[0.94; 1.11]	7.9%	7.5%
Qian 2022	72	81	67	81	1.07	[0.95; 1.22]	7.3%	6.3%
Zhu 2018	143	165	131	165	1.09	[0.99; 1.20]	14.2%	7.1%
AlSmadi 2019	41	43	51	60	1.12	[0.99; 1.27]	4.6%	6.3%
Zhang 2022	27	30	23	30	1.17	[0.93; 1.48]	2.5%	3.7%
Zhai 2023	57	60	48	60	1.19	[1.03; 1.36]	5.2%	5.9%
Zhai 2023	58	60	48	60	1.21	[1.06; 1.38]	5.2%	6.0%
Du 2019	62	62	49	60	1.22	[1.09; 1.38]	5.4%	6.5%
Zhang 2021	54	56	42	54	1.24	[1.07; 1.44]	4.6%	5.6%
Huang 2023	97	103	78	103	1.24	[1.10; 1.40]	8.4%	6.4%
Zhang 2021	54	56	37	50	1.30	[1.10; 1.55]	4.2%	5.0%
Sur 2022	9	9	6	8	1.31	[0.90; 1.90]	0.7%	1.9%
Ding 2023	130	138	39	61	1.47	[1.21; 1.79]	5.9%	4.5%
Lechevallier 2003	.	11	.	14			0.0%	0.0%
**Common effect model**		**1165**		**1146**	**1.13**	**[1.09; 1.17]**	**100.0%**	**--**
**Random effects model**					**1.12**	**[1.05; 1.19]**	**--**	**100.0%**

Heterogeneity: $I^2 = 71\%$, $\tau^2 = 0.0102$, $p < 0.01$

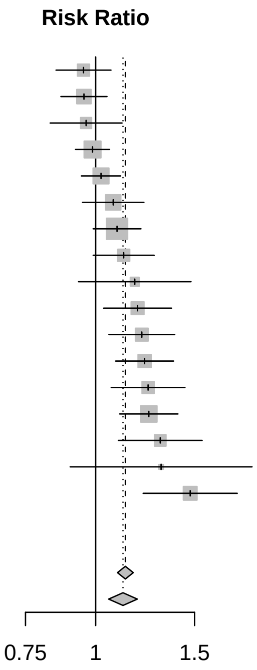

4.2.3 Trim and Fill

```r
trimfill(sfr_metabin)
```

```
## Warning in trimfill.meta(sfr_metabin): 1 observation(s) dropped due to missing
## values
```

```
## Number of studies: k = 23 (with 6 added studies)
## Number of observations: o = 3046
## Number of events: e = 2671
##
##                           RR           95%-CI    z p-value
## Random effects model 1.0468 [0.9768; 1.1219] 1.29  0.1955
##
## Quantifying heterogeneity:
##  tau^2 = 0.0225 [0.0114; 0.0571]; tau = 0.1499 [0.1070; 0.2389]
##  I^2 = 79.5% [70.0%; 86.1%]; H = 2.21 [1.82; 2.68]
##
## Test of heterogeneity:
##       Q d.f.  p-value
##  107.46   22 < 0.0001
##
## Details on meta-analytical method:
## - Inverse variance method
## - Restricted maximum-likelihood estimator for tau^2
## - Q-Profile method for confidence interval of tau^2 and tau
## - Trim-and-fill method to adjust for funnel plot asymmetry (L-estimator)
```

```r
forest(trimfill(sfr_metabin),
       sortvar = TE)
```

```
## Warning in trimfill.meta(sfr_metabin): 1 observation(s) dropped due to missing
## values
```

Study	logRR	SE(logRR)	Experimental Total	Control Total	RR	95%−CI	Weight
Filled: Ding 2023	−0.3184	0.0985	138	61	0.73	[0.60; 0.88]	3.9%
Filled: Sur 2022	−0.1991	0.1903	9	8	0.82	[0.56; 1.19]	2.1%
Filled: Zhang 2021	−0.1956	0.0877	56	50	0.82	[0.69; 0.98]	4.1%
Filled: Huang 2023	−0.1488	0.0609	103	103	0.86	[0.76; 0.97]	4.8%
Filled: Zhang 2021	−0.1458	0.0772	56	54	0.86	[0.74; 1.01]	4.4%
Filled: Du 2019	−0.1315	0.0606	62	60	0.88	[0.78; 0.99]	4.8%
Chen 2019	−0.0498	0.0575	44	45	0.95	[0.85; 1.06]	4.8%
Deng 2022	−0.0480	0.0482	57	70	0.95	[0.87; 1.05]	5.0%
Lai 2020	−0.0392	0.0752	28	56	0.96	[0.83; 1.11]	4.4%
Tang 2023	−0.0128	0.0357	86	87	0.99	[0.92; 1.06]	5.3%
Wu 2022	0.0219	0.0412	76	82	1.02	[0.94; 1.11]	5.2%
Qian 2022	0.0720	0.0642	81	81	1.07	[0.95; 1.22]	4.7%
Zhu 2018	0.0876	0.0501	165	165	1.09	[0.99; 1.20]	5.0%
AlSmadi 2019	0.1149	0.0638	43	60	1.12	[0.99; 1.27]	4.7%
Zhang 2022	0.1603	0.1177	30	30	1.17	[0.93; 1.48]	3.4%
Zhai 2023	0.1719	0.0710	60	60	1.19	[1.03; 1.36]	4.5%
Zhai 2023	0.1892	0.0689	60	60	1.21	[1.06; 1.38]	4.6%
Du 2019	0.2007	0.0606	62	60	1.22	[1.09; 1.38]	4.8%
Zhang 2021	0.2149	0.0772	56	54	1.24	[1.07; 1.44]	4.4%
Huang 2023	0.2180	0.0609	103	103	1.24	[1.10; 1.40]	4.8%
Zhang 2021	0.2647	0.0877	56	50	1.30	[1.10; 1.55]	4.1%
Sur 2022	0.2683	0.1903	9	8	1.31	[0.90; 1.90]	2.1%
Ding 2023	0.3876	0.0985	138	61	1.47	[1.21; 1.79]	3.9%
**Random effects model**			**1578**	**1468**	**1.05**	**[0.98; 1.12]**	**100.0%**

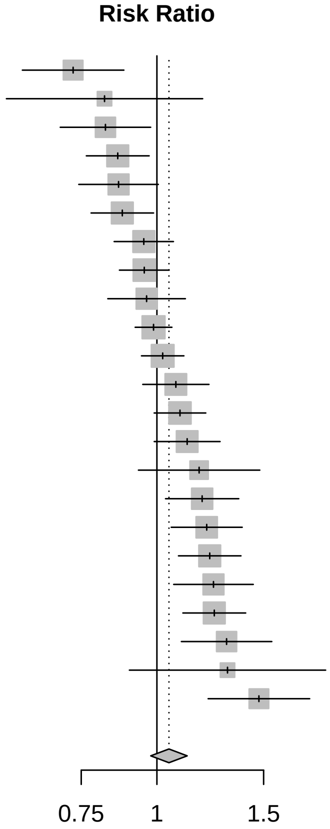

Heterogeneity: $I^2 = 80\%$, $\tau^2 = 0.0225$, $p < 0.01$

4.2.4 Funnel plot

```
funnel(trimfill(sfr_metabin))
```

```
## Warning in trimfill.meta(sfr_metabin): 1 observation(s) dropped due to missing
## values
```

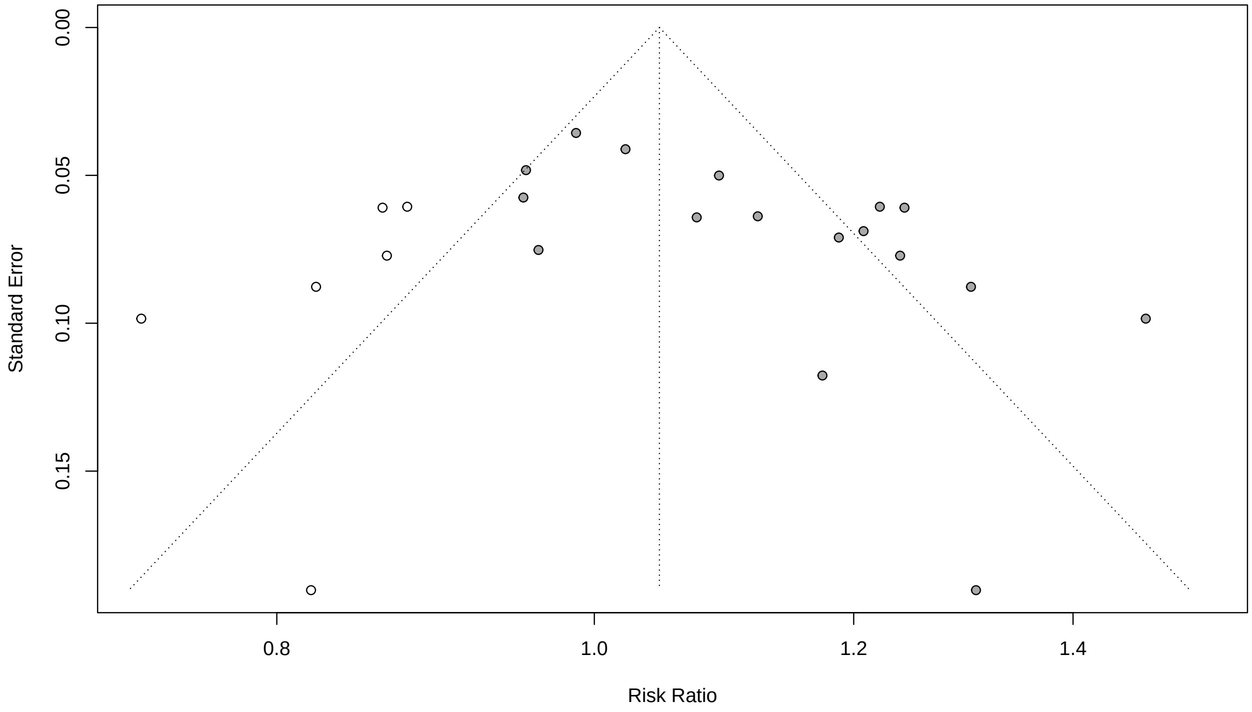

4.2.5 Baujat

```
baujat(sfr_metabin, pos = 1)
```

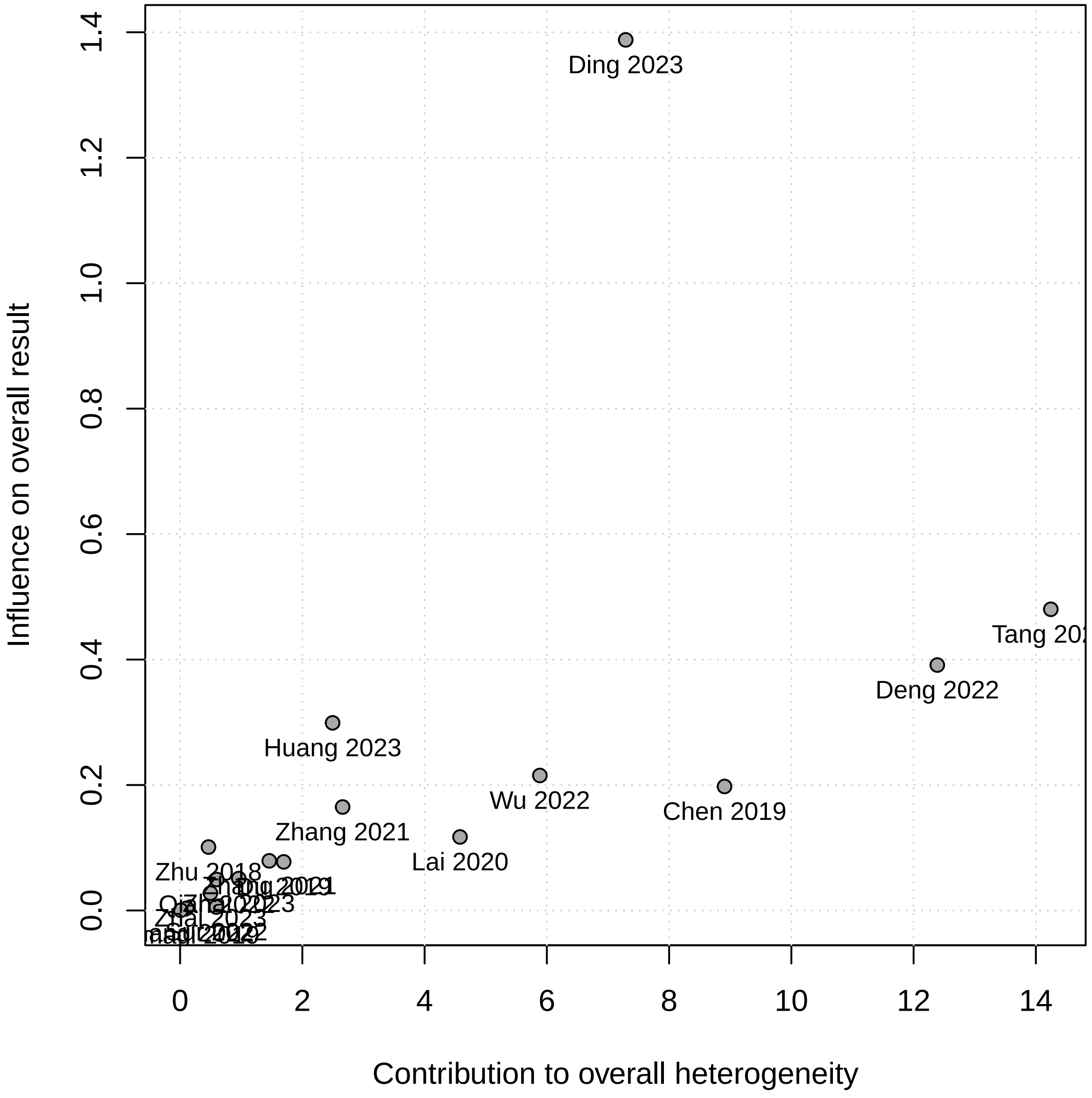

4.2.6 Leave one out

```
metainf(sfr_metabin)
```

```
## Influential analysis (common effect model)
##
##                                 RR           95%-CI  p-value   tau^2     tau
## Omitting Sur 2022           1.1281 [1.0921; 1.1653] < 0.0001  0.0103  0.1015
## Omitting Tang 2023          1.1434 [1.1044; 1.1839] < 0.0001  0.0099  0.0994
## Omitting Chen 2019          1.1381 [1.1007; 1.1768] < 0.0001  0.0094  0.0969
## Omitting Du 2019            1.1241 [1.0871; 1.1624] < 0.0001  0.0105  0.1023
## Omitting Lai 2020           1.1360 [1.0990; 1.1743] < 0.0001  0.0100  0.1002
## Omitting Zhu 2018           1.1357 [1.0977; 1.1751] < 0.0001  0.0114  0.1069
## Omitting Zhang 2021         1.1218 [1.0855; 1.1593] < 0.0001  0.0097  0.0983
## Omitting Zhang 2021         1.1241 [1.0875; 1.1620] < 0.0001  0.0103  0.1016
## Omitting Lechevallier 2003  1.1295 [1.0935; 1.1666] < 0.0001  0.0102  0.1011
## Omitting Huang 2023         1.1190 [1.0820; 1.1571] < 0.0001  0.0101  0.1006
## Omitting Wu 2022            1.1387 [1.1002; 1.1785] < 0.0001  0.0108  0.1038
## Omitting Ding 2023          1.1081 [1.0734; 1.1439] < 0.0001  0.0078  0.0882
## Omitting Zhai 2023          1.1252 [1.0883; 1.1633] < 0.0001  0.0107  0.1033
## Omitting Zhai 2023          1.1263 [1.0895; 1.1644] < 0.0001  0.0109  0.1043
## Omitting Qian 2022          1.1338 [1.0965; 1.1724] < 0.0001  0.0113  0.1061
## Omitting Zhang 2022         1.1284 [1.0921; 1.1658] < 0.0001  0.0108  0.1037
## Omitting Deng 2022          1.1418 [1.1037; 1.1811] < 0.0001  0.0091  0.0955
## Omitting AlSmadi 2019       1.1299 [1.0928; 1.1682] < 0.0001  0.0113  0.1063
##
## Pooled estimate             1.1295 [1.0935; 1.1666] < 0.0001  0.0102  0.1011
##                                I^2
## Omitting Sur 2022            72.6%
## Omitting Tang 2023           68.8%
## Omitting Chen 2019           70.3%
## Omitting Du 2019             70.8%
## Omitting Lai 2020            71.8%
## Omitting Zhu 2018            73.1%
## Omitting Zhang 2021          70.6%
## Omitting Zhang 2021          71.4%
## Omitting Lechevallier 2003   71.3%
## Omitting Huang 2023          70.0%
## Omitting Wu 2022             72.0%
## Omitting Ding 2023           67.1%
## Omitting Zhai 2023           71.7%
## Omitting Zhai 2023           72.2%
## Omitting Qian 2022           73.1%
## Omitting Zhang 2022          72.8%
## Omitting Deng 2022           68.9%
## Omitting AlSmadi 2019        72.9%
##
## Pooled estimate              71.3%
##
## Details on meta-analytical method:
## - Mantel-Haenszel method
## - Restricted maximum-likelihood estimator for tau^2
```

4.3 OR time

4.3.1 Meta-analysis

```
or_time_metacont <- metacont(data = suction_data,
                             mean.c = or_time_min_mean_control,
                             sd.c = or_time_min_sd_control,
                             mean.e = or_time_min_mean_suction,
                             sd.e = or_time_min_sd_suction,
                             n.e = sample_size_suction,
                             n.c = sample_size_control,
                       studlab = author_year)
or_time_metacont
```

```
## Number of studies: k = 17
## Number of observations: o = 2311
##
##                             MD             95%-CI      z  p-value
## Common effect model  -4.3890 [-5.2316; -3.5465] -10.21 < 0.0001
## Random effects model -2.3165 [-7.0736;  2.4406]  -0.95   0.3399
##
## Quantifying heterogeneity:
##  tau^2 = 86.9189 [43.8218; 240.2239]; tau = 9.3230 [6.6198; 15.4992]
##  I^2 = 94.6% [92.7%; 96.0%]; H = 4.32 [3.71; 5.03]
##
## Test of heterogeneity:
##       Q d.f.  p-value
##  298.66   16 < 0.0001
##
## Details on meta-analytical method:
## - Inverse variance method
## - Restricted maximum-likelihood estimator for tau^2
## - Q-Profile method for confidence interval of tau^2 and tau
```

4.3.2 Forest plot

```r
forest(or_time_metacont,
       sortvar = TE)
```

Study	Experimental Total	Experimental Mean	Experimental SD	Control Total	Control Mean	Control SD	MD	95%−CI	Weight (common)	Weight (random)
Du 2019	62	25.30	5.6000	60	47.20	9.8000	−21.90	[−24.74; −19.06]	8.8%	6.6%
Lechevallier 2003	11	37.00	18.0000	14	55.00	18.0000	−18.00	[−32.21; −3.79]	0.4%	4.2%
Zhang 2021	56	46.40	17.9000	54	57.30	28.5000	−10.90	[−19.83; −1.97]	0.9%	5.5%
Wu 2022	76	38.20	6.4000	82	46.70	5.7000	−8.50	[−10.40; −6.60]	19.8%	6.7%
Zhu 2018	165	49.70	16.3000	165	57.00	14.0000	−7.30	[−10.58; −4.02]	6.6%	6.6%
Qian 2022	81	72.90	28.1000	81	80.00	29.5000	−7.10	[−15.97; 1.77]	0.9%	5.5%
Zhang 2022	30	57.30	10.6000	30	62.70	1.2000	−5.40	[−9.22; −1.58]	4.9%	6.5%
Zhai 2023	60	38.00	8.0000	60	43.20	7.9000	−5.20	[−8.04; −2.36]	8.8%	6.6%
AlSmadi 2019	43	38.20	9.2000	60	41.20	9.3000	−3.00	[−6.62; 0.62]	5.4%	6.5%
Zhai 2023	60	41.60	10.6000	60	43.20	7.9000	−1.60	[−4.95; 1.75]	6.3%	6.6%
Tang 2023	86	61.40	5.2000	87	60.30	5.6000	1.10	[−0.51; 2.71]	27.4%	6.7%
Ding 2023	138	42.00	13.2000	61	40.60	12.1000	1.40	[−2.35; 5.15]	5.0%	6.5%
Lai 2020	28	72.40	21.3000	56	67.40	25.0000	5.00	[−5.25; 15.25]	0.7%	5.2%
Zhang 2021	56	46.40	17.9000	50	39.10	13.6000	7.30	[1.28; 13.32]	2.0%	6.1%
Chen 2019	44	65.60	22.5000	45	56.20	28.4000	9.40	[−1.23; 20.03]	0.6%	5.1%
Sur 2022	9	54.00	17.0000	8	39.00	22.0000	15.00	[−3.86; 33.86]	0.2%	3.3%
Deng 2022	57	61.80	21.1000	70	43.40	18.9000	18.40	[11.36; 25.44]	1.4%	5.9%
Huang 2023	103	.	.	103	.	.			0.0%	0.0%
**Common effect model**	**1165**			**1146**			**−4.39**	**[−5.23; −3.55]**	**100.0%**	**--**
**Random effects model**							**−2.32**	**[−7.07; 2.44]**	**--**	**100.0%**

Heterogeneity: $I^2 = 95\%$, $\tau^2 = 86.9189$, $p < 0.01$

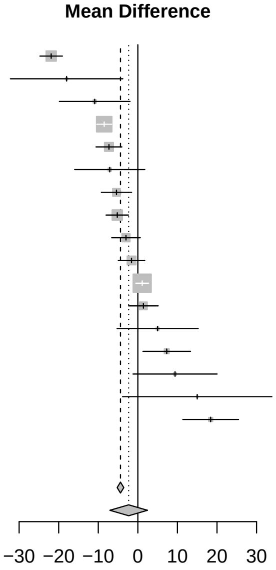

4.3.3 Trim and Fill

```r
trimfill(or_time_metacont)
```

```
## Warning in trimfill.meta(or_time_metacont): 1 observation(s) dropped due to
## missing values

## Number of studies: k = 19 (with 2 added studies)
## Number of observations: o = 2249
##
##                           MD              95%-CI     z p-value
## Random effects model -4.3665 [-9.6885; 0.9556] -1.61  0.1078
##
## Quantifying heterogeneity:
##  tau^2 = 123.4763 [65.6084; 320.2203]; tau = 11.1120 [8.0999; 17.8947]
##  I^2 = 94.8% [93.1%; 96.1%]; H = 4.38 [3.80; 5.05]
##
## Test of heterogeneity:
##       Q d.f.  p-value
##  345.16   18 < 0.0001
##
## Details on meta-analytical method:
## - Inverse variance method
## - Restricted maximum-likelihood estimator for tau^2
## - Q-Profile method for confidence interval of tau^2 and tau
## - Trim-and-fill method to adjust for funnel plot asymmetry (L-estimator)
```

```r
forest(trimfill(or_time_metacont),
       sortvar = TE)
```

```
## Warning in trimfill.meta(or_time_metacont): 1 observation(s) dropped due to
## missing values
```

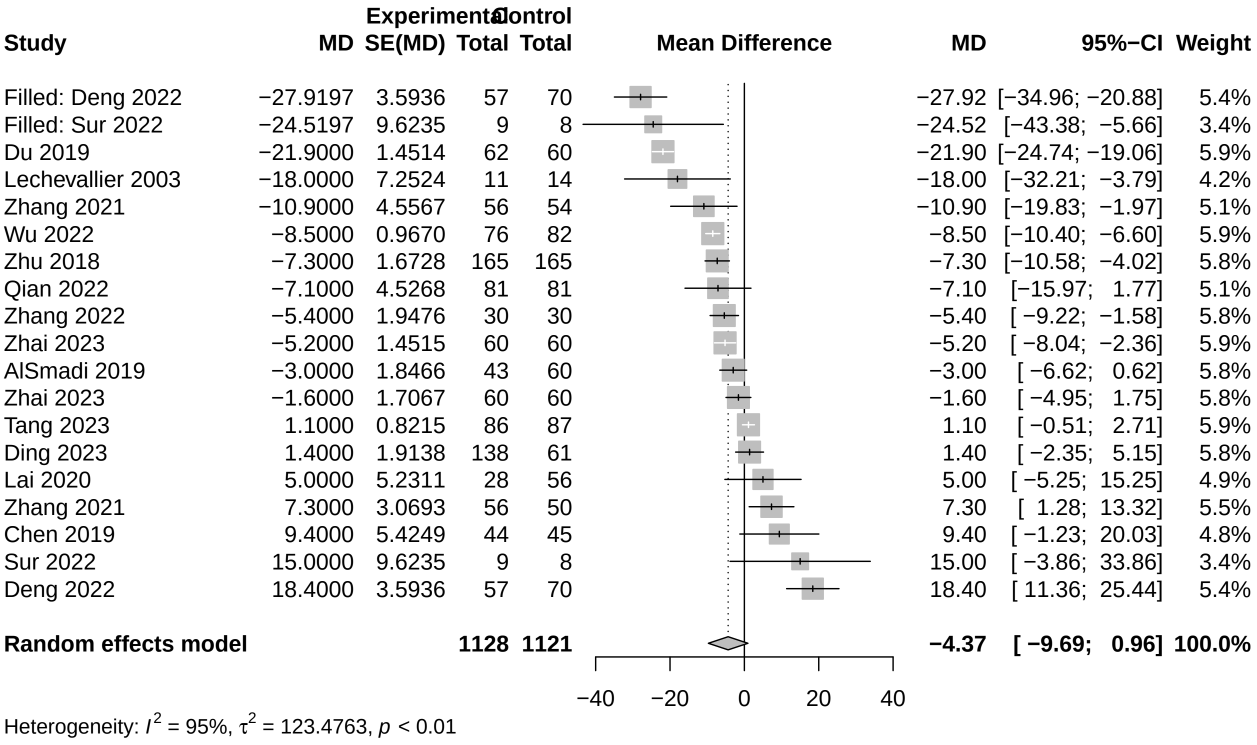

4.3.4 Funnel plot

```
funnel(trimfill(or_time_metacont))
```

```
## Warning in trimfill.meta(or_time_metacont): 1 observation(s) dropped due to
## missing values
```

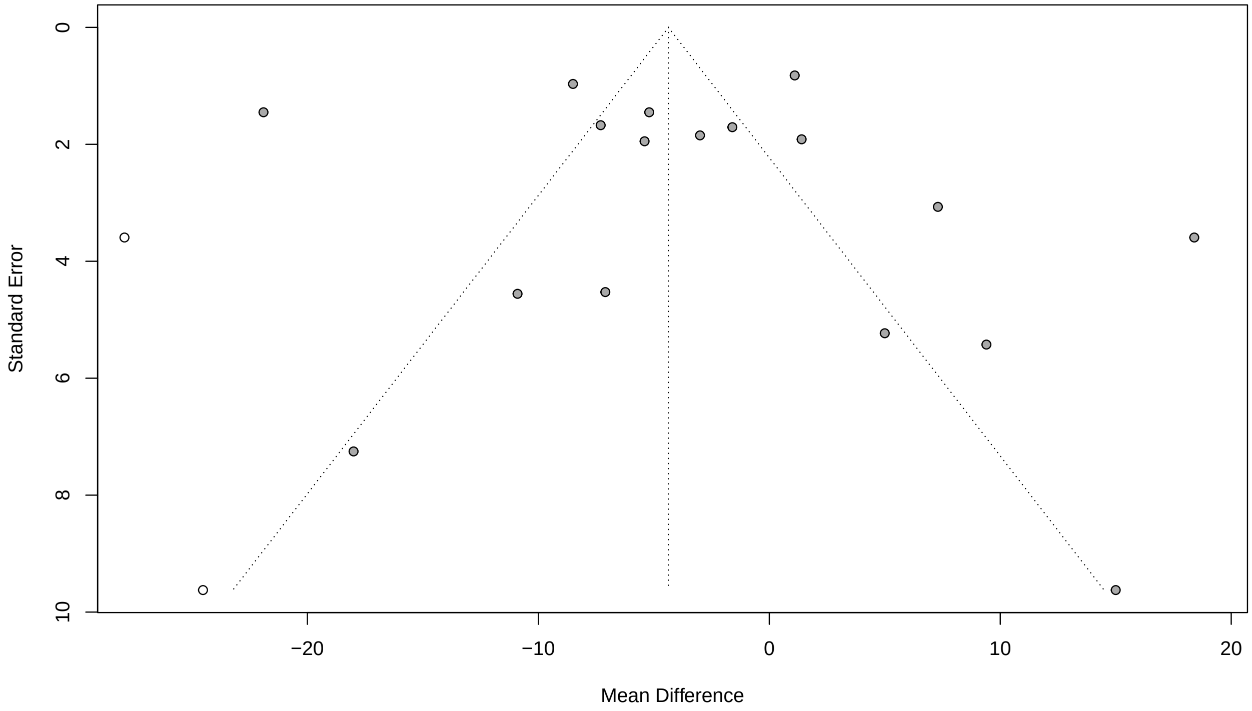

4.3.5 Baujat

```
baujat(or_time_metacont, pos = 1)
```

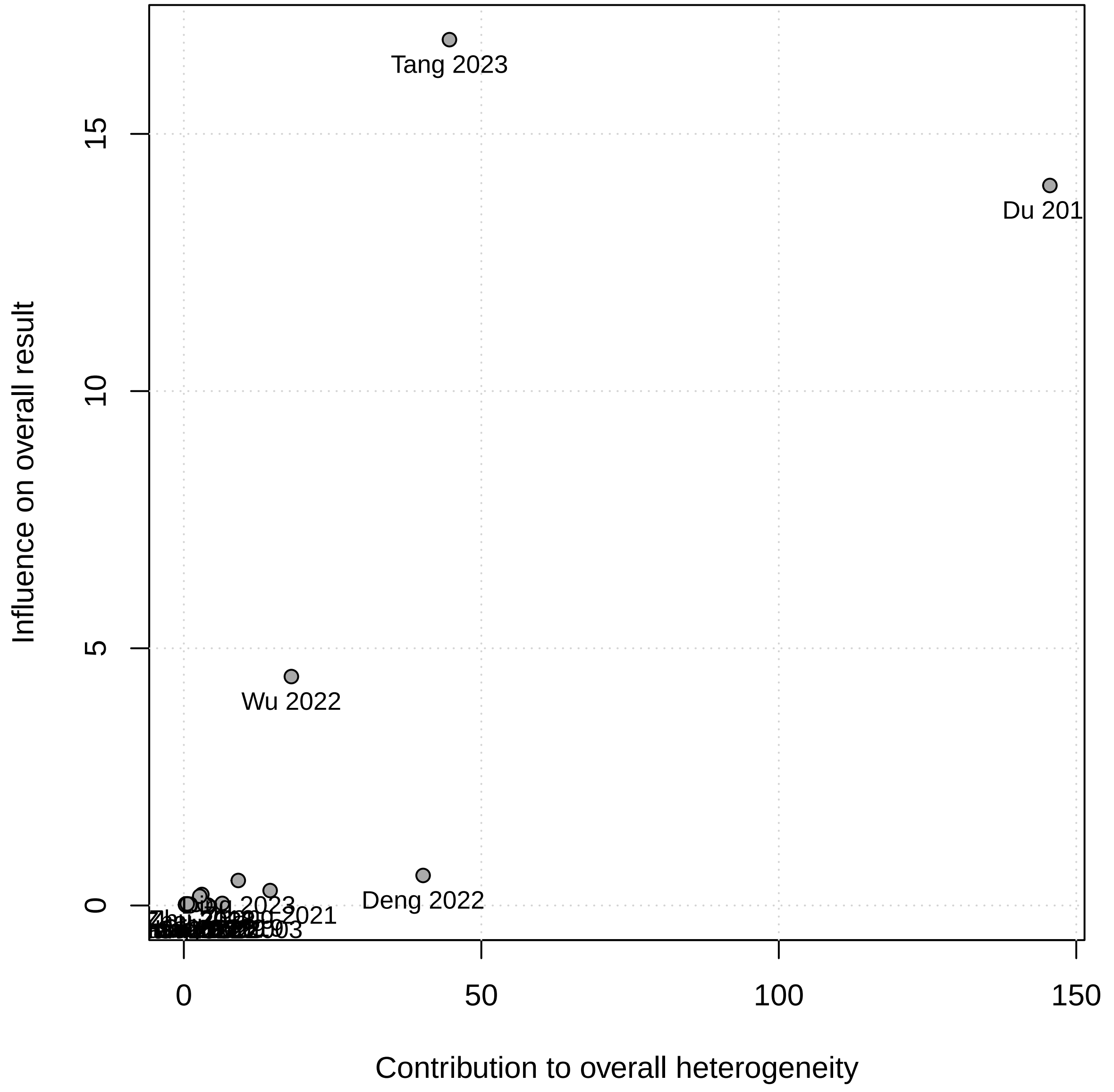

4.3.6 Leave one out

```r
metainf(or_time_metacont)
```

```
## Influential analysis (common effect model)
##
##                                    MD             95%-CI  p-value    tau^2
## Omitting Sur 2022             -4.4278 [-5.2712; -3.5845] < 0.0001  84.0556
## Omitting Tang 2023            -6.4585 [-7.4472; -5.4699] < 0.0001  93.6182
## Omitting Chen 2019            -4.4762 [-5.3213; -3.6310] < 0.0001  85.0733
## Omitting Du 2019              -2.7053 [-3.5874; -1.8232] < 0.0001  56.3894
## Omitting Lai 2020             -4.4529 [-5.2982; -3.6075] < 0.0001  89.7887
## Omitting Zhu 2018             -4.1832 [-5.0550; -3.3114] < 0.0001  92.7198
## Omitting Zhang 2021           -4.6229 [-5.4738; -3.7720] < 0.0001  86.1904
## Omitting Zhang 2021           -4.3306 [-5.1769; -3.4843] < 0.0001  89.2014
## Omitting Lechevallier 2003    -4.3411 [-5.1851; -3.4971] < 0.0001  83.7396
## Omitting Huang 2023           -4.3890 [-5.2316; -3.5465] < 0.0001  86.9189
## Omitting Wu 2022              -3.3768 [-4.3173; -2.4362] < 0.0001  91.5327
## Omitting Ding 2023            -4.6966 [-5.5612; -3.8320] < 0.0001  93.2403
## Omitting Zhai 2023            -4.5780 [-5.4485; -3.7074] < 0.0001  94.6806
## Omitting Zhai 2023            -4.3111 [-5.1932; -3.4290] < 0.0001  94.1958
## Omitting Qian 2022            -4.3644 [-5.2107; -3.5181] < 0.0001  92.2874
## Omitting Zhang 2022           -4.3373 [-5.2011; -3.4735] < 0.0001  93.9887
## Omitting Deng 2022            -4.7199 [-5.5685; -3.8713] < 0.0001  60.3555
## Omitting AlSmadi 2019         -4.4686 [-5.3349; -3.6023] < 0.0001  94.7187
##
## Pooled estimate               -4.3890 [-5.2316; -3.5465] < 0.0001  86.9189
##                                   tau    I^2
## Omitting Sur 2022              9.1682  94.9%
## Omitting Tang 2023             9.6756  93.7%
## Omitting Chen 2019             9.2235  94.9%
## Omitting Du 2019               7.5093  89.2%
## Omitting Lai 2020              9.4757  94.9%
## Omitting Zhu 2018              9.6291  94.9%
## Omitting Zhang 2021            9.2839  94.7%
## Omitting Zhang 2021            9.4446  94.9%
## Omitting Lechevallier 2003     9.1509  94.9%
## Omitting Huang 2023            9.3230  94.6%
## Omitting Wu 2022               9.5673  94.6%
## Omitting Ding 2023             9.6561  94.8%
## Omitting Zhai 2023             9.7304  94.9%
## Omitting Zhai 2023             9.7055  95.0%
## Omitting Qian 2022             9.6066  95.0%
## Omitting Zhang 2022            9.6948  95.0%
## Omitting Deng 2022             7.7689  94.2%
## Omitting AlSmadi 2019          9.7324  95.0%
##
## Pooled estimate                9.3230  94.6%
##
## Details on meta-analytical method:
## - Inverse variance method
## - Restricted maximum-likelihood estimator for tau^2
```

4.4 Auxiliary Treatments

4.4.1 Meta-analysis

```
aux_rx_metabin <- metabin(data = suction_data,
                         event.c = auxillary_tx_n_control,
                       n.c = sample_size_control,
                       event.e = auxillary_tx_n_suction,
                       n.e = sample_size_suction,
                       studlab = author_year)
aux_rx_metabin
```

```
## Number of studies: k = 8
## Number of observations: o = 2311
## Number of events: e = 99
##
##                          RR           95%-CI     z p-value
## Common effect model  0.8130 [0.5619; 1.1762] -1.10  0.2718
## Random effects model 0.8131 [0.3143; 2.1030] -0.43  0.6695
##
## Quantifying heterogeneity:
##  tau^2 = 1.2043 [0.2081; 7.9665]; tau = 1.0974 [0.4562; 2.8225]
##  I^2 = 71.5% [41.2%; 86.2%]; H = 1.87 [1.30; 2.69]
##
## Test of heterogeneity:
##      Q d.f. p-value
##  24.54    7  0.0009
##
## Details on meta-analytical method:
## - Mantel-Haenszel method
## - Inverse variance method
## - Restricted maximum-likelihood estimator for tau^2
## - Q-Profile method for confidence interval of tau^2 and tau
## - Continuity correction of 0.5 in studies with zero cell frequencies
```

4.4.2 Forest plot

```r
forest(aux_rx_metabin,
       sortvar = TE)
```

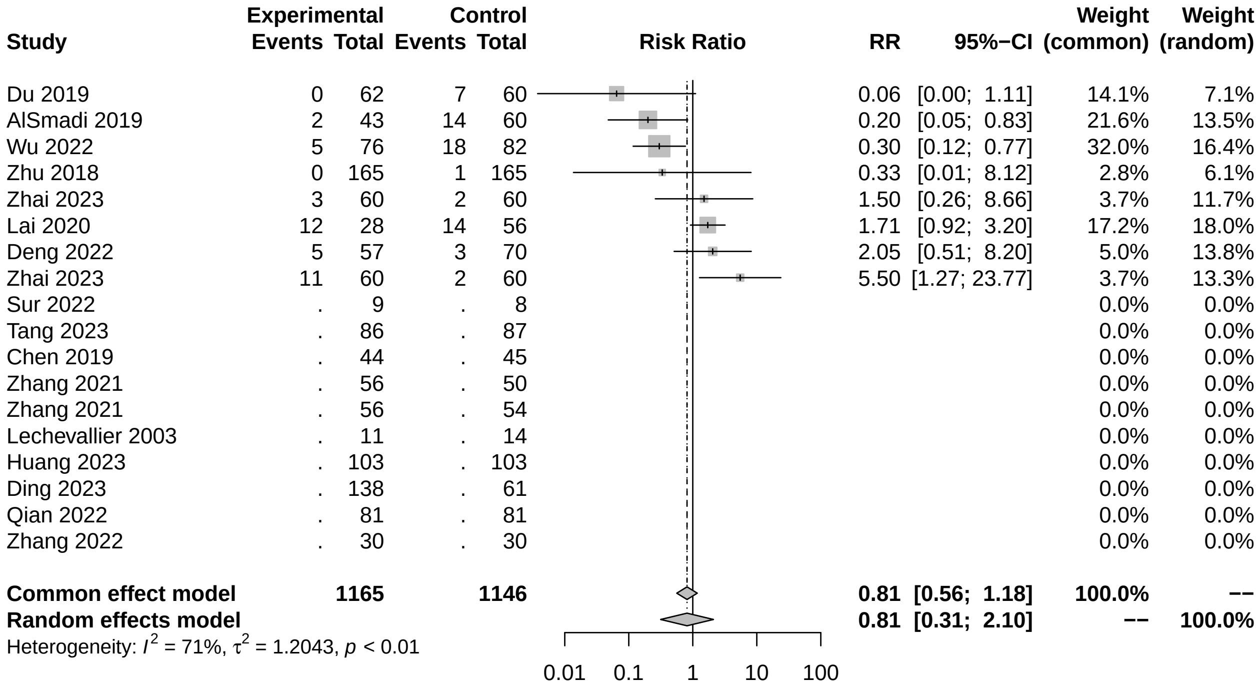

4.4.3 Trim and Fill

```
trimfill(aux_rx_metabin)
```

```
## Warning in trimfill.meta(aux_rx_metabin): 10 observation(s) dropped due to
## missing values

## Number of studies: k = 10 (with 2 added studies)
## Number of observations: o = 1389
## Number of events: e = 122
##
##                             RR           95%-CI    z p-value
## Random effects model 1.2822 [0.4699; 3.4990] 0.49  0.6274
##
## Quantifying heterogeneity:
##  tau^2 = 1.8288 [0.5190; 9.9900]; tau = 1.3523 [0.7204; 3.1607]
##  I^2 = 75.2% [53.8%; 86.7%]; H = 2.01 [1.47; 2.74]
##
## Test of heterogeneity:
##      Q d.f.  p-value
##  36.29    9 < 0.0001
##
## Details on meta-analytical method:
## - Inverse variance method
## - Restricted maximum-likelihood estimator for tau^2
## - Q-Profile method for confidence interval of tau^2 and tau
## - Trim-and-fill method to adjust for funnel plot asymmetry (L-estimator)
```

```
forest(trimfill(aux_rx_metabin),
       sortvar = TE)
```

```
## Warning in trimfill.meta(aux_rx_metabin): 10 observation(s) dropped due to
## missing values
```

Study	logRR	SE(logRR)	Experimental Total	Control Total	RR	95%−CI	Weight
Du 2019	−2.7406	1.4494	62	60	0.06	[0.00; 1.11]	6.7%
AlSmadi 2019	−1.6128	0.7290	43	60	0.20	[0.05; 0.83]	11.1%
Wu 2022	−1.2049	0.4798	76	82	0.30	[0.12; 0.77]	12.7%
Zhu 2018	−1.0986	1.6293	165	165	0.33	[0.01; 8.12]	5.9%
Zhai 2023	0.4055	0.8944	60	60	1.50	[0.26; 8.66]	10.0%
Lai 2020	0.5390	0.3181	28	56	1.71	[0.92; 3.20]	13.6%
Deng 2022	0.7163	0.7082	57	70	2.05	[0.51; 8.20]	11.3%
Zhai 2023	1.7047	0.7467	60	60	5.50	[1.27; 23.77]	11.0%
Filled: AlSmadi 2019	2.0884	0.7290	43	60	8.07	[1.93; 33.69]	11.1%
Filled: Du 2019	3.2162	1.4494	62	60	24.93	[1.46; 427.10]	6.7%
**Random effects model**			**656**	**733**	**1.28**	**[0.47; 3.50]**	**100.0%**

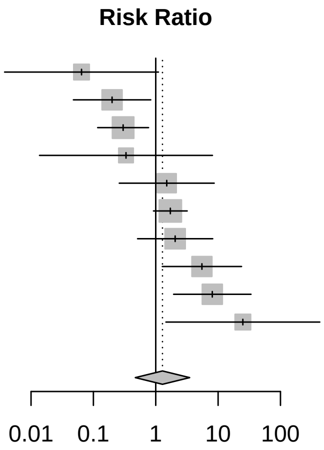

Heterogeneity: $I^2 = 75\%$, $\tau^2 = 1.8288$, $p < 0.01$

4.4.4 Funnel plot

```
funnel(trimfill(aux_rx_metabin))
```

```
## Warning in trimfill.meta(aux_rx_metabin): 10 observation(s) dropped due to
## missing values
```

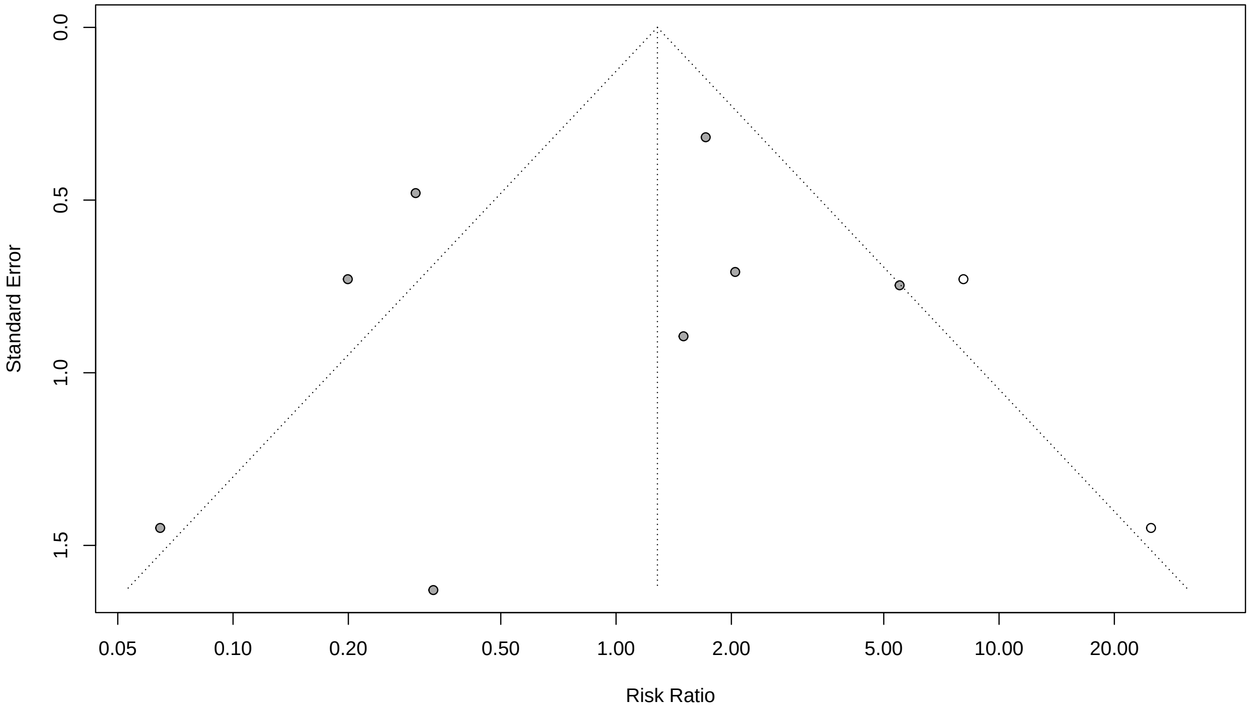

4.4.5 Baujat

```
baujat(aux_rx_metabin, pos = 1)
```

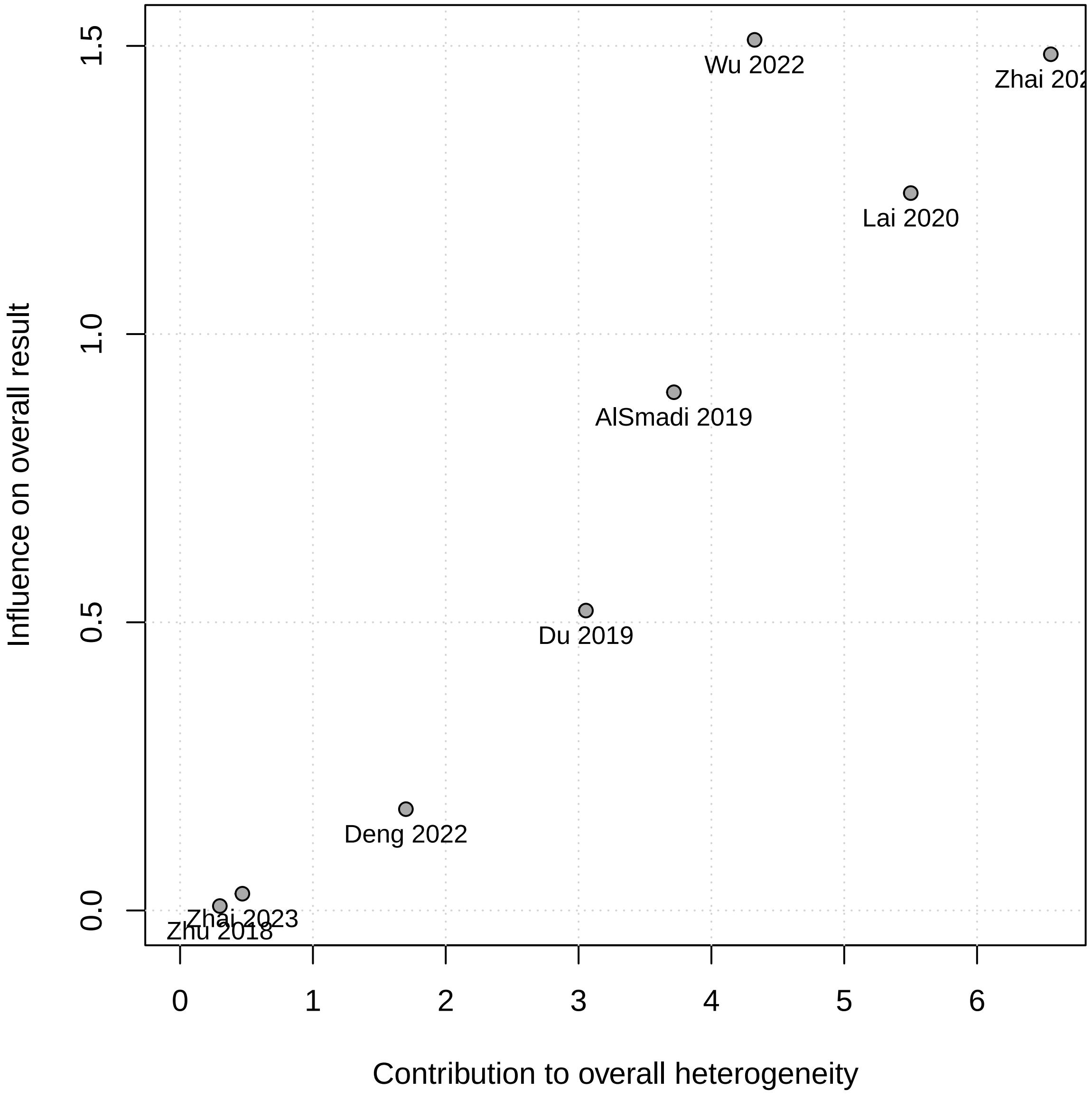

4.4.6 Leave one out

```r
metainf(aux_rx_metabin)
```

```
## Influential analysis (common effect model)
##
##                                  RR            95%-CI p-value   tau^2    tau
## Omitting Sur 2022            0.8130 [0.5619; 1.1762]  0.2718  1.2043  1.0974
## Omitting Tang 2023           0.8130 [0.5619; 1.1762]  0.2718  1.2043  1.0974
## Omitting Chen 2019           0.8130 [0.5619; 1.1762]  0.2718  1.2043  1.0974
## Omitting Du 2019             0.9355 [0.6388; 1.3702]  0.7321  1.0233  1.0116
## Omitting Lai 2020            0.6253 [0.3942; 0.9916]  0.0460  1.4577  1.2074
## Omitting Zhu 2018            0.8266 [0.5697; 1.1994]  0.3161  1.3166  1.1475
## Omitting Zhang 2021          0.8130 [0.5619; 1.1762]  0.2718  1.2043  1.0974
## Omitting Zhang 2021          0.8130 [0.5619; 1.1762]  0.2718  1.2043  1.0974
## Omitting Lechevallier 2003   0.8130 [0.5619; 1.1762]  0.2718  1.2043  1.0974
## Omitting Huang 2023          0.8130 [0.5619; 1.1762]  0.2718  1.2043  1.0974
## Omitting Wu 2022             1.0542 [0.6965; 1.5956]  0.8028  1.1805  1.0865
## Omitting Ding 2023           0.8130 [0.5619; 1.1762]  0.2718  1.2043  1.0974
## Omitting Zhai 2023           0.6332 [0.4237; 0.9464]  0.0258  0.8648  0.9300
## Omitting Zhai 2023           0.7866 [0.5387; 1.1487]  0.2141  1.4761  1.2149
## Omitting Qian 2022           0.8130 [0.5619; 1.1762]  0.2718  1.2043  1.0974
## Omitting Zhang 2022          0.8130 [0.5619; 1.1762]  0.2718  1.2043  1.0974
## Omitting Deng 2022           0.7484 [0.5083; 1.1019]  0.1420  1.4007  1.1835
## Omitting AlSmadi 2019        0.9819 [0.6646; 1.4505]  0.9268  1.0099  1.0049
##
## Pooled estimate              0.8130 [0.5619; 1.1762]  0.2718  1.2043  1.0974
##                                 I^2
## Omitting Sur 2022             71.5%
## Omitting Tang 2023            71.5%
## Omitting Chen 2019            71.5%
## Omitting Du 2019              71.2%
## Omitting Lai 2020             69.4%
## Omitting Zhu 2018             75.1%
## Omitting Zhang 2021           71.5%
## Omitting Zhang 2021           71.5%
## Omitting Lechevallier 2003    71.5%
## Omitting Huang 2023           71.5%
## Omitting Wu 2022              63.6%
## Omitting Ding 2023            71.5%
## Omitting Zhai 2023            68.4%
## Omitting Zhai 2023            75.3%
## Omitting Qian 2022            71.5%
## Omitting Zhang 2022           71.5%
## Omitting Deng 2022            74.4%
## Omitting AlSmadi 2019         68.6%
##
## Pooled estimate               71.5%
##
## Details on meta-analytical method:
## - Mantel-Haenszel method
## - Restricted maximum-likelihood estimator for tau^2
```

4.5 VAS

4.5.1 Meta-analysis

```
vas_metacont <- metacont(data = suction_data,
                              mean.c = vas_score_mean_control,
                              sd.c = vas_score_sd_control,
                              mean.e = vas_score_mean_suction,
                              sd.e = vas_score_sd_suction,
                              n.e = sample_size_suction,
                              n.c = sample_size_control,
                        studlab = author_year)
vas_metacont
```

```
## Number of studies: k = 2
## Number of observations: o = 2311
##
##                              MD             95%-CI     z  p-value
## Common effect model  -0.7490 [-1.0195; -0.4785] -5.43 < 0.0001
## Random effects model -1.2038 [-2.9282;  0.5207] -1.37   0.1713
##
## Quantifying heterogeneity:
##  tau^2 = 1.4954; tau = 1.2229; I^2 = 96.6% [90.7%; 98.7%]; H = 5.39 [3.27; 8.87]
##
## Test of heterogeneity:
##      Q d.f.  p-value
##  29.01    1 < 0.0001
##
## Details on meta-analytical method:
## - Inverse variance method
## - Restricted maximum-likelihood estimator for tau^2
```

4.5.2 Forest plot

```r
forest(vas_metacont,
       sortvar = TE)
```

Study	Experimental Total	Experimental Mean	Experimental SD	Control Total	Control Mean	Control SD	MD	95%−CI	Weight (common)	Weight (random)
Lai 2020	28	3.30	1.3000	56	5.40	1.1000	−2.10	[−2.66; −1.54]	23.2%	49.1%
Zhang 2022	30	0.27	0.4600	30	0.61	0.7300	−0.34	[−0.65; −0.03]	76.8%	50.9%
Sur 2022	9	.	.	8	.	.			0.0%	0.0%
Tang 2023	86	.	.	87	.	.			0.0%	0.0%
Chen 2019	44	.	.	45	.	.			0.0%	0.0%
Du 2019	62	.	.	60	.	.			0.0%	0.0%
Zhu 2018	165	.	.	165	.	.			0.0%	0.0%
Zhang 2021	56	.	.	50	.	.			0.0%	0.0%
Zhang 2021	56	.	.	54	.	.			0.0%	0.0%
Lechevallier 2003	11	.	.	14	.	.			0.0%	0.0%
Huang 2023	103	.	.	103	.	.			0.0%	0.0%
Wu 2022	76	.	.	82	.	.			0.0%	0.0%
Ding 2023	138	.	.	61	.	.			0.0%	0.0%
Zhai 2023	60	.	.	60	.	.			0.0%	0.0%
Zhai 2023	60	.	.	60	.	.			0.0%	0.0%
Qian 2022	81	.	.	81	.	.			0.0%	0.0%
Deng 2022	57	.	.	70	.	.			0.0%	0.0%
AlSmadi 2019	43	.	.	60	.	.			0.0%	0.0%
**Common effect model**	**1165**			**1146**			**−0.75**	**[−1.02; −0.48]**	**100.0%**	**--**
**Random effects model**							**−1.20**	**[−2.93; 0.52]**	**--**	**100.0%**

Heterogeneity: $I^2 = 97\%$, $\tau^2 = 1.4954$, $p < 0.01$

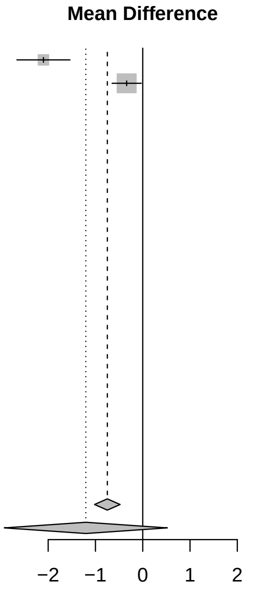

4.5.3 Trim and Fill

```r
#trimfill(vas_metacont)
#forest(trimfill(vas_metacont))
```

4.5.4 Funnel plot

```
funnel((vas_metacont))
```

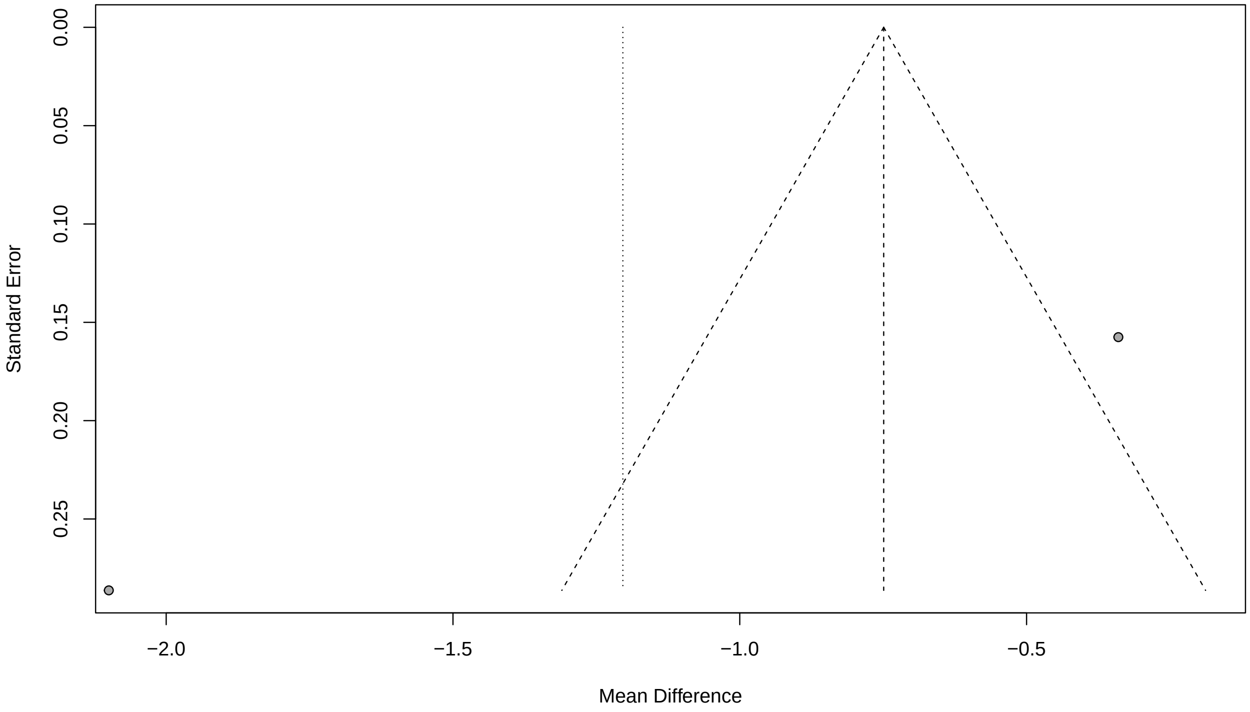

4.5.5 Baujat

```
baujat(vas_metacont, pos = 1)
```

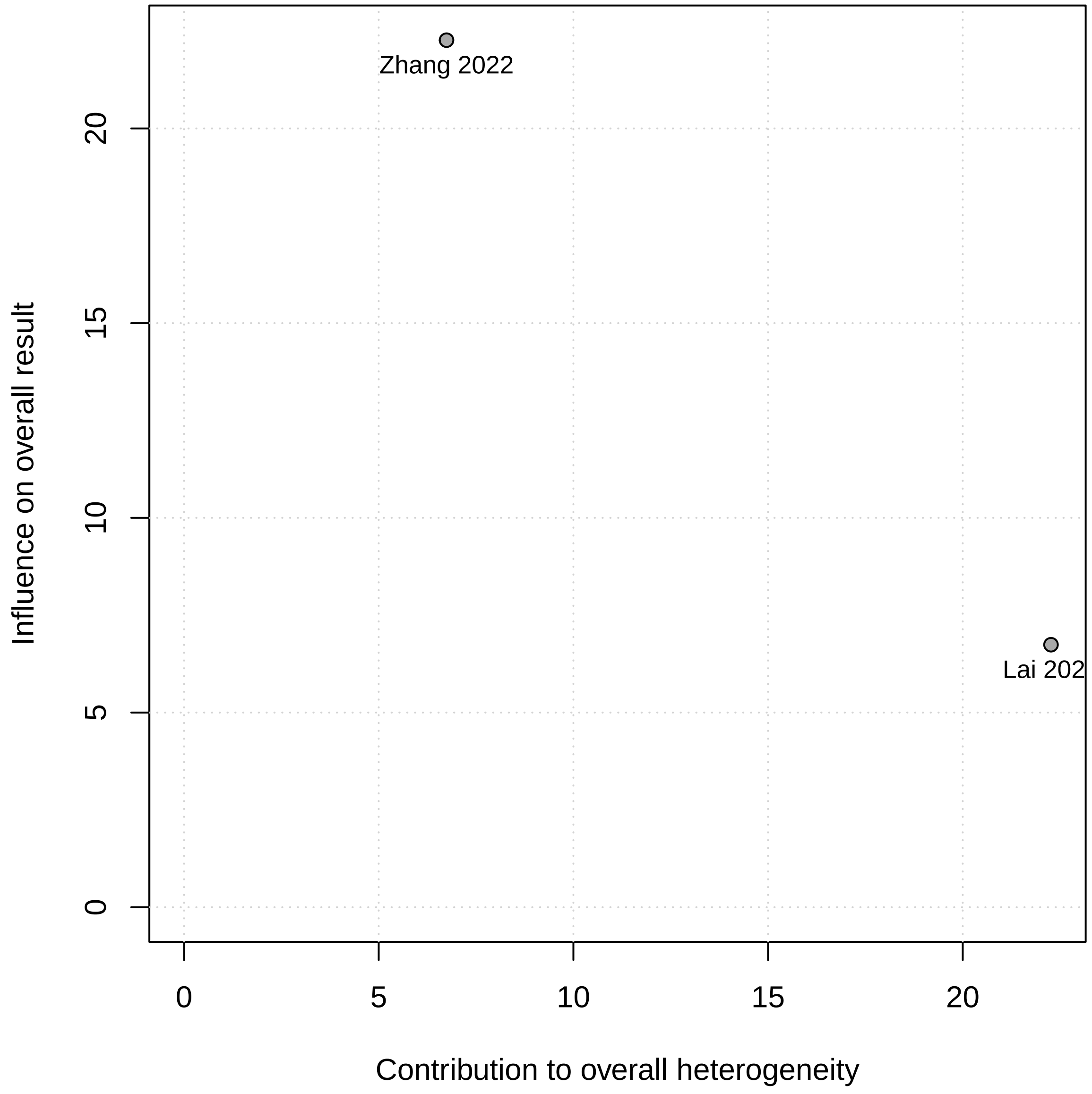

4.5.6 Leave one out

```
metainf(vas_metacont)
```

```
## Influential analysis (common effect model)
##
##                                   MD             95%-CI  p-value   tau^2
## Omitting Sur 2022            -0.7490 [-1.0195; -0.4785] < 0.0001  1.4954
## Omitting Tang 2023           -0.7490 [-1.0195; -0.4785] < 0.0001  1.4954
## Omitting Chen 2019           -0.7490 [-1.0195; -0.4785] < 0.0001  1.4954
## Omitting Du 2019             -0.7490 [-1.0195; -0.4785] < 0.0001  1.4954
## Omitting Lai 2020            -0.3400 [-0.6488; -0.0312]   0.0309
## Omitting Zhu 2018            -0.7490 [-1.0195; -0.4785] < 0.0001  1.4954
## Omitting Zhang 2021          -0.7490 [-1.0195; -0.4785] < 0.0001  1.4954
## Omitting Zhang 2021          -0.7490 [-1.0195; -0.4785] < 0.0001  1.4954
## Omitting Lechevallier 2003   -0.7490 [-1.0195; -0.4785] < 0.0001  1.4954
## Omitting Huang 2023          -0.7490 [-1.0195; -0.4785] < 0.0001  1.4954
## Omitting Wu 2022             -0.7490 [-1.0195; -0.4785] < 0.0001  1.4954
## Omitting Ding 2023           -0.7490 [-1.0195; -0.4785] < 0.0001  1.4954
## Omitting Zhai 2023           -0.7490 [-1.0195; -0.4785] < 0.0001  1.4954
## Omitting Zhai 2023           -0.7490 [-1.0195; -0.4785] < 0.0001  1.4954
## Omitting Qian 2022           -0.7490 [-1.0195; -0.4785] < 0.0001  1.4954
## Omitting Zhang 2022          -2.1000 [-2.6611; -1.5389] < 0.0001
## Omitting Deng 2022           -0.7490 [-1.0195; -0.4785] < 0.0001  1.4954
## Omitting AlSmadi 2019        -0.7490 [-1.0195; -0.4785] < 0.0001  1.4954
##
## Pooled estimate              -0.7490 [-1.0195; -0.4785] < 0.0001  1.4954
##                                 tau    I^2
## Omitting Sur 2022            1.2229  96.6%
## Omitting Tang 2023           1.2229  96.6%
## Omitting Chen 2019           1.2229  96.6%
## Omitting Du 2019             1.2229  96.6%
## Omitting Lai 2020
## Omitting Zhu 2018            1.2229  96.6%
## Omitting Zhang 2021          1.2229  96.6%
## Omitting Zhang 2021          1.2229  96.6%
## Omitting Lechevallier 2003   1.2229  96.6%
## Omitting Huang 2023          1.2229  96.6%
## Omitting Wu 2022             1.2229  96.6%
## Omitting Ding 2023           1.2229  96.6%
## Omitting Zhai 2023           1.2229  96.6%
## Omitting Zhai 2023           1.2229  96.6%
## Omitting Qian 2022           1.2229  96.6%
## Omitting Zhang 2022
## Omitting Deng 2022           1.2229  96.6%
## Omitting AlSmadi 2019        1.2229  96.6%
##
## Pooled estimate              1.2229  96.6%
##
## Details on meta-analytical method:
## - Inverse variance method
## - Restricted maximum-likelihood estimator for tau^2
```

4.6 LoS

4.6.1 Meta-analysis

```
los_metacont <- metacont(data = suction_data,
                             mean.c = los_days_mean_control,
                             sd.c = los_days_sd_control,
                             mean.e = los_days_mean_suction,
                             sd.e = los_days_sd_suction,
                             n.e = sample_size_suction,
                             n.c = sample_size_control,
                       studlab = author_year)
los_metacont
```

```
## Number of studies: k = 9
## Number of observations: o = 2311
##
##                            MD             95%-CI     z  p-value
## Common effect model  -0.3668 [-0.4500; -0.2837] -8.65 < 0.0001
## Random effects model -1.0877 [-1.9003; -0.2752] -2.62   0.0087
##
## Quantifying heterogeneity:
##  tau^2 = 1.4367 [0.5938; 5.7266]; tau = 1.1986 [0.7706; 2.3930]
##  I^2 = 92.6% [88.1%; 95.4%]; H = 3.67 [2.90; 4.65]
##
## Test of heterogeneity:
##       Q d.f.  p-value
##  107.98    8 < 0.0001
##
## Details on meta-analytical method:
## - Inverse variance method
## - Restricted maximum-likelihood estimator for tau^2
## - Q-Profile method for confidence interval of tau^2 and tau
```

4.6.2 Forest plot

```r
forest(los_metacont,
       sortvar = TE)
```

Study	Experimental Total	Experimental Mean	Experimental SD	Control Total	Control Mean	Control SD	MD	95%−CI	Weight (common)	Weight (random)
Deng 2022	57	3.60	1.5000	70	6.90	3.1000	−3.30	[−4.12; −2.48]	1.0%	10.7%
Chen 2019	44	3.50	1.3000	45	6.50	2.4000	−3.00	[−3.80; −2.20]	1.1%	10.7%
Tang 2023	86	3.70	.	87	5.90	.	−2.20		0.0%	0.0%
Lai 2020	28	4.30	2.9000	56	6.10	3.2000	−1.80	[−3.16; −0.44]	0.4%	9.0%
Zhang 2022	30	2.30	0.9000	30	3.20	1.4000	−0.90	[−1.50; −0.30]	1.9%	11.2%
Zhai 2023	60	5.30	1.1000	60	6.00	1.1000	−0.70	[−1.09; −0.31]	4.5%	11.6%
AlSmadi 2019	43	1.70	0.2000	60	2.00	0.3000	−0.30	[−0.40; −0.20]	74.0%	11.9%
Zhu 2018	165	1.10	1.2000	165	1.30	1.2000	−0.20	[−0.46; 0.06]	10.3%	11.8%
Du 2019	62	4.40	1.2000	60	4.50	1.6000	−0.10	[−0.60; 0.40]	2.7%	11.4%
Zhai 2023	60	6.00	1.2000	60	6.00	1.1000	0.00	[−0.41; 0.41]	4.1%	11.6%
Sur 2022	9	.	.	8	.	.			0.0%	0.0%
Zhang 2021	56	.	.	50	.	.			0.0%	0.0%
Zhang 2021	56	.	.	54	.	.			0.0%	0.0%
Lechevallier 2003	11	.	.	14	.	.			0.0%	0.0%
Huang 2023	103	.	.	103	.	.			0.0%	0.0%
Wu 2022	76	.	.	82	.	.			0.0%	0.0%
Ding 2023	138	.	.	61	.	.			0.0%	0.0%
Qian 2022	81	.	.	81	.	.			0.0%	0.0%
**Common effect model**	**1165**			**1146**			**−0.37**	**[−0.45; −0.28]**	**100.0%**	**−−**
**Random effects model**							**−1.09**	**[−1.90; −0.28]**	**−−**	**100.0%**

Heterogeneity: $I^2 = 93\%$, $\tau^2 = 1.4367$, $p < 0.01$

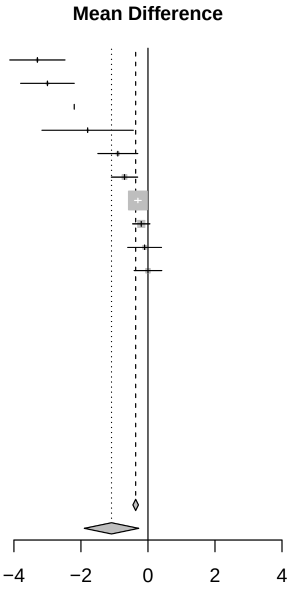

4.6.3 Trim and Fill

```r
trimfill(los_metacont)
```

```
## Warning in trimfill.meta(los_metacont): 9 observation(s) dropped due to missing
## values

## Number of studies: k = 12 (with 3 added studies)
## Number of observations: o = 1455
##
##                          MD             95%-CI     z p-value
## Random effects model -0.3345 [-1.3661; 0.6970] -0.64  0.5250
##
## Quantifying heterogeneity:
##  tau^2 = 3.1716 [1.5169; 9.6686]; tau = 1.7809 [1.2316; 3.1094]
##  I^2 = 94.8% [92.5%; 96.4%]; H = 4.37 [3.64; 5.24]
##
## Test of heterogeneity:
##       Q d.f.  p-value
##  209.61   11 < 0.0001
##
## Details on meta-analytical method:
## - Inverse variance method
## - Restricted maximum-likelihood estimator for tau^2
## - Q-Profile method for confidence interval of tau^2 and tau
## - Trim-and-fill method to adjust for funnel plot asymmetry (L-estimator)
```

```r
forest(trimfill(los_metacont),
       sortvar = TE)
```

```
## Warning in trimfill.meta(los_metacont): 9 observation(s) dropped due to missing
## values
```

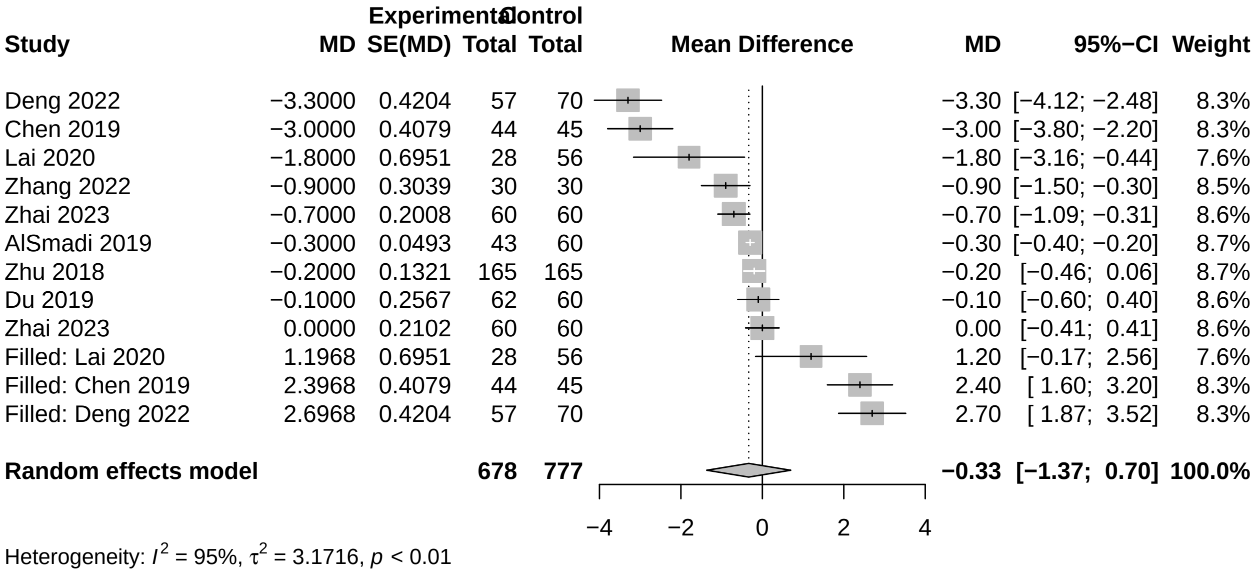

4.6.4 Funnel plot

```
funnel(trimfill(los_metacont))
```

```
## Warning in trimfill.meta(los_metacont): 9 observation(s) dropped due to missing
## values
```

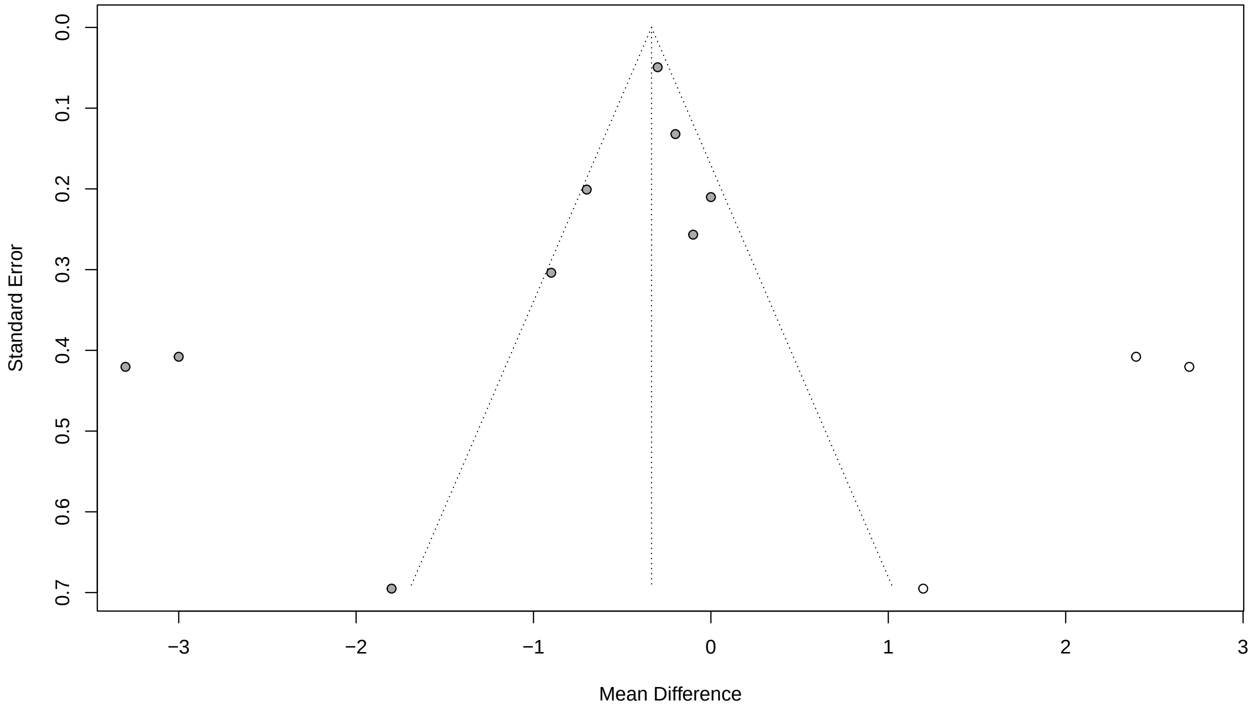

4.6.5 Baujat

```r
baujat(los_metacont, pos = 1)
```

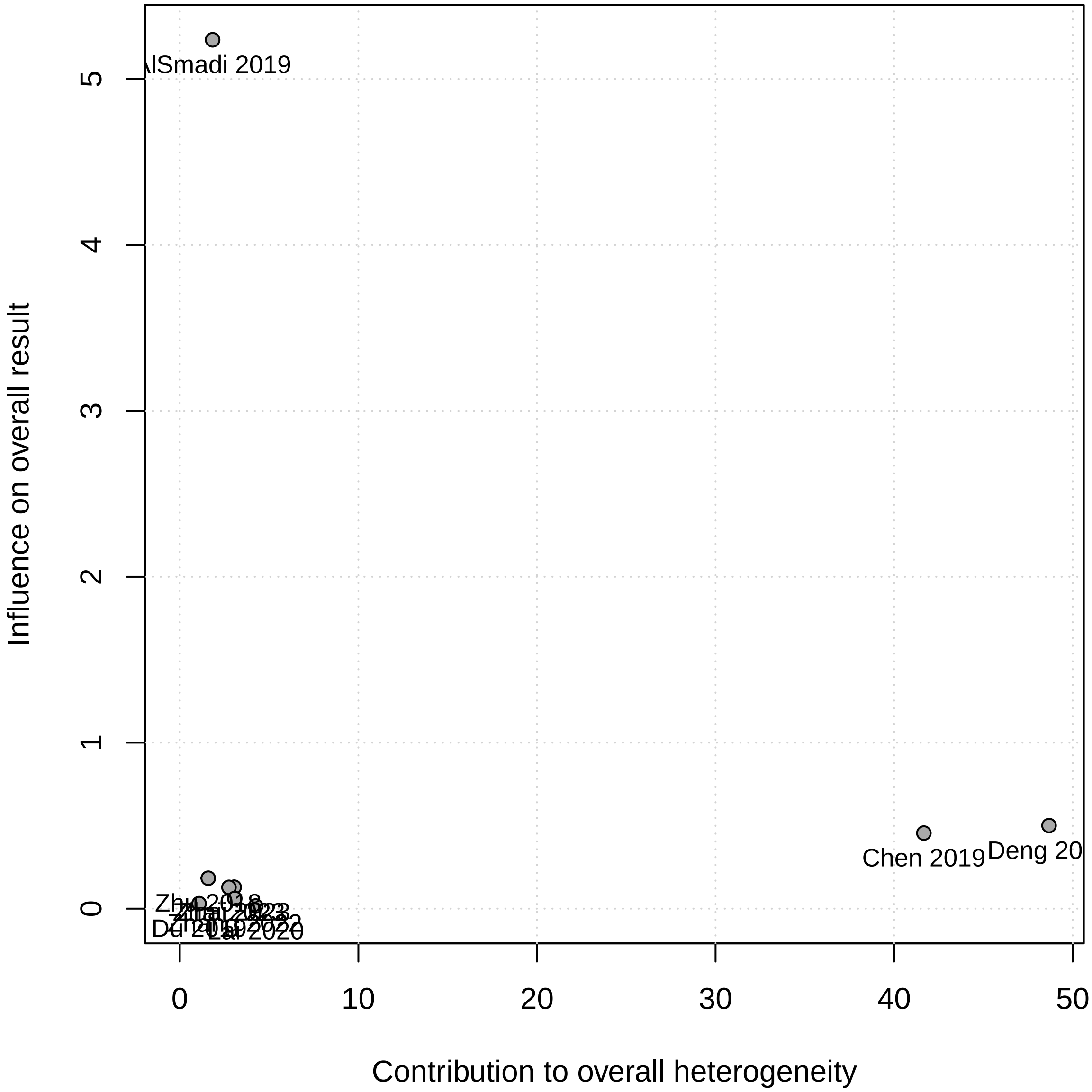

4.6.6 Leave one out

```r
metainf(los_metacont)
```

```
## Influential analysis (common effect model)
##
##                                  MD             95%-CI  p-value   tau^2
## Omitting Sur 2022            -0.3668 [-0.4500; -0.2837] < 0.0001  1.4367
## Omitting Tang 2023           -0.3668 [-0.4500; -0.2837] < 0.0001  1.4367
## Omitting Chen 2019           -0.3381 [-0.4216; -0.2545] < 0.0001  1.0516
## Omitting Du 2019             -0.3743 [-0.4586; -0.2900] < 0.0001  1.5161
## Omitting Lai 2020            -0.3615 [-0.4448; -0.2782] < 0.0001  1.5628
## Omitting Zhu 2018            -0.3860 [-0.4738; -0.2982] < 0.0001  1.5445
## Omitting Zhang 2021          -0.3668 [-0.4500; -0.2837] < 0.0001  1.4367
## Omitting Zhang 2021          -0.3668 [-0.4500; -0.2837] < 0.0001  1.4367
## Omitting Lechevallier 2003   -0.3668 [-0.4500; -0.2837] < 0.0001  1.4367
## Omitting Huang 2023          -0.3668 [-0.4500; -0.2837] < 0.0001  1.4367
## Omitting Wu 2022             -0.3668 [-0.4500; -0.2837] < 0.0001  1.4367
## Omitting Ding 2023           -0.3668 [-0.4500; -0.2837] < 0.0001  1.4367
## Omitting Zhai 2023           -0.3824 [-0.4673; -0.2975] < 0.0001  1.4780
## Omitting Zhai 2023           -0.3513 [-0.4363; -0.2662] < 0.0001  1.6539
## Omitting Qian 2022           -0.3668 [-0.4500; -0.2837] < 0.0001  1.4367
## Omitting Zhang 2022          -0.3562 [-0.4402; -0.2723] < 0.0001  1.6652
## Omitting Deng 2022           -0.3367 [-0.4202; -0.2531] < 0.0001  0.8633
## Omitting AlSmadi 2019        -0.5572 [-0.7203; -0.3942] < 0.0001  1.5741
##
## Pooled estimate              -0.3668 [-0.4500; -0.2837] < 0.0001  1.4367
##                                 tau    I^2
## Omitting Sur 2022            1.1986  92.6%
## Omitting Tang 2023           1.1986  92.6%
## Omitting Chen 2019           1.0255  89.4%
## Omitting Du 2019             1.2313  93.4%
## Omitting Lai 2020            1.2501  93.3%
## Omitting Zhu 2018            1.2428  93.4%
## Omitting Zhang 2021          1.1986  92.6%
## Omitting Zhang 2021          1.1986  92.6%
## Omitting Lechevallier 2003   1.1986  92.6%
## Omitting Huang 2023          1.1986  92.6%
## Omitting Wu 2022             1.1986  92.6%
## Omitting Ding 2023           1.1986  92.6%
## Omitting Zhai 2023           1.2157  93.3%
## Omitting Zhai 2023           1.2860  93.3%
## Omitting Qian 2022           1.1986  92.6%
## Omitting Zhang 2022          1.2904  93.3%
## Omitting Deng 2022           0.9292  88.1%
## Omitting AlSmadi 2019        1.2546  93.1%
##
## Pooled estimate              1.1986  92.6%
##
## Details on meta-analytical method:
## - Inverse variance method
## - Restricted maximum-likelihood estimator for tau^2
```

4.7 Complications - All

4.7.1 Meta-analysis

```
comp_metabin <- metabin(data = suction_data,
                          event.c = complications_postop_overall_n_control,
                        n.c = sample_size_control,
                        event.e = complications_postop_overall_n_suction,
                        n.e = sample_size_suction,
                        studlab = author_year)
comp_metabin
```

```
## Number of studies: k = 17
## Number of observations: o = 2311
## Number of events: e = 247
##
##                          RR           95%-CI     z  p-value
## Common effect model  0.4012 [0.3092; 0.5205] -6.88 < 0.0001
## Random effects model 0.4353 [0.3303; 0.5736] -5.91 < 0.0001
##
## Quantifying heterogeneity:
##  tau^2 = 0.0095 [0.0000; 0.4976]; tau = 0.0975 [0.0000; 0.7054]
##  I^2 = 0.0% [0.0%; 51.1%]; H = 1.00 [1.00; 1.43]
##
## Test of heterogeneity:
##      Q d.f. p-value
##  15.15   16  0.5137
##
## Details on meta-analytical method:
## - Mantel-Haenszel method
## - Inverse variance method
## - Restricted maximum-likelihood estimator for tau^2
## - Q-Profile method for confidence interval of tau^2 and tau
## - Continuity correction of 0.5 in studies with zero cell frequencies
```

4.7.2 Forest plot

```r
forest(comp_metabin,
       sortvar = TE)
```

Study	Experimental Events	Experimental Total	Control Events	Control Total	RR	95%−CI	Weight (common)	Weight (random)
Ding 2023	0	138	3	61	0.06	[0.00; 1.21]	2.8%	0.9%
Du 2019	1	62	10	60	0.10	[0.01; 0.73]	5.8%	1.8%
Tang 2023	5	86	29	87	0.17	[0.07; 0.43]	16.5%	9.0%
Qian 2022	3	81	12	81	0.25	[0.07; 0.85]	6.9%	4.9%
Wu 2022	3	76	12	82	0.27	[0.08; 0.92]	6.6%	4.9%
Zhang 2022	1	30	3	30	0.33	[0.04; 3.03]	1.7%	1.6%
Chen 2019	5	44	14	45	0.37	[0.14; 0.93]	7.9%	8.4%
Zhang 2021	2	56	4	50	0.45	[0.09; 2.33]	2.4%	2.7%
Zhu 2018	19	165	41	165	0.46	[0.28; 0.76]	23.4%	26.7%
AlSmadi 2019	1	43	3	60	0.47	[0.05; 4.32]	1.4%	1.5%
Deng 2022	7	57	18	70	0.48	[0.21; 1.06]	9.2%	11.3%
Zhang 2021	2	56	3	54	0.64	[0.11; 3.70]	1.7%	2.5%
Huang 2023	3	103	4	103	0.75	[0.17; 3.27]	2.3%	3.5%
Zhai 2023	4	60	5	60	0.80	[0.23; 2.83]	2.9%	4.7%
Zhai 2023	4	60	5	60	0.80	[0.23; 2.83]	2.9%	4.7%
Sur 2022	1	9	1	8	0.89	[0.07; 12.00]	0.6%	1.1%
Lai 2020	6	28	13	56	0.92	[0.39; 2.17]	5.0%	9.9%
Lechevallier 2003	.	11	.	14			0.0%	0.0%
**Common effect model**		**1165**		**1146**	**0.40**	**[0.31; 0.52]**	**100.0%**	**--**
**Random effects model**					**0.44**	**[0.33; 0.57]**	**--**	**100.0%**

Heterogeneity: $I^2 = 0\%$, $\tau^2 = 0.0095$, $p = 0.51$

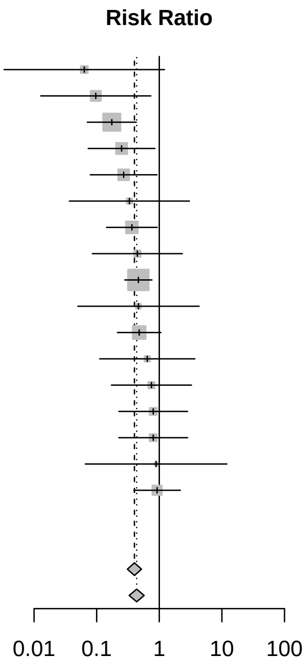

4.7.3 Trim and Fill

```r
trimfill(comp_metabin)
```

```
## Warning in trimfill.meta(comp_metabin): 1 observation(s) dropped due to missing
## values
```

```
## Number of studies: k = 17 (with 0 added studies)
## Number of observations: o = 2286
## Number of events: e = 247
## 
##                          RR           95%-CI     z  p-value
## Random effects model 0.4353 [0.3303; 0.5736] -5.91 < 0.0001
## 
## Quantifying heterogeneity:
##  tau^2 = 0.0095 [0.0000; 0.4976]; tau = 0.0975 [0.0000; 0.7054]
##  I^2 = 0.0% [0.0%; 51.1%]; H = 1.00 [1.00; 1.43]
## 
## Test of heterogeneity:
##      Q d.f. p-value
##  15.15   16  0.5137
## 
## Details on meta-analytical method:
## - Inverse variance method
## - Restricted maximum-likelihood estimator for tau^2
## - Q-Profile method for confidence interval of tau^2 and tau
## - Trim-and-fill method to adjust for funnel plot asymmetry (L-estimator)
```

```r
forest(trimfill(comp_metabin),
       sortvar = TE)
```

```
## Warning in trimfill.meta(comp_metabin): 1 observation(s) dropped due to missing
## values
```

Study	logRR	SE(logRR)	Experimental Total	Control Total	RR	95%−CI	Weight
Ding 2023	−2.7577	1.5041	138	61	0.06	[0.00; 1.21]	0.9%
Du 2019	−2.3354	1.0331	62	60	0.10	[0.01; 0.73]	1.8%
Tang 2023	−1.7463	0.4597	86	87	0.17	[0.07; 0.43]	9.0%
Qian 2022	−1.3863	0.6261	81	81	0.25	[0.07; 0.85]	4.9%
Wu 2022	−1.3103	0.6256	76	82	0.27	[0.08; 0.92]	4.9%
Zhang 2022	−1.0986	1.1255	30	30	0.33	[0.04; 3.03]	1.6%
Chen 2019	−1.0071	0.4759	44	45	0.37	[0.14; 0.93]	8.4%
Zhang 2021	−0.8065	0.8439	56	50	0.45	[0.09; 2.33]	2.7%
Zhu 2018	−0.7691	0.2548	165	165	0.46	[0.28; 0.76]	26.7%
AlSmadi 2019	−0.7655	1.1373	43	60	0.47	[0.05; 4.32]	1.5%
Deng 2022	−0.7390	0.4081	57	70	0.48	[0.21; 1.06]	11.3%
Zhang 2021	−0.4418	0.8927	56	54	0.64	[0.11; 3.70]	2.5%
Huang 2023	−0.2877	0.7509	103	103	0.75	[0.17; 3.27]	3.5%
Zhai 2023	−0.2231	0.6455	60	60	0.80	[0.23; 2.83]	4.7%
Zhai 2023	−0.2231	0.6455	60	60	0.80	[0.23; 2.83]	4.7%
Sur 2022	−0.1178	1.3281	9	8	0.89	[0.07; 12.00]	1.1%
Lai 2020	−0.0800	0.4359	28	56	0.92	[0.39; 2.17]	9.9%
**Random effects model**			**1154**	**1132**	**0.44**	**[0.33; 0.57]**	**100.0%**

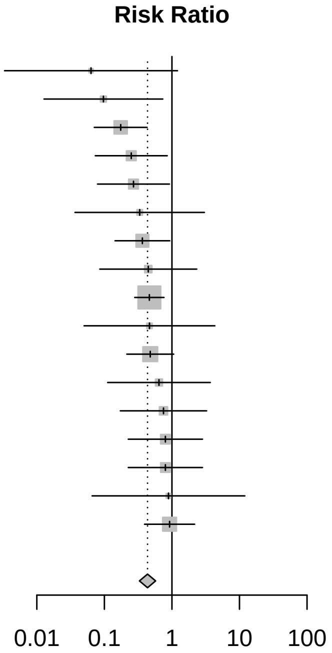

Heterogeneity: $I^2 = 0\%$, $\tau^2 = 0.0095$, $p = 0.51$

4.7.4 Funnel plot

```
funnel(trimfill(comp_metabin))
```

```
## Warning in trimfill.meta(comp_metabin): 1 observation(s) dropped due to missing
## values
```

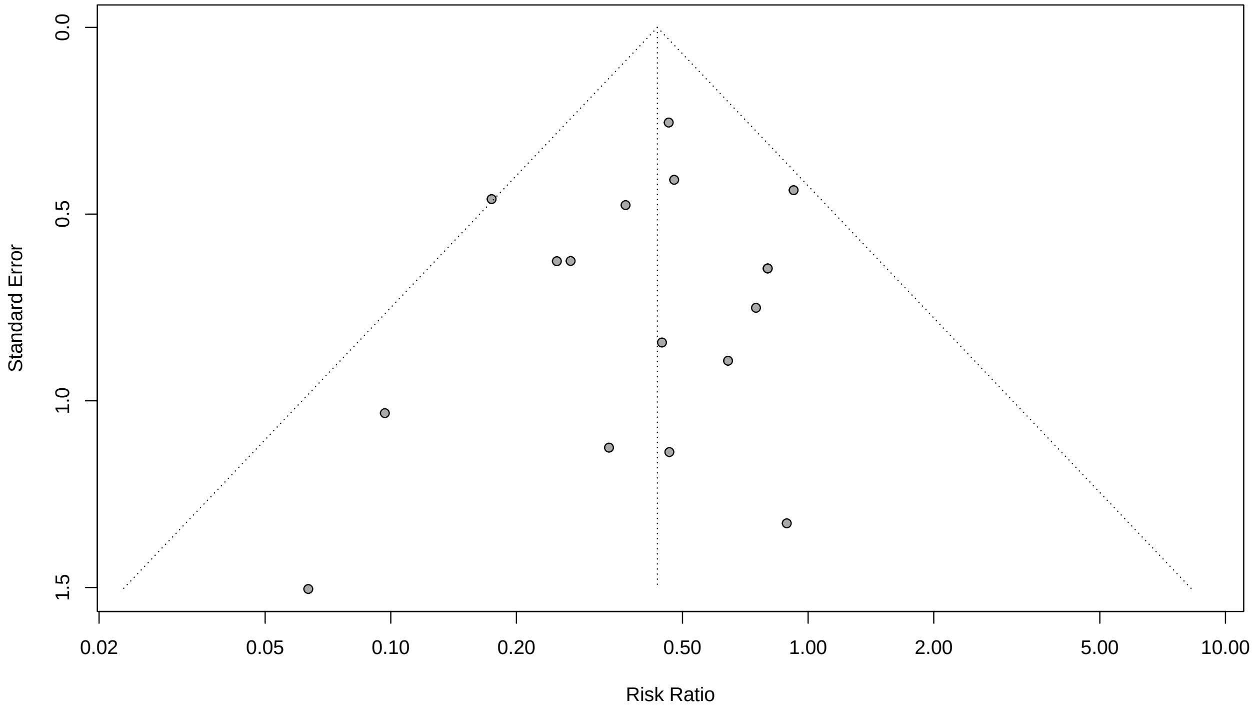

4.7.5 Baujat

```r
baujat(comp_metabin, pos = 1)
```

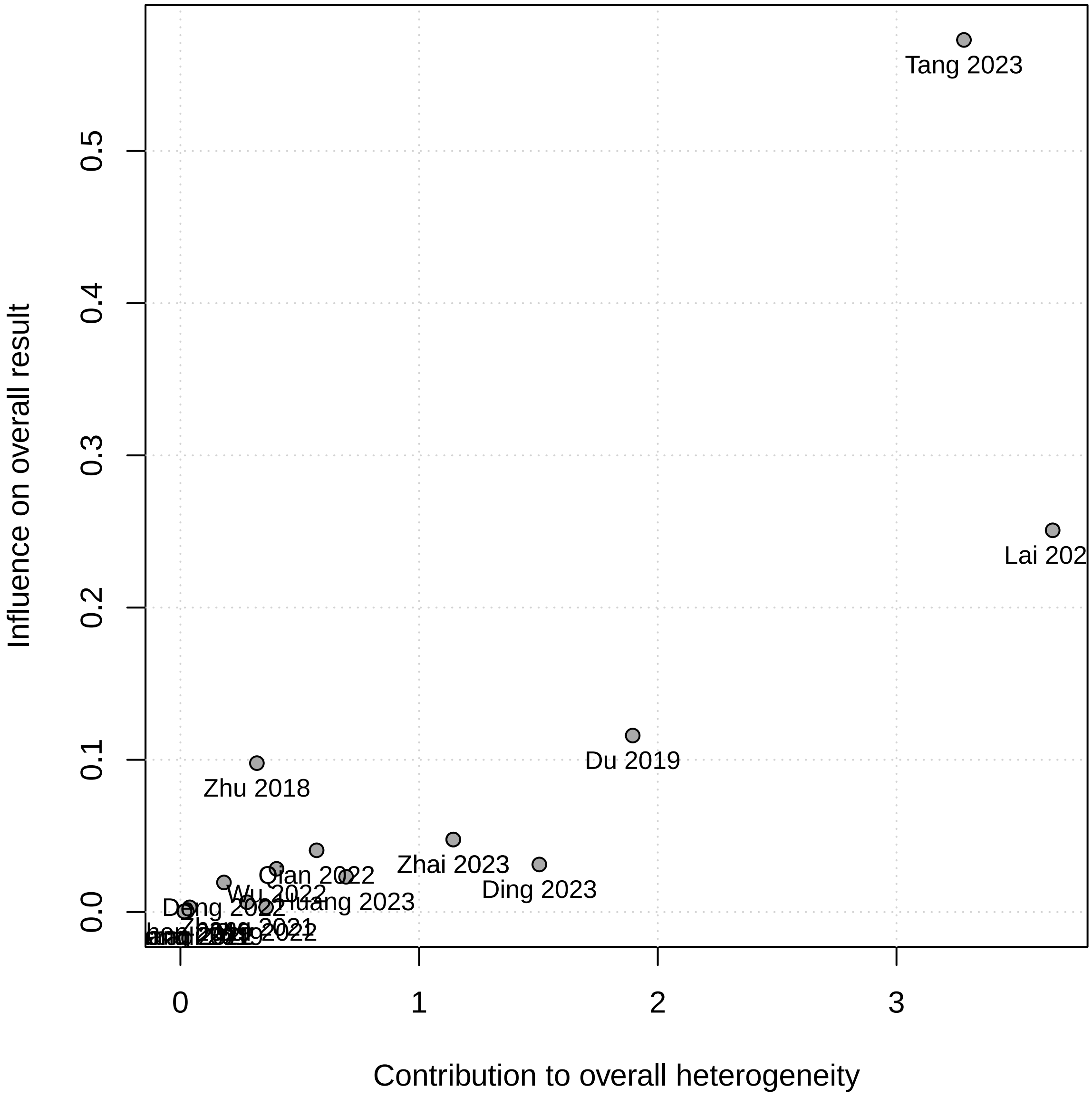

4.7.6 Leave one out

```
metainf(comp_metabin)
```

```
## Influential analysis (common effect model)
##
##                                 RR           95%-CI  p-value   tau^2     tau
## Omitting Sur 2022           0.3982 [0.3065; 0.5174] < 0.0001  0.0146  0.1208
## Omitting Tang 2023          0.4459 [0.3391; 0.5865] < 0.0001  0.0000  0.0000
## Omitting Chen 2019          0.4043 [0.3083; 0.5302] < 0.0001  0.0376  0.1938
## Omitting Du 2019            0.4200 [0.3227; 0.5465] < 0.0001  0.0000  0.0000
## Omitting Lai 2020           0.3740 [0.2841; 0.4923] < 0.0001  0.0000  0.0000
## Omitting Zhu 2018           0.3821 [0.2817; 0.5184] < 0.0001  0.0760  0.2757
## Omitting Zhang 2021         0.4001 [0.3074; 0.5207] < 0.0001  0.0215  0.1467
## Omitting Zhang 2021         0.3969 [0.3050; 0.5165] < 0.0001  0.0201  0.1417
## Omitting Lechevallier 2003  0.4012 [0.3092; 0.5205] < 0.0001  0.0095  0.0975
## Omitting Huang 2023         0.3930 [0.3016; 0.5122] < 0.0001  0.0208  0.1444
## Omitting Wu 2022            0.4105 [0.3145; 0.5358] < 0.0001  0.0151  0.1230
## Omitting Ding 2023          0.4108 [0.3160; 0.5341] < 0.0001  0.0052  0.0719
## Omitting Zhai 2023          0.3894 [0.2983; 0.5085] < 0.0001  0.0187  0.1368
## Omitting Zhai 2023          0.3894 [0.2983; 0.5085] < 0.0001  0.0187  0.1368
## Omitting Qian 2022          0.4123 [0.3158; 0.5383] < 0.0001  0.0105  0.1023
## Omitting Zhang 2022         0.4024 [0.3096; 0.5230] < 0.0001  0.0151  0.1230
## Omitting Deng 2022          0.3934 [0.2987; 0.5181] < 0.0001  0.0521  0.2282
## Omitting AlSmadi 2019       0.4003 [0.3080; 0.5202] < 0.0001  0.0162  0.1273
##
## Pooled estimate             0.4012 [0.3092; 0.5205] < 0.0001  0.0095  0.0975
##                               I^2
## Omitting Sur 2022            0.0%
## Omitting Tang 2023           0.0%
## Omitting Chen 2019           0.0%
## Omitting Du 2019             0.0%
## Omitting Lai 2020            0.0%
## Omitting Zhu 2018            0.5%
## Omitting Zhang 2021          1.0%
## Omitting Zhang 2021          0.0%
## Omitting Lechevallier 2003   0.0%
## Omitting Huang 2023          0.0%
## Omitting Wu 2022             0.0%
## Omitting Ding 2023           0.0%
## Omitting Zhai 2023           0.0%
## Omitting Zhai 2023           0.0%
## Omitting Qian 2022           0.0%
## Omitting Zhang 2022          0.6%
## Omitting Deng 2022           0.6%
## Omitting AlSmadi 2019        1.0%
##
## Pooled estimate              0.0%
##
## Details on meta-analytical method:
## - Mantel-Haenszel method
## - Restricted maximum-likelihood estimator for tau^2
```

4.8 Fever

4.8.1 Meta-analysis

```
fever_metabin <- metabin(data = suction_data,
                          event.c = fever_n_control,
                        n.c = sample_size_control,
                        event.e = fever_n_suction,
                        n.e = sample_size_suction,
                        studlab = author_year)
fever_metabin
```

```
## Number of studies: k = 13
## Number of observations: o = 2311
## Number of events: e = 129
##
##                             RR           95%-CI     z  p-value
## Common effect model  0.4058 [0.2814; 0.5852] -4.83 < 0.0001
## Random effects model 0.4371 [0.2985; 0.6402] -4.25 < 0.0001
##
## Quantifying heterogeneity:
##  tau^2 = 0 [0.0000; 0.8333]; tau = 0 [0.0000; 0.9129]
##  I^2 = 0.0% [0.0%; 56.6%]; H = 1.00 [1.00; 1.52]
##
## Test of heterogeneity:
##     Q d.f. p-value
##  9.84   12  0.6299
##
## Details on meta-analytical method:
## - Mantel-Haenszel method
## - Inverse variance method
## - Restricted maximum-likelihood estimator for tau^2
## - Q-Profile method for confidence interval of tau^2 and tau
## - Continuity correction of 0.5 in studies with zero cell frequencies
```

4.8.2 Forest plot

```r
forest(fever_metabin,
       sortvar = TE)
```

Study	Experimental Events	Experimental Total	Control Events	Control Total	RR	95%−CI	Weight (common)	Weight (random)
Ding 2023	0	138	3	61	0.06	[0.00; 1.21]	5.2%	1.7%
Du 2019	1	62	7	60	0.14	[0.02; 1.09]	7.7%	3.4%
Tang 2023	2	86	9	87	0.22	[0.05; 1.01]	9.7%	6.4%
Qian 2022	3	81	12	81	0.25	[0.07; 0.85]	13.0%	9.7%
Wu 2022	3	76	12	82	0.27	[0.08; 0.92]	12.5%	9.7%
Zhang 2022	1	30	3	30	0.33	[0.04; 3.03]	3.2%	3.0%
Zhu 2018	9	165	23	165	0.39	[0.19; 0.82]	24.9%	26.6%
Zhang 2021	2	56	3	50	0.60	[0.10; 3.42]	3.4%	4.8%
Zhang 2021	2	56	3	54	0.64	[0.11; 3.70]	3.3%	4.8%
Huang 2023	3	103	4	103	0.75	[0.17; 3.27]	4.3%	6.7%
Lai 2020	2	28	5	56	0.80	[0.17; 3.87]	3.6%	5.9%
Chen 2019	3	44	3	45	1.02	[0.22; 4.80]	3.2%	6.1%
Deng 2022	5	57	6	70	1.02	[0.33; 3.18]	5.8%	11.3%
Sur 2022	0	9	0	8			0.0%	0.0%
Lechevallier 2003	.	11	.	14			0.0%	0.0%
Zhai 2023	.	60	.	60			0.0%	0.0%
Zhai 2023	.	60	.	60			0.0%	0.0%
AlSmadi 2019	.	43	.	60			0.0%	0.0%
**Common effect model**		**1165**		**1146**	**0.41**	**[0.28; 0.59]**	**100.0%**	**−−**
**Random effects model**					**0.44**	**[0.30; 0.64]**	**−−**	**100.0%**

Heterogeneity: $I^2 = 0\%$, $\tau^2 = 0$, $p = 0.63$

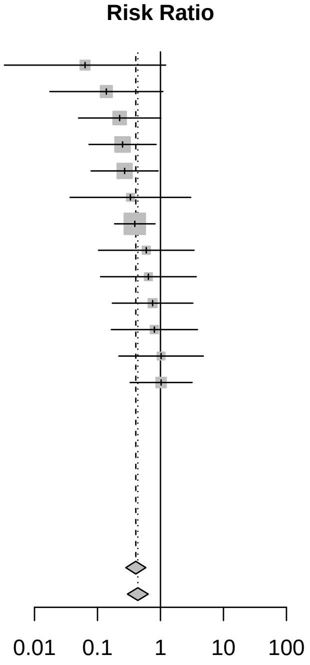

4.8.3 Trim and Fill

```r
trimfill(fever_metabin)
```

```
## Warning in trimfill.meta(fever_metabin): 5 observation(s) dropped due to
## missing values

## Number of studies: k = 14 (with 1 added studies)
## Number of observations: o = 2125
## Number of events: e = 132
##
##                            RR           95%-CI      z  p-value
## Random effects model 0.4517 [0.3094; 0.6595] -4.12 < 0.0001
##
## Quantifying heterogeneity:
##  tau^2 < 0.0001 [0.0000; 1.0892]; tau = 0.0025 [0.0000; 1.0436]
##  I^2 = 0.0% [0.0%; 55.0%]; H = 1.00 [1.00; 1.49]
##
## Test of heterogeneity:
##      Q d.f. p-value
##  11.57   13  0.5630
##
## Details on meta-analytical method:
## - Inverse variance method
## - Restricted maximum-likelihood estimator for tau^2
## - Q-Profile method for confidence interval of tau^2 and tau
## - Trim-and-fill method to adjust for funnel plot asymmetry (L-estimator)
```

```r
forest(trimfill(fever_metabin),
       sortvar = TE)
```

```
## Warning in trimfill.meta(fever_metabin): 5 observation(s) dropped due to
## missing values
```

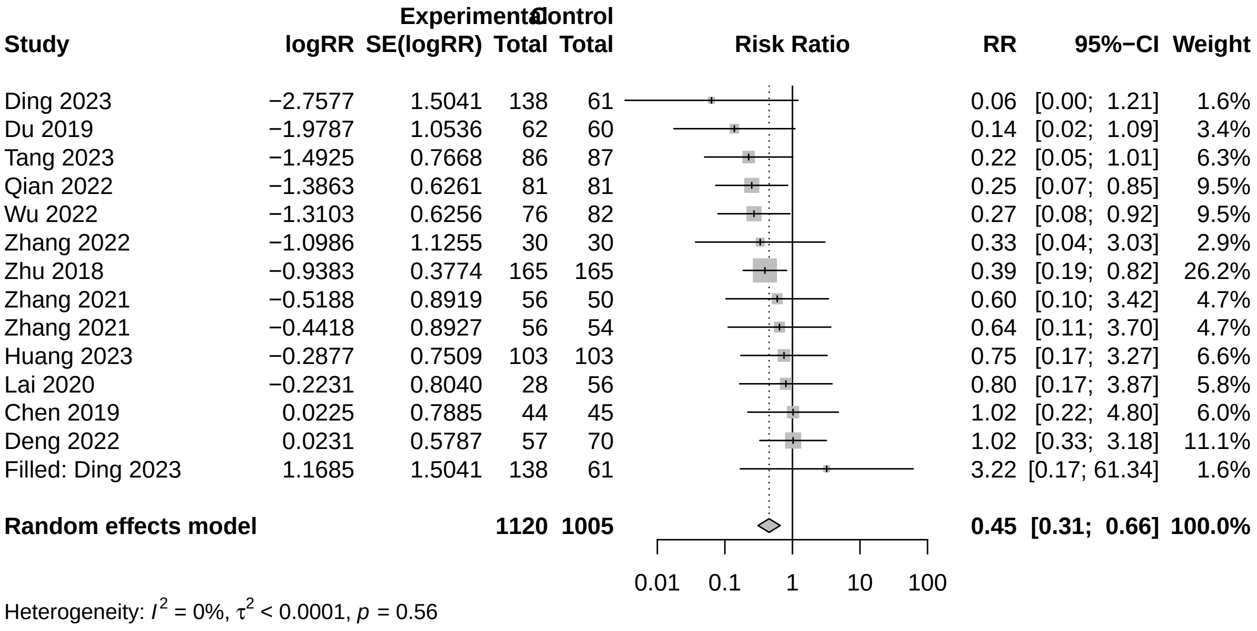

4.8.4 Funnel plot

```
funnel(trimfill(fever_metabin))
```

```
## Warning in trimfill.meta(fever_metabin): 5 observation(s) dropped due to
## missing values
```

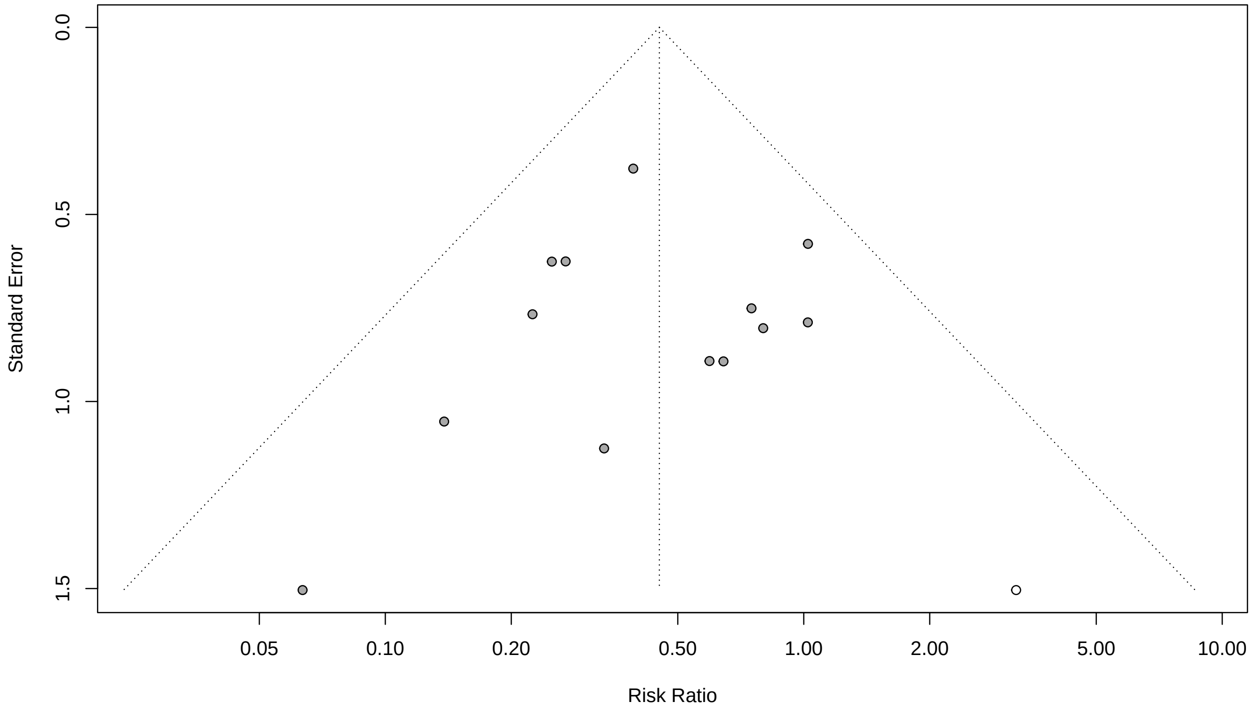

4.8.5 Baujat

```
baujat(fever_metabin, pos = 1)
```

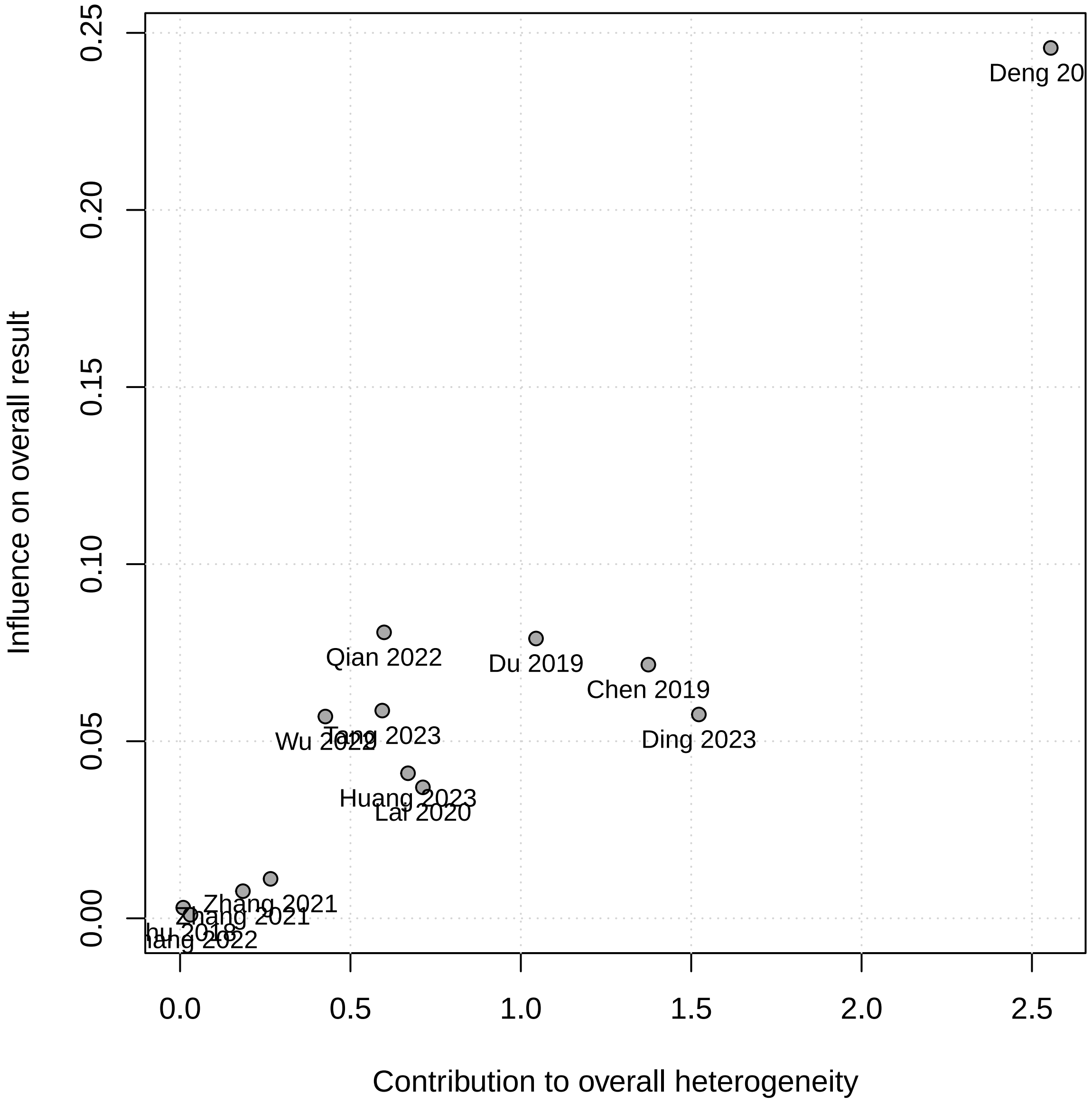

4.8.6 Leave one out

```
metainf(fever_metabin)
```

```
## Influential analysis (common effect model)
##
##                                   RR           95%-CI  p-value   tau^2     tau
## Omitting Sur 2022             0.4058 [0.2814; 0.5852] < 0.0001  0.0000  0.0000
## Omitting Tang 2023            0.4252 [0.2913; 0.6207] < 0.0001  0.0000  0.0000
## Omitting Chen 2019            0.3853 [0.2638; 0.5630] < 0.0001  0.0000  0.0000
## Omitting Du 2019              0.4281 [0.2947; 0.6221] < 0.0001  0.0000  0.0000
## Omitting Lai 2020             0.3911 [0.2681; 0.5705] < 0.0001  0.0000  0.0000
## Omitting Zhu 2018             0.4106 [0.2694; 0.6258] < 0.0001  0.0000  0.0000
## Omitting Zhang 2021           0.3991 [0.2744; 0.5805] < 0.0001  0.0000  0.0000
## Omitting Zhang 2021           0.3977 [0.2734; 0.5786] < 0.0001  0.0000  0.0000
## Omitting Lechevallier 2003    0.4058 [0.2814; 0.5852] < 0.0001  0.0000  0.0000
## Omitting Huang 2023           0.3902 [0.2671; 0.5702] < 0.0001  0.0000  0.0000
## Omitting Wu 2022              0.4253 [0.2896; 0.6245] < 0.0001  0.0000  0.0000
## Omitting Ding 2023            0.4247 [0.2927; 0.6163] < 0.0001  0.0000  0.0000
## Omitting Zhai 2023            0.4058 [0.2814; 0.5852] < 0.0001  0.0000  0.0000
## Omitting Zhai 2023            0.4058 [0.2814; 0.5852] < 0.0001  0.0000  0.0000
## Omitting Qian 2022            0.4291 [0.2921; 0.6303] < 0.0001  0.0000  0.0000
## Omitting Zhang 2022           0.4082 [0.2816; 0.5918] < 0.0001  0.0000  0.0019
## Omitting Deng 2022            0.3676 [0.2485; 0.5436] < 0.0001  0.0000  0.0000
## Omitting AlSmadi 2019         0.4058 [0.2814; 0.5852] < 0.0001  0.0000  0.0000
##
## Pooled estimate               0.4058 [0.2814; 0.5852] < 0.0001  0.0000  0.0000
##                                  I^2
## Omitting Sur 2022               0.0%
## Omitting Tang 2023              0.0%
## Omitting Chen 2019              0.0%
## Omitting Du 2019                0.0%
## Omitting Lai 2020               0.0%
## Omitting Zhu 2018               0.0%
## Omitting Zhang 2021             0.0%
## Omitting Zhang 2021             0.0%
## Omitting Lechevallier 2003      0.0%
## Omitting Huang 2023             0.0%
## Omitting Wu 2022                0.0%
## Omitting Ding 2023              0.0%
## Omitting Zhai 2023              0.0%
## Omitting Zhai 2023              0.0%
## Omitting Qian 2022              0.0%
## Omitting Zhang 2022             0.0%
## Omitting Deng 2022              0.0%
## Omitting AlSmadi 2019           0.0%
##
## Pooled estimate                 0.0%
##
## Details on meta-analytical method:
## - Mantel-Haenszel method
## - Restricted maximum-likelihood estimator for tau^2
```

4.9 Infections

4.9.1 Meta-analysis

```
infection_metabin <- metabin(data = suction_data,
                              event.c = infection_n_control,
                            n.c = sample_size_control,
                            event.e = infection_n_suction,
                            n.e = sample_size_suction,
                            studlab = author_year)
infection_metabin
```

```
## Number of studies: k = 14
## Number of observations: o = 2311
## Number of events: e = 126
##
##                             RR           95%-CI     z  p-value
## Common effect model  0.3983 [0.2754; 0.5762] -4.89 < 0.0001
## Random effects model 0.4285 [0.2916; 0.6296] -4.32 < 0.0001
##
## Quantifying heterogeneity:
##  tau^2 = 0 [0.0000; 0.6581]; tau = 0 [0.0000; 0.8113]
##  I^2 = 0.0% [0.0%; 55.0%]; H = 1.00 [1.00; 1.49]
##
## Test of heterogeneity:
##     Q d.f. p-value
##  9.80   13  0.7103
##
## Details on meta-analytical method:
## - Mantel-Haenszel method
## - Inverse variance method
## - Restricted maximum-likelihood estimator for tau^2
## - Q-Profile method for confidence interval of tau^2 and tau
## - Continuity correction of 0.5 in studies with zero cell frequencies
```

4.9.2 Forest plot

```r
forest(infection_metabin,
       sortvar = TE)
```

Study	Experimental Events	Experimental Total	Control Events	Control Total	RR	95%−CI	Weight (common)	Weight (random)
Ding 2023	0	138	3	61	0.06	[0.00; 1.21]	5.3%	1.7%
Du 2019	1	62	7	60	0.14	[0.02; 1.09]	7.7%	3.5%
Tang 2023	2	86	9	87	0.22	[0.05; 1.01]	9.7%	6.6%
Qian 2022	3	81	12	81	0.25	[0.07; 0.85]	13.1%	9.8%
Wu 2022	3	76	12	82	0.27	[0.08; 0.92]	12.6%	9.9%
Sur 2022	0	9	1	8	0.30	[0.01; 6.39]	1.7%	1.6%
Zhang 2022	1	30	3	30	0.33	[0.04; 3.03]	3.3%	3.0%
Zhu 2018	9	165	23	165	0.39	[0.19; 0.82]	25.0%	27.1%
Zhang 2021	2	56	3	50	0.60	[0.10; 3.42]	3.4%	4.8%
Zhang 2021	2	56	3	54	0.64	[0.11; 3.70]	3.3%	4.8%
Huang 2023	3	103	4	103	0.75	[0.17; 3.27]	4.4%	6.8%
Lai 2020	1	28	2	56	1.00	[0.09; 10.56]	1.5%	2.7%
Chen 2019	3	44	3	45	1.02	[0.22; 4.80]	3.2%	6.2%
Deng 2022	5	57	6	70	1.02	[0.33; 3.18]	5.9%	11.5%
Lechevallier 2003	.	11	.	14			0.0%	0.0%
Zhai 2023	.	60	.	60			0.0%	0.0%
Zhai 2023	.	60	.	60			0.0%	0.0%
AlSmadi 2019	.	43	.	60			0.0%	0.0%
**Common effect model**		**1165**		**1146**	**0.40**	**[0.28; 0.58]**	**100.0%**	**--**
**Random effects model**					**0.43**	**[0.29; 0.63]**	**--**	**100.0%**

Heterogeneity: $I^2 = 0\%$, $\tau^2 = 0$, $p = 0.71$

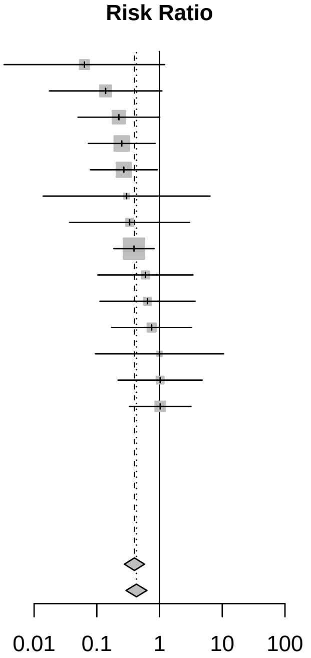

4.9.3 Trim and Fill

```
trimfill(infection_metabin)
```

```
## Warning in trimfill.meta(infection_metabin): 4 observation(s) dropped due to
## missing values
```

```
## Number of studies: k = 15 (with 1 added studies)
## Number of observations: o = 2142
## Number of events: e = 129
##
##                           RR           95%-CI     z  p-value
## Random effects model 0.4429 [0.3024; 0.6487] -4.18 < 0.0001
##
## Quantifying heterogeneity:
##  tau^2 = 0 [0.0000; 0.8711]; tau = 0 [0.0000; 0.9333]
##  I^2 = 0.0% [0.0%; 53.6%]; H = 1.00 [1.00; 1.47]
##
## Test of heterogeneity:
##      Q d.f. p-value
##  11.50   14  0.6467
##
## Details on meta-analytical method:
## - Inverse variance method
## - Restricted maximum-likelihood estimator for tau^2
## - Q-Profile method for confidence interval of tau^2 and tau
## - Trim-and-fill method to adjust for funnel plot asymmetry (L-estimator)
```

```
forest(trimfill(infection_metabin),
       sortvar = TE)
```

```
## Warning in trimfill.meta(infection_metabin): 4 observation(s) dropped due to
## missing values
```

**Study**	**logRR**	**SE(logRR)**	**Experimental Total**	**Control Total**	**Risk Ratio**	**RR**	**95%−CI**	**Weight**
Ding 2023	−2.7577	1.5041	138	61		0.06	[0.00; 1.21]	1.7%
Du 2019	−1.9787	1.0536	62	60		0.14	[0.02; 1.09]	3.4%
Tang 2023	−1.4925	0.7668	86	87		0.22	[0.05; 1.01]	6.4%
Qian 2022	−1.3863	0.6261	81	81		0.25	[0.07; 0.85]	9.7%
Wu 2022	−1.3103	0.6256	76	82		0.27	[0.08; 0.92]	9.7%
Sur 2022	−1.2098	1.5633	9	8		0.30	[0.01; 6.39]	1.6%
Zhang 2022	−1.0986	1.1255	30	30		0.33	[0.04; 3.03]	3.0%
Zhu 2018	−0.9383	0.3774	165	165		0.39	[0.19; 0.82]	26.6%
Zhang 2021	−0.5188	0.8919	56	50		0.60	[0.10; 3.42]	4.8%
Zhang 2021	−0.4418	0.8927	56	54		0.64	[0.11; 3.70]	4.8%
Huang 2023	−0.2877	0.7509	103	103		0.75	[0.17; 3.27]	6.7%
Lai 2020	0.0000	1.2027	28	56		1.00	[0.09; 10.56]	2.6%
Chen 2019	0.0225	0.7885	44	45		1.02	[0.22; 4.80]	6.1%
Deng 2022	0.0231	0.5787	57	70		1.02	[0.33; 3.18]	11.3%
Filled: Ding 2023	1.1289	1.5041	138	61		3.09	[0.16; 58.96]	1.7%
**Random effects model**			**1129**	**1013**		**0.44**	**[0.30; 0.65]**	**100.0%**

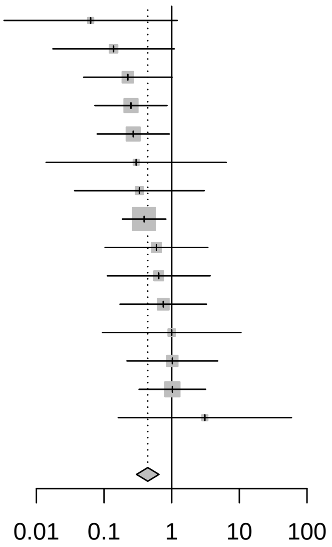

Heterogeneity: $I^2 = 0\%$, $\tau^2 = 0$, $p = 0.65$

4.9.4 Funnel plot

```
funnel(trimfill(infection_metabin))
```

```
## Warning in trimfill.meta(infection_metabin): 4 observation(s) dropped due to
## missing values
```

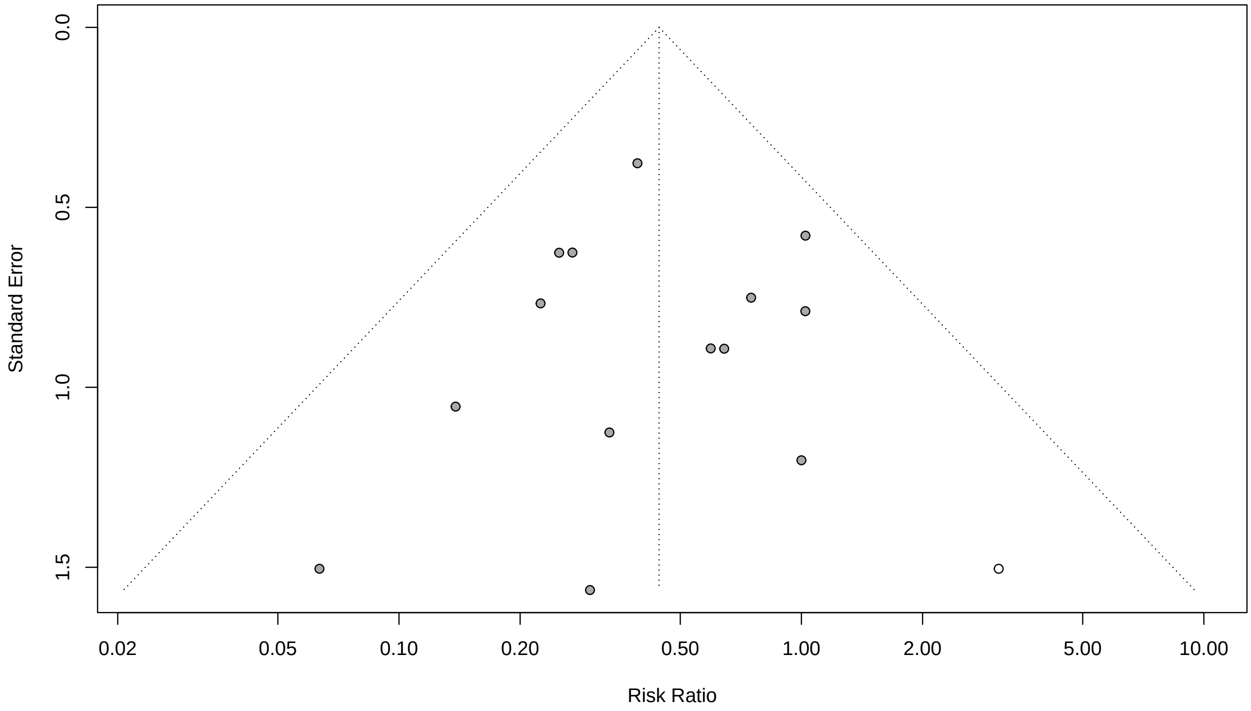

4.9.5 Baujat

```r
baujat(infection_metabin, pos = 1)
```

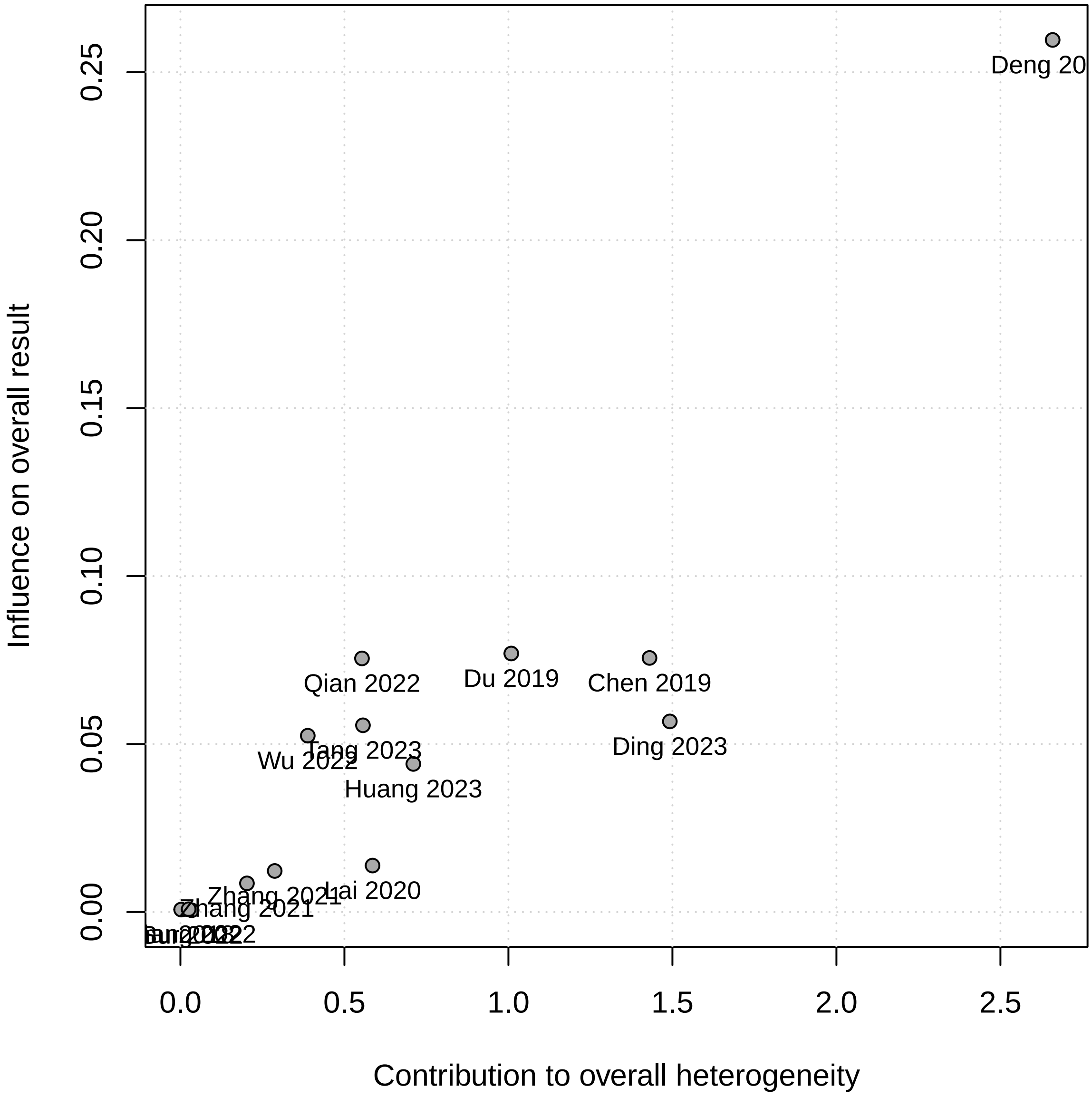

4.9.6 Leave one out

```r
metainf(infection_metabin)
```

```
## Influential analysis (common effect model)
##
##                                   RR           95%-CI  p-value   tau^2     tau
## Omitting Sur 2022             0.4000 [0.2758; 0.5803] < 0.0001  0.0000  0.0000
## Omitting Tang 2023            0.4170 [0.2847; 0.6108] < 0.0001  0.0000  0.0000
## Omitting Chen 2019            0.3775 [0.2575; 0.5534] < 0.0001  0.0000  0.0000
## Omitting Du 2019              0.4201 [0.2883; 0.6124] < 0.0001  0.0000  0.0000
## Omitting Lai 2020             0.3895 [0.2677; 0.5665] < 0.0001  0.0000  0.0000
## Omitting Zhu 2018             0.4007 [0.2617; 0.6135] < 0.0001  0.0000  0.0000
## Omitting Zhang 2021           0.3913 [0.2681; 0.5710] < 0.0001  0.0000  0.0000
## Omitting Zhang 2021           0.3899 [0.2671; 0.5691] < 0.0001  0.0000  0.0000
## Omitting Lechevallier 2003    0.3983 [0.2754; 0.5762] < 0.0001  0.0000  0.0000
## Omitting Huang 2023           0.3823 [0.2608; 0.5605] < 0.0001  0.0000  0.0000
## Omitting Wu 2022              0.4168 [0.2829; 0.6142] < 0.0001  0.0000  0.0000
## Omitting Ding 2023            0.4169 [0.2864; 0.6069] < 0.0001  0.0000  0.0000
## Omitting Zhai 2023            0.3983 [0.2754; 0.5762] < 0.0001  0.0000  0.0000
## Omitting Zhai 2023            0.3983 [0.2754; 0.5762] < 0.0001  0.0000  0.0000
## Omitting Qian 2022            0.4206 [0.2853; 0.6200] < 0.0001  0.0000  0.0000
## Omitting Zhang 2022           0.4005 [0.2754; 0.5824] < 0.0001  0.0000  0.0000
## Omitting Deng 2022            0.3594 [0.2421; 0.5337] < 0.0001  0.0000  0.0000
## Omitting AlSmadi 2019         0.3983 [0.2754; 0.5762] < 0.0001  0.0000  0.0000
##
## Pooled estimate               0.3983 [0.2754; 0.5762] < 0.0001  0.0000  0.0000
##                                 I^2
## Omitting Sur 2022              0.0%
## Omitting Tang 2023             0.0%
## Omitting Chen 2019             0.0%
## Omitting Du 2019               0.0%
## Omitting Lai 2020              0.0%
## Omitting Zhu 2018              0.0%
## Omitting Zhang 2021            0.0%
## Omitting Zhang 2021            0.0%
## Omitting Lechevallier 2003     0.0%
## Omitting Huang 2023            0.0%
## Omitting Wu 2022               0.0%
## Omitting Ding 2023             0.0%
## Omitting Zhai 2023             0.0%
## Omitting Zhai 2023             0.0%
## Omitting Qian 2022             0.0%
## Omitting Zhang 2022            0.0%
## Omitting Deng 2022             0.0%
## Omitting AlSmadi 2019          0.0%
##
## Pooled estimate                0.0%
##
## Details on meta-analytical method:
## - Mantel-Haenszel method
## - Restricted maximum-likelihood estimator for tau^2
```

4.10 Sepsis

4.10.1 Meta-analysis

```
sepsis_metabin <- metabin(data = suction_data,
                         event.c = sepsis_n_control,
                       n.c = sample_size_control,
                       event.e = sepsis_n_suction,
                       n.e = sample_size_suction,
                       studlab = author_year)
sepsis_metabin
```

```
## Number of studies: k = 2
## Number of observations: o = 2311
## Number of events: e = 18
##
##                          RR           95%-CI     z p-value
## Common effect model  0.2264 [0.0719; 0.7124] -2.54  0.0111
## Random effects model 0.2371 [0.0747; 0.7524] -2.44  0.0146
##
## Quantifying heterogeneity:
##  tau^2 = 0; tau = 0; I^2 = 0.0%; H = 1.00
##
## Test of heterogeneity:
##     Q d.f. p-value
##  0.30    1  0.5833
##
## Details on meta-analytical method:
## - Mantel-Haenszel method
## - Inverse variance method
## - Restricted maximum-likelihood estimator for tau^2
## - Continuity correction of 0.5 in studies with zero cell frequencies
```

4.10.2 Forest plot

```r
forest(sepsis_metabin,
       sortvar = TE)
```

Study	Experimental Events	Experimental Total	Control Events	Control Total	RR	95%−CI	Weight (common)	Weight (random)
Tang 2023	0	86	4	87	0.11	[0.01; 2.06]	28.9%	15.8%
Zhu 2018	3	165	11	165	0.27	[0.08; 0.96]	71.1%	84.2%
Sur 2022	0	9	0	8			0.0%	0.0%
Chen 2019	0	44	0	45			0.0%	0.0%
Du 2019	0	62	0	60			0.0%	0.0%
Lai 2020	0	28	0	56			0.0%	0.0%
Zhang 2021	0	56	0	50			0.0%	0.0%
Zhang 2021	0	56	0	54			0.0%	0.0%
Lechevallier 2003	.	11	.	14			0.0%	0.0%
Huang 2023	0	103	0	103			0.0%	0.0%
Wu 2022	0	76	0	82			0.0%	0.0%
Ding 2023	0	138	0	61			0.0%	0.0%
Zhai 2023	0	60	0	60			0.0%	0.0%
Zhai 2023	0	60	0	60			0.0%	0.0%
Qian 2022	0	81	0	81			0.0%	0.0%
Zhang 2022	0	30	0	30			0.0%	0.0%
Deng 2022	0	57	0	70			0.0%	0.0%
AlSmadi 2019	0	43	0	60			0.0%	0.0%
**Common effect model**		**1165**		**1146**	**0.23**	**[0.07; 0.71]**	**100.0%**	**−−**
**Random effects model**					**0.24**	**[0.07; 0.75]**	**−−**	**100.0%**

Heterogeneity: $I^2 = 0\%$, $\tau^2 = 0$, $p = 0.58$

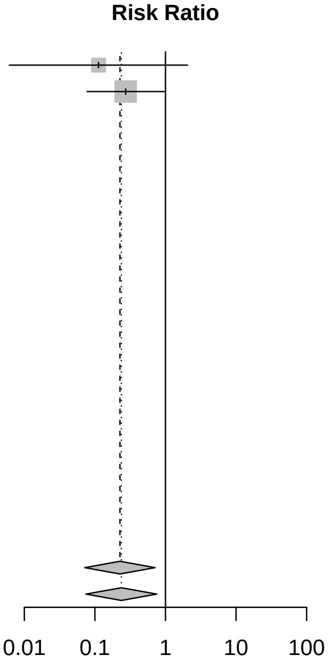

4.10.3 Trim and Fill

```
#trimfill(sepsis_metabin)
#forest(trimfill(sepsis_metabin),
#       sortvar = TE)
```

4.10.4 Funnel plot

```
funnel((sepsis_metabin))
```

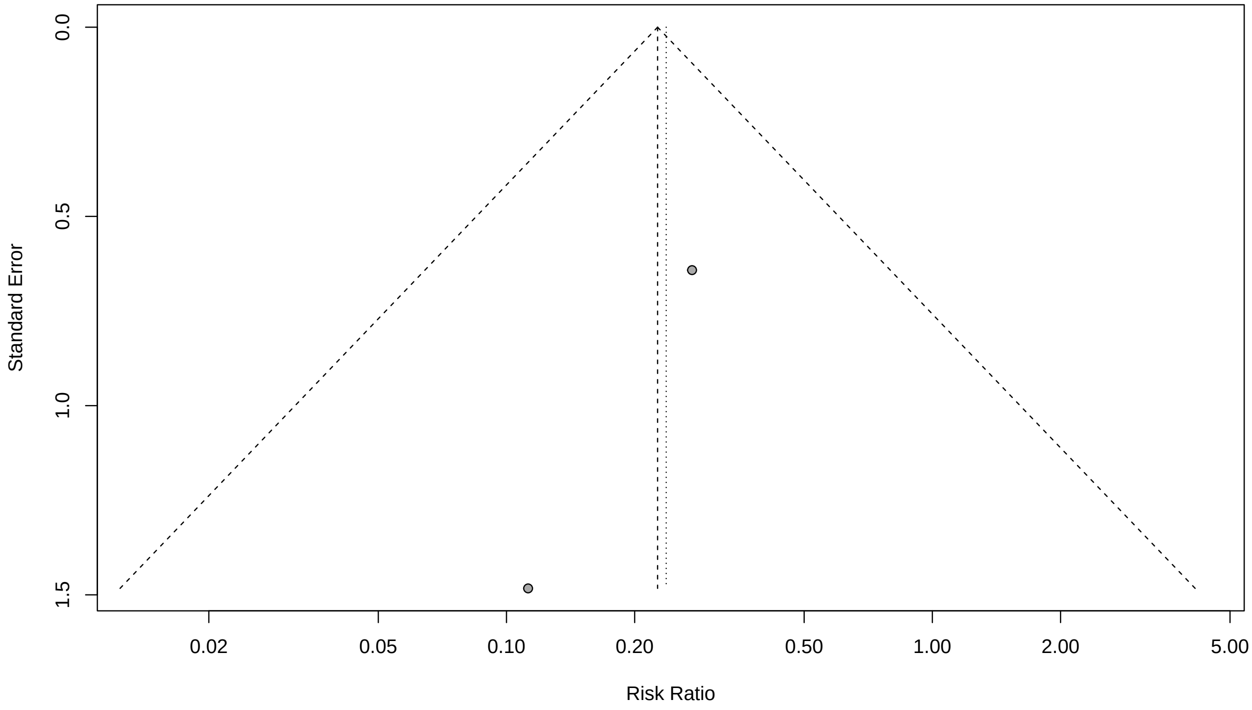

4.10.5 Baujat

```
baujat(sepsis_metabin, pos = 1)
```

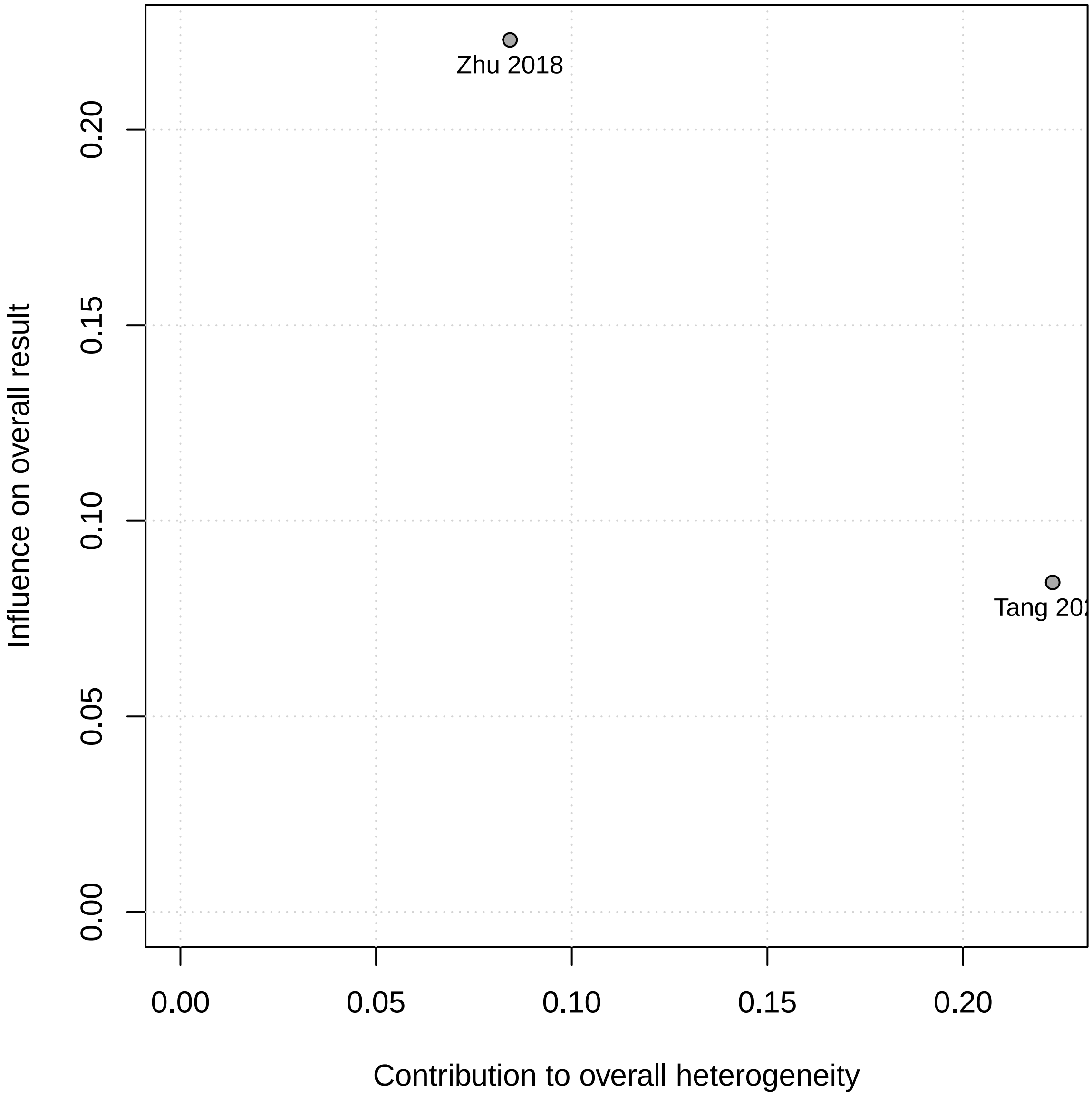

4.10.6 Leave one out

```
metainf(sepsis_metabin)
```

```
## Influential analysis (common effect model)
##
##                                  RR           95%-CI p-value   tau^2     tau
## Omitting Sur 2022            0.2264 [0.0719; 0.7124]  0.0111  0.0000  0.0000
## Omitting Tang 2023           0.2727 [0.0775; 0.9598]  0.0430
## Omitting Chen 2019           0.2264 [0.0719; 0.7124]  0.0111  0.0000  0.0000
## Omitting Du 2019             0.2264 [0.0719; 0.7124]  0.0111  0.0000  0.0000
## Omitting Lai 2020            0.2264 [0.0719; 0.7124]  0.0111  0.0000  0.0000
## Omitting Zhu 2018            0.1124 [0.0061; 2.0562]  0.1405
## Omitting Zhang 2021          0.2264 [0.0719; 0.7124]  0.0111  0.0000  0.0000
## Omitting Zhang 2021          0.2264 [0.0719; 0.7124]  0.0111  0.0000  0.0000
## Omitting Lechevallier 2003   0.2264 [0.0719; 0.7124]  0.0111  0.0000  0.0000
## Omitting Huang 2023          0.2264 [0.0719; 0.7124]  0.0111  0.0000  0.0000
## Omitting Wu 2022             0.2264 [0.0719; 0.7124]  0.0111  0.0000  0.0000
## Omitting Ding 2023           0.2264 [0.0719; 0.7124]  0.0111  0.0000  0.0000
## Omitting Zhai 2023           0.2264 [0.0719; 0.7124]  0.0111  0.0000  0.0000
## Omitting Zhai 2023           0.2264 [0.0719; 0.7124]  0.0111  0.0000  0.0000
## Omitting Qian 2022           0.2264 [0.0719; 0.7124]  0.0111  0.0000  0.0000
## Omitting Zhang 2022          0.2264 [0.0719; 0.7124]  0.0111  0.0000  0.0000
## Omitting Deng 2022           0.2264 [0.0719; 0.7124]  0.0111  0.0000  0.0000
## Omitting AlSmadi 2019        0.2264 [0.0719; 0.7124]  0.0111  0.0000  0.0000
##
## Pooled estimate              0.2264 [0.0719; 0.7124]  0.0111  0.0000  0.0000
##                                 I^2
## Omitting Sur 2022              0.0%
## Omitting Tang 2023
## Omitting Chen 2019             0.0%
## Omitting Du 2019               0.0%
## Omitting Lai 2020              0.0%
## Omitting Zhu 2018
## Omitting Zhang 2021            0.0%
## Omitting Zhang 2021            0.0%
## Omitting Lechevallier 2003     0.0%
## Omitting Huang 2023            0.0%
## Omitting Wu 2022               0.0%
## Omitting Ding 2023             0.0%
## Omitting Zhai 2023             0.0%
## Omitting Zhai 2023             0.0%
## Omitting Qian 2022             0.0%
## Omitting Zhang 2022            0.0%
## Omitting Deng 2022             0.0%
## Omitting AlSmadi 2019          0.0%
##
## Pooled estimate                0.0%
##
## Details on meta-analytical method:
## - Mantel-Haenszel method
## - Restricted maximum-likelihood estimator for tau^2
```

4.11 Abscess

4.11.1 Meta-analysis

```
abscess_metabin <- metabin(data = suction_data,
                         event.c = abscess_n_control,
                       n.c = sample_size_control,
                       event.e = abscess_n_suction,
                       n.e = sample_size_suction,
                       studlab = author_year)
abscess_metabin
```

```
## Number of studies: k = 0
## Number of observations: o = 2311
## Number of events: e = 0
##
##                        RR 95%-CI  z p-value
## Common effect model  NA        --      --
## Random effects model NA        --      --
##
## Quantifying heterogeneity:
##  tau^2 = NA; tau = NA; I^2 = NA; H = NA
##
## Details on meta-analytical method:
## - Mantel-Haenszel method
## - Inverse variance method
## - Restricted maximum-likelihood estimator for tau^2
```

4.11.2 Forest plot

```r
forest(abscess_metabin,
       sortvar = TE)
```

Study	Experimental Events	Experimental Total	Control Events	Control Total	Risk Ratio	RR	95%−CI	Weight (common)	Weight (random)
Sur 2022	0	9	0	8				0.0%	0.0%
Tang 2023	0	86	0	87				0.0%	0.0%
Chen 2019	0	44	0	45				0.0%	0.0%
Du 2019	0	62	0	60				0.0%	0.0%
Lai 2020	0	28	0	56				0.0%	0.0%
Zhu 2018	0	165	0	165				0.0%	0.0%
Zhang 2021	0	56	0	50				0.0%	0.0%
Zhang 2021	0	56	0	54				0.0%	0.0%
Lechevallier 2003	.	11	.	14				0.0%	0.0%
Huang 2023	0	103	0	103				0.0%	0.0%
Wu 2022	0	76	0	82				0.0%	0.0%
Ding 2023	0	138	0	61				0.0%	0.0%
Zhai 2023	0	60	0	60				0.0%	0.0%
Zhai 2023	0	60	0	60				0.0%	0.0%
Qian 2022	0	81	0	81				0.0%	0.0%
Zhang 2022	0	30	0	30				0.0%	0.0%
Deng 2022	0	57	0	70				0.0%	0.0%
AlSmadi 2019	0	43	0	60				0.0%	0.0%
**Common effect model**		**1165**		**1146**				**100.0%**	**--**
**Random effects model**								**--**	**100.0%**

Heterogeneity: I^2 = NA%, τ^2 = NA, p = NA

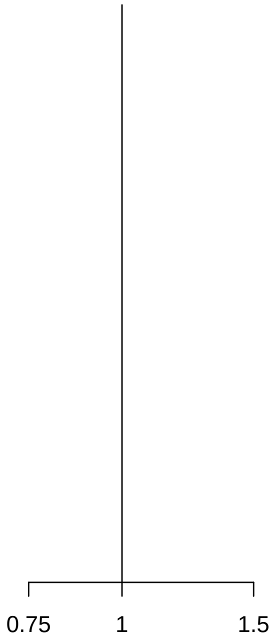

4.11.3 Trim and Fill

```
#trimfill(abscess_metabin)
#forest(trimfill(abscess_metabin),
#       sortvar = TE)
```

4.11.4 Funnel plot

```r
#funnel((abscess_metabin))
```

4.11.5 Baujat

```
#baujat(abscess_metabin, pos = 1)
```

4.11.6 Leave one out

```
#metainf(abscess_metabin)
```

4.12 Haematoma

4.12.1 Meta-analysis

```
haematoma_metabin <- metabin(data = suction_data,
                         event.c = hematoma_n_control,
                       n.c = sample_size_control,
                       event.e = hematoma_n_suction,
                       n.e = sample_size_suction,
                       studlab = author_year)
haematoma_metabin
```

```
## Number of studies: k = 1
## Number of observations: o = 2311
## Number of events: e = 1
##
##                          RR           95%-CI     z p-value
## Common effect model  0.4080 [0.0169; 9.8294] -0.55  0.5808
## Random effects model 0.4087 [0.0170; 9.8436] -0.55  0.5815
##
## Quantifying heterogeneity:
##  tau^2 = NA; tau = NA; I^2 = NA; H = NA
##
## Details on meta-analytical method:
## - Mantel-Haenszel method
## - Inverse variance method
## - Restricted maximum-likelihood estimator for tau^2
## - Continuity correction of 0.5 in studies with zero cell frequencies
```

4.12.2 Forest plot

```r
forest(haematoma_metabin,
       sortvar = TE)
```

Study	Experimental Events	Experimental Total	Control Events	Control Total	Risk Ratio	RR	95%−CI	Weight (common)	Weight (random)
Deng 2022	0	57	1	70		0.41	[0.02; 9.84]	100.0%	100.0%
Sur 2022	0	9	0	8				0.0%	0.0%
Tang 2023	0	86	0	87				0.0%	0.0%
Chen 2019	0	44	0	45				0.0%	0.0%
Du 2019	0	62	0	60				0.0%	0.0%
Lai 2020	0	28	0	56				0.0%	0.0%
Zhu 2018	0	165	0	165				0.0%	0.0%
Zhang 2021	0	56	0	50				0.0%	0.0%
Zhang 2021	0	56	0	54				0.0%	0.0%
Lechevallier 2003	.	11	.	14				0.0%	0.0%
Huang 2023	0	103	0	103				0.0%	0.0%
Wu 2022	0	76	0	82				0.0%	0.0%
Ding 2023	0	138	0	61				0.0%	0.0%
Zhai 2023	0	60	0	60				0.0%	0.0%
Zhai 2023	0	60	0	60				0.0%	0.0%
Qian 2022	0	81	0	81				0.0%	0.0%
Zhang 2022	0	30	0	30				0.0%	0.0%
AlSmadi 2019	0	43	0	60				0.0%	0.0%
**Common effect model**		**1165**		**1146**		**0.41**	**[0.02; 9.83]**	**100.0%**	**−−**
**Random effects model**						**0.41**	**[0.02; 9.84]**	**−−**	**100.0%**

Heterogeneity: I^2 = NA%, τ^2 = NA, p = NA

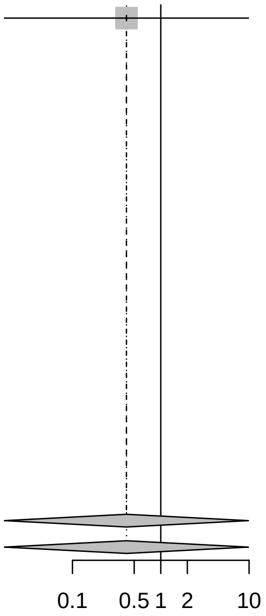

4.12.3 Trim and Fill

```
#trimfill(haematoma_metabin)
#forest(trimfill(haematoma_metabin),
#       sortvar = TE)
```

4.12.4 Funnel plot

```
funnel((haematoma_metabin))
```

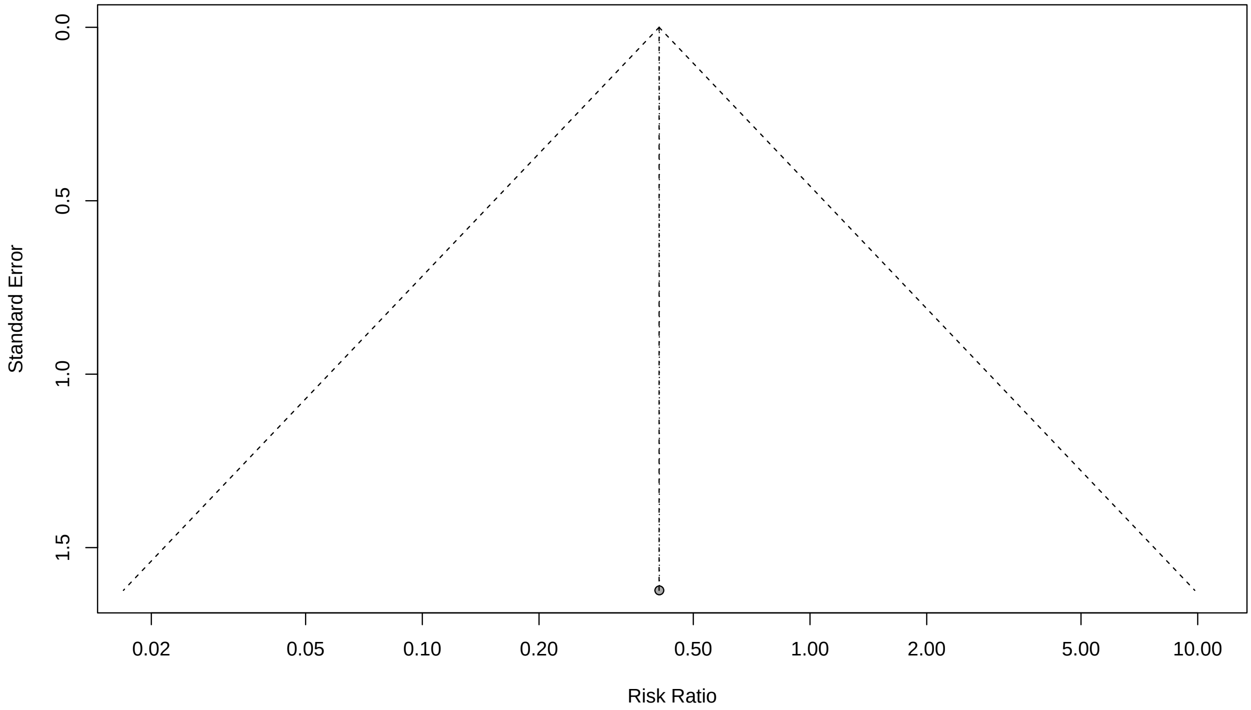

4.12.5 Baujat

```r
baujat(haematoma_metabin, pos = 1)
```

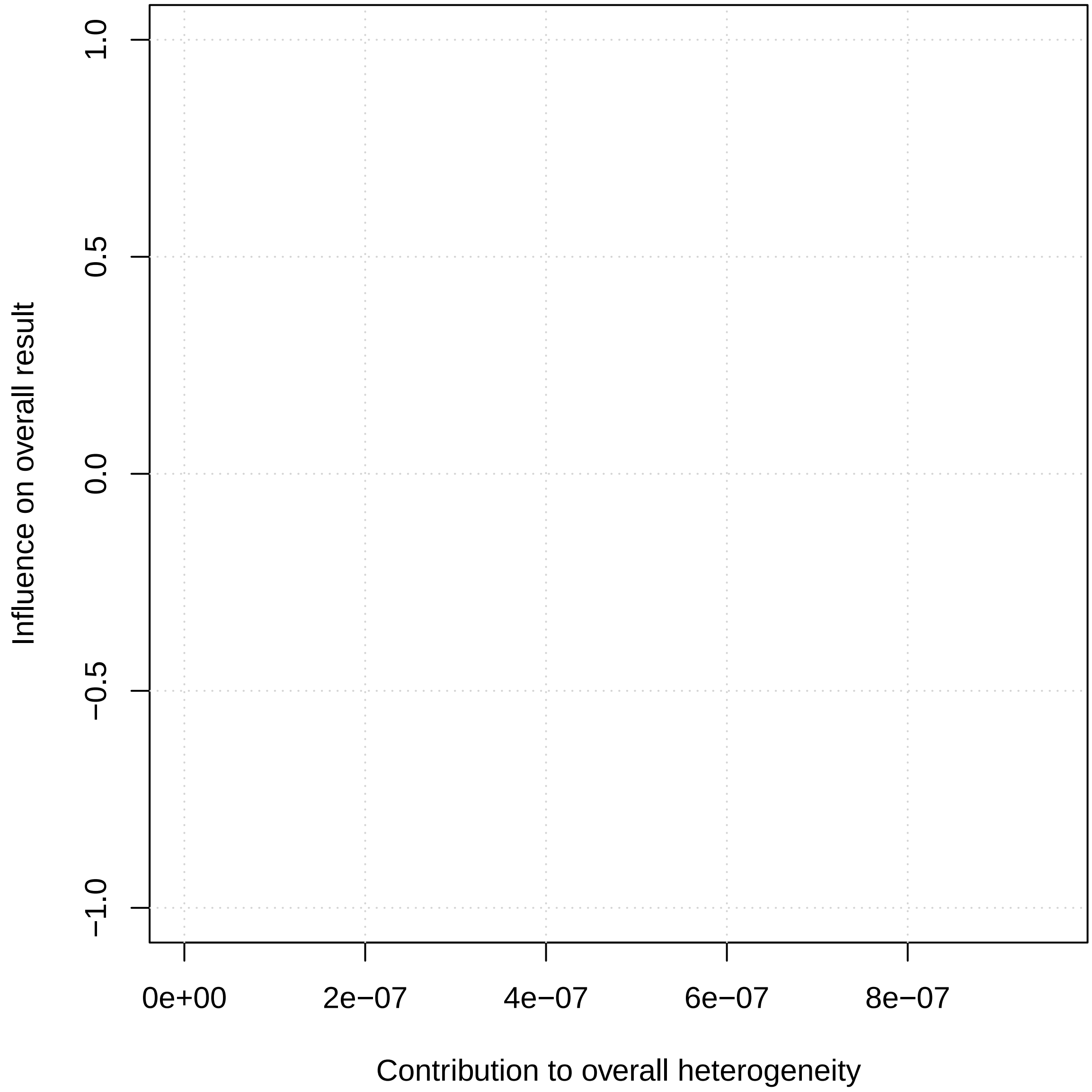

4.12.6 Leave one out

```
metainf(haematoma_metabin)
```

```
## Influential analysis (common effect model)
##
##                                  RR           95%-CI p-value tau^2 tau I^2
## Omitting Sur 2022            0.4080 [0.0169; 9.8294]  0.5808
## Omitting Tang 2023           0.4080 [0.0169; 9.8294]  0.5808
## Omitting Chen 2019           0.4080 [0.0169; 9.8294]  0.5808
## Omitting Du 2019             0.4080 [0.0169; 9.8294]  0.5808
## Omitting Lai 2020            0.4080 [0.0169; 9.8294]  0.5808
## Omitting Zhu 2018            0.4080 [0.0169; 9.8294]  0.5808
## Omitting Zhang 2021          0.4080 [0.0169; 9.8294]  0.5808
## Omitting Zhang 2021          0.4080 [0.0169; 9.8294]  0.5808
## Omitting Lechevallier 2003   0.4080 [0.0169; 9.8294]  0.5808
## Omitting Huang 2023          0.4080 [0.0169; 9.8294]  0.5808
## Omitting Wu 2022             0.4080 [0.0169; 9.8294]  0.5808
## Omitting Ding 2023           0.4080 [0.0169; 9.8294]  0.5808
## Omitting Zhai 2023           0.4080 [0.0169; 9.8294]  0.5808
## Omitting Zhai 2023           0.4080 [0.0169; 9.8294]  0.5808
## Omitting Qian 2022           0.4080 [0.0169; 9.8294]  0.5808
## Omitting Zhang 2022          0.4080 [0.0169; 9.8294]  0.5808
## Omitting Deng 2022
## Omitting AlSmadi 2019        0.4080 [0.0169; 9.8294]  0.5808
##
## Pooled estimate              0.4080 [0.0169; 9.8294]  0.5808
##
## Details on meta-analytical method:
## - Mantel-Haenszel method
## - Restricted maximum-likelihood estimator for tau^2
```

4.13 Pain

4.13.1 Meta-analysis

```
pain_metabin <- metabin(data = suction_data,
                          event.c = pain_n_control,
                        n.c = sample_size_control,
                        event.e = pain_n_suction,
                        n.e = sample_size_suction,
                        studlab = author_year)
pain_metabin
```

```
## Number of studies: k = 3
## Number of observations: o = 2311
## Number of events: e = 25
##
##                           RR           95%-CI     z p-value
## Common effect model  0.2103 [0.0780; 0.5670] -3.08  0.0021
## Random effects model 0.2180 [0.0804; 0.5913] -2.99  0.0028
##
## Quantifying heterogeneity:
##  tau^2 = 0 [0.0000; 4.5123]; tau = 0 [0.0000; 2.1242]
##  I^2 = 0.0% [0.0%; 89.6%]; H = 1.00 [1.00; 3.10]
##
## Test of heterogeneity:
##     Q d.f. p-value
##  0.39    2  0.8246
##
## Details on meta-analytical method:
## - Mantel-Haenszel method
## - Inverse variance method
## - Restricted maximum-likelihood estimator for tau^2
## - Q-Profile method for confidence interval of tau^2 and tau
## - Continuity correction of 0.5 in studies with zero cell frequencies
```

4.13.2 Forest plot

```r
forest(pain_metabin,
       sortvar = TE)
```

Study	Experimental Events	Experimental Total	Control Events	Control Total	RR	95%−CI	Weight (common)	Weight (random)
Tang 2023	2	86	13	87	0.16	[0.04; 0.67]	60.3%	46.8%
Chen 2019	2	44	7	45	0.29	[0.06; 1.33]	32.3%	43.3%
Zhang 2021	0	56	1	50	0.30	[0.01; 7.15]	7.4%	9.9%
Sur 2022	.	9	.	8			0.0%	0.0%
Du 2019	.	62	.	60			0.0%	0.0%
Lai 2020	.	28	.	56			0.0%	0.0%
Zhu 2018	.	165	.	165			0.0%	0.0%
Zhang 2021	0	56	0	54			0.0%	0.0%
Lechevallier 2003	.	11	.	14			0.0%	0.0%
Huang 2023	.	103	.	103			0.0%	0.0%
Wu 2022	.	76	.	82			0.0%	0.0%
Ding 2023	.	138	.	61			0.0%	0.0%
Zhai 2023	.	60	.	60			0.0%	0.0%
Zhai 2023	.	60	.	60			0.0%	0.0%
Qian 2022	.	81	.	81			0.0%	0.0%
Zhang 2022	.	30	.	30			0.0%	0.0%
Deng 2022	.	57	.	70			0.0%	0.0%
AlSmadi 2019	0	43	0	60			0.0%	0.0%
**Common effect model**		**1165**		**1146**	**0.21**	**[0.08; 0.57]**	**100.0%**	**--**
**Random effects model**					**0.22**	**[0.08; 0.59]**	**--**	**100.0%**

Heterogeneity: $I^2 = 0\%$, $\tau^2 = 0$, $p = 0.82$

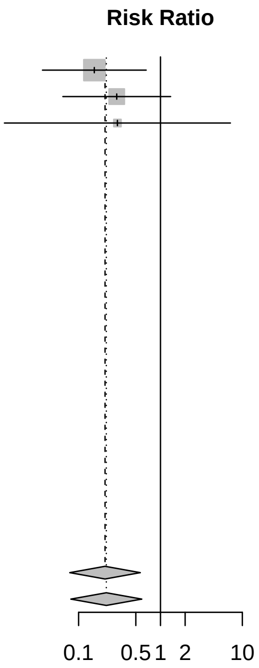

4.13.3 Trim and Fill

```r
trimfill(pain_metabin)
```

```
## Warning in trimfill.meta(pain_metabin): 15 observation(s) dropped due to
## missing values
```

```
## Number of studies: k = 3 (with 0 added studies)
## Number of observations: o = 368
## Number of events: e = 25
##
##                          RR           95%-CI     z p-value
## Random effects model 0.2180 [0.0804; 0.5913] -2.99  0.0028
##
## Quantifying heterogeneity:
##  tau^2 = 0 [0.0000; 4.5123]; tau = 0 [0.0000; 2.1242]
##  I^2 = 0.0% [0.0%; 89.6%]; H = 1.00 [1.00; 3.10]
##
## Test of heterogeneity:
##     Q d.f. p-value
##  0.39    2  0.8246
##
## Details on meta-analytical method:
## - Inverse variance method
## - Restricted maximum-likelihood estimator for tau^2
## - Q-Profile method for confidence interval of tau^2 and tau
## - Trim-and-fill method to adjust for funnel plot asymmetry (L-estimator)
```

```r
forest(trimfill(pain_metabin),
       sortvar = TE)
```

```
## Warning in trimfill.meta(pain_metabin): 15 observation(s) dropped due to
## missing values
```

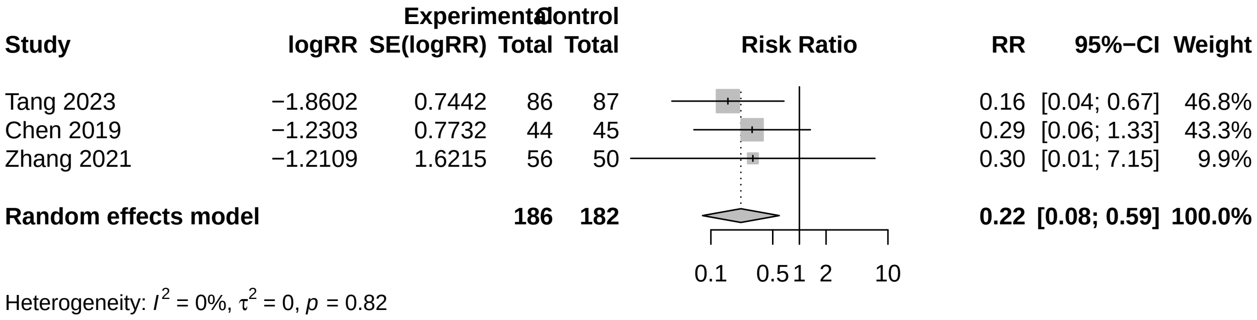

4.13.4 Funnel plot

```
funnel(trimfill(pain_metabin))
```

```
## Warning in trimfill.meta(pain_metabin): 15 observation(s) dropped due to
## missing values
```

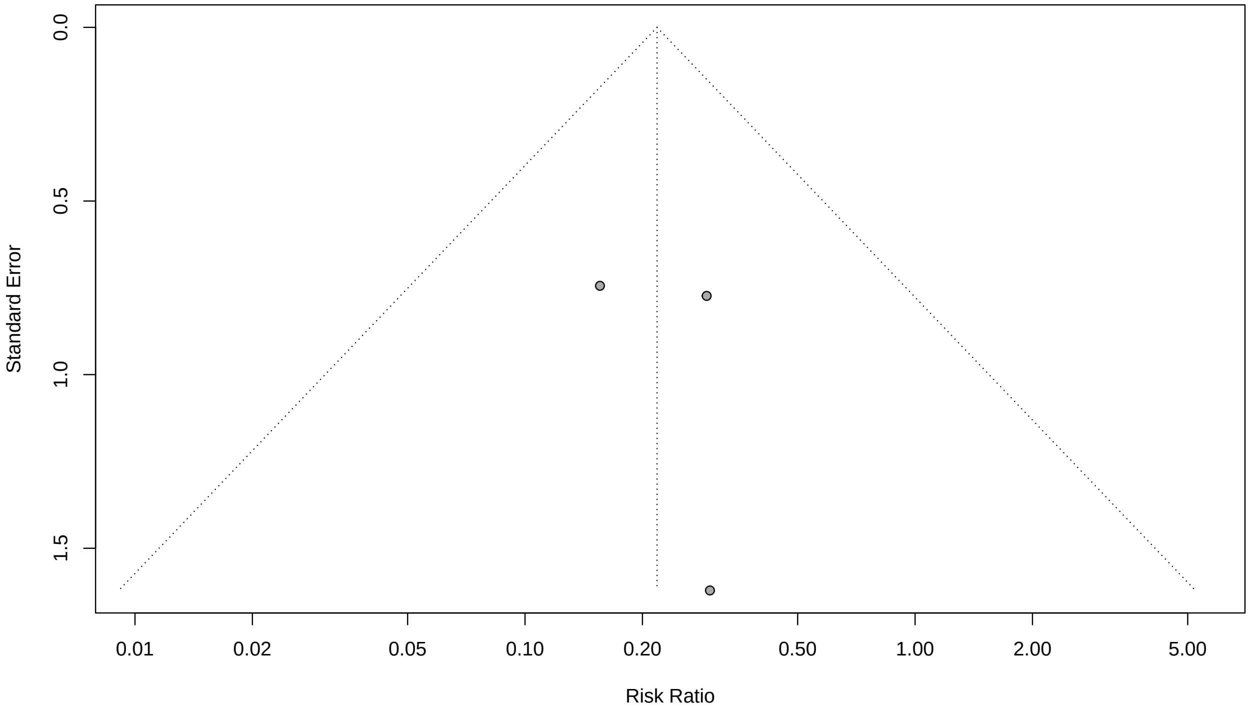

4.13.5 Baujat

```
baujat(pain_metabin, pos = 1)
```

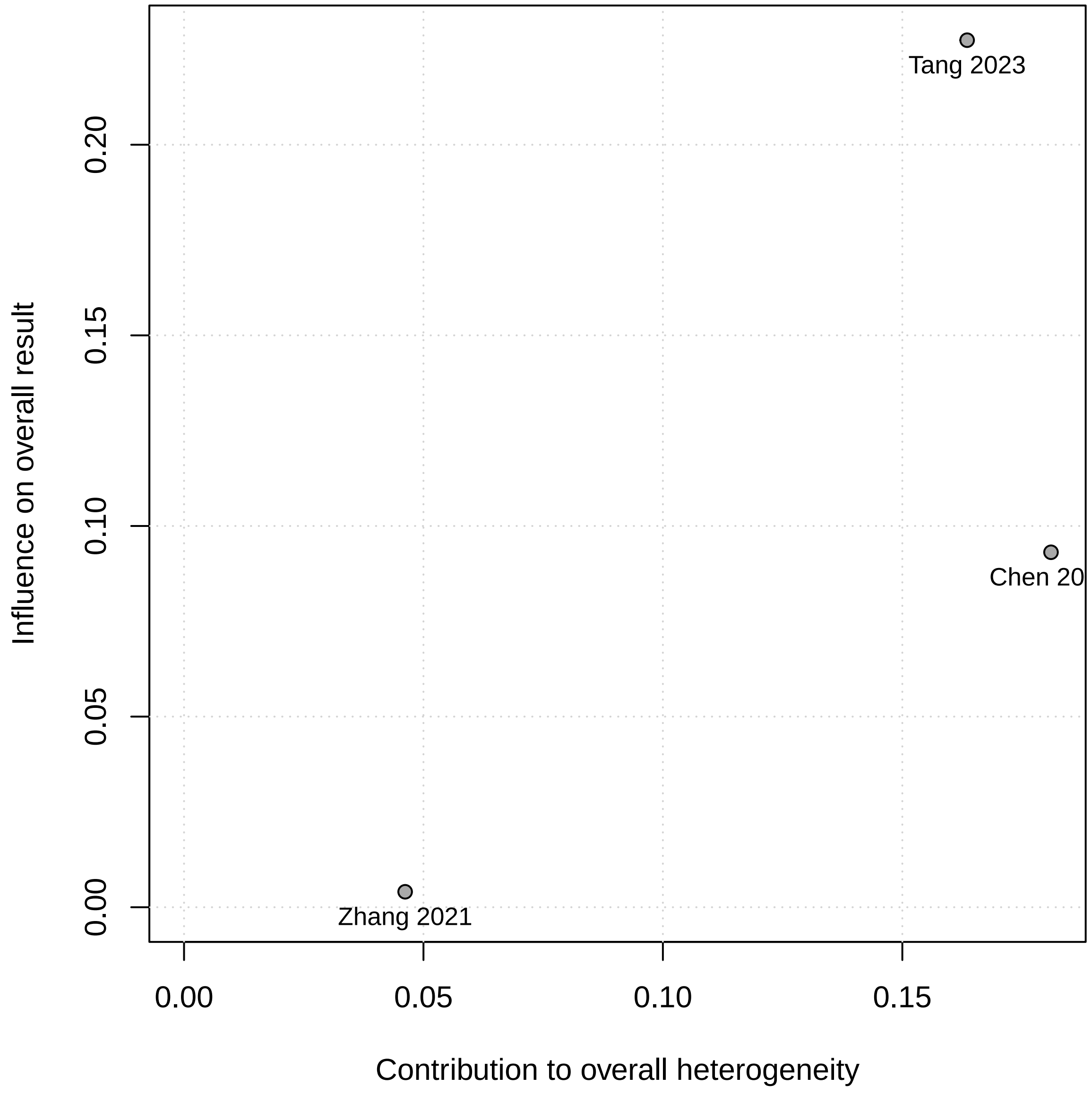

4.13.6 Leave one out

```
metainf(pain_metabin, pos = 1)
```

```
## Influential analysis (common effect model)
##
##                                  RR           95%-CI p-value   tau^2    tau
## Omitting Sur 2022            0.2103 [0.0780; 0.5670]  0.0021  0.0000  0.0000
## Omitting Tang 2023           0.2933 [0.0747; 1.1520]  0.0789  0.0000  0.0000
## Omitting Chen 2019           0.1712 [0.0457; 0.6414]  0.0088  0.0000  0.0000
## Omitting Du 2019             0.2103 [0.0780; 0.5670]  0.0021  0.0000  0.0000
## Omitting Lai 2020            0.2103 [0.0780; 0.5670]  0.0021  0.0000  0.0000
## Omitting Zhu 2018            0.2103 [0.0780; 0.5670]  0.0021  0.0000  0.0000
## Omitting Zhang 2021          0.2033 [0.0715; 0.5781]  0.0028  0.0000  0.0000
## Omitting Zhang 2021          0.2103 [0.0780; 0.5670]  0.0021  0.0000  0.0000
## Omitting Lechevallier 2003   0.2103 [0.0780; 0.5670]  0.0021  0.0000  0.0000
## Omitting Huang 2023          0.2103 [0.0780; 0.5670]  0.0021  0.0000  0.0000
## Omitting Wu 2022             0.2103 [0.0780; 0.5670]  0.0021  0.0000  0.0000
## Omitting Ding 2023           0.2103 [0.0780; 0.5670]  0.0021  0.0000  0.0000
## Omitting Zhai 2023           0.2103 [0.0780; 0.5670]  0.0021  0.0000  0.0000
## Omitting Zhai 2023           0.2103 [0.0780; 0.5670]  0.0021  0.0000  0.0000
## Omitting Qian 2022           0.2103 [0.0780; 0.5670]  0.0021  0.0000  0.0000
## Omitting Zhang 2022          0.2103 [0.0780; 0.5670]  0.0021  0.0000  0.0000
## Omitting Deng 2022           0.2103 [0.0780; 0.5670]  0.0021  0.0000  0.0000
## Omitting AlSmadi 2019        0.2103 [0.0780; 0.5670]  0.0021  0.0000  0.0000
##
## Pooled estimate              0.2103 [0.0780; 0.5670]  0.0021  0.0000  0.0000
##                                I^2
## Omitting Sur 2022             0.0%
## Omitting Tang 2023            0.0%
## Omitting Chen 2019            0.0%
## Omitting Du 2019              0.0%
## Omitting Lai 2020             0.0%
## Omitting Zhu 2018             0.0%
## Omitting Zhang 2021           0.0%
## Omitting Zhang 2021           0.0%
## Omitting Lechevallier 2003    0.0%
## Omitting Huang 2023           0.0%
## Omitting Wu 2022              0.0%
## Omitting Ding 2023            0.0%
## Omitting Zhai 2023            0.0%
## Omitting Zhai 2023            0.0%
## Omitting Qian 2022            0.0%
## Omitting Zhang 2022           0.0%
## Omitting Deng 2022            0.0%
## Omitting AlSmadi 2019         0.0%
##
## Pooled estimate               0.0%
##
## Details on meta-analytical method:
## - Mantel-Haenszel method
## - Restricted maximum-likelihood estimator for tau^2
```

4.14 Stricture

4.14.1 Meta-analysis

```
stricture_metabin <- metabin(data = suction_data,
                          event.c = stricture_n_control,
                        n.c = sample_size_control,
                        event.e = stricture_n_suction,
                        n.e = sample_size_suction,
                        studlab = author_year)
stricture_metabin
```

```
## Number of studies: k = 2
## Number of observations: o = 2311
## Number of events: e = 4
##
##                          RR            95%-CI     z p-value
## Common effect model  0.4911 [0.0916;  2.6336] -0.83  0.4066
## Random effects model 0.6034 [0.0297; 12.2755] -0.33  0.7424
##
## Quantifying heterogeneity:
##  tau^2 = 2.2802; tau = 1.5100; I^2 = 48.2%; H = 1.39
##
## Test of heterogeneity:
##     Q d.f. p-value
##  1.93    1  0.1648
##
## Details on meta-analytical method:
## - Mantel-Haenszel method
## - Inverse variance method
## - Restricted maximum-likelihood estimator for tau^2
## - Continuity correction of 0.5 in studies with zero cell frequencies
```

4.14.2 Forest plot

```r
forest(stricture_metabin,
       sortvar = TE)
```

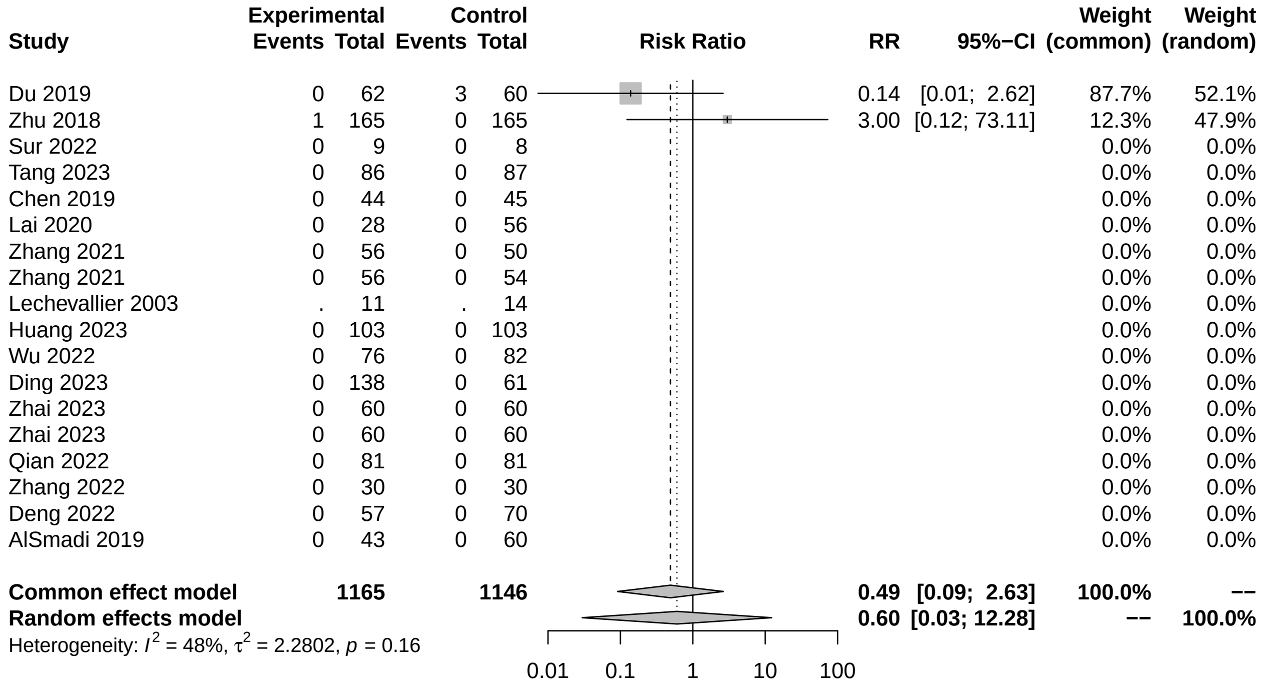

4.14.3 Trim and Fill

```
#trimfill(stricture_metabin)
#forest(trimfill(stricture_metabin),
#       sortvar = TE)
```

4.14.4 Funnel plot

```
funnel((stricture_metabin))
```

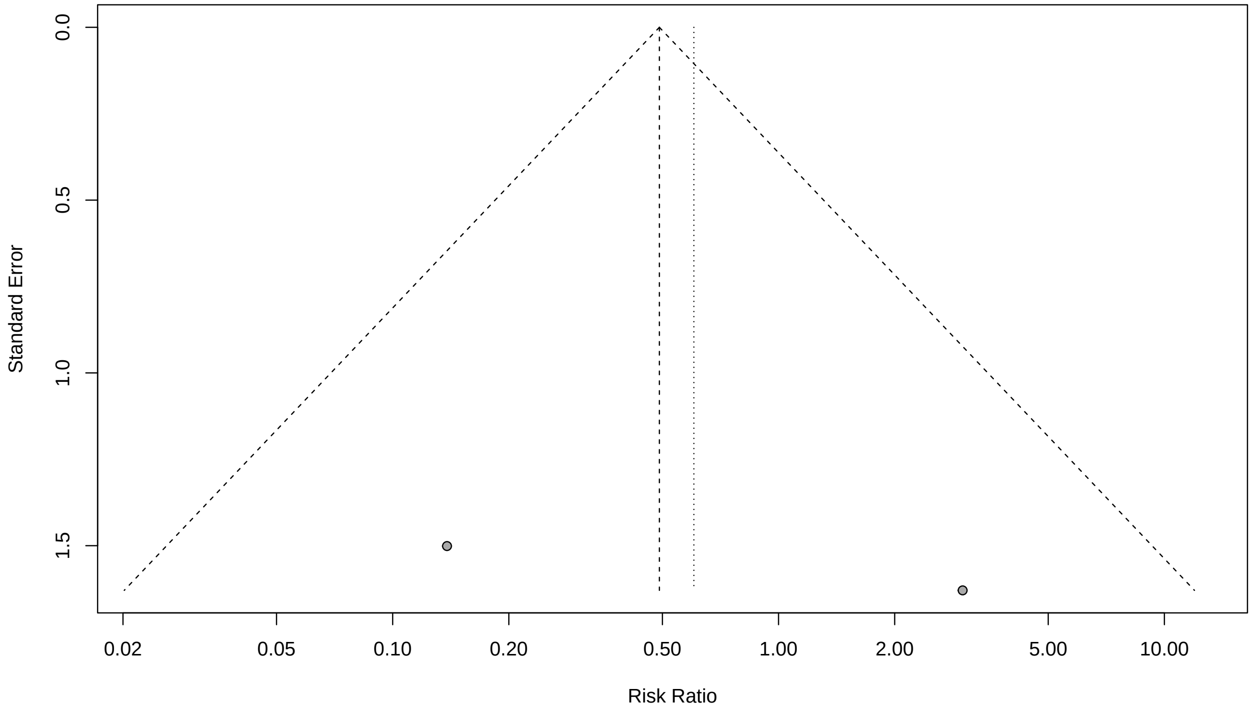

4.14.5 Baujat

```
baujat(stricture_metabin, pos = 1)
```

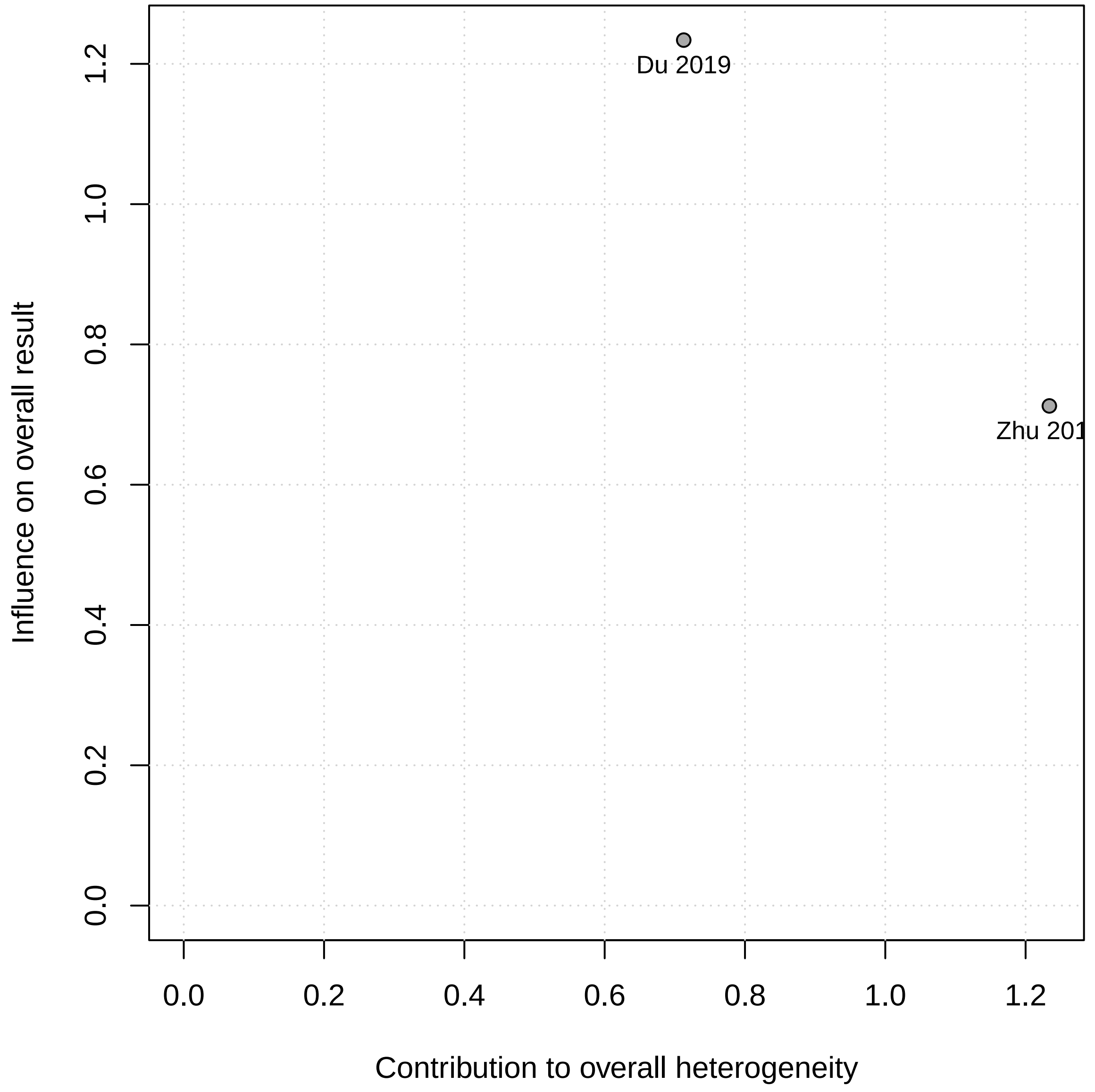

4.14.6 Leave one out

```
metainf(stricture_metabin)
```

```
## Influential analysis (common effect model)
##
##                                   RR            95%-CI p-value   tau^2     tau
## Omitting Sur 2022             0.4911 [0.0916;  2.6336]  0.4066  2.2802  1.5100
## Omitting Tang 2023            0.4911 [0.0916;  2.6336]  0.4066  2.2802  1.5100
## Omitting Chen 2019            0.4911 [0.0916;  2.6336]  0.4066  2.2802  1.5100
## Omitting Du 2019              3.0000 [0.1231; 73.1112]  0.5001
## Omitting Lai 2020             0.4911 [0.0916;  2.6336]  0.4066  2.2802  1.5100
## Omitting Zhu 2018             0.1383 [0.0073;  2.6222]  0.1876
## Omitting Zhang 2021           0.4911 [0.0916;  2.6336]  0.4066  2.2802  1.5100
## Omitting Zhang 2021           0.4911 [0.0916;  2.6336]  0.4066  2.2802  1.5100
## Omitting Lechevallier 2003    0.4911 [0.0916;  2.6336]  0.4066  2.2802  1.5100
## Omitting Huang 2023           0.4911 [0.0916;  2.6336]  0.4066  2.2802  1.5100
## Omitting Wu 2022              0.4911 [0.0916;  2.6336]  0.4066  2.2802  1.5100
## Omitting Ding 2023            0.4911 [0.0916;  2.6336]  0.4066  2.2802  1.5100
## Omitting Zhai 2023            0.4911 [0.0916;  2.6336]  0.4066  2.2802  1.5100
## Omitting Zhai 2023            0.4911 [0.0916;  2.6336]  0.4066  2.2802  1.5100
## Omitting Qian 2022            0.4911 [0.0916;  2.6336]  0.4066  2.2802  1.5100
## Omitting Zhang 2022           0.4911 [0.0916;  2.6336]  0.4066  2.2802  1.5100
## Omitting Deng 2022            0.4911 [0.0916;  2.6336]  0.4066  2.2802  1.5100
## Omitting AlSmadi 2019         0.4911 [0.0916;  2.6336]  0.4066  2.2802  1.5100
##
## Pooled estimate               0.4911 [0.0916;  2.6336]  0.4066  2.2802  1.5100
##                                  I^2
## Omitting Sur 2022              48.2%
## Omitting Tang 2023             48.2%
## Omitting Chen 2019             48.2%
## Omitting Du 2019
## Omitting Lai 2020              48.2%
## Omitting Zhu 2018
## Omitting Zhang 2021            48.2%
## Omitting Zhang 2021            48.2%
## Omitting Lechevallier 2003     48.2%
## Omitting Huang 2023            48.2%
## Omitting Wu 2022               48.2%
## Omitting Ding 2023             48.2%
## Omitting Zhai 2023             48.2%
## Omitting Zhai 2023             48.2%
## Omitting Qian 2022             48.2%
## Omitting Zhang 2022            48.2%
## Omitting Deng 2022             48.2%
## Omitting AlSmadi 2019          48.2%
##
## Pooled estimate                48.2%
##
## Details on meta-analytical method:
## - Mantel-Haenszel method
## - Restricted maximum-likelihood estimator for tau^2
```

4.15 Embolisation required

4.15.1 Meta-analysis

```
ir_embolisation_metabin <- metabin(data = suction_data,
                          event.c = embolism_ir_intervention_n_control,
                        n.c = sample_size_control,
                        event.e = embolism_ir_intervention_n_suction,
                        n.e = sample_size_suction,
                        studlab = author_year)
ir_embolisation_metabin
```

```
## Number of studies: k = 2
## Number of observations: o = 2311
## Number of events: e = 3
##
##                          RR           95%-CI     z p-value
## Common effect model  0.2830 [0.0318; 2.5161] -1.13  0.2575
## Random effects model 0.2867 [0.0322; 2.5524] -1.12  0.2627
##
## Quantifying heterogeneity:
##  tau^2 = 0; tau = 0; I^2 = 0.0%; H = 1.00
##
## Test of heterogeneity:
##     Q d.f. p-value
##  0.02    1  0.8828
##
## Details on meta-analytical method:
## - Mantel-Haenszel method
## - Inverse variance method
## - Restricted maximum-likelihood estimator for tau^2
## - Continuity correction of 0.5 in studies with zero cell frequencies
```

4.15.2 Forest plot

```r
forest(ir_embolisation_metabin,
       sortvar = TE)
```

Study	Experimental Events	Experimental Total	Control Events	Control Total	RR	95%−CI	Weight (common)	Weight (random)
Deng 2022	0	57	2	70	0.25	[0.01; 5.01]	60.2%	52.5%
Chen 2019	0	44	1	45	0.34	[0.01; 8.15]	39.8%	47.5%
Sur 2022	0	9	0	8			0.0%	0.0%
Tang 2023	0	86	0	87			0.0%	0.0%
Du 2019	0	62	0	60			0.0%	0.0%
Lai 2020	0	28	0	56			0.0%	0.0%
Zhu 2018	0	165	0	165			0.0%	0.0%
Zhang 2021	0	56	0	50			0.0%	0.0%
Zhang 2021	0	56	0	54			0.0%	0.0%
Lechevallier 2003	.	11	.	14			0.0%	0.0%
Huang 2023	0	103	0	103			0.0%	0.0%
Wu 2022	0	76	0	82			0.0%	0.0%
Ding 2023	0	138	0	61			0.0%	0.0%
Zhai 2023	0	60	0	60			0.0%	0.0%
Zhai 2023	0	60	0	60			0.0%	0.0%
Qian 2022	0	81	0	81			0.0%	0.0%
Zhang 2022	0	30	0	30			0.0%	0.0%
AlSmadi 2019	0	43	0	60			0.0%	0.0%
**Common effect model**		**1165**		**1146**	**0.28**	**[0.03; 2.52]**	**100.0%**	**--**
**Random effects model**					**0.29**	**[0.03; 2.55]**	**--**	**100.0%**

Heterogeneity: $I^2 = 0\%$, $\tau^2 = 0$, $p = 0.88$

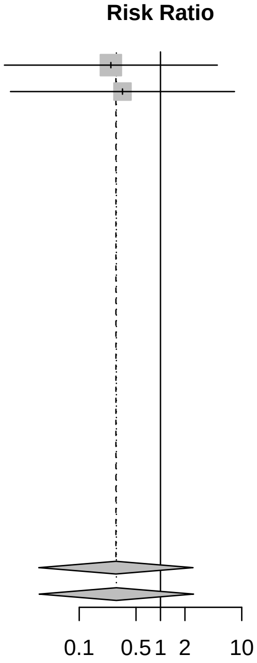

4.15.3 Trim and Fill

```
#trimfill(ir_embolisation_metabin)
#forest(trimfill(ir_embolisation_metabin),
#       sortvar = TE)
```

4.15.4 Funnel plot

```
funnel((ir_embolisation_metabin))
```

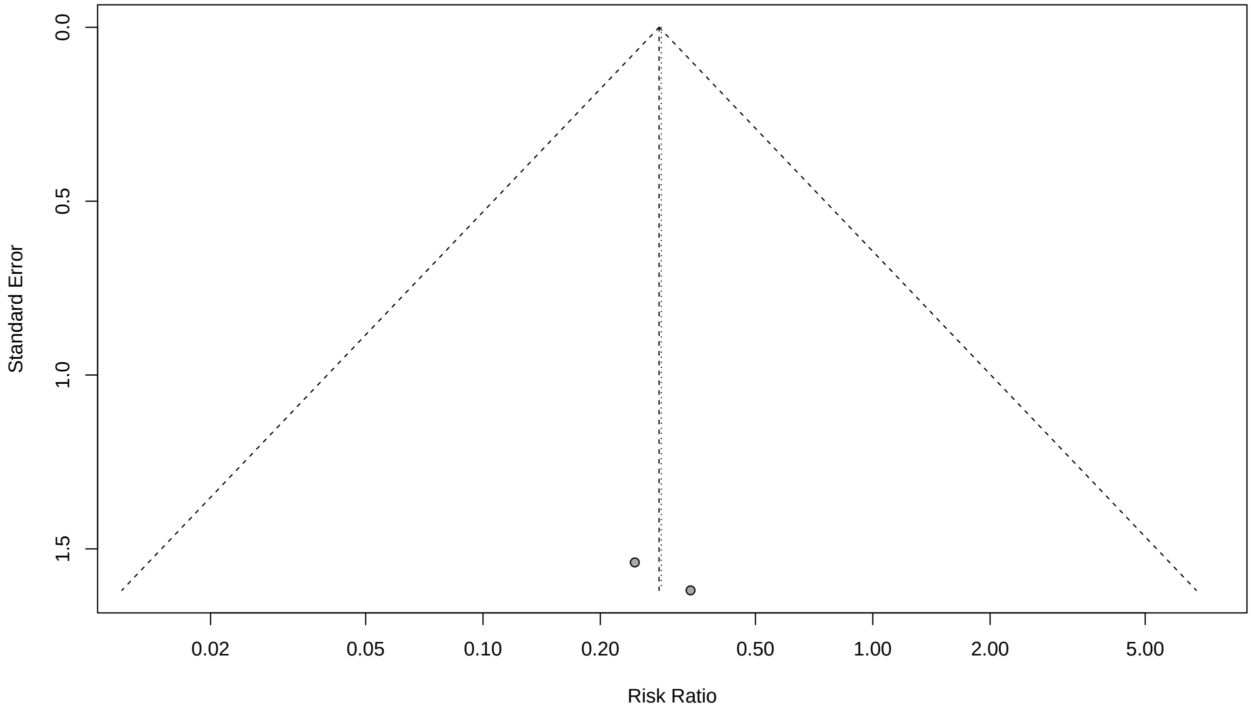

4.15.5 Baujat

```r
baujat(ir_embolisation_metabin, pos = 1)
```

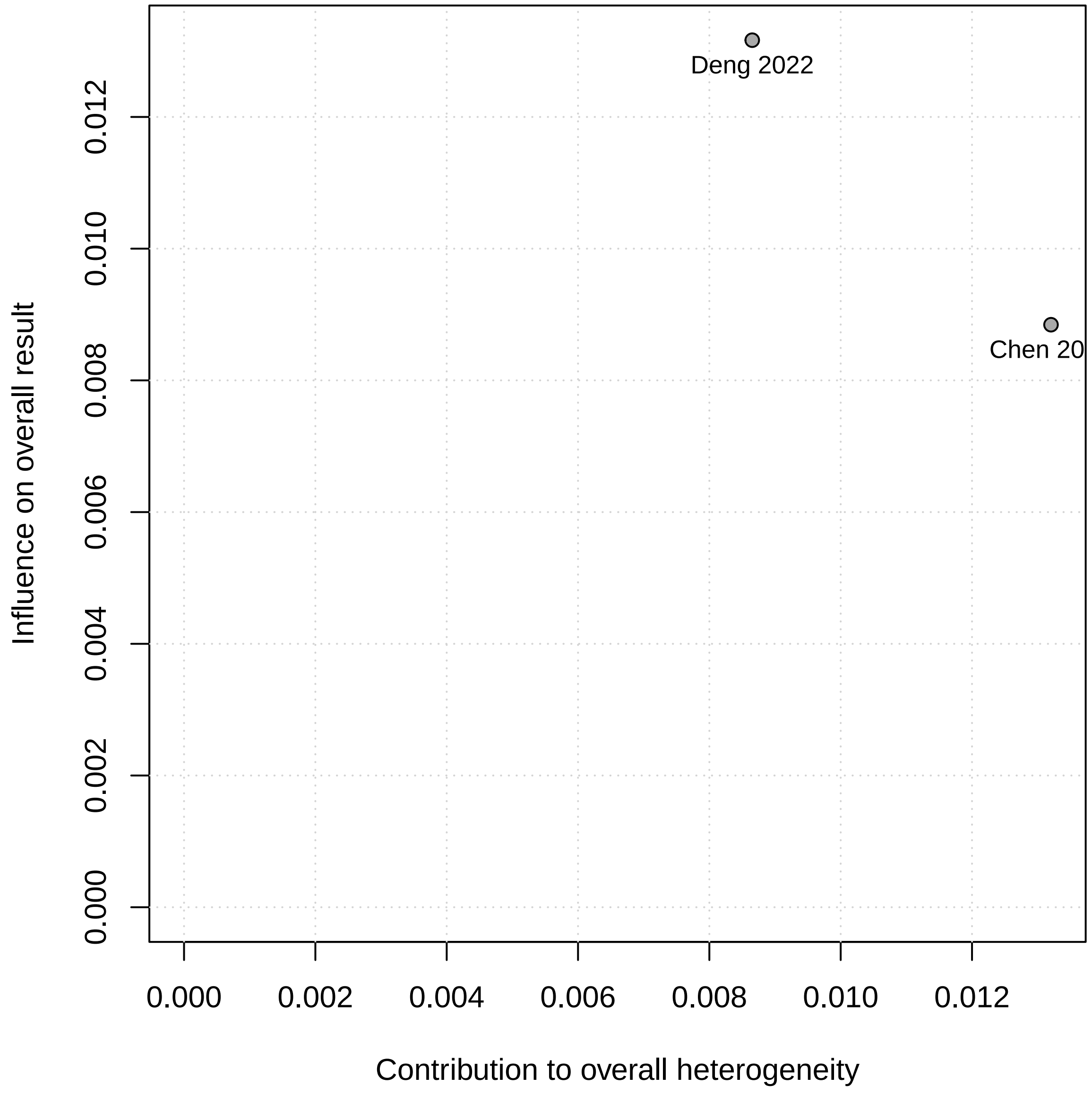

4.15.6 Leave one out

```
metainf(ir_embolisation_metabin)
```

```
## Influential analysis (common effect model)
##
##                                  RR           95%-CI p-value   tau^2     tau
## Omitting Sur 2022            0.2830 [0.0318; 2.5161]  0.2575  0.0000  0.0000
## Omitting Tang 2023           0.2830 [0.0318; 2.5161]  0.2575  0.0000  0.0000
## Omitting Chen 2019           0.2448 [0.0120; 4.9992]  0.3605
## Omitting Du 2019             0.2830 [0.0318; 2.5161]  0.2575  0.0000  0.0000
## Omitting Lai 2020            0.2830 [0.0318; 2.5161]  0.2575  0.0000  0.0000
## Omitting Zhu 2018            0.2830 [0.0318; 2.5161]  0.2575  0.0000  0.0000
## Omitting Zhang 2021          0.2830 [0.0318; 2.5161]  0.2575  0.0000  0.0000
## Omitting Zhang 2021          0.2830 [0.0318; 2.5161]  0.2575  0.0000  0.0000
## Omitting Lechevallier 2003   0.2830 [0.0318; 2.5161]  0.2575  0.0000  0.0000
## Omitting Huang 2023          0.2830 [0.0318; 2.5161]  0.2575  0.0000  0.0000
## Omitting Wu 2022             0.2830 [0.0318; 2.5161]  0.2575  0.0000  0.0000
## Omitting Ding 2023           0.2830 [0.0318; 2.5161]  0.2575  0.0000  0.0000
## Omitting Zhai 2023           0.2830 [0.0318; 2.5161]  0.2575  0.0000  0.0000
## Omitting Zhai 2023           0.2830 [0.0318; 2.5161]  0.2575  0.0000  0.0000
## Omitting Qian 2022           0.2830 [0.0318; 2.5161]  0.2575  0.0000  0.0000
## Omitting Zhang 2022          0.2830 [0.0318; 2.5161]  0.2575  0.0000  0.0000
## Omitting Deng 2022           0.3407 [0.0143; 8.1456]  0.5062
## Omitting AlSmadi 2019        0.2830 [0.0318; 2.5161]  0.2575  0.0000  0.0000
##
## Pooled estimate              0.2830 [0.0318; 2.5161]  0.2575  0.0000  0.0000
##                                 I^2
## Omitting Sur 2022              0.0%
## Omitting Tang 2023             0.0%
## Omitting Chen 2019
## Omitting Du 2019               0.0%
## Omitting Lai 2020              0.0%
## Omitting Zhu 2018              0.0%
## Omitting Zhang 2021            0.0%
## Omitting Zhang 2021            0.0%
## Omitting Lechevallier 2003     0.0%
## Omitting Huang 2023            0.0%
## Omitting Wu 2022               0.0%
## Omitting Ding 2023             0.0%
## Omitting Zhai 2023             0.0%
## Omitting Zhai 2023             0.0%
## Omitting Qian 2022             0.0%
## Omitting Zhang 2022            0.0%
## Omitting Deng 2022
## Omitting AlSmadi 2019          0.0%
##
## Pooled estimate                0.0%
##
## Details on meta-analytical method:
## - Mantel-Haenszel method
## - Restricted maximum-likelihood estimator for tau^2
```

4.16 Transfusion

4.16.1 Meta-analysis

```
transfusion_metabin <- metabin(data = suction_data,
                          event.c = transfusion_n_control,
                        n.c = sample_size_control,
                        event.e = transfusion_n_suction,
                        n.e = sample_size_suction,
                        studlab = author_year)
transfusion_metabin
```

```
## Number of studies: k = 3
## Number of observations: o = 2311
## Number of events: e = 12
##
##                             RR           95%-CI     z p-value
## Common effect model  0.1445 [0.0280; 0.7467] -2.31  0.0210
## Random effects model 0.1632 [0.0302; 0.8826] -2.10  0.0353
##
## Quantifying heterogeneity:
##  tau^2 = 0 [0.0000; 22.9527]; tau = 0 [0.0000; 4.7909]
##  I^2 = 0.0% [0.0%; 89.6%]; H = 1.00 [1.00; 3.10]
##
## Test of heterogeneity:
##     Q d.f. p-value
##  0.57    2  0.7525
##
## Details on meta-analytical method:
## - Mantel-Haenszel method
## - Inverse variance method
## - Restricted maximum-likelihood estimator for tau^2
## - Q-Profile method for confidence interval of tau^2 and tau
## - Continuity correction of 0.5 in studies with zero cell frequencies
```

4.16.2 Forest plot

```r
forest(transfusion_metabin,
       sortvar = TE)
```

Study	Experimental Events	Experimental Total	Control Events	Control Total	RR	95%−CI	Weight (common)	Weight (random)
Deng 2022	0	57	7	70	0.08	[0.00; 1.40]	56.7%	35.3%
Chen 2019	0	44	3	45	0.15	[0.01; 2.75]	29.1%	33.1%
Lai 2020	0	28	2	56	0.40	[0.02; 7.99]	14.2%	31.6%
Sur 2022	0	9	0	8			0.0%	0.0%
Tang 2023	0	86	0	87			0.0%	0.0%
Du 2019	0	62	0	60			0.0%	0.0%
Zhu 2018	0	165	0	165			0.0%	0.0%
Zhang 2021	0	56	0	50			0.0%	0.0%
Zhang 2021	0	56	0	54			0.0%	0.0%
Lechevallier 2003	.	11	.	14			0.0%	0.0%
Huang 2023	0	103	0	103			0.0%	0.0%
Wu 2022	0	76	0	82			0.0%	0.0%
Ding 2023	0	138	0	61			0.0%	0.0%
Zhai 2023	0	60	0	60			0.0%	0.0%
Zhai 2023	0	60	0	60			0.0%	0.0%
Qian 2022	0	81	0	81			0.0%	0.0%
Zhang 2022	0	30	0	30			0.0%	0.0%
AlSmadi 2019	0	43	0	60			0.0%	0.0%
**Common effect model**		**1165**		**1146**	**0.14**	**[0.03; 0.75]**	**100.0%**	**--**
**Random effects model**					**0.16**	**[0.03; 0.88]**	**--**	**100.0%**

Heterogeneity: $I^2 = 0\%$, $\tau^2 = 0$, $p = 0.75$

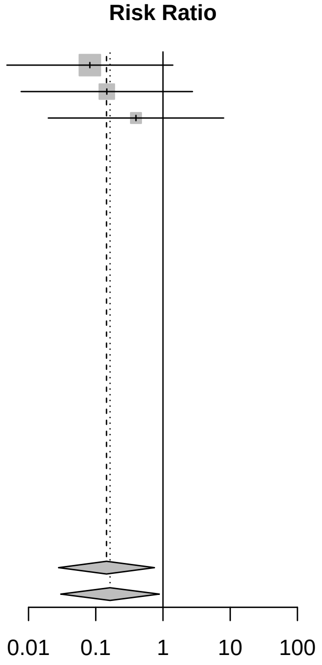

4.16.3 Trim and Fill

```
trimfill(transfusion_metabin)
```

```
## Warning in trimfill.meta(transfusion_metabin): 15 observation(s) dropped due to
## missing values

## Number of studies: k = 3 (with 0 added studies)
## Number of observations: o = 300
## Number of events: e = 12
##
##                          RR           95%-CI     z p-value
## Random effects model 0.1632 [0.0302; 0.8826] -2.10  0.0353
##
## Quantifying heterogeneity:
##  tau^2 = 0 [0.0000; 22.9527]; tau = 0 [0.0000; 4.7909]
##  I^2 = 0.0% [0.0%; 89.6%]; H = 1.00 [1.00; 3.10]
##
## Test of heterogeneity:
##     Q d.f. p-value
##  0.57    2  0.7525
##
## Details on meta-analytical method:
## - Inverse variance method
## - Restricted maximum-likelihood estimator for tau^2
## - Q-Profile method for confidence interval of tau^2 and tau
## - Trim-and-fill method to adjust for funnel plot asymmetry (L-estimator)
```

```
forest(trimfill(transfusion_metabin),
       sortvar = TE)
```

```
## Warning in trimfill.meta(transfusion_metabin): 15 observation(s) dropped due to
## missing values
```

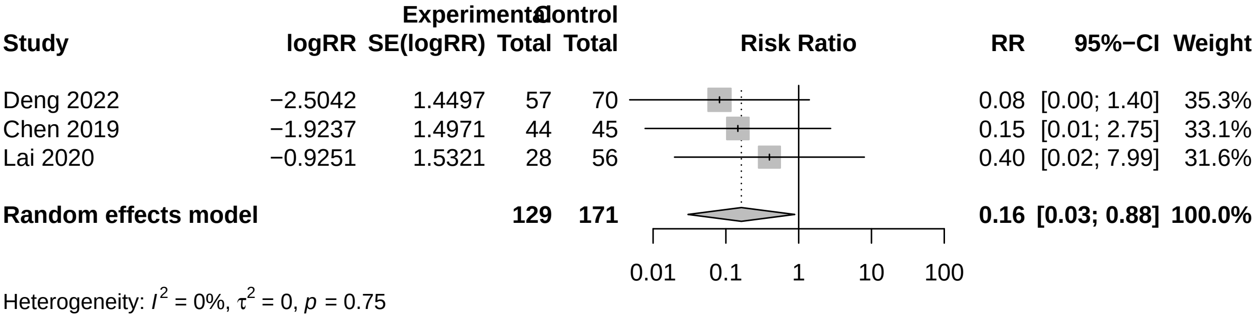

4.16.4 Funnel plot

```
funnel(trimfill(transfusion_metabin))
```

```
## Warning in trimfill.meta(transfusion_metabin): 15 observation(s) dropped due to
## missing values
```

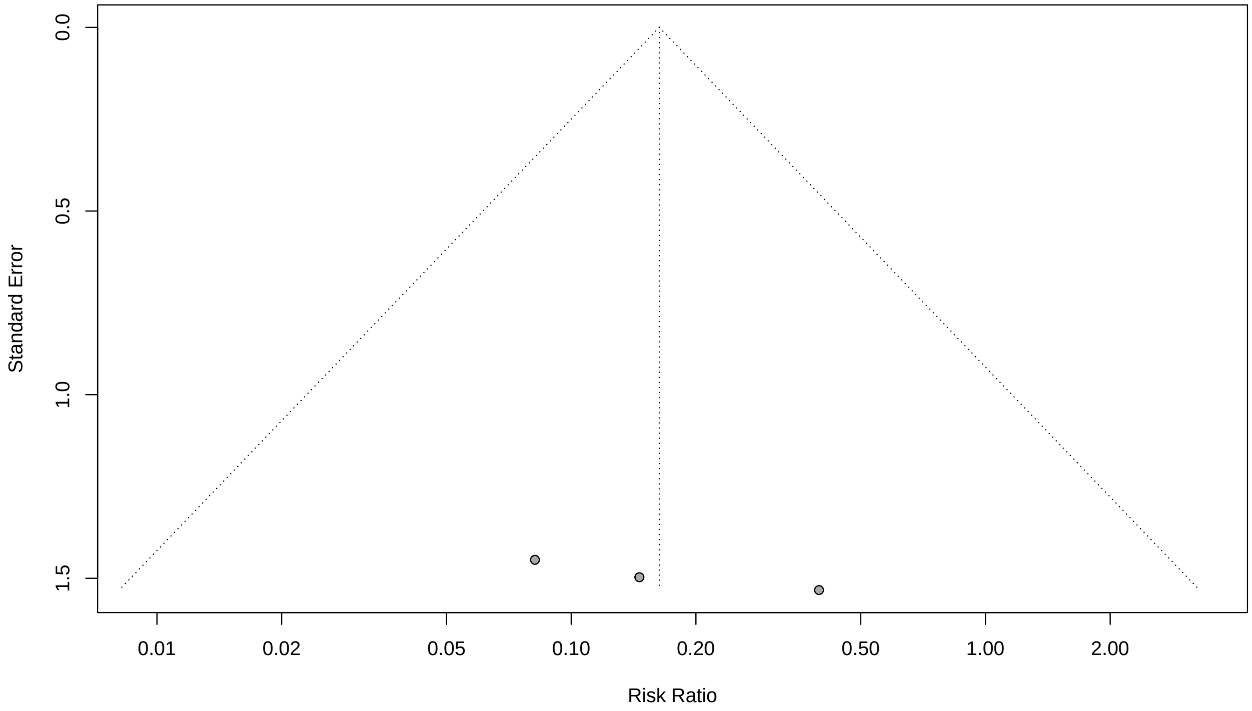

4.16.5 Baujat

```
baujat(transfusion_metabin, pos = 1)
```

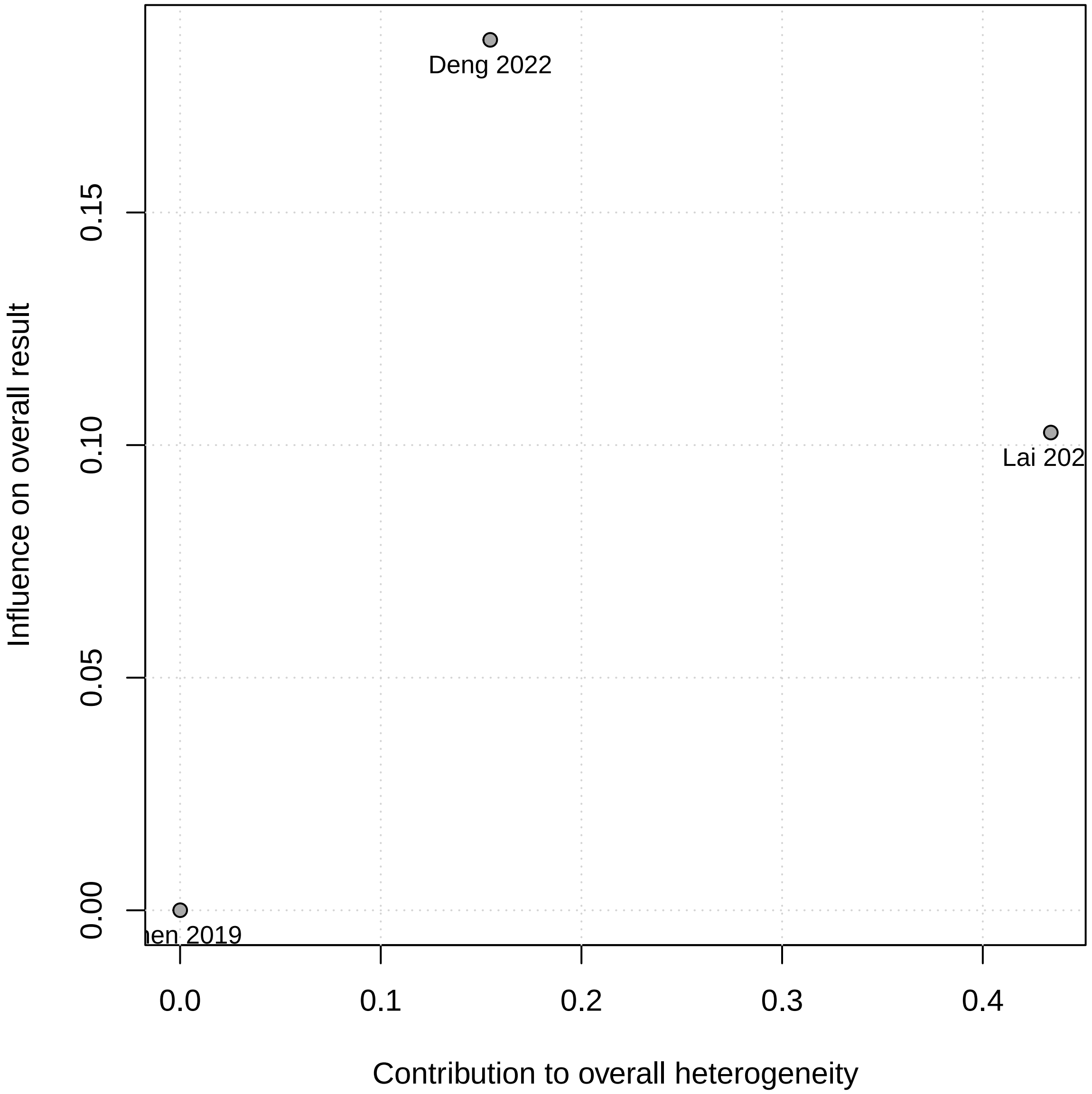

4.16.6 Leave one out

```
metainf(transfusion_metabin)
```

```
## Influential analysis (common effect model)
##
##                                 RR           95%-CI p-value   tau^2    tau
## Omitting Sur 2022           0.1445 [0.0280; 0.7467]  0.0210  0.0000  0.0000
## Omitting Tang 2023          0.1445 [0.0280; 0.7467]  0.0210  0.0000  0.0000
## Omitting Chen 2019          0.1439 [0.0199; 1.0412]  0.0549  0.0000  0.0000
## Omitting Du 2019            0.1445 [0.0280; 0.7467]  0.0210  0.0000  0.0000
## Omitting Lai 2020           0.1035 [0.0134; 0.7992]  0.0296  0.0000  0.0000
## Omitting Zhu 2018           0.1445 [0.0280; 0.7467]  0.0210  0.0000  0.0000
## Omitting Zhang 2021         0.1445 [0.0280; 0.7467]  0.0210  0.0000  0.0000
## Omitting Zhang 2021         0.1445 [0.0280; 0.7467]  0.0210  0.0000  0.0000
## Omitting Lechevallier 2003  0.1445 [0.0280; 0.7467]  0.0210  0.0000  0.0000
## Omitting Huang 2023         0.1445 [0.0280; 0.7467]  0.0210  0.0000  0.0000
## Omitting Wu 2022            0.1445 [0.0280; 0.7467]  0.0210  0.0000  0.0000
## Omitting Ding 2023          0.1445 [0.0280; 0.7467]  0.0210  0.0000  0.0000
## Omitting Zhai 2023          0.1445 [0.0280; 0.7467]  0.0210  0.0000  0.0000
## Omitting Zhai 2023          0.1445 [0.0280; 0.7467]  0.0210  0.0000  0.0000
## Omitting Qian 2022          0.1445 [0.0280; 0.7467]  0.0210  0.0000  0.0000
## Omitting Zhang 2022         0.1445 [0.0280; 0.7467]  0.0210  0.0000  0.0000
## Omitting Deng 2022          0.2270 [0.0294; 1.7540]  0.1552  0.0000  0.0000
## Omitting AlSmadi 2019       0.1445 [0.0280; 0.7467]  0.0210  0.0000  0.0000
##
## Pooled estimate             0.1445 [0.0280; 0.7467]  0.0210  0.0000  0.0000
##                               I^2
## Omitting Sur 2022            0.0%
## Omitting Tang 2023           0.0%
## Omitting Chen 2019           0.0%
## Omitting Du 2019             0.0%
## Omitting Lai 2020            0.0%
## Omitting Zhu 2018            0.0%
## Omitting Zhang 2021          0.0%
## Omitting Zhang 2021          0.0%
## Omitting Lechevallier 2003   0.0%
## Omitting Huang 2023          0.0%
## Omitting Wu 2022             0.0%
## Omitting Ding 2023           0.0%
## Omitting Zhai 2023           0.0%
## Omitting Zhai 2023           0.0%
## Omitting Qian 2022           0.0%
## Omitting Zhang 2022          0.0%
## Omitting Deng 2022           0.0%
## Omitting AlSmadi 2019        0.0%
##
## Pooled estimate              0.0%
##
## Details on meta-analytical method:
## - Mantel-Haenszel method
## - Restricted maximum-likelihood estimator for tau^2
```

4.17 Clavien I

4.17.1 Meta-analysis

```r
clav_i_metabin <- metabin(data = suction_data,
                          event.c = clavien_i_n_control,
                        n.c = sample_size_control,
                        event.e = clavien_i_n_suction,
                        n.e = sample_size_suction,
                        studlab = author_year)
clav_i_metabin
```

```
## Number of studies: k = 8
## Number of observations: o = 2311
## Number of events: e = 92
##
##                           RR           95%-CI     z p-value
## Common effect model  0.6019 [0.4026; 0.9000] -2.47  0.0134
## Random effects model 0.5978 [0.3984; 0.8970] -2.48  0.0130
##
## Quantifying heterogeneity:
##  tau^2 = 0; tau = 0; I^2 = 0.0% [0.0%; 67.6%]; H = 1.00 [1.00; 1.76]
##
## Test of heterogeneity:
##     Q d.f. p-value
##  1.57    7  0.9798
##
## Details on meta-analytical method:
## - Mantel-Haenszel method
## - Inverse variance method
## - Restricted maximum-likelihood estimator for tau^2
## - Continuity correction of 0.5 in studies with zero cell frequencies
```

4.17.2 Forest plot

```
forest(clav_i_metabin,
       sortvar = TE)
```

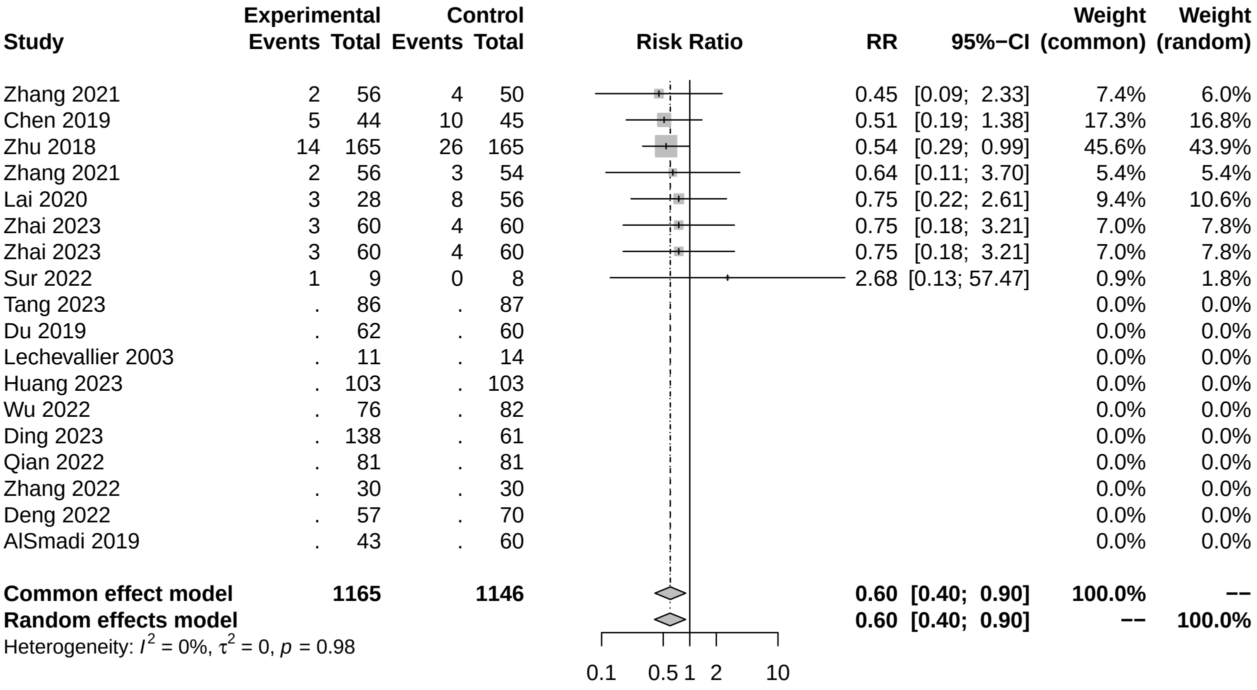

Study	Experimental Events	Experimental Total	Control Events	Control Total	RR	95%−CI	Weight (common)	Weight (random)
Zhang 2021	2	56	4	50	0.45	[0.09; 2.33]	7.4%	6.0%
Chen 2019	5	44	10	45	0.51	[0.19; 1.38]	17.3%	16.8%
Zhu 2018	14	165	26	165	0.54	[0.29; 0.99]	45.6%	43.9%
Zhang 2021	2	56	3	54	0.64	[0.11; 3.70]	5.4%	5.4%
Lai 2020	3	28	8	56	0.75	[0.22; 2.61]	9.4%	10.6%
Zhai 2023	3	60	4	60	0.75	[0.18; 3.21]	7.0%	7.8%
Zhai 2023	3	60	4	60	0.75	[0.18; 3.21]	7.0%	7.8%
Sur 2022	1	9	0	8	2.68	[0.13; 57.47]	0.9%	1.8%
Tang 2023	.	86	.	87			0.0%	0.0%
Du 2019	.	62	.	60			0.0%	0.0%
Lechevallier 2003	.	11	.	14			0.0%	0.0%
Huang 2023	.	103	.	103			0.0%	0.0%
Wu 2022	.	76	.	82			0.0%	0.0%
Ding 2023	.	138	.	61			0.0%	0.0%
Qian 2022	.	81	.	81			0.0%	0.0%
Zhang 2022	.	30	.	30			0.0%	0.0%
Deng 2022	.	57	.	70			0.0%	0.0%
AlSmadi 2019	.	43	.	60			0.0%	0.0%
**Common effect model**		**1165**		**1146**	**0.60**	**[0.40; 0.90]**	**100.0%**	**--**
**Random effects model**					**0.60**	**[0.40; 0.90]**	**--**	**100.0%**

Heterogeneity: $I^2 = 0\%$, $\tau^2 = 0$, $p = 0.98$

4.17.3 Trim and Fill

```
trimfill(clav_i_metabin)
```

```
## Warning in trimfill.meta(clav_i_metabin): 10 observation(s) dropped due to
## missing values

## Number of studies: k = 11 (with 3 added studies)
## Number of observations: o = 1233
## Number of events: e = 107
##
##                           RR           95%-CI     z p-value
## Random effects model 0.5548 [0.3814; 0.8069] -3.08  0.0021
##
## Quantifying heterogeneity:
##  tau^2 = 0; tau = 0; I^2 = 0.0% [0.0%; 60.2%]; H = 1.00 [1.00; 1.59]
##
## Test of heterogeneity:
##     Q d.f. p-value
##  3.05   10  0.9803
##
## Details on meta-analytical method:
## - Inverse variance method
## - Restricted maximum-likelihood estimator for tau^2
## - Trim-and-fill method to adjust for funnel plot asymmetry (L-estimator)
```

```
forest(trimfill(clav_i_metabin),
       sortvar = TE)
```

```
## Warning in trimfill.meta(clav_i_metabin): 10 observation(s) dropped due to
## missing values
```

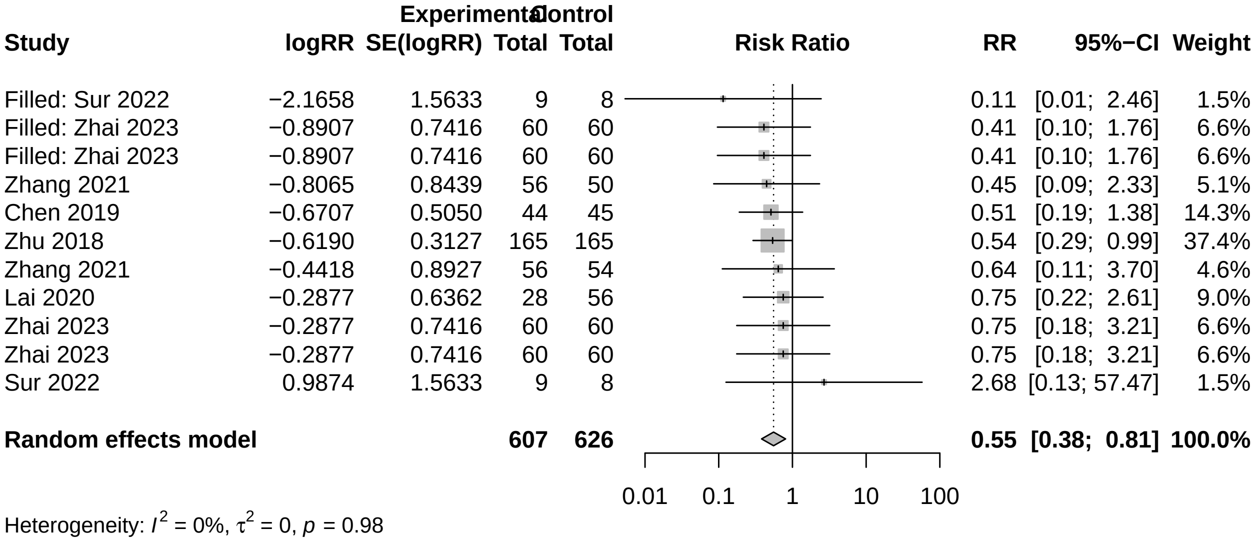

4.17.4 Funnel plot

```
funnel(trimfill(clav_i_metabin))
```

```
## Warning in trimfill.meta(clav_i_metabin): 10 observation(s) dropped due to
## missing values
```

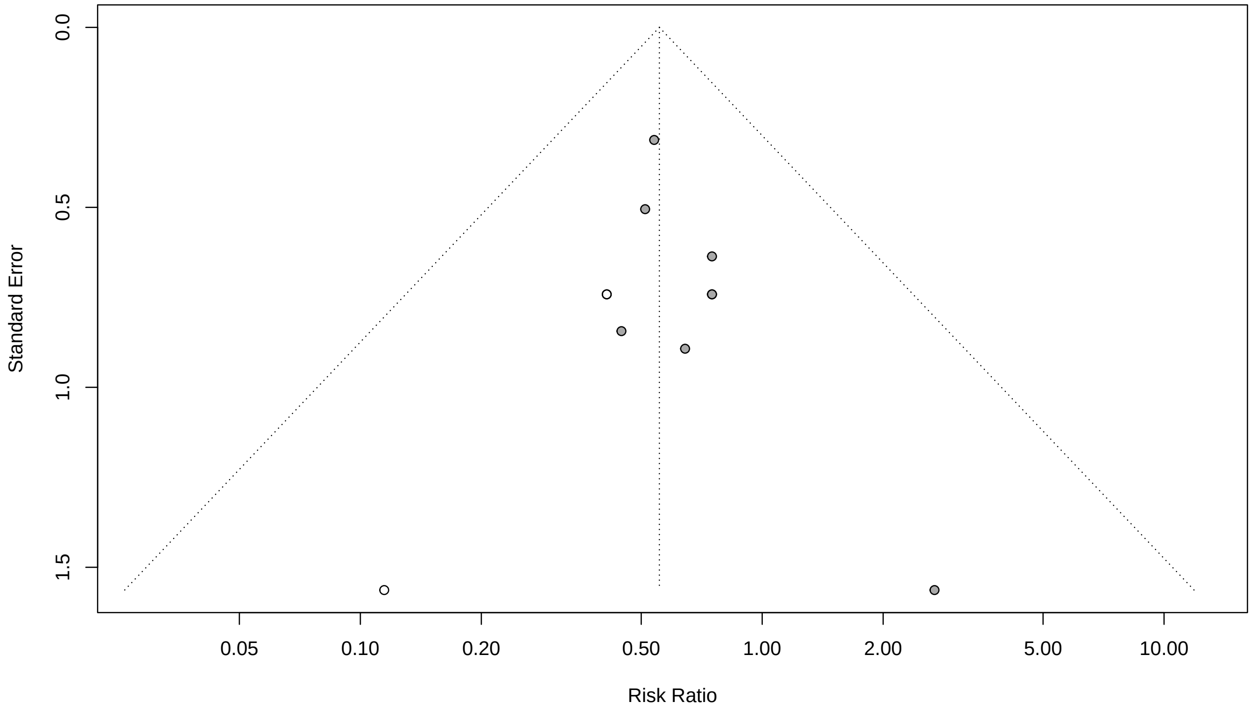

4.17.5 Baujat

```r
baujat(clav_i_metabin, pos = 1)
```

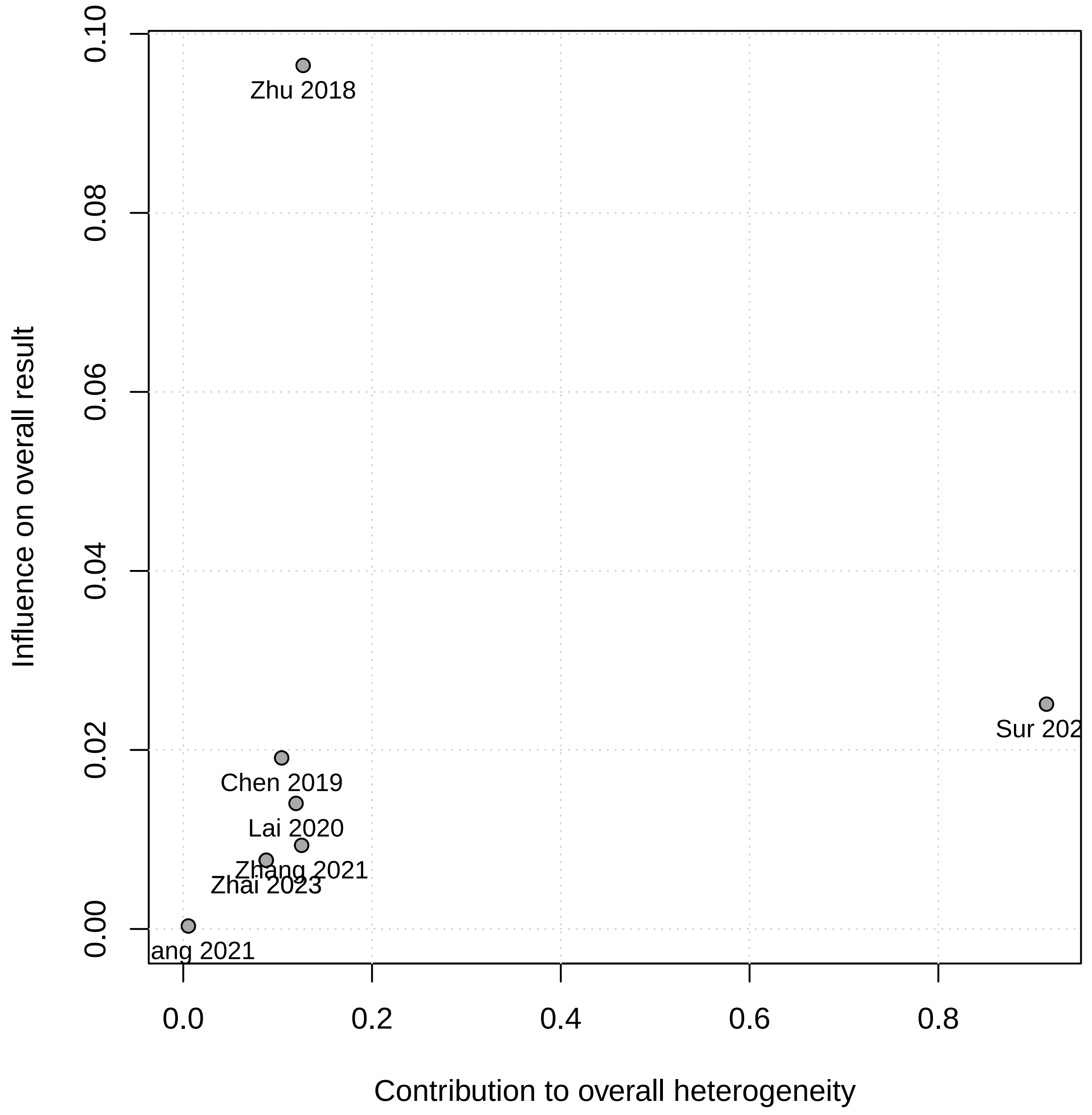

4.17.6 Leave one out

```
metainf(clav_i_metabin)
```

```
## Influential analysis (common effect model)
##
##                                   RR           95%-CI p-value   tau^2     tau
## Omitting Sur 2022             0.5824 [0.3872; 0.8760]  0.0094  0.0000  0.0000
## Omitting Tang 2023            0.6019 [0.4026; 0.9000]  0.0134  0.0000  0.0000
## Omitting Chen 2019            0.6209 [0.3997; 0.9647]  0.0340  0.0000  0.0000
## Omitting Du 2019              0.6019 [0.4026; 0.9000]  0.0134  0.0000  0.0000
## Omitting Lai 2020             0.5867 [0.3834; 0.8977]  0.0140  0.0000  0.0000
## Omitting Zhu 2018             0.6551 [0.3839; 1.1179]  0.1208  0.0000  0.0000
## Omitting Zhang 2021           0.6144 [0.4057; 0.9304]  0.0214  0.0000  0.0000
## Omitting Zhang 2021           0.5996 [0.3966; 0.9065]  0.0153  0.0000  0.0000
## Omitting Lechevallier 2003    0.6019 [0.4026; 0.9000]  0.0134  0.0000  0.0000
## Omitting Huang 2023           0.6019 [0.4026; 0.9000]  0.0134  0.0000  0.0000
## Omitting Wu 2022              0.6019 [0.4026; 0.9000]  0.0134  0.0000  0.0000
## Omitting Ding 2023            0.6019 [0.4026; 0.9000]  0.0134  0.0000  0.0000
## Omitting Zhai 2023            0.5908 [0.3886; 0.8981]  0.0138  0.0000  0.0000
## Omitting Zhai 2023            0.5908 [0.3886; 0.8981]  0.0138  0.0000  0.0000
## Omitting Qian 2022            0.6019 [0.4026; 0.9000]  0.0134  0.0000  0.0000
## Omitting Zhang 2022           0.6019 [0.4026; 0.9000]  0.0134  0.0000  0.0000
## Omitting Deng 2022            0.6019 [0.4026; 0.9000]  0.0134  0.0000  0.0000
## Omitting AlSmadi 2019         0.6019 [0.4026; 0.9000]  0.0134  0.0000  0.0000
##
## Pooled estimate               0.6019 [0.4026; 0.9000]  0.0134  0.0000  0.0000
##                                 I^2
## Omitting Sur 2022              0.0%
## Omitting Tang 2023             0.0%
## Omitting Chen 2019             0.0%
## Omitting Du 2019               0.0%
## Omitting Lai 2020              0.0%
## Omitting Zhu 2018              0.0%
## Omitting Zhang 2021            0.0%
## Omitting Zhang 2021            0.0%
## Omitting Lechevallier 2003     0.0%
## Omitting Huang 2023            0.0%
## Omitting Wu 2022               0.0%
## Omitting Ding 2023             0.0%
## Omitting Zhai 2023             0.0%
## Omitting Zhai 2023             0.0%
## Omitting Qian 2022             0.0%
## Omitting Zhang 2022            0.0%
## Omitting Deng 2022             0.0%
## Omitting AlSmadi 2019          0.0%
##
## Pooled estimate                0.0%
##
## Details on meta-analytical method:
## - Mantel-Haenszel method
## - Restricted maximum-likelihood estimator for tau^2
```

4.18 Clavien II

4.18.1 Meta-analysis

```
clav_ii_metabin <- metabin(data = suction_data,
                         event.c = clavien_ii_n_control,
                       n.c = sample_size_control,
                       event.e = clavien_ii_n_suction,
                       n.e = sample_size_suction,
                       studlab = author_year)
clav_ii_metabin
```

```
## Number of studies: k = 6
## Number of observations: o = 2311
## Number of events: e = 27
##
##                          RR           95%-CI     z p-value
## Common effect model  0.3531 [0.1531; 0.8147] -2.44  0.0147
## Random effects model 0.3714 [0.1564; 0.8821] -2.24  0.0248
##
## Quantifying heterogeneity:
##  tau^2 = 0 [0.0000; 1.6543]; tau = 0 [0.0000; 1.2862]
##  I^2 = 0.0% [0.0%; 74.6%]; H = 1.00 [1.00; 1.99]
##
## Test of heterogeneity:
##     Q d.f. p-value
##  1.71    5  0.8875
##
## Details on meta-analytical method:
## - Mantel-Haenszel method
## - Inverse variance method
## - Restricted maximum-likelihood estimator for tau^2
## - Q-Profile method for confidence interval of tau^2 and tau
## - Continuity correction of 0.5 in studies with zero cell frequencies
```

4.18.2 Forest plot

```r
forest(clav_ii_metabin,
       sortvar = TE)
```

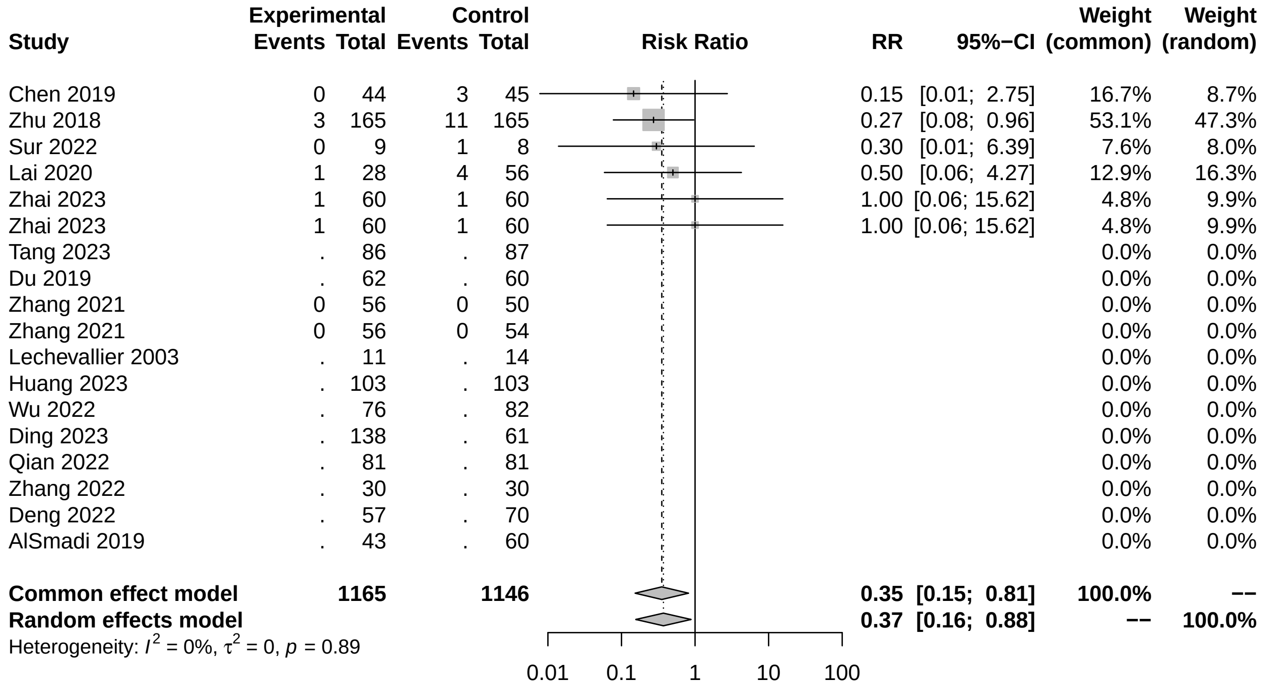

4.18.3 Trim and Fill

```r
trimfill(clav_ii_metabin)
```

```
## Warning in trimfill.meta(clav_ii_metabin): 12 observation(s) dropped due to
## missing values
```

```
## Number of studies: k = 7 (with 1 added studies)
## Number of observations: o = 880
## Number of events: e = 29
##
##                          RR           95%-CI     z p-value
## Random effects model 0.3331 [0.1460; 0.7602] -2.61  0.0090
##
## Quantifying heterogeneity:
##  tau^2 = 0 [0.0000; 1.6913]; tau = 0 [0.0000; 1.3005]
##  I^2 = 0.0% [0.0%; 70.8%]; H = 1.00 [1.00; 1.85]
##
## Test of heterogeneity:
##     Q d.f. p-value
##  2.39    6  0.8809
##
## Details on meta-analytical method:
## - Inverse variance method
## - Restricted maximum-likelihood estimator for tau^2
## - Q-Profile method for confidence interval of tau^2 and tau
## - Trim-and-fill method to adjust for funnel plot asymmetry (L-estimator)
```

```r
forest(trimfill(clav_ii_metabin),
       sortvar = TE)
```

```
## Warning in trimfill.meta(clav_ii_metabin): 12 observation(s) dropped due to
## missing values
```

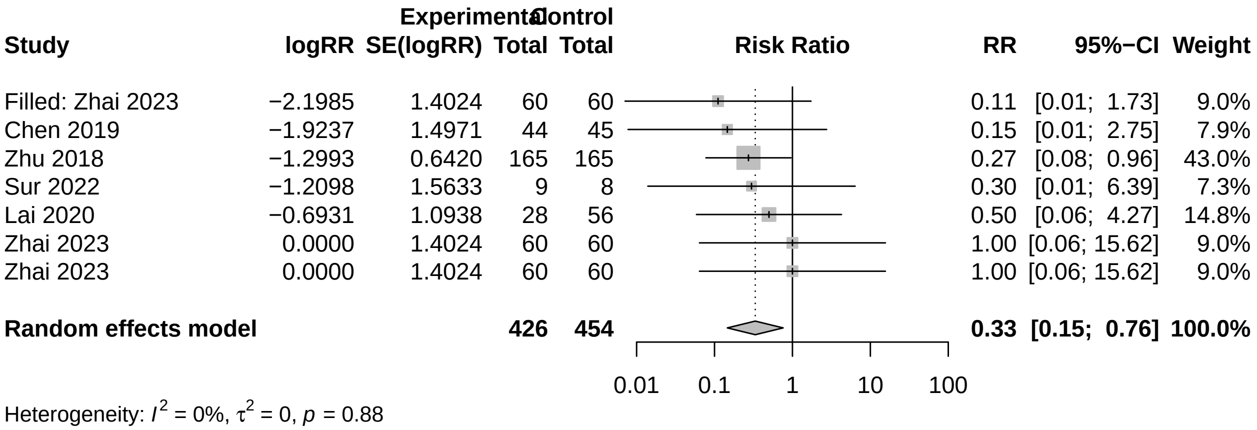

4.18.4 Funnel plot

```
funnel(trimfill(clav_ii_metabin))
```

```
## Warning in trimfill.meta(clav_ii_metabin): 12 observation(s) dropped due to
## missing values
```

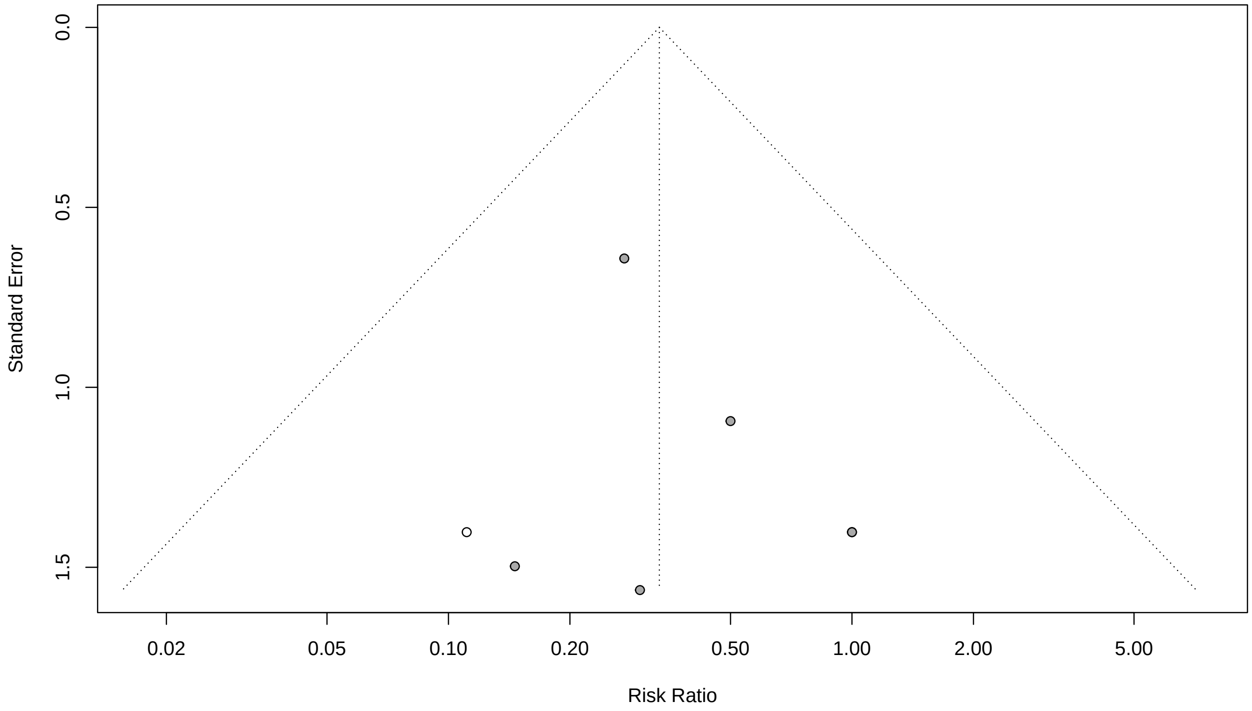

4.18.5 Baujat

```
baujat(clav_ii_metabin, pos = 1)
```

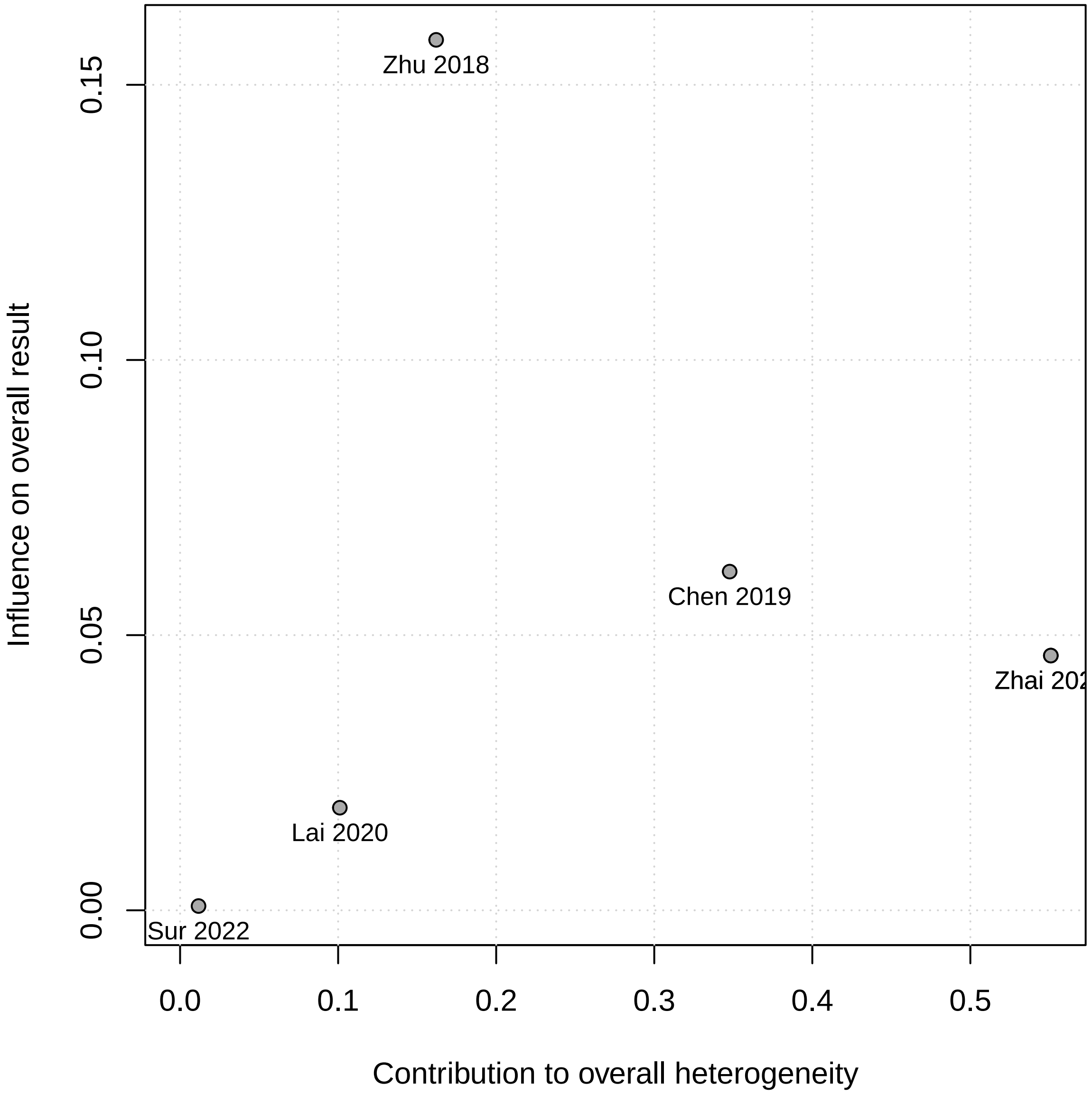

4.18.6 Leave one out

```
metainf(clav_ii_metabin)
```

```
## Influential analysis (common effect model)
##
##                                  RR           95%-CI p-value   tau^2     tau
## Omitting Sur 2022            0.3575 [0.1500; 0.8524]  0.0203  0.0000  0.0000
## Omitting Tang 2023           0.3531 [0.1531; 0.8147]  0.0147  0.0000  0.0000
## Omitting Chen 2019           0.3947 [0.1638; 0.9509]  0.0382  0.0000  0.0000
## Omitting Du 2019             0.3531 [0.1531; 0.8147]  0.0147  0.0000  0.0000
## Omitting Lai 2020            0.3314 [0.1333; 0.8240]  0.0175  0.0000  0.0000
## Omitting Zhu 2018            0.4443 [0.1433; 1.3770]  0.1598  0.0000  0.0000
## Omitting Zhang 2021          0.3531 [0.1531; 0.8147]  0.0147  0.0000  0.0000
## Omitting Zhang 2021          0.3531 [0.1531; 0.8147]  0.0147  0.0000  0.0000
## Omitting Lechevallier 2003   0.3531 [0.1531; 0.8147]  0.0147  0.0000  0.0000
## Omitting Huang 2023          0.3531 [0.1531; 0.8147]  0.0147  0.0000  0.0000
## Omitting Wu 2022             0.3531 [0.1531; 0.8147]  0.0147  0.0000  0.0000
## Omitting Ding 2023           0.3531 [0.1531; 0.8147]  0.0147  0.0000  0.0000
## Omitting Zhai 2023           0.3203 [0.1317; 0.7790]  0.0121  0.0000  0.0000
## Omitting Zhai 2023           0.3203 [0.1317; 0.7790]  0.0121  0.0000  0.0000
## Omitting Qian 2022           0.3531 [0.1531; 0.8147]  0.0147  0.0000  0.0000
## Omitting Zhang 2022          0.3531 [0.1531; 0.8147]  0.0147  0.0000  0.0000
## Omitting Deng 2022           0.3531 [0.1531; 0.8147]  0.0147  0.0000  0.0000
## Omitting AlSmadi 2019        0.3531 [0.1531; 0.8147]  0.0147  0.0000  0.0000
##
## Pooled estimate              0.3531 [0.1531; 0.8147]  0.0147  0.0000  0.0000
##                                I^2
## Omitting Sur 2022             0.0%
## Omitting Tang 2023            0.0%
## Omitting Chen 2019            0.0%
## Omitting Du 2019              0.0%
## Omitting Lai 2020             0.0%
## Omitting Zhu 2018             0.0%
## Omitting Zhang 2021           0.0%
## Omitting Zhang 2021           0.0%
## Omitting Lechevallier 2003    0.0%
## Omitting Huang 2023           0.0%
## Omitting Wu 2022              0.0%
## Omitting Ding 2023            0.0%
## Omitting Zhai 2023            0.0%
## Omitting Zhai 2023            0.0%
## Omitting Qian 2022            0.0%
## Omitting Zhang 2022           0.0%
## Omitting Deng 2022            0.0%
## Omitting AlSmadi 2019         0.0%
##
## Pooled estimate               0.0%
##
## Details on meta-analytical method:
## - Mantel-Haenszel method
## - Restricted maximum-likelihood estimator for tau^2
```

4.19 Clavien III

4.19.1 Meta-analysis

```
clav_iii_metabin <- metabin(data = suction_data,
                         event.c = clavien_iii_n_control,
                       n.c = sample_size_control,
                       event.e = clavien_iii_n_suction,
                       n.e = sample_size_suction,
                       studlab = author_year)
clav_iii_metabin
```

```
## Number of studies: k = 3
## Number of observations: o = 2311
## Number of events: e = 6
##
##                          RR           95%-CI     z p-value
## Common effect model  0.7438 [0.1625; 3.4043] -0.38  0.7029
## Random effects model 0.7707 [0.1617; 3.6724] -0.33  0.7437
##
## Quantifying heterogeneity:
##  tau^2 = 0 [0.0000; 12.9354]; tau = 0 [0.0000; 3.5966]
##  I^2 = 0.0% [0.0%; 89.6%]; H = 1.00 [1.00; 3.10]
##
## Test of heterogeneity:
##     Q d.f. p-value
##  0.33    2  0.8458
##
## Details on meta-analytical method:
## - Mantel-Haenszel method
## - Inverse variance method
## - Restricted maximum-likelihood estimator for tau^2
## - Q-Profile method for confidence interval of tau^2 and tau
## - Continuity correction of 0.5 in studies with zero cell frequencies
```

4.19.2 Forest plot

```r
forest(clav_iii_metabin,
       sortvar = TE)
```

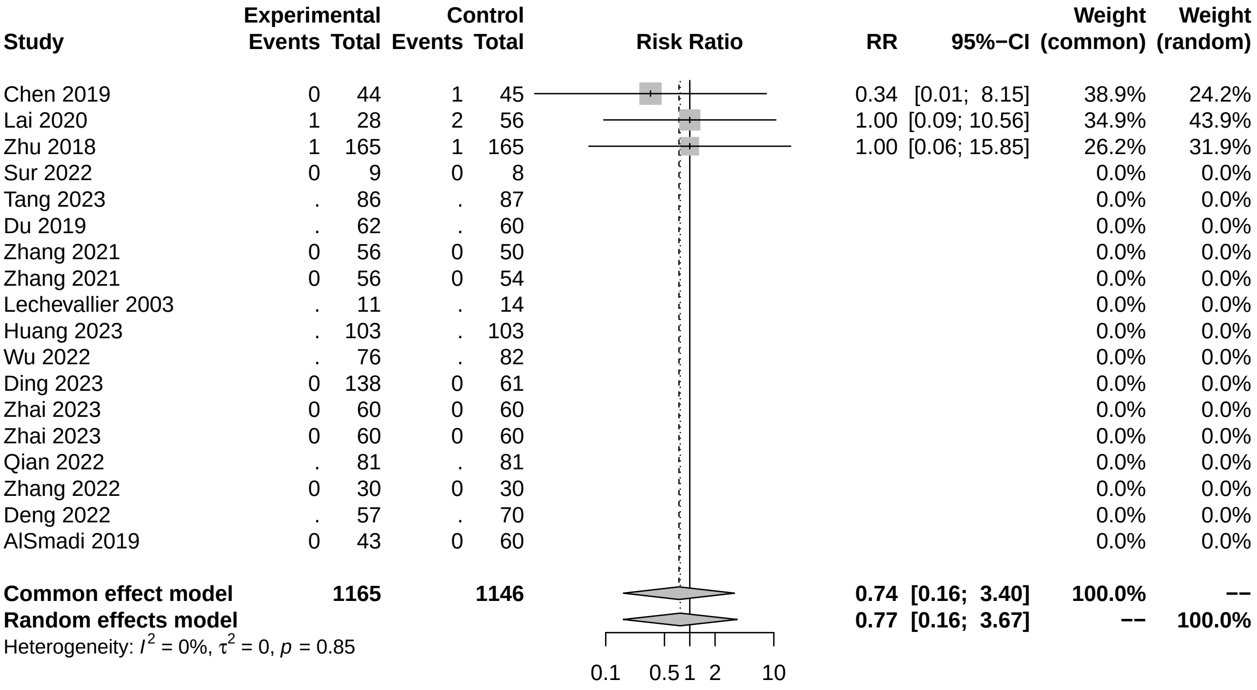

4.19.3 Trim and Fill

```r
trimfill(clav_iii_metabin)
```

```
## Warning in trimfill.meta(clav_iii_metabin): 15 observation(s) dropped due to
## missing values
```

```
## Number of studies: k = 3 (with 0 added studies)
## Number of observations: o = 503
## Number of events: e = 6
##
##                          RR           95%-CI     z p-value
## Random effects model 0.7707 [0.1617; 3.6724] -0.33  0.7437
##
## Quantifying heterogeneity:
##  tau^2 = 0 [0.0000; 12.9354]; tau = 0 [0.0000; 3.5966]
##  I^2 = 0.0% [0.0%; 89.6%]; H = 1.00 [1.00; 3.10]
##
## Test of heterogeneity:
##     Q d.f. p-value
##  0.33    2  0.8458
##
## Details on meta-analytical method:
## - Inverse variance method
## - Restricted maximum-likelihood estimator for tau^2
## - Q-Profile method for confidence interval of tau^2 and tau
## - Trim-and-fill method to adjust for funnel plot asymmetry (L-estimator)
```

```r
forest(trimfill(clav_iii_metabin),
       sortvar = TE)
```

```
## Warning in trimfill.meta(clav_iii_metabin): 15 observation(s) dropped due to
## missing values
```

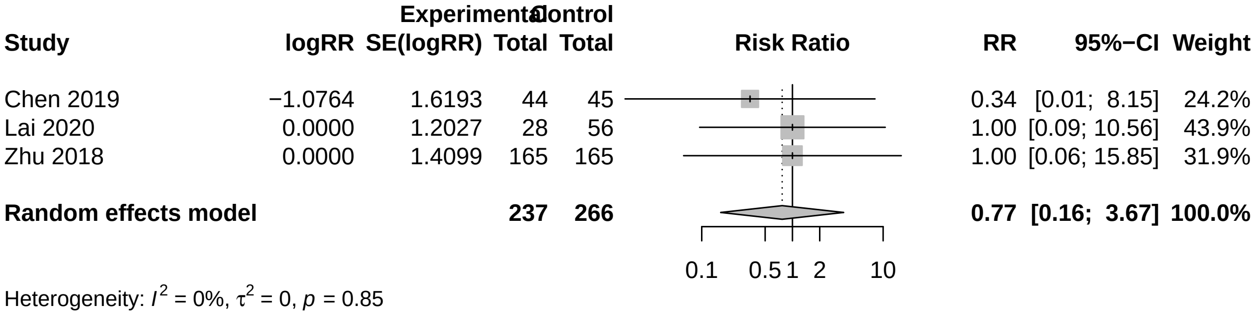

4.19.4 Funnel plot

```
funnel(trimfill(clav_iii_metabin))
```

```
## Warning in trimfill.meta(clav_iii_metabin): 15 observation(s) dropped due to
## missing values
```

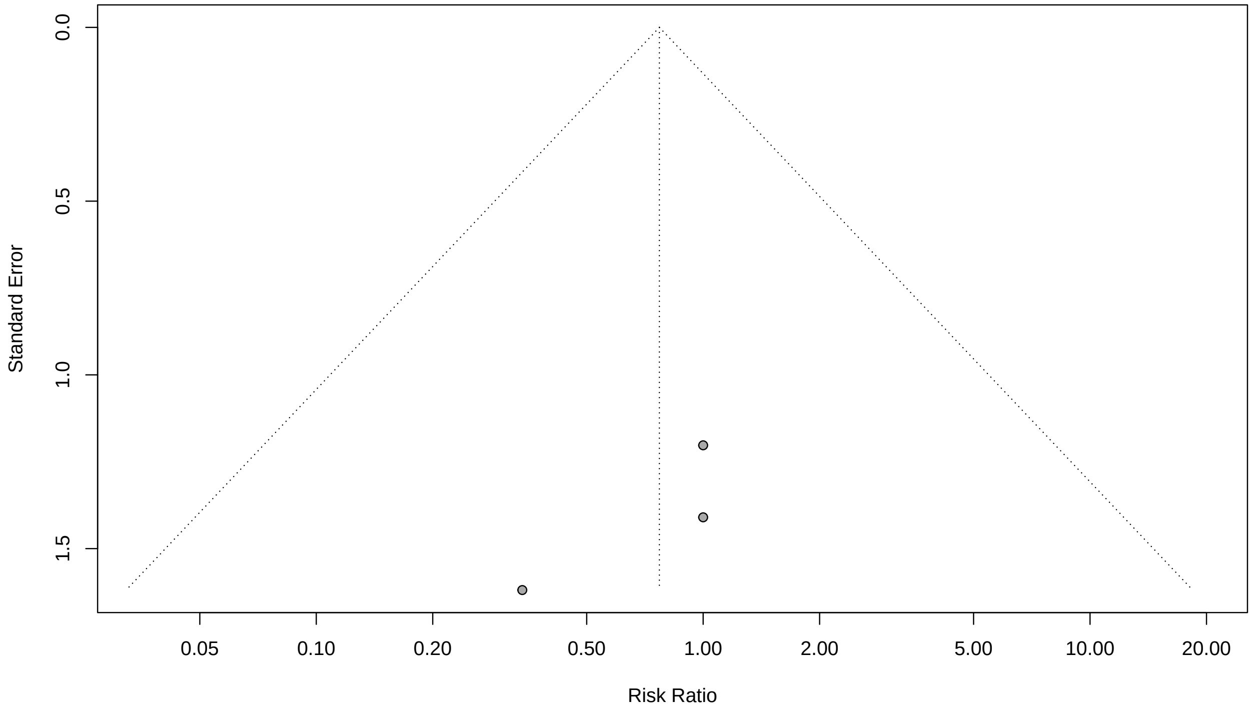

4.19.5 Baujat

```r
baujat(clav_iii_metabin, pos = 1)
```

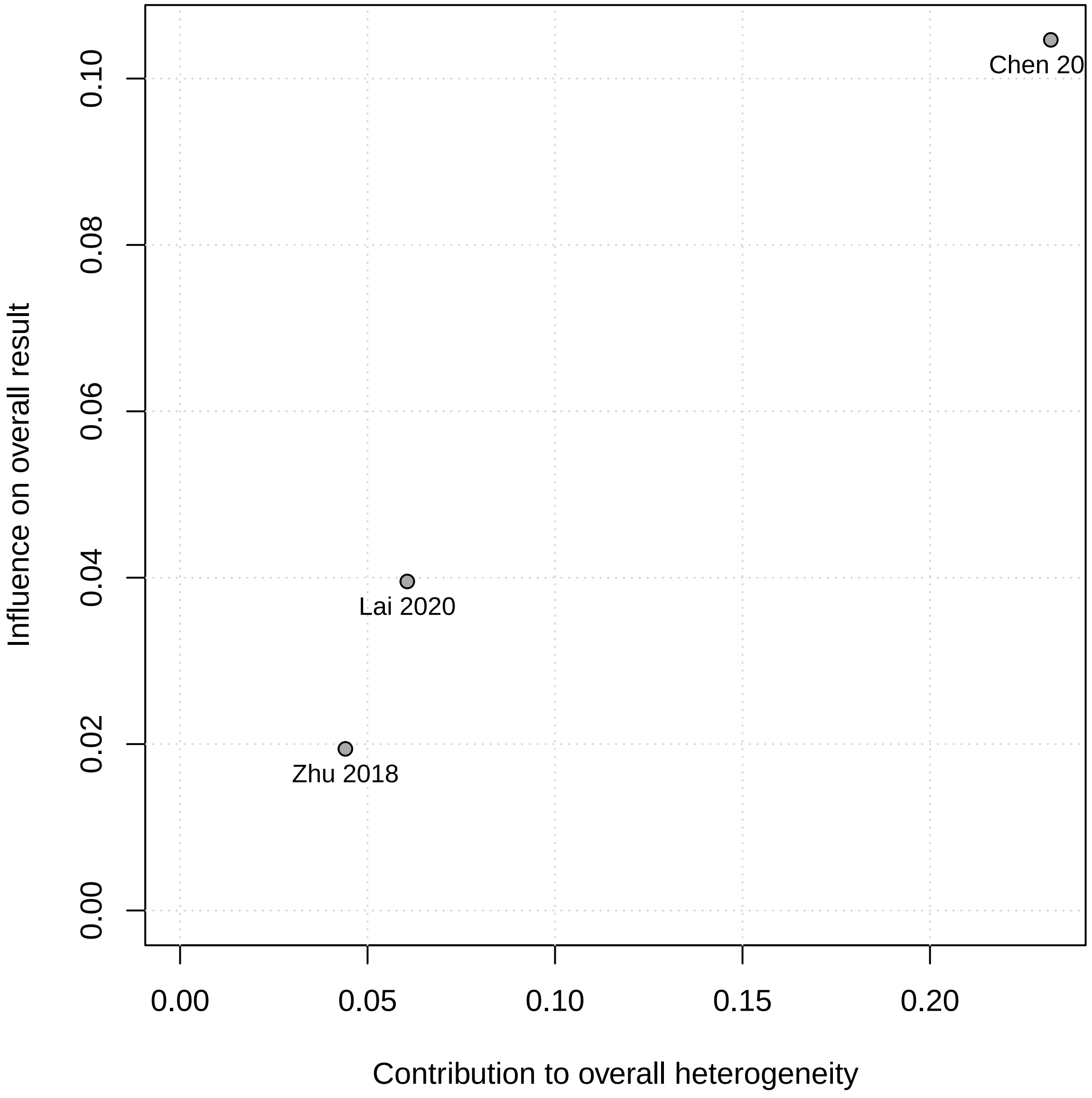

4.19.6 Leave one out

```
metainf(clav_iii_metabin)
```

```
## Influential analysis (common effect model)
##
##                                  RR           95%-CI p-value   tau^2     tau
## Omitting Sur 2022            0.7438 [0.1625; 3.4043]  0.7029  0.0000  0.0000
## Omitting Tang 2023           0.7438 [0.1625; 3.4043]  0.7029  0.0000  0.0000
## Omitting Chen 2019           1.0000 [0.1664; 6.0109]  1.0000  0.0000  0.0000
## Omitting Du 2019             0.7438 [0.1625; 3.4043]  0.7029  0.0000  0.0000
## Omitting Lai 2020            0.6062 [0.0808; 4.5502]  0.6265  0.0000  0.0000
## Omitting Zhu 2018            0.6528 [0.1042; 4.0882]  0.6487  0.0000  0.0000
## Omitting Zhang 2021          0.7438 [0.1625; 3.4043]  0.7029  0.0000  0.0000
## Omitting Zhang 2021          0.7438 [0.1625; 3.4043]  0.7029  0.0000  0.0000
## Omitting Lechevallier 2003   0.7438 [0.1625; 3.4043]  0.7029  0.0000  0.0000
## Omitting Huang 2023          0.7438 [0.1625; 3.4043]  0.7029  0.0000  0.0000
## Omitting Wu 2022             0.7438 [0.1625; 3.4043]  0.7029  0.0000  0.0000
## Omitting Ding 2023           0.7438 [0.1625; 3.4043]  0.7029  0.0000  0.0000
## Omitting Zhai 2023           0.7438 [0.1625; 3.4043]  0.7029  0.0000  0.0000
## Omitting Zhai 2023           0.7438 [0.1625; 3.4043]  0.7029  0.0000  0.0000
## Omitting Qian 2022           0.7438 [0.1625; 3.4043]  0.7029  0.0000  0.0000
## Omitting Zhang 2022          0.7438 [0.1625; 3.4043]  0.7029  0.0000  0.0000
## Omitting Deng 2022           0.7438 [0.1625; 3.4043]  0.7029  0.0000  0.0000
## Omitting AlSmadi 2019        0.7438 [0.1625; 3.4043]  0.7029  0.0000  0.0000
##
## Pooled estimate              0.7438 [0.1625; 3.4043]  0.7029  0.0000  0.0000
##                                I^2
## Omitting Sur 2022             0.0%
## Omitting Tang 2023            0.0%
## Omitting Chen 2019            0.0%
## Omitting Du 2019              0.0%
## Omitting Lai 2020             0.0%
## Omitting Zhu 2018             0.0%
## Omitting Zhang 2021           0.0%
## Omitting Zhang 2021           0.0%
## Omitting Lechevallier 2003    0.0%
## Omitting Huang 2023           0.0%
## Omitting Wu 2022              0.0%
## Omitting Ding 2023            0.0%
## Omitting Zhai 2023            0.0%
## Omitting Zhai 2023            0.0%
## Omitting Qian 2022            0.0%
## Omitting Zhang 2022           0.0%
## Omitting Deng 2022            0.0%
## Omitting AlSmadi 2019         0.0%
##
## Pooled estimate               0.0%
##
## Details on meta-analytical method:
## - Mantel-Haenszel method
## - Restricted maximum-likelihood estimator for tau^2
```

4.20 Clavien IV

4.20.1 Meta-analysis

```
clav_iv_metabin <- metabin(data = suction_data,
                           event.c = clavien_iv_n_control,
                         n.c = sample_size_control,
                         event.e = clavien_iv_n_suction,
                         n.e = sample_size_suction,
                         studlab = author_year)
clav_iv_metabin
```

```
## Number of studies: k = 1
## Number of observations: o = 2311
## Number of events: e = 4
##
##                          RR           95%-CI     z p-value
## Common effect model  0.3333 [0.0350; 3.1716] -0.96  0.3392
## Random effects model 0.3333 [0.0350; 3.1716] -0.96  0.3392
##
## Quantifying heterogeneity:
##  tau^2 = NA; tau = NA; I^2 = NA; H = NA
##
## Details on meta-analytical method:
## - Mantel-Haenszel method
## - Inverse variance method
## - Restricted maximum-likelihood estimator for tau^2
```

4.20.2 Forest plot

```r
forest(clav_iv_metabin,
       sortvar = TE)
```

Study	Experimental Events	Experimental Total	Control Events	Control Total	Risk Ratio	RR	95%−CI	Weight (common)	Weight (random)
Zhu 2018	1	165	3	165		0.33	[0.04; 3.17]	100.0%	100.0%
Sur 2022	0	9	0	8				0.0%	0.0%
Tang 2023	.	86	.	87				0.0%	0.0%
Chen 2019	0	44	0	45				0.0%	0.0%
Du 2019	.	62	.	60				0.0%	0.0%
Lai 2020	0	28	0	56				0.0%	0.0%
Zhang 2021	0	56	0	50				0.0%	0.0%
Zhang 2021	0	56	0	54				0.0%	0.0%
Lechevallier 2003	.	11	.	14				0.0%	0.0%
Huang 2023	.	103	.	103				0.0%	0.0%
Wu 2022	.	76	.	82				0.0%	0.0%
Ding 2023	0	138	0	61				0.0%	0.0%
Zhai 2023	0	60	0	60				0.0%	0.0%
Zhai 2023	0	60	0	60				0.0%	0.0%
Qian 2022	.	81	.	81				0.0%	0.0%
Zhang 2022	0	30	0	30				0.0%	0.0%
Deng 2022	.	57	.	70				0.0%	0.0%
AlSmadi 2019	0	43	0	60				0.0%	0.0%
**Common effect model**		**1165**		**1146**		**0.33**	**[0.04; 3.17]**	**100.0%**	**−−**
**Random effects model**						**0.33**	**[0.04; 3.17]**	**−−**	**100.0%**

Heterogeneity: I^2 = NA%, τ^2 = NA, p = NA

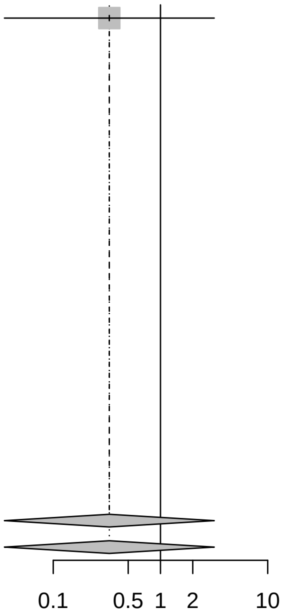

4.20.3 Trim and Fill

```
#trimfill(clav_iv_metabin)
#forest(trimfill(clav_iv_metabin),
#       sortvar = TE)
```

4.20.4 Funnel plot

```
funnel((clav_iv_metabin))
```

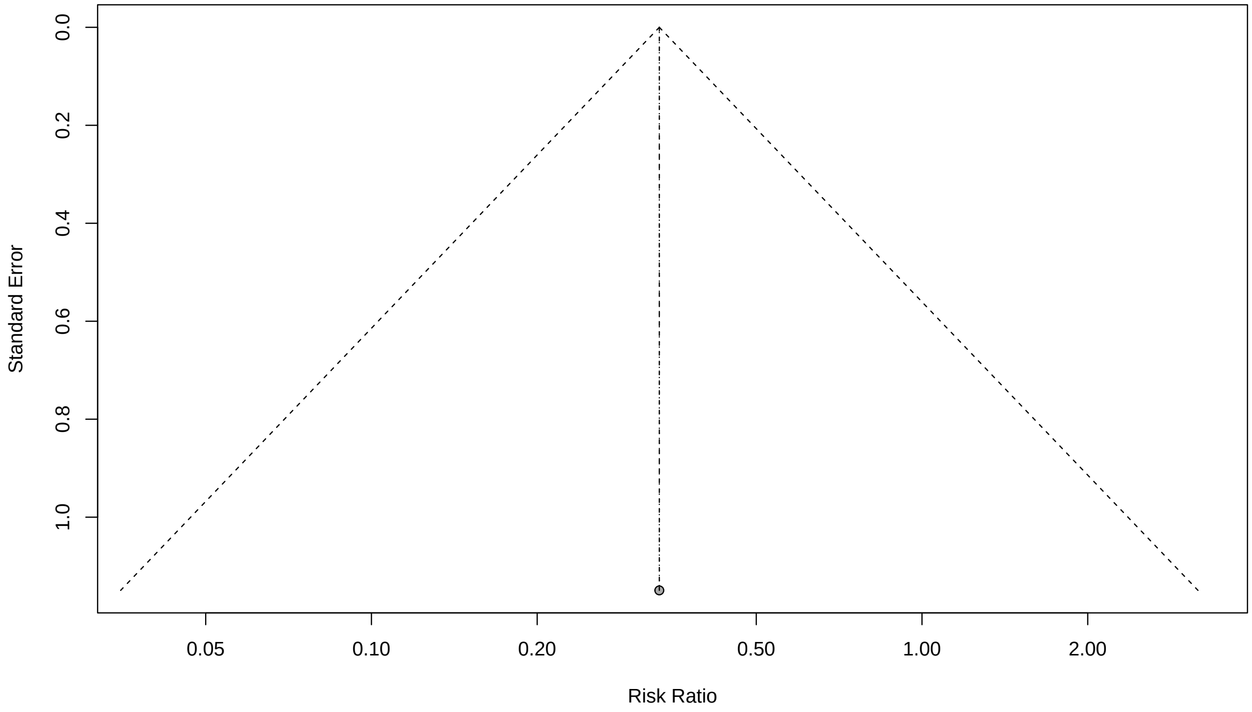

4.20.5 Baujat

```
baujat(clav_iv_metabin, pos = 1)
```

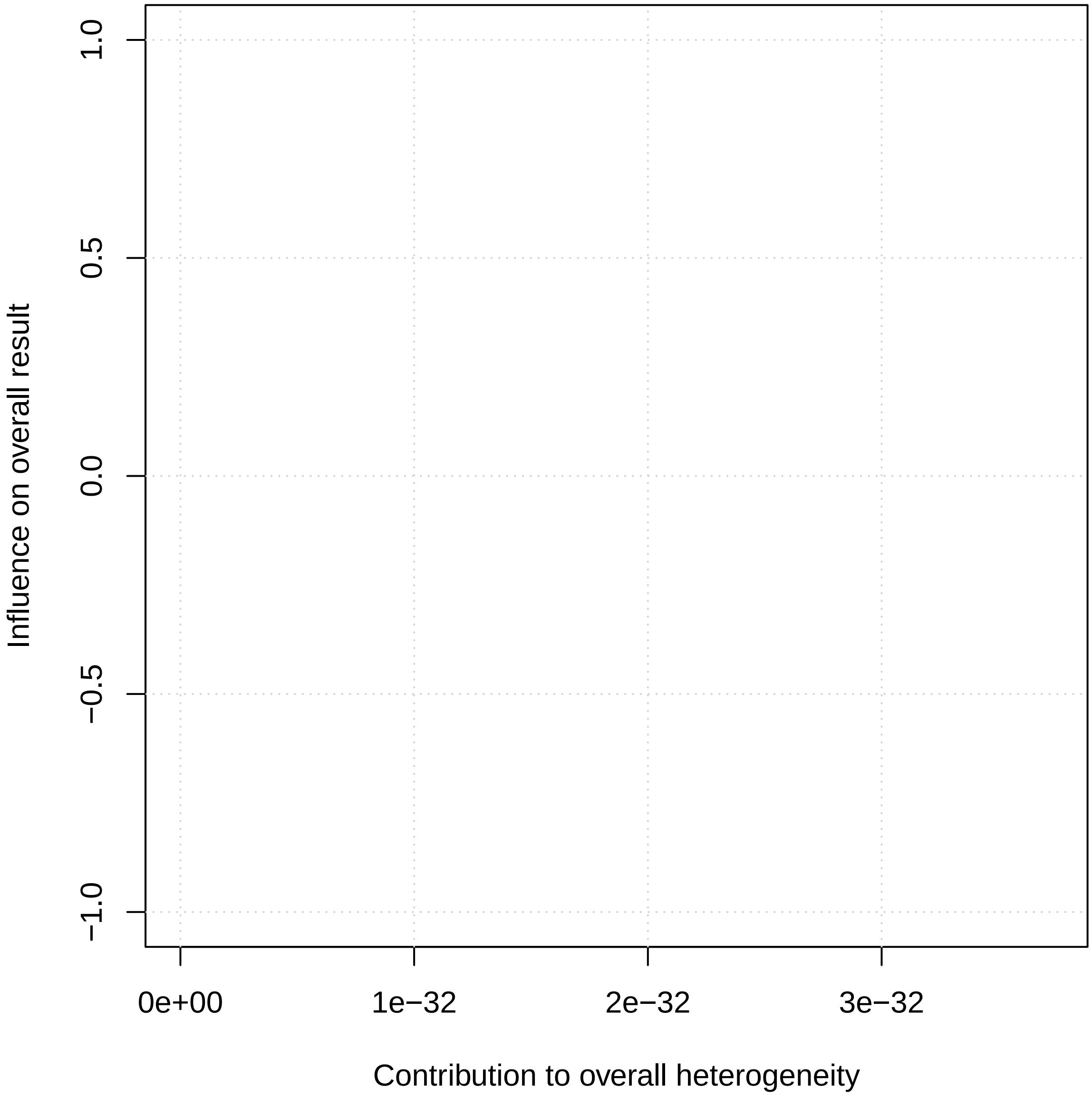

4.20.6 Leave one out

```
metainf(clav_iv_metabin)
```

```
## Influential analysis (common effect model)
##
##                                    RR           95%-CI p-value tau^2 tau I^2
## Omitting Sur 2022              0.3333 [0.0350; 3.1716]  0.3392
## Omitting Tang 2023             0.3333 [0.0350; 3.1716]  0.3392
## Omitting Chen 2019             0.3333 [0.0350; 3.1716]  0.3392
## Omitting Du 2019               0.3333 [0.0350; 3.1716]  0.3392
## Omitting Lai 2020              0.3333 [0.0350; 3.1716]  0.3392
## Omitting Zhu 2018
## Omitting Zhang 2021            0.3333 [0.0350; 3.1716]  0.3392
## Omitting Zhang 2021            0.3333 [0.0350; 3.1716]  0.3392
## Omitting Lechevallier 2003     0.3333 [0.0350; 3.1716]  0.3392
## Omitting Huang 2023            0.3333 [0.0350; 3.1716]  0.3392
## Omitting Wu 2022               0.3333 [0.0350; 3.1716]  0.3392
## Omitting Ding 2023             0.3333 [0.0350; 3.1716]  0.3392
## Omitting Zhai 2023             0.3333 [0.0350; 3.1716]  0.3392
## Omitting Zhai 2023             0.3333 [0.0350; 3.1716]  0.3392
## Omitting Qian 2022             0.3333 [0.0350; 3.1716]  0.3392
## Omitting Zhang 2022            0.3333 [0.0350; 3.1716]  0.3392
## Omitting Deng 2022             0.3333 [0.0350; 3.1716]  0.3392
## Omitting AlSmadi 2019          0.3333 [0.0350; 3.1716]  0.3392
##
## Pooled estimate                0.3333 [0.0350; 3.1716]  0.3392
##
## Details on meta-analytical method:
## - Mantel-Haenszel method
## - Restricted maximum-likelihood estimator for tau^2
```

4.21 Clavien V

4.21.1 Meta-analysis

```
clav_v_metabin <- metabin(data = suction_data,
                         event.c = clavien_v_n_control,
                       n.c = sample_size_control,
                       event.e = clavien_v_n_suction,
                       n.e = sample_size_suction,
                       studlab = author_year)
clav_v_metabin
```

```
## Number of studies: k = 0
## Number of observations: o = 2311
## Number of events: e = 0
##
##                      RR 95%-CI  z p-value
## Common effect model  NA        --      --
## Random effects model NA        --      --
##
## Quantifying heterogeneity:
##  tau^2 = NA; tau = NA; I^2 = NA; H = NA
##
## Details on meta-analytical method:
## - Mantel-Haenszel method
## - Inverse variance method
## - Restricted maximum-likelihood estimator for tau^2
```

4.21.2 Forest plot

```
#forest(clav_v_metabin,
#        sortvar = TE)
```

4.21.3 Trim and Fill

```
#trimfill(clav_v_metabin)
#forest(trimfill(clav_v_metabin),
#       sortvar = TE)
```

4.21.4 Funnel plot

```r
#funnel(trimfill(clav_v_metabin))
```

4.21.5 Baujat

```
#baujat(clav_v_metabin, pos = 1)
```

4.21.6 Leave one out

```
#metainf(clav_v_metabin)
```

4.22 Clavien I-II

4.22.1 Meta-analysis

```
clav_i_ii_metabin <- metabin(data = suction_data,
                         event.c = clav_i_ii_n_control,
                       n.c = sample_size_control,
                       event.e = clav_i_ii_n_suction,
                       n.e = sample_size_suction,
                       studlab = author_year)
clav_i_ii_metabin
```

```
## Number of studies: k = 8
## Number of observations: o = 2311
## Number of events: e = 119
##
##                           RR           95%-CI     z p-value
## Common effect model  0.5274 [0.3686; 0.7545] -3.50  0.0005
## Random effects model 0.5276 [0.3677; 0.7570] -3.47  0.0005
##
## Quantifying heterogeneity:
##  tau^2 = 0 [0.0000; 0.0306]; tau = 0 [0.0000; 0.1749]
##  I^2 = 0.0% [0.0%; 67.6%]; H = 1.00 [1.00; 1.76]
##
## Test of heterogeneity:
##     Q d.f. p-value
##  1.90    7  0.9651
##
## Details on meta-analytical method:
## - Mantel-Haenszel method
## - Inverse variance method
## - Restricted maximum-likelihood estimator for tau^2
## - Q-Profile method for confidence interval of tau^2 and tau
```

4.22.2 Forest plot

```r
forest(clav_i_ii_metabin,
       sortvar = TE)
```

Study	Experimental Events	Experimental Total	Control Events	Control Total	RR	95%−CI	Weight (common)	Weight (random)
Chen 2019	5	44	13	45	0.39	[0.15; 1.01]	16.9%	14.6%
Zhang 2021	2	56	4	50	0.45	[0.09; 2.33]	5.5%	4.8%
Zhu 2018	17	165	37	165	0.46	[0.27; 0.78]	48.6%	46.0%
Zhang 2021	2	56	3	54	0.64	[0.11; 3.70]	4.0%	4.3%
Lai 2020	4	28	12	56	0.67	[0.24; 1.88]	10.5%	12.1%
Zhai 2023	4	60	5	60	0.80	[0.23; 2.83]	6.6%	8.1%
Zhai 2023	4	60	5	60	0.80	[0.23; 2.83]	6.6%	8.1%
Sur 2022	1	9	1	8	0.89	[0.07; 12.00]	1.4%	1.9%
Tang 2023	.	86	.	87			0.0%	0.0%
Du 2019	.	62	.	60			0.0%	0.0%
Lechevallier 2003	.	11	.	14			0.0%	0.0%
Huang 2023	.	103	.	103			0.0%	0.0%
Wu 2022	.	76	.	82			0.0%	0.0%
Ding 2023	.	138	.	61			0.0%	0.0%
Qian 2022	.	81	.	81			0.0%	0.0%
Zhang 2022	.	30	.	30			0.0%	0.0%
Deng 2022	.	57	.	70			0.0%	0.0%
AlSmadi 2019	.	43	.	60			0.0%	0.0%
**Common effect model**		**1165**		**1146**	**0.53**	**[0.37; 0.75]**	**100.0%**	**--**
**Random effects model**					**0.53**	**[0.37; 0.76]**	**--**	**100.0%**

Heterogeneity: $I^2 = 0\%$, $\tau^2 = 0$, $p = 0.97$

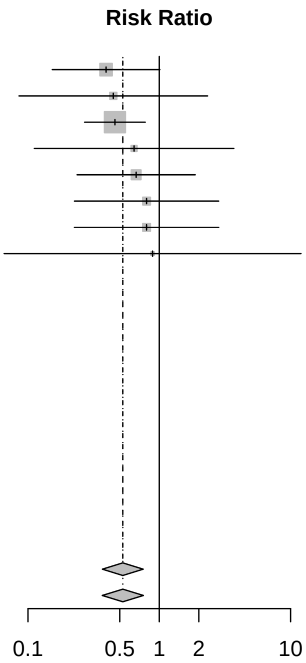

4.22.3 Trim and Fill

```r
trimfill(clav_i_ii_metabin)
```

```
## Warning in trimfill.meta(clav_i_ii_metabin): 10 observation(s) dropped due to
## missing values
```

```
## Number of studies: k = 11 (with 3 added studies)
## Number of observations: o = 1233
## Number of events: e = 139
##
##                           RR           95%-CI     z  p-value
## Random effects model 0.4798 [0.3442; 0.6687] -4.34 < 0.0001
##
## Quantifying heterogeneity:
##  tau^2 = 0 [0.0000; 0.0505]; tau = 0 [0.0000; 0.2246]
##  I^2 = 0.0% [0.0%; 60.2%]; H = 1.00 [1.00; 1.59]
##
## Test of heterogeneity:
##     Q d.f. p-value
##  3.64   10  0.9622
##
## Details on meta-analytical method:
## - Inverse variance method
## - Restricted maximum-likelihood estimator for tau^2
## - Q-Profile method for confidence interval of tau^2 and tau
## - Trim-and-fill method to adjust for funnel plot asymmetry (L-estimator)
```

```r
forest(trimfill(clav_i_ii_metabin),
       sortvar = TE)
```

```
## Warning in trimfill.meta(clav_i_ii_metabin): 10 observation(s) dropped due to
## missing values
```

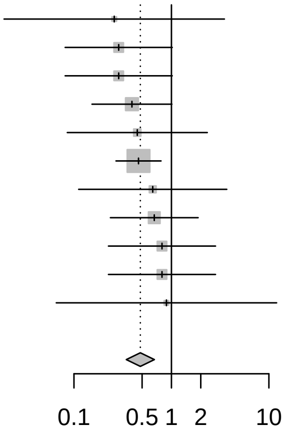

Study	logRR	SE(logRR)	Experimental Total	Control Total	Risk Ratio	RR	95%−CI	Weight
Filled: Sur 2022	−1.3512	1.3281	9	8		0.26	[0.02; 3.50]	1.6%
Filled: Zhai 2023	−1.2458	0.6455	60	60		0.29	[0.08; 1.02]	6.9%
Filled: Zhai 2023	−1.2458	0.6455	60	60		0.29	[0.08; 1.02]	6.9%
Chen 2019	−0.9330	0.4816	44	45		0.39	[0.15; 1.01]	12.4%
Zhang 2021	−0.8065	0.8439	56	50		0.45	[0.09; 2.33]	4.0%
Zhu 2018	−0.7777	0.2715	165	165		0.46	[0.27; 0.78]	38.9%
Zhang 2021	−0.4418	0.8927	56	54		0.64	[0.11; 3.70]	3.6%
Lai 2020	−0.4055	0.5289	28	56		0.67	[0.24; 1.88]	10.3%
Zhai 2023	−0.2231	0.6455	60	60		0.80	[0.23; 2.83]	6.9%
Zhai 2023	−0.2231	0.6455	60	60		0.80	[0.23; 2.83]	6.9%
Sur 2022	−0.1178	1.3281	9	8		0.89	[0.07; 12.00]	1.6%
**Random effects model**			**607**	**626**		**0.48**	**[0.34; 0.67]**	**100.0%**

Heterogeneity: $I^2 = 0\%$, $\tau^2 = 0$, $p = 0.96$

4.22.4 Funnel plot

```
funnel(trimfill(clav_i_ii_metabin))
```

```
## Warning in trimfill.meta(clav_i_ii_metabin): 10 observation(s) dropped due to
## missing values
```

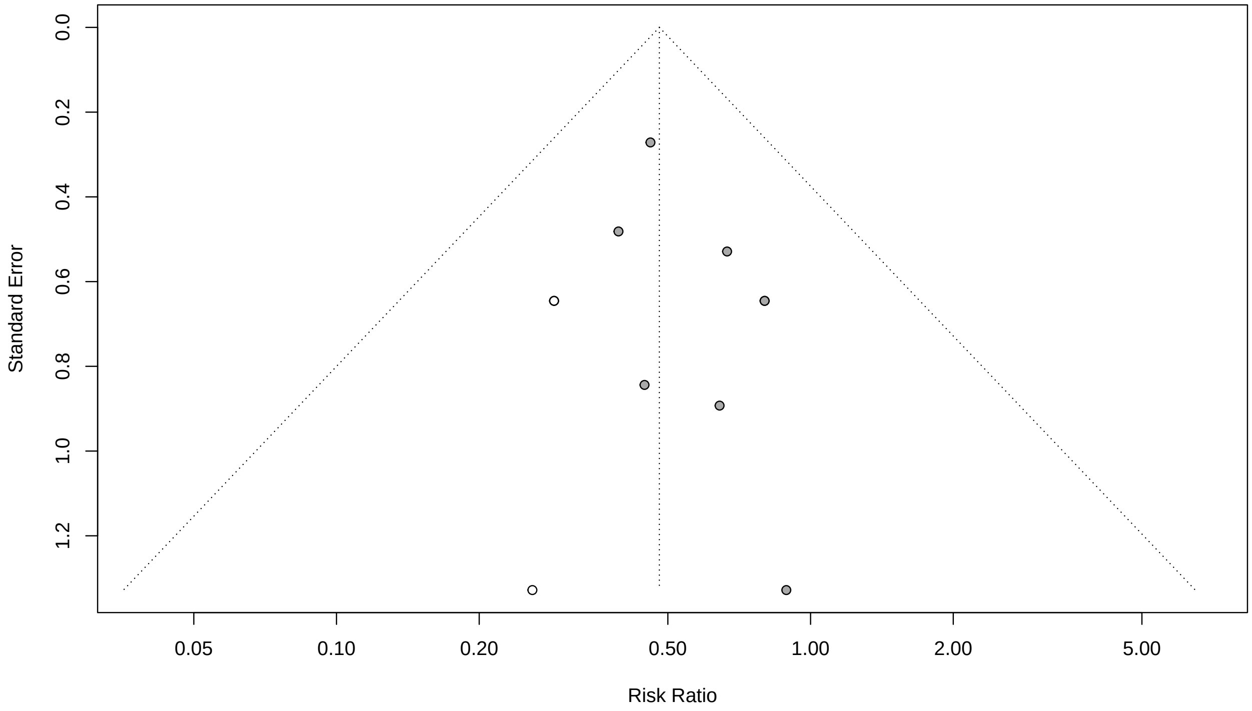

4.22.5 Baujat

```
baujat(clav_i_ii_metabin, pos = 1)
```

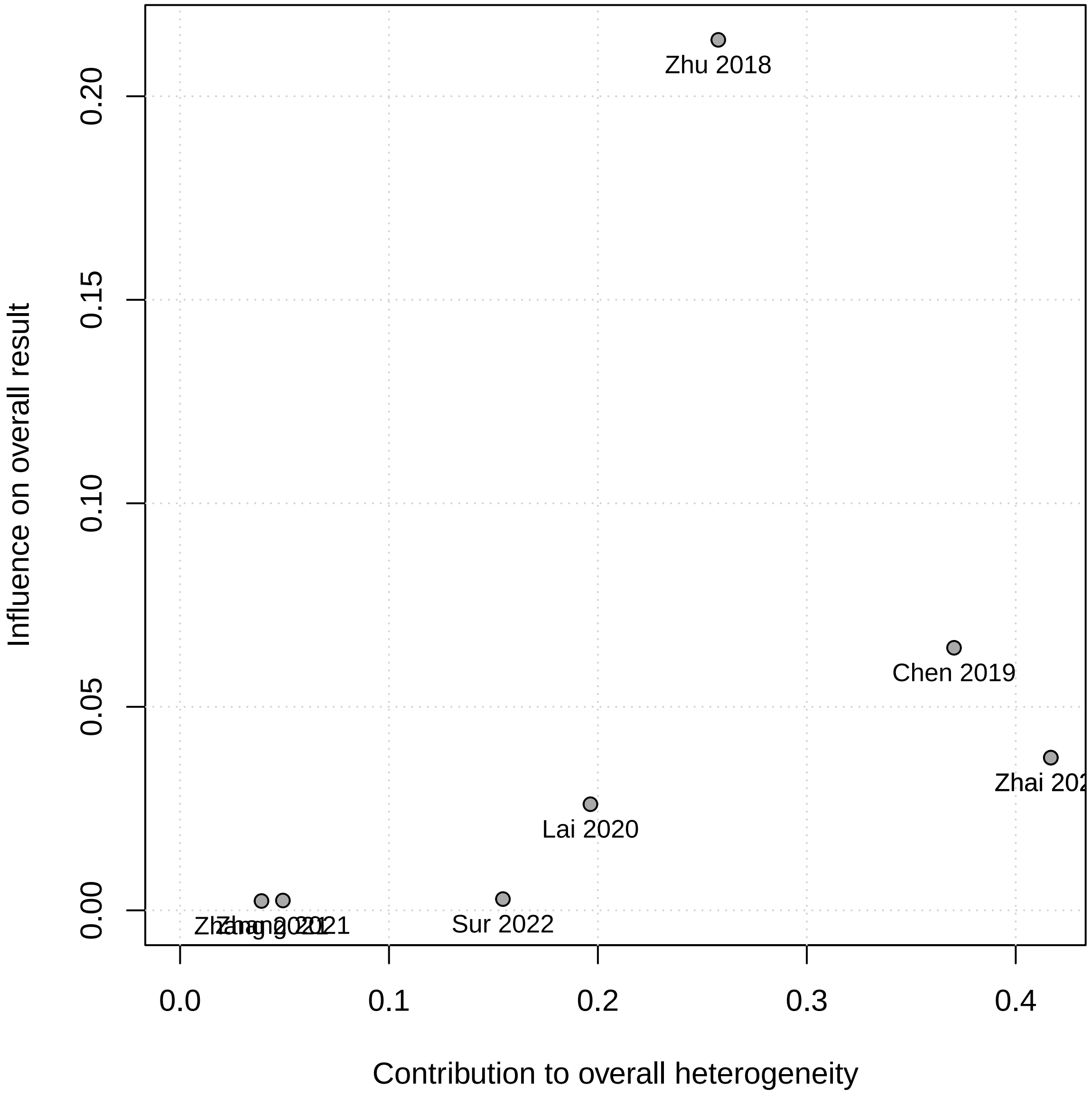

4.22.6 Leave one out

```
metainf(clav_i_ii_metabin)
```

```
## Influential analysis (common effect model)
##
##                                  RR           95%-CI p-value   tau^2     tau
## Omitting Sur 2022            0.5223 [0.3637; 0.7500]  0.0004  0.0000  0.0000
## Omitting Tang 2023           0.5274 [0.3686; 0.7545]  0.0005  0.0000  0.0000
## Omitting Chen 2019           0.5545 [0.3762; 0.8174]  0.0029  0.0000  0.0000
## Omitting Du 2019             0.5274 [0.3686; 0.7545]  0.0005  0.0000  0.0000
## Omitting Lai 2020            0.5110 [0.3487; 0.7488]  0.0006  0.0000  0.0000
## Omitting Zhu 2018            0.5914 [0.3637; 0.9617]  0.0342  0.0000  0.0000
## Omitting Zhang 2021          0.5321 [0.3687; 0.7680]  0.0008  0.0000  0.0000
## Omitting Zhang 2021          0.5225 [0.3624; 0.7535]  0.0005  0.0000  0.0000
## Omitting Lechevallier 2003   0.5274 [0.3686; 0.7545]  0.0005  0.0000  0.0000
## Omitting Huang 2023          0.5274 [0.3686; 0.7545]  0.0005  0.0000  0.0000
## Omitting Wu 2022             0.5274 [0.3686; 0.7545]  0.0005  0.0000  0.0000
## Omitting Ding 2023           0.5274 [0.3686; 0.7545]  0.0005  0.0000  0.0000
## Omitting Zhai 2023           0.5082 [0.3495; 0.7389]  0.0004  0.0000  0.0000
## Omitting Zhai 2023           0.5082 [0.3495; 0.7389]  0.0004  0.0000  0.0000
## Omitting Qian 2022           0.5274 [0.3686; 0.7545]  0.0005  0.0000  0.0000
## Omitting Zhang 2022          0.5274 [0.3686; 0.7545]  0.0005  0.0000  0.0000
## Omitting Deng 2022           0.5274 [0.3686; 0.7545]  0.0005  0.0000  0.0000
## Omitting AlSmadi 2019        0.5274 [0.3686; 0.7545]  0.0005  0.0000  0.0000
##
## Pooled estimate              0.5274 [0.3686; 0.7545]  0.0005  0.0000  0.0000
##                                I^2
## Omitting Sur 2022             0.0%
## Omitting Tang 2023            0.0%
## Omitting Chen 2019            0.0%
## Omitting Du 2019              0.0%
## Omitting Lai 2020             0.0%
## Omitting Zhu 2018             0.0%
## Omitting Zhang 2021           0.0%
## Omitting Zhang 2021           0.0%
## Omitting Lechevallier 2003    0.0%
## Omitting Huang 2023           0.0%
## Omitting Wu 2022              0.0%
## Omitting Ding 2023            0.0%
## Omitting Zhai 2023            0.0%
## Omitting Zhai 2023            0.0%
## Omitting Qian 2022            0.0%
## Omitting Zhang 2022           0.0%
## Omitting Deng 2022            0.0%
## Omitting AlSmadi 2019         0.0%
##
## Pooled estimate               0.0%
##
## Details on meta-analytical method:
## - Mantel-Haenszel method
## - Restricted maximum-likelihood estimator for tau^2
```

4.23 Clavien III-V

4.23.1 Meta-analysis

```
clav_iii_v_metabin <- metabin(data = suction_data,
                             event.c = clav_iii_v_n_control,
                           n.c = sample_size_control,
                           event.e = clav_iii_v_n_suction,
                           n.e = sample_size_suction,
                           studlab = author_year)
clav_iii_v_metabin
```

```
## Number of studies: k = 3
## Number of observations: o = 2311
## Number of events: e = 10
##
##                           RR           95%-CI     z p-value
## Common effect model  0.5631 [0.1633; 1.9422] -0.91  0.3633
## Random effects model 0.5735 [0.1630; 2.0175] -0.87  0.3863
##
## Quantifying heterogeneity:
##  tau^2 = 0 [0.0000; 9.8745]; tau = 0 [0.0000; 3.1424]
##  I^2 = 0.0% [0.0%; 89.6%]; H = 1.00 [1.00; 3.10]
##
## Test of heterogeneity:
##     Q d.f. p-value
##  0.34    2  0.8426
##
## Details on meta-analytical method:
## - Mantel-Haenszel method
## - Inverse variance method
## - Restricted maximum-likelihood estimator for tau^2
## - Q-Profile method for confidence interval of tau^2 and tau
## - Continuity correction of 0.5 in studies with zero cell frequencies
```

4.23.2 Forest plot

```
forest(clav_iii_v_metabin,
       sortvar = TE)
```

Study	Experimental Events	Experimental Total	Control Events	Control Total	RR	95%−CI	Weight (common)	Weight (random)
Chen 2019	0	44	1	45	0.34	[0.01; 8.15]	21.8%	15.7%
Zhu 2018	2	165	4	165	0.50	[0.09; 2.69]	58.7%	55.8%
Lai 2020	1	28	2	56	1.00	[0.09; 10.56]	19.6%	28.5%
Sur 2022	0	9	0	8			0.0%	0.0%
Tang 2023	.	86	.	87			0.0%	0.0%
Du 2019	.	62	.	60			0.0%	0.0%
Zhang 2021	0	56	0	50			0.0%	0.0%
Zhang 2021	0	56	0	54			0.0%	0.0%
Lechevallier 2003	.	11	.	14			0.0%	0.0%
Huang 2023	.	103	.	103			0.0%	0.0%
Wu 2022	.	76	.	82			0.0%	0.0%
Ding 2023	0	138	0	61			0.0%	0.0%
Zhai 2023	0	60	0	60			0.0%	0.0%
Zhai 2023	0	60	0	60			0.0%	0.0%
Qian 2022	.	81	.	81			0.0%	0.0%
Zhang 2022	0	30	0	30			0.0%	0.0%
Deng 2022	.	57	.	70			0.0%	0.0%
AlSmadi 2019	0	43	0	60			0.0%	0.0%
**Common effect model**		**1165**		**1146**	**0.56**	**[0.16; 1.94]**	**100.0%**	**−−**
**Random effects model**					**0.57**	**[0.16; 2.02]**	**−−**	**100.0%**

Heterogeneity: $I^2 = 0\%$, $\tau^2 = 0$, $p = 0.84$

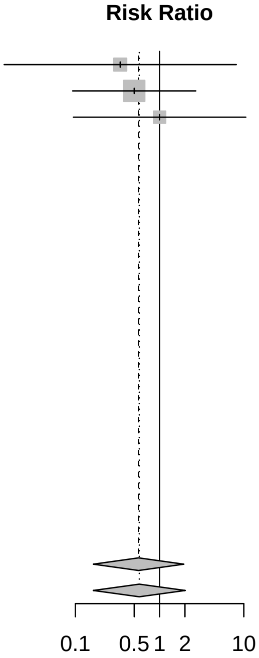

4.23.3 Trim and Fill

```r
trimfill(clav_iii_v_metabin)
```

```
## Warning in trimfill.meta(clav_iii_v_metabin): 15 observation(s) dropped due to
## missing values
```

```
## Number of studies: k = 3 (with 0 added studies)
## Number of observations: o = 503
## Number of events: e = 10
##
##                             RR           95%-CI     z p-value
## Random effects model 0.5735 [0.1630; 2.0175] -0.87  0.3863
##
## Quantifying heterogeneity:
##  tau^2 = 0 [0.0000; 9.8745]; tau = 0 [0.0000; 3.1424]
##  I^2 = 0.0% [0.0%; 89.6%]; H = 1.00 [1.00; 3.10]
##
## Test of heterogeneity:
##     Q d.f. p-value
##  0.34    2  0.8426
##
## Details on meta-analytical method:
## - Inverse variance method
## - Restricted maximum-likelihood estimator for tau^2
## - Q-Profile method for confidence interval of tau^2 and tau
## - Trim-and-fill method to adjust for funnel plot asymmetry (L-estimator)
```

```r
forest(trimfill(clav_iii_v_metabin),
       sortvar = TE)
```

```
## Warning in trimfill.meta(clav_iii_v_metabin): 15 observation(s) dropped due to
## missing values
```

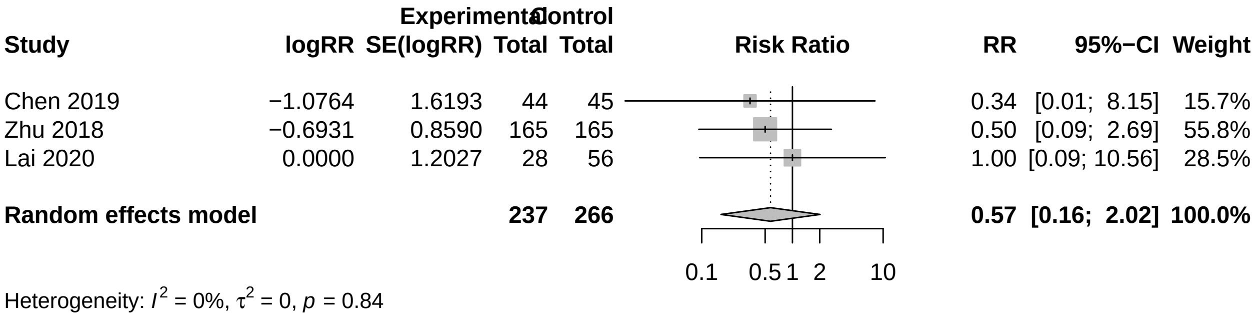

4.23.4 Funnel plot

```
funnel(trimfill(clav_iii_v_metabin))
```

```
## Warning in trimfill.meta(clav_iii_v_metabin): 15 observation(s) dropped due to
## missing values
```

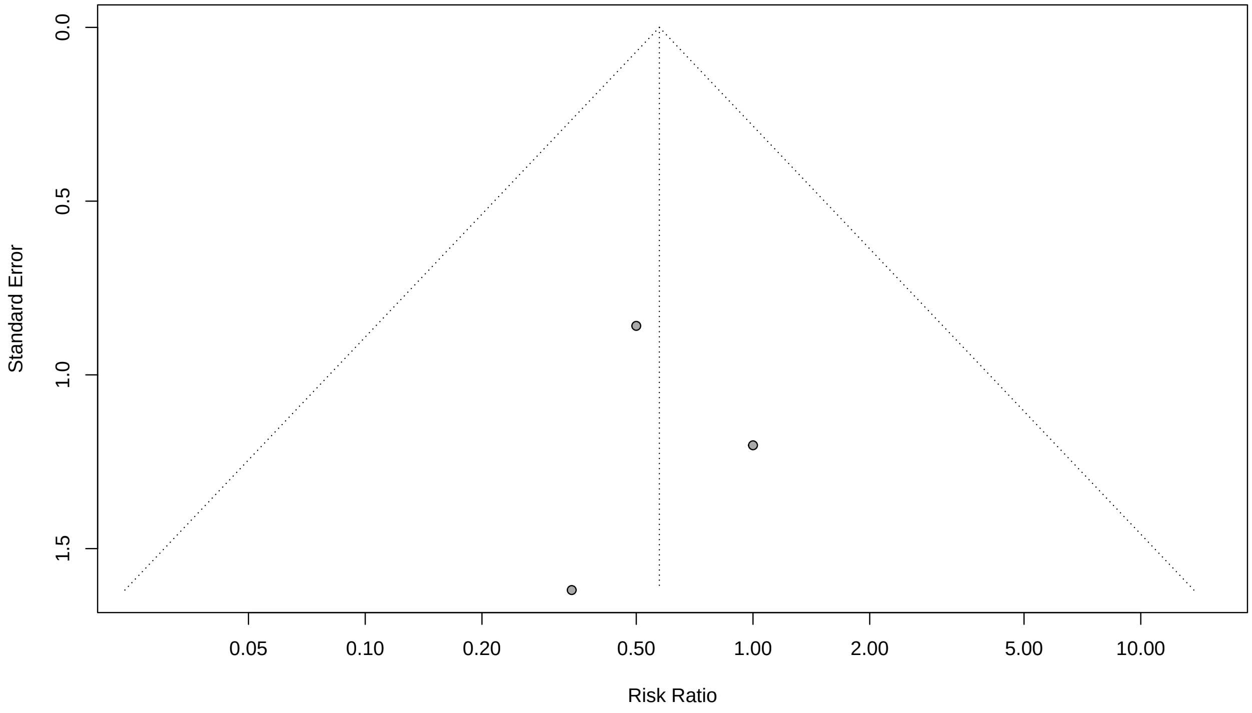

4.23.5 Baujat

```
baujat(clav_iii_v_metabin, pos = 1)
```

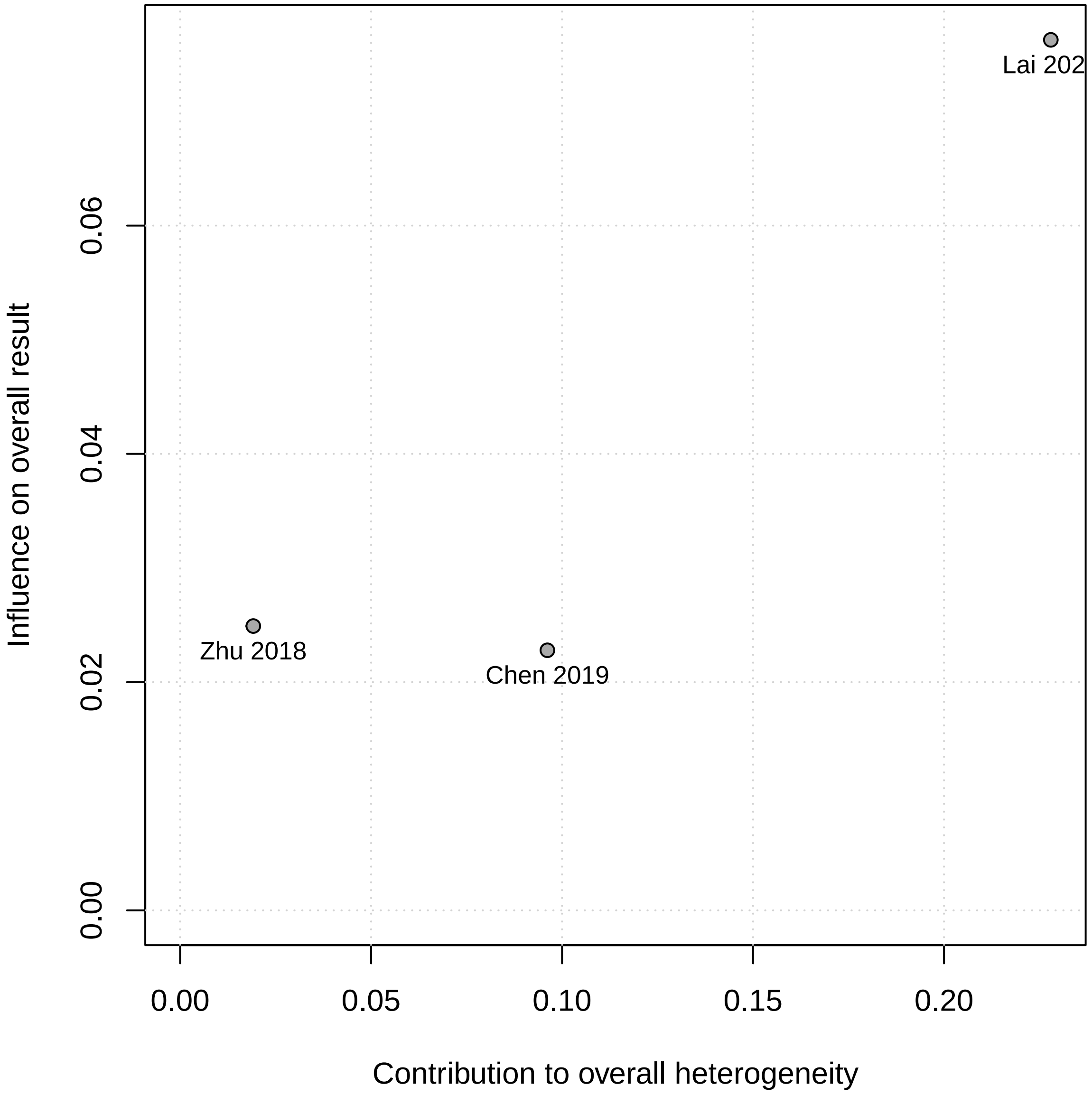

4.23.6 Leave one out

```
metainf(clav_iii_v_metabin)
```

```
## Influential analysis (common effect model)
##
##                                  RR           95%-CI p-value   tau^2     tau
## Omitting Sur 2022            0.5631 [0.1633; 1.9422]  0.3633  0.0000  0.0000
## Omitting Tang 2023           0.5631 [0.1633; 1.9422]  0.3633  0.0000  0.0000
## Omitting Chen 2019           0.6250 [0.1615; 2.4186]  0.4960  0.0000  0.0000
## Omitting Du 2019             0.5631 [0.1633; 1.9422]  0.3633  0.0000  0.0000
## Omitting Lai 2020            0.4569 [0.1037; 2.0140]  0.3007  0.0000  0.0000
## Omitting Zhu 2018            0.6528 [0.1042; 4.0882]  0.6487  0.0000  0.0000
## Omitting Zhang 2021          0.5631 [0.1633; 1.9422]  0.3633  0.0000  0.0000
## Omitting Zhang 2021          0.5631 [0.1633; 1.9422]  0.3633  0.0000  0.0000
## Omitting Lechevallier 2003   0.5631 [0.1633; 1.9422]  0.3633  0.0000  0.0000
## Omitting Huang 2023          0.5631 [0.1633; 1.9422]  0.3633  0.0000  0.0000
## Omitting Wu 2022             0.5631 [0.1633; 1.9422]  0.3633  0.0000  0.0000
## Omitting Ding 2023           0.5631 [0.1633; 1.9422]  0.3633  0.0000  0.0000
## Omitting Zhai 2023           0.5631 [0.1633; 1.9422]  0.3633  0.0000  0.0000
## Omitting Zhai 2023           0.5631 [0.1633; 1.9422]  0.3633  0.0000  0.0000
## Omitting Qian 2022           0.5631 [0.1633; 1.9422]  0.3633  0.0000  0.0000
## Omitting Zhang 2022          0.5631 [0.1633; 1.9422]  0.3633  0.0000  0.0000
## Omitting Deng 2022           0.5631 [0.1633; 1.9422]  0.3633  0.0000  0.0000
## Omitting AlSmadi 2019        0.5631 [0.1633; 1.9422]  0.3633  0.0000  0.0000
##
## Pooled estimate              0.5631 [0.1633; 1.9422]  0.3633  0.0000  0.0000
##                                I^2
## Omitting Sur 2022             0.0%
## Omitting Tang 2023            0.0%
## Omitting Chen 2019            0.0%
## Omitting Du 2019              0.0%
## Omitting Lai 2020             0.0%
## Omitting Zhu 2018             0.0%
## Omitting Zhang 2021           0.0%
## Omitting Zhang 2021           0.0%
## Omitting Lechevallier 2003    0.0%
## Omitting Huang 2023           0.0%
## Omitting Wu 2022              0.0%
## Omitting Ding 2023            0.0%
## Omitting Zhai 2023            0.0%
## Omitting Zhai 2023            0.0%
## Omitting Qian 2022            0.0%
## Omitting Zhang 2022           0.0%
## Omitting Deng 2022            0.0%
## Omitting AlSmadi 2019         0.0%
##
## Pooled estimate               0.0%
##
## Details on meta-analytical method:
## - Mantel-Haenszel method
## - Restricted maximum-likelihood estimator for tau^2
```

5 Overall Summary Forest plots

```
overall <- cbind(
  "Outcome" = c(
    "Immediate SFR",
    "Final SFR",
    "Auxiliary Treatment",
    "Overall Complications",
    "Fever",
    "Infection",
    "Sepsis",
    "Abscess",
    "Haematoma",
    "Pain",
    "Stricture",
    "Embolisation",
    "Transfusion",
    "CD I",
    "CD II",
    "CD III",
    "CD IV",
    "CD I-II",
    "CD III-V",
    "Operative time",
    "VAS",
    "Length of Stay"
  ),
  "type" = c(
    "binary",
    "binary",
    "binary",
    "binary",
    "binary",
    "binary",
    "binary",
    "binary",
    "binary",
    "binary",
    "binary",
    "binary",
    "binary",
    "binary",
    "binary",
    "binary",
    "binary",
    "binary",
    "binary",
    "cont",
    "cont",
    "cont"
  ),
  "n_studies" = c(
    immediate_sfr_metabin$k,
```

```
  sfr_metabin$k,
aux_rx_metabin$k,
comp_metabin$k,
fever_metabin$k,
infection_metabin$k,
sepsis_metabin$k,
abscess_metabin$k,
haematoma_metabin$k,
pain_metabin$k,
stricture_metabin$k,
ir_embolisation_metabin$k,
transfusion_metabin$k,
clav_i_metabin$k,
clav_ii_metabin$k,
clav_iii_metabin$k,
clav_iv_metabin$k,
clav_i_ii_metabin$k,
clav_iii_v_metabin$k,
or_time_metacont$k,
vas_metacont$k,
los_metacont$k),
"es" = c(
  immediate_sfr_metabin$TE.random %>% exp(),
  sfr_metabin$TE.random %>% exp(),
aux_rx_metabin$TE.random %>% exp(),
comp_metabin$TE.random %>% exp(),
fever_metabin$TE.random %>% exp(),
infection_metabin$TE.random %>% exp(),
sepsis_metabin$TE.random %>% exp(),
abscess_metabin$TE.random %>% exp(),
haematoma_metabin$TE.random %>% exp(),
pain_metabin$TE.random %>% exp(),
stricture_metabin$TE.random %>% exp(),
ir_embolisation_metabin$TE.random %>% exp(),
transfusion_metabin$TE.random %>% exp(),
clav_i_metabin$TE.random %>% exp(),
clav_ii_metabin$TE.random %>% exp(),
clav_iii_metabin$TE.random %>% exp(),
clav_iv_metabin$TE.random %>% exp(),
clav_i_ii_metabin$TE.random %>% exp(),
clav_iii_v_metabin$TE.random %>% exp(),
or_time_metacont$TE.random,
vas_metacont$TE.random,
los_metacont$TE.random),
"lower_ci" = c(
  immediate_sfr_metabin$lower.random %>% exp(),
  sfr_metabin$lower.random %>% exp(),
aux_rx_metabin$lower.random %>% exp(),
comp_metabin$lower.random %>% exp(),
fever_metabin$lower.random %>% exp(),
infection_metabin$lower.random %>% exp(),
sepsis_metabin$lower.random %>% exp(),
abscess_metabin$lower.random %>% exp(),
```

```r
haematoma_metabin$lower.random %>% exp(),
pain_metabin$lower.random %>% exp(),
stricture_metabin$lower.random %>% exp(),
ir_embolisation_metabin$lower.random %>% exp(),
transfusion_metabin$lower.random %>% exp(),
clav_i_metabin$lower.random %>% exp(),
clav_ii_metabin$lower.random %>% exp(),
clav_iii_metabin$lower.random %>% exp(),
clav_iv_metabin$lower.random %>% exp(),
clav_i_ii_metabin$lower.random %>% exp(),
clav_iii_v_metabin$lower.random %>% exp(),
or_time_metacont$lower.random,
vas_metacont$lower.random,
los_metacont$lower.random),
"upper_ci" = c(immediate_sfr_metabin$upper.random %>% exp(),
               sfr_metabin$upper.random %>% exp(),
aux_rx_metabin$upper.random %>% exp(),
comp_metabin$upper.random %>% exp(),
fever_metabin$upper.random %>% exp(),
infection_metabin$upper.random %>% exp(),
sepsis_metabin$upper.random %>% exp(),
abscess_metabin$upper.random %>% exp(),
haematoma_metabin$upper.random %>% exp(),
pain_metabin$upper.random %>% exp(),
stricture_metabin$upper.random %>% exp(),
ir_embolisation_metabin$upper.random %>% exp(),
transfusion_metabin$upper.random %>% exp(),
clav_i_metabin$upper.random %>% exp(),
clav_ii_metabin$upper.random %>% exp(),
clav_iii_metabin$upper.random %>% exp(),
clav_iv_metabin$upper.random %>% exp(),
clav_i_ii_metabin$upper.random %>% exp(),
clav_iii_v_metabin$upper.random %>% exp(),
or_time_metacont$upper.random,
vas_metacont$upper.random,
los_metacont$upper.random),
"p" = c(
  immediate_sfr_metabin$pval.random,
  sfr_metabin$pval.random,
aux_rx_metabin$pval.random,
comp_metabin$pval.random,
fever_metabin$pval.random,
infection_metabin$pval.random,
sepsis_metabin$pval.random,
abscess_metabin$pval.random,
haematoma_metabin$pval.random,
pain_metabin$pval.random,
stricture_metabin$pval.random,
ir_embolisation_metabin$pval.random,
transfusion_metabin$pval.random,
clav_i_metabin$pval.random,
clav_ii_metabin$pval.random,
clav_iii_metabin$pval.random,
```

```r
clav_iv_metabin$pval.random,
clav_i_ii_metabin$pval.random,
clav_iii_v_metabin$pval.random,
or_time_metacont$pval.random,
vas_metacont$pval.random,
los_metacont$pval.random)) %>% as_tibble() %>% drop_na(es)

overall$es <- as.numeric(overall$es)
overall$es <- round(overall$es, digits = 2)

overall$lower_ci <- as.numeric(overall$lower_ci)
overall$lower_ci <- round(overall$lower_ci, digits = 2)

overall$upper_ci <- as.numeric(overall$upper_ci)
overall$upper_ci <- round(overall$upper_ci, digits = 2)

overall$p <- as.numeric(overall$p)
overall$p <- round(overall$p, digits = 2)
overall$p <- ifelse(overall$p<0.001, "<0.001", overall$p)
```

5.1 Summary Table of number of studies for each outcome included in meta-analysis

```
overall %>% subset(select = c(Outcome, n_studies)) %>% gt() %>% tab_header(title = "Summary table of Nu
```

Summary table of Number of Studies for Each Outcome Meta-Analyis

Outcome	Studies, n
Immediate SFR	9
Final SFR	17
Auxiliary Treatment	8
Overall Complications	17
Fever	13
Infection	14
Sepsis	2
Haematoma	1
Pain	3
Stricture	2
Embolisation	2
Transfusion	3
CD I	8
CD II	6
CD III	3
CD IV	1
CD I-II	8
CD III-V	3
Operative time	17
VAS	2
Length of Stay	9

5.2 Summary Forest plot of Continuous outcomes

5.2.1 Continuous Outcomes Table

md = mean difference lb = lower bound of 95% confidence interval ub = upper bound of 95% confidence interval tf = trim and fill

```
overall_continuous <-
  overall %>% subset(type ==  "cont") %>% subset(select = c(Outcome,
                                                              n_studies,
                                                              es,
                                                              lower_ci,
                                                              upper_ci,
                                                              p)) %>% as_tibble()
overall_continuous %>% gt() %>% tab_header(title = "Summary Table for Continuous Outcomes") %>% cols_me
```

Summary Table for Continuous Outcomes

Outcome	Studies, n	MD (95% CI)	p
Operative time	17	-2.32 (-7.07-2.44)	0.34
VAS	2	-1.20 (-2.93-0.52)	0.17
Length of Stay	9	-1.09 (-1.90–0.28)	0.01

5.2.2 Continuous Forest plot

```r
continuous_plot <- overall_continuous %>%
  forestplot(
    mean = es,
    lower = lower_ci,
    upper = upper_ci,
    labeltext = c(Outcome, n_studies, es, lower_ci, upper_ci, p),
  zero = 0,
  vertices = TRUE,
  title = "A. Forest plot of MA Outcomes for Continuous Outcomes",
  clip = c(-4, 2),
  xlab = "MD [95% CI]",
  graph.pos = 3
) %>% fp_set_style(
    box = c("black"),
    line = "black",
    txt_gp = fpTxtGp(
      ticks = gpar(fontfamily = "", cex = 1),
      xlab  = gpar(fontfamily = "", cex = 1)
    )) %>% fp_add_lines("steelblue") %>%
  fp_add_header("Outcome",
                "Studies, n",
                  "MD",
                "[ 95%", " CI ]",
                "P-Value")  %>% fp_decorate_graph(box = TRUE) %>% fp_set_zebra_style("#EFEFEF")
continuous_plot
```

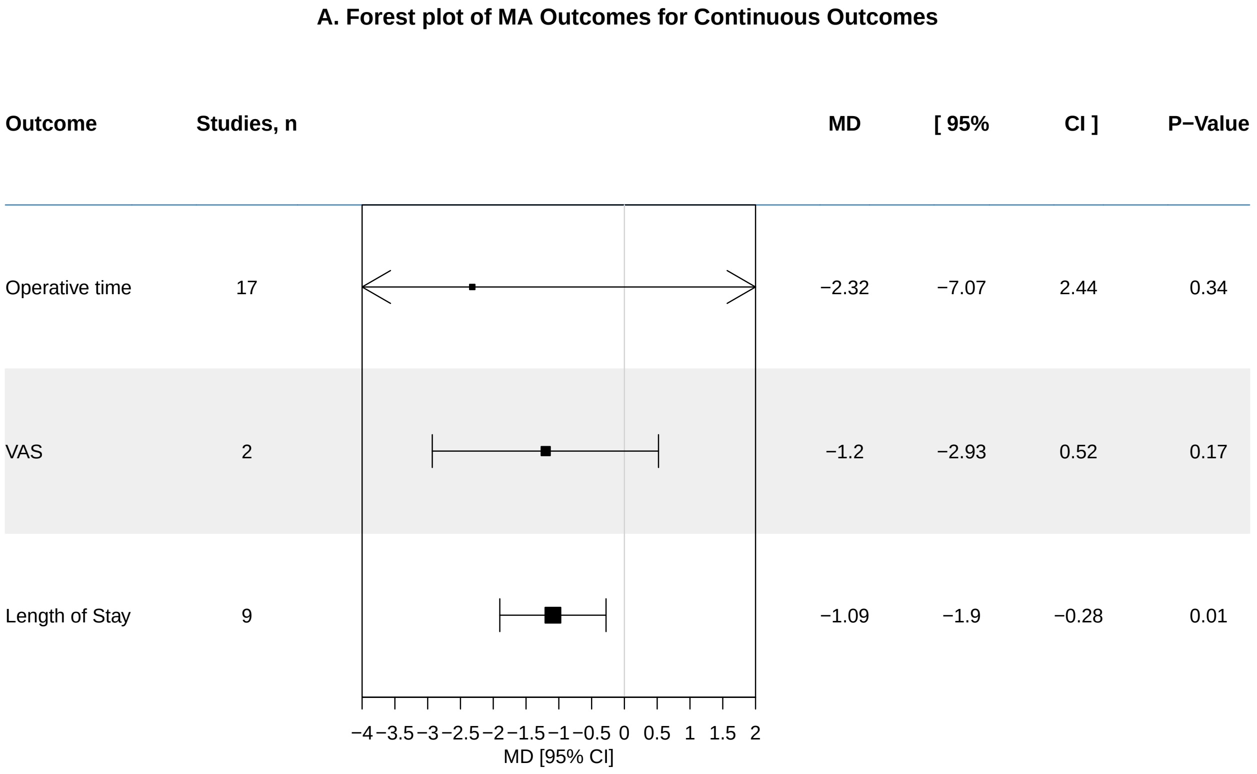

5.3 Summary Forest plot of Binary outcomes

5.3.1 Binary Outcomes Table

md = mean difference lb = lower bound of 95% confidence interval ub = upper bound of 95% confidence interval tf = trim and fill

```
overall_binary <-
  overall %>% subset(type ==  "binary") %>% subset(select = c(Outcome,
                                                            n_studies,
                                                            es,
                                                            lower_ci,
                                                            upper_ci,
                                                            p)) %>% as_tibble()
overall_binary %>% gt() %>% tab_header(title = "Summary Table for Binary Outcomes") %>% cols_merge(colu
```

Summary Table for Binary Outcomes

Outcome	Studies, n	RR (95% CI)	p
Immediate SFR	9	1.15 (0.99-1.34)	0.08
Final SFR	17	1.12 (1.05-1.19)	<0.001
Auxiliary Treatment	8	0.81 (0.31-2.10)	0.67
Overall Complications	17	0.44 (0.33-0.57)	<0.001
Fever	13	0.44 (0.30-0.64)	<0.001
Infection	14	0.43 (0.29-0.63)	<0.001
Sepsis	2	0.24 (0.07-0.75)	0.01
Haematoma	1	0.41 (0.02-9.84)	0.58
Pain	3	0.22 (0.08-0.59)	<0.001
Stricture	2	0.60 (0.03-12.28)	0.74
Embolisation	2	0.29 (0.03-2.55)	0.26
Transfusion	3	0.16 (0.03-0.88)	0.04
CD I	8	0.60 (0.40-0.90)	0.01
CD II	6	0.37 (0.16-0.88)	0.02
CD III	3	0.77 (0.16-3.67)	0.74
CD IV	1	0.33 (0.04-3.17)	0.34
CD I-II	8	0.53 (0.37-0.76)	<0.001
CD III-V	3	0.57 (0.16-2.02)	0.39

5.3.2 Binary Forest plot - Meta-Analysis

Reference = No Suction

```
binary_plot <- overall_binary %>%
  forestplot(
    mean = es,
    lower = lower_ci,
    upper = upper_ci,
    zero = 1,
    labeltext = c(Outcome, n_studies, es, lower_ci, upper_ci, p),
  vertices = TRUE,
  title = "A. Forest plot of MA Outcomes for Binary Outcomes",
  clip = c(-1.5, 1.5),
  xlab = "RR [95% CI]",
  graph.pos = 3
) %>% fp_set_style(
    box = c("black"),
    line = "black",
    txt_gp = fpTxtGp(
      ticks = gpar(fontfamily = "", cex = 1),
      xlab  = gpar(fontfamily = "", cex = 1)
    )) %>% fp_add_lines("steelblue") %>%
  fp_add_header("Outcome",
                "Studies, n",
                  "RR",
                "[ 95%", " CI ]",
                "P-Value")  %>% fp_decorate_graph(box = TRUE) %>% fp_set_zebra_style("#EFEFEF")
binary_plot
```

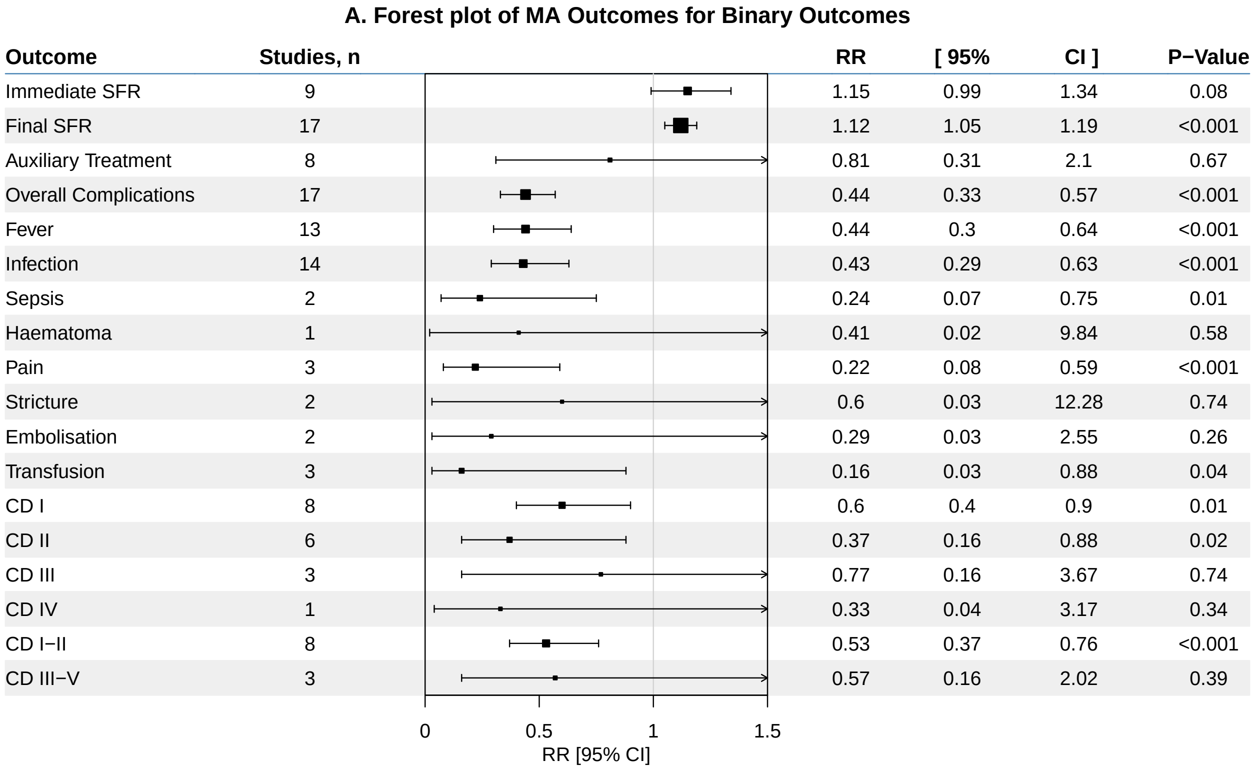

6 RCT only Meta-Analysis Outcomes

```
rct_only <- suction_data %>% subset(design == "RCT")
```

6.1 Immediate SFR

6.1.1 Meta-analysis

```
immediate_sfr_metabin <- metabin(data = suction_data,
                          event.c = sfr_immediate_n_control,
                        n.c = sample_size_control,
                        event.e = sfr_immediate_n_suction,
                        n.e = sample_size_suction,
                        studlab = author_year)
immediate_sfr_metabin
```

```
## Number of studies: k = 9
## Number of observations: o = 2311
## Number of events: e = 979
##
##                           RR           95%-CI    z  p-value
## Common effect model  1.1581 [1.0834; 1.2380] 4.31 < 0.0001
## Random effects model 1.1478 [0.9854; 1.3370] 1.77   0.0765
##
## Quantifying heterogeneity:
##  tau^2 = 0.0365 [0.0088; 0.2318]; tau = 0.1910 [0.0939; 0.4814]
##  I^2 = 74.6% [50.9%; 86.9%]; H = 1.99 [1.43; 2.76]
##
## Test of heterogeneity:
##      Q d.f. p-value
##  31.55    8  0.0001
##
## Details on meta-analytical method:
## - Mantel-Haenszel method
## - Inverse variance method
## - Restricted maximum-likelihood estimator for tau^2
## - Q-Profile method for confidence interval of tau^2 and tau
```

6.1.2 Forest plot

```r
forest(immediate_sfr_metabin,
       sortvar = TE)
```

Study	Experimental Events	Experimental Total	Control Events	Control Total	Risk Ratio	RR	95%−CI	Weight (common)	Weight (random)
Lai 2020	14	28	41	56		0.68	[0.46; 1.02]	6.1%	7.7%
Tang 2023	63	86	75	87		0.85	[0.73; 0.99]	16.6%	14.2%
Zhu 2018	136	165	118	165		1.15	[1.02; 1.30]	26.3%	15.1%
Qian 2022	70	81	58	81		1.21	[1.03; 1.42]	12.9%	14.0%
AlSmadi 2019	40	43	46	60		1.21	[1.03; 1.43]	8.6%	14.0%
Wu 2022	67	76	59	82		1.23	[1.05; 1.44]	12.6%	14.1%
Zhang 2021	18	56	13	54		1.34	[0.73; 2.45]	2.9%	4.6%
Huang 2023	81	103	52	103		1.56	[1.25; 1.93]	11.6%	12.5%
Zhang 2021	18	56	10	50		1.61	[0.82; 3.15]	2.4%	3.9%
Sur 2022	.	9	.	8				0.0%	0.0%
Chen 2019	.	44	.	45				0.0%	0.0%
Du 2019	.	62	.	60				0.0%	0.0%
Lechevallier 2003	.	11	.	14				0.0%	0.0%
Ding 2023	.	138	.	61				0.0%	0.0%
Zhai 2023	.	60	.	60				0.0%	0.0%
Zhai 2023	.	60	.	60				0.0%	0.0%
Zhang 2022	.	30	.	30				0.0%	0.0%
Deng 2022	.	57	.	70				0.0%	0.0%
**Common effect model**		**1165**		**1146**		**1.16**	**[1.08; 1.24]**	**100.0%**	**--**
**Random effects model**						**1.15**	**[0.99; 1.34]**	**--**	**100.0%**

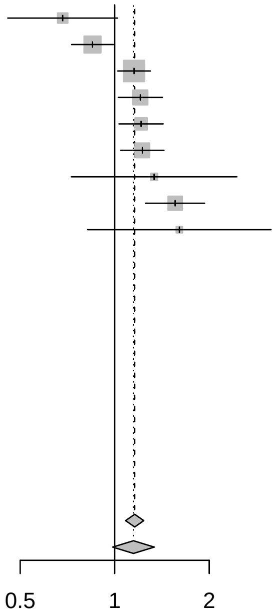

Heterogeneity: $I^2 = 75\%$, $\tau^2 = 0.0365$, $p < 0.01$

6.1.3 Trim and Fill

```r
trimfill(immediate_sfr_metabin)
```

```
## Warning in trimfill.meta(immediate_sfr_metabin): 9 observation(s) dropped due
## to missing values

## Number of studies: k = 11 (with 2 added studies)
## Number of observations: o = 1744
## Number of events: e = 1140
##
##                            RR           95%-CI    z p-value
## Random effects model 1.0855 [0.9289; 1.2685] 1.03  0.3019
##
## Quantifying heterogeneity:
##  tau^2 = 0.0471 [0.0138; 0.2196]; tau = 0.2170 [0.1173; 0.4686]
##  I^2 = 76.9% [58.8%; 87.1%]; H = 2.08 [1.56; 2.78]
##
## Test of heterogeneity:
##      Q d.f.  p-value
##  43.33   10 < 0.0001
##
## Details on meta-analytical method:
## - Inverse variance method
## - Restricted maximum-likelihood estimator for tau^2
## - Q-Profile method for confidence interval of tau^2 and tau
## - Trim-and-fill method to adjust for funnel plot asymmetry (L-estimator)
```

```r
forest(trimfill(immediate_sfr_metabin),
       sortvar = TE)
```

```
## Warning in trimfill.meta(immediate_sfr_metabin): 9 observation(s) dropped due
## to missing values
```

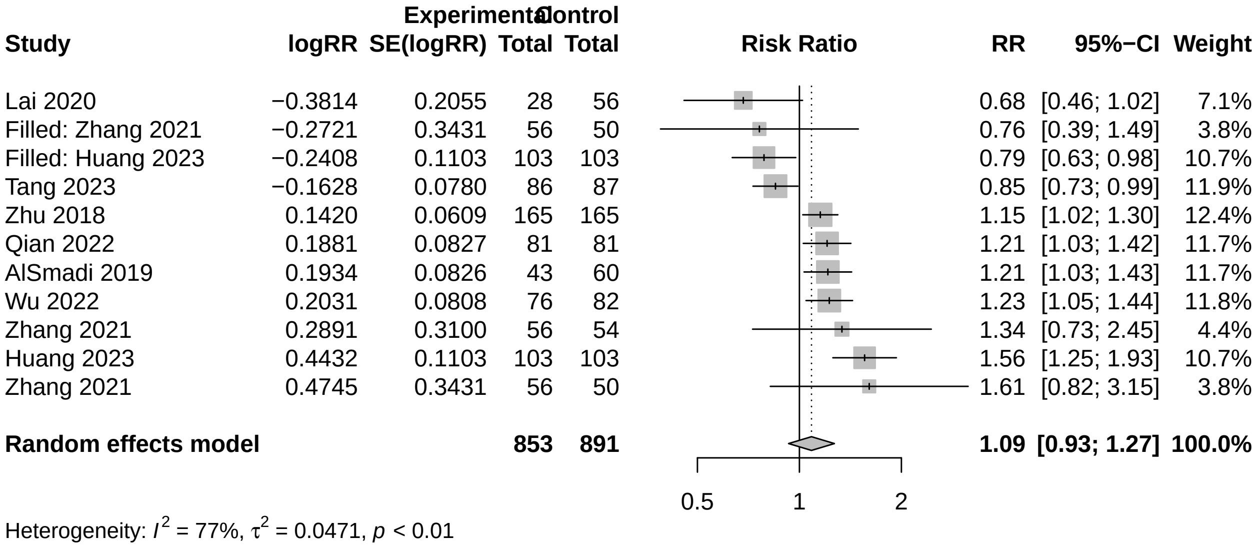

6.1.4 Funnel plot

```
funnel(trimfill(immediate_sfr_metabin))
```

```
## Warning in trimfill.meta(immediate_sfr_metabin): 9 observation(s) dropped due
## to missing values
```

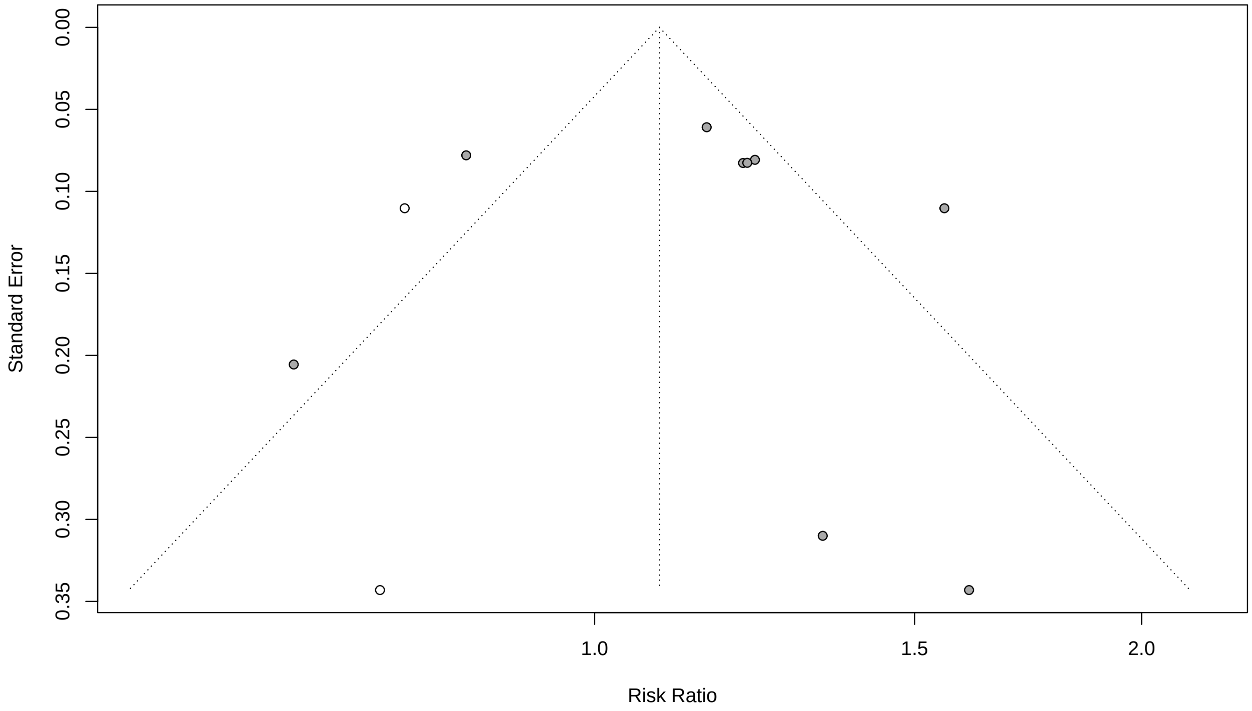

6.1.5 Baujat

```
baujat(immediate_sfr_metabin, pos = 1)
```

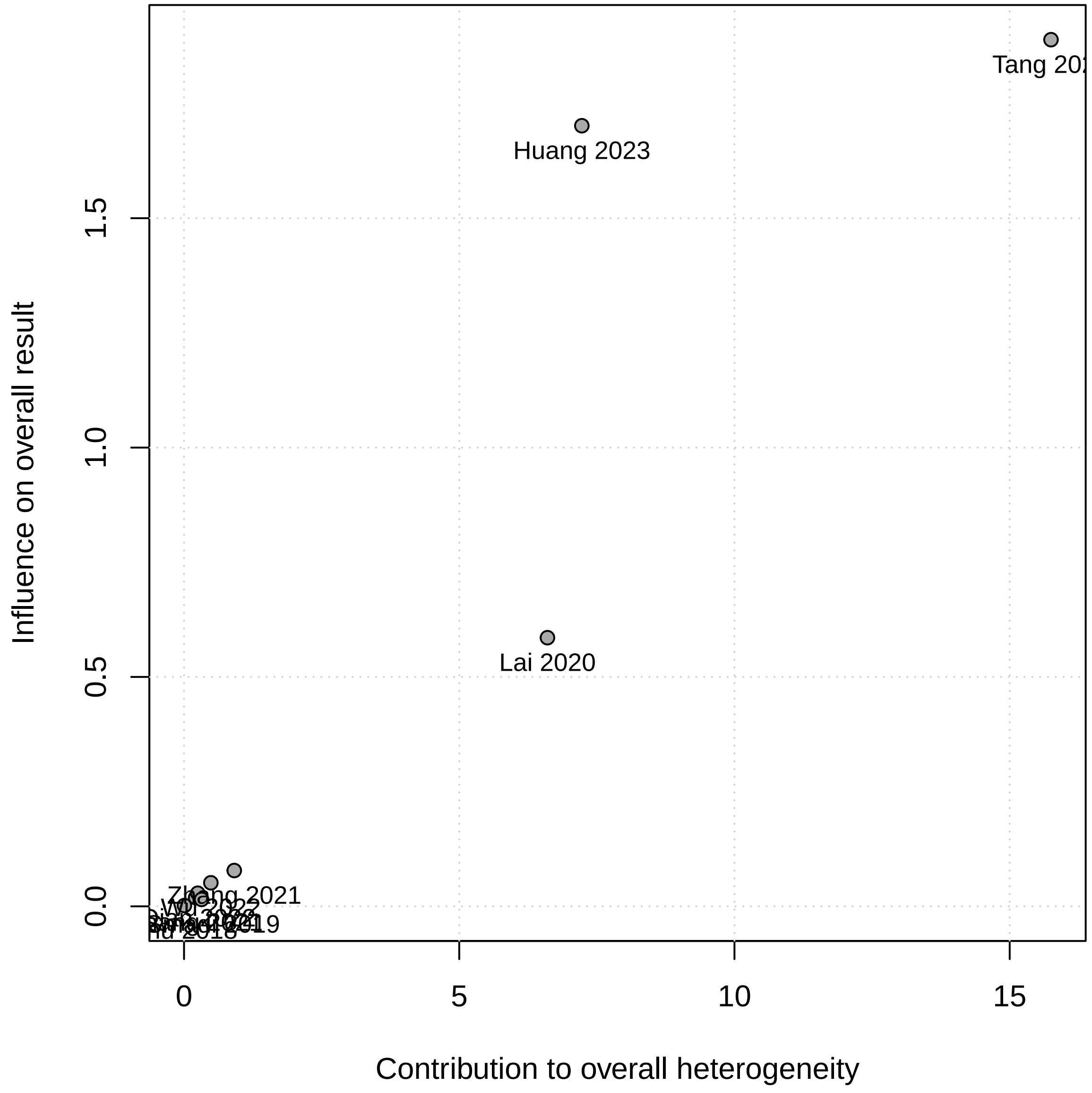

6.1.6 Leave one out

```
metainf(immediate_sfr_metabin)
```

```
## Influential analysis (common effect model)
##
##                                 RR           95%-CI  p-value  tau^2    tau
## Omitting Sur 2022           1.1581 [1.0834; 1.2380] < 0.0001  0.0365  0.1910
## Omitting Tang 2023          1.2195 [1.1329; 1.3128] < 0.0001  0.0076  0.0873
## Omitting Chen 2019          1.1581 [1.0834; 1.2380] < 0.0001  0.0365  0.1910
## Omitting Du 2019            1.1581 [1.0834; 1.2380] < 0.0001  0.0365  0.1910
## Omitting Lai 2020           1.1889 [1.1116; 1.2716] < 0.0001  0.0258  0.1607
## Omitting Zhu 2018           1.1601 [1.0710; 1.2565]   0.0003  0.0482  0.2196
## Omitting Zhang 2021         1.1473 [1.0741; 1.2254] < 0.0001  0.0376  0.1938
## Omitting Zhang 2021         1.1527 [1.0791; 1.2313] < 0.0001  0.0396  0.1990
## Omitting Lechevallier 2003  1.1581 [1.0834; 1.2380] < 0.0001  0.0365  0.1910
## Omitting Huang 2023         1.1057 [1.0315; 1.1853]   0.0046  0.0246  0.1568
## Omitting Wu 2022            1.1484 [1.0676; 1.2353]   0.0002  0.0464  0.2153
## Omitting Ding 2023          1.1581 [1.0834; 1.2380] < 0.0001  0.0365  0.1910
## Omitting Zhai 2023          1.1581 [1.0834; 1.2380] < 0.0001  0.0365  0.1910
## Omitting Zhai 2023          1.1581 [1.0834; 1.2380] < 0.0001  0.0365  0.1910
## Omitting Qian 2022          1.1508 [1.0700; 1.2377]   0.0002  0.0468  0.2164
## Omitting Zhang 2022         1.1581 [1.0834; 1.2380] < 0.0001  0.0365  0.1910
## Omitting Deng 2022          1.1581 [1.0834; 1.2380] < 0.0001  0.0365  0.1910
## Omitting AlSmadi 2019       1.1529 [1.0734; 1.2383] < 0.0001  0.0467  0.2160
##
## Pooled estimate             1.1581 [1.0834; 1.2380] < 0.0001  0.0365  0.1910
##                                I^2
## Omitting Sur 2022            74.6%
## Omitting Tang 2023           51.5%
## Omitting Chen 2019           74.6%
## Omitting Du 2019             74.6%
## Omitting Lai 2020            72.2%
## Omitting Zhu 2018            77.8%
## Omitting Zhang 2021          77.1%
## Omitting Zhang 2021          77.6%
## Omitting Lechevallier 2003   74.6%
## Omitting Huang 2023          69.4%
## Omitting Wu 2022             77.2%
## Omitting Ding 2023           74.6%
## Omitting Zhai 2023           74.6%
## Omitting Zhai 2023           74.6%
## Omitting Qian 2022           77.4%
## Omitting Zhang 2022          74.6%
## Omitting Deng 2022           74.6%
## Omitting AlSmadi 2019        77.4%
##
## Pooled estimate              74.6%
##
## Details on meta-analytical method:
## - Mantel-Haenszel method
## - Restricted maximum-likelihood estimator for tau^2
```

6.2 Final SFR

6.2.1 Meta-analysis

```
sfr_metabin <- metabin(data = rct_only,
                         event.c = sfr_final_n_control,
                       n.c = sample_size_control,
                       event.e = sfr_final_n_suction,
                       n.e = sample_size_suction,
                       studlab = author_year)
sfr_metabin
```

```
## Number of studies: k = 4
## Number of observations: o = 397
## Number of events: e = 340
##
##                          RR           95%-CI    z p-value
## Common effect model  1.1002 [1.0307; 1.1744] 2.87  0.0041
## Random effects model 1.1259 [0.9804; 1.2930] 1.68  0.0929
##
## Quantifying heterogeneity:
##  tau^2 = 0.0118 [0.0006; 0.1885]; tau = 0.1087 [0.0239; 0.4342]
##  I^2 = 73.8% [26.7%; 90.6%]; H = 1.95 [1.17; 3.27]
##
## Test of heterogeneity:
##      Q d.f. p-value
##  11.45    3  0.0095
##
## Details on meta-analytical method:
## - Mantel-Haenszel method
## - Inverse variance method
## - Restricted maximum-likelihood estimator for tau^2
## - Q-Profile method for confidence interval of tau^2 and tau
## - Continuity correction of 0.5 in studies with zero cell frequencies
```

6.2.2 Forest plot

```r
forest(sfr_metabin,
       sortvar = TE)
```

Study	Experimental Events	Experimental Total	Control Events	Control Total	Risk Ratio	RR	95%−CI	Weight (common)	Weight (random)
Tang 2023	81	86	83	87		0.99	[0.92; 1.06]	50.7%	38.1%
Zhang 2022	27	30	23	30		1.17	[0.93; 1.48]	14.1%	19.4%
Du 2019	62	62	49	60		1.22	[1.09; 1.38]	30.9%	32.2%
Sur 2022	9	9	6	8		1.31	[0.90; 1.90]	4.2%	10.4%
Lechevallier 2003	.	11	.	14				0.0%	0.0%
**Common effect model**		**198**		**199**		**1.10**	**[1.03; 1.17]**	**100.0%**	**−−**
**Random effects model**						**1.13**	**[0.98; 1.29]**	**−−**	**100.0%**

Heterogeneity: $I^2 = 74\%$, $\tau^2 = 0.0118$, $p < 0.01$

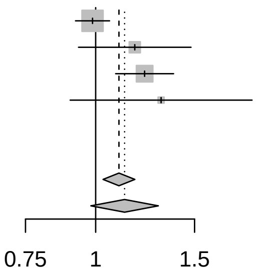

6.2.3 Trim and Fill

```
trimfill(sfr_metabin)
```

```
## Warning in trimfill.meta(sfr_metabin): 1 observation(s) dropped due to missing
## values

## Number of studies: k = 6 (with 2 added studies)
## Number of observations: o = 511
## Number of events: e = 466
##
##                             RR           95%-CI    z p-value
## Random effects model 1.0237 [0.8722; 1.2015] 0.29  0.7745
##
## Quantifying heterogeneity:
##  tau^2 = 0.0286 [0.0059; 0.2698]; tau = 0.1692 [0.0771; 0.5194]
##  I^2 = 81.8% [61.2%; 91.5%]; H = 2.34 [1.61; 3.42]
##
## Test of heterogeneity:
##      Q d.f.  p-value
##  27.46    5 < 0.0001
##
## Details on meta-analytical method:
## - Inverse variance method
## - Restricted maximum-likelihood estimator for tau^2
## - Q-Profile method for confidence interval of tau^2 and tau
## - Trim-and-fill method to adjust for funnel plot asymmetry (L-estimator)
```

```
forest(trimfill(sfr_metabin),
       sortvar = TE)
```

```
## Warning in trimfill.meta(sfr_metabin): 1 observation(s) dropped due to missing
## values
```

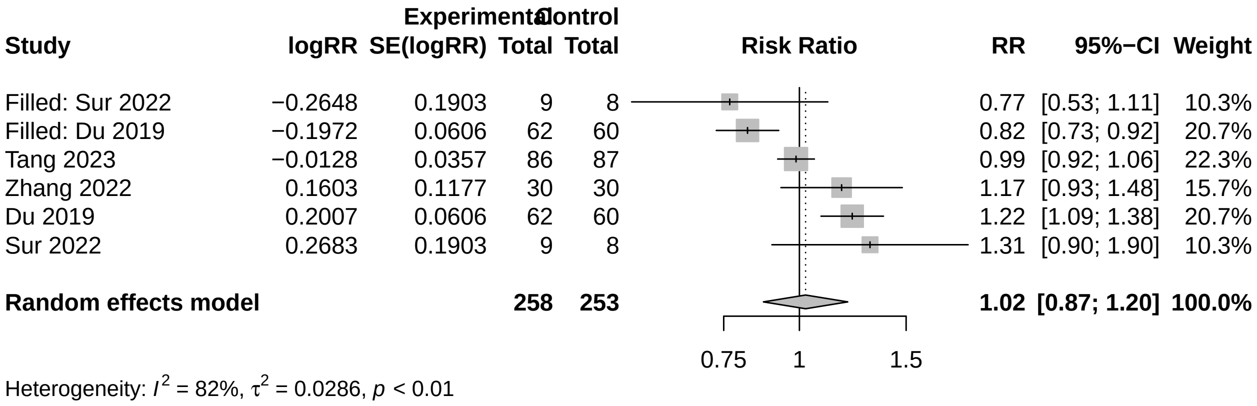

6.2.4 Funnel plot

```
funnel(trimfill(sfr_metabin))
```

```
## Warning in trimfill.meta(sfr_metabin): 1 observation(s) dropped due to missing
## values
```

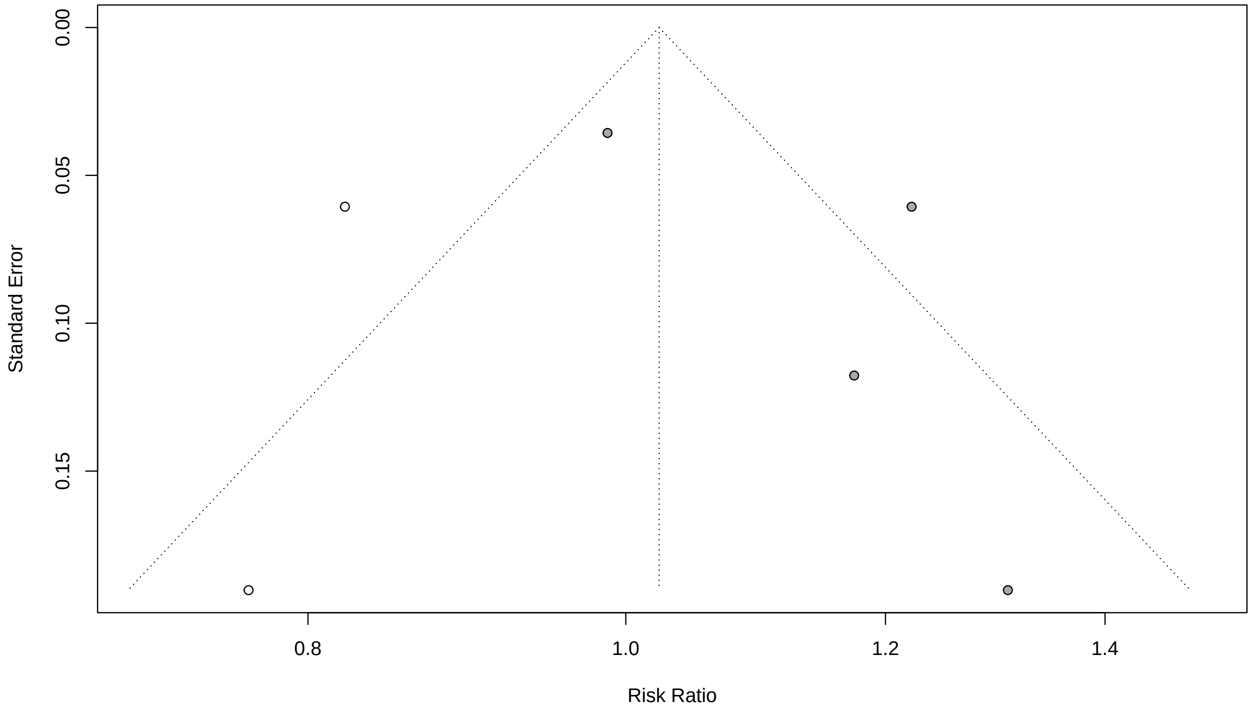

6.2.5 Baujat

```
baujat(sfr_metabin, pos = 1)
```

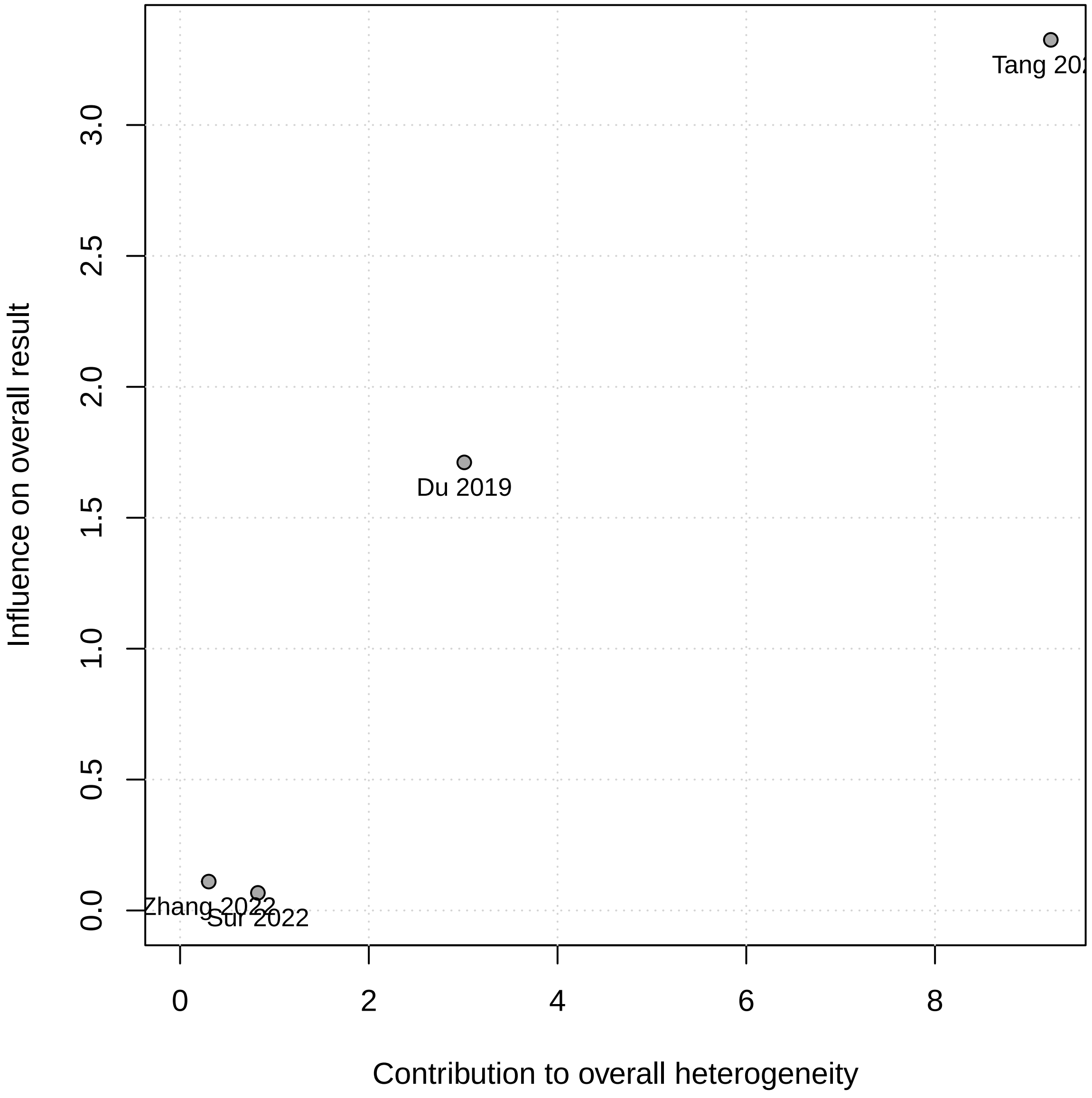

6.2.6 Leave one out

```
metainf(sfr_metabin)
```

```
## Influential analysis (common effect model)
##
##                                   RR           95%-CI p-value   tau^2    tau
## Omitting Sur 2022             1.0908 [1.0219; 1.1643]  0.0090  0.0125 0.1120
## Omitting Tang 2023            1.2165 [1.0920; 1.3553]  0.0004  0.0000 0.0000
## Omitting Du 2019              1.0454 [0.9685; 1.1285]  0.2549  0.0109 0.1046
## Omitting Lechevallier 2003    1.1002 [1.0307; 1.1744]  0.0041  0.0118 0.1087
## Omitting Zhang 2022           1.0881 [1.0191; 1.1617]  0.0115  0.0168 0.1296
##
## Pooled estimate               1.1002 [1.0307; 1.1744]  0.0041  0.0118 0.1087
##                                  I^2
## Omitting Sur 2022              80.3%
## Omitting Tang 2023              0.0%
## Omitting Du 2019               48.5%
## Omitting Lechevallier 2003     73.8%
## Omitting Zhang 2022            81.1%
##
## Pooled estimate                73.8%
##
## Details on meta-analytical method:
## - Mantel-Haenszel method
## - Restricted maximum-likelihood estimator for tau^2
```

6.3 OR time

6.3.1 Meta-analysis

```
or_time_metacont <- metacont(data = rct_only,
                             mean.c = or_time_min_mean_control,
                             sd.c = or_time_min_sd_control,
                             mean.e = or_time_min_mean_suction,
                             sd.e = or_time_min_sd_suction,
                             n.e = sample_size_suction,
                             n.c = sample_size_control,
                       studlab = author_year)
or_time_metacont
```

```
## Number of studies: k = 5
## Number of observations: o = 397
##
##                             MD               95%-CI     z  p-value
## Common effect model  -4.6093 [ -5.9159; -3.3026] -6.91 < 0.0001
## Random effects model -6.8746 [-18.7055;  4.9562] -1.14   0.2548
##
## Quantifying heterogeneity:
##  tau^2 = 158.1445 [43.6831; >1581.4445]; tau = 12.5755 [6.6093; >39.7674]
##  I^2 = 98.0% [96.9%; 98.7%]; H = 7.03 [5.64; 8.78]
##
## Test of heterogeneity:
##       Q d.f.  p-value
##  197.95    4 < 0.0001
##
## Details on meta-analytical method:
## - Inverse variance method
## - Restricted maximum-likelihood estimator for tau^2
## - Q-Profile method for confidence interval of tau^2 and tau
```

6.3.2 Forest plot

```r
forest(or_time_metacont,
       sortvar = TE)
```

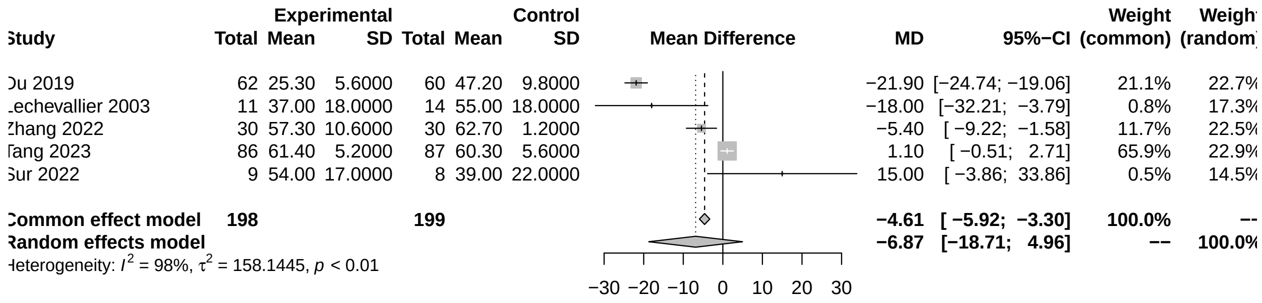

6.3.3 Trim and Fill

```
trimfill(or_time_metacont)
```

```
## Number of studies: k = 5 (with 0 added studies)
## Number of observations: o = 397
##
##                          MD             95%-CI     z p-value
## Random effects model -6.8746 [-18.7055; 4.9562] -1.14  0.2548
##
## Quantifying heterogeneity:
##  tau^2 = 158.1445 [43.6831; >1581.4445]; tau = 12.5755 [6.6093; >39.7674]
##  I^2 = 98.0% [96.9%; 98.7%]; H = 7.03 [5.64; 8.78]
##
## Test of heterogeneity:
##       Q d.f.  p-value
##  197.95    4 < 0.0001
##
## Details on meta-analytical method:
## - Inverse variance method
## - Restricted maximum-likelihood estimator for tau^2
## - Q-Profile method for confidence interval of tau^2 and tau
## - Trim-and-fill method to adjust for funnel plot asymmetry (L-estimator)
```

```
forest(trimfill(or_time_metacont),
       sortvar = TE)
```

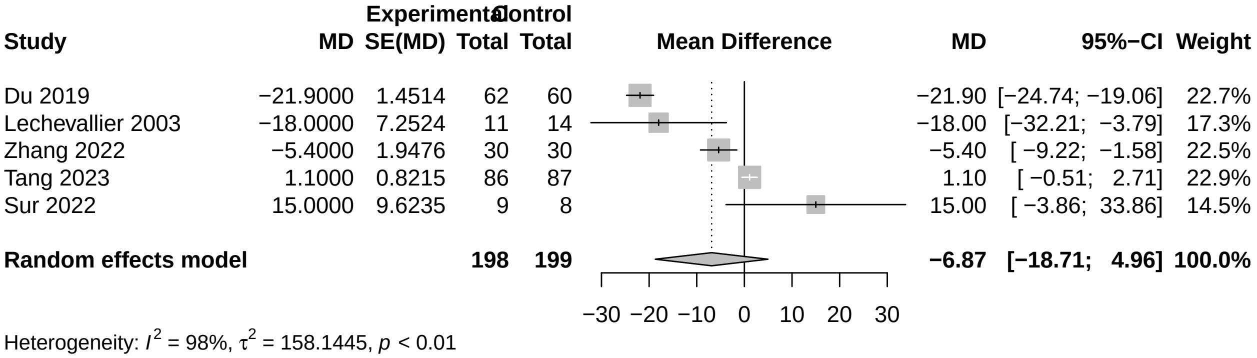

6.3.4 Funnel plot

```
funnel(trimfill(or_time_metacont))
```

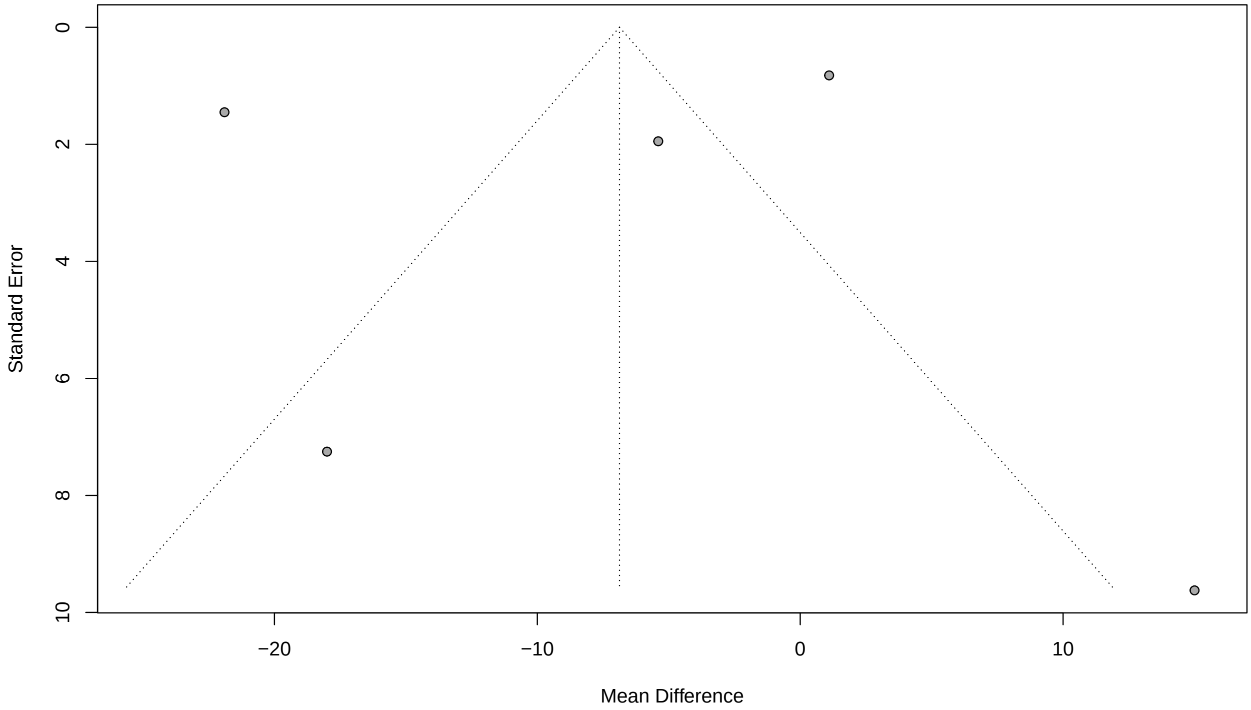

6.3.5 Baujat

```
baujat(or_time_metacont, pos = 1)
```

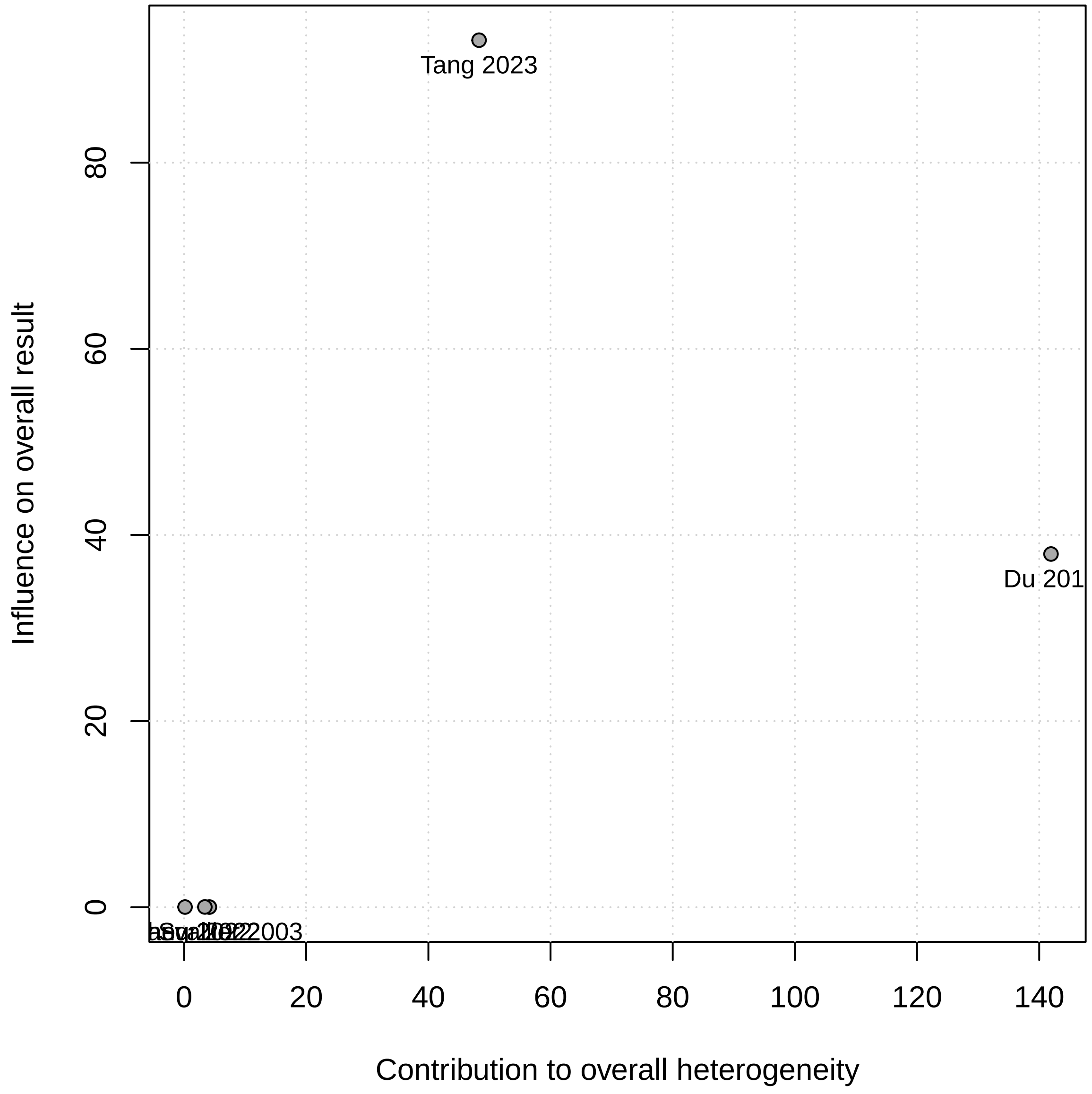

6.3.6 Leave one out

```r
metainf(or_time_metacont)
```

```
## Influential analysis (common effect model)
##
##                                    MD              95%-CI  p-value    tau^2
## Omitting Sur 2022             -4.7038 [ -6.0136;  -3.3940] < 0.0001  114.2692
## Omitting Tang 2023           -15.6222 [-17.8585; -13.3860] < 0.0001  190.4982
## Omitting Du 2019               0.0147 [ -1.4563;   1.4858]   0.9844   77.7955
## Omitting Lechevallier 2003    -4.4951 [ -5.8074;  -3.1829] < 0.0001  184.0636
## Omitting Zhang 2022           -4.5043 [ -5.8950;  -3.1136] < 0.0001  233.2375
##
## Pooled estimate               -4.6093 [ -5.9159;  -3.3026] < 0.0001  158.1445
##                                   tau    I^2
## Omitting Sur 2022             10.6897  98.5%
## Omitting Tang 2023            13.8021  94.7%
## Omitting Du 2019               8.8202  83.4%
## Omitting Lechevallier 2003    13.5670  98.5%
## Omitting Zhang 2022           15.2721  98.5%
##
## Pooled estimate               12.5755  98.0%
##
## Details on meta-analytical method:
## - Inverse variance method
## - Restricted maximum-likelihood estimator for tau^2
```

6.4 Auxiliary Treatments

6.4.1 Meta-analysis

```
aux_rx_metabin <- metabin(data = rct_only,
                         event.c = auxillary_tx_n_control,
                       n.c = sample_size_control,
                       event.e = auxillary_tx_n_suction,
                       n.e = sample_size_suction,
                       studlab = author_year)
aux_rx_metabin
```

```
## Number of studies: k = 1
## Number of observations: o = 397
## Number of events: e = 7
##
##                           RR           95%-CI     z p-value
## Common effect model  0.0646 [0.0038; 1.1059] -1.89  0.0587
## Random effects model 0.0645 [0.0038; 1.1054] -1.89  0.0586
##
## Quantifying heterogeneity:
##  tau^2 = NA; tau = NA; I^2 = NA; H = NA
##
## Details on meta-analytical method:
## - Mantel-Haenszel method
## - Inverse variance method
## - Restricted maximum-likelihood estimator for tau^2
## - Continuity correction of 0.5 in studies with zero cell frequencies
```

6.4.2 Forest plot

```r
forest(aux_rx_metabin,
       sortvar = TE)
```

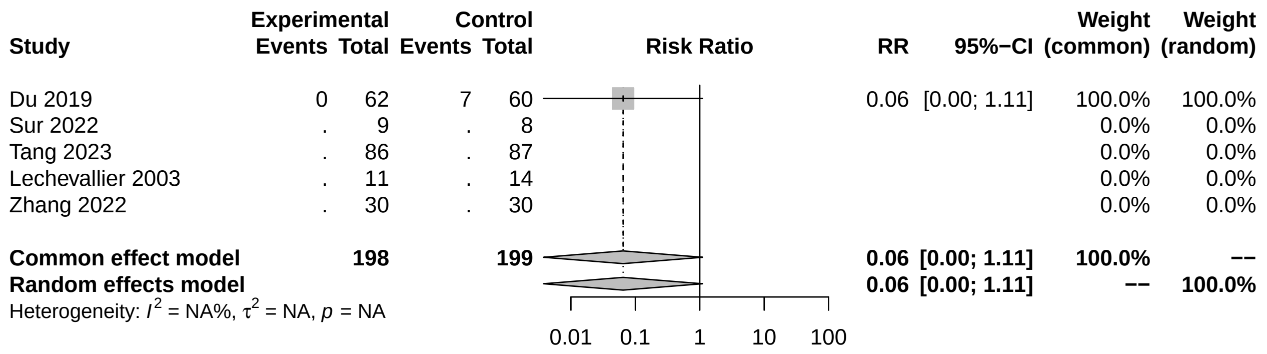

6.4.3 Trim and Fill

```
#trimfill(aux_rx_metabin)
#forest(trimfill(aux_rx_metabin),
#       sortvar = TE)
```

6.4.4 Funnel plot

```r
funnel((aux_rx_metabin))
```

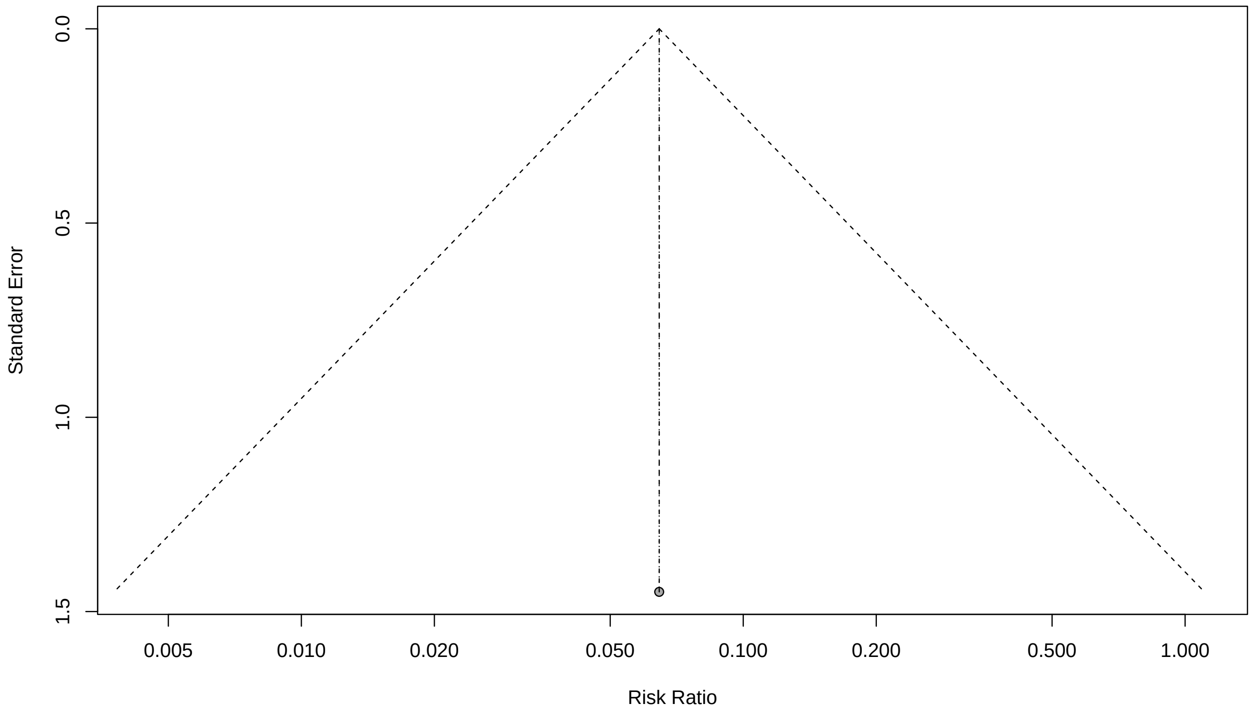

6.4.5 Baujat

```
baujat(aux_rx_metabin, pos = 1)
```

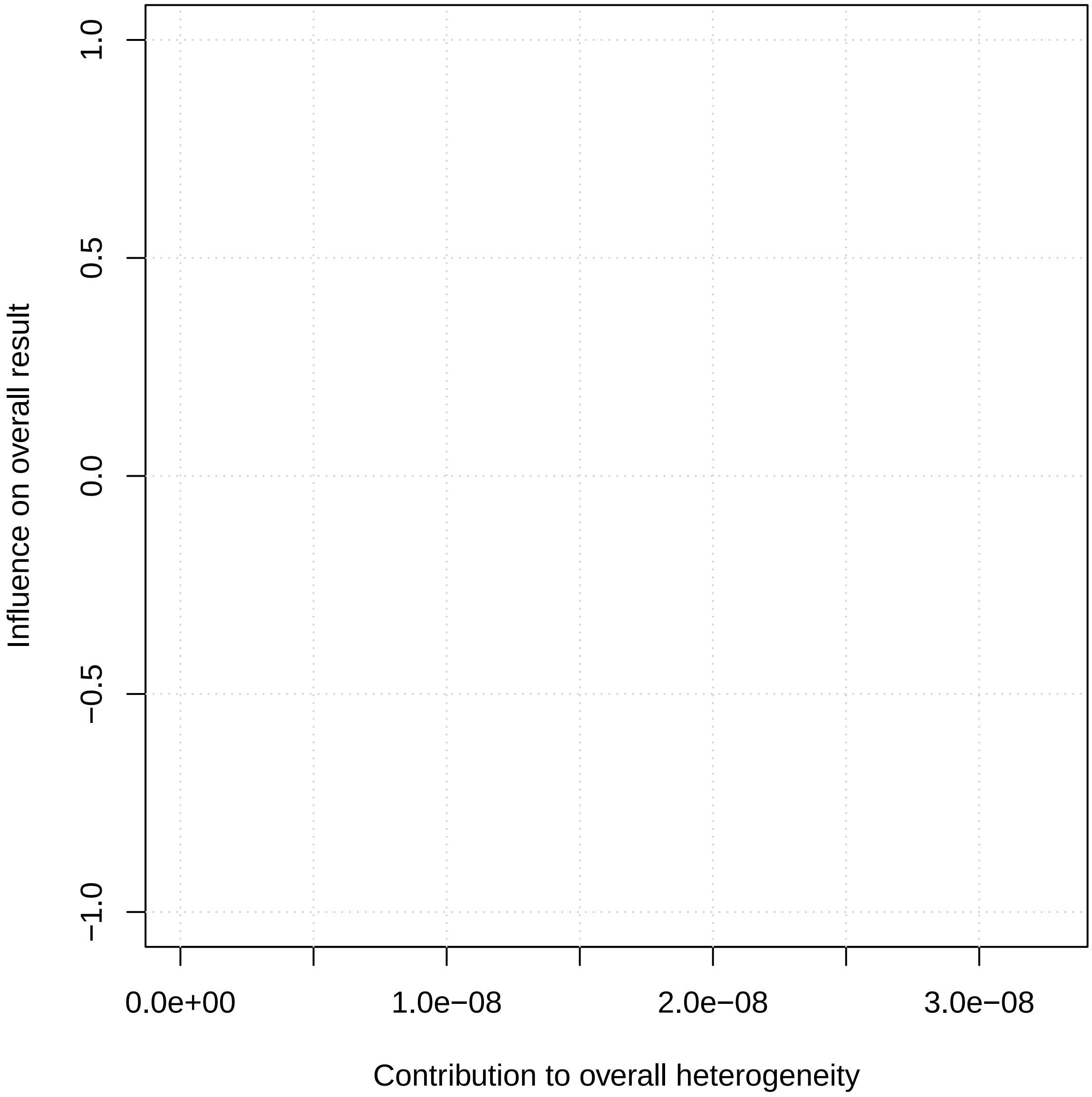

6.4.6 Leave one out

```r
metainf(aux_rx_metabin)
```

```
## Influential analysis (common effect model)
##
##                                  RR           95%-CI p-value tau^2 tau I^2
## Omitting Sur 2022            0.0646 [0.0038; 1.1059]  0.0587
## Omitting Tang 2023           0.0646 [0.0038; 1.1059]  0.0587
## Omitting Du 2019
## Omitting Lechevallier 2003   0.0646 [0.0038; 1.1059]  0.0587
## Omitting Zhang 2022          0.0646 [0.0038; 1.1059]  0.0587
##
## Pooled estimate              0.0646 [0.0038; 1.1059]  0.0587
##
## Details on meta-analytical method:
## - Mantel-Haenszel method
## - Restricted maximum-likelihood estimator for tau^2
```

6.5 VAS

6.5.1 Meta-analysis

```
vas_metacont <- metacont(data = rct_only,
                            mean.c = vas_score_mean_control,
                            sd.c = vas_score_sd_control,
                            mean.e = vas_score_mean_suction,
                            sd.e = vas_score_sd_suction,
                            n.e = sample_size_suction,
                            n.c = sample_size_control,
                      studlab = author_year)
vas_metacont
```

```
## Number of studies: k = 1
## Number of observations: o = 397
##
##                           MD             95%-CI     z p-value
## Common effect model  -0.3400 [-0.6488; -0.0312] -2.16  0.0309
## Random effects model -0.3400 [-0.6488; -0.0312] -2.16  0.0309
##
## Quantifying heterogeneity:
##  tau^2 = NA; tau = NA; I^2 = NA; H = NA
##
## Details on meta-analytical method:
## - Inverse variance method
## - Restricted maximum-likelihood estimator for tau^2
```

6.5.2 Forest plot

```r
forest(vas_metacont,
       sortvar = TE)
```

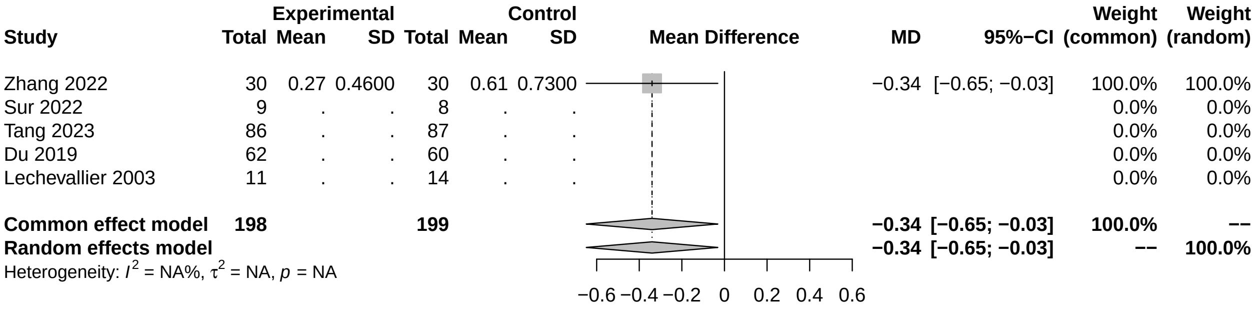

6.5.3 Trim and Fill

```r
#trimfill(vas_metacont)
#forest(trimfill(vas_metacont))
```

6.5.4 Funnel plot

```
funnel((vas_metacont))
```

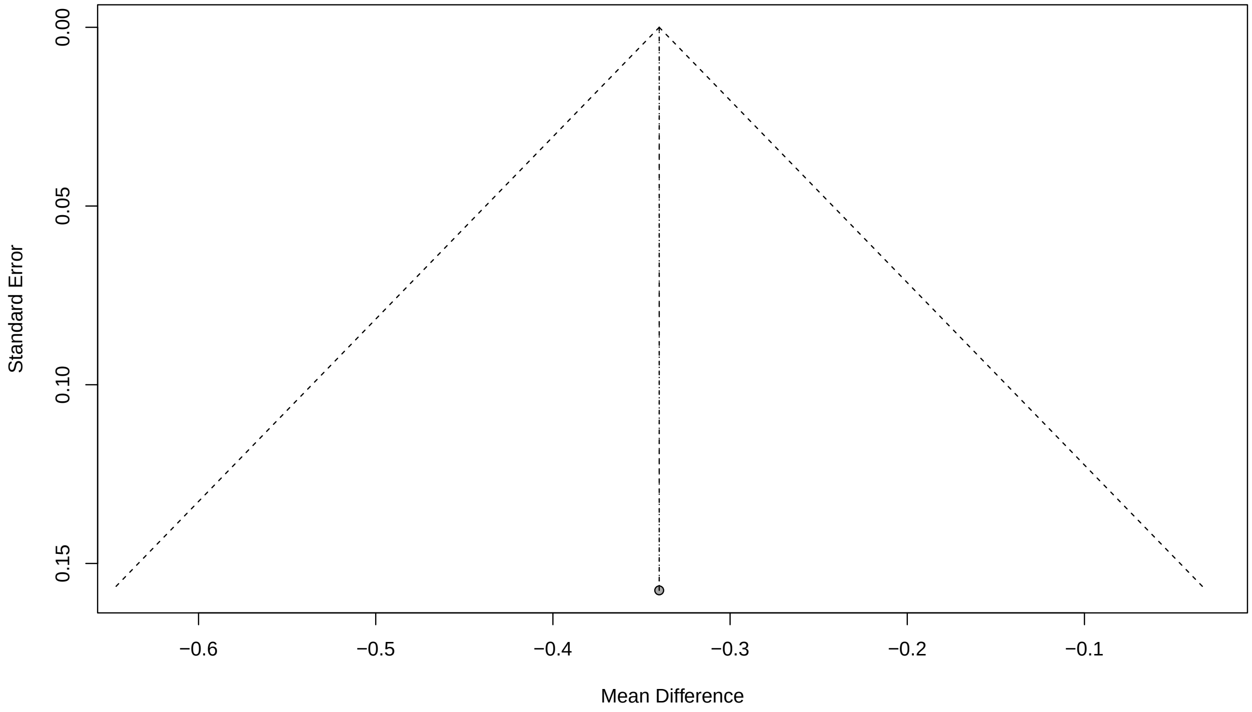

6.5.5 Baujat

```
baujat(vas_metacont, pos = 1)
```

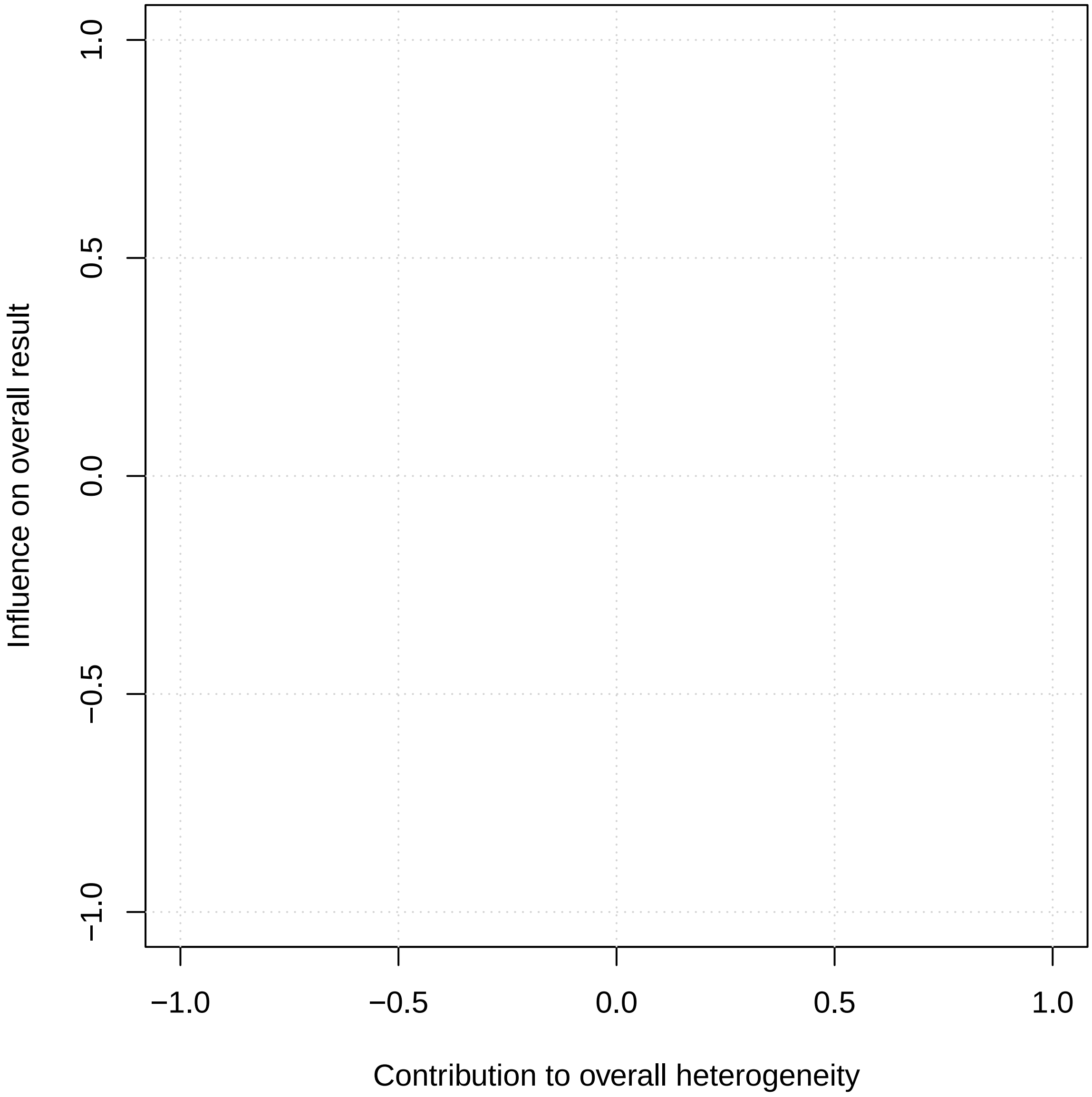

6.5.6 Leave one out

```r
metainf(vas_metacont)
```

```
## Influential analysis (common effect model)
##
##                                    MD             95%-CI p-value tau^2 tau I^2
## Omitting Sur 2022            -0.3400 [-0.6488; -0.0312]  0.0309
## Omitting Tang 2023           -0.3400 [-0.6488; -0.0312]  0.0309
## Omitting Du 2019             -0.3400 [-0.6488; -0.0312]  0.0309
## Omitting Lechevallier 2003   -0.3400 [-0.6488; -0.0312]  0.0309
## Omitting Zhang 2022
##
## Pooled estimate              -0.3400 [-0.6488; -0.0312]  0.0309
##
## Details on meta-analytical method:
## - Inverse variance method
## - Restricted maximum-likelihood estimator for tau^2
```

6.6 LoS

6.6.1 Meta-analysis

```
los_metacont <- metacont(data = rct_only,
                             mean.c = los_days_mean_control,
                             sd.c = los_days_sd_control,
                             mean.e = los_days_mean_suction,
                             sd.e = los_days_sd_suction,
                             n.e = sample_size_suction,
                             n.c = sample_size_control,
                       studlab = author_year)
los_metacont
```

```
## Number of studies: k = 2
## Number of observations: o = 397
##
##                              MD             95%-CI     z p-value
## Common effect model  -0.4332 [-0.8175; -0.0488] -2.21  0.0272
## Random effects model -0.4835 [-1.2668;  0.2998] -1.21  0.2264
##
## Quantifying heterogeneity:
##  tau^2 = 0.2409; tau = 0.4908; I^2 = 75.3% [0.0%; 94.4%]; H = 2.01 [1.00; 4.23]
##
## Test of heterogeneity:
##     Q d.f. p-value
##  4.04    1  0.0443
##
## Details on meta-analytical method:
## - Inverse variance method
## - Restricted maximum-likelihood estimator for tau^2
```

6.6.2 Forest plot

```r
forest(los_metacont,
       sortvar = TE)
```

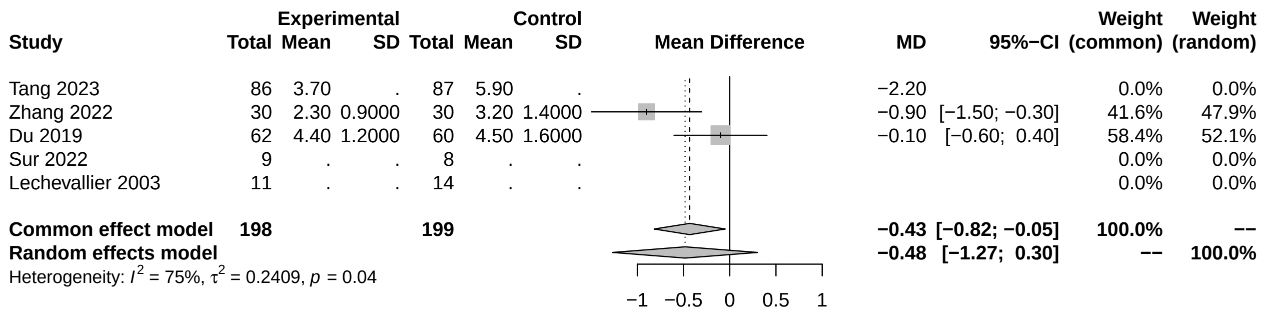

6.6.3 Trim and Fill

```
#trimfill(los_metacont)
#forest(trimfill(los_metacont),
#       sortvar = TE)
```

6.6.4 Funnel plot

```r
funnel((los_metacont))
```

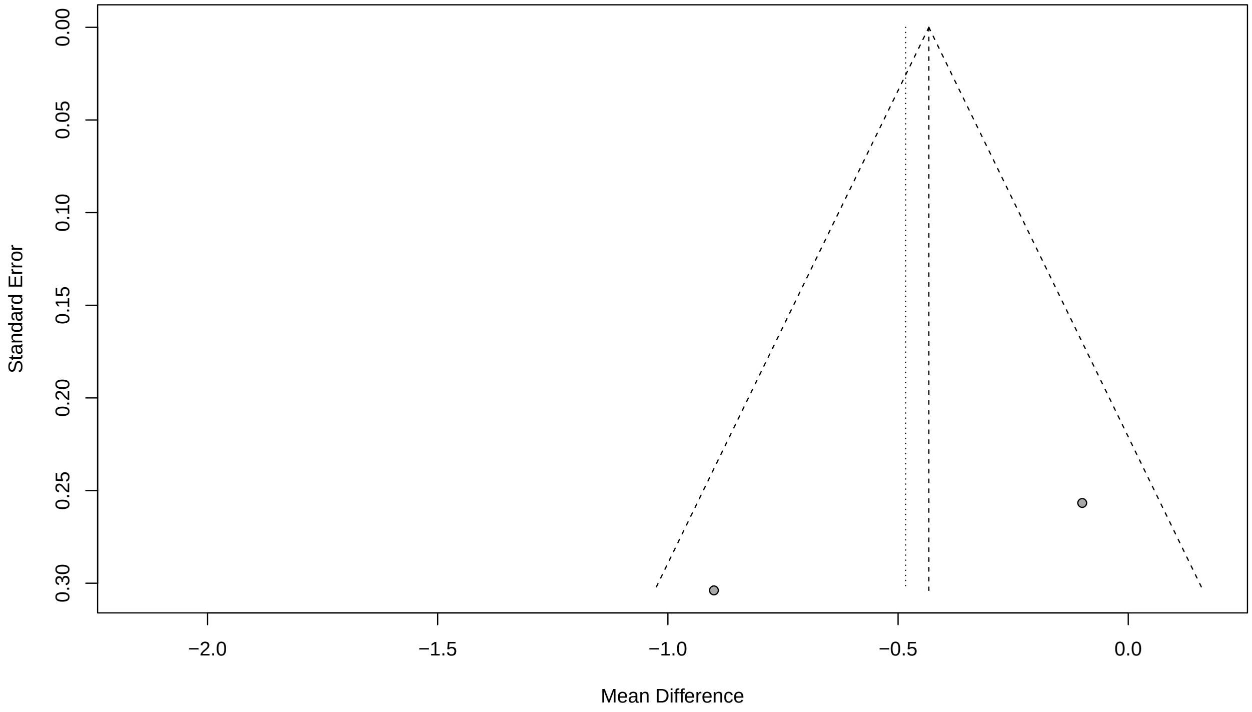

6.6.5 Baujat

```
baujat(los_metacont, pos = 1)
```

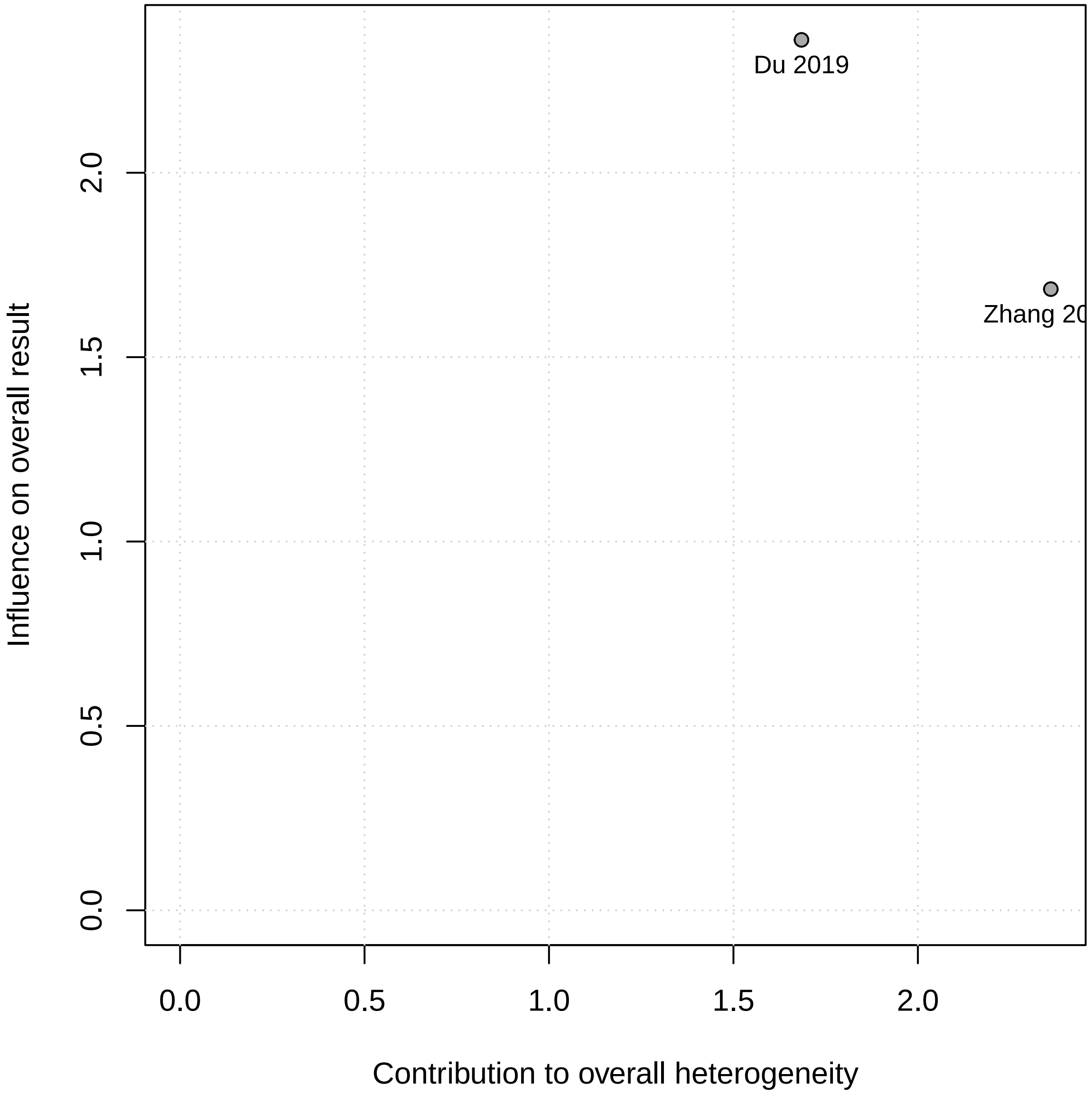

6.6.6 Leave one out

```r
metainf(los_metacont)
```

```
## Influential analysis (common effect model)
##
##                                   MD             95%-CI p-value   tau^2     tau
## Omitting Sur 2022            -0.4332 [-0.8175; -0.0488]  0.0272  0.2409  0.4908
## Omitting Tang 2023           -0.4332 [-0.8175; -0.0488]  0.0272  0.2409  0.4908
## Omitting Du 2019             -0.9000 [-1.4956; -0.3044]  0.0031
## Omitting Lechevallier 2003   -0.4332 [-0.8175; -0.0488]  0.0272  0.2409  0.4908
## Omitting Zhang 2022          -0.1000 [-0.6031;  0.4031]  0.6969
##
## Pooled estimate              -0.4332 [-0.8175; -0.0488]  0.0272  0.2409  0.4908
##                                 I^2
## Omitting Sur 2022             75.3%
## Omitting Tang 2023            75.3%
## Omitting Du 2019
## Omitting Lechevallier 2003    75.3%
## Omitting Zhang 2022
##
## Pooled estimate               75.3%
##
## Details on meta-analytical method:
## - Inverse variance method
## - Restricted maximum-likelihood estimator for tau^2
```

6.7 Complications - All

6.7.1 Meta-analysis

```
comp_metabin <- metabin(data = rct_only,
                          event.c = complications_postop_overall_n_control,
                        n.c = sample_size_control,
                        event.e = complications_postop_overall_n_suction,
                        n.e = sample_size_suction,
                        studlab = author_year)
comp_metabin
```

```
## Number of studies: k = 4
## Number of observations: o = 397
## Number of events: e = 51
##
##                          RR           95%-CI     z  p-value
## Common effect model  0.1847 [0.0895; 0.3815] -4.56 < 0.0001
## Random effects model 0.1978 [0.0944; 0.4143] -4.30 < 0.0001
##
## Quantifying heterogeneity:
##  tau^2 = 0 [0.0000; 11.1598]; tau = 0 [0.0000; 3.3406]
##  I^2 = 0.0% [0.0%; 84.7%]; H = 1.00 [1.00; 2.56]
##
## Test of heterogeneity:
##     Q d.f. p-value
##  2.05    3  0.5623
##
## Details on meta-analytical method:
## - Mantel-Haenszel method
## - Inverse variance method
## - Restricted maximum-likelihood estimator for tau^2
## - Q-Profile method for confidence interval of tau^2 and tau
```

6.7.2 Forest plot

```r
forest(comp_metabin,
       sortvar = TE)
```

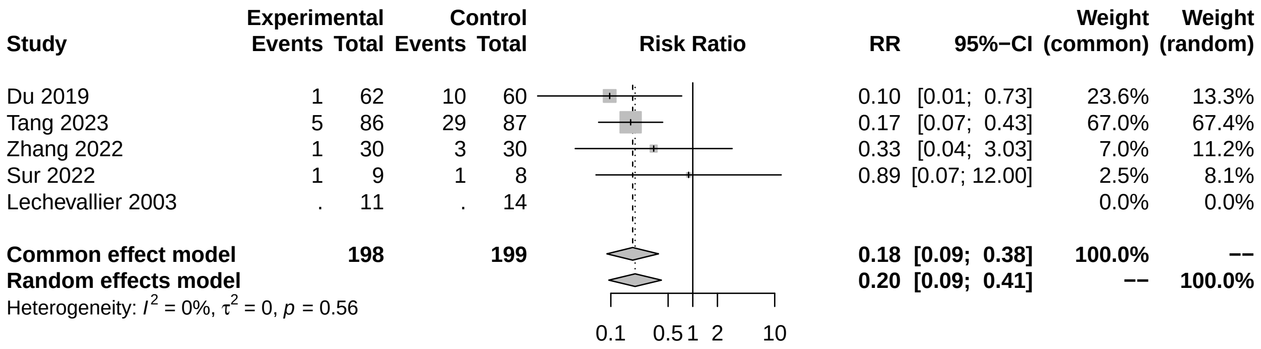

6.7.3 Trim and Fill

```r
trimfill(comp_metabin)
```

```
## Warning in trimfill.meta(comp_metabin): 1 observation(s) dropped due to missing
## values
```

```
## Number of studies: k = 6 (with 2 added studies)
## Number of observations: o = 449
## Number of events: e = 57
##
##                             RR           95%-CI     z  p-value
## Random effects model 0.1582 [0.0804; 0.3114] -5.34 < 0.0001
##
## Quantifying heterogeneity:
##  tau^2 < 0.0001 [0.0000; 7.1152]; tau = 0.0019 [0.0000; 2.6674]
##  I^2 = 0.0% [0.0%; 74.6%]; H = 1.00 [1.00; 1.99]
##
## Test of heterogeneity:
##     Q d.f. p-value
##  4.53    5  0.4765
##
## Details on meta-analytical method:
## - Inverse variance method
## - Restricted maximum-likelihood estimator for tau^2
## - Q-Profile method for confidence interval of tau^2 and tau
## - Trim-and-fill method to adjust for funnel plot asymmetry (L-estimator)
```

```r
forest(trimfill(comp_metabin),
       sortvar = TE)
```

```
## Warning in trimfill.meta(comp_metabin): 1 observation(s) dropped due to missing
## values
```

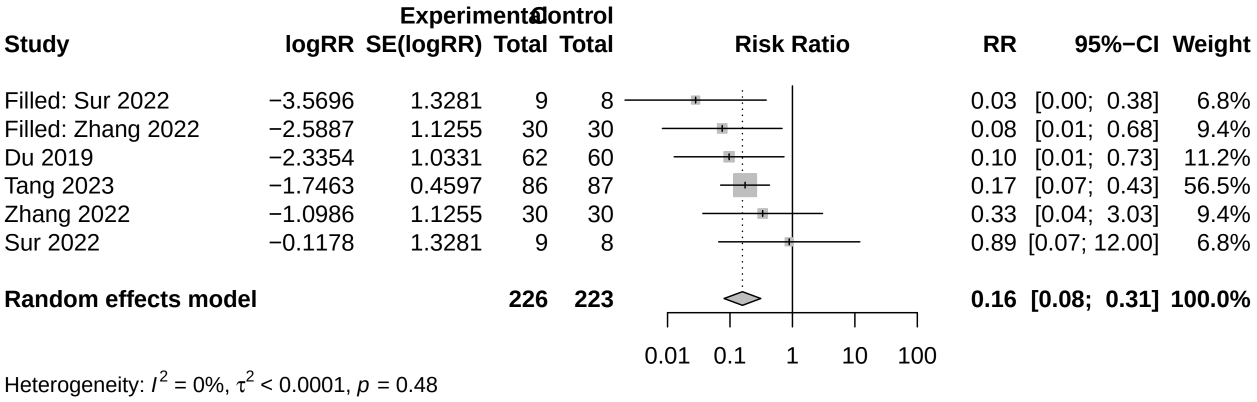

6.7.4 Funnel plot

```
funnel(trimfill(comp_metabin))
```

```
## Warning in trimfill.meta(comp_metabin): 1 observation(s) dropped due to missing
## values
```

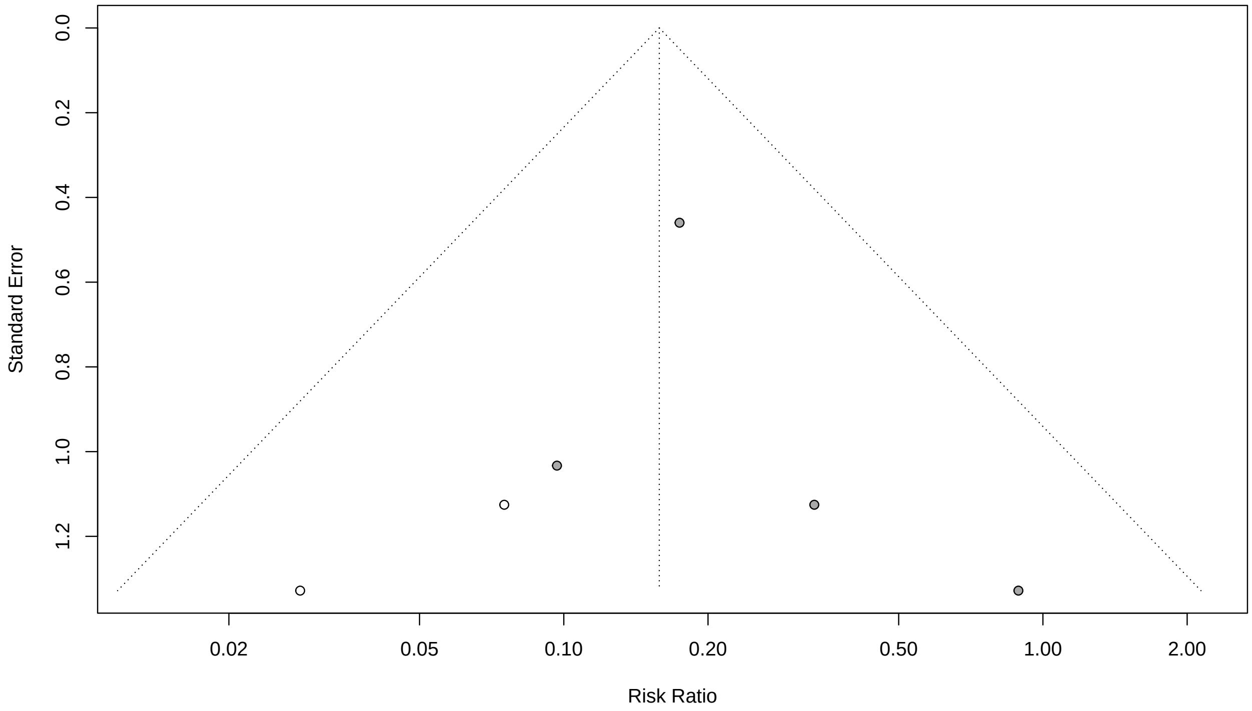

6.7.5 Baujat

```
baujat(comp_metabin, pos = 1)
```

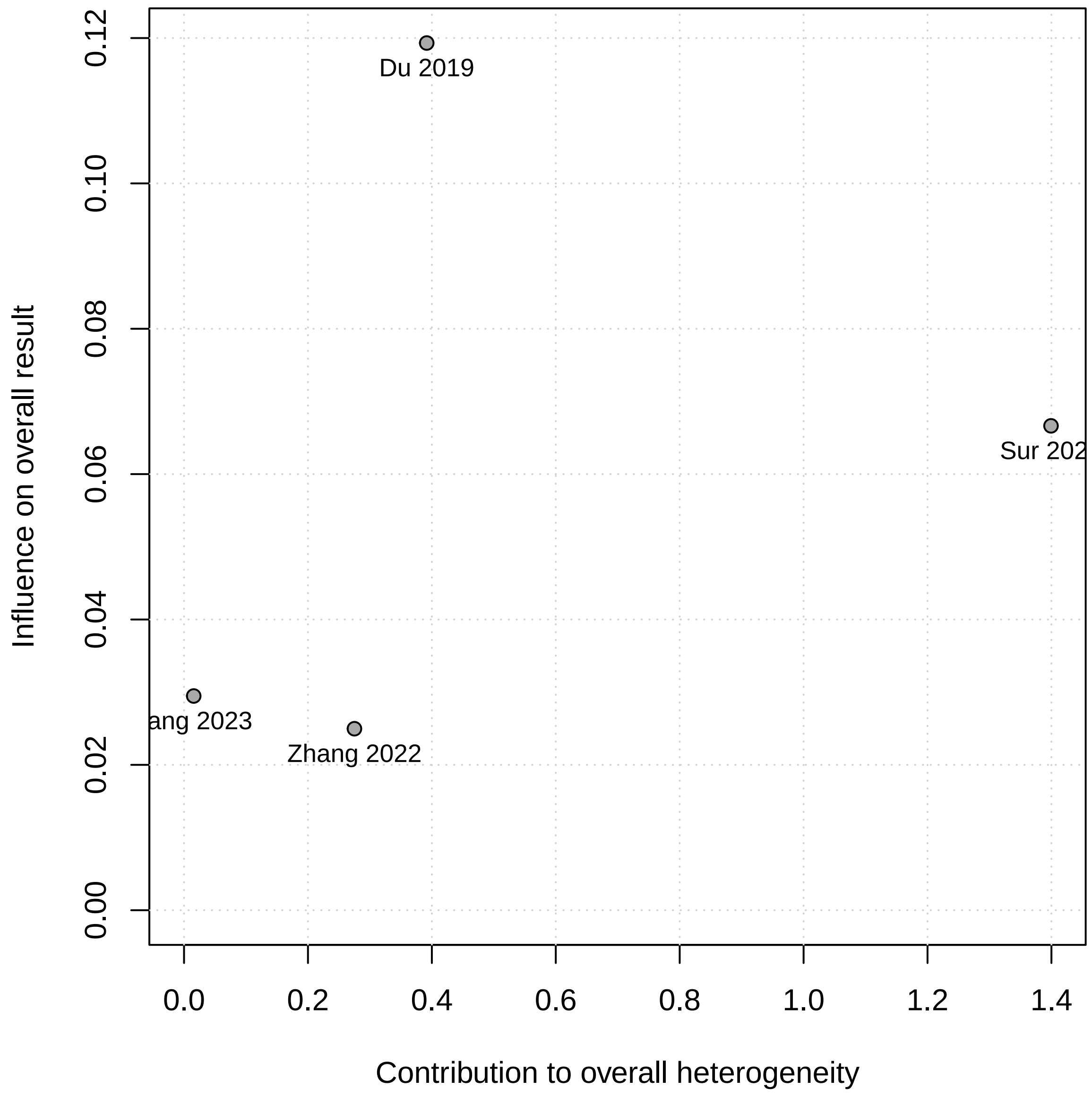

6.7.6 Leave one out

```r
metainf(comp_metabin)
```

```
## Influential analysis (common effect model)
##
##                                   RR           95%-CI  p-value   tau^2     tau
## Omitting Sur 2022             0.1670 [0.0775; 0.3596] < 0.0001  0.0000  0.0000
## Omitting Tang 2023            0.2056 [0.0605; 0.6994]   0.0113  0.0000  0.0000
## Omitting Du 2019              0.2119 [0.0973; 0.4618] < 0.0001  0.0000  0.0000
## Omitting Lechevallier 2003    0.1847 [0.0895; 0.3815] < 0.0001  0.0000  0.0000
## Omitting Zhang 2022           0.1736 [0.0803; 0.3752] < 0.0001  0.0000  0.0012
##
## Pooled estimate               0.1847 [0.0895; 0.3815] < 0.0001  0.0000  0.0000
##                                  I^2
## Omitting Sur 2022               0.0%
## Omitting Tang 2023              0.0%
## Omitting Du 2019                0.0%
## Omitting Lechevallier 2003      0.0%
## Omitting Zhang 2022             0.0%
##
## Pooled estimate                 0.0%
##
## Details on meta-analytical method:
## - Mantel-Haenszel method
## - Restricted maximum-likelihood estimator for tau^2
```

6.8 Fever

6.8.1 Meta-analysis

```
fever_metabin <- metabin(data = rct_only,
                         event.c = fever_n_control,
                       n.c = sample_size_control,
                       event.e = fever_n_suction,
                       n.e = sample_size_suction,
                       studlab = author_year)
fever_metabin
```

```
## Number of studies: k = 3
## Number of observations: o = 397
## Number of events: e = 23
##
##                           RR           95%-CI     z p-value
## Common effect model  0.2096 [0.0728; 0.6031] -2.90  0.0038
## Random effects model 0.2165 [0.0747; 0.6277] -2.82  0.0048
##
## Quantifying heterogeneity:
##  tau^2 = 0 [0.0000; 6.4956]; tau = 0 [0.0000; 2.5486]
##  I^2 = 0.0% [0.0%; 89.6%]; H = 1.00 [1.00; 3.10]
##
## Test of heterogeneity:
##     Q d.f. p-value
##  0.33    2  0.8476
##
## Details on meta-analytical method:
## - Mantel-Haenszel method
## - Inverse variance method
## - Restricted maximum-likelihood estimator for tau^2
## - Q-Profile method for confidence interval of tau^2 and tau
```

6.8.2 Forest plot

```r
forest(fever_metabin,
       sortvar = TE)
```

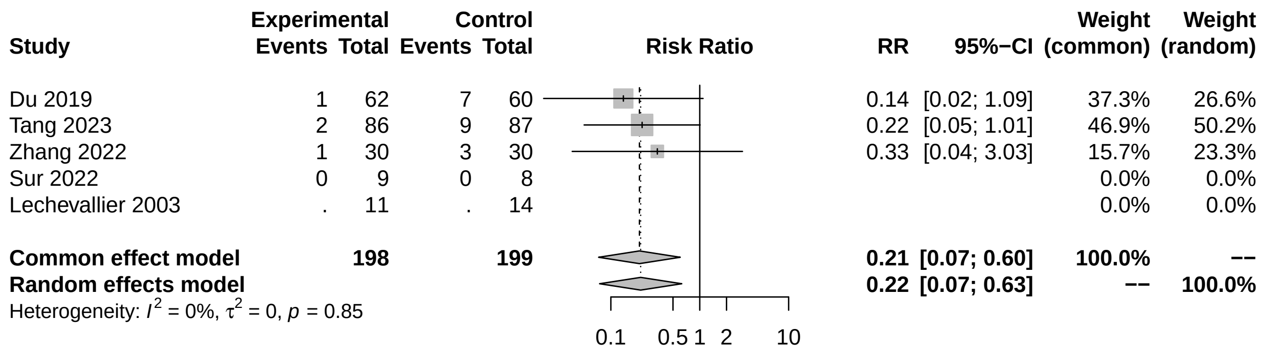

6.8.3 Trim and Fill

```r
trimfill(fever_metabin)
```

```
## Warning in trimfill.meta(fever_metabin): 2 observation(s) dropped due to
## missing values

## Number of studies: k = 3 (with 0 added studies)
## Number of observations: o = 355
## Number of events: e = 23
##
##                            RR           95%-CI     z p-value
## Random effects model 0.2165 [0.0747; 0.6277] -2.82  0.0048
##
## Quantifying heterogeneity:
##  tau^2 = 0 [0.0000; 6.4956]; tau = 0 [0.0000; 2.5486]
##  I^2 = 0.0% [0.0%; 89.6%]; H = 1.00 [1.00; 3.10]
##
## Test of heterogeneity:
##     Q d.f. p-value
##  0.33    2  0.8476
##
## Details on meta-analytical method:
## - Inverse variance method
## - Restricted maximum-likelihood estimator for tau^2
## - Q-Profile method for confidence interval of tau^2 and tau
## - Trim-and-fill method to adjust for funnel plot asymmetry (L-estimator)
```

```r
forest(trimfill(fever_metabin),
       sortvar = TE)
```

```
## Warning in trimfill.meta(fever_metabin): 2 observation(s) dropped due to
## missing values
```

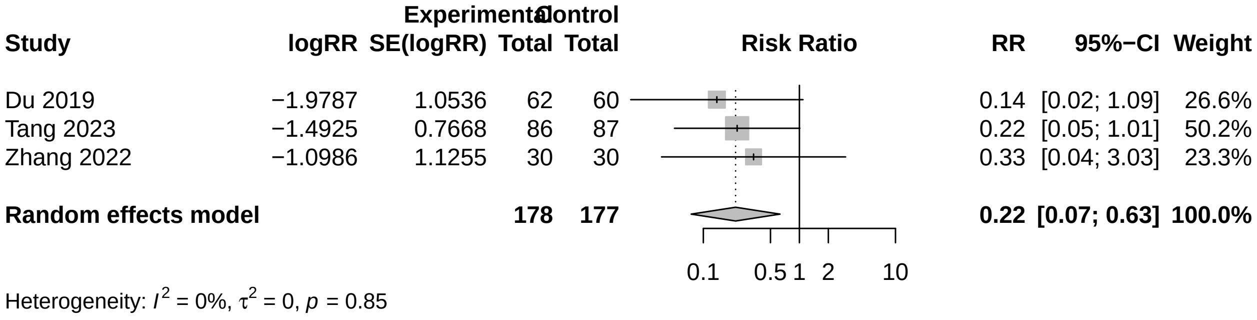

6.8.4 Funnel plot

```
funnel(trimfill(fever_metabin))
```

```
## Warning in trimfill.meta(fever_metabin): 2 observation(s) dropped due to
## missing values
```

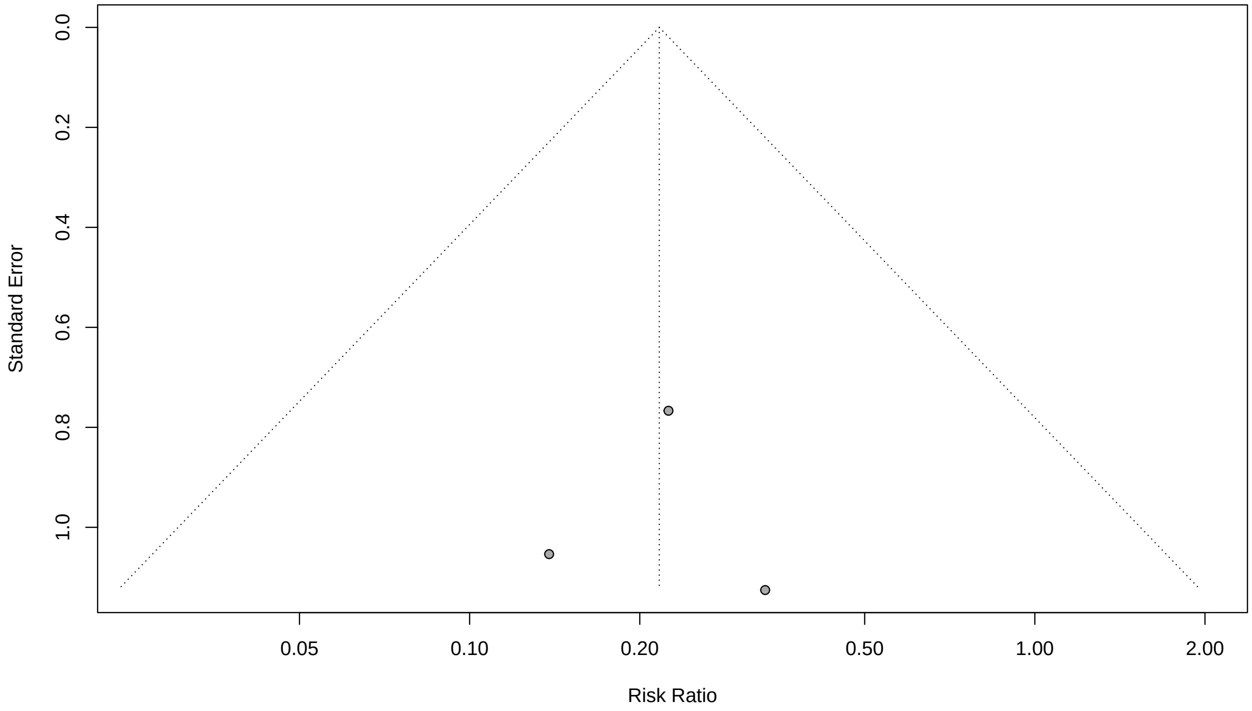

6.8.5 Baujat

```
baujat(fever_metabin, pos = 1)
```

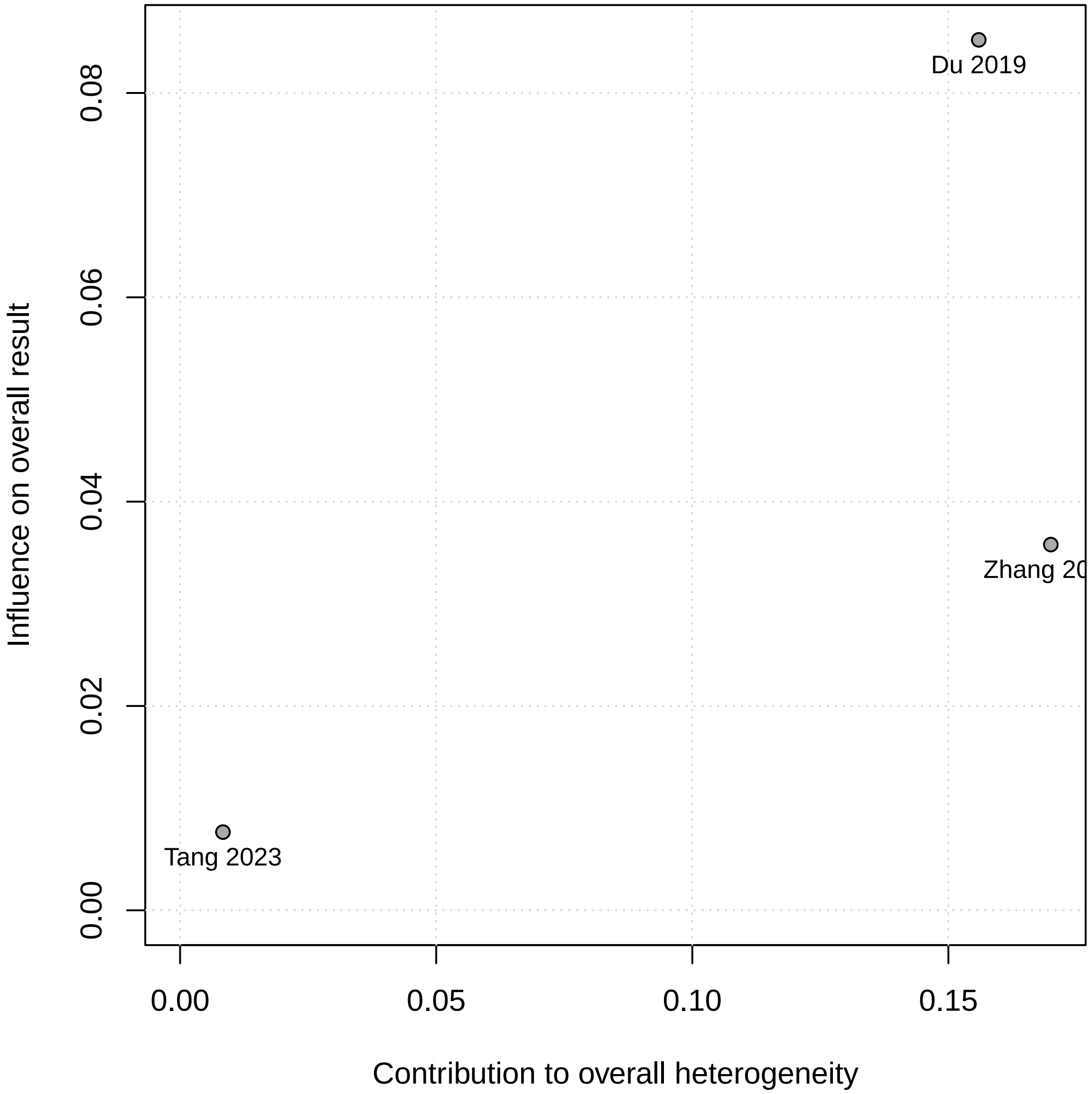

6.8.6 Leave one out

```r
metainf(fever_metabin)
```

```
## Influential analysis (common effect model)
##
##                                   RR            95%-CI p-value   tau^2     tau
## Omitting Sur 2022             0.2096 [0.0728; 0.6031]  0.0038  0.0000  0.0000
## Omitting Tang 2023            0.1961 [0.0443; 0.8686]  0.0319  0.0000  0.0000
## Omitting Du 2019              0.2521 [0.0730; 0.8703]  0.0293  0.0000  0.0000
## Omitting Lechevallier 2003    0.2096 [0.0728; 0.6031]  0.0038  0.0000  0.0000
## Omitting Zhang 2022           0.1865 [0.0556; 0.6256]  0.0065  0.0000  0.0000
##
## Pooled estimate               0.2096 [0.0728; 0.6031]  0.0038  0.0000  0.0000
##                                  I^2
## Omitting Sur 2022               0.0%
## Omitting Tang 2023              0.0%
## Omitting Du 2019                0.0%
## Omitting Lechevallier 2003      0.0%
## Omitting Zhang 2022             0.0%
##
## Pooled estimate                 0.0%
##
## Details on meta-analytical method:
## - Mantel-Haenszel method
## - Restricted maximum-likelihood estimator for tau^2
```

6.9 Infections

6.9.1 Meta-analysis

```
infection_metabin <- metabin(data = rct_only,
                         event.c = infection_n_control,
                       n.c = sample_size_control,
                       event.e = infection_n_suction,
                       n.e = sample_size_suction,
                       studlab = author_year)
infection_metabin
```

```
## Number of studies: k = 4
## Number of observations: o = 397
## Number of events: e = 24
##
##                          RR           95%-CI     z p-value
## Common effect model  0.2165 [0.0797; 0.5882] -3.00  0.0027
## Random effects model 0.2241 [0.0820; 0.6126] -2.92  0.0036
##
## Quantifying heterogeneity:
##  tau^2 = 0 [0.0000; 0.8702]; tau = 0 [0.0000; 0.9328]
##  I^2 = 0.0% [0.0%; 84.7%]; H = 1.00 [1.00; 2.56]
##
## Test of heterogeneity:
##     Q d.f. p-value
##  0.37    3  0.9468
##
## Details on meta-analytical method:
## - Mantel-Haenszel method
## - Inverse variance method
## - Restricted maximum-likelihood estimator for tau^2
## - Q-Profile method for confidence interval of tau^2 and tau
## - Continuity correction of 0.5 in studies with zero cell frequencies
```

6.9.2 Forest plot

```r
forest(infection_metabin,
       sortvar = TE)
```

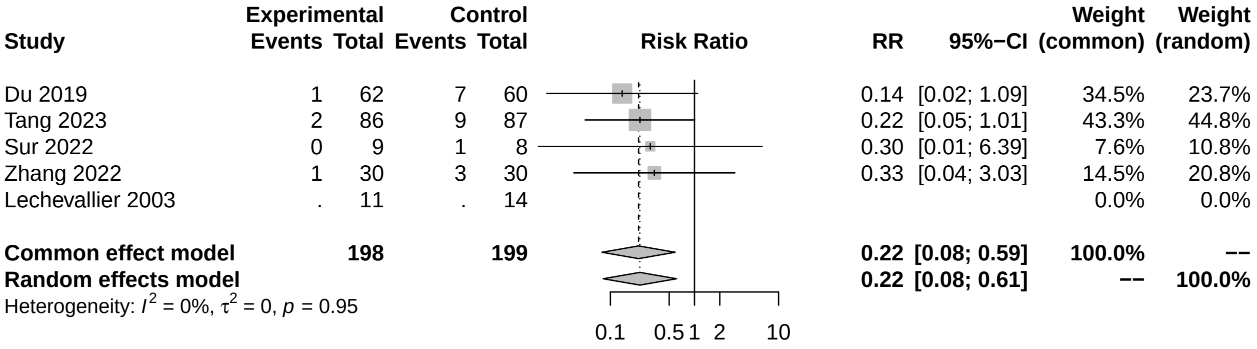

6.9.3 Trim and Fill

```r
trimfill(infection_metabin)
```

```
## Warning in trimfill.meta(infection_metabin): 1 observation(s) dropped due to
## missing values

## Number of studies: k = 6 (with 2 added studies)
## Number of observations: o = 449
## Number of events: e = 29
##
##                           RR           95%-CI     z p-value
## Random effects model 0.1900 [0.0791; 0.4565] -3.71  0.0002
##
## Quantifying heterogeneity:
##  tau^2 = 0; tau = 0; I^2 = 0.0% [0.0%; 74.6%]; H = 1.00 [1.00; 1.99]
##
## Test of heterogeneity:
##     Q d.f. p-value
##  0.80    5  0.9767
##
## Details on meta-analytical method:
## - Inverse variance method
## - Restricted maximum-likelihood estimator for tau^2
## - Trim-and-fill method to adjust for funnel plot asymmetry (L-estimator)
```

```r
forest(trimfill(infection_metabin),
       sortvar = TE)
```

```
## Warning in trimfill.meta(infection_metabin): 1 observation(s) dropped due to
## missing values
```

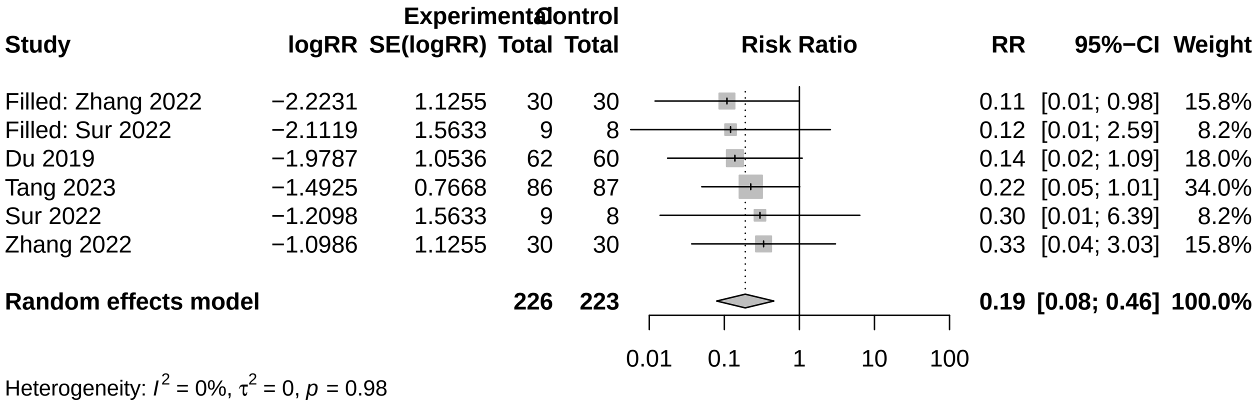

6.9.4 Funnel plot

```
funnel(trimfill(infection_metabin))
```

```
## Warning in trimfill.meta(infection_metabin): 1 observation(s) dropped due to
## missing values
```

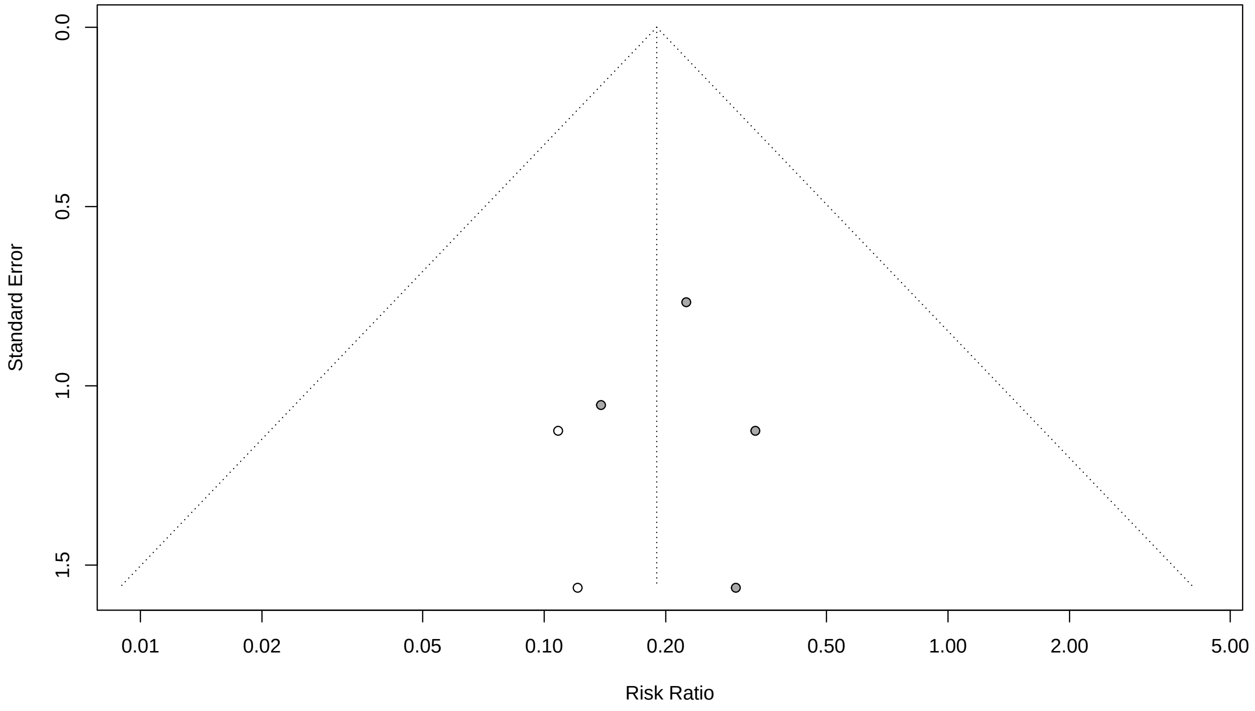

6.9.5 Baujat

```
baujat(infection_metabin, pos = 1)
```

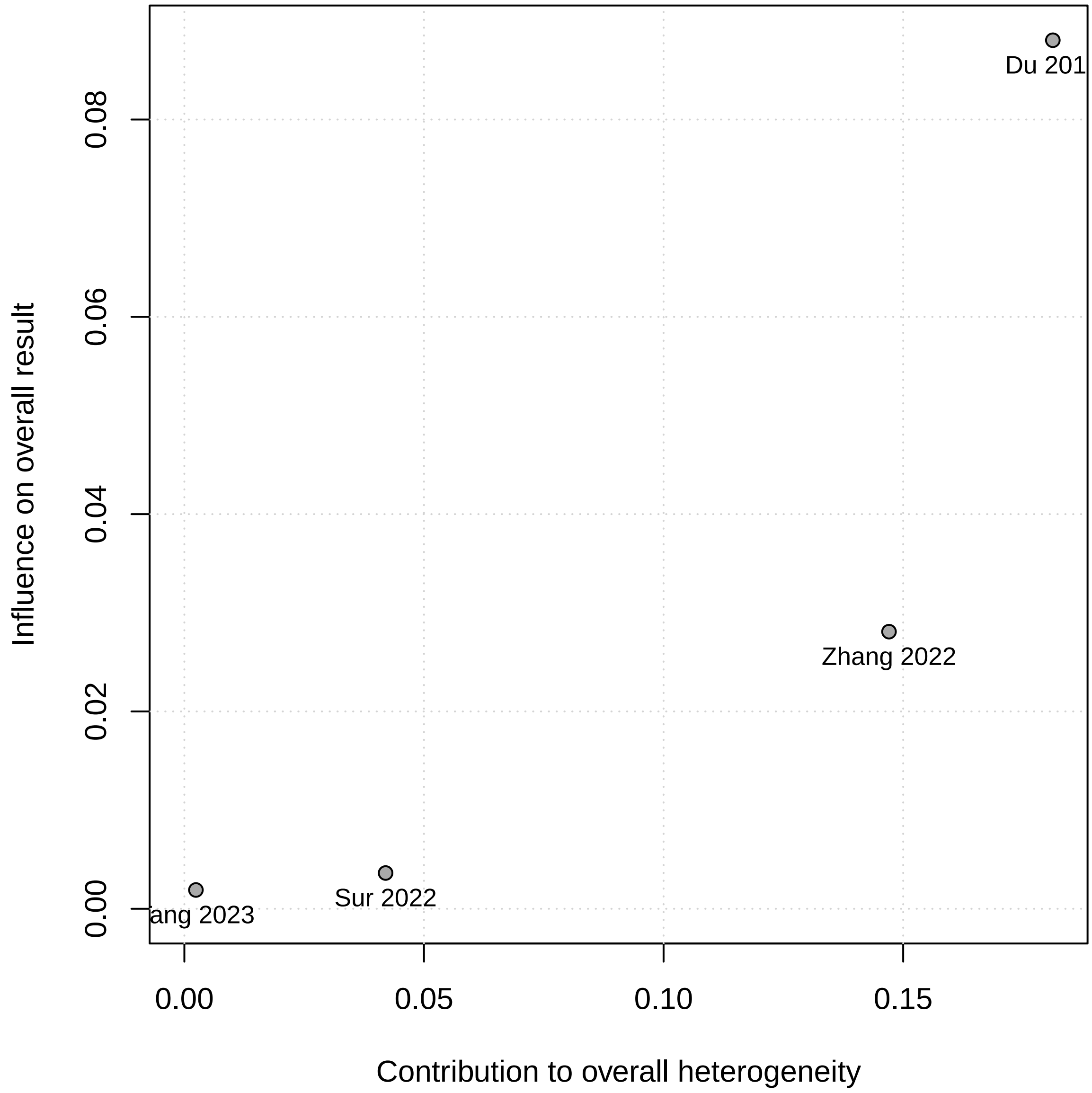

6.9.6 Leave one out

```
metainf(infection_metabin)
```

```
## Influential analysis (common effect model)
##
##                                    RR            95%-CI p-value   tau^2    tau
## Omitting Sur 2022              0.2096 [0.0728; 0.6031]  0.0038  0.0000  0.0000
## Omitting Tang 2023             0.2101 [0.0551; 0.8015]  0.0224  0.0000  0.0000
## Omitting Du 2019               0.2577 [0.0816; 0.8134]  0.0208  0.0000  0.0000
## Omitting Lechevallier 2003     0.2165 [0.0797; 0.5882]  0.0027  0.0000  0.0000
## Omitting Zhang 2022            0.1966 [0.0638; 0.6061]  0.0046  0.0000  0.0000
##
## Pooled estimate                0.2165 [0.0797; 0.5882]  0.0027  0.0000  0.0000
##                                 I^2
## Omitting Sur 2022               0.0%
## Omitting Tang 2023              0.0%
## Omitting Du 2019                0.0%
## Omitting Lechevallier 2003      0.0%
## Omitting Zhang 2022             0.0%
##
## Pooled estimate                 0.0%
##
## Details on meta-analytical method:
## - Mantel-Haenszel method
## - Restricted maximum-likelihood estimator for tau^2
```

6.10 Sepsis

6.10.1 Meta-analysis

```
sepsis_metabin <- metabin(data = rct_only,
                         event.c = sepsis_n_control,
                       n.c = sample_size_control,
                       event.e = sepsis_n_suction,
                       n.e = sample_size_suction,
                       studlab = author_year)
sepsis_metabin
```

```
## Number of studies: k = 1
## Number of observations: o = 397
## Number of events: e = 4
##
##                            RR           95%-CI     z p-value
## Common effect model  0.1124 [0.0061; 2.0562] -1.47  0.1405
## Random effects model 0.1124 [0.0061; 2.0562] -1.47  0.1405
##
## Quantifying heterogeneity:
##  tau^2 = NA; tau = NA; I^2 = NA; H = NA
##
## Details on meta-analytical method:
## - Mantel-Haenszel method
## - Inverse variance method
## - Restricted maximum-likelihood estimator for tau^2
## - Continuity correction of 0.5 in studies with zero cell frequencies
```

6.10.2 Forest plot

```r
forest(sepsis_metabin,
       sortvar = TE)
```

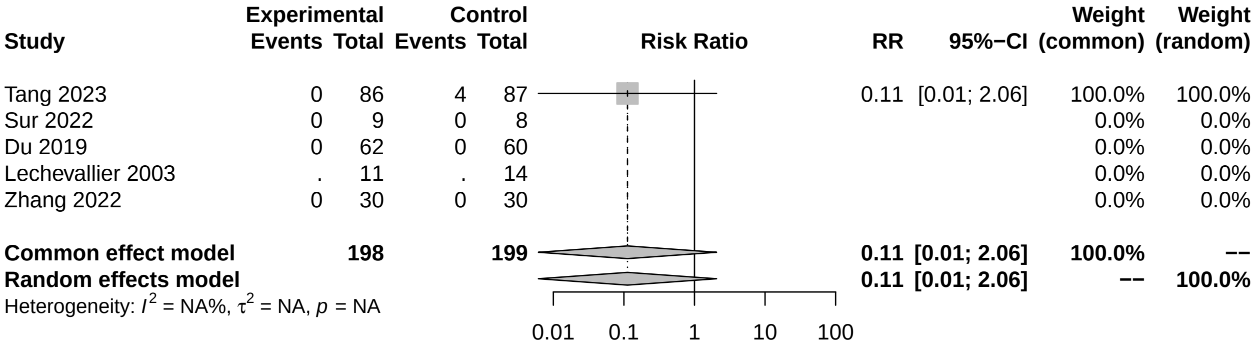

6.10.3 Trim and Fill

```
#trimfill(sepsis_metabin)
#forest(trimfill(sepsis_metabin),
#       sortvar = TE)
```

6.10.4 Funnel plot

```
funnel((sepsis_metabin))
```

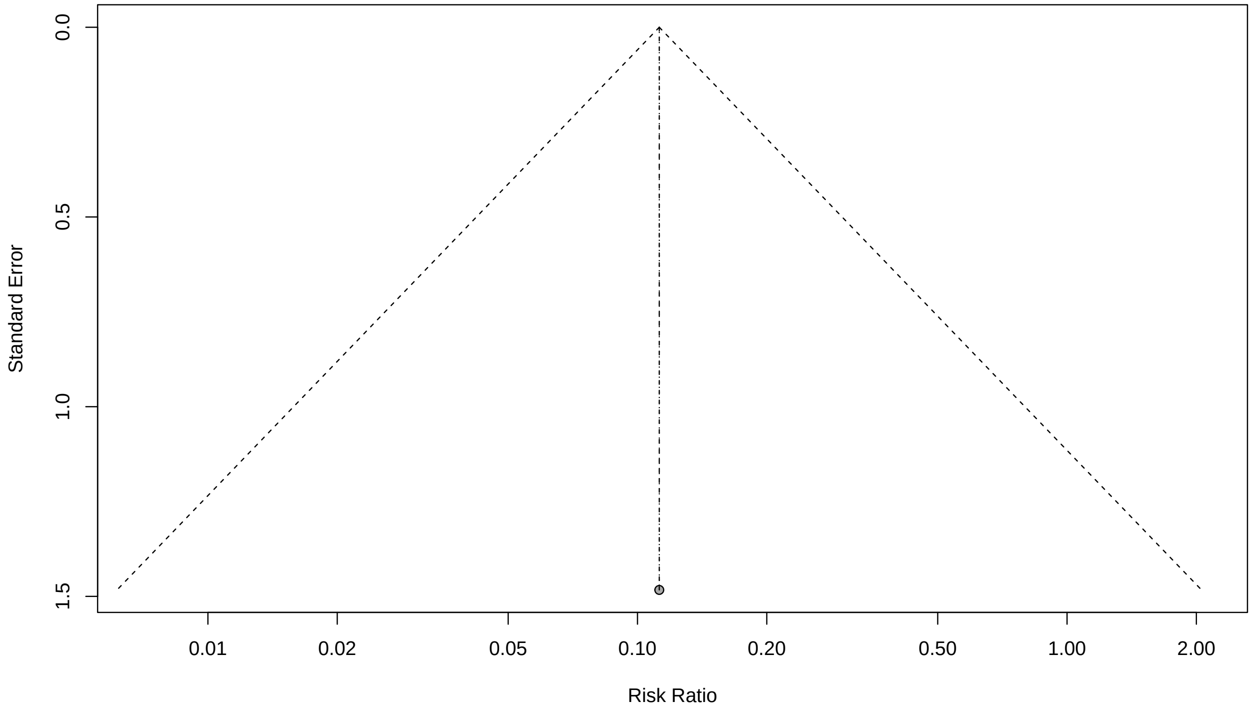

6.10.5 Baujat

```r
baujat(sepsis_metabin, pos = 1)
```

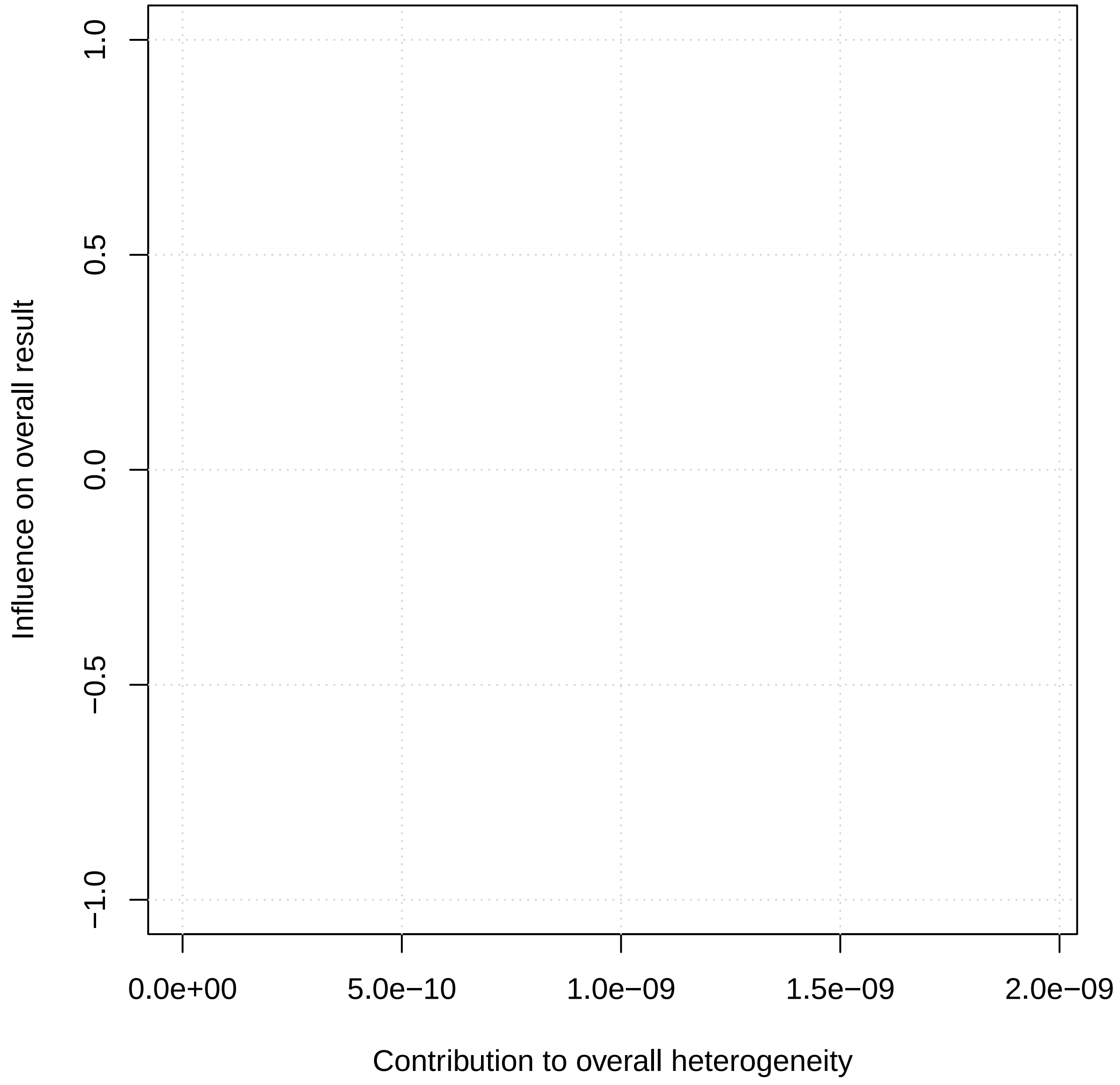

6.10.6 Leave one out

```r
metainf(sepsis_metabin)
```

```
## Influential analysis (common effect model)
##
##                                   RR           95%-CI p-value tau^2 tau I^2
## Omitting Sur 2022            0.1124 [0.0061; 2.0562]  0.1405
## Omitting Tang 2023
## Omitting Du 2019             0.1124 [0.0061; 2.0562]  0.1405
## Omitting Lechevallier 2003   0.1124 [0.0061; 2.0562]  0.1405
## Omitting Zhang 2022          0.1124 [0.0061; 2.0562]  0.1405
##
## Pooled estimate              0.1124 [0.0061; 2.0562]  0.1405
##
## Details on meta-analytical method:
## - Mantel-Haenszel method
## - Restricted maximum-likelihood estimator for tau^2
```

6.11 Abscess

6.11.1 Meta-analysis

```
abscess_metabin <- metabin(data = rct_only,
                         event.c = abscess_n_control,
                       n.c = sample_size_control,
                       event.e = abscess_n_suction,
                       n.e = sample_size_suction,
                       studlab = author_year)
abscess_metabin
```

```
## Number of studies: k = 0
## Number of observations: o = 397
## Number of events: e = 0
##
##                        RR 95%-CI  z p-value
## Common effect model  NA         --      --
## Random effects model NA         --      --
##
## Quantifying heterogeneity:
##  tau^2 = NA; tau = NA; I^2 = NA; H = NA
##
## Details on meta-analytical method:
## - Mantel-Haenszel method
## - Inverse variance method
## - Restricted maximum-likelihood estimator for tau^2
```

6.11.2 Forest plot

```r
forest(abscess_metabin,
       sortvar = TE)
```

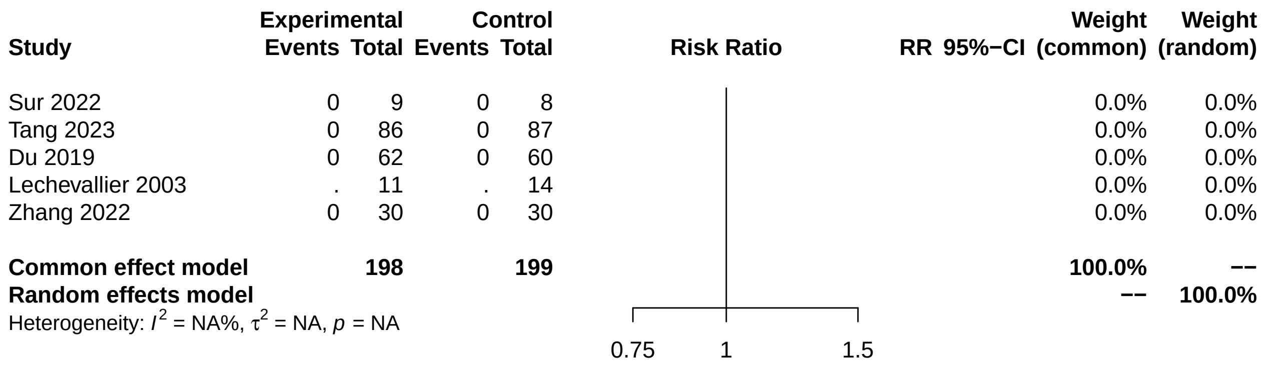

6.11.3 Trim and Fill

```
#trimfill(abscess_metabin)
#forest(trimfill(abscess_metabin),
#       sortvar = TE)
```

6.11.4 Funnel plot

```
#funnel((abscess_metabin))
```

6.11.5 Baujat

```
#baujat(abscess_metabin, pos = 1)
```

6.11.6 Leave one out

```
#metainf(abscess_metabin)
```

6.12 Haematoma

6.12.1 Meta-analysis

```
haematoma_metabin <- metabin(data = rct_only,
                           event.c = hematoma_n_control,
                         n.c = sample_size_control,
                         event.e = hematoma_n_suction,
                         n.e = sample_size_suction,
                         studlab = author_year)
haematoma_metabin
```

```
## Number of studies: k = 0
## Number of observations: o = 397
## Number of events: e = 0
##
##                       RR 95%-CI  z p-value
## Common effect model  NA        --      --
## Random effects model NA        --      --
##
## Quantifying heterogeneity:
##  tau^2 = NA; tau = NA; I^2 = NA; H = NA
##
## Details on meta-analytical method:
## - Mantel-Haenszel method
## - Inverse variance method
## - Restricted maximum-likelihood estimator for tau^2
```

6.12.2 Forest plot

```
forest(haematoma_metabin,
       sortvar = TE)
```

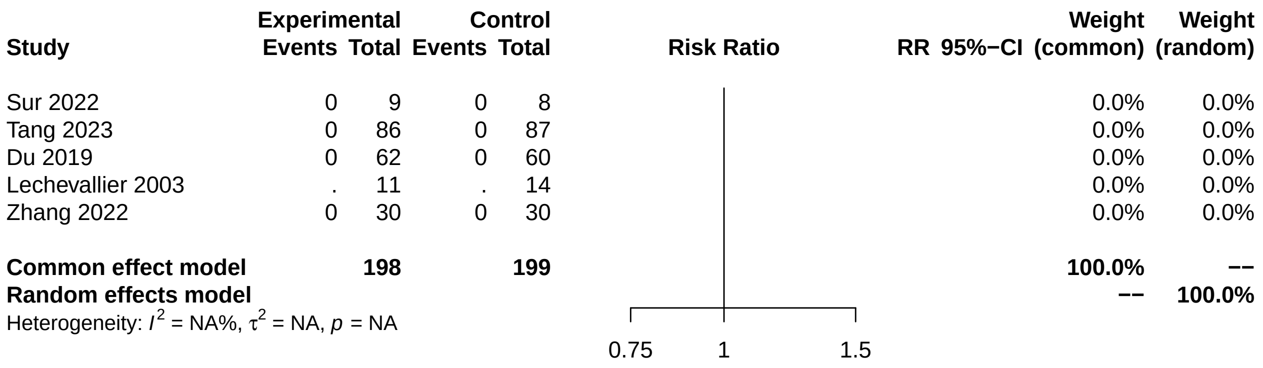

6.12.3 Trim and Fill

```
#trimfill(haematoma_metabin)
#forest(trimfill(haematoma_metabin),
#       sortvar = TE)
```

6.12.4 Funnel plot

```
#funnel((haematoma_metabin))
```

6.12.5 Baujat

```
#baujat(haematoma_metabin, pos = 1)
```

6.12.6 Leave one out

```
#metainf(haematoma_metabin)
```

6.13 Pain

6.13.1 Meta-analysis

```
pain_metabin <- metabin(data = rct_only,
                         event.c = pain_n_control,
                       n.c = sample_size_control,
                       event.e = pain_n_suction,
                       n.e = sample_size_suction,
                       studlab = author_year)
pain_metabin
```

```
## Number of studies: k = 1
## Number of observations: o = 397
## Number of events: e = 15
##
##                          RR           95%-CI     z p-value
## Common effect model  0.1556 [0.0362; 0.6692] -2.50  0.0124
## Random effects model 0.1556 [0.0362; 0.6692] -2.50  0.0124
##
## Quantifying heterogeneity:
##  tau^2 = NA; tau = NA; I^2 = NA; H = NA
##
## Details on meta-analytical method:
## - Mantel-Haenszel method
## - Inverse variance method
## - Restricted maximum-likelihood estimator for tau^2
```

6.13.2 Forest plot

```r
forest(pain_metabin,
       sortvar = TE)
```

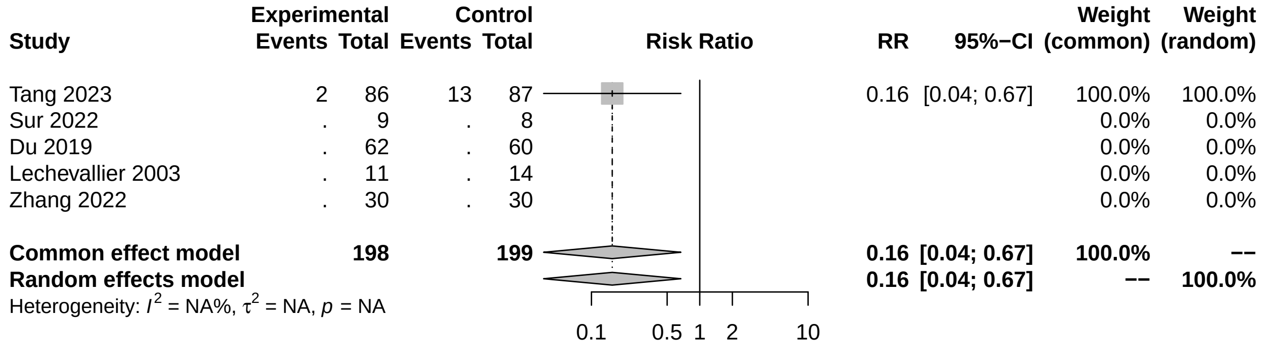

6.13.3 Trim and Fill

```
#trimfill(pain_metabin)
#forest(trimfill(pain_metabin),
#       sortvar = TE)
```

6.13.4 Funnel plot

```
funnel((pain_metabin))
```

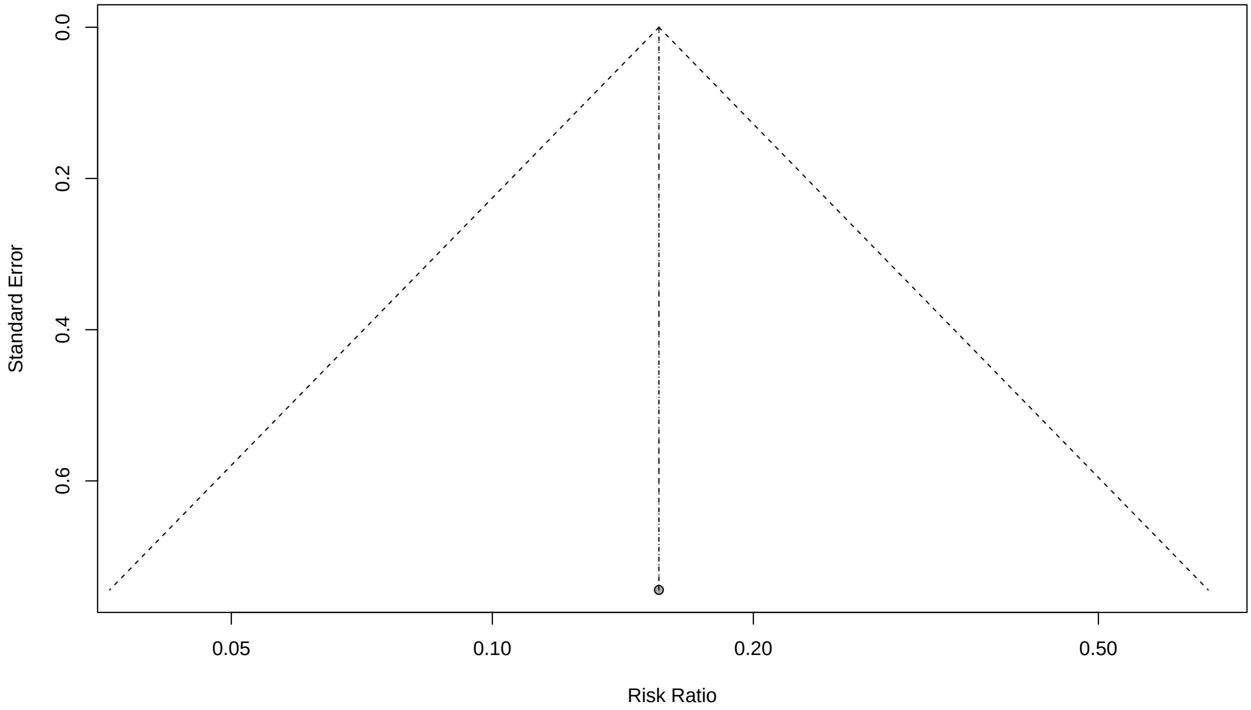

6.13.5 Baujat

```r
baujat(pain_metabin, pos = 1)
```

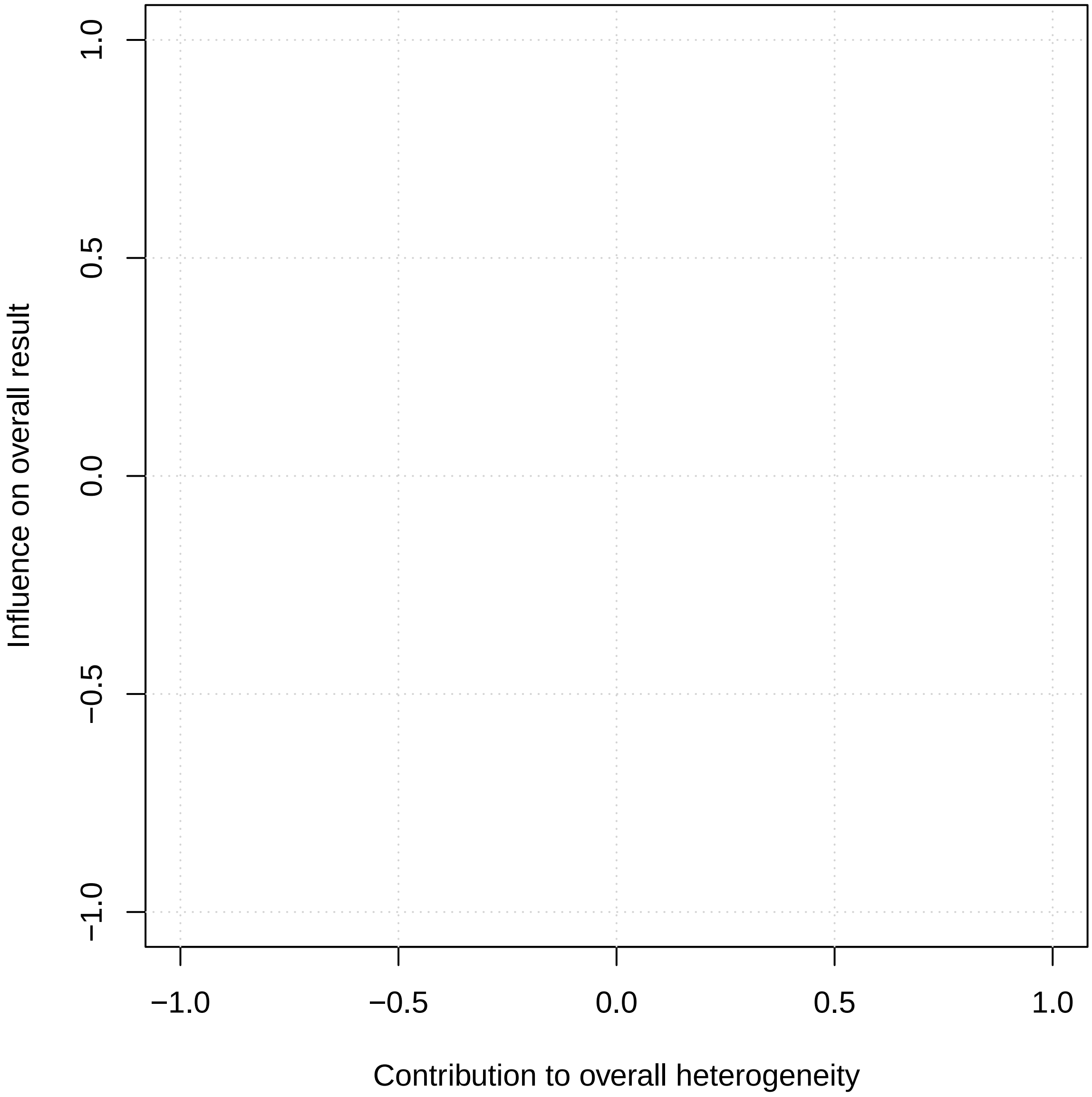

6.13.6 Leave one out

```
metainf(pain_metabin, pos = 1)
```

```
## Influential analysis (common effect model)
##
##                                    RR           95%-CI p-value tau^2 tau I^2
## Omitting Sur 2022            0.1556 [0.0362; 0.6692]  0.0124
## Omitting Tang 2023
## Omitting Du 2019             0.1556 [0.0362; 0.6692]  0.0124
## Omitting Lechevallier 2003   0.1556 [0.0362; 0.6692]  0.0124
## Omitting Zhang 2022          0.1556 [0.0362; 0.6692]  0.0124
##
## Pooled estimate              0.1556 [0.0362; 0.6692]  0.0124
##
## Details on meta-analytical method:
## - Mantel-Haenszel method
## - Restricted maximum-likelihood estimator for tau^2
```

6.14 Stricture

6.14.1 Meta-analysis

```
stricture_metabin <- metabin(data = rct_only,
                         event.c = stricture_n_control,
                       n.c = sample_size_control,
                       event.e = stricture_n_suction,
                       n.e = sample_size_suction,
                       studlab = author_year)
stricture_metabin
```

```
## Number of studies: k = 1
## Number of observations: o = 397
## Number of events: e = 3
##
##                          RR           95%-CI     z p-value
## Common effect model  0.1383 [0.0073; 2.6222] -1.32  0.1876
## Random effects model 0.1383 [0.0073; 2.6211] -1.32  0.1875
##
## Quantifying heterogeneity:
##  tau^2 = NA; tau = NA; I^2 = NA; H = NA
##
## Details on meta-analytical method:
## - Mantel-Haenszel method
## - Inverse variance method
## - Restricted maximum-likelihood estimator for tau^2
## - Continuity correction of 0.5 in studies with zero cell frequencies
```

6.14.2 Forest plot

```r
forest(stricture_metabin,
       sortvar = TE)
```

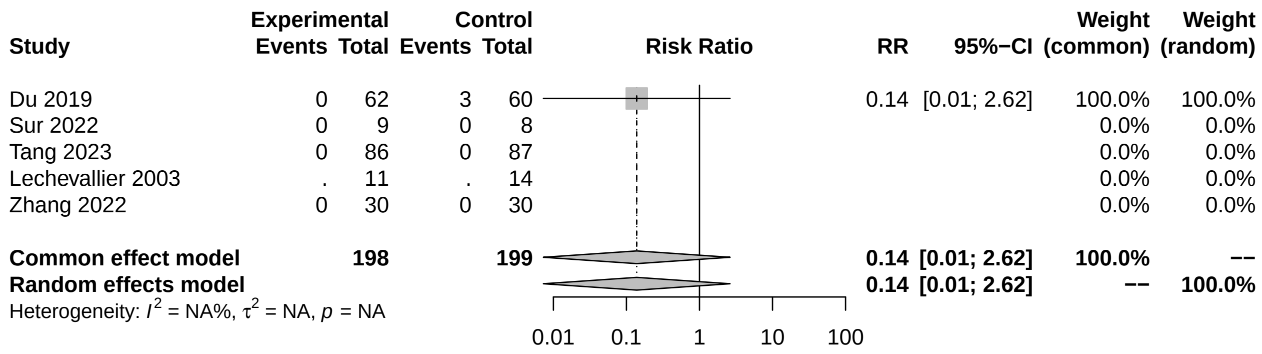

6.14.3 Trim and Fill

```
#trimfill(stricture_metabin)
#forest(trimfill(stricture_metabin),
#       sortvar = TE)
```

6.14.4 Funnel plot

```
funnel((stricture_metabin))
```

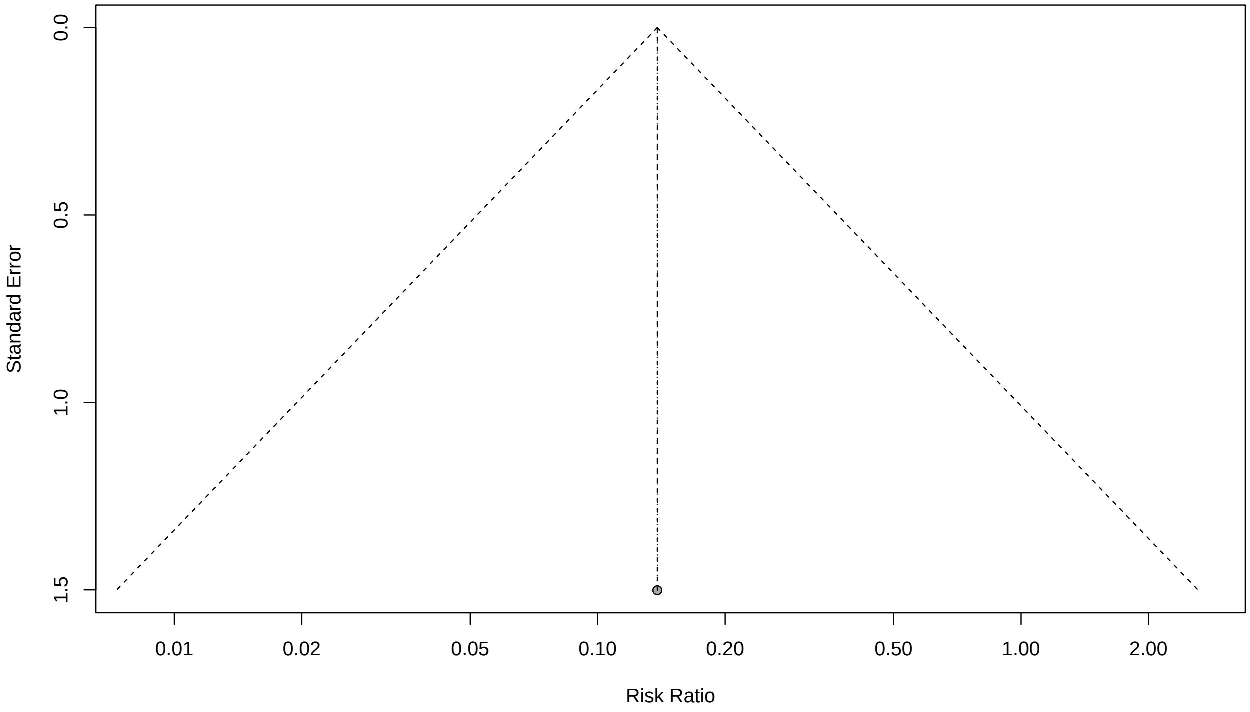

6.14.5 Baujat

```
baujat(stricture_metabin, pos = 1)
```

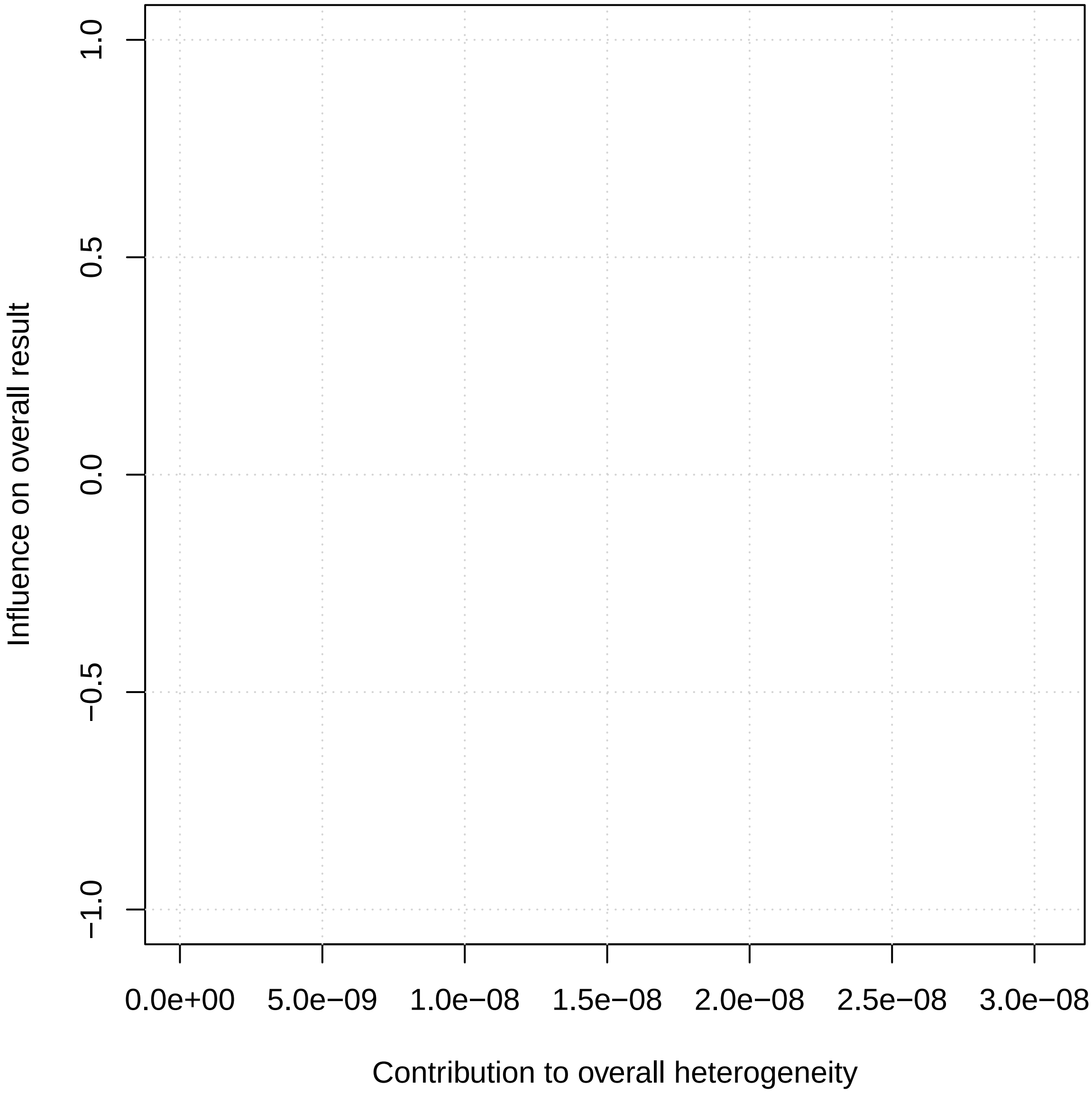

6.14.6 Leave one out

```
metainf(stricture_metabin)
```

```
## Influential analysis (common effect model)
##
##                                  RR           95%-CI p-value tau^2 tau I^2
## Omitting Sur 2022            0.1383 [0.0073; 2.6222]  0.1876
## Omitting Tang 2023           0.1383 [0.0073; 2.6222]  0.1876
## Omitting Du 2019
## Omitting Lechevallier 2003   0.1383 [0.0073; 2.6222]  0.1876
## Omitting Zhang 2022          0.1383 [0.0073; 2.6222]  0.1876
##
## Pooled estimate              0.1383 [0.0073; 2.6222]  0.1876
##
## Details on meta-analytical method:
## - Mantel-Haenszel method
## - Restricted maximum-likelihood estimator for tau^2
```

6.15 Embolisation required

6.15.1 Meta-analysis

```
ir_embolisation_metabin <- metabin(data = rct_only,
                           event.c = embolism_ir_intervention_n_control,
                         n.c = sample_size_control,
                         event.e = embolism_ir_intervention_n_suction,
                         n.e = sample_size_suction,
                         studlab = author_year)
ir_embolisation_metabin
```

```
## Number of studies: k = 0
## Number of observations: o = 397
## Number of events: e = 0
##
##                      RR 95%-CI  z p-value
## Common effect model  NA        --      --
## Random effects model NA        --      --
##
## Quantifying heterogeneity:
##  tau^2 = NA; tau = NA; I^2 = NA; H = NA
##
## Details on meta-analytical method:
## - Mantel-Haenszel method
## - Inverse variance method
## - Restricted maximum-likelihood estimator for tau^2
```

6.15.2 Forest plot

```r
forest(ir_embolisation_metabin,
       sortvar = TE)
```

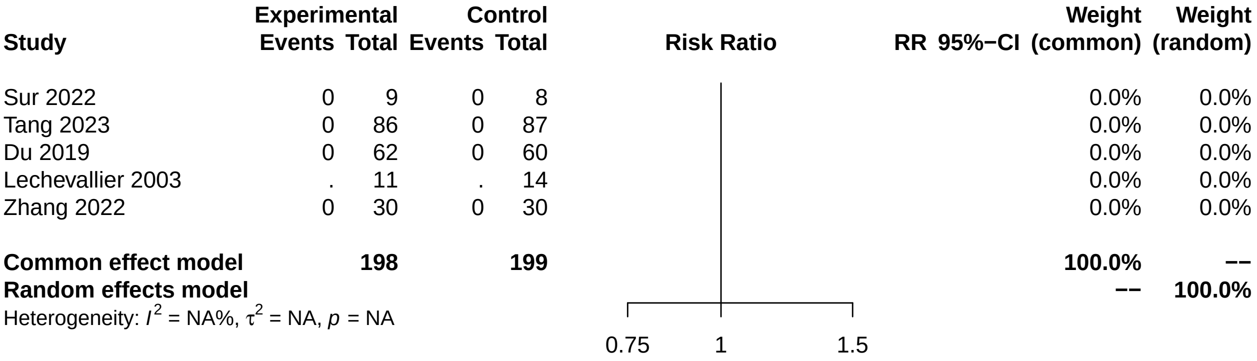

6.15.3 Trim and Fill

```
#trimfill(ir_embolisation_metabin)
#forest(trimfill(ir_embolisation_metabin),
#       sortvar = TE)
```

6.15.4 Funnel plot

```
#funnel((ir_embolisation_metabin))
```

6.15.5 Baujat

```
#baujat(ir_embolisation_metabin, pos = 1)
```

6.15.6 Leave one out

```
#metainf(ir_embolisation_metabin)
```

6.16 Transfusion

6.16.1 Meta-analysis

```
transfusion_metabin <- metabin(data = rct_only,
                          event.c = transfusion_n_control,
                        n.c = sample_size_control,
                        event.e = transfusion_n_suction,
                        n.e = sample_size_suction,
                        studlab = author_year)
transfusion_metabin
```

```
## Number of studies: k = 0
## Number of observations: o = 397
## Number of events: e = 0
##
##                        RR 95%-CI  z p-value
## Common effect model  NA        --      --
## Random effects model NA        --      --
##
## Quantifying heterogeneity:
##  tau^2 = NA; tau = NA; I^2 = NA; H = NA
##
## Details on meta-analytical method:
## - Mantel-Haenszel method
## - Inverse variance method
## - Restricted maximum-likelihood estimator for tau^2
```

6.16.2 Forest plot

```r
forest(transfusion_metabin,
       sortvar = TE)
```

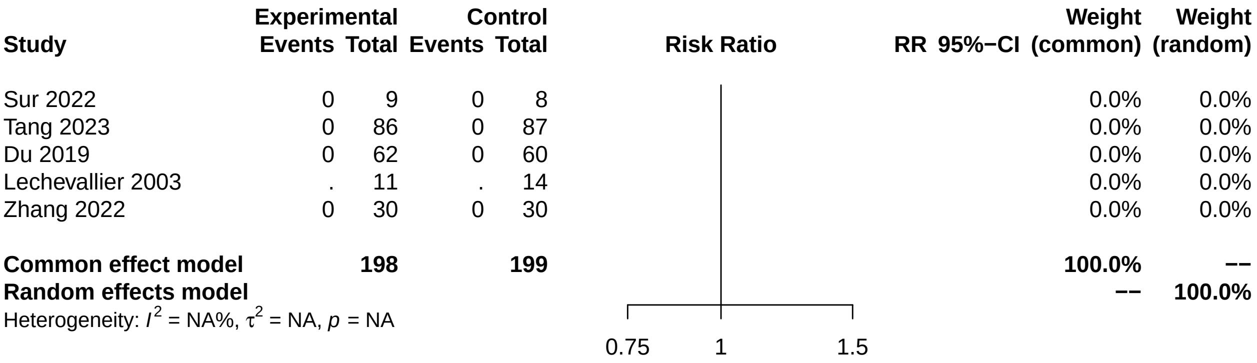

6.16.3 Trim and Fill

```r
#trimfill(transfusion_metabin)
#forest(trimfill(transfusion_metabin),
#       sortvar = TE)
```

6.16.4 Funnel plot

```
#funnel((transfusion_metabin))
```

6.16.5 Baujat

```
#baujat(transfusion_metabin, pos = 1)
```

6.16.6 Leave one out

```
#metainf(transfusion_metabin)
```

6.17 Clavien I

6.17.1 Meta-analysis

```
clav_i_metabin <- metabin(data = rct_only,
                           event.c = clavien_i_n_control,
                         n.c = sample_size_control,
                         event.e = clavien_i_n_suction,
                         n.e = sample_size_suction,
                         studlab = author_year)
clav_i_metabin
```

```
## Number of studies: k = 1
## Number of observations: o = 397
## Number of events: e = 1
##
##                           RR            95%-CI    z p-value
## Common effect model  2.7000 [0.1252; 58.2391] 0.63  0.5262
## Random effects model 2.6842 [0.1254; 57.4724] 0.63  0.5276
##
## Quantifying heterogeneity:
##  tau^2 = NA; tau = NA; I^2 = NA; H = NA
##
## Details on meta-analytical method:
## - Mantel-Haenszel method
## - Inverse variance method
## - Restricted maximum-likelihood estimator for tau^2
## - Continuity correction of 0.5 in studies with zero cell frequencies
```

6.17.2 Forest plot

```r
forest(clav_i_metabin,
       sortvar = TE)
```

Study	Experimental Events	Experimental Total	Control Events	Control Total	Risk Ratio	RR	95%-CI	Weight (common)	Weight (random)
Sur 2022	1	9	0	8		2.68	[0.13; 57.47]	100.0%	100.0%
Tang 2023	.	86	.	87				0.0%	0.0%
Du 2019	.	62	.	60				0.0%	0.0%
Lechevallier 2003	.	11	.	14				0.0%	0.0%
Zhang 2022	.	30	.	30				0.0%	0.0%
**Common effect model**		**198**		**199**		**2.70**	**[0.13; 58.24]**	**100.0%**	**--**
**Random effects model**						**2.68**	**[0.13; 57.47]**	**--**	**100.0%**

Heterogeneity: I^2 = NA%, τ^2 = NA, p = NA

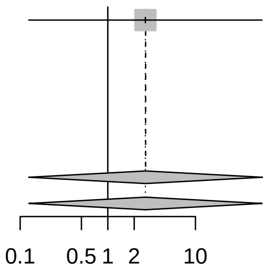

6.17.3 Trim and Fill

```
#trimfill(clav_i_metabin)
#forest(trimfill(clav_i_metabin),
#       sortvar = TE)
```

6.17.4 Funnel plot

```
funnel((clav_i_metabin))
```

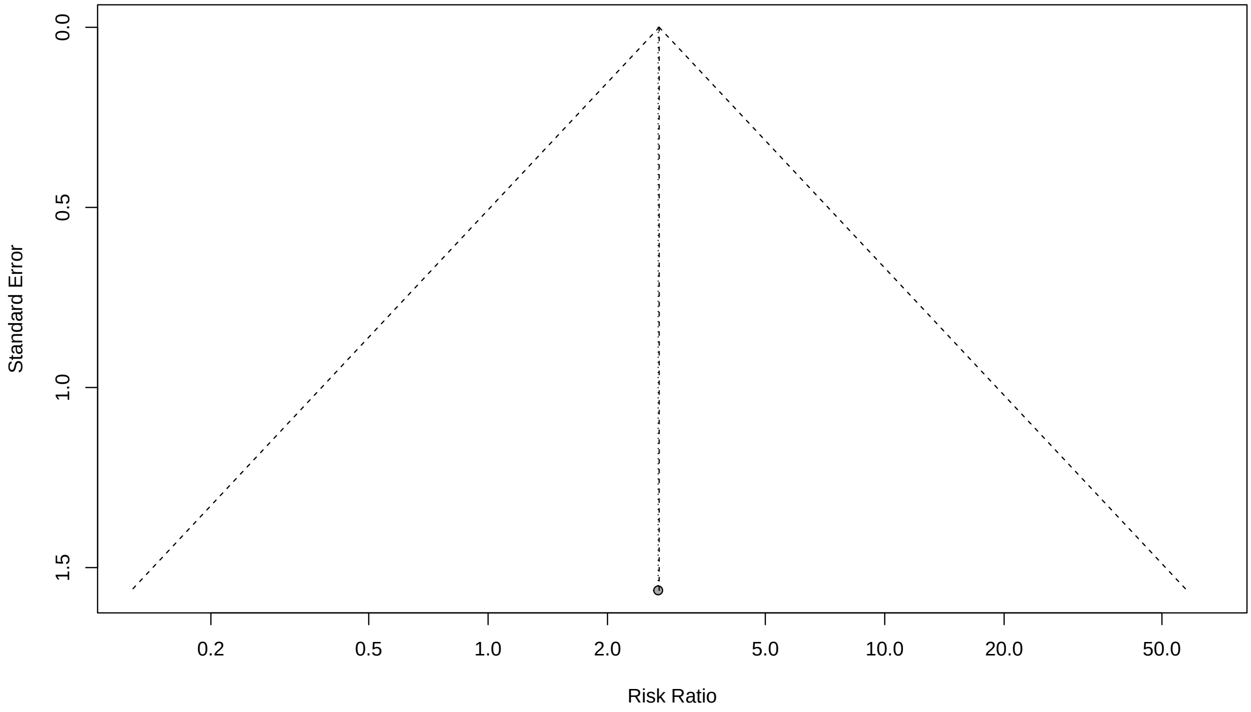

6.17.5 Baujat

```
baujat(clav_i_metabin, pos = 1)
```

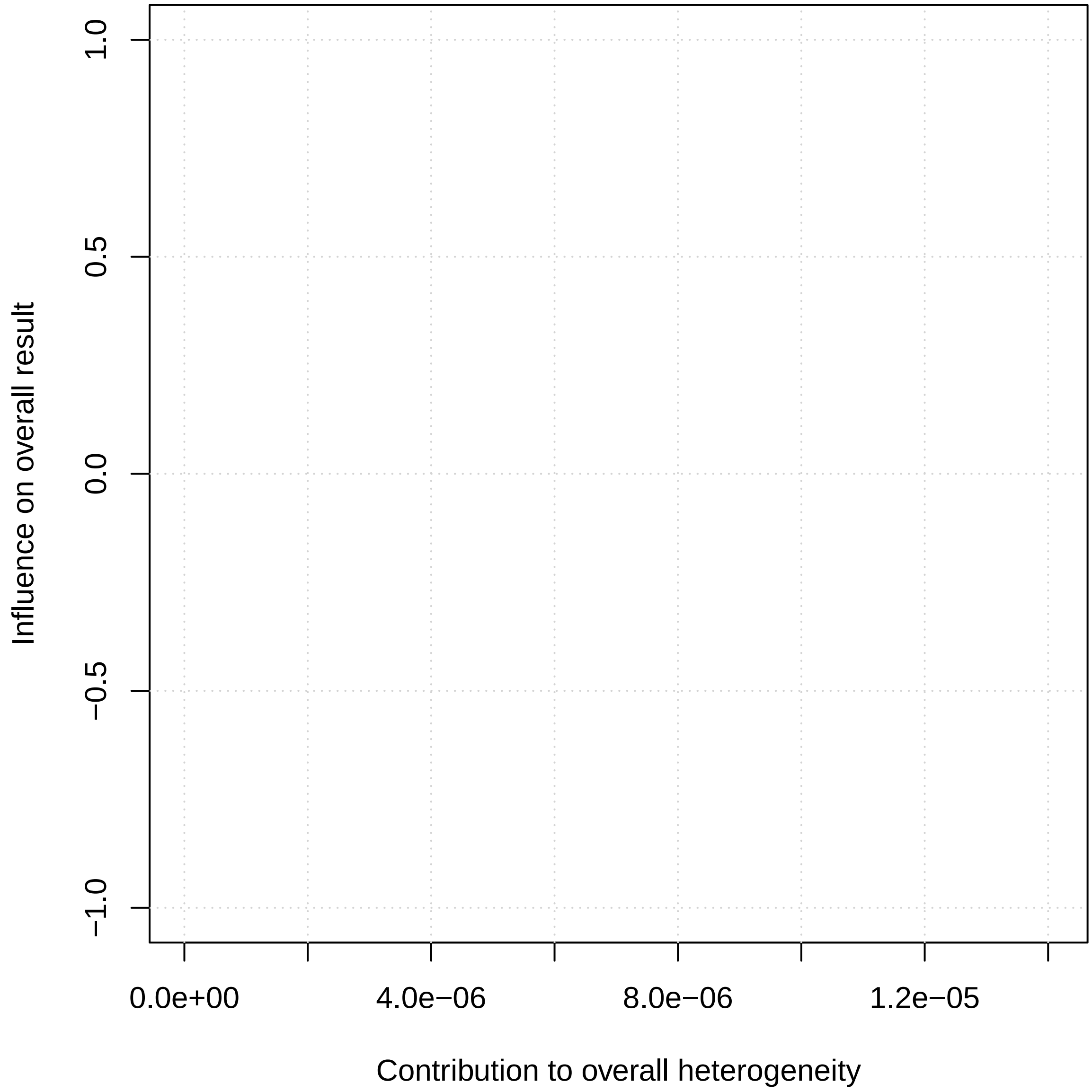

6.17.6 Leave one out

```
metainf(clav_i_metabin)
```

```
## Influential analysis (common effect model)
##
##                                 RR            95%-CI p-value tau^2 tau I^2
## Omitting Sur 2022
## Omitting Tang 2023           2.7000 [0.1252; 58.2391]  0.5262
## Omitting Du 2019             2.7000 [0.1252; 58.2391]  0.5262
## Omitting Lechevallier 2003   2.7000 [0.1252; 58.2391]  0.5262
## Omitting Zhang 2022          2.7000 [0.1252; 58.2391]  0.5262
##
## Pooled estimate              2.7000 [0.1252; 58.2391]  0.5262
##
## Details on meta-analytical method:
## - Mantel-Haenszel method
## - Restricted maximum-likelihood estimator for tau^2
```

6.18 Clavien II

6.18.1 Meta-analysis

```
clav_ii_metabin <- metabin(data = rct_only,
                         event.c = clavien_ii_n_control,
                       n.c = sample_size_control,
                       event.e = clavien_ii_n_suction,
                       n.e = sample_size_suction,
                       studlab = author_year)
clav_ii_metabin
```

```
## Number of studies: k = 1
## Number of observations: o = 397
## Number of events: e = 1
##
##                            RR           95%-CI     z p-value
## Common effect model  0.3000 [0.0139; 6.4710] -0.77  0.4423
## Random effects model 0.2982 [0.0139; 6.3858] -0.77  0.4390
##
## Quantifying heterogeneity:
##  tau^2 = NA; tau = NA; I^2 = NA; H = NA
##
## Details on meta-analytical method:
## - Mantel-Haenszel method
## - Inverse variance method
## - Restricted maximum-likelihood estimator for tau^2
## - Continuity correction of 0.5 in studies with zero cell frequencies
```

6.18.2 Forest plot

```r
forest(clav_ii_metabin,
       sortvar = TE)
```

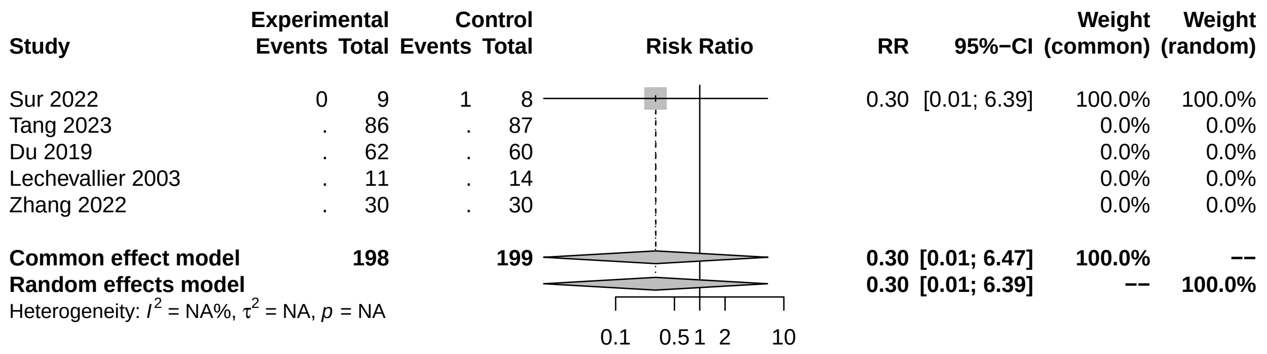

6.18.3 Trim and Fill

```
#trimfill(clav_ii_metabin)
#forest(trimfill(clav_ii_metabin),
#       sortvar = TE)
```

6.18.4 Funnel plot

```r
funnel((clav_ii_metabin))
```

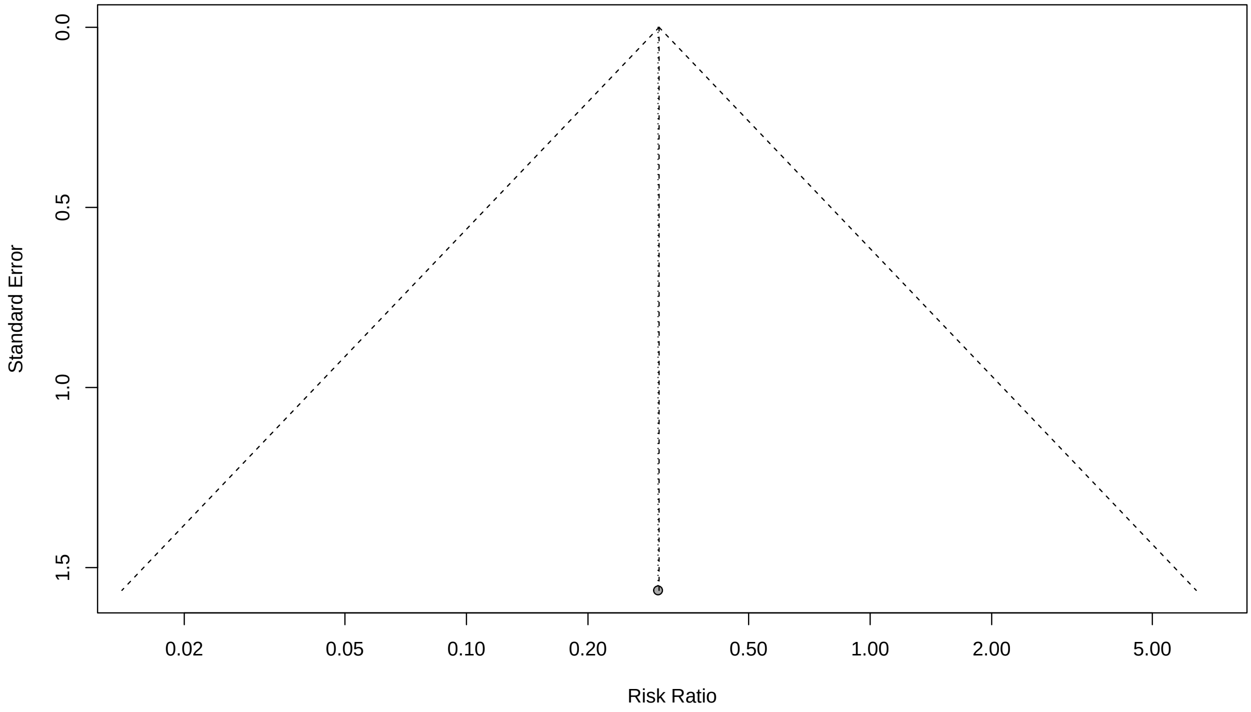

6.18.5 Baujat

```
baujat(clav_ii_metabin, pos = 1)
```

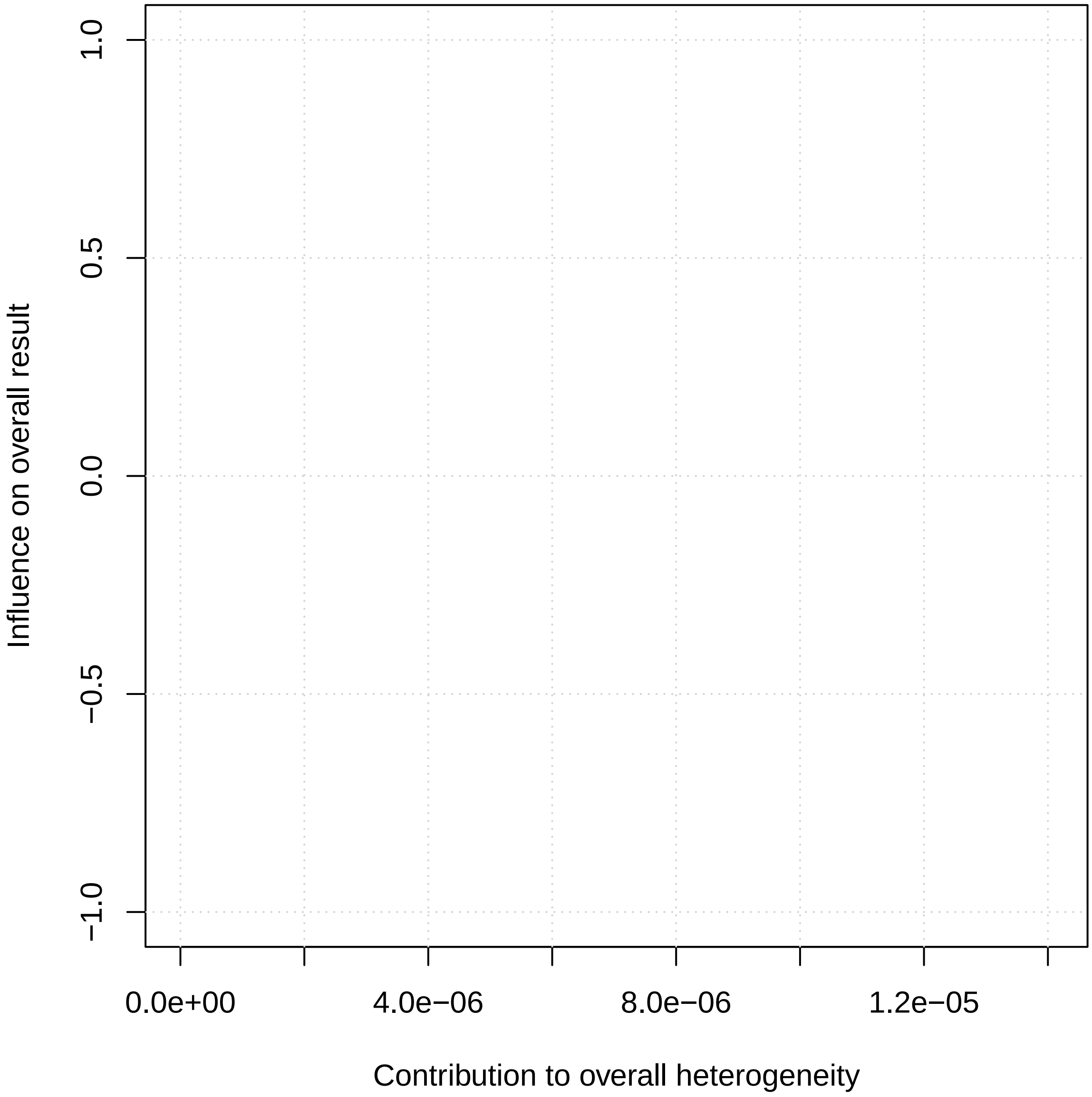

6.18.6 Leave one out

```
metainf(clav_ii_metabin)
```

```
## Influential analysis (common effect model)
##
##                                    RR           95%-CI p-value tau^2 tau I^2
## Omitting Sur 2022
## Omitting Tang 2023           0.3000 [0.0139; 6.4710]  0.4423
## Omitting Du 2019             0.3000 [0.0139; 6.4710]  0.4423
## Omitting Lechevallier 2003   0.3000 [0.0139; 6.4710]  0.4423
## Omitting Zhang 2022          0.3000 [0.0139; 6.4710]  0.4423
##
## Pooled estimate              0.3000 [0.0139; 6.4710]  0.4423
##
## Details on meta-analytical method:
## - Mantel-Haenszel method
## - Restricted maximum-likelihood estimator for tau^2
```

6.19 Clavien III

6.19.1 Meta-analysis

```
clav_iii_metabin <- metabin(data = rct_only,
                             event.c = clavien_iii_n_control,
                           n.c = sample_size_control,
                           event.e = clavien_iii_n_suction,
                           n.e = sample_size_suction,
                           studlab = author_year)
clav_iii_metabin
```

```
## Number of studies: k = 0
## Number of observations: o = 397
## Number of events: e = 0
##
##                        RR 95%-CI  z p-value
## Common effect model  NA         --      --
## Random effects model NA         --      --
##
## Quantifying heterogeneity:
##  tau^2 = NA; tau = NA; I^2 = NA; H = NA
##
## Details on meta-analytical method:
## - Mantel-Haenszel method
## - Inverse variance method
## - Restricted maximum-likelihood estimator for tau^2
```

6.19.2 Forest plot

```r
forest(clav_iii_metabin,
       sortvar = TE)
```

Study	Experimental Events	Experimental Total	Control Events	Control Total	Risk Ratio	RR	95%−CI	Weight (common)	Weight (random)
Sur 2022	0	9	0	8				0.0%	0.0%
Tang 2023	.	86	.	87				0.0%	0.0%
Du 2019	.	62	.	60				0.0%	0.0%
Lechevallier 2003	.	11	.	14				0.0%	0.0%
Zhang 2022	0	30	0	30				0.0%	0.0%
**Common effect model**		**198**		**199**				**100.0%**	**−−**
**Random effects model**								**−−**	**100.0%**

Heterogeneity: I^2 = NA%, τ^2 = NA, p = NA

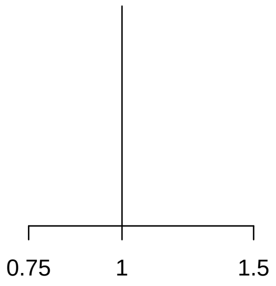

6.19.3 Trim and Fill

```
#trimfill(clav_iii_metabin)
#forest(trimfill(clav_iii_metabin),
#       sortvar = TE)
```

6.19.4 Funnel plot

```
#funnel((clav_iii_metabin))
```

6.19.5 Baujat

```
#baujat(clav_iii_metabin, pos = 1)
```

6.19.6 Leave one out

```
#metainf(clav_iii_metabin)
```

6.20 Clavien IV

6.20.1 Meta-analysis

```
clav_iv_metabin <- metabin(data = rct_only,
                         event.c = clavien_iv_n_control,
                       n.c = sample_size_control,
                       event.e = clavien_iv_n_suction,
                       n.e = sample_size_suction,
                       studlab = author_year)
clav_iv_metabin
```

```
## Number of studies: k = 0
## Number of observations: o = 397
## Number of events: e = 0
##
##                        RR 95%-CI  z p-value
## Common effect model  NA        --      --
## Random effects model NA        --      --
##
## Quantifying heterogeneity:
##  tau^2 = NA; tau = NA; I^2 = NA; H = NA
##
## Details on meta-analytical method:
## - Mantel-Haenszel method
## - Inverse variance method
## - Restricted maximum-likelihood estimator for tau^2
```

6.20.2 Forest plot

```r
forest(clav_iv_metabin,
       sortvar = TE)
```

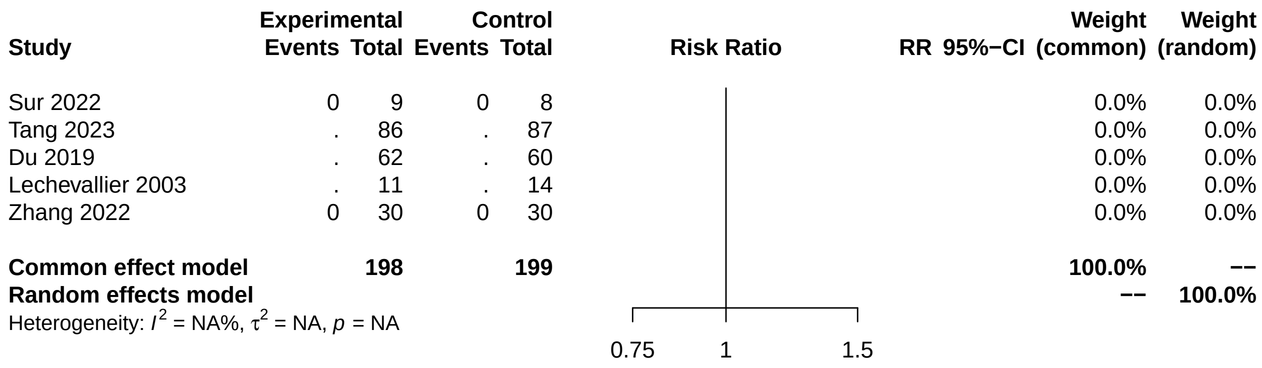

6.20.3 Trim and Fill

```
#trimfill(clav_iv_metabin)
#forest(trimfill(clav_iv_metabin),
#       sortvar = TE)
```

6.20.4 Funnel plot

```
#funnel((clav_iv_metabin))
```

6.20.5 Baujat

```
#baujat(clav_iv_metabin, pos = 1)
```

6.20.6 Leave one out

```
#metainf(clav_iv_metabin)
```

6.21 Clavien V

6.21.1 Meta-analysis

```
clav_v_metabin <- metabin(data = rct_only,
                           event.c = clavien_v_n_control,
                         n.c = sample_size_control,
                         event.e = clavien_v_n_suction,
                         n.e = sample_size_suction,
                         studlab = author_year)
clav_v_metabin
```

```
## Number of studies: k = 0
## Number of observations: o = 397
## Number of events: e = 0
##
##                       RR 95%-CI  z p-value
## Common effect model  NA        --      --
## Random effects model NA        --      --
##
## Quantifying heterogeneity:
##  tau^2 = NA; tau = NA; I^2 = NA; H = NA
##
## Details on meta-analytical method:
## - Mantel-Haenszel method
## - Inverse variance method
## - Restricted maximum-likelihood estimator for tau^2
```

6.21.2 Forest plot

```
#forest(clav_v_metabin,
#       sortvar = TE)
```

6.21.3 Trim and Fill

```
#trimfill(clav_v_metabin)
#forest(trimfill(clav_v_metabin),
#       sortvar = TE)
```

6.21.4 Funnel plot

```
#funnel(trimfill(clav_v_metabin))
```

6.21.5 Baujat

```
#baujat(clav_v_metabin, pos = 1)
```

6.21.6 Leave one out

```
#metainf(clav_v_metabin)
```

6.22 Clavien I-II

6.22.1 Meta-analysis

```
clav_i_ii_metabin <- metabin(data = rct_only,
                         event.c = clav_i_ii_n_control,
                       n.c = sample_size_control,
                       event.e = clav_i_ii_n_suction,
                       n.e = sample_size_suction,
                       studlab = author_year)
clav_i_ii_metabin
```

```
## Number of studies: k = 1
## Number of observations: o = 397
## Number of events: e = 2
##
##                         RR           95%-CI     z p-value
## Common effect model  0.8889 [0.0658; 12.0044] -0.09  0.9293
## Random effects model 0.8889 [0.0658; 12.0044] -0.09  0.9293
##
## Quantifying heterogeneity:
##  tau^2 = NA; tau = NA; I^2 = NA; H = NA
##
## Details on meta-analytical method:
## - Mantel-Haenszel method
## - Inverse variance method
## - Restricted maximum-likelihood estimator for tau^2
```

6.22.2 Forest plot

```r
forest(clav_i_ii_metabin,
       sortvar = TE)
```

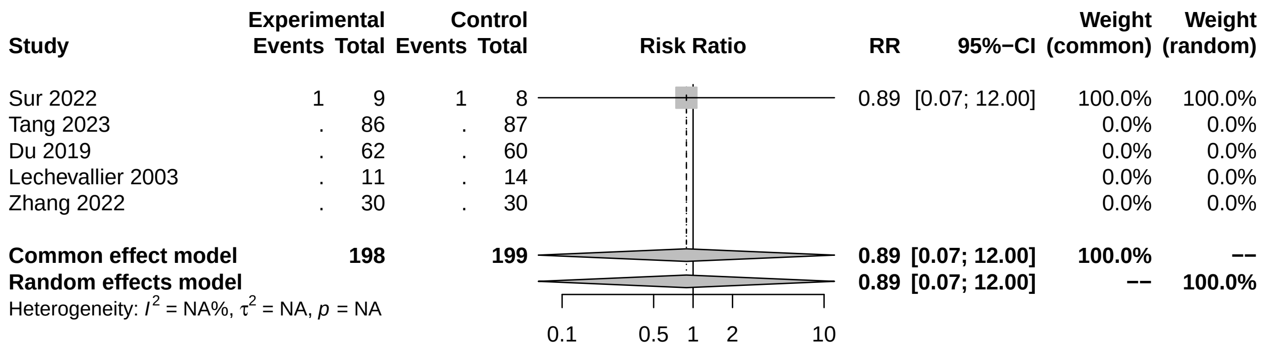

6.22.3 Trim and Fill

```
#trimfill(clav_i_ii_metabin)
#forest(trimfill(clav_i_ii_metabin),
#       sortvar = TE)
```

6.22.4 Funnel plot

```
#funnel(trimfill(clav_i_ii_metabin))
```

6.22.5 Baujat

```
#baujat(clav_i_ii_metabin, pos = 1)
```

6.22.6 Leave one out

```
#metainf(clav_i_ii_metabin)
```

6.23 Clavien III-V

6.23.1 Meta-analysis

```
clav_iii_v_metabin <- metabin(data = rct_only,
                         event.c = clav_iii_v_n_control,
                       n.c = sample_size_control,
                       event.e = clav_iii_v_n_suction,
                       n.e = sample_size_suction,
                       studlab = author_year)
clav_iii_v_metabin
```

```
## Number of studies: k = 0
## Number of observations: o = 397
## Number of events: e = 0
##
##                       RR 95%-CI  z p-value
## Common effect model  NA         --      --
## Random effects model NA         --      --
##
## Quantifying heterogeneity:
##  tau^2 = NA; tau = NA; I^2 = NA; H = NA
##
## Details on meta-analytical method:
## - Mantel-Haenszel method
## - Inverse variance method
## - Restricted maximum-likelihood estimator for tau^2
```

6.23.2 Forest plot

```r
forest(clav_iii_v_metabin,
       sortvar = TE)
```

Study	Experimental Events	Experimental Total	Control Events	Control Total	Risk Ratio	RR	95%−CI	Weight (common)	Weight (random)
Sur 2022	0	9	0	8				0.0%	0.0%
Tang 2023	.	86	.	87				0.0%	0.0%
Du 2019	.	62	.	60				0.0%	0.0%
Lechevallier 2003	.	11	.	14				0.0%	0.0%
Zhang 2022	0	30	0	30				0.0%	0.0%
**Common effect model**		**198**		**199**				**100.0%**	**−−**
**Random effects model**								**−−**	**100.0%**

Heterogeneity: I^2 = NA%, τ^2 = NA, p = NA

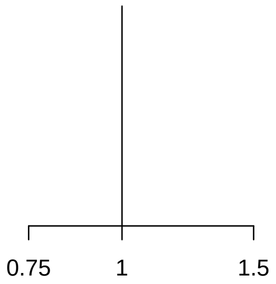

6.23.3 Trim and Fill

```r
#trimfill(clav_iii_v_metabin)
#forest(trimfill(clav_iii_v_metabin),
#       sortvar = TE)
```

6.23.4 Funnel plot

```
#funnel(trimfill(clav_iii_v_metabin))
```

6.23.5 Baujat

```
#baujat(clav_iii_v_metabin, pos = 1)
```

6.23.6 Leave one out

```
#metainf(clav_iii_v_metabin)
```

7 RCT Only Summary Forest plots

```
overall <- cbind(
  "Outcome" = c(
    "Immediate SFR",
    "Final SFR",
    "Auxiliary Treatment",
    "Overall Complications",
    "Fever",
    "Infection",
    "Sepsis",
    "Abscess",
    "Haematoma",
    "Pain",
    "Stricture",
    "Embolisation",
    "Transfusion",
    "CD I",
    "CD II",
    "CD III",
    "CD IV",
    "CD I-II",
    "CD III-V",
    "Operative time",
    "VAS",
    "Length of Stay"
  ),
  "type" = c(
    "binary",
    "binary",
    "binary",
    "binary",
    "binary",
    "binary",
    "binary",
    "binary",
    "binary",
    "binary",
    "binary",
    "binary",
    "binary",
    "binary",
    "binary",
    "binary",
    "binary",
    "binary",
    "binary",
    "cont",
    "cont",
    "cont"
  ),
  "n_studies" = c(
    immediate_sfr_metabin$k,
```

```
  sfr_metabin$k,
aux_rx_metabin$k,
comp_metabin$k,
fever_metabin$k,
infection_metabin$k,
sepsis_metabin$k,
abscess_metabin$k,
haematoma_metabin$k,
pain_metabin$k,
stricture_metabin$k,
ir_embolisation_metabin$k,
transfusion_metabin$k,
clav_i_metabin$k,
clav_ii_metabin$k,
clav_iii_metabin$k,
clav_iv_metabin$k,
clav_i_ii_metabin$k,
clav_iii_v_metabin$k,
or_time_metacont$k,
vas_metacont$k,
los_metacont$k),
"es" = c(
  immediate_sfr_metabin$TE.random %>% exp(),
  sfr_metabin$TE.random %>% exp(),
aux_rx_metabin$TE.random %>% exp(),
comp_metabin$TE.random %>% exp(),
fever_metabin$TE.random %>% exp(),
infection_metabin$TE.random %>% exp(),
sepsis_metabin$TE.random %>% exp(),
abscess_metabin$TE.random %>% exp(),
haematoma_metabin$TE.random %>% exp(),
pain_metabin$TE.random %>% exp(),
stricture_metabin$TE.random %>% exp(),
ir_embolisation_metabin$TE.random %>% exp(),
transfusion_metabin$TE.random %>% exp(),
clav_i_metabin$TE.random %>% exp(),
clav_ii_metabin$TE.random %>% exp(),
clav_iii_metabin$TE.random %>% exp(),
clav_iv_metabin$TE.random %>% exp(),
clav_i_ii_metabin$TE.random %>% exp(),
clav_iii_v_metabin$TE.random %>% exp(),
or_time_metacont$TE.random,
vas_metacont$TE.random,
los_metacont$TE.random),
"lower_ci" = c(
  immediate_sfr_metabin$lower.random %>% exp(),
  sfr_metabin$lower.random %>% exp(),
aux_rx_metabin$lower.random %>% exp(),
comp_metabin$lower.random %>% exp(),
fever_metabin$lower.random %>% exp(),
infection_metabin$lower.random %>% exp(),
sepsis_metabin$lower.random %>% exp(),
abscess_metabin$lower.random %>% exp(),
```

```r
haematoma_metabin$lower.random %>% exp(),
pain_metabin$lower.random %>% exp(),
stricture_metabin$lower.random %>% exp(),
ir_embolisation_metabin$lower.random %>% exp(),
transfusion_metabin$lower.random %>% exp(),
clav_i_metabin$lower.random %>% exp(),
clav_ii_metabin$lower.random %>% exp(),
clav_iii_metabin$lower.random %>% exp(),
clav_iv_metabin$lower.random %>% exp(),
clav_i_ii_metabin$lower.random %>% exp(),
clav_iii_v_metabin$lower.random %>% exp(),
or_time_metacont$lower.random,
vas_metacont$lower.random,
los_metacont$lower.random),
"upper_ci" = c(immediate_sfr_metabin$upper.random %>% exp(),
               sfr_metabin$upper.random %>% exp(),
aux_rx_metabin$upper.random %>% exp(),
comp_metabin$upper.random %>% exp(),
fever_metabin$upper.random %>% exp(),
infection_metabin$upper.random %>% exp(),
sepsis_metabin$upper.random %>% exp(),
abscess_metabin$upper.random %>% exp(),
haematoma_metabin$upper.random %>% exp(),
pain_metabin$upper.random %>% exp(),
stricture_metabin$upper.random %>% exp(),
ir_embolisation_metabin$upper.random %>% exp(),
transfusion_metabin$upper.random %>% exp(),
clav_i_metabin$upper.random %>% exp(),
clav_ii_metabin$upper.random %>% exp(),
clav_iii_metabin$upper.random %>% exp(),
clav_iv_metabin$upper.random %>% exp(),
clav_i_ii_metabin$upper.random %>% exp(),
clav_iii_v_metabin$upper.random %>% exp(),
or_time_metacont$upper.random,
vas_metacont$upper.random,
los_metacont$upper.random),
"p" = c(
  immediate_sfr_metabin$pval.random,
  sfr_metabin$pval.random,
aux_rx_metabin$pval.random,
comp_metabin$pval.random,
fever_metabin$pval.random,
infection_metabin$pval.random,
sepsis_metabin$pval.random,
abscess_metabin$pval.random,
haematoma_metabin$pval.random,
pain_metabin$pval.random,
stricture_metabin$pval.random,
ir_embolisation_metabin$pval.random,
transfusion_metabin$pval.random,
clav_i_metabin$pval.random,
clav_ii_metabin$pval.random,
clav_iii_metabin$pval.random,
```

```r
clav_iv_metabin$pval.random,
clav_i_ii_metabin$pval.random,
clav_iii_v_metabin$pval.random,
or_time_metacont$pval.random,
vas_metacont$pval.random,
los_metacont$pval.random)) %>% as_tibble() %>% drop_na(es)

overall$es <- as.numeric(overall$es)
overall$es <- round(overall$es, digits = 2)

overall$lower_ci <- as.numeric(overall$lower_ci)
overall$lower_ci <- round(overall$lower_ci, digits = 2)

overall$upper_ci <- as.numeric(overall$upper_ci)
overall$upper_ci <- round(overall$upper_ci, digits = 2)

overall$p <- as.numeric(overall$p)
overall$p <- round(overall$p, digits = 2)
overall$p <- ifelse(overall$p<0.001, "<0.001", overall$p)
```

7.1 Summary Table of number of studies for each outcome included in meta-analysis

```
overall %>% subset(select = c(Outcome, n_studies)) %>% gt() %>% tab_header(title = "Summary table of Nu
```

Summary table of Number of Studies for Each Outcome Meta-Analyis

Outcome	Studies, n
Immediate SFR	9
Final SFR	4
Auxiliary Treatment	1
Overall Complications	4
Fever	3
Infection	4
Sepsis	1
Pain	1
Stricture	1
CD I	1
CD II	1
CD I-II	1
Operative time	5
VAS	1
Length of Stay	2

7.2 Summary Forest plot of Continuous outcomes

7.2.1 Continuous Outcomes Table

md = mean difference lb = lower bound of 95% confidence interval ub = upper bound of 95% confidence interval tf = trim and fill

```
overall_continuous <-
  overall %>% subset(type ==  "cont") %>% subset(select = c(Outcome,
                                                            n_studies,
                                                            es,
                                                            lower_ci,
                                                            upper_ci,
                                                            p)) %>% as_tibble()
overall_continuous %>% gt() %>% tab_header(title = "Summary Table for Continuous Outcomes") %>% cols_me
```

Summary Table for Continuous Outcomes

Outcome	Studies, n	MD (95% CI)	p
Operative time	5	-6.87 (-18.71-4.96)	0.25
VAS	1	-0.34 (-0.65–0.03)	0.03
Length of Stay	2	-0.48 (-1.27-0.30)	0.23

7.2.2 Continuous Forest plot

```
continuous_plot <- overall_continuous %>%
  forestplot(
    mean = es,
    lower = lower_ci,
    upper = upper_ci,
    labeltext = c(Outcome, n_studies, es, lower_ci, upper_ci, p),
  zero = 0,
  vertices = TRUE,
  title = "A. Forest plot of MA Outcomes for Continuous Outcomes",
  clip = c(-4, 2),
  xlab = "MD [95% CI]",
  graph.pos = 3
) %>% fp_set_style(
    box = c("black"),
    line = "black",
    txt_gp = fpTxtGp(
      ticks = gpar(fontfamily = "", cex = 1),
      xlab  = gpar(fontfamily = "", cex = 1)
    )) %>% fp_add_lines("steelblue") %>%
  fp_add_header("Outcome",
                "Studies, n",
                  "MD",
                "[ 95%", " CI ]",
                "P-Value")  %>% fp_decorate_graph(box = TRUE) %>% fp_set_zebra_style("#EFEFEF")
continuous_plot
```

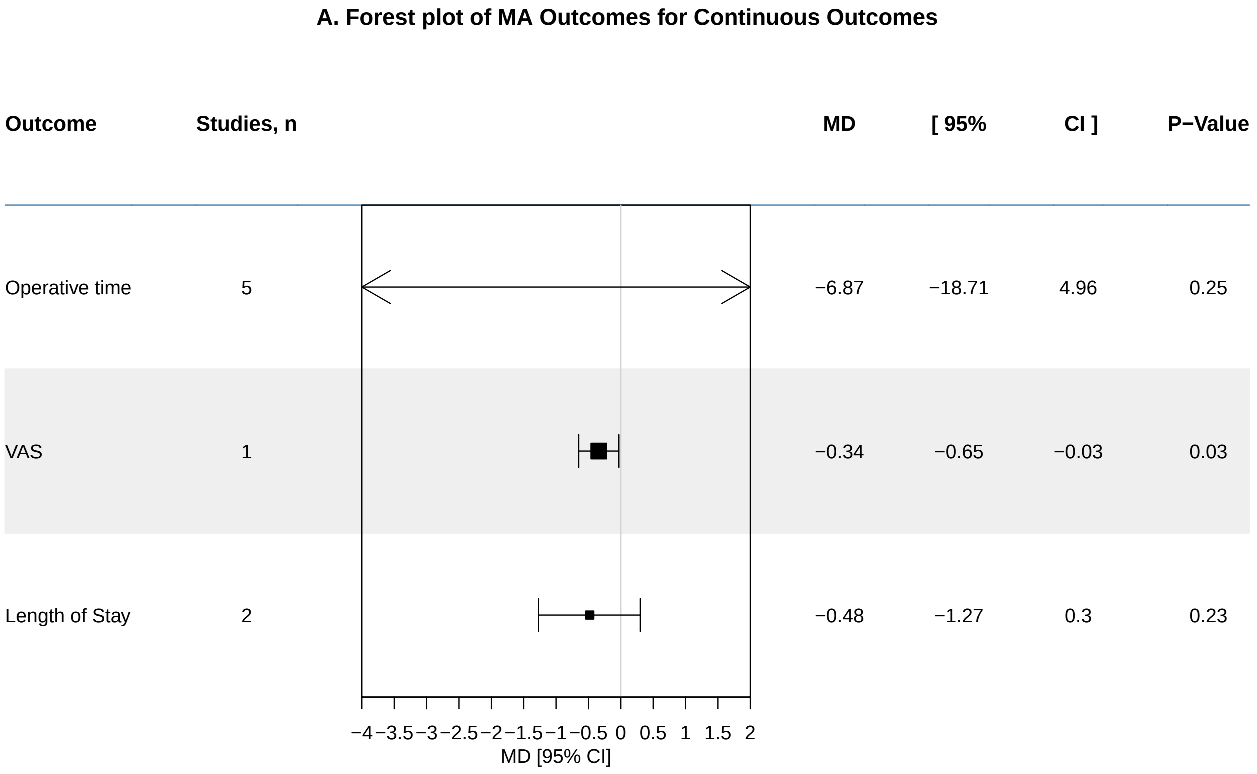

7.3 Summary Forest plot of Binary outcomes

7.3.1 Binary Outcomes Table

md = mean difference lb = lower bound of 95% confidence interval ub = upper bound of 95% confidence interval tf = trim and fill

```
overall_binary <-
  overall %>% subset(type ==  "binary") %>% subset(select = c(Outcome,
                                                            n_studies,
                                                            es,
                                                            lower_ci,
                                                            upper_ci,
                                                            p)) %>% as_tibble()
overall_binary %>% gt() %>% tab_header(title = "Summary Table for Binary Outcomes") %>% cols_merge(colu
```

Summary Table for Binary Outcomes

Outcome	Studies, n	RR (95% CI)	p
Immediate SFR	9	1.15 (0.99-1.34)	0.08
Final SFR	4	1.13 (0.98-1.29)	0.09
Auxiliary Treatment	1	0.06 (0.00-1.11)	0.06
Overall Complications	4	0.20 (0.09-0.41)	<0.001
Fever	3	0.22 (0.07-0.63)	<0.001
Infection	4	0.22 (0.08-0.61)	<0.001
Sepsis	1	0.11 (0.01-2.06)	0.14
Pain	1	0.16 (0.04-0.67)	0.01
Stricture	1	0.14 (0.01-2.62)	0.19
CD I	1	2.68 (0.13-57.47)	0.53
CD II	1	0.30 (0.01-6.39)	0.44
CD I-II	1	0.89 (0.07-12.00)	0.93

7.3.2 Binary Forest plot - Meta-Analysis

Reference = No Suction

```
binary_plot <- overall_binary %>%
  forestplot(
    mean = es,
    lower = lower_ci,
    upper = upper_ci,
    labeltext = c(Outcome, n_studies, es, lower_ci, upper_ci, p),
  zero = 1,
  vertices = TRUE,
  title = "A. Forest plot of MA Outcomes for Binary Outcomes",
  clip = c(-1.5, 1.5),
  xlab = "RR [95% CI]",
  graph.pos = 3
) %>% fp_set_style(
    box = c("black"),
    line = "black",
    txt_gp = fpTxtGp(
      ticks = gpar(fontfamily = "", cex = 1),
      xlab  = gpar(fontfamily = "", cex = 1)
    )) %>% fp_add_lines("steelblue") %>%
  fp_add_header("Outcome",
              "Studies, n",
                "RR",
              "[ 95%", " CI ]",
              "P-Value")  %>% fp_decorate_graph(box = TRUE) %>% fp_set_zebra_style("#EFEFEF")
binary_plot
```

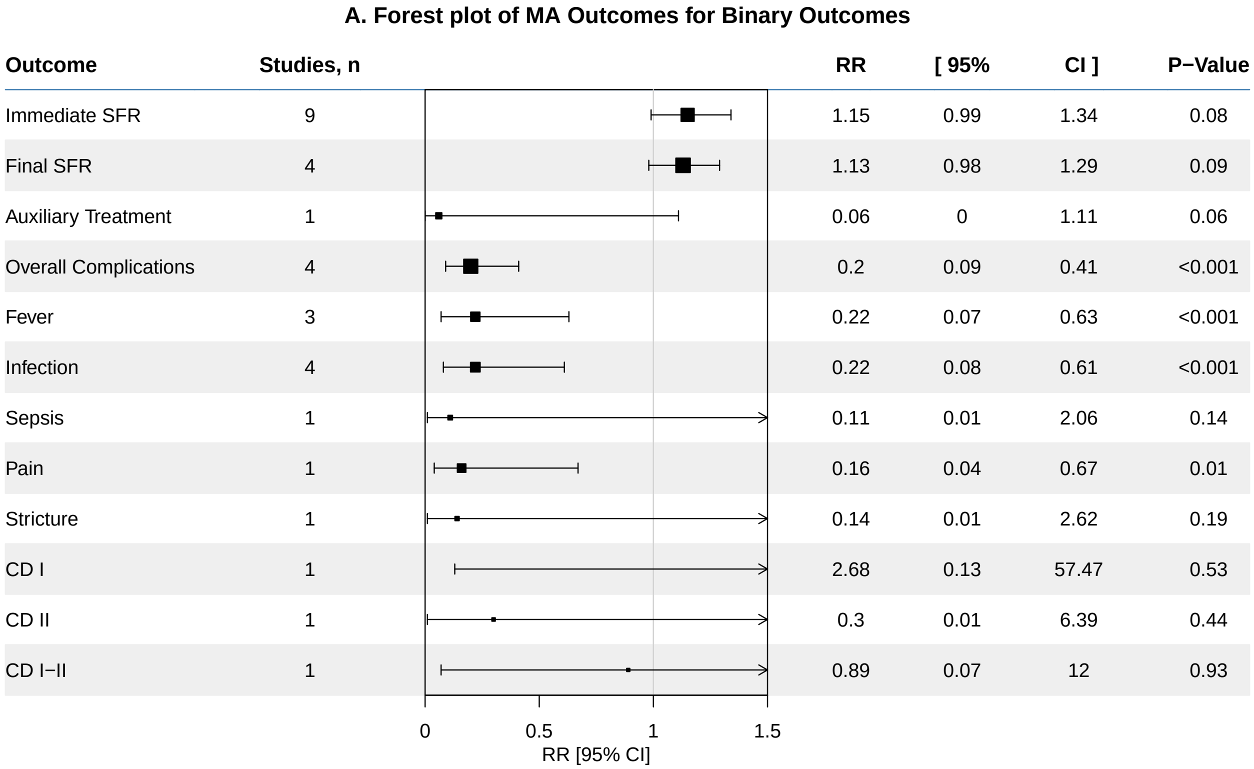

8 Semi-rigid Only Meta-Analysis Outcomes

```
semirigid_only <- suction_data %>% subset(control_group == "Semi-rigid URS")
```

8.1 Immediate SFR

8.1.1 Meta-analysis

```
immediate_sfr_metabin <- metabin(data = suction_data,
                         event.c = sfr_immediate_n_control,
                       n.c = sample_size_control,
                       event.e = sfr_immediate_n_suction,
                       n.e = sample_size_suction,
                       studlab = author_year)
immediate_sfr_metabin
```

```
## Number of studies: k = 9
## Number of observations: o = 2311
## Number of events: e = 979
##
##                           RR           95%-CI    z  p-value
## Common effect model  1.1581 [1.0834; 1.2380] 4.31 < 0.0001
## Random effects model 1.1478 [0.9854; 1.3370] 1.77   0.0765
##
## Quantifying heterogeneity:
##  tau^2 = 0.0365 [0.0088; 0.2318]; tau = 0.1910 [0.0939; 0.4814]
##  I^2 = 74.6% [50.9%; 86.9%]; H = 1.99 [1.43; 2.76]
##
## Test of heterogeneity:
##      Q d.f. p-value
##  31.55    8  0.0001
##
## Details on meta-analytical method:
## - Mantel-Haenszel method
## - Inverse variance method
## - Restricted maximum-likelihood estimator for tau^2
## - Q-Profile method for confidence interval of tau^2 and tau
```

8.1.2 Forest plot

```r
forest(immediate_sfr_metabin,
       sortvar = TE)
```

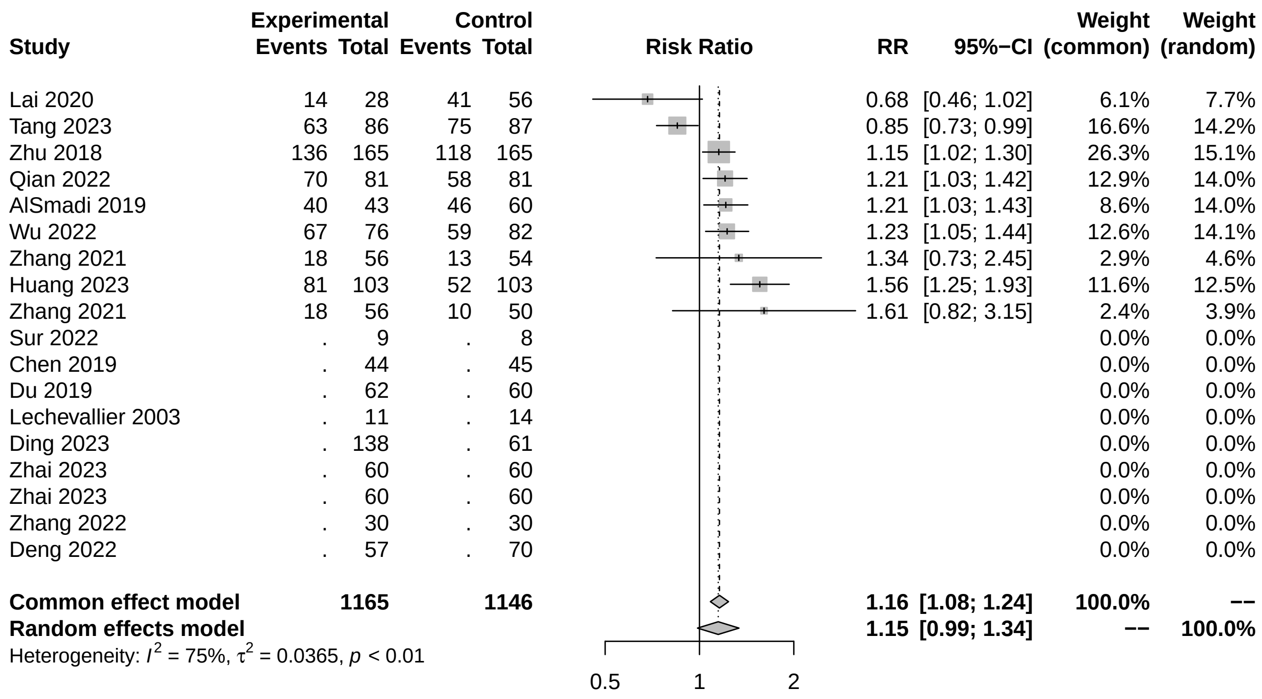

8.1.3 Trim and Fill

```
trimfill(immediate_sfr_metabin)
```

```
## Warning in trimfill.meta(immediate_sfr_metabin): 9 observation(s) dropped due
## to missing values

## Number of studies: k = 11 (with 2 added studies)
## Number of observations: o = 1744
## Number of events: e = 1140
##
##                          RR           95%-CI     z p-value
## Random effects model 1.0855 [0.9289; 1.2685] 1.03  0.3019
##
## Quantifying heterogeneity:
##  tau^2 = 0.0471 [0.0138; 0.2196]; tau = 0.2170 [0.1173; 0.4686]
##  I^2 = 76.9% [58.8%; 87.1%]; H = 2.08 [1.56; 2.78]
##
## Test of heterogeneity:
##      Q d.f.  p-value
##  43.33   10 < 0.0001
##
## Details on meta-analytical method:
## - Inverse variance method
## - Restricted maximum-likelihood estimator for tau^2
## - Q-Profile method for confidence interval of tau^2 and tau
## - Trim-and-fill method to adjust for funnel plot asymmetry (L-estimator)
```

```
forest(trimfill(immediate_sfr_metabin),
       sortvar = TE)
```

```
## Warning in trimfill.meta(immediate_sfr_metabin): 9 observation(s) dropped due
## to missing values
```

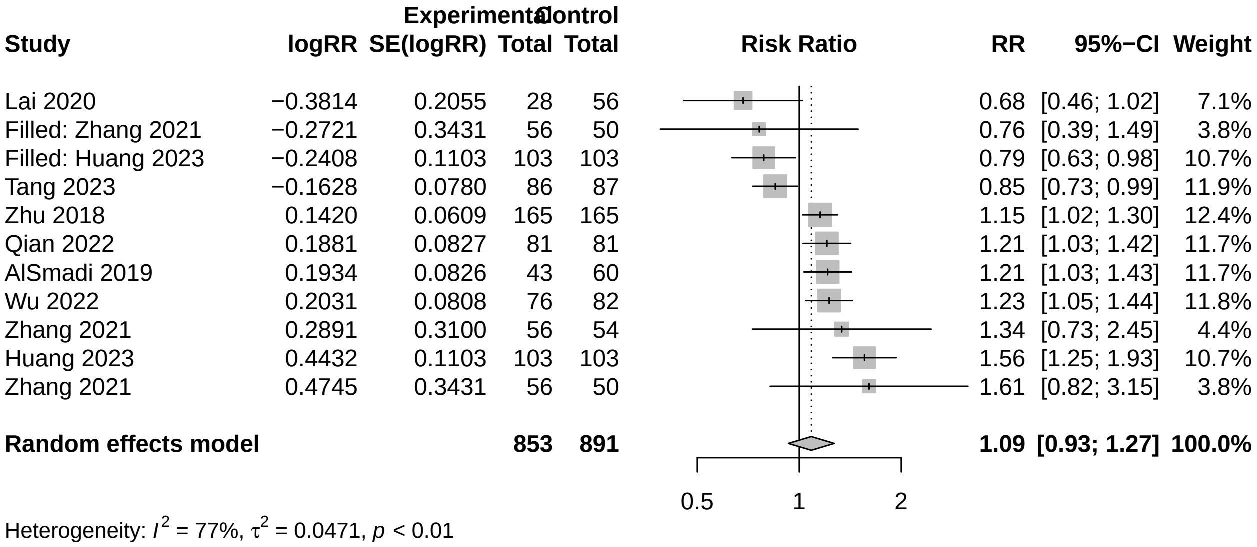

8.1.4 Funnel plot

```
funnel(trimfill(immediate_sfr_metabin))
```

```
## Warning in trimfill.meta(immediate_sfr_metabin): 9 observation(s) dropped due
## to missing values
```

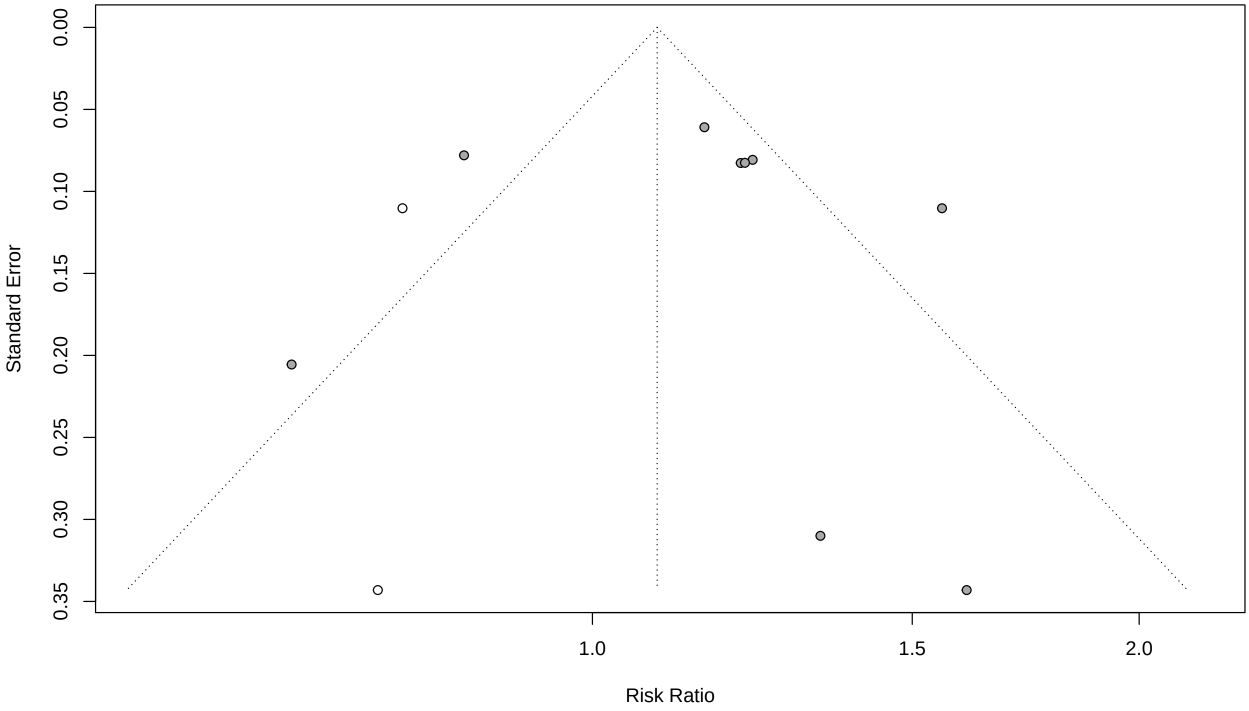

8.1.5 Baujat

```
baujat(immediate_sfr_metabin, pos = 1)
```

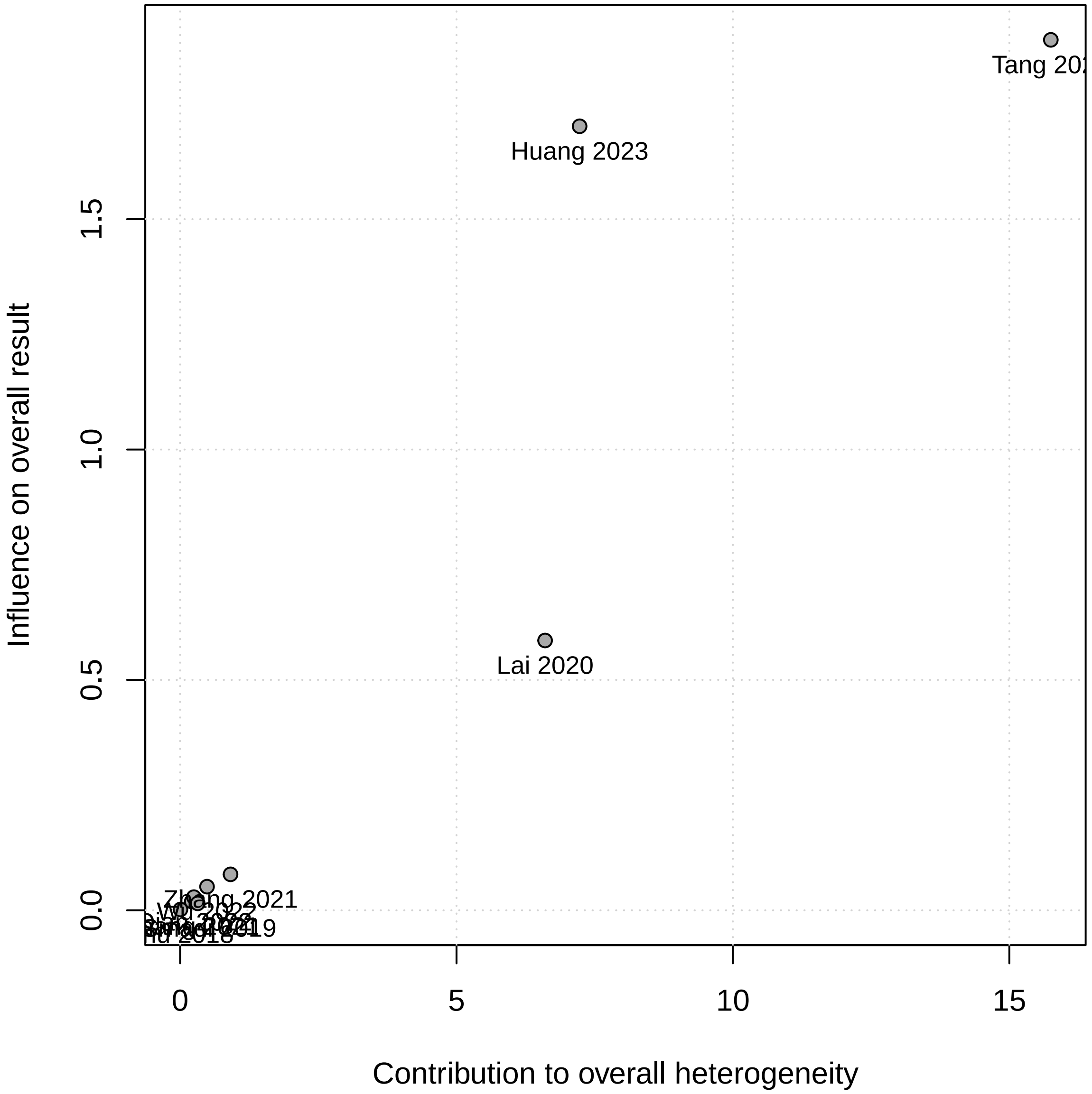

8.1.6 Leave one out

```
metainf(immediate_sfr_metabin)
```

```
## Influential analysis (common effect model)
##
##                                  RR           95%-CI  p-value   tau^2     tau
## Omitting Sur 2022            1.1581 [1.0834; 1.2380] < 0.0001  0.0365  0.1910
## Omitting Tang 2023           1.2195 [1.1329; 1.3128] < 0.0001  0.0076  0.0873
## Omitting Chen 2019           1.1581 [1.0834; 1.2380] < 0.0001  0.0365  0.1910
## Omitting Du 2019             1.1581 [1.0834; 1.2380] < 0.0001  0.0365  0.1910
## Omitting Lai 2020            1.1889 [1.1116; 1.2716] < 0.0001  0.0258  0.1607
## Omitting Zhu 2018            1.1601 [1.0710; 1.2565]   0.0003  0.0482  0.2196
## Omitting Zhang 2021          1.1473 [1.0741; 1.2254] < 0.0001  0.0376  0.1938
## Omitting Zhang 2021          1.1527 [1.0791; 1.2313] < 0.0001  0.0396  0.1990
## Omitting Lechevallier 2003   1.1581 [1.0834; 1.2380] < 0.0001  0.0365  0.1910
## Omitting Huang 2023          1.1057 [1.0315; 1.1853]   0.0046  0.0246  0.1568
## Omitting Wu 2022             1.1484 [1.0676; 1.2353]   0.0002  0.0464  0.2153
## Omitting Ding 2023           1.1581 [1.0834; 1.2380] < 0.0001  0.0365  0.1910
## Omitting Zhai 2023           1.1581 [1.0834; 1.2380] < 0.0001  0.0365  0.1910
## Omitting Zhai 2023           1.1581 [1.0834; 1.2380] < 0.0001  0.0365  0.1910
## Omitting Qian 2022           1.1508 [1.0700; 1.2377]   0.0002  0.0468  0.2164
## Omitting Zhang 2022          1.1581 [1.0834; 1.2380] < 0.0001  0.0365  0.1910
## Omitting Deng 2022           1.1581 [1.0834; 1.2380] < 0.0001  0.0365  0.1910
## Omitting AlSmadi 2019        1.1529 [1.0734; 1.2383] < 0.0001  0.0467  0.2160
##
## Pooled estimate              1.1581 [1.0834; 1.2380] < 0.0001  0.0365  0.1910
##                                 I^2
## Omitting Sur 2022             74.6%
## Omitting Tang 2023            51.5%
## Omitting Chen 2019            74.6%
## Omitting Du 2019              74.6%
## Omitting Lai 2020             72.2%
## Omitting Zhu 2018             77.8%
## Omitting Zhang 2021           77.1%
## Omitting Zhang 2021           77.6%
## Omitting Lechevallier 2003    74.6%
## Omitting Huang 2023           69.4%
## Omitting Wu 2022              77.2%
## Omitting Ding 2023            74.6%
## Omitting Zhai 2023            74.6%
## Omitting Zhai 2023            74.6%
## Omitting Qian 2022            77.4%
## Omitting Zhang 2022           74.6%
## Omitting Deng 2022            74.6%
## Omitting AlSmadi 2019         77.4%
##
## Pooled estimate               74.6%
##
## Details on meta-analytical method:
## - Mantel-Haenszel method
## - Restricted maximum-likelihood estimator for tau^2
```

8.2 Final SFR

8.2.1 Meta-analysis

```
sfr_metabin <- metabin(data = semirigid_only,
                         event.c = sfr_final_n_control,
                       n.c = sample_size_control,
                       event.e = sfr_final_n_suction,
                       n.e = sample_size_suction,
                       studlab = author_year)
sfr_metabin
```

```
## Number of studies: k = 6
## Number of observations: o = 754
## Number of events: e = 653
##
##                          RR           95%-CI    z  p-value
## Common effect model  1.1622 [1.1042; 1.2233] 5.75 < 0.0001
## Random effects model 1.1577 [1.0743; 1.2476] 3.84   0.0001
##
## Quantifying heterogeneity:
##  tau^2 = 0.0046 [0.0000; 0.0380]; tau = 0.0679 [0.0000; 0.1949]
##  I^2 = 57.3% [0.0%; 82.7%]; H = 1.53 [1.00; 2.41]
##
## Test of heterogeneity:
##      Q d.f. p-value
##  11.70    5  0.0391
##
## Details on meta-analytical method:
## - Mantel-Haenszel method
## - Inverse variance method
## - Restricted maximum-likelihood estimator for tau^2
## - Q-Profile method for confidence interval of tau^2 and tau
## - Continuity correction of 0.5 in studies with zero cell frequencies
```

8.2.2 Forest plot

```r
forest(sfr_metabin,
       sortvar = TE)
```

Study	Experimental Events	Experimental Total	Control Events	Control Total	Risk Ratio	RR	95%−CI	Weight (common)	Weight (random)
Wu 2022	72	76	76	82		1.02	[0.94; 1.11]	24.3%	23.1%
AlSmadi 2019	41	43	51	60		1.12	[0.99; 1.27]	14.1%	16.8%
Zhai 2023	57	60	48	60		1.19	[1.03; 1.36]	15.9%	15.1%
Zhai 2023	58	60	48	60		1.21	[1.06; 1.38]	15.9%	15.6%
Du 2019	62	62	49	60		1.22	[1.09; 1.38]	16.7%	17.6%
Zhang 2021	54	56	37	50		1.30	[1.10; 1.55]	13.0%	11.8%
Lechevallier 2003	.	11	.	14				0.0%	0.0%
**Common effect model**		**368**		**386**		**1.16**	**[1.10; 1.22]**	**100.0%**	**−−**
**Random effects model**						**1.16**	**[1.07; 1.25]**	**−−**	**100.0%**

Heterogeneity: $I^2 = 57\%$, $\tau^2 = 0.0046$, $p = 0.04$

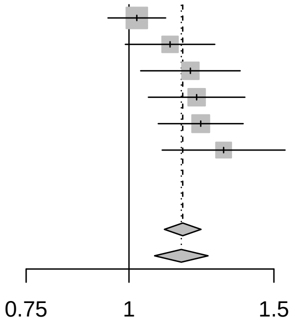

8.2.3 Trim and Fill

```r
trimfill(sfr_metabin)
```

```
## Warning in trimfill.meta(sfr_metabin): 1 observation(s) dropped due to missing
## values
```

```
## Number of studies: k = 9 (with 3 added studies)
## Number of observations: o = 1077
## Number of events: e = 961
##
##                             RR           95%-CI    z p-value
## Random effects model 1.0848 [0.9981; 1.1791] 1.91  0.0555
##
## Quantifying heterogeneity:
##  tau^2 = 0.0117 [0.0029; 0.0615]; tau = 0.1081 [0.0538; 0.2480]
##  I^2 = 71.6% [43.9%; 85.6%]; H = 1.88 [1.34; 2.63]
##
## Test of heterogeneity:
##      Q d.f. p-value
##  28.14    8  0.0004
##
## Details on meta-analytical method:
## - Inverse variance method
## - Restricted maximum-likelihood estimator for tau^2
## - Q-Profile method for confidence interval of tau^2 and tau
## - Trim-and-fill method to adjust for funnel plot asymmetry (L-estimator)
```

```r
forest(trimfill(sfr_metabin),
       sortvar = TE)
```

```
## Warning in trimfill.meta(sfr_metabin): 1 observation(s) dropped due to missing
## values
```

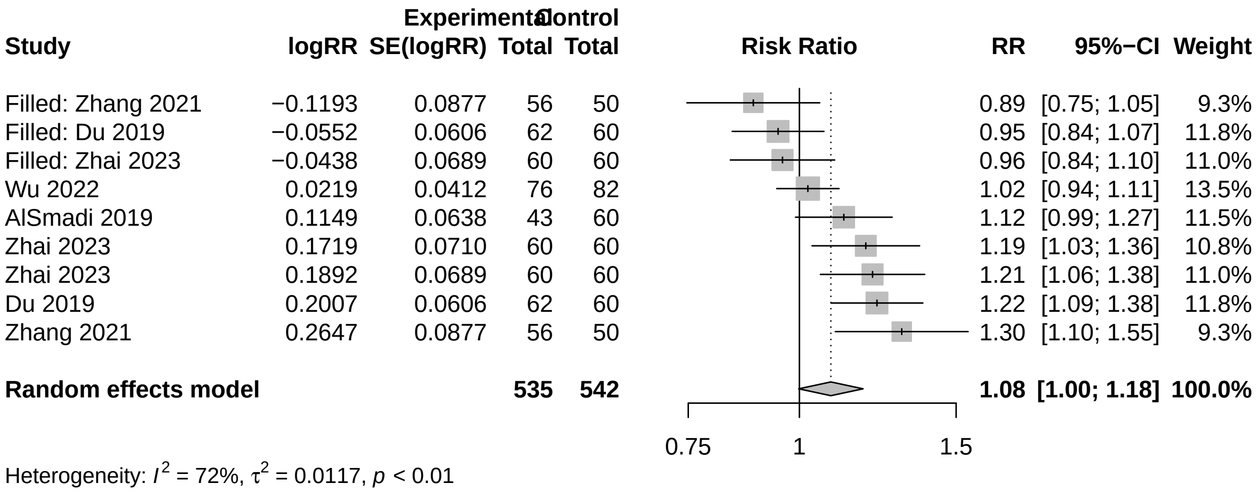

8.2.4 Funnel plot

```
funnel(trimfill(sfr_metabin))
```

```
## Warning in trimfill.meta(sfr_metabin): 1 observation(s) dropped due to missing
## values
```

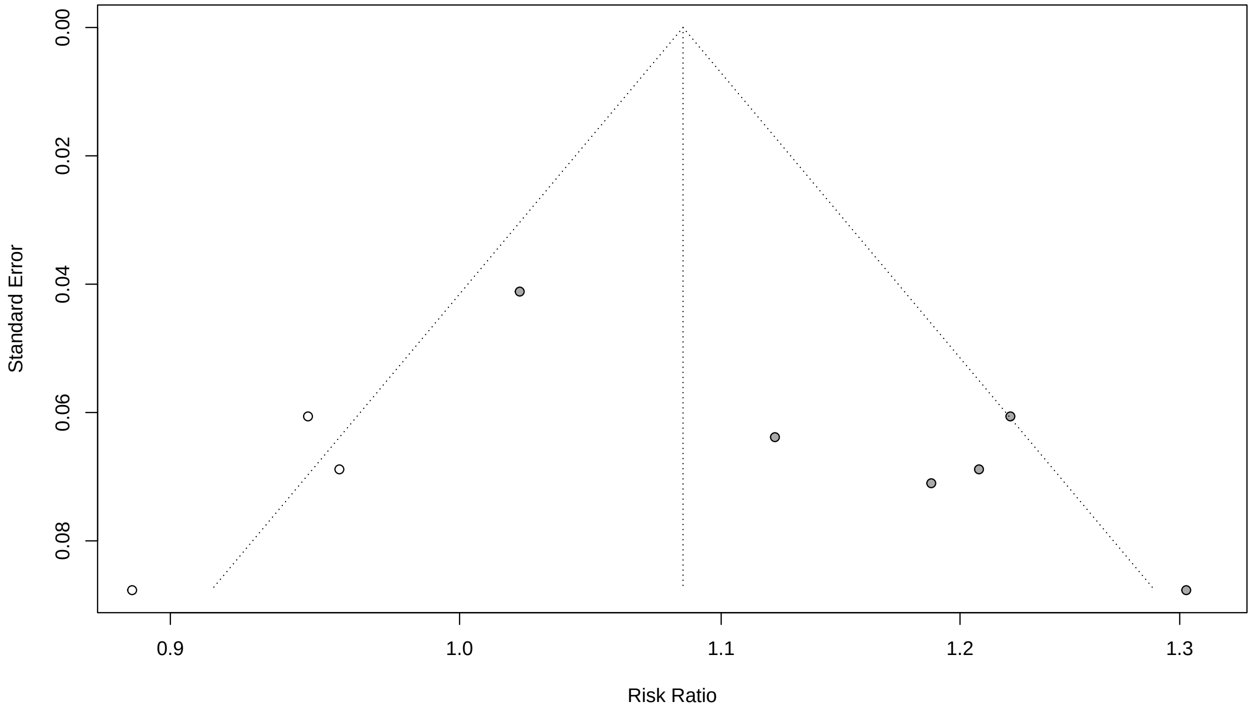

8.2.5 Baujat

```
baujat(sfr_metabin, pos = 1)
```

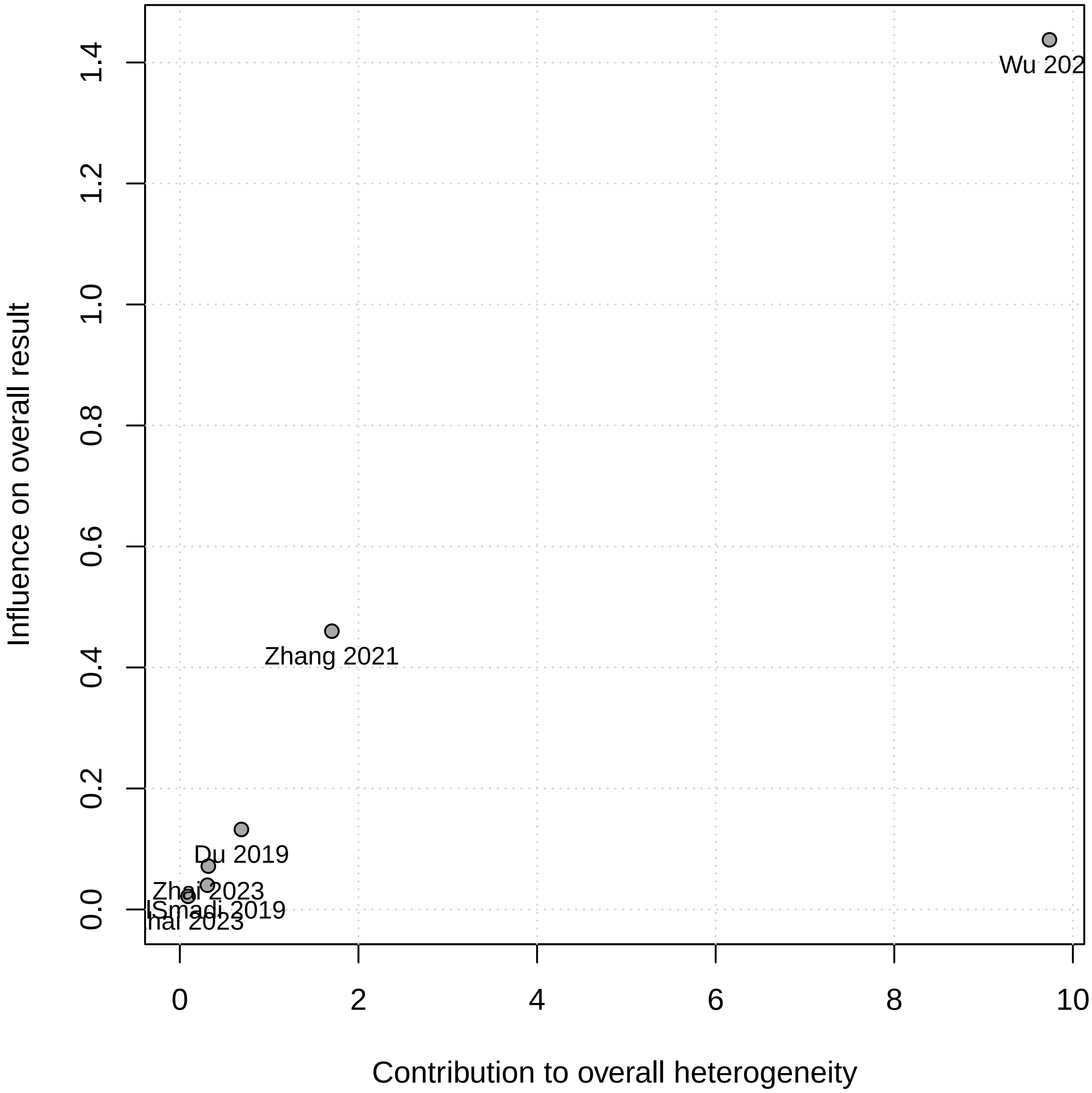

8.2.6 Leave one out

```
metainf(sfr_metabin)
```

```
## Influential analysis (common effect model)
##
##                                 RR           95%-CI  p-value   tau^2     tau
## Omitting Du 2019            1.1501 [1.0871; 1.2168] < 0.0001  0.0052  0.0723
## Omitting Zhang 2021         1.1412 [1.0826; 1.2030] < 0.0001  0.0040  0.0629
## Omitting Lechevallier 2003   1.1622 [1.1042; 1.2233] < 0.0001  0.0046  0.0679
## Omitting Wu 2022            1.2072 [1.1346; 1.2844] < 0.0001  0.0000  0.0000
## Omitting Zhai 2023          1.1535 [1.0914; 1.2191] < 0.0001  0.0056  0.0747
## Omitting Zhai 2023          1.1574 [1.0956; 1.2228] < 0.0001  0.0059  0.0768
## Omitting AlSmadi 2019       1.1689 [1.1053; 1.2362] < 0.0001  0.0062  0.0788
##
## Pooled estimate             1.1622 [1.1042; 1.2233] < 0.0001  0.0046  0.0679
##                                I^2
## Omitting Du 2019             59.1%
## Omitting Zhang 2021          55.1%
## Omitting Lechevallier 2003   57.3%
## Omitting Wu 2022              0.0%
## Omitting Zhai 2023           62.5%
## Omitting Zhai 2023           64.2%
## Omitting AlSmadi 2019        65.7%
##
## Pooled estimate              57.3%
##
## Details on meta-analytical method:
## - Mantel-Haenszel method
## - Restricted maximum-likelihood estimator for tau^2
```

8.3 OR time

8.3.1 Meta-analysis

```
or_time_metacont <- metacont(data = semirigid_only,
                             mean.c = or_time_min_mean_control,
                             sd.c = or_time_min_sd_control,
                             mean.e = or_time_min_mean_suction,
                             sd.e = or_time_min_sd_suction,
                             n.e = sample_size_suction,
                             n.c = sample_size_control,
                       studlab = author_year)
or_time_metacont
```

```
## Number of studies: k = 7
## Number of observations: o = 754
##
##                            MD              95%-CI      z  p-value
## Common effect model  -8.2542 [ -9.4297; -7.0788] -13.76 < 0.0001
## Random effects model -6.8657 [-14.0651;  0.3336]  -1.87   0.0616
##
## Quantifying heterogeneity:
##  tau^2 = 86.2668 [31.6285; 469.1099]; tau = 9.2880 [5.6239; 21.6589]
##  I^2 = 95.8% [93.4%; 97.4%]; H = 4.89 [3.89; 6.15]
##
## Test of heterogeneity:
##       Q d.f.  p-value
##  143.67    6 < 0.0001
##
## Details on meta-analytical method:
## - Inverse variance method
## - Restricted maximum-likelihood estimator for tau^2
## - Q-Profile method for confidence interval of tau^2 and tau
```

8.3.2 Forest plot

```r
forest(or_time_metacont,
       sortvar = TE)
```

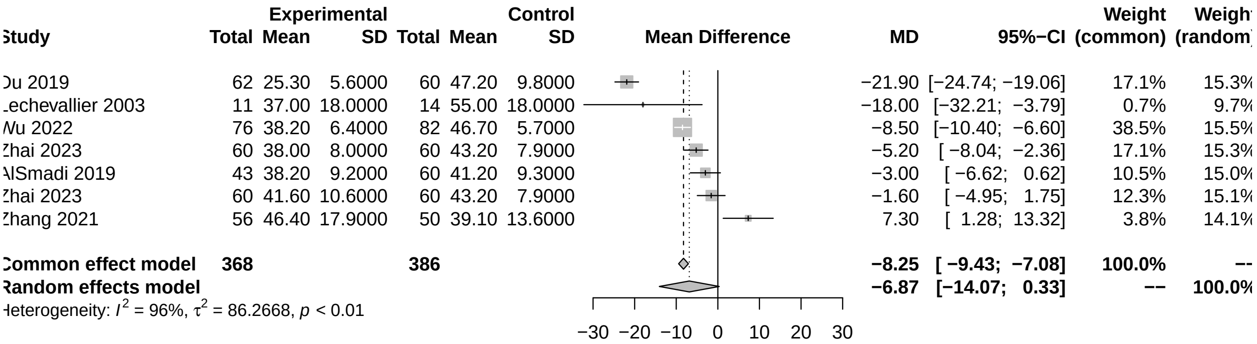

8.3.3 Trim and Fill

```r
trimfill(or_time_metacont)
```

```
## Number of studies: k = 8 (with 1 added studies)
## Number of observations: o = 860
##
##                             MD              95%-CI     z p-value
## Random effects model -9.1790 [-16.8799; -1.4781] -2.34  0.0195
##
## Quantifying heterogeneity:
##  tau^2 = 114.8028 [45.5270; 506.6692]; tau = 10.7146 [6.7474; 22.5093]
##  I^2 = 95.9% [93.8%; 97.3%]; H = 4.96 [4.02; 6.12]
##
## Test of heterogeneity:
##       Q d.f.  p-value
##  172.49    7 < 0.0001
##
## Details on meta-analytical method:
## - Inverse variance method
## - Restricted maximum-likelihood estimator for tau^2
## - Q-Profile method for confidence interval of tau^2 and tau
## - Trim-and-fill method to adjust for funnel plot asymmetry (L-estimator)
```

```r
forest(trimfill(or_time_metacont),
       sortvar = TE)
```

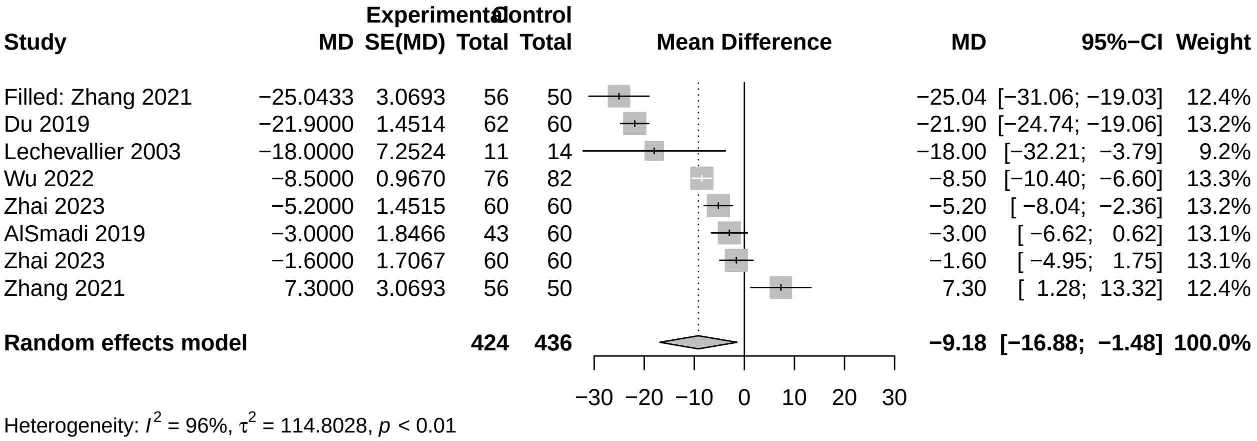

8.3.4 Funnel plot

```
funnel(trimfill(or_time_metacont))
```

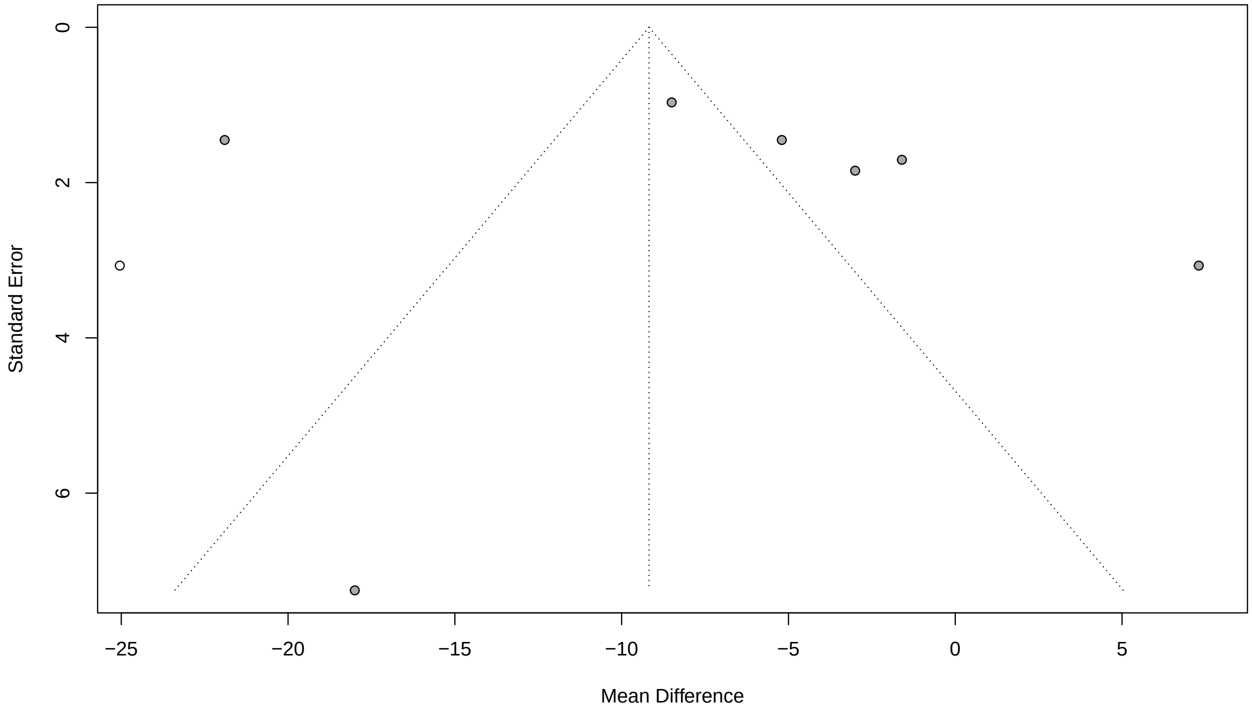

8.3.5 Baujat

```
baujat(or_time_metacont, pos = 1)
```

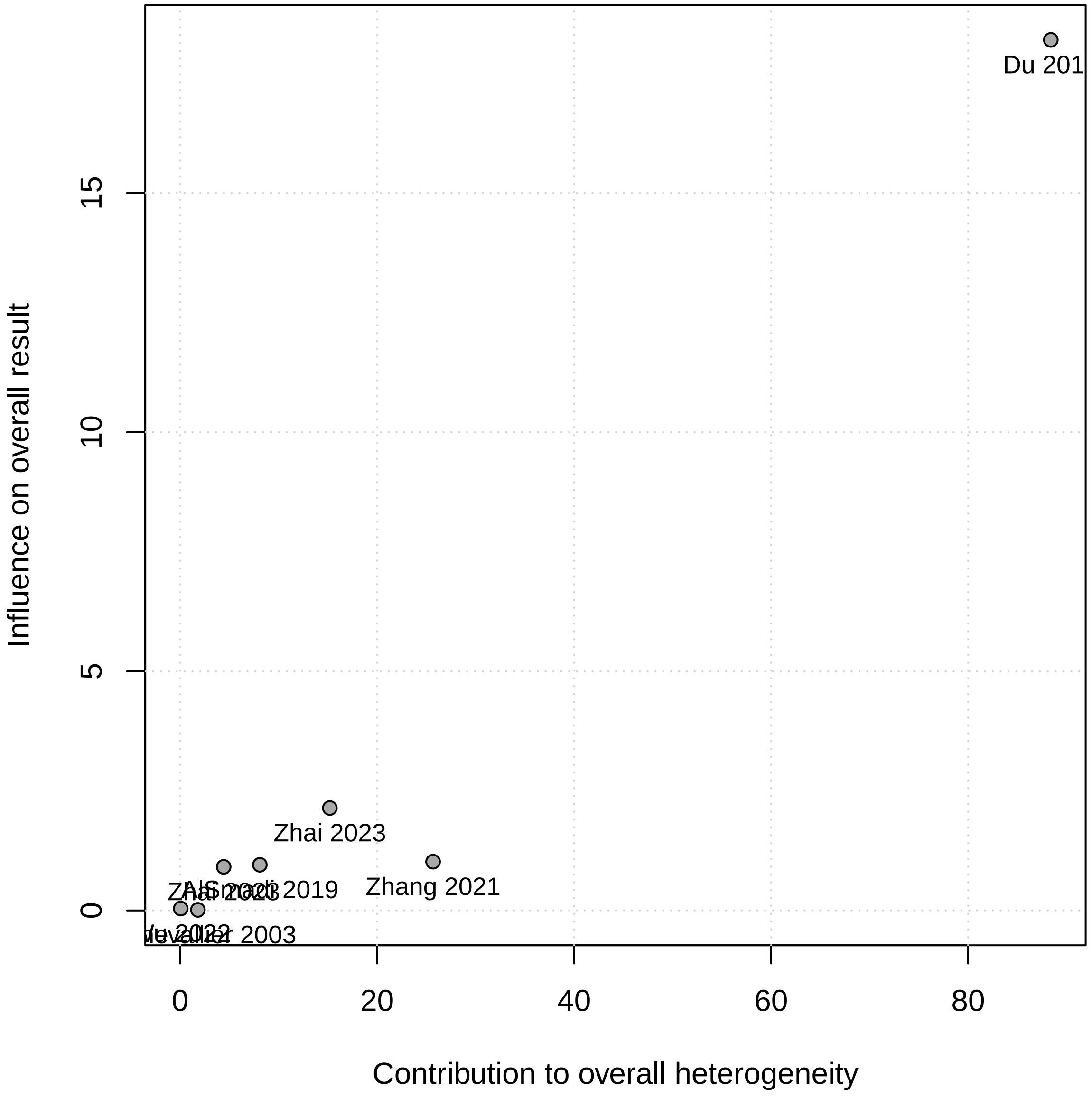

8.3.6 Leave one out

```
metainf(or_time_metacont)
```

```
## Influential analysis (common effect model)
##
##                                   MD              95%-CI  p-value     tau^2
## Omitting Du 2019             -5.4447 [ -6.7355; -4.1539] < 0.0001   32.4249
## Omitting Zhang 2021          -8.8716 [-10.0701; -7.6731] < 0.0001   62.0728
## Omitting Lechevallier 2003   -8.1871 [ -9.3666; -7.0077] < 0.0001   86.9262
## Omitting Wu 2022             -8.1007 [ -9.5990; -6.6023] < 0.0001  106.0931
## Omitting Zhai 2023           -9.1916 [-10.4471; -7.9361] < 0.0001   99.7092
## Omitting Zhai 2023           -8.8830 [-10.1737; -7.5922] < 0.0001  106.1781
## Omitting AlSmadi 2019        -8.8738 [-10.1166; -7.6310] < 0.0001  102.9498
##
## Pooled estimate              -8.2542 [ -9.4297; -7.0788] < 0.0001   86.2668
##                                   tau    I^2
## Omitting Du 2019               5.6943  86.5%
## Omitting Zhang 2021            7.8786  95.7%
## Omitting Lechevallier 2003     9.3234  96.5%
## Omitting Wu 2022              10.3002  96.5%
## Omitting Zhai 2023             9.9854  96.0%
## Omitting Zhai 2023            10.3043  96.4%
## Omitting AlSmadi 2019         10.1464  96.3%
##
## Pooled estimate                9.2880  95.8%
##
## Details on meta-analytical method:
## - Inverse variance method
## - Restricted maximum-likelihood estimator for tau^2
```

8.4 Auxiliary Treatments

8.4.1 Meta-analysis

```
aux_rx_metabin <- metabin(data = semirigid_only,
                          event.c = auxillary_tx_n_control,
                        n.c = sample_size_control,
                        event.e = auxillary_tx_n_suction,
                        n.e = sample_size_suction,
                        studlab = author_year)
aux_rx_metabin
```

```
## Number of studies: k = 5
## Number of observations: o = 754
## Number of events: e = 64
##
##                           RR           95%-CI     z p-value
## Common effect model  0.5418 [0.3260; 0.9006] -2.36  0.0181
## Random effects model 0.5696 [0.1350; 2.4033] -0.77  0.4435
##
## Quantifying heterogeneity:
##  tau^2 = 1.9745 [0.2816; 24.2153]; tau = 1.4052 [0.5306; 4.9209]
##  I^2 = 75.7% [40.5%; 90.1%]; H = 2.03 [1.30; 3.18]
##
## Test of heterogeneity:
##      Q d.f. p-value
##  16.49    4  0.0024
##
## Details on meta-analytical method:
## - Mantel-Haenszel method
## - Inverse variance method
## - Restricted maximum-likelihood estimator for tau^2
## - Q-Profile method for confidence interval of tau^2 and tau
## - Continuity correction of 0.5 in studies with zero cell frequencies
```

8.4.2 Forest plot

```r
forest(aux_rx_metabin,
       sortvar = TE)
```

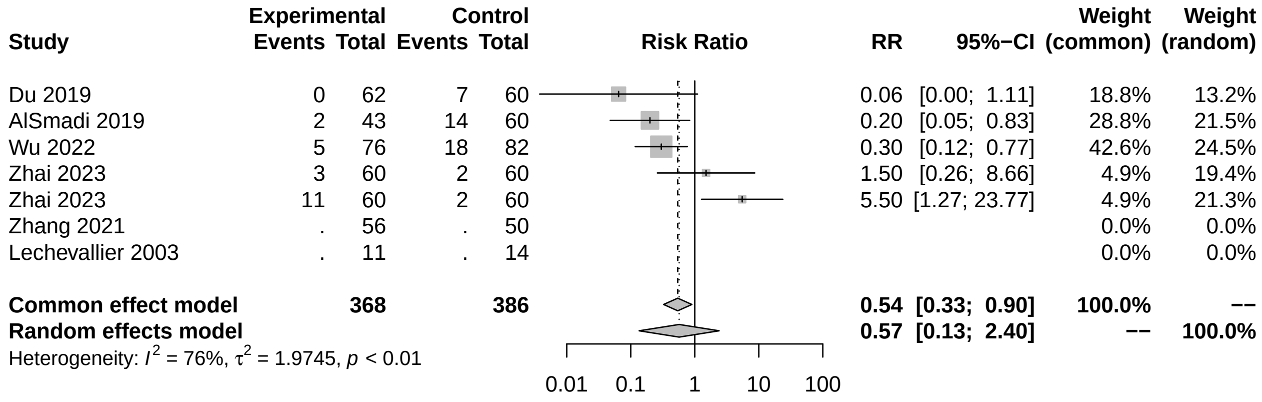

8.4.3 Trim and Fill

```r
trimfill(aux_rx_metabin)
```

```
## Warning in trimfill.meta(aux_rx_metabin): 2 observation(s) dropped due to
## missing values

## Number of studies: k = 5 (with 0 added studies)
## Number of observations: o = 623
## Number of events: e = 64
##
##                          RR            95%-CI     z p-value
## Random effects model 0.5696 [0.1350; 2.4033] -0.77  0.4435
##
## Quantifying heterogeneity:
##  tau^2 = 1.9745 [0.2816; 24.2153]; tau = 1.4052 [0.5306; 4.9209]
##  I^2 = 75.7% [40.5%; 90.1%]; H = 2.03 [1.30; 3.18]
##
## Test of heterogeneity:
##      Q d.f. p-value
##  16.49    4  0.0024
##
## Details on meta-analytical method:
## - Inverse variance method
## - Restricted maximum-likelihood estimator for tau^2
## - Q-Profile method for confidence interval of tau^2 and tau
## - Trim-and-fill method to adjust for funnel plot asymmetry (L-estimator)
```

```r
forest(trimfill(aux_rx_metabin),
       sortvar = TE)
```

```
## Warning in trimfill.meta(aux_rx_metabin): 2 observation(s) dropped due to
## missing values
```

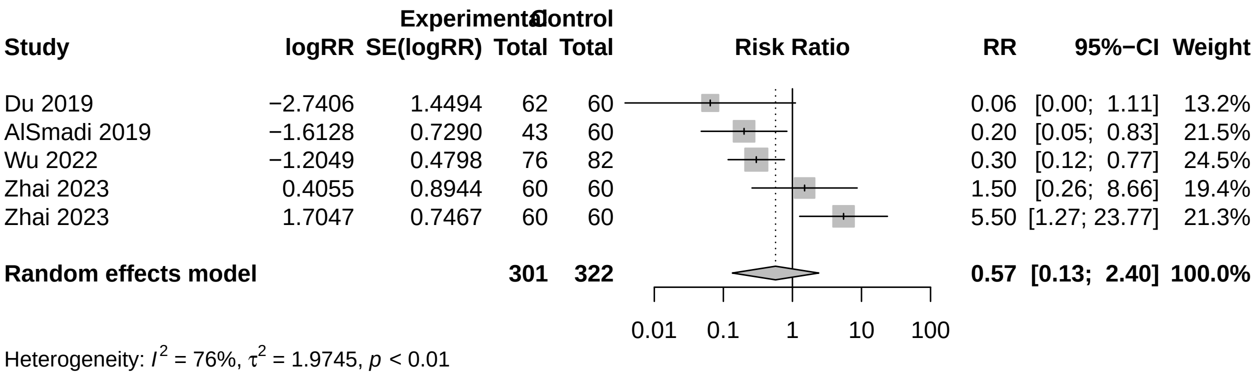

8.4.4 Funnel plot

```
funnel(trimfill(aux_rx_metabin))
```

```
## Warning in trimfill.meta(aux_rx_metabin): 2 observation(s) dropped due to
## missing values
```

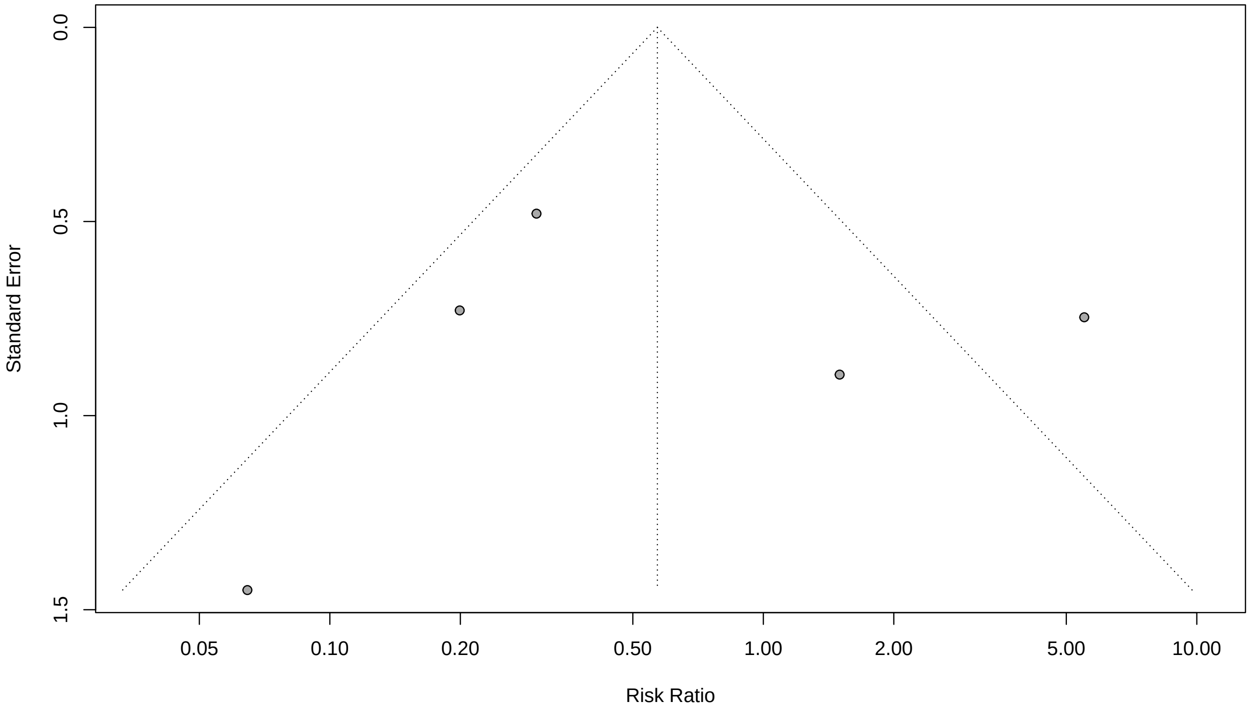

8.4.5 Baujat

```
baujat(aux_rx_metabin, pos = 1)
```

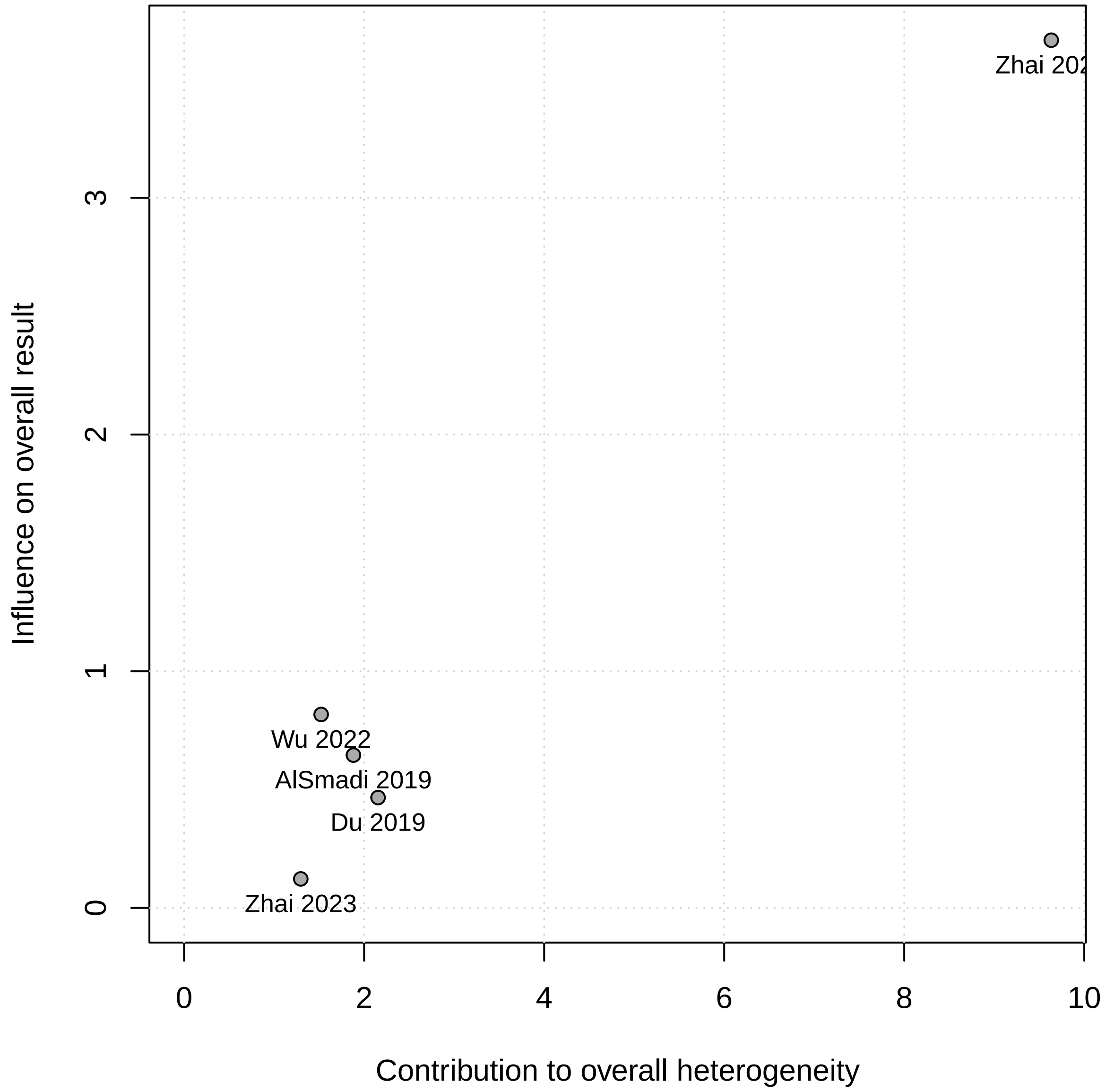

8.4.6 Leave one out

```
metainf(aux_rx_metabin)
```

```
## Influential analysis (common effect model)
##
##                                RR           95%-CI p-value   tau^2     tau
## Omitting Du 2019           0.6520 [0.3832; 1.1094]  0.1147  1.8370  1.3554
## Omitting Zhang 2021        0.5418 [0.3260; 0.9006]  0.0181  1.9745  1.4052
## Omitting Lechevallier 2003   0.5418 [0.3260; 0.9006]  0.0181  1.9745  1.4052
## Omitting Wu 2022           0.7217 [0.3877; 1.3434]  0.3036  2.7940  1.6715
## Omitting Zhai 2023         0.2851 [0.1477; 0.5501]  0.0002  0.1125  0.3354
## Omitting Zhai 2023         0.4922 [0.2874; 0.8428]  0.0098  2.5861  1.6081
## Omitting AlSmadi 2019      0.6802 [0.3905; 1.1845]  0.1733  2.3649  1.5378
##
## Pooled estimate            0.5418 [0.3260; 0.9006]  0.0181  1.9745  1.4052
##                              I^2
## Omitting Du 2019           78.9%
## Omitting Zhang 2021        75.7%
## Omitting Lechevallier 2003   75.7%
## Omitting Wu 2022           78.2%
## Omitting Zhai 2023         35.4%
## Omitting Zhai 2023         80.0%
## Omitting AlSmadi 2019      78.8%
##
## Pooled estimate            75.7%
##
## Details on meta-analytical method:
## - Mantel-Haenszel method
## - Restricted maximum-likelihood estimator for tau^2
```

8.5 VAS

8.5.1 Meta-analysis

```
vas_metacont <- metacont(data = semirigid_only,
                             mean.c = vas_score_mean_control,
                             sd.c = vas_score_sd_control,
                             mean.e = vas_score_mean_suction,
                             sd.e = vas_score_sd_suction,
                             n.e = sample_size_suction,
                             n.c = sample_size_control,
                       studlab = author_year)
vas_metacont
```

```
## Number of studies: k = 0
## Number of observations: o = 754
##
##                      MD 95%-CI  z p-value
## Common effect model  NA        --      --
## Random effects model NA        --      --
##
## Quantifying heterogeneity:
##  tau^2 = NA; tau = NA; I^2 = NA; H = NA
##
## Details on meta-analytical method:
## - Inverse variance method
## - Restricted maximum-likelihood estimator for tau^2
```

8.5.2 Forest plot

```r
forest(vas_metacont,
       sortvar = TE)
```

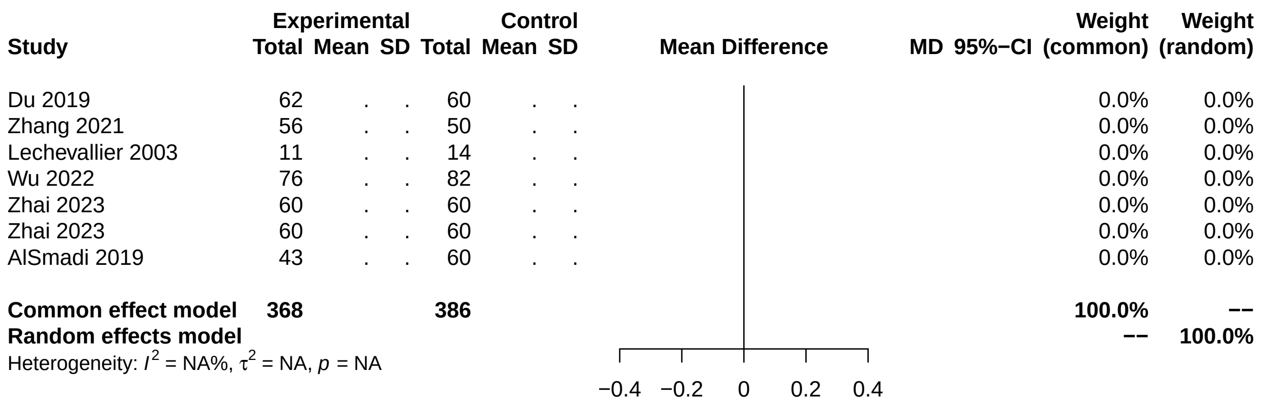

8.5.3 Trim and Fill

```r
#trimfill(vas_metacont)
#forest(trimfill(vas_metacont))
```

8.5.4 Funnel plot

```
#funnel((vas_metacont))
```

8.5.5 Baujat

```
#baujat(vas_metacont, pos = 1)
```

8.5.6 Leave one out

```
metainf(vas_metacont)
```

```
## Influential analysis (common effect model)
##
##                              MD 95%-CI p-value tau^2 tau I^2
## Omitting Du 2019
## Omitting Zhang 2021
## Omitting Lechevallier 2003
## Omitting Wu 2022
## Omitting Zhai 2023
## Omitting Zhai 2023
## Omitting AlSmadi 2019
##
## Pooled estimate
##
## Details on meta-analytical method:
## - Inverse variance method
## - Restricted maximum-likelihood estimator for tau^2
```

8.6 LoS

8.6.1 Meta-analysis

```
los_metacont <- metacont(data = semirigid_only,
                             mean.c = los_days_mean_control,
                             sd.c = los_days_sd_control,
                             mean.e = los_days_mean_suction,
                             sd.e = los_days_sd_suction,
                             n.e = sample_size_suction,
                             n.c = sample_size_control,
                       studlab = author_year)
los_metacont
```

```
## Number of studies: k = 4
## Number of observations: o = 754
##
##                             MD             95%-CI     z  p-value
## Common effect model  -0.3002 [-0.3902; -0.2102] -6.54 < 0.0001
## Random effects model -0.2916 [-0.5517; -0.0316] -2.20   0.0280
##
## Quantifying heterogeneity:
##  tau^2 = 0.0406 [0.0000; 1.2883]; tau = 0.2015 [0.0000; 1.1350]
##  I^2 = 54.6% [0.0%; 85.0%]; H = 1.48 [1.00; 2.58]
##
## Test of heterogeneity:
##     Q d.f. p-value
##  6.61    3  0.0854
##
## Details on meta-analytical method:
## - Inverse variance method
## - Restricted maximum-likelihood estimator for tau^2
## - Q-Profile method for confidence interval of tau^2 and tau
```

8.6.2 Forest plot

```r
forest(los_metacont,
       sortvar = TE)
```

Study	Experimental Total	Experimental Mean	Experimental SD	Control Total	Control Mean	Control SD	MD	95%−CI	Weight (common)	Weight (random)
Zhai 2023	60	5.30	1.1000	60	6.00	1.1000	−0.70	[−1.09; −0.31]	5.2%	21.8%
AlSmadi 2019	43	1.70	0.2000	60	2.00	0.3000	−0.30	[−0.40; −0.20]	86.8%	40.9%
Du 2019	62	4.40	1.2000	60	4.50	1.6000	−0.10	[−0.60; 0.40]	3.2%	16.5%
Zhai 2023	60	6.00	1.2000	60	6.00	1.1000	0.00	[−0.41; 0.41]	4.8%	20.8%
Zhang 2021	56	.	.	50	.	.			0.0%	0.0%
Lechevallier 2003	11	.	.	14	.	.			0.0%	0.0%
Wu 2022	76	.	.	82	.	.			0.0%	0.0%
**Common effect model**	**368**			**386**			**−0.30**	**[−0.39; −0.21]**	**100.0%**	**−−**
**Random effects model**							**−0.29**	**[−0.55; −0.03]**	**−−**	**100.0%**

Heterogeneity: I^2 = 55%, τ^2 = 0.0406, p = 0.09

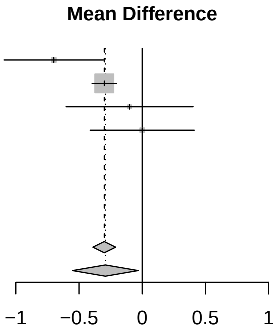

8.6.3 Trim and Fill

```r
trimfill(los_metacont)
```

```
## Warning in trimfill.meta(los_metacont): 3 observation(s) dropped due to missing
## values
```

```
## Number of studies: k = 5 (with 1 added studies)
## Number of observations: o = 585
##
##                           MD             95%-CI     z p-value
## Random effects model -0.3500 [-0.5882; -0.1118] -2.88  0.0040
##
## Quantifying heterogeneity:
##  tau^2 = 0.0414 [0.0000; 0.7562]; tau = 0.2035 [0.0000; 0.8696]
##  I^2 = 55.4% [0.0%; 83.5%]; H = 1.50 [1.00; 2.46]
##
## Test of heterogeneity:
##     Q d.f. p-value
##  8.97    4  0.0619
##
## Details on meta-analytical method:
## - Inverse variance method
## - Restricted maximum-likelihood estimator for tau^2
## - Q-Profile method for confidence interval of tau^2 and tau
## - Trim-and-fill method to adjust for funnel plot asymmetry (L-estimator)
```

```r
forest(trimfill(los_metacont),
       sortvar = TE)
```

```
## Warning in trimfill.meta(los_metacont): 3 observation(s) dropped due to missing
## values
```

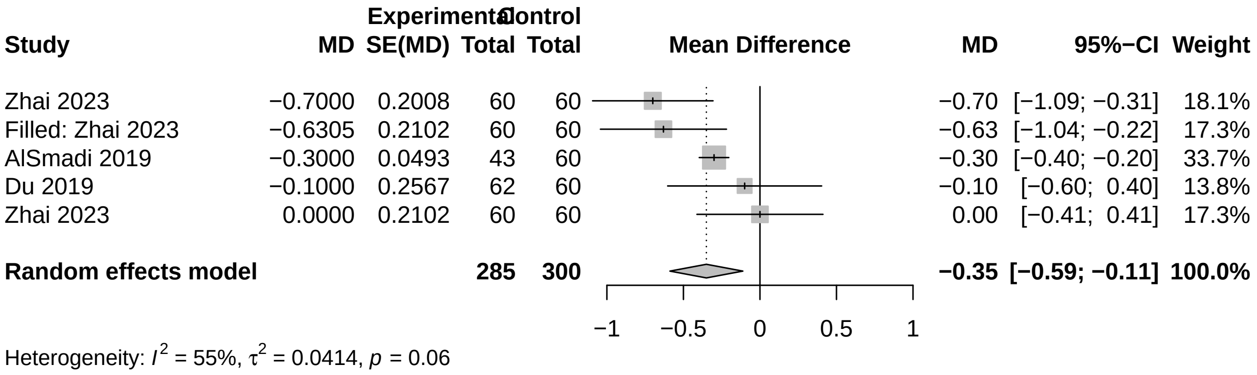

8.6.4 Funnel plot

```
funnel(trimfill(los_metacont))
```

```
## Warning in trimfill.meta(los_metacont): 3 observation(s) dropped due to missing
## values
```

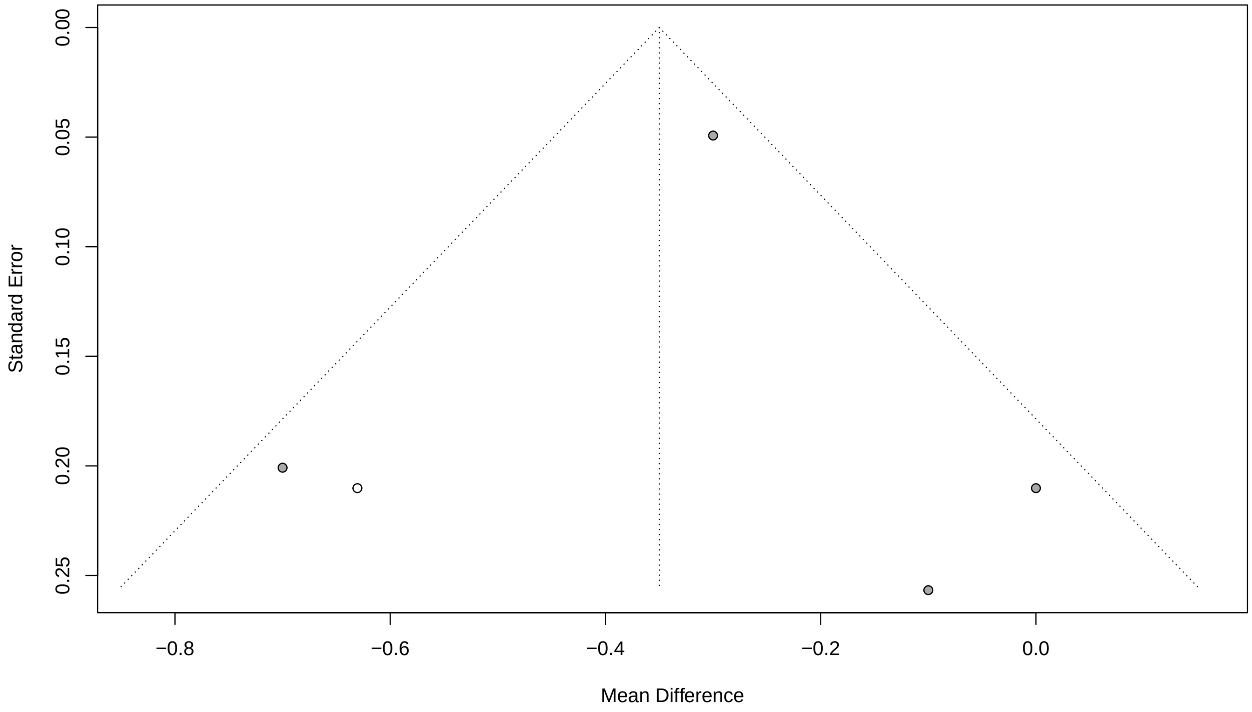

8.6.5 Baujat

```
baujat(los_metacont, pos = 1)
```

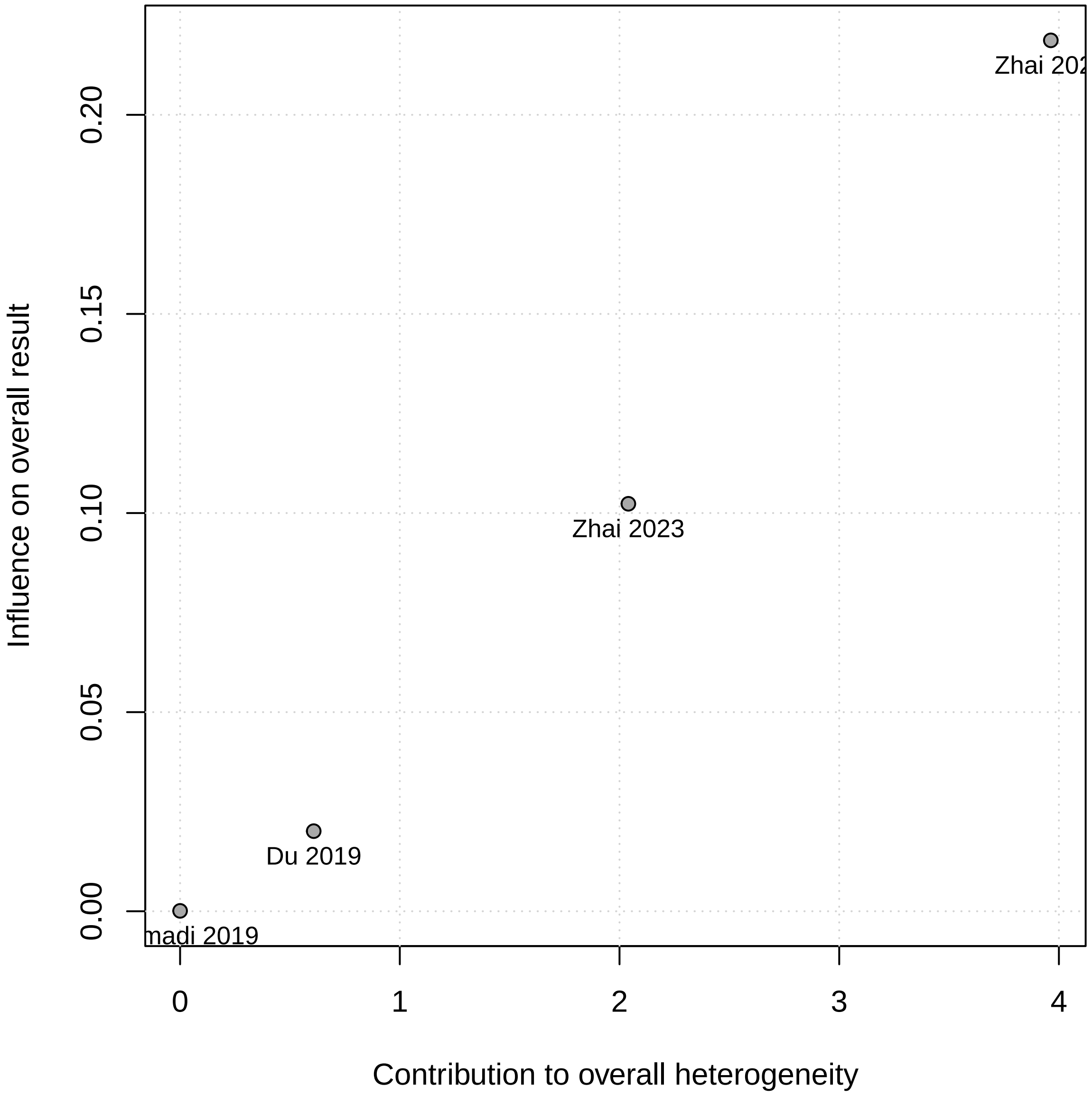

8.6.6 Leave one out

```
metainf(los_metacont)
```

```
## Influential analysis (common effect model)
##
##                                  MD             95%-CI  p-value   tau^2
## Omitting Du 2019             -0.3068 [-0.3983; -0.2153] < 0.0001  0.0648
## Omitting Zhang 2021          -0.3002 [-0.3902; -0.2102] < 0.0001  0.0406
## Omitting Lechevallier 2003   -0.3002 [-0.3902; -0.2102] < 0.0001  0.0406
## Omitting Wu 2022             -0.3002 [-0.3902; -0.2102] < 0.0001  0.0406
## Omitting Zhai 2023           -0.3152 [-0.4075; -0.2230] < 0.0001  0.0340
## Omitting Zhai 2023           -0.2781 [-0.3706; -0.1857] < 0.0001  0.0125
## Omitting AlSmadi 2019        -0.3014 [-0.5491; -0.0537]   0.0171  0.1057
##
## Pooled estimate              -0.3002 [-0.3902; -0.2102] < 0.0001  0.0406
##                                 tau    I^2
## Omitting Du 2019              0.2545  66.6%
## Omitting Zhang 2021           0.2015  54.6%
## Omitting Lechevallier 2003    0.2015  54.6%
## Omitting Wu 2022              0.2015  54.6%
## Omitting Zhai 2023            0.1843  55.2%
## Omitting Zhai 2023            0.1118  17.7%
## Omitting AlSmadi 2019         0.3251  69.8%
##
## Pooled estimate               0.2015  54.6%
##
## Details on meta-analytical method:
## - Inverse variance method
## - Restricted maximum-likelihood estimator for tau^2
```

8.7 Complications - All

8.7.1 Meta-analysis

```
comp_metabin <- metabin(data = semirigid_only,
                          event.c = complications_postop_overall_n_control,
                        n.c = sample_size_control,
                        event.e = complications_postop_overall_n_suction,
                        n.e = sample_size_suction,
                        studlab = author_year)
comp_metabin
```

```
## Number of studies: k = 6
## Number of observations: o = 754
## Number of events: e = 54
##
##                           RR           95%-CI     z p-value
## Common effect model  0.3941 [0.2208; 0.7033] -3.15  0.0016
## Random effects model 0.4512 [0.2462; 0.8269] -2.58  0.0100
##
## Quantifying heterogeneity:
##  tau^2 = 0 [0.0000; 2.9976]; tau = 0 [0.0000; 1.7314]
##  I^2 = 0.0% [0.0%; 74.6%]; H = 1.00 [1.00; 1.99]
##
## Test of heterogeneity:
##     Q d.f. p-value
##  4.47    5  0.4836
##
## Details on meta-analytical method:
## - Mantel-Haenszel method
## - Inverse variance method
## - Restricted maximum-likelihood estimator for tau^2
## - Q-Profile method for confidence interval of tau^2 and tau
```

8.7.2 Forest plot

```r
forest(comp_metabin,
       sortvar = TE)
```

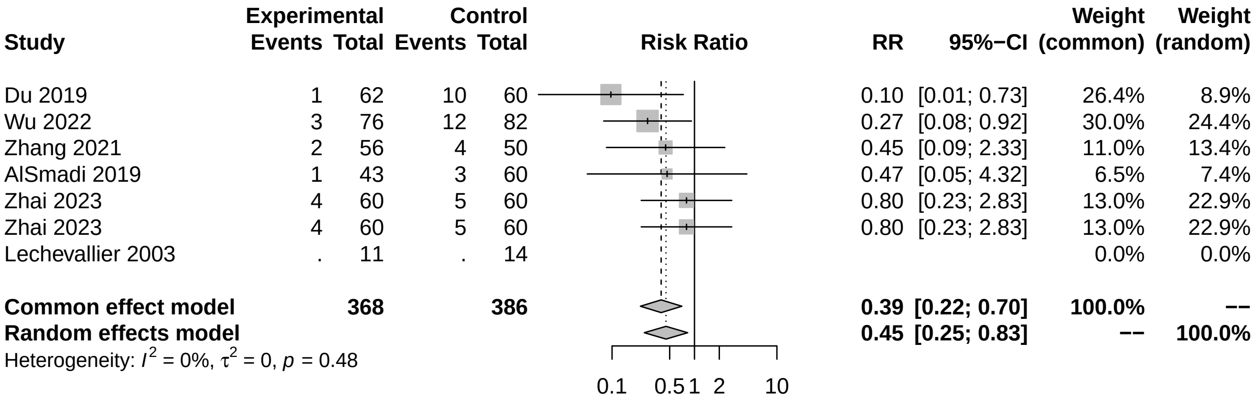

8.7.3 Trim and Fill

```r
trimfill(comp_metabin)
```

```
## Warning in trimfill.meta(comp_metabin): 1 observation(s) dropped due to missing
## values
```

```
## Number of studies: k = 6 (with 0 added studies)
## Number of observations: o = 729
## Number of events: e = 54
##
##                          RR           95%-CI     z p-value
## Random effects model 0.4512 [0.2462; 0.8269] -2.58  0.0100
##
## Quantifying heterogeneity:
##  tau^2 = 0 [0.0000; 2.9976]; tau = 0 [0.0000; 1.7314]
##  I^2 = 0.0% [0.0%; 74.6%]; H = 1.00 [1.00; 1.99]
##
## Test of heterogeneity:
##     Q d.f. p-value
##  4.47    5  0.4836
##
## Details on meta-analytical method:
## - Inverse variance method
## - Restricted maximum-likelihood estimator for tau^2
## - Q-Profile method for confidence interval of tau^2 and tau
## - Trim-and-fill method to adjust for funnel plot asymmetry (L-estimator)
```

```r
forest(trimfill(comp_metabin),
       sortvar = TE)
```

```
## Warning in trimfill.meta(comp_metabin): 1 observation(s) dropped due to missing
## values
```

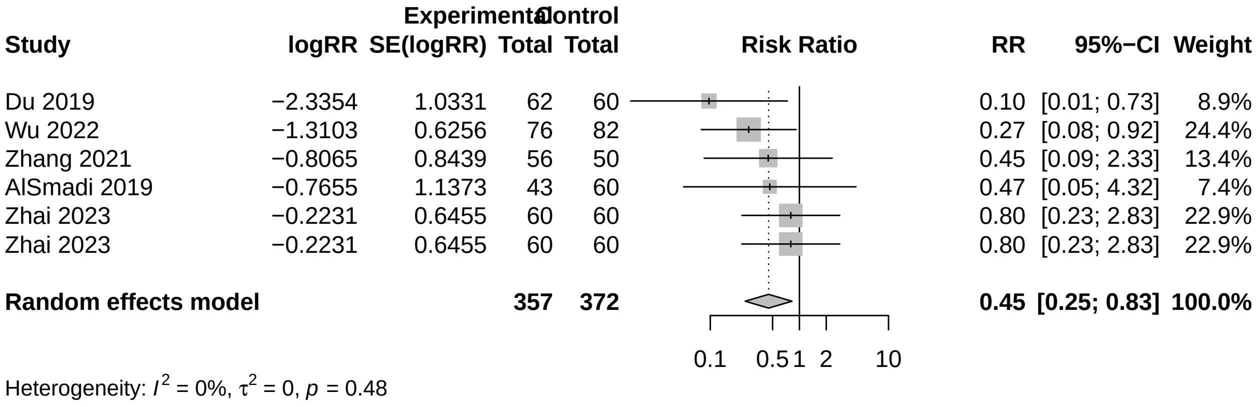

8.7.4 Funnel plot

```
funnel(trimfill(comp_metabin))
```

```
## Warning in trimfill.meta(comp_metabin): 1 observation(s) dropped due to missing
## values
```

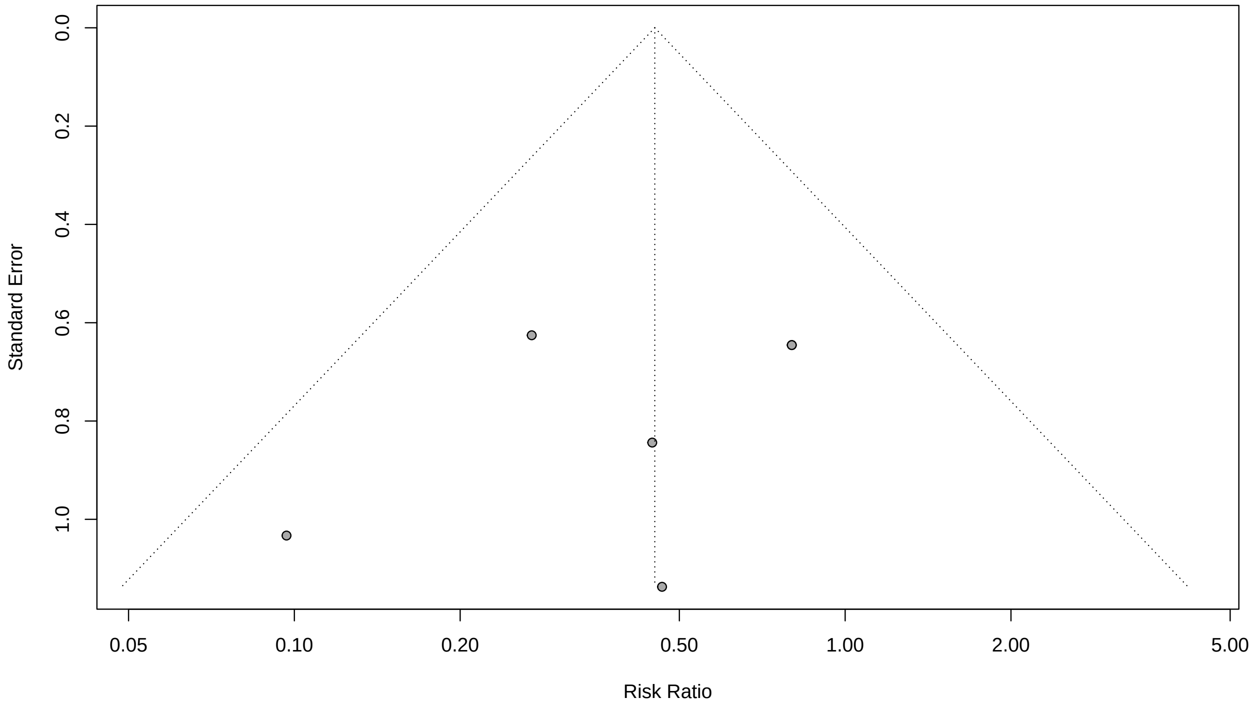

8.7.5 Baujat

```
baujat(comp_metabin, pos = 1)
```

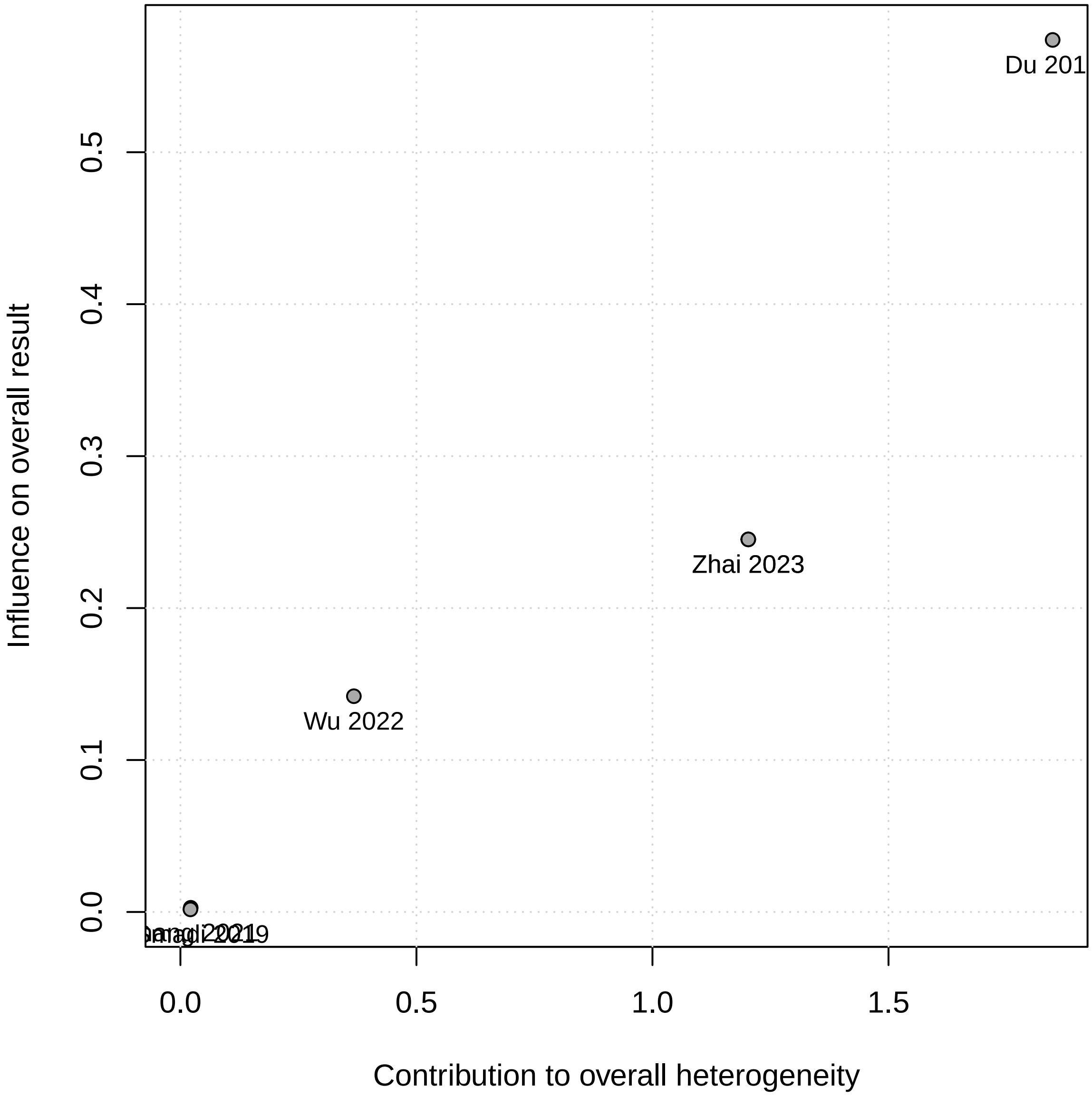

8.7.6 Leave one out

```
metainf(comp_metabin)
```

```
## Influential analysis (common effect model)
##
##                                RR            95%-CI p-value   tau^2     tau
## Omitting Du 2019           0.5010 [0.2693; 0.9321]  0.0291  0.0000  0.0000
## Omitting Zhang 2021        0.3876 [0.2089; 0.7195]  0.0027  0.0516  0.2271
## Omitting Lechevallier 2003   0.3941 [0.2208; 0.7033]  0.0016  0.0000  0.0000
## Omitting Wu 2022           0.4475 [0.2311; 0.8665]  0.0171  0.0000  0.0000
## Omitting Zhai 2023         0.3334 [0.1720; 0.6463]  0.0011  0.0000  0.0000
## Omitting Zhai 2023         0.3334 [0.1720; 0.6463]  0.0011  0.0000  0.0000
## Omitting AlSmadi 2019      0.3892 [0.2136; 0.7090]  0.0020  0.0039  0.0626
##
## Pooled estimate            0.3941 [0.2208; 0.7033]  0.0016  0.0000  0.0000
##                             I^2
## Omitting Du 2019           0.0%
## Omitting Zhang 2021       10.6%
## Omitting Lechevallier 2003   0.0%
## Omitting Wu 2022           0.0%
## Omitting Zhai 2023         0.0%
## Omitting Zhai 2023         0.0%
## Omitting AlSmadi 2019     10.5%
##
## Pooled estimate            0.0%
##
## Details on meta-analytical method:
## - Mantel-Haenszel method
## - Restricted maximum-likelihood estimator for tau^2
```

8.8 Fever

8.8.1 Meta-analysis

```
fever_metabin <- metabin(data = semirigid_only,
                         event.c = fever_n_control,
                       n.c = sample_size_control,
                       event.e = fever_n_suction,
                       n.e = sample_size_suction,
                       studlab = author_year)
fever_metabin
```

```
## Number of studies: k = 3
## Number of observations: o = 754
## Number of events: e = 28
##
##                            RR           95%-CI     z p-value
## Common effect model  0.2741 [0.1132; 0.6638] -2.87  0.0041
## Random effects model 0.2932 [0.1189; 0.7231] -2.66  0.0077
##
## Quantifying heterogeneity:
##  tau^2 = 0 [0.0000; 20.1531]; tau = 0 [0.0000; 4.4892]
##  I^2 = 0.0% [0.0%; 89.6%]; H = 1.00 [1.00; 3.10]
##
## Test of heterogeneity:
##     Q d.f. p-value
##  1.16    2  0.5607
##
## Details on meta-analytical method:
## - Mantel-Haenszel method
## - Inverse variance method
## - Restricted maximum-likelihood estimator for tau^2
## - Q-Profile method for confidence interval of tau^2 and tau
```

8.8.2 Forest plot

```r
forest(fever_metabin,
       sortvar = TE)
```

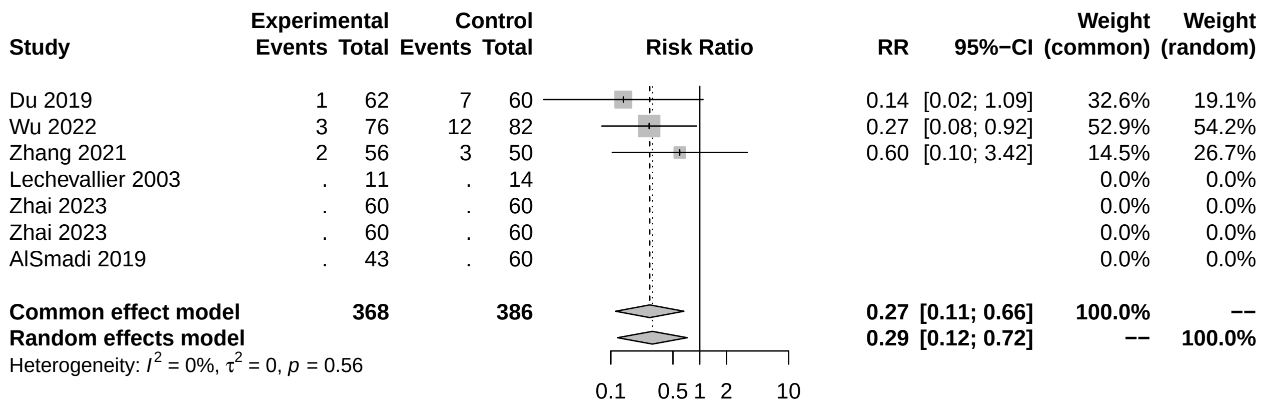

8.8.3 Trim and Fill

```r
trimfill(fever_metabin)
```

```
## Warning in trimfill.meta(fever_metabin): 4 observation(s) dropped due to
## missing values
```

```
## Number of studies: k = 4 (with 1 added studies)
## Number of observations: o = 508
## Number of events: e = 36
##
##                            RR           95%-CI     z p-value
## Random effects model 0.3502 [0.1531; 0.8008] -2.49  0.0129
##
## Quantifying heterogeneity:
##  tau^2 = 0 [0.0000; 8.5047]; tau = 0 [0.0000; 2.9163]
##  I^2 = 0.0% [0.0%; 84.7%]; H = 1.00 [1.00; 2.56]
##
## Test of heterogeneity:
##     Q d.f. p-value
##  2.08    3  0.5551
##
## Details on meta-analytical method:
## - Inverse variance method
## - Restricted maximum-likelihood estimator for tau^2
## - Q-Profile method for confidence interval of tau^2 and tau
## - Trim-and-fill method to adjust for funnel plot asymmetry (L-estimator)
```

```r
forest(trimfill(fever_metabin),
       sortvar = TE)
```

```
## Warning in trimfill.meta(fever_metabin): 4 observation(s) dropped due to
## missing values
```

Study	logRR	SE(logRR)	Experimental Total	Control Total	Risk Ratio	RR	95%−CI	Weight
Du 2019	−1.9787	1.0536	62	60		0.14	[0.02; 1.09]	16.0%
Wu 2022	−1.3103	0.6256	76	82		0.27	[0.08; 0.92]	45.5%
Zhang 2021	−0.5188	0.8919	56	50		0.60	[0.10; 3.42]	22.4%
Filled: Du 2019	−0.1200	1.0536	62	60		0.89	[0.11; 6.99]	16.0%
**Random effects model**			**256**	**252**		**0.35**	**[0.15; 0.80]**	**100.0%**

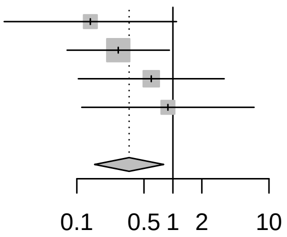

Heterogeneity: $I^2 = 0\%$, $\tau^2 = 0$, $p = 0.56$

8.8.4 Funnel plot

```
funnel(trimfill(fever_metabin))
```

```
## Warning in trimfill.meta(fever_metabin): 4 observation(s) dropped due to
## missing values
```

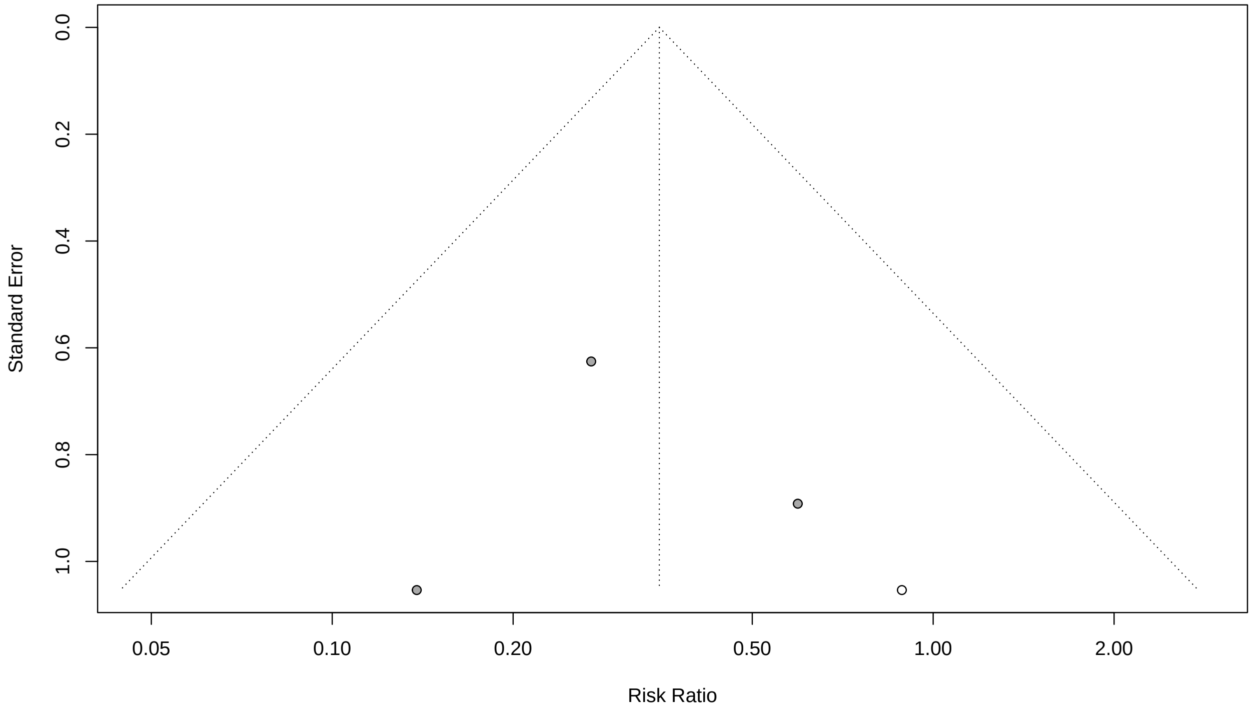

8.8.5 Baujat

```
baujat(fever_metabin, pos = 1)
```

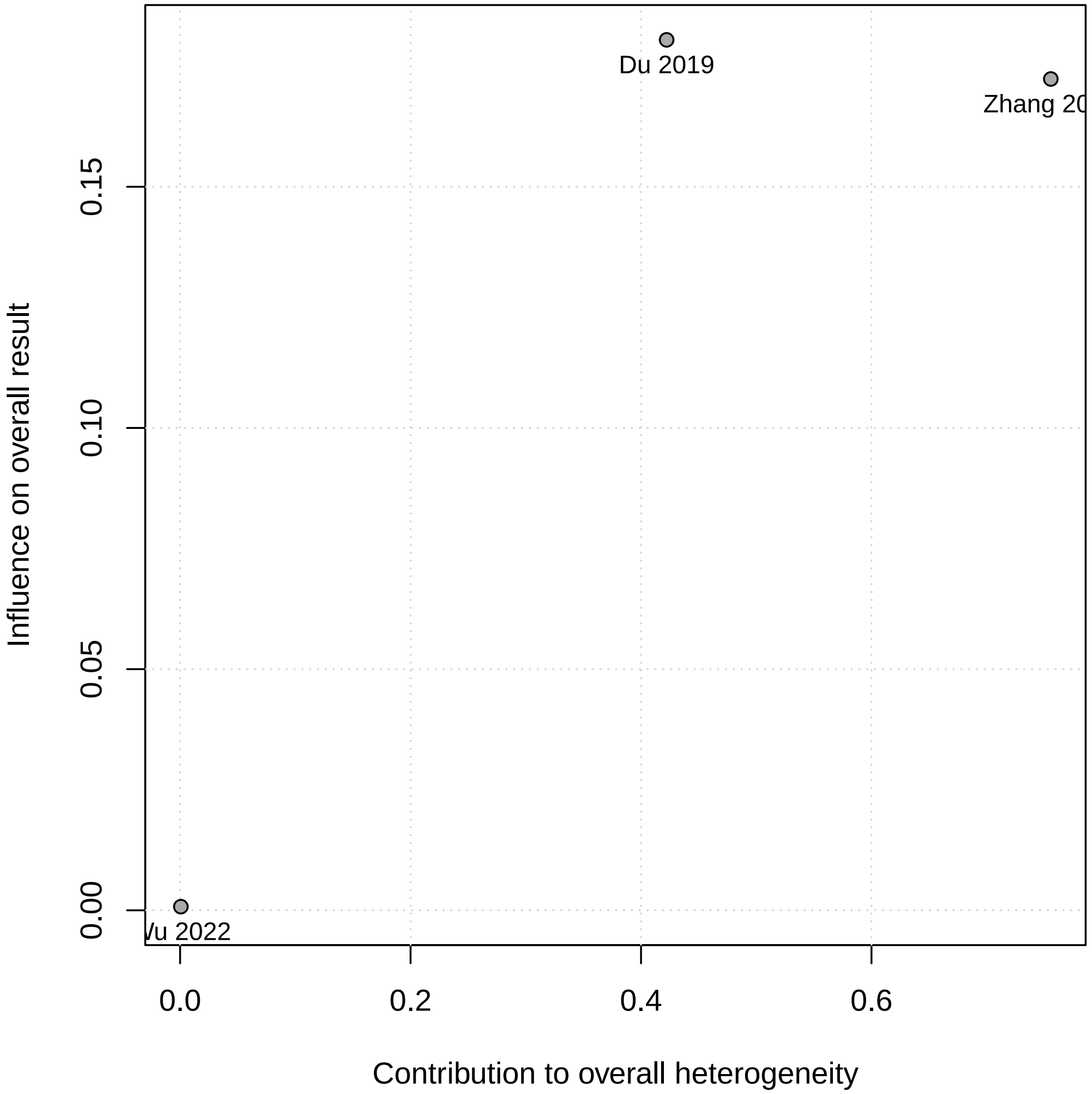

8.8.6 Leave one out

```
metainf(fever_metabin)
```

```
## Influential analysis (common effect model)
##
##                                 RR           95%-CI p-value   tau^2     tau
## Omitting Du 2019            0.3399 [0.1261; 0.9159]  0.0329  0.0000  0.0000
## Omitting Zhang 2021         0.2196 [0.0770; 0.6259]  0.0046  0.0000  0.0000
## Omitting Lechevallier 2003   0.2741 [0.1132; 0.6638]  0.0041  0.0000  0.0000
## Omitting Wu 2022            0.2791 [0.0779; 1.0005]  0.0501  0.1129  0.3360
## Omitting Zhai 2023          0.2741 [0.1132; 0.6638]  0.0041  0.0000  0.0000
## Omitting Zhai 2023          0.2741 [0.1132; 0.6638]  0.0041  0.0000  0.0000
## Omitting AlSmadi 2019       0.2741 [0.1132; 0.6638]  0.0041  0.0000  0.0000
##
## Pooled estimate             0.2741 [0.1132; 0.6638]  0.0041  0.0000  0.0000
##                                I^2
## Omitting Du 2019              0.0%
## Omitting Zhang 2021           0.0%
## Omitting Lechevallier 2003     0.0%
## Omitting Wu 2022             10.6%
## Omitting Zhai 2023            0.0%
## Omitting Zhai 2023            0.0%
## Omitting AlSmadi 2019         0.0%
##
## Pooled estimate               0.0%
##
## Details on meta-analytical method:
## - Mantel-Haenszel method
## - Restricted maximum-likelihood estimator for tau^2
```

8.9 Infections

8.9.1 Meta-analysis

```
infection_metabin <- metabin(data = semirigid_only,
                         event.c = infection_n_control,
                       n.c = sample_size_control,
                       event.e = infection_n_suction,
                       n.e = sample_size_suction,
                       studlab = author_year)
infection_metabin
```

```
## Number of studies: k = 3
## Number of observations: o = 754
## Number of events: e = 28
##
##                            RR           95%-CI     z p-value
## Common effect model  0.2741 [0.1132; 0.6638] -2.87  0.0041
## Random effects model 0.2932 [0.1189; 0.7231] -2.66  0.0077
##
## Quantifying heterogeneity:
##  tau^2 = 0 [0.0000; 20.1531]; tau = 0 [0.0000; 4.4892]
##  I^2 = 0.0% [0.0%; 89.6%]; H = 1.00 [1.00; 3.10]
##
## Test of heterogeneity:
##     Q d.f. p-value
##  1.16    2  0.5607
##
## Details on meta-analytical method:
## - Mantel-Haenszel method
## - Inverse variance method
## - Restricted maximum-likelihood estimator for tau^2
## - Q-Profile method for confidence interval of tau^2 and tau
```

8.9.2 Forest plot

```r
forest(infection_metabin,
       sortvar = TE)
```

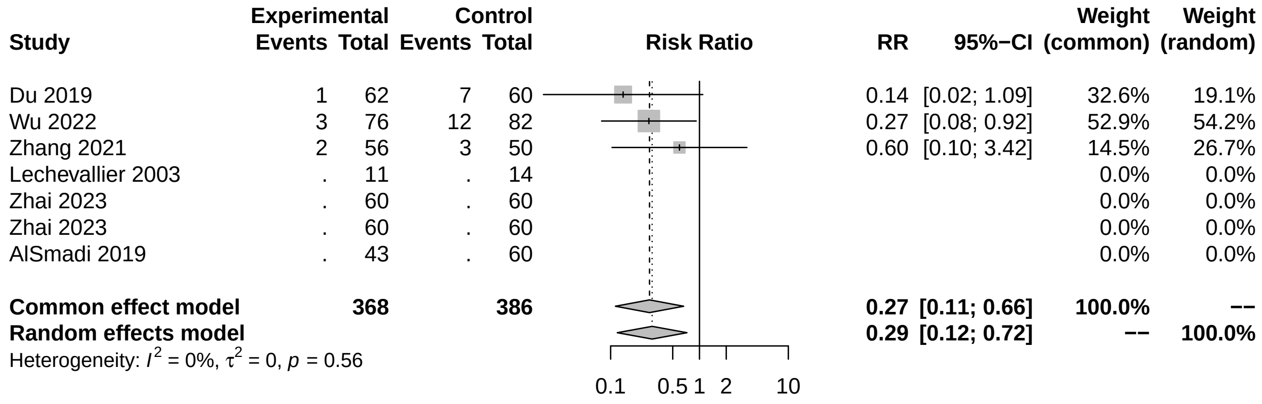

8.9.3 Trim and Fill

```r
trimfill(infection_metabin)
```

```
## Warning in trimfill.meta(infection_metabin): 4 observation(s) dropped due to
## missing values
```

```
## Number of studies: k = 4 (with 1 added studies)
## Number of observations: o = 508
## Number of events: e = 36
##
##                              RR           95%-CI     z p-value
## Random effects model 0.3502 [0.1531; 0.8008] -2.49  0.0129
##
## Quantifying heterogeneity:
##  tau^2 = 0 [0.0000; 8.5047]; tau = 0 [0.0000; 2.9163]
##  I^2 = 0.0% [0.0%; 84.7%]; H = 1.00 [1.00; 2.56]
##
## Test of heterogeneity:
##     Q d.f. p-value
##  2.08    3  0.5551
##
## Details on meta-analytical method:
## - Inverse variance method
## - Restricted maximum-likelihood estimator for tau^2
## - Q-Profile method for confidence interval of tau^2 and tau
## - Trim-and-fill method to adjust for funnel plot asymmetry (L-estimator)
```

```r
forest(trimfill(infection_metabin),
       sortvar = TE)
```

```
## Warning in trimfill.meta(infection_metabin): 4 observation(s) dropped due to
## missing values
```

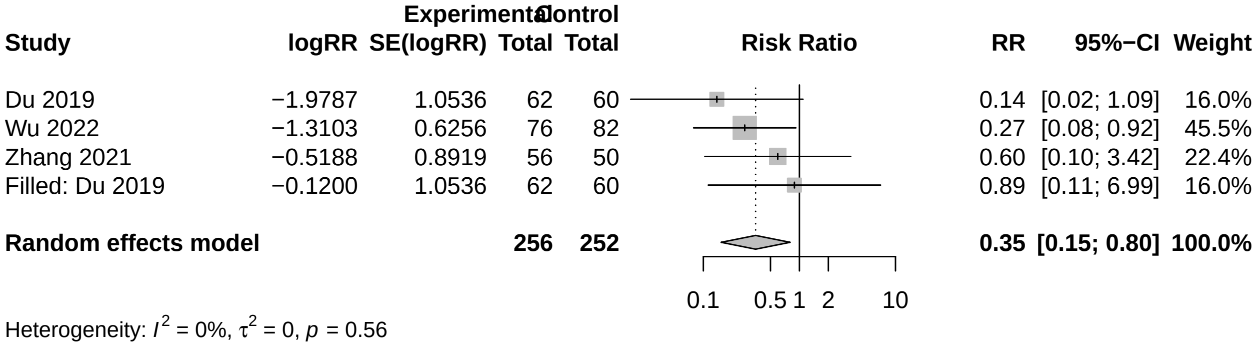

8.9.4 Funnel plot

```
funnel(trimfill(infection_metabin))
```

```
## Warning in trimfill.meta(infection_metabin): 4 observation(s) dropped due to
## missing values
```

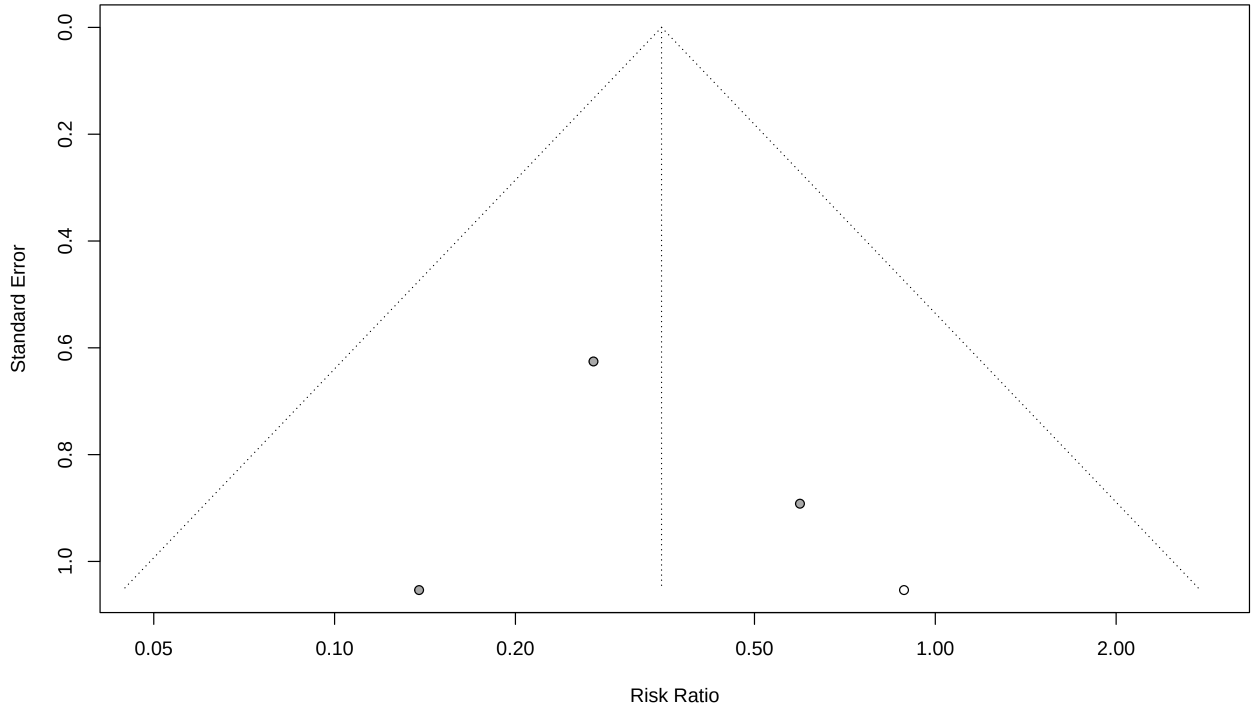

8.9.5 Baujat

```
baujat(infection_metabin, pos = 1)
```

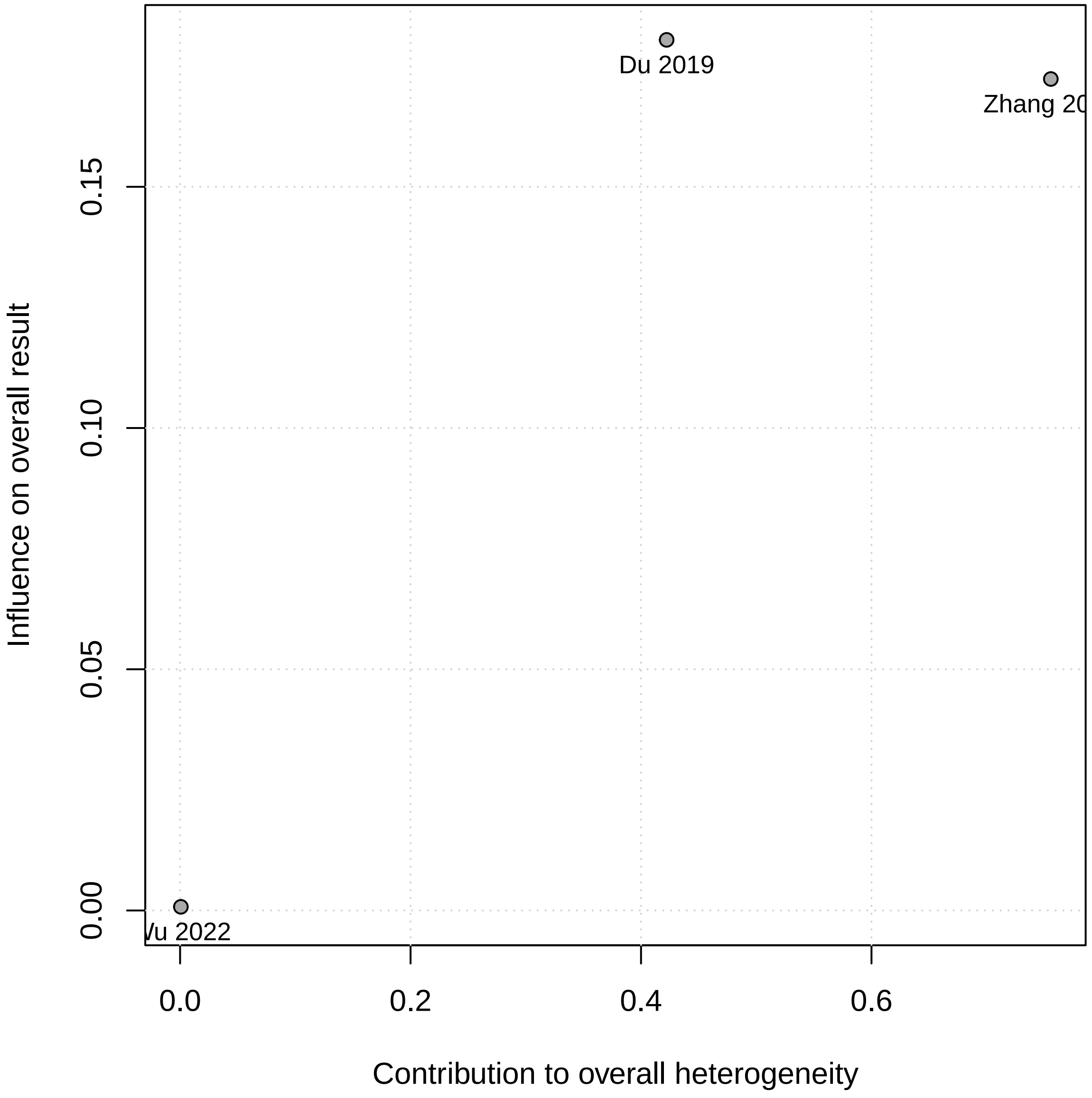

8.9.6 Leave one out

```
metainf(infection_metabin)
```

```
## Influential analysis (common effect model)
##
##                                   RR           95%-CI p-value   tau^2     tau
## Omitting Du 2019              0.3399 [0.1261; 0.9159]  0.0329  0.0000  0.0000
## Omitting Zhang 2021           0.2196 [0.0770; 0.6259]  0.0046  0.0000  0.0000
## Omitting Lechevallier 2003    0.2741 [0.1132; 0.6638]  0.0041  0.0000  0.0000
## Omitting Wu 2022              0.2791 [0.0779; 1.0005]  0.0501  0.1129  0.3360
## Omitting Zhai 2023            0.2741 [0.1132; 0.6638]  0.0041  0.0000  0.0000
## Omitting Zhai 2023            0.2741 [0.1132; 0.6638]  0.0041  0.0000  0.0000
## Omitting AlSmadi 2019         0.2741 [0.1132; 0.6638]  0.0041  0.0000  0.0000
##
## Pooled estimate               0.2741 [0.1132; 0.6638]  0.0041  0.0000  0.0000
##                                  I^2
## Omitting Du 2019                0.0%
## Omitting Zhang 2021             0.0%
## Omitting Lechevallier 2003      0.0%
## Omitting Wu 2022               10.6%
## Omitting Zhai 2023              0.0%
## Omitting Zhai 2023              0.0%
## Omitting AlSmadi 2019           0.0%
##
## Pooled estimate                 0.0%
##
## Details on meta-analytical method:
## - Mantel-Haenszel method
## - Restricted maximum-likelihood estimator for tau^2
```

8.10 Sepsis

8.10.1 Meta-analysis

```
sepsis_metabin <- metabin(data = semirigid_only,
                         event.c = sepsis_n_control,
                       n.c = sample_size_control,
                       event.e = sepsis_n_suction,
                       n.e = sample_size_suction,
                       studlab = author_year)
sepsis_metabin
```

```
## Number of studies: k = 0
## Number of observations: o = 754
## Number of events: e = 0
##
##                      RR 95%-CI  z p-value
## Common effect model  NA        --      --
## Random effects model NA        --      --
##
## Quantifying heterogeneity:
##  tau^2 = NA; tau = NA; I^2 = NA; H = NA
##
## Details on meta-analytical method:
## - Mantel-Haenszel method
## - Inverse variance method
## - Restricted maximum-likelihood estimator for tau^2
```

8.10.2 Forest plot

```r
forest(sepsis_metabin,
       sortvar = TE)
```

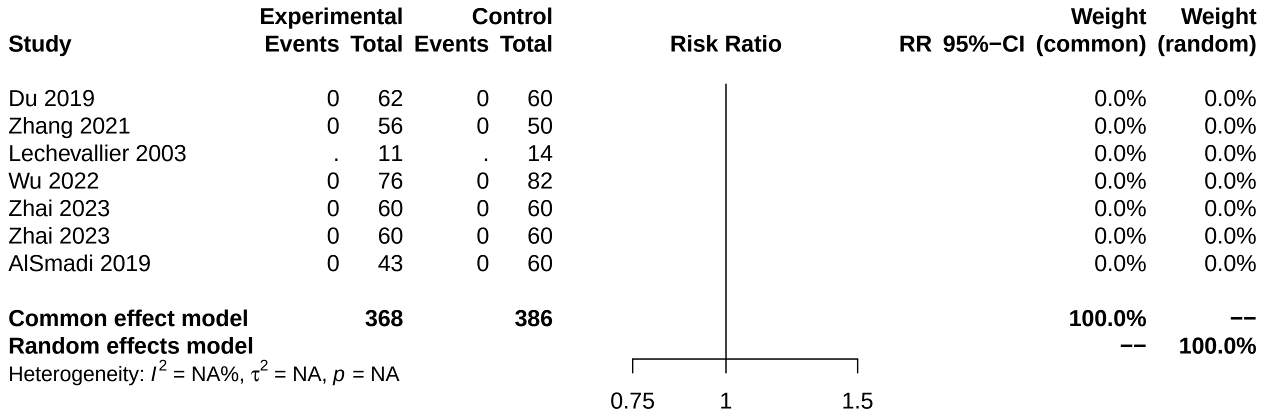

8.10.3 Trim and Fill

```
#trimfill(sepsis_metabin)
#forest(trimfill(sepsis_metabin),
#       sortvar = TE)
```

8.10.4 Funnel plot

```
#funnel((sepsis_metabin))
```

8.10.5 Baujat

```r
#baujat(sepsis_metabin, pos = 1)
```

8.10.6 Leave one out

```
metainf(sepsis_metabin)
```

```
## Influential analysis (common effect model)
##
##                              RR 95%-CI p-value tau^2 tau I^2
## Omitting Du 2019
## Omitting Zhang 2021
## Omitting Lechevallier 2003
## Omitting Wu 2022
## Omitting Zhai 2023
## Omitting Zhai 2023
## Omitting AlSmadi 2019
##
## Pooled estimate
##
## Details on meta-analytical method:
## - Mantel-Haenszel method
## - Restricted maximum-likelihood estimator for tau^2
```

8.11 Abscess

8.11.1 Meta-analysis

```
abscess_metabin <- metabin(data = semirigid_only,
                             event.c = abscess_n_control,
                           n.c = sample_size_control,
                           event.e = abscess_n_suction,
                           n.e = sample_size_suction,
                           studlab = author_year)
abscess_metabin
```

```
## Number of studies: k = 0
## Number of observations: o = 754
## Number of events: e = 0
##
##                        RR 95%-CI  z p-value
## Common effect model  NA        --      --
## Random effects model NA        --      --
##
## Quantifying heterogeneity:
##  tau^2 = NA; tau = NA; I^2 = NA; H = NA
##
## Details on meta-analytical method:
## - Mantel-Haenszel method
## - Inverse variance method
## - Restricted maximum-likelihood estimator for tau^2
```

8.11.2 Forest plot

```r
forest(abscess_metabin,
       sortvar = TE)
```

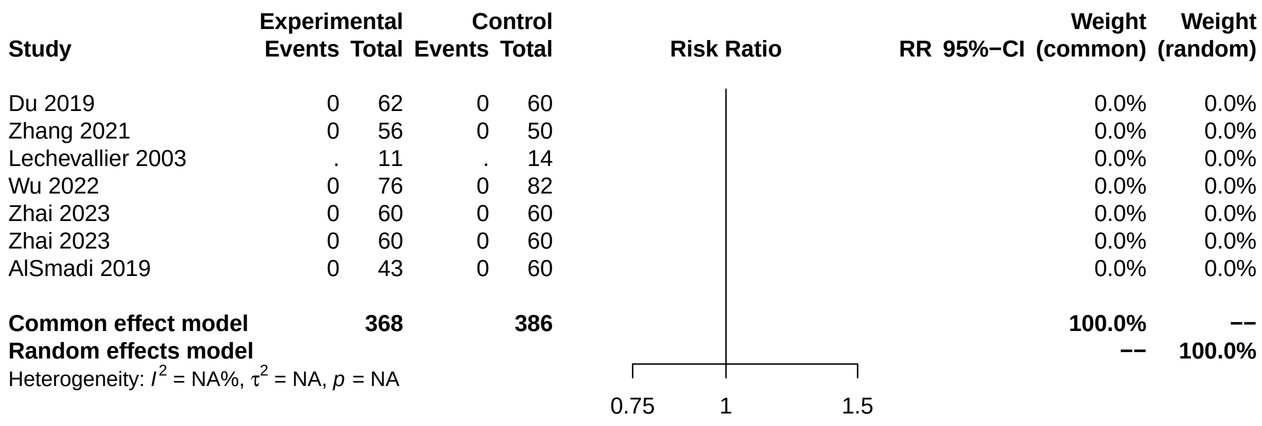

8.11.3 Trim and Fill

```
#trimfill(abscess_metabin)
#forest(trimfill(abscess_metabin),
#       sortvar = TE)
```

8.11.4 Funnel plot

```r
#funnel((abscess_metabin))
```

8.11.5 Baujat

```
#baujat(abscess_metabin, pos = 1)
```

8.11.6 Leave one out

```
#metainf(abscess_metabin)
```

8.12 Haematoma

8.12.1 Meta-analysis

```
haematoma_metabin <- metabin(data = semirigid_only,
                          event.c = hematoma_n_control,
                        n.c = sample_size_control,
                        event.e = hematoma_n_suction,
                        n.e = sample_size_suction,
                        studlab = author_year)
haematoma_metabin
```

```
## Number of studies: k = 0
## Number of observations: o = 754
## Number of events: e = 0
##
##                       RR 95%-CI  z p-value
## Common effect model   NA        --      --
## Random effects model NA        --      --
##
## Quantifying heterogeneity:
##  tau^2 = NA; tau = NA; I^2 = NA; H = NA
##
## Details on meta-analytical method:
## - Mantel-Haenszel method
## - Inverse variance method
## - Restricted maximum-likelihood estimator for tau^2
```

8.12.2 Forest plot

```r
forest(haematoma_metabin,
       sortvar = TE)
```

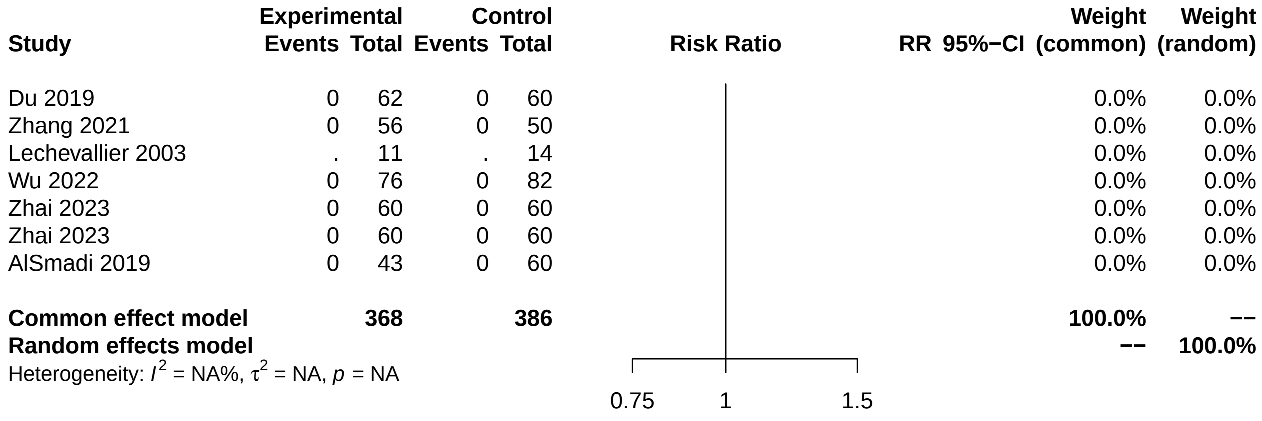

8.12.3 Trim and Fill

```
#trimfill(haematoma_metabin)
#forest(trimfill(haematoma_metabin),
#        sortvar = TE)
```

8.12.4 Funnel plot

```
#funnel((haematoma_metabin))
```

8.12.5 Baujat

```
#baujat(haematoma_metabin, pos = 1)
```

8.12.6 Leave one out

```
#metainf(haematoma_metabin)
```

8.13 Pain

8.13.1 Meta-analysis

```
pain_metabin <- metabin(data = semirigid_only,
                          event.c = pain_n_control,
                        n.c = sample_size_control,
                        event.e = pain_n_suction,
                        n.e = sample_size_suction,
                        studlab = author_year)
pain_metabin
```

```
## Number of studies: k = 1
## Number of observations: o = 754
## Number of events: e = 1
##
##                             RR           95%-CI     z p-value
## Common effect model  0.2982 [0.0124; 7.1592] -0.75  0.4556
## Random effects model 0.2979 [0.0124; 7.1502] -0.75  0.4552
##
## Quantifying heterogeneity:
##  tau^2 = NA; tau = NA; I^2 = NA; H = NA
##
## Details on meta-analytical method:
## - Mantel-Haenszel method
## - Inverse variance method
## - Restricted maximum-likelihood estimator for tau^2
## - Continuity correction of 0.5 in studies with zero cell frequencies
```

8.13.2 Forest plot

```r
forest(pain_metabin,
       sortvar = TE)
```

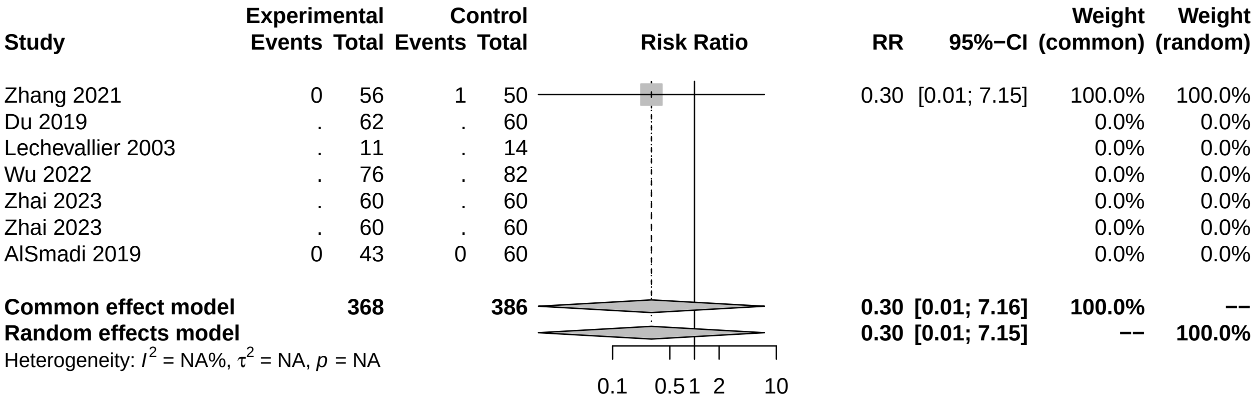

8.13.3 Trim and Fill

```
#trimfill(pain_metabin)
#forest(trimfill(pain_metabin),
#       sortvar = TE)
```

8.13.4 Funnel plot

```
funnel((pain_metabin))
```

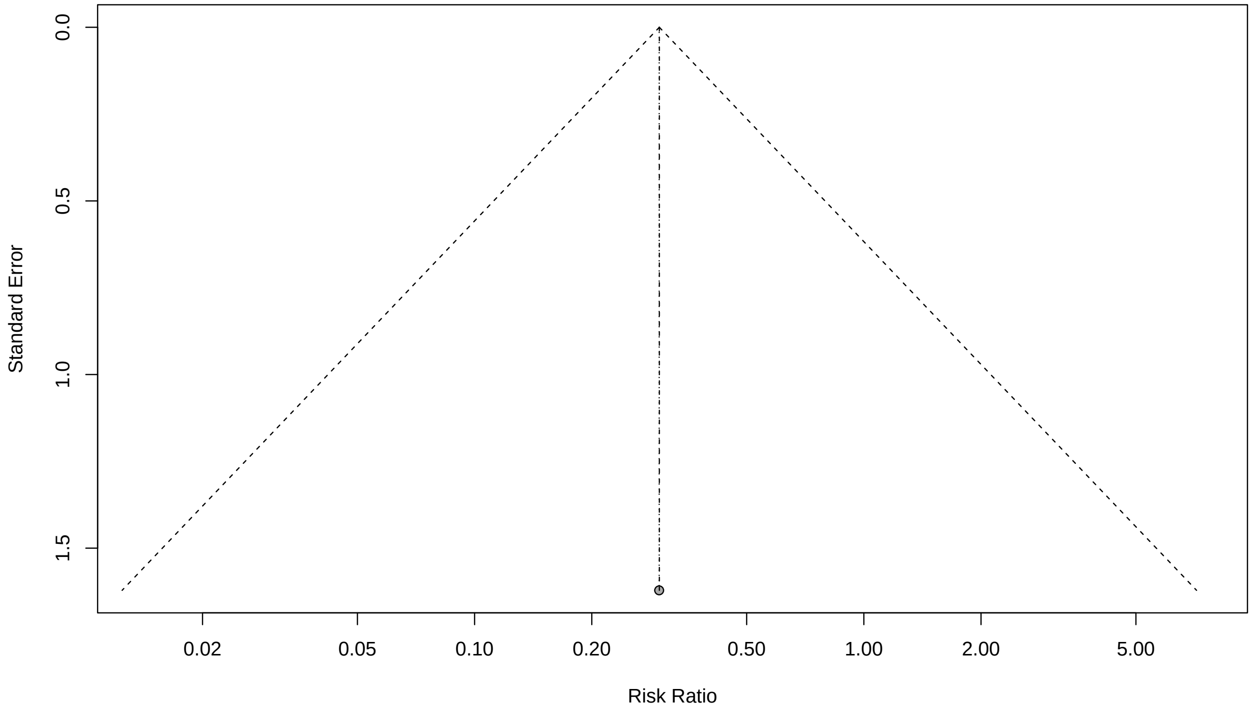

8.13.5 Baujat

```
baujat(pain_metabin, pos = 1)
```

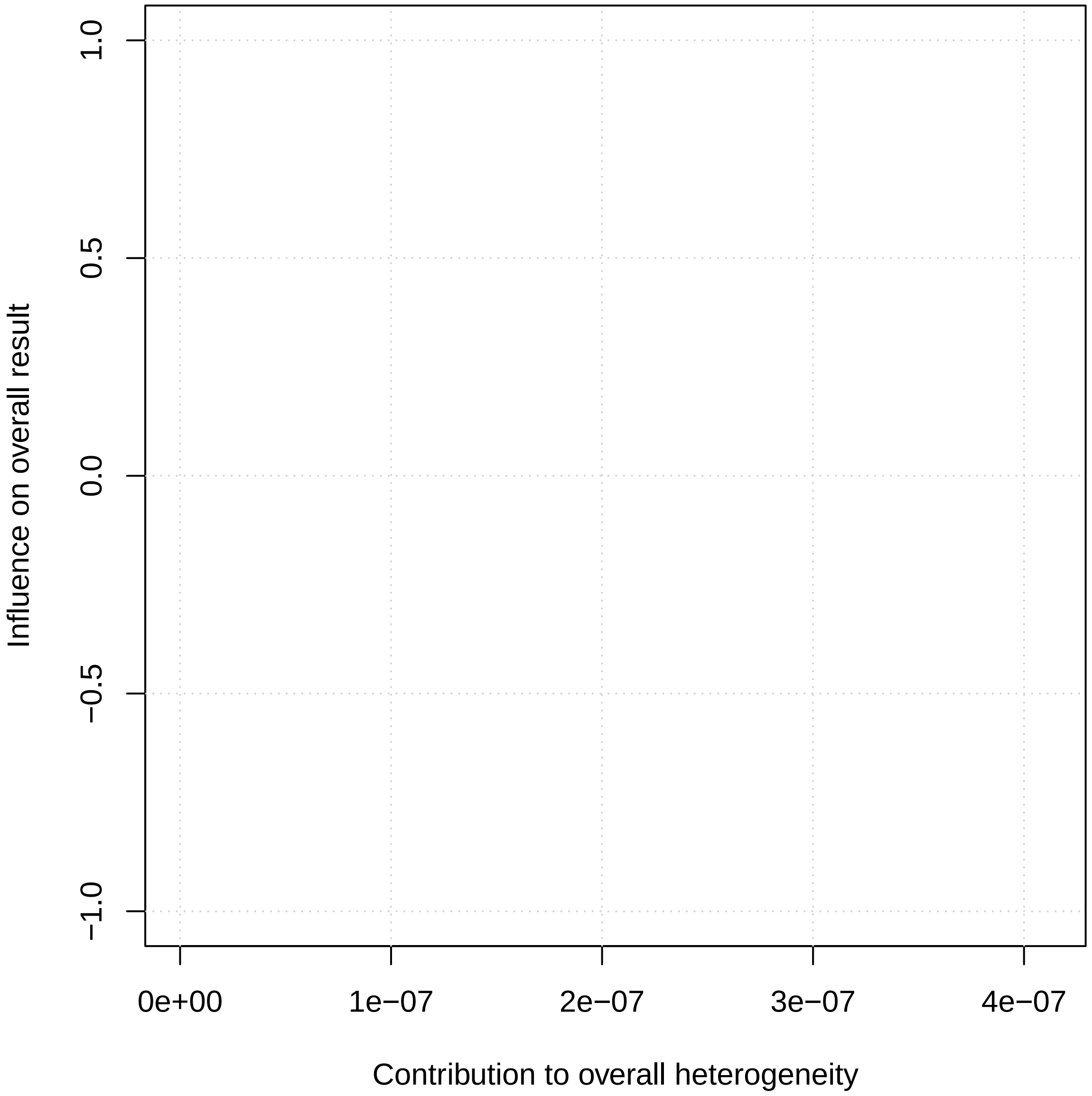

8.13.6 Leave one out

```r
metainf(pain_metabin, pos = 1)
```

```
## Influential analysis (common effect model)
##
##                                    RR           95%-CI p-value tau^2 tau I^2
## Omitting Du 2019             0.2982 [0.0124; 7.1592]  0.4556
## Omitting Zhang 2021
## Omitting Lechevallier 2003   0.2982 [0.0124; 7.1592]  0.4556
## Omitting Wu 2022             0.2982 [0.0124; 7.1592]  0.4556
## Omitting Zhai 2023           0.2982 [0.0124; 7.1592]  0.4556
## Omitting Zhai 2023           0.2982 [0.0124; 7.1592]  0.4556
## Omitting AlSmadi 2019        0.2982 [0.0124; 7.1592]  0.4556
##
## Pooled estimate              0.2982 [0.0124; 7.1592]  0.4556
##
## Details on meta-analytical method:
## - Mantel-Haenszel method
## - Restricted maximum-likelihood estimator for tau^2
```

8.14 Stricture

8.14.1 Meta-analysis

```
stricture_metabin <- metabin(data = semirigid_only,
                         event.c = stricture_n_control,
                       n.c = sample_size_control,
                       event.e = stricture_n_suction,
                       n.e = sample_size_suction,
                       studlab = author_year)
stricture_metabin
```

```
## Number of studies: k = 1
## Number of observations: o = 754
## Number of events: e = 3
##
##                              RR           95%-CI     z p-value
## Common effect model  0.1383 [0.0073; 2.6222] -1.32  0.1876
## Random effects model 0.1383 [0.0073; 2.6211] -1.32  0.1875
##
## Quantifying heterogeneity:
##  tau^2 = NA; tau = NA; I^2 = NA; H = NA
##
## Details on meta-analytical method:
## - Mantel-Haenszel method
## - Inverse variance method
## - Restricted maximum-likelihood estimator for tau^2
## - Continuity correction of 0.5 in studies with zero cell frequencies
```

8.14.2 Forest plot

```r
forest(stricture_metabin,
       sortvar = TE)
```

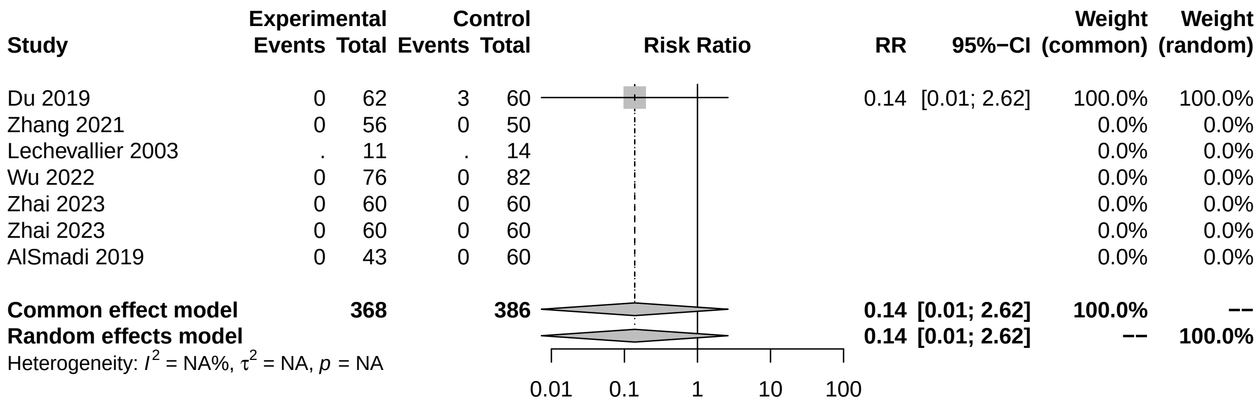

8.14.3 Trim and Fill

```
#trimfill(stricture_metabin)
#forest(trimfill(stricture_metabin),
#       sortvar = TE)
```

8.14.4 Funnel plot

```
funnel((stricture_metabin))
```

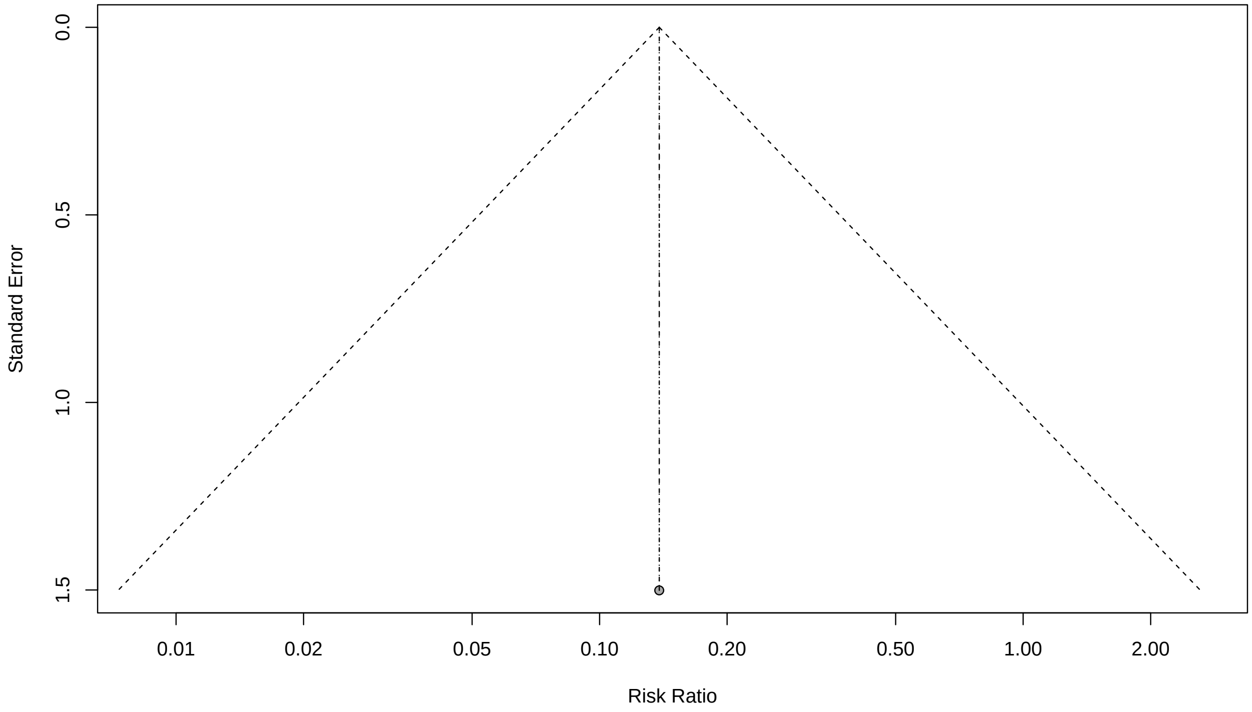

8.14.5 Baujat

```
baujat(stricture_metabin, pos = 1)
```

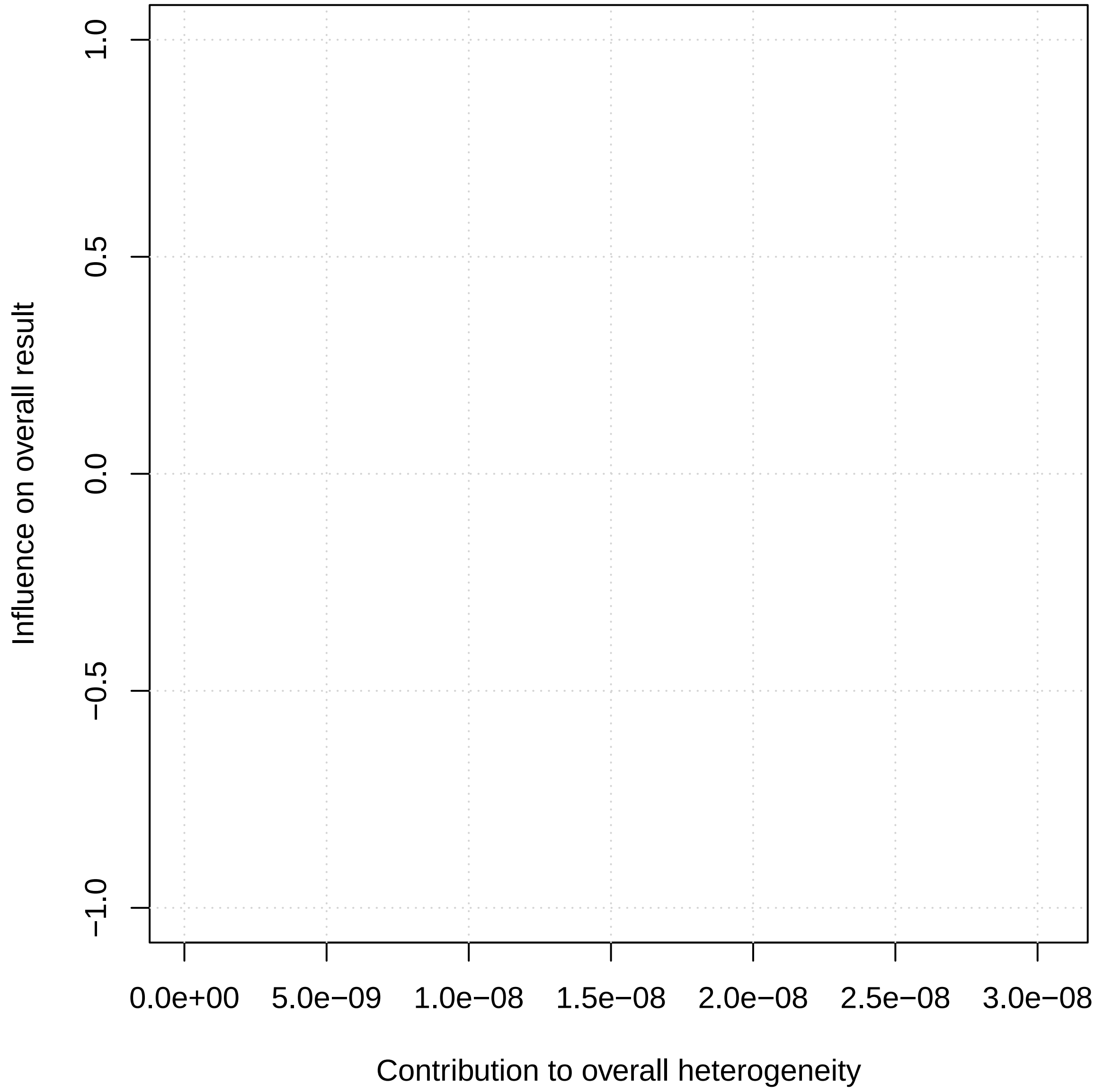

8.14.6 Leave one out

```r
metainf(stricture_metabin)
```

```
## Influential analysis (common effect model)
##
##                                   RR           95%-CI p-value tau^2 tau I^2
## Omitting Du 2019
## Omitting Zhang 2021          0.1383 [0.0073; 2.6222]  0.1876
## Omitting Lechevallier 2003   0.1383 [0.0073; 2.6222]  0.1876
## Omitting Wu 2022             0.1383 [0.0073; 2.6222]  0.1876
## Omitting Zhai 2023           0.1383 [0.0073; 2.6222]  0.1876
## Omitting Zhai 2023           0.1383 [0.0073; 2.6222]  0.1876
## Omitting AlSmadi 2019        0.1383 [0.0073; 2.6222]  0.1876
##
## Pooled estimate              0.1383 [0.0073; 2.6222]  0.1876
##
## Details on meta-analytical method:
## - Mantel-Haenszel method
## - Restricted maximum-likelihood estimator for tau^2
```

8.15 Embolisation required

8.15.1 Meta-analysis

```
ir_embolisation_metabin <- metabin(data = semirigid_only,
                         event.c = embolism_ir_intervention_n_control,
                       n.c = sample_size_control,
                       event.e = embolism_ir_intervention_n_suction,
                       n.e = sample_size_suction,
                       studlab = author_year)
ir_embolisation_metabin
```

```
## Number of studies: k = 0
## Number of observations: o = 754
## Number of events: e = 0
##
##                       RR 95%-CI  z p-value
## Common effect model  NA        --      --
## Random effects model NA        --      --
##
## Quantifying heterogeneity:
##  tau^2 = NA; tau = NA; I^2 = NA; H = NA
##
## Details on meta-analytical method:
## - Mantel-Haenszel method
## - Inverse variance method
## - Restricted maximum-likelihood estimator for tau^2
```

8.15.2 Forest plot

```r
forest(ir_embolisation_metabin,
       sortvar = TE)
```

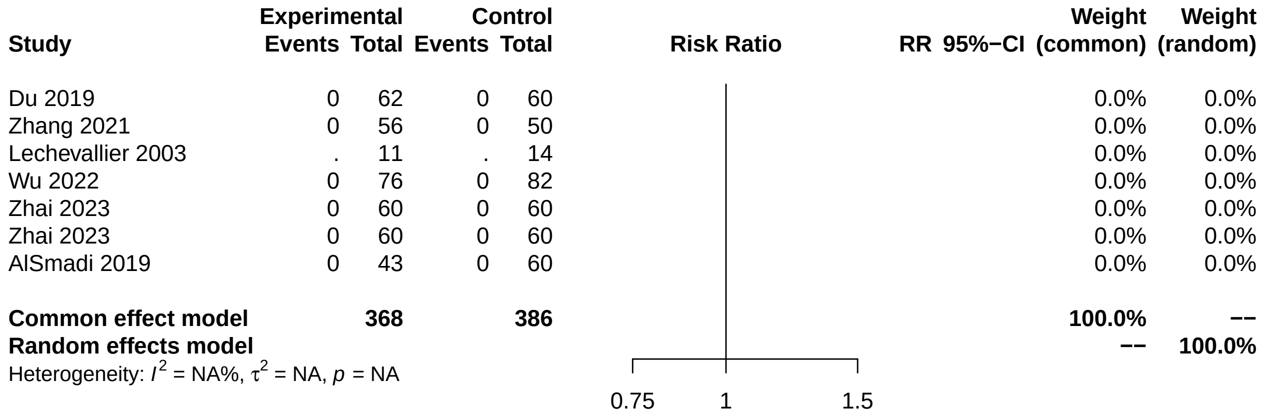

8.15.3 Trim and Fill

```
#trimfill(ir_embolisation_metabin)
#forest(trimfill(ir_embolisation_metabin),
#       sortvar = TE)
```

8.15.4 Funnel plot

```
#funnel((ir_embolisation_metabin))
```

8.15.5 Baujat

```
#baujat(ir_embolisation_metabin, pos = 1)
```

8.15.6 Leave one out

```
#metainf(ir_embolisation_metabin)
```

8.16 Transfusion

8.16.1 Meta-analysis

```
transfusion_metabin <- metabin(data = semirigid_only,
                                 event.c = transfusion_n_control,
                               n.c = sample_size_control,
                               event.e = transfusion_n_suction,
                               n.e = sample_size_suction,
                               studlab = author_year)
transfusion_metabin
```

```
## Number of studies: k = 0
## Number of observations: o = 754
## Number of events: e = 0
##
##                        RR 95%-CI  z p-value
## Common effect model  NA        --      --
## Random effects model NA        --      --
##
## Quantifying heterogeneity:
##  tau^2 = NA; tau = NA; I^2 = NA; H = NA
##
## Details on meta-analytical method:
## - Mantel-Haenszel method
## - Inverse variance method
## - Restricted maximum-likelihood estimator for tau^2
```

8.16.2 Forest plot

```r
forest(transfusion_metabin,
       sortvar = TE)
```

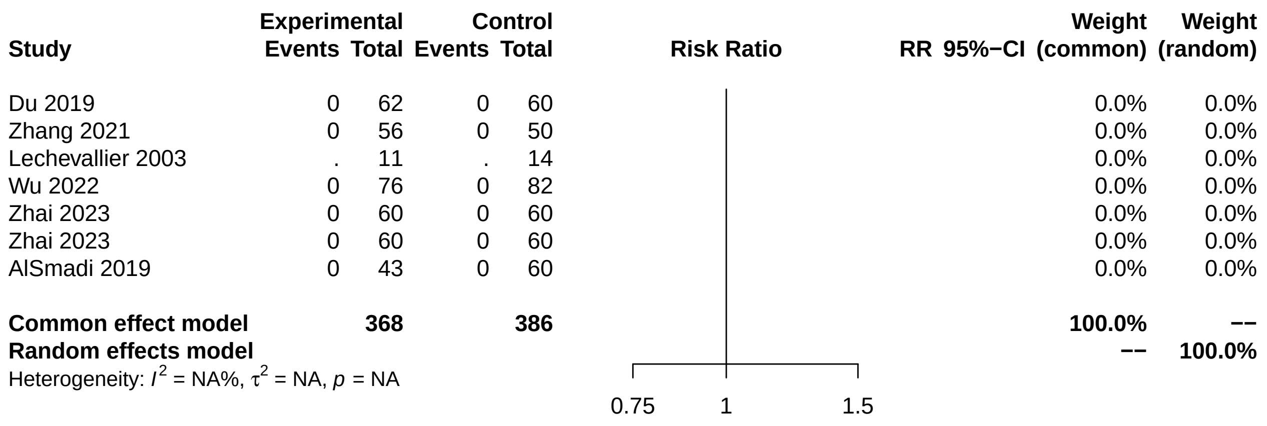

8.16.3 Trim and Fill

```
#trimfill(transfusion_metabin)
#forest(trimfill(transfusion_metabin),
#       sortvar = TE)
```

8.16.4 Funnel plot

```
#funnel(trimfill(transfusion_metabin))
```

8.16.5 Baujat

```
#baujat(transfusion_metabin, pos = 1)
```

8.16.6 Leave one out

```
#metainf(transfusion_metabin)
```

8.17 Clavien I

8.17.1 Meta-analysis

```
clav_i_metabin <- metabin(data = semirigid_only,
                         event.c = clavien_i_n_control,
                       n.c = sample_size_control,
                       event.e = clavien_i_n_suction,
                       n.e = sample_size_suction,
                       studlab = author_year)
clav_i_metabin
```

```
## Number of studies: k = 3
## Number of observations: o = 754
## Number of events: e = 20
##
##                          RR           95%-CI     z p-value
## Common effect model  0.6451 [0.2710; 1.5355] -0.99  0.3218
## Random effects model 0.6491 [0.2711; 1.5539] -0.97  0.3319
##
## Quantifying heterogeneity:
##  tau^2 = 0 [0.0000; 2.8855]; tau = 0 [0.0000; 1.6987]
##  I^2 = 0.0% [0.0%; 89.6%]; H = 1.00 [1.00; 3.10]
##
## Test of heterogeneity:
##     Q d.f. p-value
##  0.27    2  0.8726
##
## Details on meta-analytical method:
## - Mantel-Haenszel method
## - Inverse variance method
## - Restricted maximum-likelihood estimator for tau^2
## - Q-Profile method for confidence interval of tau^2 and tau
```

8.17.2 Forest plot

```r
forest(clav_i_metabin,
       sortvar = TE)
```

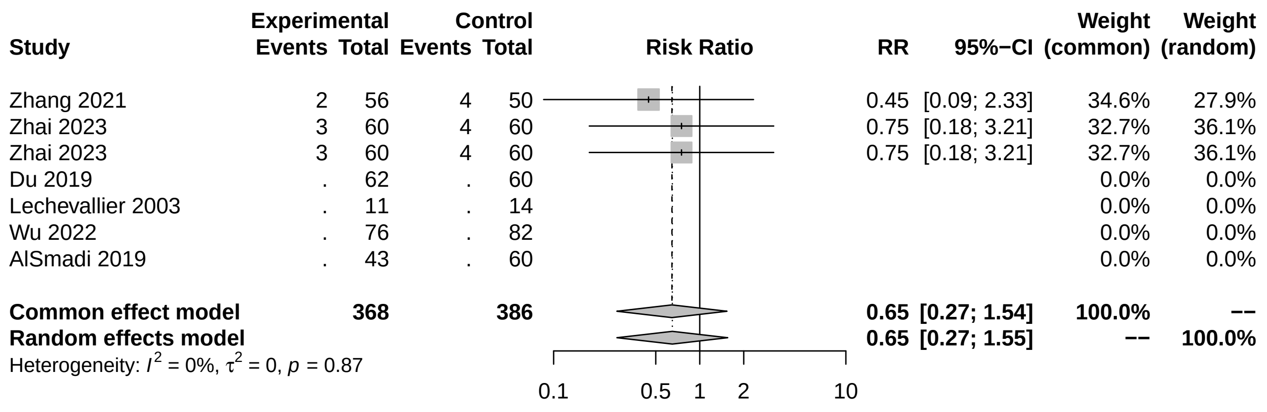

8.17.3 Trim and Fill

```r
trimfill(clav_i_metabin)
```

```
## Warning in trimfill.meta(clav_i_metabin): 4 observation(s) dropped due to
## missing values

## Number of studies: k = 3 (with 0 added studies)
## Number of observations: o = 346
## Number of events: e = 20
##
##                            RR           95%-CI     z p-value
## Random effects model 0.6491 [0.2711; 1.5539] -0.97  0.3319
##
## Quantifying heterogeneity:
##  tau^2 = 0 [0.0000; 2.8855]; tau = 0 [0.0000; 1.6987]
##  I^2 = 0.0% [0.0%; 89.6%]; H = 1.00 [1.00; 3.10]
##
## Test of heterogeneity:
##     Q d.f. p-value
##  0.27    2  0.8726
##
## Details on meta-analytical method:
## - Inverse variance method
## - Restricted maximum-likelihood estimator for tau^2
## - Q-Profile method for confidence interval of tau^2 and tau
## - Trim-and-fill method to adjust for funnel plot asymmetry (L-estimator)
```

```r
forest(trimfill(clav_i_metabin),
       sortvar = TE)
```

```
## Warning in trimfill.meta(clav_i_metabin): 4 observation(s) dropped due to
## missing values
```

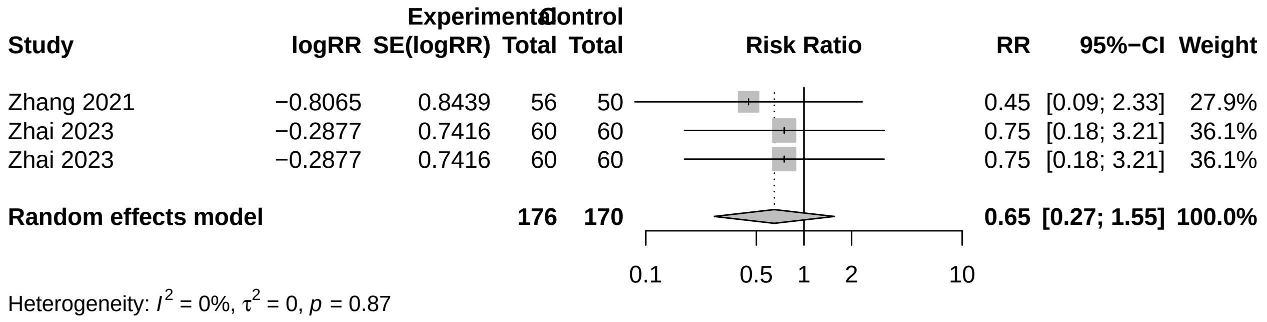

8.17.4 Funnel plot

```
funnel(trimfill(clav_i_metabin))
```

```
## Warning in trimfill.meta(clav_i_metabin): 4 observation(s) dropped due to
## missing values
```

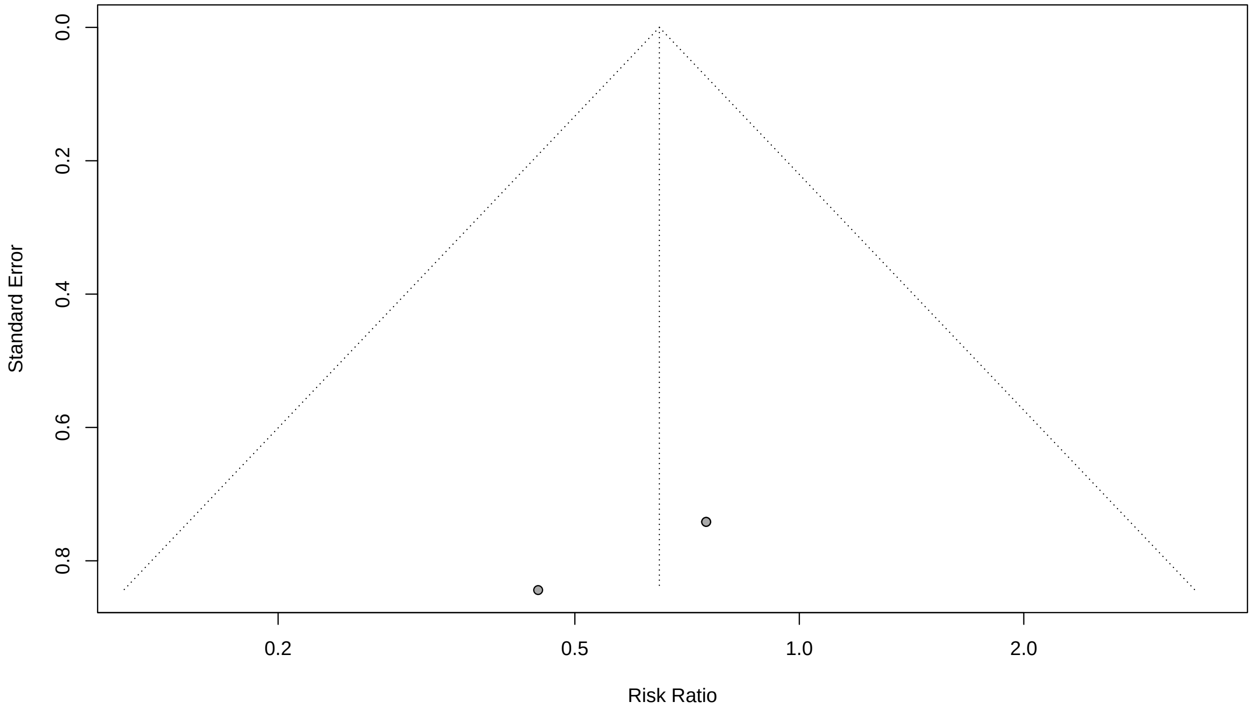

8.17.5 Baujat

```
baujat(clav_i_metabin, pos = 1)
```

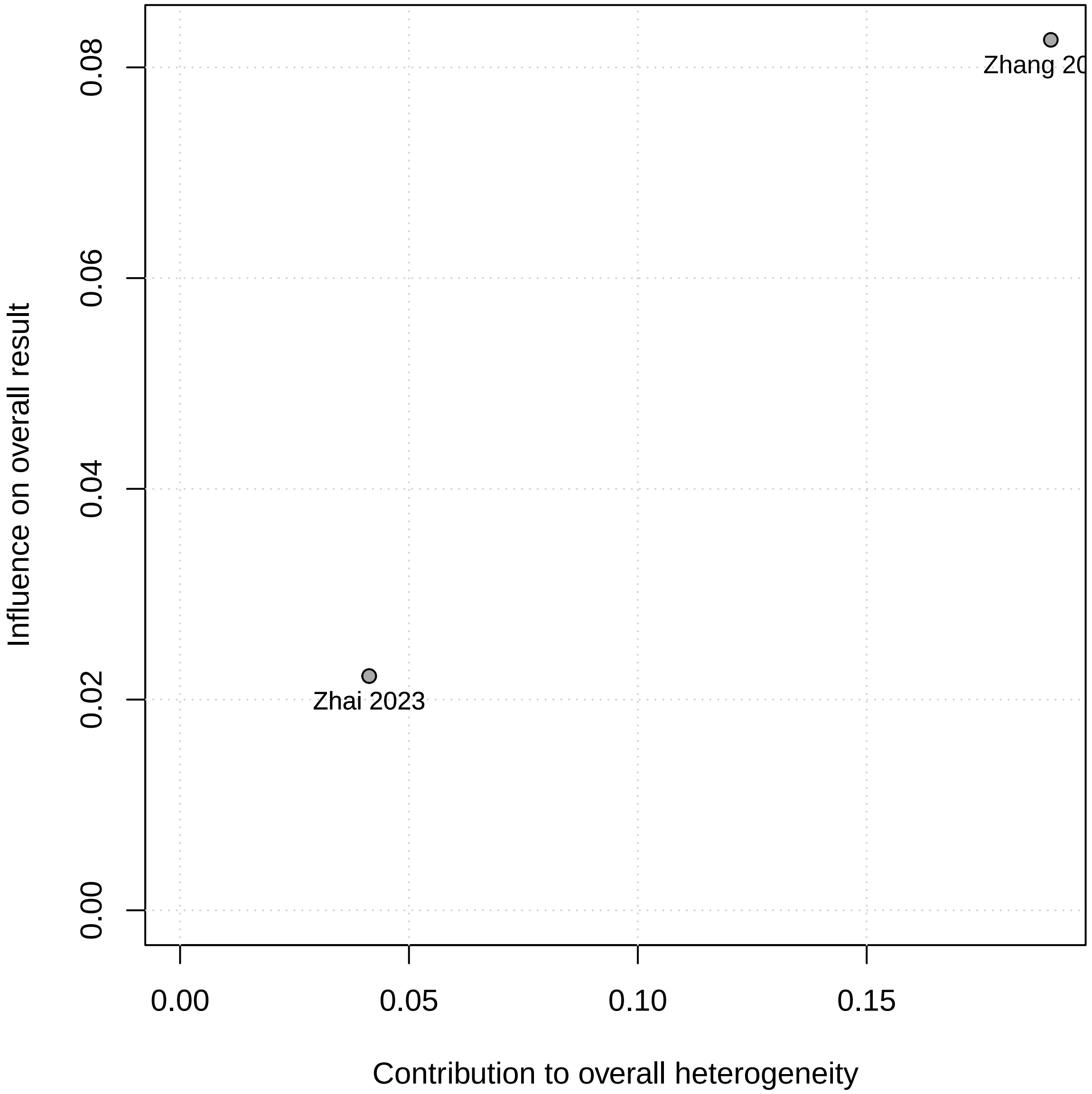

8.17.6 Leave one out

```
metainf(clav_i_metabin)
```

```
## Influential analysis (common effect model)
##
##                                    RR           95%-CI p-value   tau^2     tau
## Omitting Du 2019               0.6451 [0.2710; 1.5355]  0.3218  0.0000  0.0000
## Omitting Zhang 2021            0.7500 [0.2683; 2.0962]  0.5833  0.0000  0.0000
## Omitting Lechevallier 2003     0.6451 [0.2710; 1.5355]  0.3218  0.0000  0.0000
## Omitting Wu 2022               0.6451 [0.2710; 1.5355]  0.3218  0.0000  0.0000
## Omitting Zhai 2023             0.5940 [0.2011; 1.7549]  0.3460  0.0000  0.0000
## Omitting Zhai 2023             0.5940 [0.2011; 1.7549]  0.3460  0.0000  0.0000
## Omitting AlSmadi 2019          0.6451 [0.2710; 1.5355]  0.3218  0.0000  0.0000
##
## Pooled estimate                0.6451 [0.2710; 1.5355]  0.3218  0.0000  0.0000
##                                  I^2
## Omitting Du 2019                0.0%
## Omitting Zhang 2021             0.0%
## Omitting Lechevallier 2003      0.0%
## Omitting Wu 2022                0.0%
## Omitting Zhai 2023              0.0%
## Omitting Zhai 2023              0.0%
## Omitting AlSmadi 2019           0.0%
##
## Pooled estimate                 0.0%
##
## Details on meta-analytical method:
## - Mantel-Haenszel method
## - Restricted maximum-likelihood estimator for tau^2
```

8.18 Clavien II

8.18.1 Meta-analysis

```
clav_ii_metabin <- metabin(data = semirigid_only,
                         event.c = clavien_ii_n_control,
                       n.c = sample_size_control,
                       event.e = clavien_ii_n_suction,
                       n.e = sample_size_suction,
                       studlab = author_year)
clav_ii_metabin
```

```
## Number of studies: k = 2
## Number of observations: o = 754
## Number of events: e = 4
##
##                           RR           95%-CI    z p-value
## Common effect model  1.0000 [0.1432; 6.9836] 0.00  1.0000
## Random effects model 1.0000 [0.1432; 6.9836] 0.00  1.0000
##
## Quantifying heterogeneity:
##  tau^2 = 0; tau = 0; I^2 = 0.0%; H = 1.00
##
## Test of heterogeneity:
##     Q d.f. p-value
##  0.00    1  1.0000
##
## Details on meta-analytical method:
## - Mantel-Haenszel method
## - Inverse variance method
## - Restricted maximum-likelihood estimator for tau^2
```

8.18.2 Forest plot

```r
forest(clav_ii_metabin,
       sortvar = TE)
```

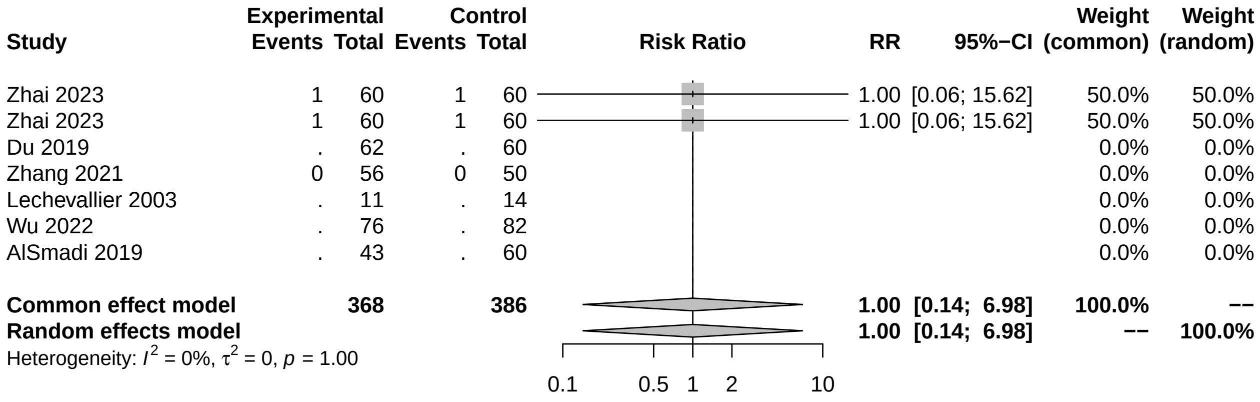

8.18.3 Trim and Fill

```r
#trimfill(clav_ii_metabin)
#forest(trimfill(clav_ii_metabin),
#       sortvar = TE)
```

8.18.4 Funnel plot

```
funnel((clav_ii_metabin))
```

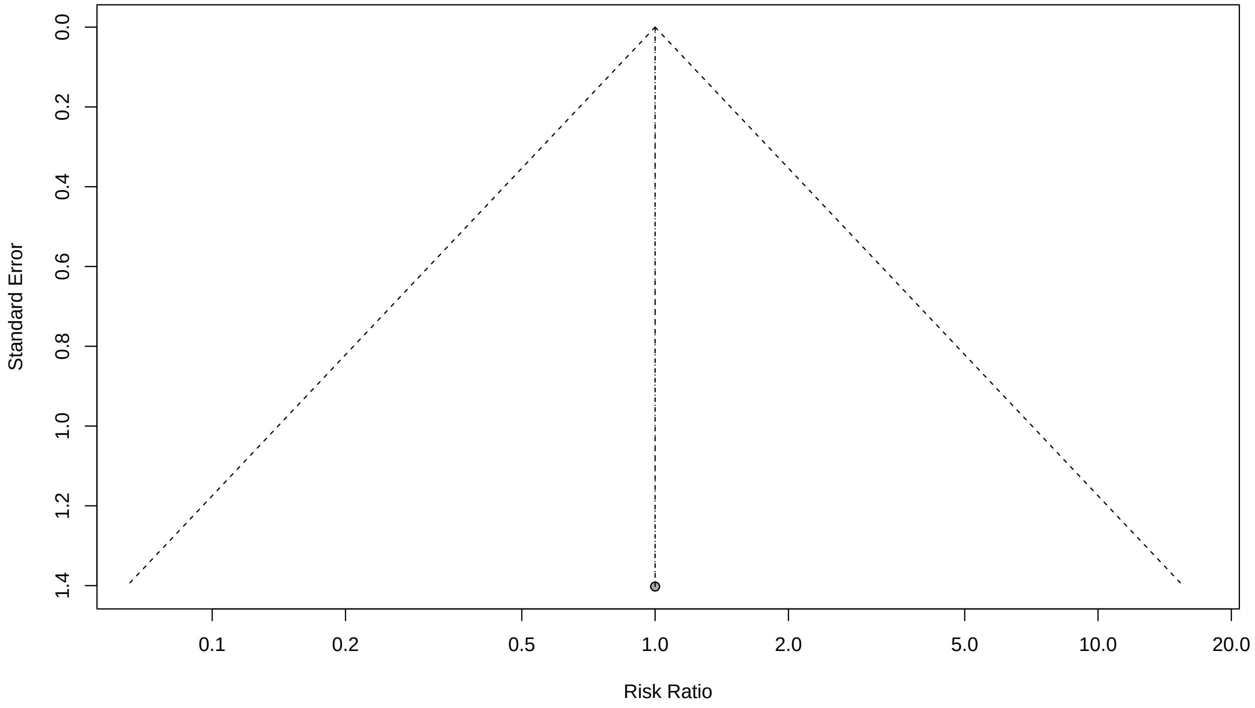

8.18.5 Baujat

```
baujat(clav_ii_metabin, pos = 1)
```

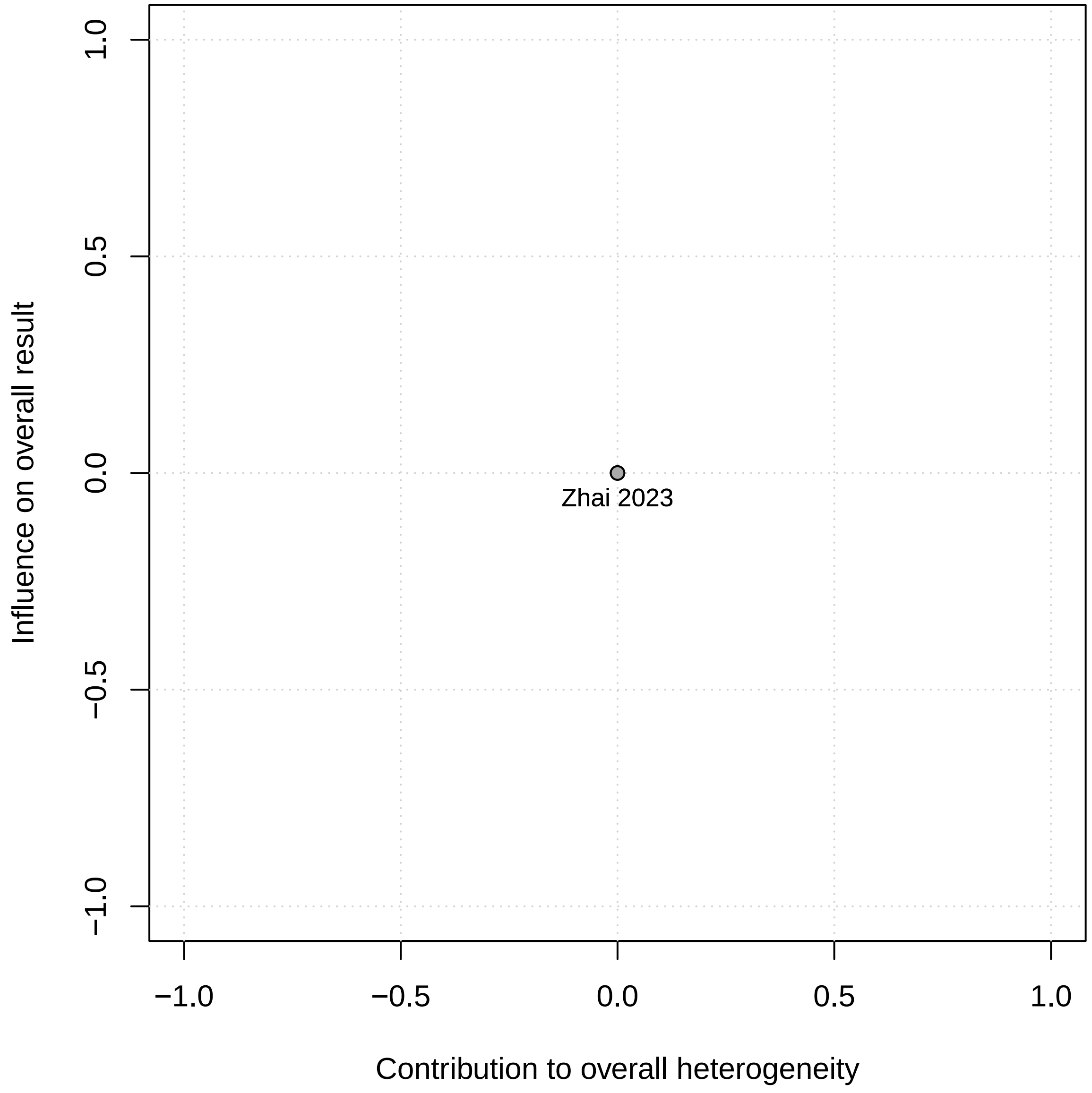

8.18.6 Leave one out

```r
metainf(clav_ii_metabin)
```

```
## Influential analysis (common effect model)
##
##                                  RR            95%-CI p-value   tau^2     tau
## Omitting Du 2019             1.0000 [0.1432;  6.9836]  1.0000  0.0000  0.0000
## Omitting Zhang 2021          1.0000 [0.1432;  6.9836]  1.0000  0.0000  0.0000
## Omitting Lechevallier 2003   1.0000 [0.1432;  6.9836]  1.0000  0.0000  0.0000
## Omitting Wu 2022             1.0000 [0.1432;  6.9836]  1.0000  0.0000  0.0000
## Omitting Zhai 2023           1.0000 [0.0640; 15.6209]  1.0000
## Omitting Zhai 2023           1.0000 [0.0640; 15.6209]  1.0000
## Omitting AlSmadi 2019        1.0000 [0.1432;  6.9836]  1.0000  0.0000  0.0000
##
## Pooled estimate              1.0000 [0.1432;  6.9836]  1.0000  0.0000  0.0000
##                                I^2
## Omitting Du 2019              0.0%
## Omitting Zhang 2021           0.0%
## Omitting Lechevallier 2003    0.0%
## Omitting Wu 2022              0.0%
## Omitting Zhai 2023
## Omitting Zhai 2023
## Omitting AlSmadi 2019         0.0%
##
## Pooled estimate               0.0%
##
## Details on meta-analytical method:
## - Mantel-Haenszel method
## - Restricted maximum-likelihood estimator for tau^2
```

8.19 Clavien III

8.19.1 Meta-analysis

```
clav_iii_metabin <- metabin(data = semirigid_only,
                         event.c = clavien_iii_n_control,
                       n.c = sample_size_control,
                       event.e = clavien_iii_n_suction,
                       n.e = sample_size_suction,
                       studlab = author_year)
clav_iii_metabin
```

```
## Number of studies: k = 0
## Number of observations: o = 754
## Number of events: e = 0
##
##                        RR 95%-CI  z p-value
## Common effect model  NA         --      --
## Random effects model NA         --      --
##
## Quantifying heterogeneity:
##  tau^2 = NA; tau = NA; I^2 = NA; H = NA
##
## Details on meta-analytical method:
## - Mantel-Haenszel method
## - Inverse variance method
## - Restricted maximum-likelihood estimator for tau^2
```

8.19.2 Forest plot

```r
forest(clav_iii_metabin,
       sortvar = TE)
```

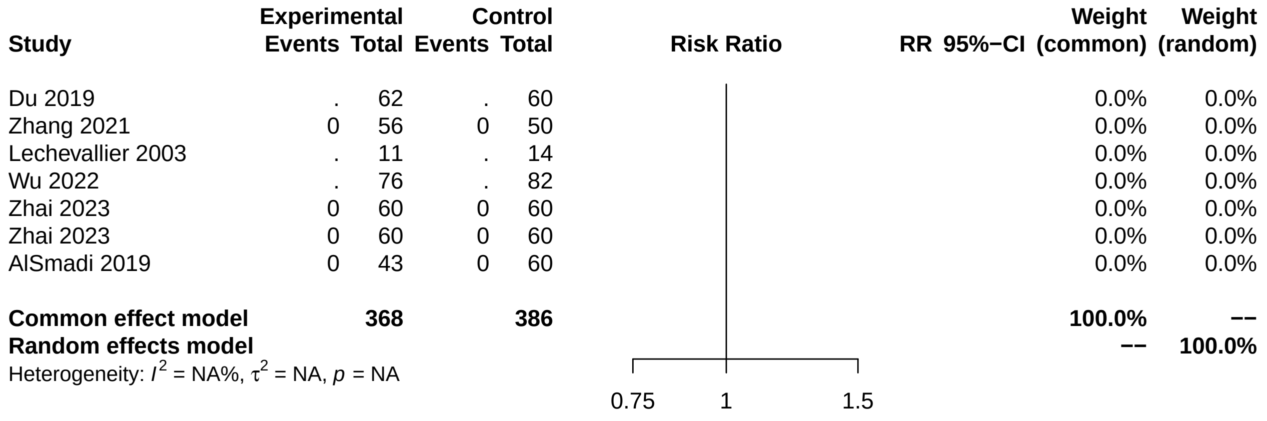

8.19.3 Trim and Fill

```
#trimfill(clav_iii_metabin)
#forest(trimfill(clav_iii_metabin),
#       sortvar = TE)
```

8.19.4 Funnel plot

```
#funnel(trimfill(clav_iii_metabin))
```

8.19.5 Baujat

```
#baujat(clav_iii_metabin, pos = 1)
```

8.19.6 Leave one out

```
#metainf(clav_iii_metabin)
```

8.20 Clavien IV

8.20.1 Meta-analysis

```
clav_iv_metabin <- metabin(data = semirigid_only,
                         event.c = clavien_iv_n_control,
                       n.c = sample_size_control,
                       event.e = clavien_iv_n_suction,
                       n.e = sample_size_suction,
                       studlab = author_year)
clav_iv_metabin
```

```
## Number of studies: k = 0
## Number of observations: o = 754
## Number of events: e = 0
##
##                        RR 95%-CI  z p-value
## Common effect model  NA        --      --
## Random effects model NA        --      --
##
## Quantifying heterogeneity:
##  tau^2 = NA; tau = NA; I^2 = NA; H = NA
##
## Details on meta-analytical method:
## - Mantel-Haenszel method
## - Inverse variance method
## - Restricted maximum-likelihood estimator for tau^2
```

8.20.2 Forest plot

```r
forest(clav_iv_metabin,
       sortvar = TE)
```

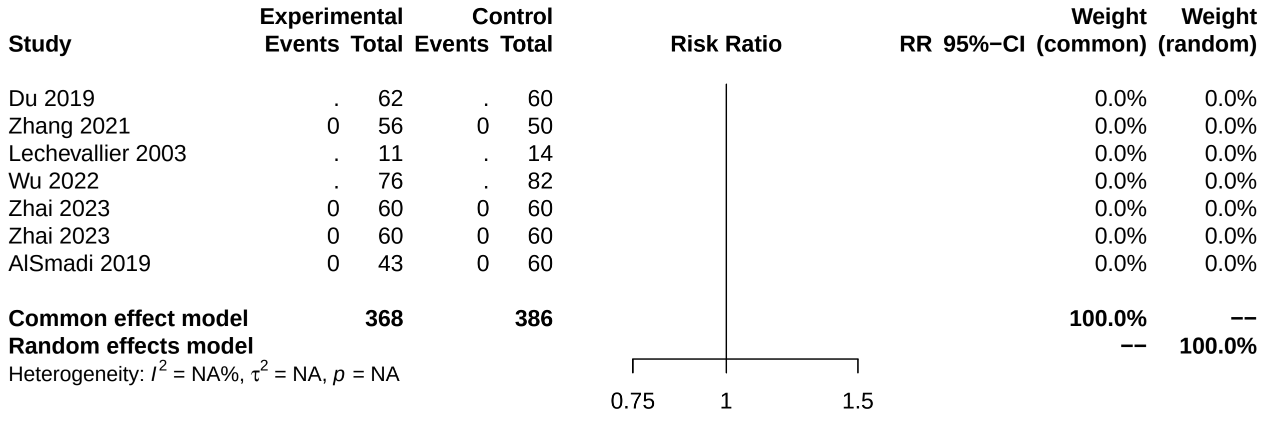

8.20.3 Trim and Fill

```
#trimfill(clav_iv_metabin)
#forest(trimfill(clav_iv_metabin),
#       sortvar = TE)
```

8.20.4 Funnel plot

```
#funnel((clav_iv_metabin))
```

8.20.5 Baujat

```
#baujat(clav_iv_metabin, pos = 1)
```

8.20.6 Leave one out

```
#metainf(clav_iv_metabin)
```

8.21 Clavien V

8.21.1 Meta-analysis

```
clav_v_metabin <- metabin(data = semirigid_only,
                         event.c = clavien_v_n_control,
                       n.c = sample_size_control,
                       event.e = clavien_v_n_suction,
                       n.e = sample_size_suction,
                       studlab = author_year)
clav_v_metabin
```

```
## Number of studies: k = 0
## Number of observations: o = 754
## Number of events: e = 0
##
##                        RR 95%-CI  z p-value
## Common effect model  NA         --      --
## Random effects model NA         --      --
##
## Quantifying heterogeneity:
##  tau^2 = NA; tau = NA; I^2 = NA; H = NA
##
## Details on meta-analytical method:
## - Mantel-Haenszel method
## - Inverse variance method
## - Restricted maximum-likelihood estimator for tau^2
```

8.21.2 Forest plot

```
#forest(clav_v_metabin,
#       sortvar = TE)
```

8.21.3 Trim and Fill

```
#trimfill(clav_v_metabin)
#forest(trimfill(clav_v_metabin),
#        sortvar = TE)
```

8.21.4 Funnel plot

```
#funnel(trimfill(clav_v_metabin))
```

8.21.5 Baujat

```r
#baujat(clav_v_metabin, pos = 1)
```

8.21.6 Leave one out

```
#metainf(clav_v_metabin)
```

8.22 Clavien I-II

8.22.1 Meta-analysis

```
clav_i_ii_metabin <- metabin(data = semirigid_only,
                              event.c = clav_i_ii_n_control,
                            n.c = sample_size_control,
                            event.e = clav_i_ii_n_suction,
                            n.e = sample_size_suction,
                            studlab = author_year)
clav_i_ii_metabin
```

```
## Number of studies: k = 3
## Number of observations: o = 754
## Number of events: e = 24
##
##                           RR           95%-CI     z p-value
## Common effect model  0.6950 [0.3182; 1.5179] -0.91  0.3613
## Random effects model 0.7011 [0.3192; 1.5399] -0.88  0.3763
##
## Quantifying heterogeneity:
##  tau^2 = 0 [0.0000; 3.8664]; tau = 0 [0.0000; 1.9663]
##  I^2 = 0.0% [0.0%; 89.6%]; H = 1.00 [1.00; 3.10]
##
## Test of heterogeneity:
##     Q d.f. p-value
##  0.37    2  0.8312
##
## Details on meta-analytical method:
## - Mantel-Haenszel method
## - Inverse variance method
## - Restricted maximum-likelihood estimator for tau^2
## - Q-Profile method for confidence interval of tau^2 and tau
```

8.22.2 Forest plot

```r
forest(clav_i_ii_metabin,
       sortvar = TE)
```

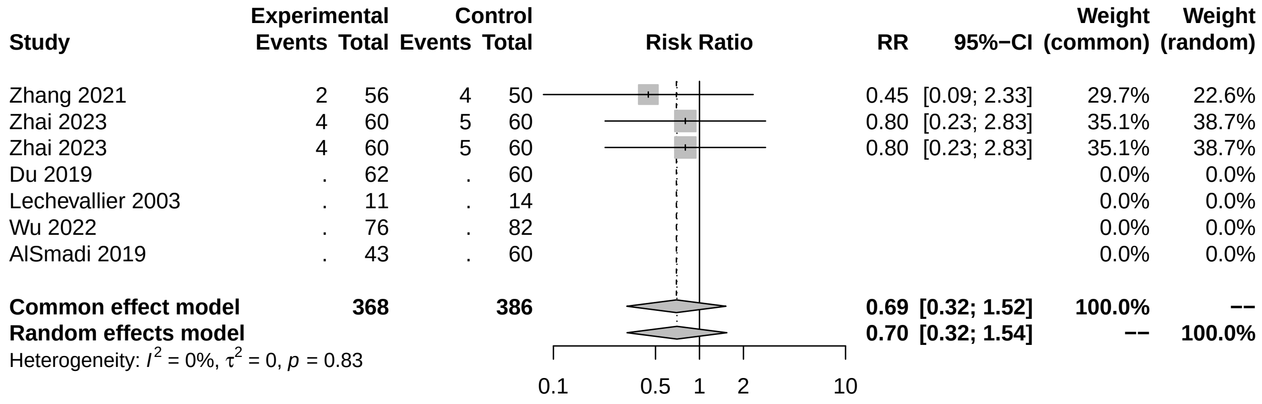

8.22.3 Trim and Fill

```r
trimfill(clav_i_ii_metabin)
```

```
## Warning in trimfill.meta(clav_i_ii_metabin): 4 observation(s) dropped due to
## missing values
```

```
## Number of studies: k = 3 (with 0 added studies)
## Number of observations: o = 346
## Number of events: e = 24
##
##                          RR           95%-CI     z p-value
## Random effects model 0.7011 [0.3192; 1.5399] -0.88  0.3763
##
## Quantifying heterogeneity:
##  tau^2 = 0 [0.0000; 3.8664]; tau = 0 [0.0000; 1.9663]
##  I^2 = 0.0% [0.0%; 89.6%]; H = 1.00 [1.00; 3.10]
##
## Test of heterogeneity:
##     Q d.f. p-value
##  0.37    2  0.8312
##
## Details on meta-analytical method:
## - Inverse variance method
## - Restricted maximum-likelihood estimator for tau^2
## - Q-Profile method for confidence interval of tau^2 and tau
## - Trim-and-fill method to adjust for funnel plot asymmetry (L-estimator)
```

```r
forest(trimfill(clav_i_ii_metabin),
       sortvar = TE)
```

```
## Warning in trimfill.meta(clav_i_ii_metabin): 4 observation(s) dropped due to
## missing values
```

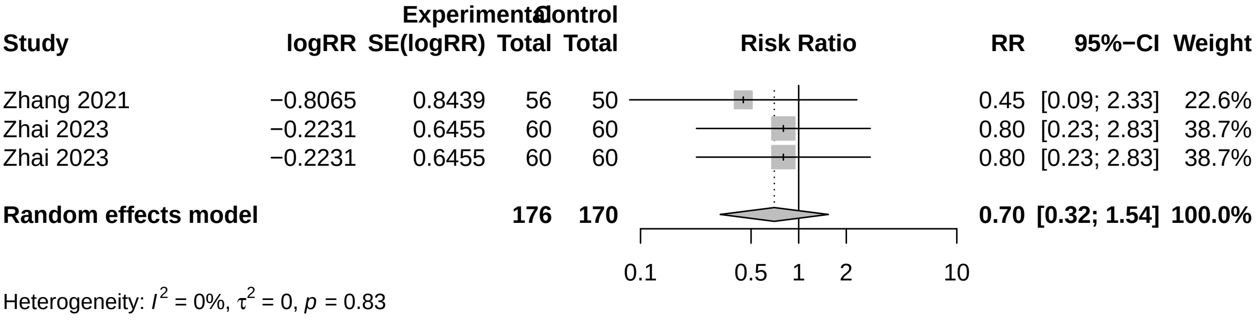

8.22.4 Funnel plot

```
funnel(trimfill(clav_i_ii_metabin))
```

```
## Warning in trimfill.meta(clav_i_ii_metabin): 4 observation(s) dropped due to
## missing values
```

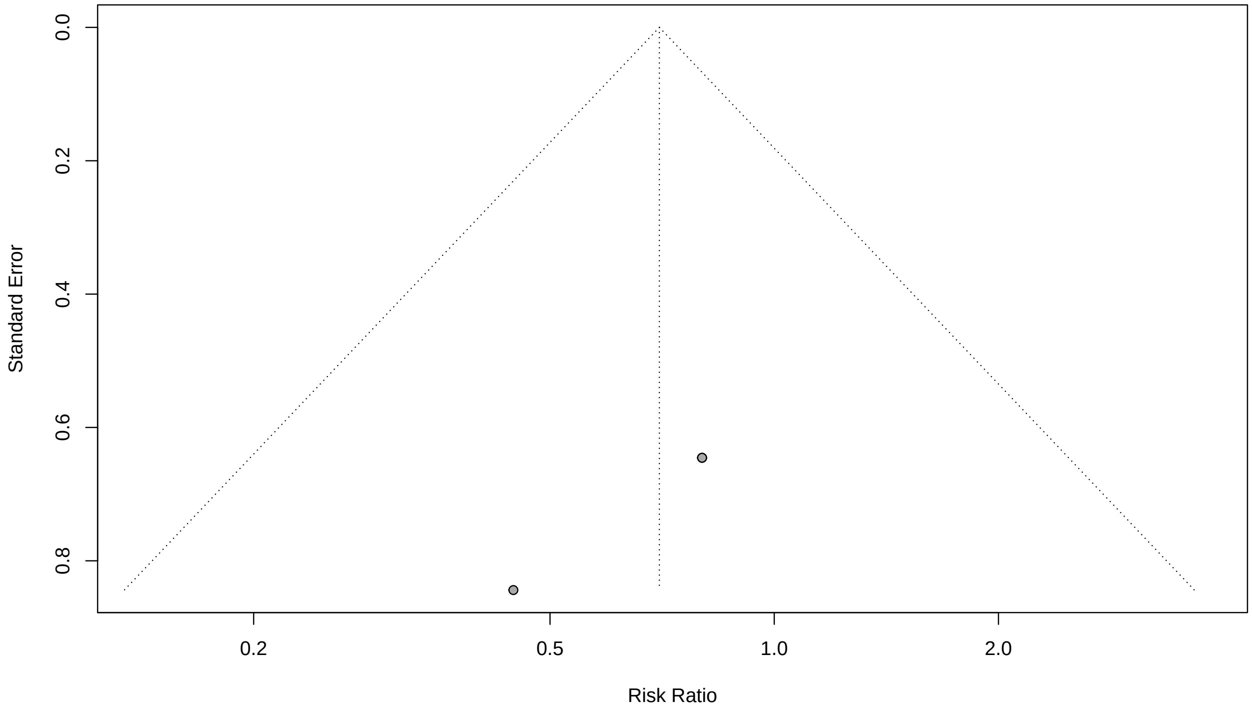

8.22.5 Baujat

```
baujat(clav_i_ii_metabin, pos = 1)
```

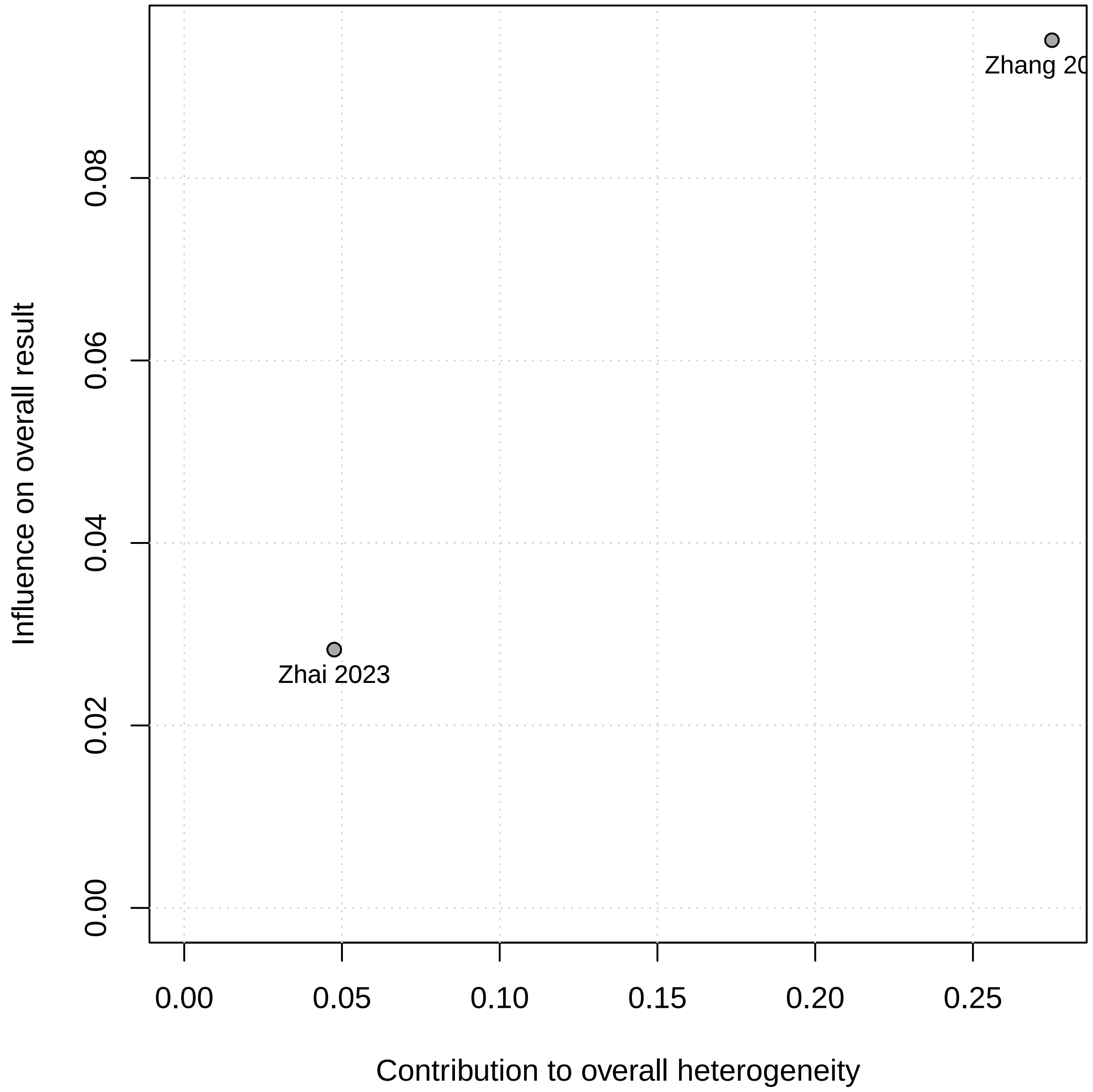

8.22.6 Leave one out

```
metainf(clav_i_ii_metabin)
```

```
## Influential analysis (common effect model)
##
##                                   RR           95%-CI p-value   tau^2    tau
## Omitting Du 2019              0.6950 [0.3182; 1.5179]  0.3613  0.0000  0.0000
## Omitting Zhang 2021           0.8000 [0.3270; 1.9571]  0.6249  0.0000  0.0000
## Omitting Lechevallier 2003    0.6950 [0.3182; 1.5179]  0.3613  0.0000  0.0000
## Omitting Wu 2022              0.6950 [0.3182; 1.5179]  0.3613  0.0000  0.0000
## Omitting Zhai 2023            0.6380 [0.2358; 1.7266]  0.3763  0.0000  0.0000
## Omitting Zhai 2023            0.6380 [0.2358; 1.7266]  0.3763  0.0000  0.0000
## Omitting AlSmadi 2019         0.6950 [0.3182; 1.5179]  0.3613  0.0000  0.0000
##
## Pooled estimate               0.6950 [0.3182; 1.5179]  0.3613  0.0000  0.0000
##                                 I^2
## Omitting Du 2019               0.0%
## Omitting Zhang 2021            0.0%
## Omitting Lechevallier 2003     0.0%
## Omitting Wu 2022               0.0%
## Omitting Zhai 2023             0.0%
## Omitting Zhai 2023             0.0%
## Omitting AlSmadi 2019          0.0%
##
## Pooled estimate                0.0%
##
## Details on meta-analytical method:
## - Mantel-Haenszel method
## - Restricted maximum-likelihood estimator for tau^2
```

8.23 Clavien III-V

8.23.1 Meta-analysis

```
clav_iii_v_metabin <- metabin(data = semirigid_only,
                          event.c = clav_iii_v_n_control,
                        n.c = sample_size_control,
                        event.e = clav_iii_v_n_suction,
                        n.e = sample_size_suction,
                        studlab = author_year)
clav_iii_v_metabin
```

```
## Number of studies: k = 0
## Number of observations: o = 754
## Number of events: e = 0
##
##                        RR 95%-CI  z p-value
## Common effect model  NA        --      --
## Random effects model NA        --      --
##
## Quantifying heterogeneity:
##  tau^2 = NA; tau = NA; I^2 = NA; H = NA
##
## Details on meta-analytical method:
## - Mantel-Haenszel method
## - Inverse variance method
## - Restricted maximum-likelihood estimator for tau^2
```

8.23.2 Forest plot

```r
forest(clav_iii_v_metabin,
       sortvar = TE)
```

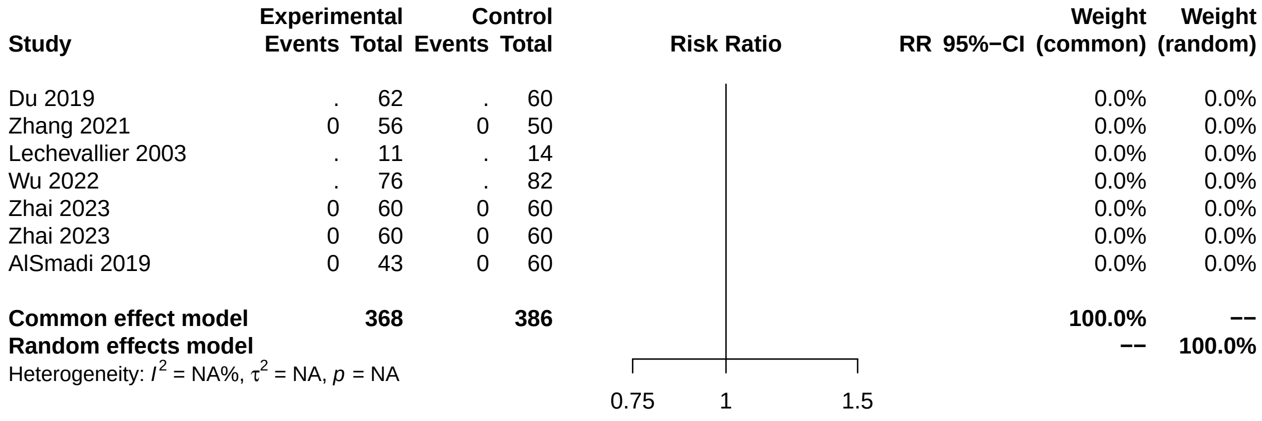

8.23.3 Trim and Fill

```
#trimfill(clav_iii_v_metabin)
#forest(trimfill(clav_iii_v_metabin),
#       sortvar = TE)
```

8.23.4 Funnel plot

```
#funnel(trimfill(clav_iii_v_metabin))
```

8.23.5 Baujat

```
#baujat(clav_iii_v_metabin, pos = 1)
```

8.23.6 Leave one out

```
#metainf(clav_iii_v_metabin)
```

9 Semi-Rigid URS Only Summary Forest plots

```
overall <- cbind(
  "Outcome" = c(
    "Immediate SFR",
    "Final SFR",
    "Auxiliary Treatment",
    "Overall Complications",
    "Fever",
    "Infection",
    "Sepsis",
    "Abscess",
    "Haematoma",
    "Pain",
    "Stricture",
    "Embolisation",
    "Transfusion",
    "CD I",
    "CD II",
    "CD III",
    "CD IV",
    "CD I-II",
    "CD III-V",
    "Operative time",
    "VAS",
    "Length of Stay"
  ),
  "type" = c(
    "binary",
    "binary",
    "binary",
    "binary",
    "binary",
    "binary",
    "binary",
    "binary",
    "binary",
    "binary",
    "binary",
    "binary",
    "binary",
    "binary",
    "binary",
    "binary",
    "binary",
    "binary",
    "binary",
    "cont",
    "cont",
    "cont"
  ),
  "n_studies" = c(
    immediate_sfr_metabin$k,
```

```
  sfr_metabin$k,
aux_rx_metabin$k,
comp_metabin$k,
fever_metabin$k,
infection_metabin$k,
sepsis_metabin$k,
abscess_metabin$k,
haematoma_metabin$k,
pain_metabin$k,
stricture_metabin$k,
ir_embolisation_metabin$k,
transfusion_metabin$k,
clav_i_metabin$k,
clav_ii_metabin$k,
clav_iii_metabin$k,
clav_iv_metabin$k,
clav_i_ii_metabin$k,
clav_iii_v_metabin$k,
or_time_metacont$k,
vas_metacont$k,
los_metacont$k),
"es" = c(
  immediate_sfr_metabin$TE.random %>% exp(),
  sfr_metabin$TE.random %>% exp(),
aux_rx_metabin$TE.random %>% exp(),
comp_metabin$TE.random %>% exp(),
fever_metabin$TE.random %>% exp(),
infection_metabin$TE.random %>% exp(),
sepsis_metabin$TE.random %>% exp(),
abscess_metabin$TE.random %>% exp(),
haematoma_metabin$TE.random %>% exp(),
pain_metabin$TE.random %>% exp(),
stricture_metabin$TE.random %>% exp(),
ir_embolisation_metabin$TE.random %>% exp(),
transfusion_metabin$TE.random %>% exp(),
clav_i_metabin$TE.random %>% exp(),
clav_ii_metabin$TE.random %>% exp(),
clav_iii_metabin$TE.random %>% exp(),
clav_iv_metabin$TE.random %>% exp(),
clav_i_ii_metabin$TE.random %>% exp(),
clav_iii_v_metabin$TE.random %>% exp(),
or_time_metacont$TE.random,
vas_metacont$TE.random,
los_metacont$TE.random),
"lower_ci" = c(
  immediate_sfr_metabin$lower.random %>% exp(),
  sfr_metabin$lower.random %>% exp(),
aux_rx_metabin$lower.random %>% exp(),
comp_metabin$lower.random %>% exp(),
fever_metabin$lower.random %>% exp(),
infection_metabin$lower.random %>% exp(),
sepsis_metabin$lower.random %>% exp(),
abscess_metabin$lower.random %>% exp(),
```

```
haematoma_metabin$lower.random %>% exp(),
pain_metabin$lower.random %>% exp(),
stricture_metabin$lower.random %>% exp(),
ir_embolisation_metabin$lower.random %>% exp(),
transfusion_metabin$lower.random %>% exp(),
clav_i_metabin$lower.random %>% exp(),
clav_ii_metabin$lower.random %>% exp(),
clav_iii_metabin$lower.random %>% exp(),
clav_iv_metabin$lower.random %>% exp(),
clav_i_ii_metabin$lower.random %>% exp(),
clav_iii_v_metabin$lower.random %>% exp(),
or_time_metacont$lower.random,
vas_metacont$lower.random,
los_metacont$lower.random),
"upper_ci" = c(immediate_sfr_metabin$upper.random %>% exp(),
               sfr_metabin$upper.random %>% exp(),
aux_rx_metabin$upper.random %>% exp(),
comp_metabin$upper.random %>% exp(),
fever_metabin$upper.random %>% exp(),
infection_metabin$upper.random %>% exp(),
sepsis_metabin$upper.random %>% exp(),
abscess_metabin$upper.random %>% exp(),
haematoma_metabin$upper.random %>% exp(),
pain_metabin$upper.random %>% exp(),
stricture_metabin$upper.random %>% exp(),
ir_embolisation_metabin$upper.random %>% exp(),
transfusion_metabin$upper.random %>% exp(),
clav_i_metabin$upper.random %>% exp(),
clav_ii_metabin$upper.random %>% exp(),
clav_iii_metabin$upper.random %>% exp(),
clav_iv_metabin$upper.random %>% exp(),
clav_i_ii_metabin$upper.random %>% exp(),
clav_iii_v_metabin$upper.random %>% exp(),
or_time_metacont$upper.random,
vas_metacont$upper.random,
los_metacont$upper.random),
"p" = c(
  immediate_sfr_metabin$pval.random,
  sfr_metabin$pval.random,
aux_rx_metabin$pval.random,
comp_metabin$pval.random,
fever_metabin$pval.random,
infection_metabin$pval.random,
sepsis_metabin$pval.random,
abscess_metabin$pval.random,
haematoma_metabin$pval.random,
pain_metabin$pval.random,
stricture_metabin$pval.random,
ir_embolisation_metabin$pval.random,
transfusion_metabin$pval.random,
clav_i_metabin$pval.random,
clav_ii_metabin$pval.random,
clav_iii_metabin$pval.random,
```

```r
clav_iv_metabin$pval.random,
clav_i_ii_metabin$pval.random,
clav_iii_v_metabin$pval.random,
or_time_metacont$pval.random,
vas_metacont$pval.random,
los_metacont$pval.random)) %>% as_tibble() %>% drop_na(es)

overall$es <- as.numeric(overall$es)
overall$es <- round(overall$es, digits = 2)

overall$lower_ci <- as.numeric(overall$lower_ci)
overall$lower_ci <- round(overall$lower_ci, digits = 2)

overall$upper_ci <- as.numeric(overall$upper_ci)
overall$upper_ci <- round(overall$upper_ci, digits = 2)

overall$p <- as.numeric(overall$p)
overall$p <- round(overall$p, digits = 2)
overall$p <- ifelse(overall$p<0.001, "<0.001", overall$p)
```

9.1 Summary Table of number of studies for each outcome included in meta-analysis

```
overall %>% subset(select = c(Outcome, n_studies)) %>% gt() %>% tab_header(title = "Summary table of Nu
```

Summary table of Number of Studies for Each Outcome Meta-Analyis

Outcome	Studies, n
Immediate SFR	9
Final SFR	6
Auxiliary Treatment	5
Overall Complications	6
Fever	3
Infection	3
Pain	1
Stricture	1
CD I	3
CD II	2
CD I-II	3
Operative time	7
Length of Stay	4

9.2 Summary Forest plot of Continuous outcomes

9.2.1 Continuous Outcomes Table

md = mean difference lb = lower bound of 95% confidence interval ub = upper bound of 95% confidence interval tf = trim and fill

```
overall_continuous <-
  overall %>% subset(type ==  "cont") %>% subset(select = c(Outcome,
                                                            n_studies,
                                                            es,
                                                            lower_ci,
                                                            upper_ci,
                                                            p)) %>% as_tibble()
overall_continuous %>% gt() %>% tab_header(title = "Summary Table for Continuous Outcomes") %>% cols_me
```

Summary Table for Continuous Outcomes

Outcome	Studies, n	MD (95% CI)	p
Operative time	7	-6.87 (-14.07-0.33)	0.06
Length of Stay	4	-0.29 (-0.55–0.03)	0.03

9.2.2 Continuous Forest plot

```
continuous_plot <- overall_continuous %>%
  forestplot(
    mean = es,
    lower = lower_ci,
    upper = upper_ci,
    labeltext = c(Outcome, n_studies, es, lower_ci, upper_ci, p),
  zero = 0,
  vertices = TRUE,
  title = "A. Forest plot of MA Outcomes for Continuous Outcomes",
  clip = c(-4, 2),
  xlab = "MD [95% CI]",
  graph.pos = 3
) %>% fp_set_style(
    box = c("black"),
    line = "black",
    txt_gp = fpTxtGp(
      ticks = gpar(fontfamily = "", cex = 1),
      xlab  = gpar(fontfamily = "", cex = 1)
    )) %>% fp_add_lines("steelblue") %>%
  fp_add_header("Outcome",
                "Studies, n",
                  "MD",
                "[ 95%", " CI ]",
                "P-Value")  %>% fp_decorate_graph(box = TRUE) %>% fp_set_zebra_style("#EFEFEF")
continuous_plot
```

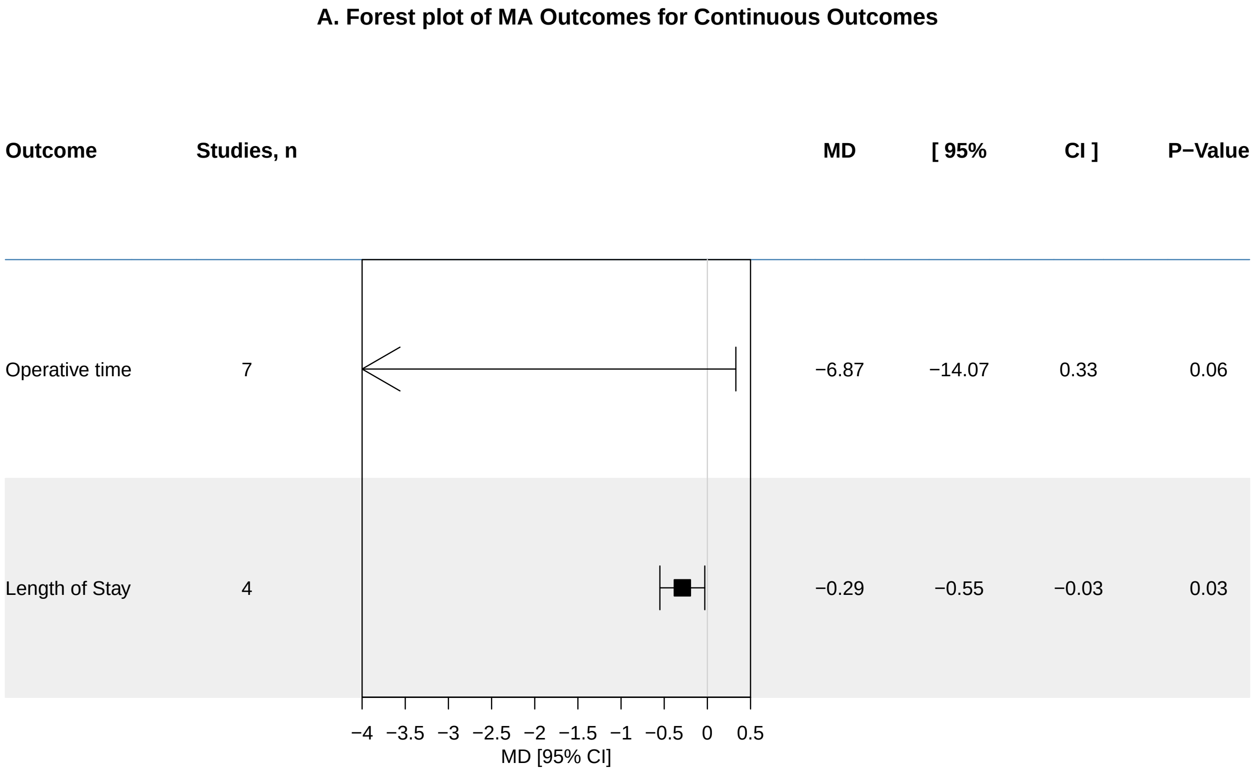

9.3 Summary Forest plot of Binary outcomes

9.3.1 Binary Outcomes Table

md = mean difference lb = lower bound of 95% confidence interval ub = upper bound of 95% confidence interval tf = trim and fill

```
overall_binary <-
  overall %>% subset(type ==  "binary") %>% subset(select = c(Outcome,
                                                               n_studies,
                                                               es,
                                                               lower_ci,
                                                               upper_ci,
                                                               p)) %>% as_tibble()
overall_binary %>% gt() %>% tab_header(title = "Summary Table for Binary Outcomes") %>% cols_merge(colu
```

Summary Table for Binary Outcomes

Outcome	Studies, n	RR (95% CI)	p
Immediate SFR	9	1.15 (0.99-1.34)	0.08
Final SFR	6	1.16 (1.07-1.25)	<0.001
Auxiliary Treatment	5	0.57 (0.13-2.40)	0.44
Overall Complications	6	0.45 (0.25-0.83)	0.01
Fever	3	0.29 (0.12-0.72)	0.01
Infection	3	0.29 (0.12-0.72)	0.01
Pain	1	0.30 (0.01-7.15)	0.46
Stricture	1	0.14 (0.01-2.62)	0.19
CD I	3	0.65 (0.27-1.55)	0.33
CD II	2	1.00 (0.14-6.98)	1
CD I-II	3	0.70 (0.32-1.54)	0.38

9.3.2 Binary Forest plot - Meta-Analysis

Reference = No Suction

```r
binary_plot <- overall_binary %>%
  forestplot(
    mean = es,
    lower = lower_ci,
    upper = upper_ci,
    labeltext = c(Outcome, n_studies, es, lower_ci, upper_ci, p),
  zero = 1,
  vertices = TRUE,
  title = "A. Forest plot of MA Outcomes for Binary Outcomes",
  clip = c(-1.5, 1.5),
  xlab = "RR [95% CI]",
  graph.pos = 3
) %>% fp_set_style(
    box = c("black"),
    line = "black",
    txt_gp = fpTxtGp(
      ticks = gpar(fontfamily = "", cex = 1),
      xlab  = gpar(fontfamily = "", cex = 1)
    )) %>% fp_add_lines("steelblue") %>%
  fp_add_header("Outcome",
                "Studies, n",
                  "RR",
                "[ 95%", " CI ]",
                "P-Value")  %>% fp_decorate_graph(box = TRUE) %>% fp_set_zebra_style("#EFEFEF")
binary_plot
```

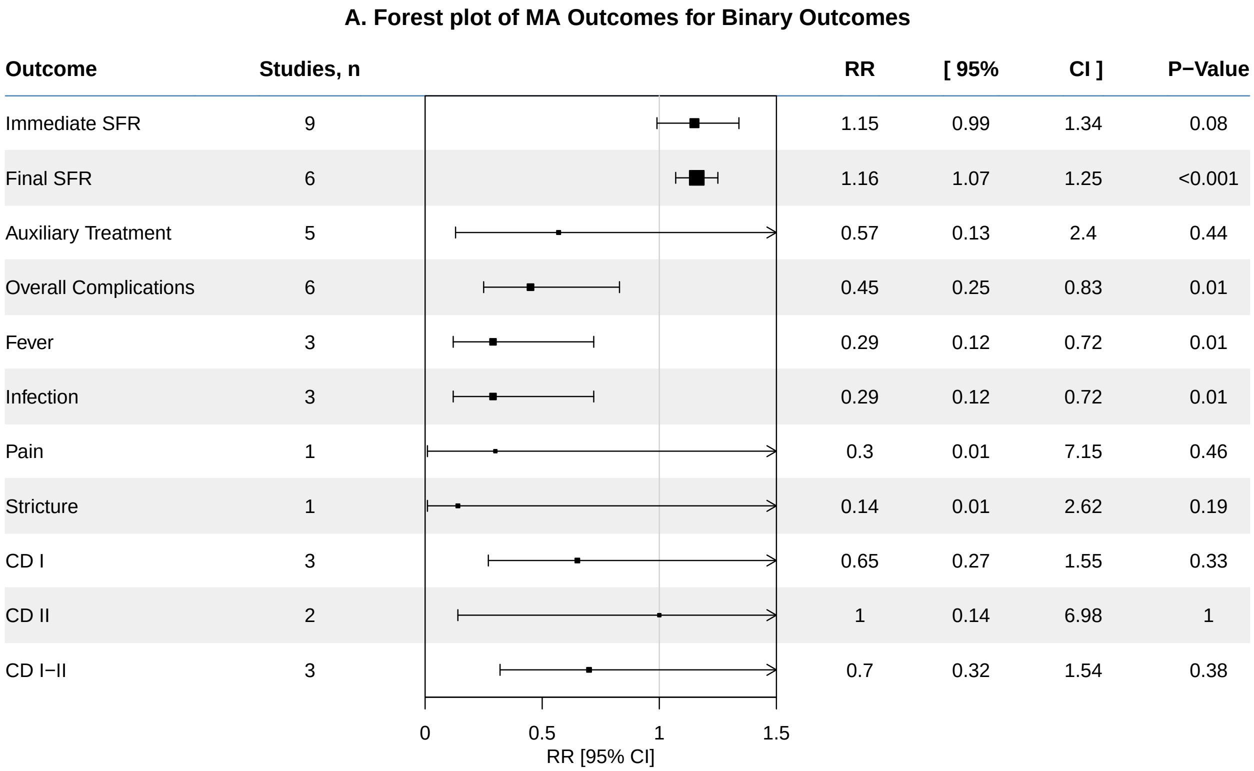

10 Flexi URS Only Meta-Analysis Outcomes

```
flexiurs_only <- suction_data %>% subset(control_group == "Flexible URS")
```

10.1 Immediate SFR

10.1.1 Meta-analysis

```
immediate_sfr_metabin <- metabin(data = suction_data,
                         event.c = sfr_immediate_n_control,
                       n.c = sample_size_control,
                       event.e = sfr_immediate_n_suction,
                       n.e = sample_size_suction,
                       studlab = author_year)
immediate_sfr_metabin
```

```
## Number of studies: k = 9
## Number of observations: o = 2311
## Number of events: e = 979
##
##                          RR           95%-CI    z  p-value
## Common effect model  1.1581 [1.0834; 1.2380] 4.31 < 0.0001
## Random effects model 1.1478 [0.9854; 1.3370] 1.77   0.0765
##
## Quantifying heterogeneity:
##  tau^2 = 0.0365 [0.0088; 0.2318]; tau = 0.1910 [0.0939; 0.4814]
##  I^2 = 74.6% [50.9%; 86.9%]; H = 1.99 [1.43; 2.76]
##
## Test of heterogeneity:
##      Q d.f. p-value
##  31.55    8  0.0001
##
## Details on meta-analytical method:
## - Mantel-Haenszel method
## - Inverse variance method
## - Restricted maximum-likelihood estimator for tau^2
## - Q-Profile method for confidence interval of tau^2 and tau
```

10.1.2 Forest plot

```r
forest(immediate_sfr_metabin,
       sortvar = TE)
```

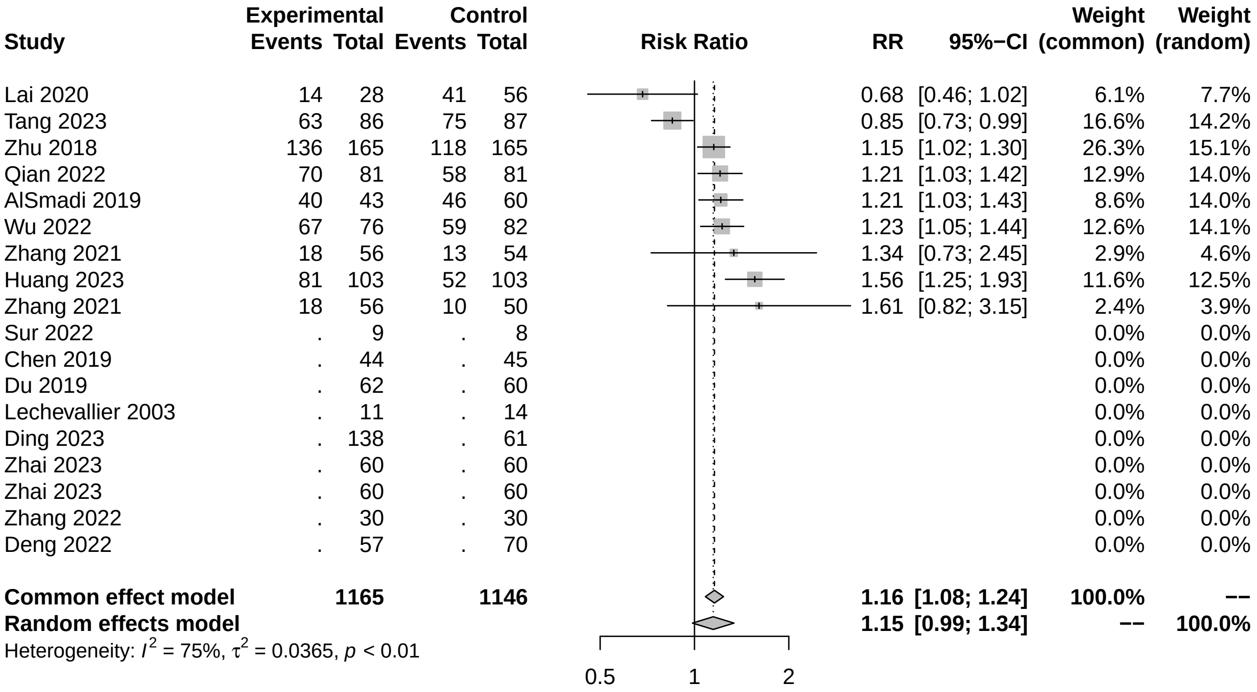

10.1.3 Trim and Fill

```
trimfill(immediate_sfr_metabin)
```

```
## Warning in trimfill.meta(immediate_sfr_metabin): 9 observation(s) dropped due
## to missing values

## Number of studies: k = 11 (with 2 added studies)
## Number of observations: o = 1744
## Number of events: e = 1140
##
##                              RR           95%-CI    z p-value
## Random effects model 1.0855 [0.9289; 1.2685] 1.03  0.3019
##
## Quantifying heterogeneity:
##  tau^2 = 0.0471 [0.0138; 0.2196]; tau = 0.2170 [0.1173; 0.4686]
##  I^2 = 76.9% [58.8%; 87.1%]; H = 2.08 [1.56; 2.78]
##
## Test of heterogeneity:
##      Q d.f.  p-value
##  43.33   10 < 0.0001
##
## Details on meta-analytical method:
## - Inverse variance method
## - Restricted maximum-likelihood estimator for tau^2
## - Q-Profile method for confidence interval of tau^2 and tau
## - Trim-and-fill method to adjust for funnel plot asymmetry (L-estimator)
```

```
forest(trimfill(immediate_sfr_metabin),
       sortvar = TE)
```

```
## Warning in trimfill.meta(immediate_sfr_metabin): 9 observation(s) dropped due
## to missing values
```

Study	logRR	SE(logRR)	Experimental Total	Control Total	RR	95%−CI	Weight
Lai 2020	−0.3814	0.2055	28	56	0.68	[0.46; 1.02]	7.1%
Filled: Zhang 2021	−0.2721	0.3431	56	50	0.76	[0.39; 1.49]	3.8%
Filled: Huang 2023	−0.2408	0.1103	103	103	0.79	[0.63; 0.98]	10.7%
Tang 2023	−0.1628	0.0780	86	87	0.85	[0.73; 0.99]	11.9%
Zhu 2018	0.1420	0.0609	165	165	1.15	[1.02; 1.30]	12.4%
Qian 2022	0.1881	0.0827	81	81	1.21	[1.03; 1.42]	11.7%
AlSmadi 2019	0.1934	0.0826	43	60	1.21	[1.03; 1.43]	11.7%
Wu 2022	0.2031	0.0808	76	82	1.23	[1.05; 1.44]	11.8%
Zhang 2021	0.2891	0.3100	56	54	1.34	[0.73; 2.45]	4.4%
Huang 2023	0.4432	0.1103	103	103	1.56	[1.25; 1.93]	10.7%
Zhang 2021	0.4745	0.3431	56	50	1.61	[0.82; 3.15]	3.8%
**Random effects model**			**853**	**891**	**1.09**	**[0.93; 1.27]**	**100.0%**

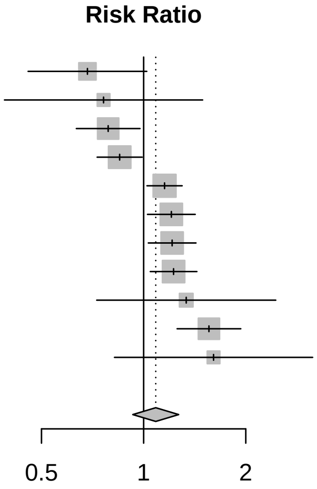

Heterogeneity: $I^2 = 77\%$, $\tau^2 = 0.0471$, $p < 0.01$

10.1.4 Funnel plot

```
funnel(trimfill(immediate_sfr_metabin))
```

```
## Warning in trimfill.meta(immediate_sfr_metabin): 9 observation(s) dropped due
## to missing values
```

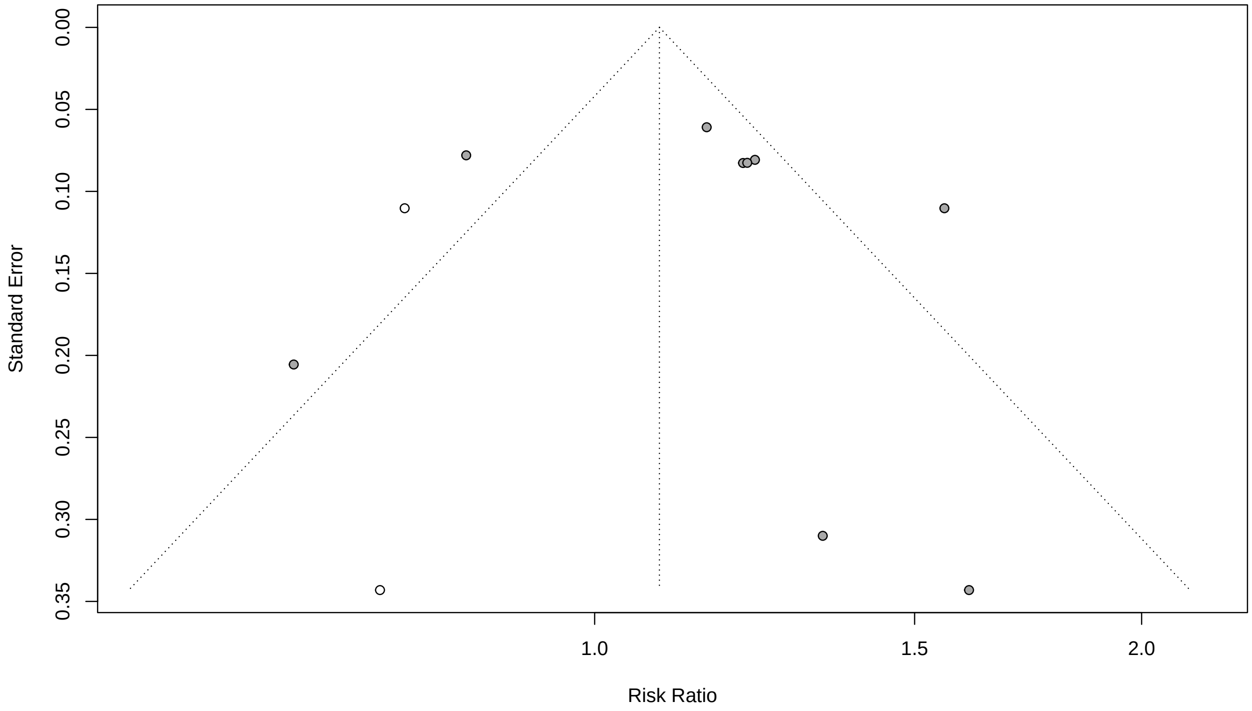

10.1.5 Baujat

```
baujat(immediate_sfr_metabin, pos = 1)
```

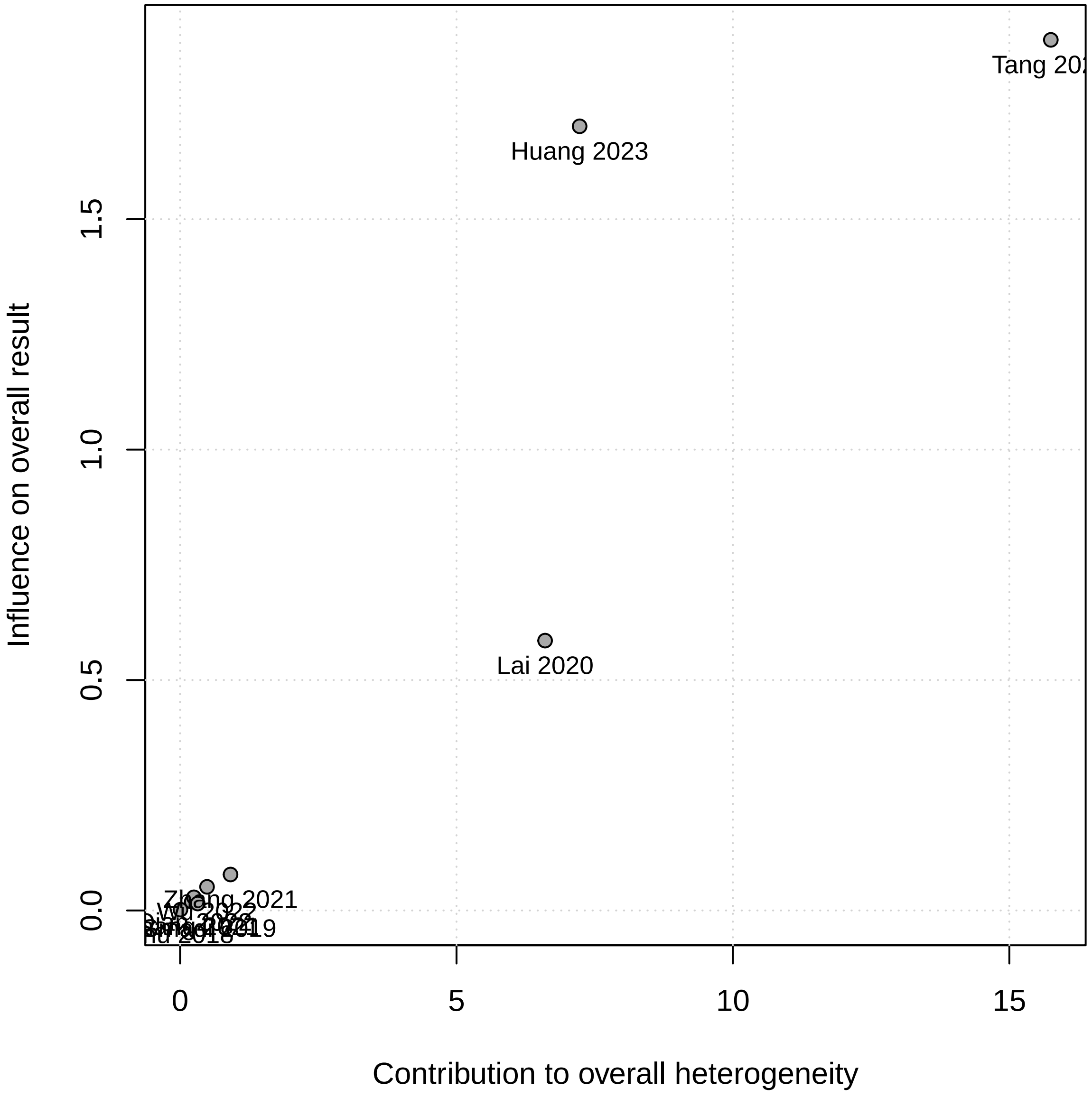

10.1.6 Leave one out

```
metainf(immediate_sfr_metabin)
```

```
## Influential analysis (common effect model)
##
##                                     RR           95%-CI  p-value   tau^2    tau
## Omitting Sur 2022               1.1581 [1.0834; 1.2380] < 0.0001  0.0365  0.1910
## Omitting Tang 2023              1.2195 [1.1329; 1.3128] < 0.0001  0.0076  0.0873
## Omitting Chen 2019              1.1581 [1.0834; 1.2380] < 0.0001  0.0365  0.1910
## Omitting Du 2019                1.1581 [1.0834; 1.2380] < 0.0001  0.0365  0.1910
## Omitting Lai 2020               1.1889 [1.1116; 1.2716] < 0.0001  0.0258  0.1607
## Omitting Zhu 2018               1.1601 [1.0710; 1.2565]   0.0003  0.0482  0.2196
## Omitting Zhang 2021             1.1473 [1.0741; 1.2254] < 0.0001  0.0376  0.1938
## Omitting Zhang 2021             1.1527 [1.0791; 1.2313] < 0.0001  0.0396  0.1990
## Omitting Lechevallier 2003      1.1581 [1.0834; 1.2380] < 0.0001  0.0365  0.1910
## Omitting Huang 2023             1.1057 [1.0315; 1.1853]   0.0046  0.0246  0.1568
## Omitting Wu 2022                1.1484 [1.0676; 1.2353]   0.0002  0.0464  0.2153
## Omitting Ding 2023              1.1581 [1.0834; 1.2380] < 0.0001  0.0365  0.1910
## Omitting Zhai 2023              1.1581 [1.0834; 1.2380] < 0.0001  0.0365  0.1910
## Omitting Zhai 2023              1.1581 [1.0834; 1.2380] < 0.0001  0.0365  0.1910
## Omitting Qian 2022              1.1508 [1.0700; 1.2377]   0.0002  0.0468  0.2164
## Omitting Zhang 2022             1.1581 [1.0834; 1.2380] < 0.0001  0.0365  0.1910
## Omitting Deng 2022              1.1581 [1.0834; 1.2380] < 0.0001  0.0365  0.1910
## Omitting AlSmadi 2019           1.1529 [1.0734; 1.2383] < 0.0001  0.0467  0.2160
##
## Pooled estimate                 1.1581 [1.0834; 1.2380] < 0.0001  0.0365  0.1910
##                                    I^2
## Omitting Sur 2022                74.6%
## Omitting Tang 2023               51.5%
## Omitting Chen 2019               74.6%
## Omitting Du 2019                 74.6%
## Omitting Lai 2020                72.2%
## Omitting Zhu 2018                77.8%
## Omitting Zhang 2021              77.1%
## Omitting Zhang 2021              77.6%
## Omitting Lechevallier 2003       74.6%
## Omitting Huang 2023              69.4%
## Omitting Wu 2022                 77.2%
## Omitting Ding 2023               74.6%
## Omitting Zhai 2023               74.6%
## Omitting Zhai 2023               74.6%
## Omitting Qian 2022               77.4%
## Omitting Zhang 2022              74.6%
## Omitting Deng 2022               74.6%
## Omitting AlSmadi 2019            77.4%
##
## Pooled estimate                  74.6%
##
## Details on meta-analytical method:
## - Mantel-Haenszel method
## - Restricted maximum-likelihood estimator for tau^2
```

10.2 Final SFR

10.2.1 Meta-analysis

```
sfr_metabin <- metabin(data = flexiurs_only,
                         event.c = sfr_final_n_control,
                       n.c = sample_size_control,
                       event.e = sfr_final_n_suction,
                       n.e = sample_size_suction,
                       studlab = author_year)
sfr_metabin
```

```
## Number of studies: k = 7
## Number of observations: o = 1084
## Number of events: e = 918
##
##                          RR           95%-CI    z  p-value
## Common effect model  1.1937 [1.1280; 1.2633] 6.13 < 0.0001
## Random effects model 1.1956 [1.1008; 1.2986] 4.24 < 0.0001
##
## Quantifying heterogeneity:
##  tau^2 = 0.0056 [0.0000; 0.0491]; tau = 0.0746 [0.0000; 0.2216]
##  I^2 = 45.7% [0.0%; 77.1%]; H = 1.36 [1.00; 2.09]
##
## Test of heterogeneity:
##      Q d.f. p-value
##  11.05    6  0.0869
##
## Details on meta-analytical method:
## - Mantel-Haenszel method
## - Inverse variance method
## - Restricted maximum-likelihood estimator for tau^2
## - Q-Profile method for confidence interval of tau^2 and tau
## - Continuity correction of 0.5 in studies with zero cell frequencies
```

10.2.2 Forest plot

```r
forest(sfr_metabin,
       sortvar = TE)
```

Study	Experimental Events	Experimental Total	Control Events	Control Total	RR	95%−CI	Weight (common)	Weight (random)
Qian 2022	72	81	67	81	1.07	[0.95; 1.22]	16.6%	18.3%
Zhu 2018	143	165	131	165	1.09	[0.99; 1.20]	32.5%	22.0%
Zhang 2022	27	30	23	30	1.17	[0.93; 1.48]	5.7%	9.2%
Zhang 2021	54	56	42	54	1.24	[1.07; 1.44]	10.6%	15.4%
Huang 2023	97	103	78	103	1.24	[1.10; 1.40]	19.4%	19.2%
Sur 2022	9	9	6	8	1.31	[0.90; 1.90]	1.7%	4.3%
Ding 2023	130	138	39	61	1.47	[1.21; 1.79]	13.4%	11.6%
**Common effect model**		**582**		**502**	**1.19**	**[1.13; 1.26]**	**100.0%**	**−−**
**Random effects model**					**1.20**	**[1.10; 1.30]**	**−−**	**100.0%**

Heterogeneity: $I^2 = 46\%$, $\tau^2 = 0.0056$, $p = 0.09$

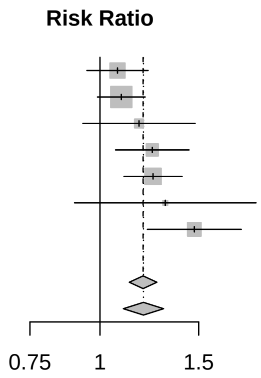

10.2.3 Trim and Fill

```r
trimfill(sfr_metabin)
```

```
## Number of studies: k = 10 (with 3 added studies)
## Number of observations: o = 1506
## Number of events: e = 1277
##
##                           RR           95%-CI    z p-value
## Random effects model 1.1277 [1.0267; 1.2385] 2.51  0.0120
##
## Quantifying heterogeneity:
##  tau^2 = 0.0141 [0.0024; 0.0736]; tau = 0.1188 [0.0486; 0.2713]
##  I^2 = 64.4% [29.9%; 81.9%]; H = 1.68 [1.19; 2.35]
##
## Test of heterogeneity:
##      Q d.f. p-value
##  25.27    9  0.0027
##
## Details on meta-analytical method:
## - Inverse variance method
## - Restricted maximum-likelihood estimator for tau^2
## - Q-Profile method for confidence interval of tau^2 and tau
## - Trim-and-fill method to adjust for funnel plot asymmetry (L-estimator)
```

```r
forest(trimfill(sfr_metabin),
       sortvar = TE)
```

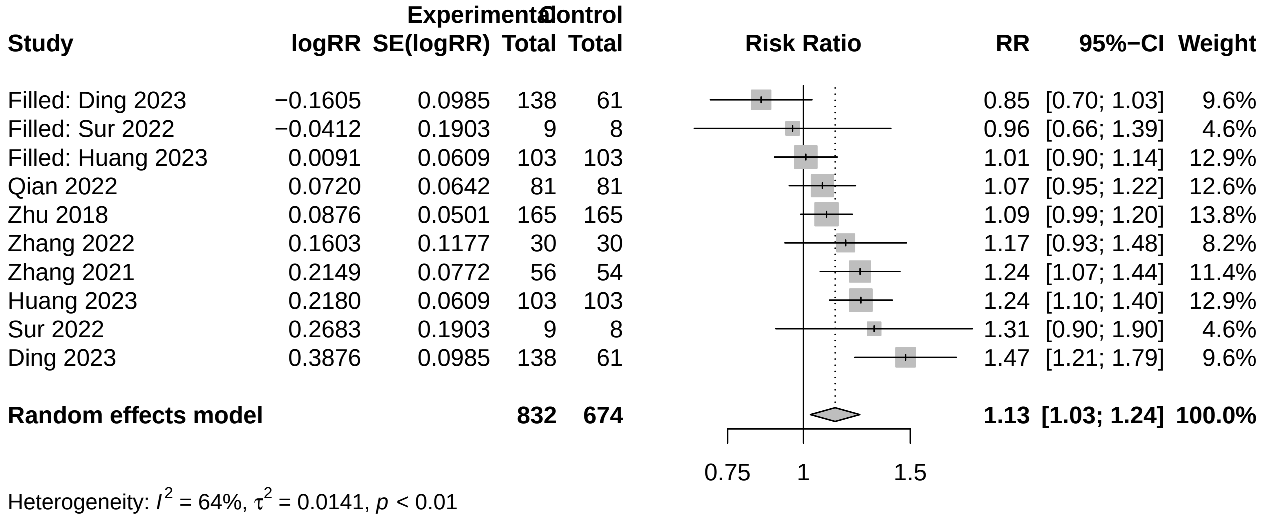

10.2.4 Funnel plot

```
funnel(trimfill(sfr_metabin))
```

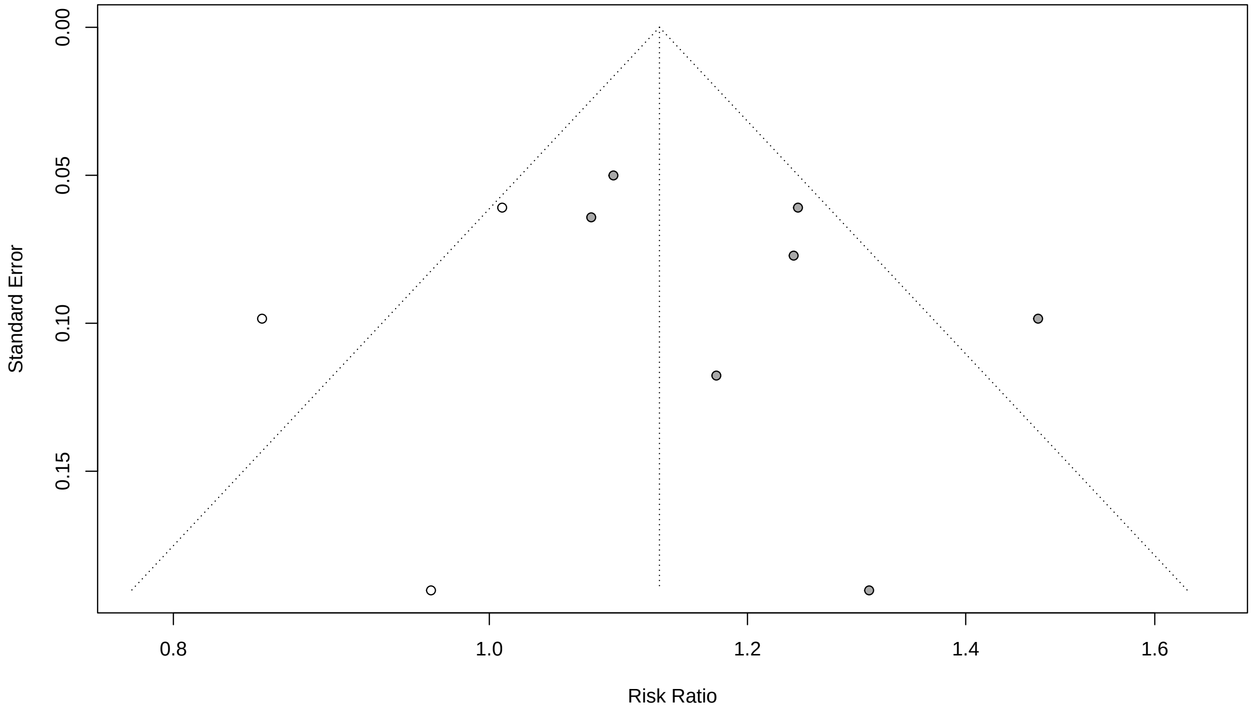

10.2.5 Baujat

```
baujat(sfr_metabin, pos = 1)
```

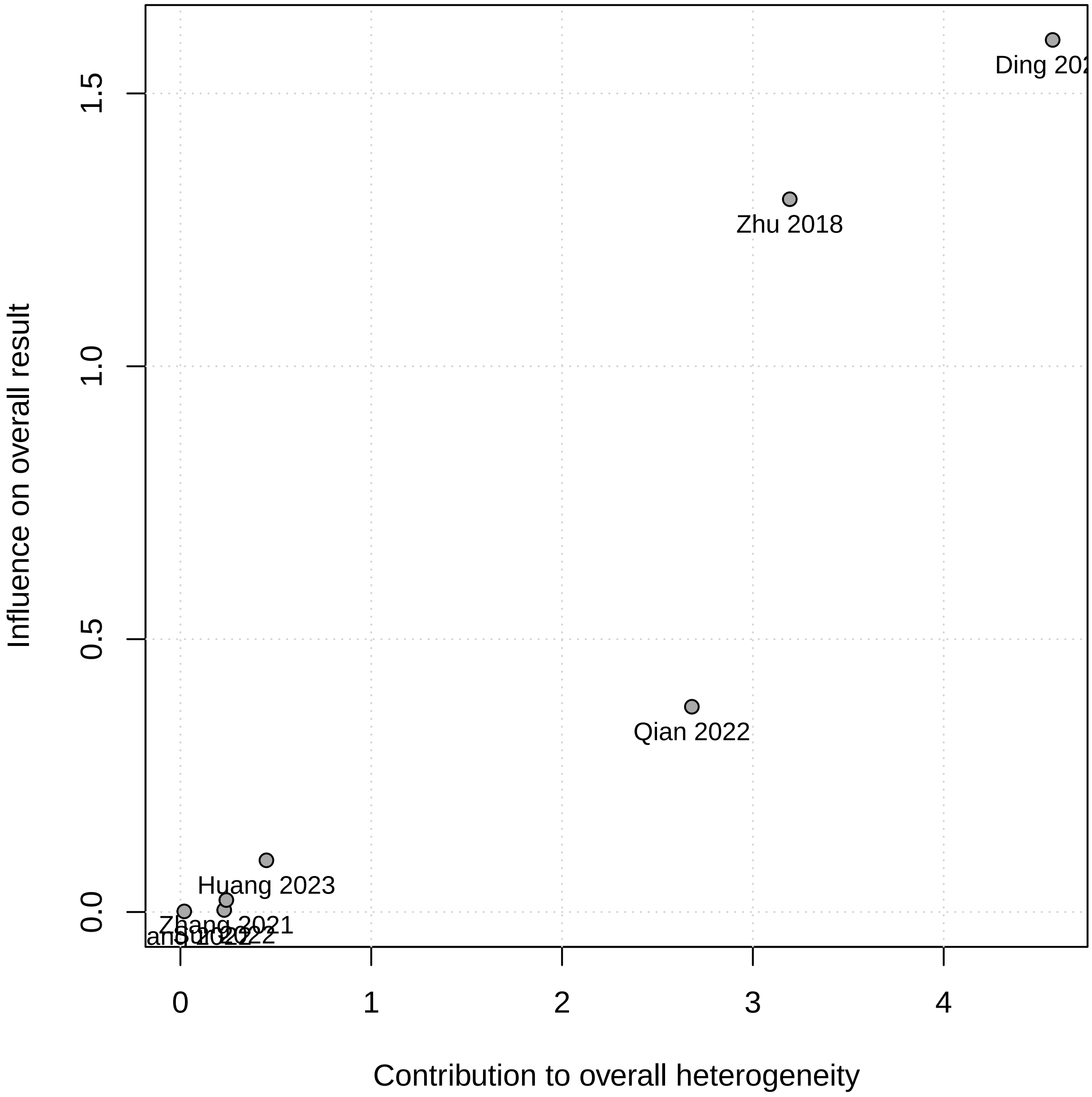

10.2.6 Leave one out

```r
metainf(sfr_metabin)
```

```
## Influential analysis (common effect model)
##
##                            RR           95%-CI  p-value   tau^2    tau    I^2
## Omitting Sur 2022      1.1916 [1.1254; 1.2617] < 0.0001  0.0060 0.0774  53.4%
## Omitting Zhu 2018      1.2430 [1.1597; 1.3322] < 0.0001  0.0052 0.0719  37.0%
## Omitting Zhang 2021    1.1883 [1.1182; 1.2628] < 0.0001  0.0076 0.0872  52.3%
## Omitting Huang 2023    1.1818 [1.1083; 1.2601] < 0.0001  0.0077 0.0880  49.7%
## Omitting Ding 2023     1.1503 [1.0862; 1.2183] < 0.0001  0.0016 0.0395   5.3%
## Omitting Qian 2022     1.2175 [1.1432; 1.2967] < 0.0001  0.0054 0.0733  42.4%
## Omitting Zhang 2022    1.1949 [1.1271; 1.2669] < 0.0001  0.0073 0.0852  54.7%
##
## Pooled estimate        1.1937 [1.1280; 1.2633] < 0.0001  0.0056 0.0746  45.7%
##
## Details on meta-analytical method:
## - Mantel-Haenszel method
## - Restricted maximum-likelihood estimator for tau^2
```

10.3 OR time

10.3.1 Meta-analysis

```
or_time_metacont <- metacont(data = flexiurs_only,
                             mean.c = or_time_min_mean_control,
                             sd.c = or_time_min_sd_control,
                             mean.e = or_time_min_mean_suction,
                             sd.e = or_time_min_sd_suction,
                             n.e = sample_size_suction,
                             n.c = sample_size_control,
                       studlab = author_year)
or_time_metacont
```

```
## Number of studies: k = 6
## Number of observations: o = 1084
##
##                            MD             95%-CI     z  p-value
## Common effect model  -4.3519 [-6.3102; -2.3937] -4.36 < 0.0001
## Random effects model -4.3074 [-8.6117; -0.0031] -1.96   0.0498
##
## Quantifying heterogeneity:
##  tau^2 = 17.2246 [2.8614; >172.2459]; tau = 4.1503 [1.6916; >13.1242]
##  I^2 = 73.6% [39.5%; 88.4%]; H = 1.94 [1.29; 2.94]
##
## Test of heterogeneity:
##      Q d.f. p-value
##  18.91    5  0.0020
##
## Details on meta-analytical method:
## - Inverse variance method
## - Restricted maximum-likelihood estimator for tau^2
## - Q-Profile method for confidence interval of tau^2 and tau
```

10.3.2 Forest plot

```
forest(or_time_metacont,
       sortvar = TE)
```

	Experimental			Control							
**Study**	**Total**	**Mean**	**SD**	**Total**	**Mean**	**SD**	**Mean Difference**	**MD**	**95%−CI**	**Weight (common)**	**Weight (random)**
Zhang 2021	56	46.40	17.9000	54	57.30	28.5000		−10.90	[−19.83; −1.97]	4.8%	12.7%
Zhu 2018	165	49.70	16.3000	165	57.00	14.0000		−7.30	[−10.58; −4.02]	35.7%	24.1%
Qian 2022	81	72.90	28.1000	81	80.00	29.5000		−7.10	[−15.97; 1.77]	4.9%	12.8%
Zhang 2022	30	57.30	10.6000	30	62.70	1.2000		−5.40	[−9.22; −1.58]	26.3%	22.9%
Ding 2023	138	42.00	13.2000	61	40.60	12.1000		1.40	[−2.35; 5.15]	27.3%	23.1%
Sur 2022	9	54.00	17.0000	8	39.00	22.0000		15.00	[−3.86; 33.86]	1.1%	4.4%
Huang 2023	103	.	.	103	.	.				0.0%	0.0%
**Common effect model**	**582**			**502**				**−4.35**	**[−6.31; −2.39]**	**100.0%**	**−−**
**Random effects model**								**−4.31**	**[−8.61; −0.00]**	**−−**	**100.0%**

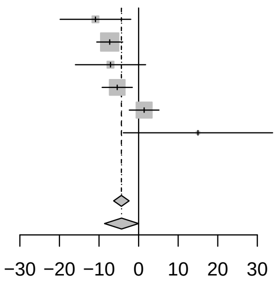

Heterogeneity: $I^2 = 74\%$, $\tau^2 = 17.2246$, $p < 0.01$

10.3.3 Trim and Fill

```r
trimfill(or_time_metacont)
```

```
## Warning in trimfill.meta(or_time_metacont): 1 observation(s) dropped due to
## missing values

## Number of studies: k = 6 (with 0 added studies)
## Number of observations: o = 878
##
##                             MD             95%-CI     z p-value
## Random effects model -4.3074 [-8.6117; -0.0031] -1.96  0.0498
##
## Quantifying heterogeneity:
##  tau^2 = 17.2246 [2.8614; >172.2459]; tau = 4.1503 [1.6916; >13.1242]
##  I^2 = 73.6% [39.5%; 88.4%]; H = 1.94 [1.29; 2.94]
##
## Test of heterogeneity:
##      Q d.f. p-value
##  18.91    5  0.0020
##
## Details on meta-analytical method:
## - Inverse variance method
## - Restricted maximum-likelihood estimator for tau^2
## - Q-Profile method for confidence interval of tau^2 and tau
## - Trim-and-fill method to adjust for funnel plot asymmetry (L-estimator)
```

```r
forest(trimfill(or_time_metacont),
       sortvar = TE)
```

```
## Warning in trimfill.meta(or_time_metacont): 1 observation(s) dropped due to
## missing values
```

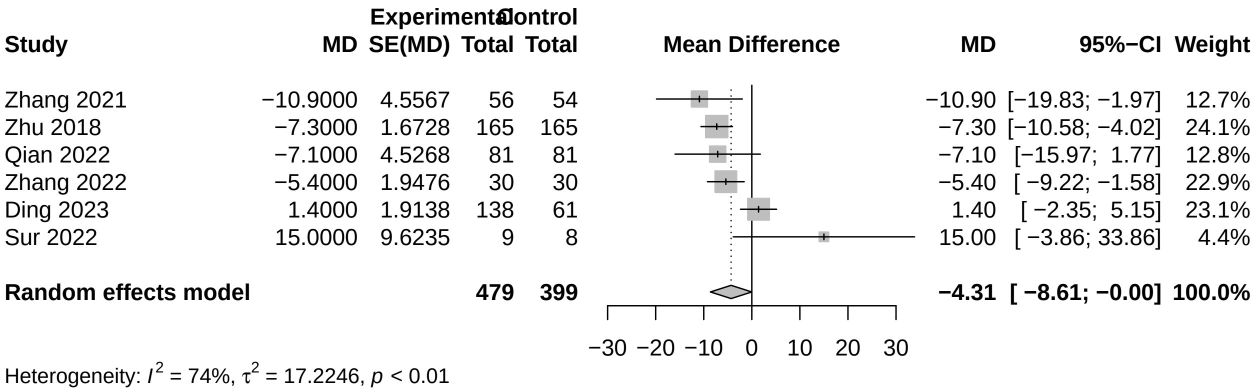

10.3.4 Funnel plot

```
funnel(trimfill(or_time_metacont))
```

```
## Warning in trimfill.meta(or_time_metacont): 1 observation(s) dropped due to
## missing values
```

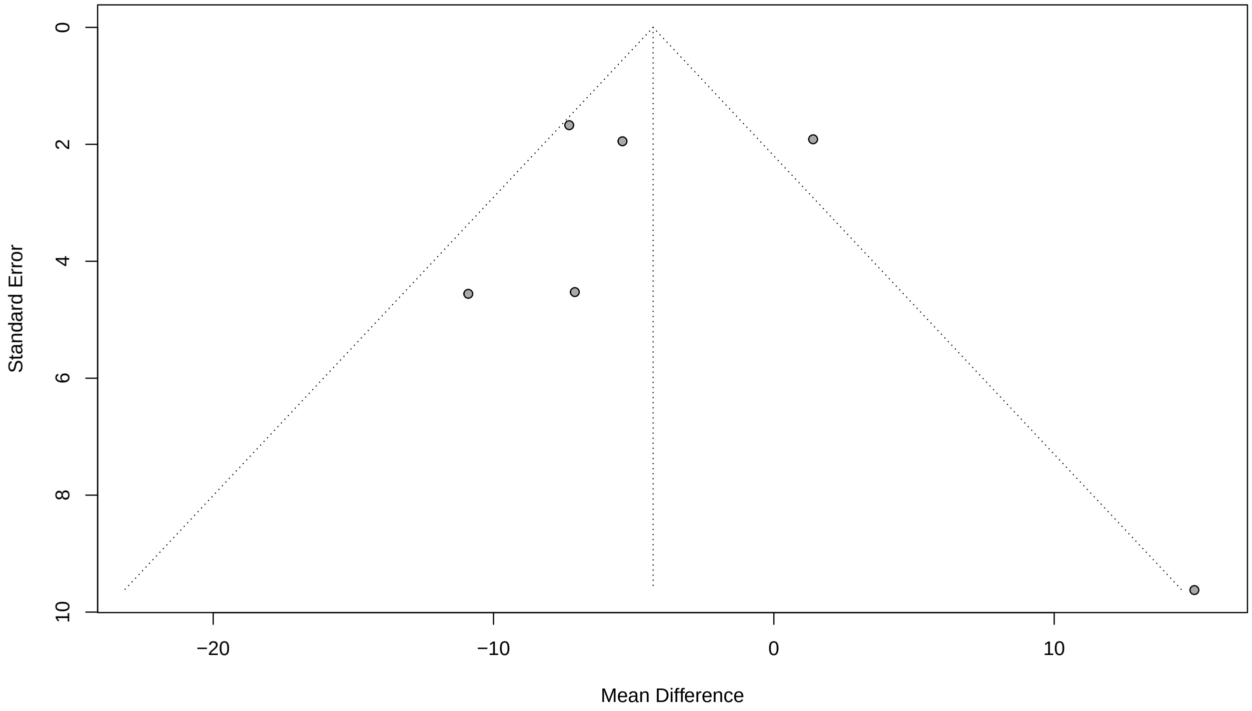

10.3.5 Baujat

```
baujat(or_time_metacont, pos = 1)
```

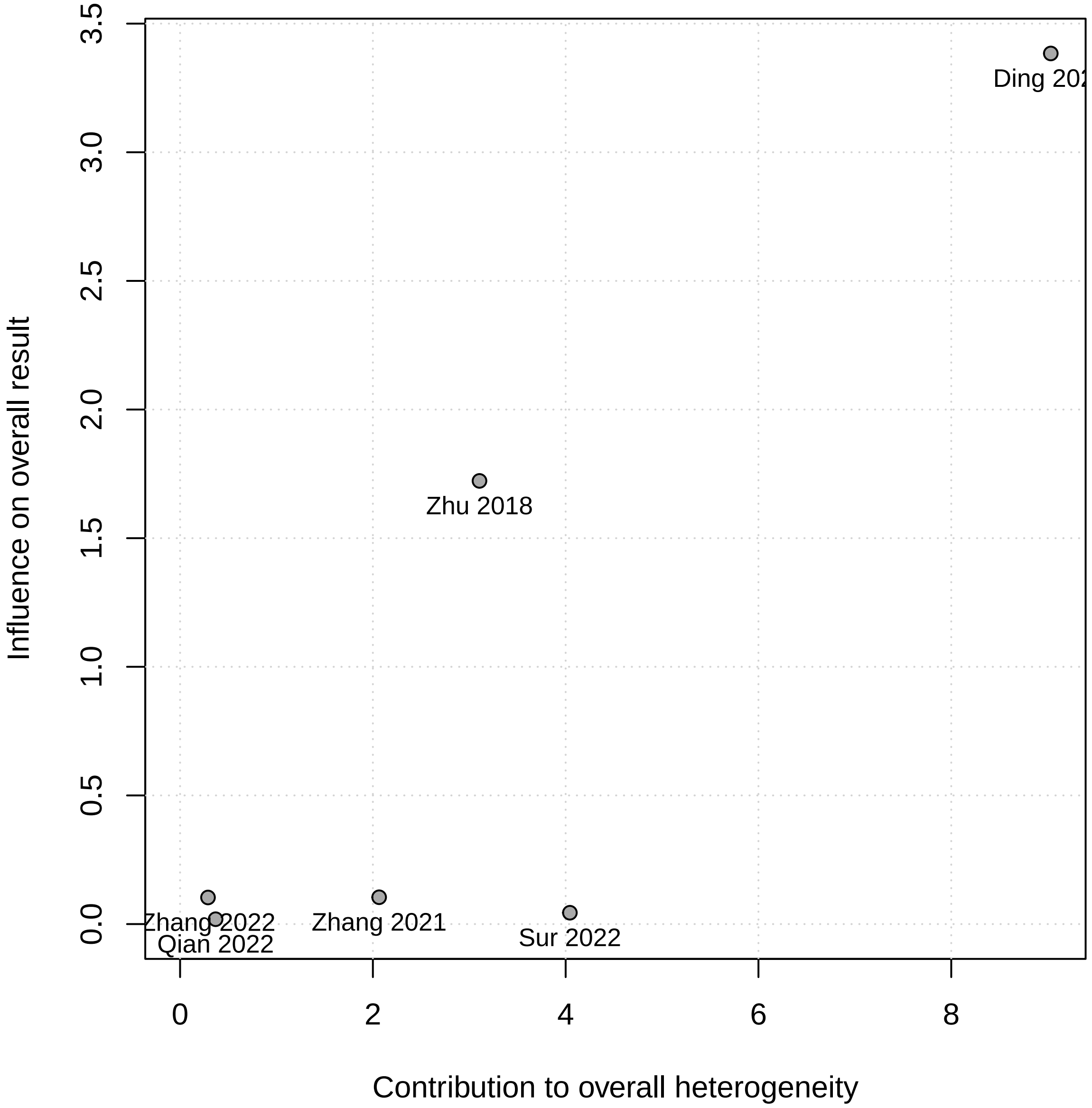

10.3.6 Leave one out

```
metainf(or_time_metacont)
```

```
## Influential analysis (common effect model)
##
##                            MD            95%-CI  p-value   tau^2    tau
## Omitting Sur 2022     -4.5628 [-6.5317; -2.5939] < 0.0001  14.4616  3.8028
## Omitting Zhu 2018     -2.7170 [-5.1585; -0.2754]   0.0292  23.5074  4.8484
## Omitting Zhang 2021   -4.0212 [-6.0283; -2.0142] < 0.0001  17.2725  4.1560
## Omitting Huang 2023   -4.3519 [-6.3102; -2.3937] < 0.0001  17.2246  4.1503
## Omitting Ding 2023    -6.5068 [-8.8027; -4.2109] < 0.0001   0.0000  0.0025
## Omitting Qian 2022    -4.2112 [-6.2190; -2.2035] < 0.0001  23.6032  4.8583
## Omitting Zhang 2022   -3.9777 [-6.2589; -1.6964]   0.0006  29.5130  5.4326
##
## Pooled estimate       -4.3519 [-6.3102; -2.3937] < 0.0001  17.2246  4.1503
##                         I^2
## Omitting Sur 2022      73.0%
## Omitting Zhu 2018      71.6%
## Omitting Zhang 2021    76.1%
## Omitting Huang 2023    73.6%
## Omitting Ding 2023     38.4%
## Omitting Qian 2022     78.4%
## Omitting Zhang 2022    78.4%
##
## Pooled estimate        73.6%
##
## Details on meta-analytical method:
## - Inverse variance method
## - Restricted maximum-likelihood estimator for tau^2
```

10.4 Auxiliary Treatments

10.4.1 Meta-analysis

```
aux_rx_metabin <- metabin(data = flexiurs_only,
                         event.c = auxillary_tx_n_control,
                       n.c = sample_size_control,
                       event.e = auxillary_tx_n_suction,
                       n.e = sample_size_suction,
                       studlab = author_year)
aux_rx_metabin
```

```
## Number of studies: k = 1
## Number of observations: o = 1084
## Number of events: e = 1
##
##                          RR           95%-CI     z p-value
## Common effect model  0.3333 [0.0137; 8.1235] -0.67  0.5001
## Random effects model 0.3333 [0.0137; 8.1233] -0.67  0.5001
##
## Quantifying heterogeneity:
##  tau^2 = NA; tau = NA; I^2 = NA; H = NA
##
## Details on meta-analytical method:
## - Mantel-Haenszel method
## - Inverse variance method
## - Restricted maximum-likelihood estimator for tau^2
## - Continuity correction of 0.5 in studies with zero cell frequencies
```

10.4.2 Forest plot

```r
forest(aux_rx_metabin,
       sortvar = TE)
```

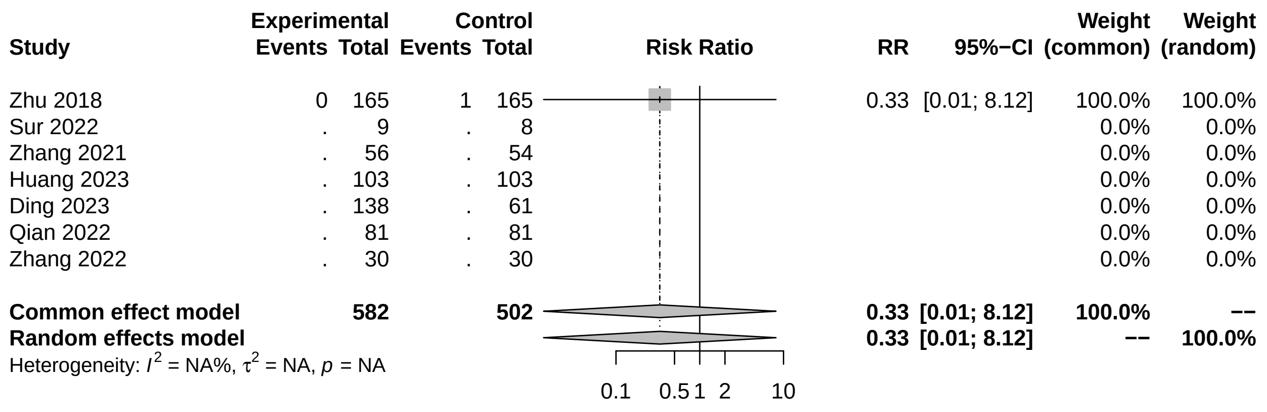

10.4.3 Trim and Fill

```
#trimfill(aux_rx_metabin)
#forest(trimfill(aux_rx_metabin),
 #      sortvar = TE)
```

10.4.4 Funnel plot

```r
#funnel(trimfill(aux_rx_metabin))
```

10.4.5 Baujat

```
#baujat(aux_rx_metabin, pos = 1)
```

10.4.6 Leave one out

```
#metainf(aux_rx_metabin)
```

10.5 VAS

10.5.1 Meta-analysis

```
vas_metacont <- metacont(data = flexiurs_only,
                             mean.c = vas_score_mean_control,
                             sd.c = vas_score_sd_control,
                             mean.e = vas_score_mean_suction,
                             sd.e = vas_score_sd_suction,
                             n.e = sample_size_suction,
                             n.c = sample_size_control,
                       studlab = author_year)
vas_metacont
```

```
## Number of studies: k = 1
## Number of observations: o = 1084
##
##                           MD             95%-CI     z p-value
## Common effect model  -0.3400 [-0.6488; -0.0312] -2.16  0.0309
## Random effects model -0.3400 [-0.6488; -0.0312] -2.16  0.0309
##
## Quantifying heterogeneity:
##  tau^2 = NA; tau = NA; I^2 = NA; H = NA
##
## Details on meta-analytical method:
## - Inverse variance method
## - Restricted maximum-likelihood estimator for tau^2
```

10.5.2 Forest plot

```r
forest(vas_metacont,
       sortvar = TE)
```

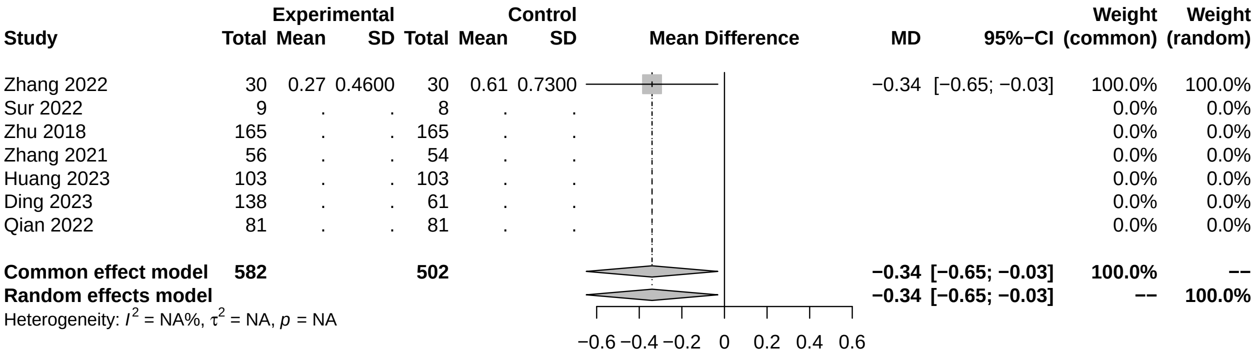

10.5.3 Trim and Fill

```r
#trimfill(vas_metacont)
#forest(trimfill(vas_metacont))
```

10.5.4 Funnel plot

```r
funnel((vas_metacont))
```

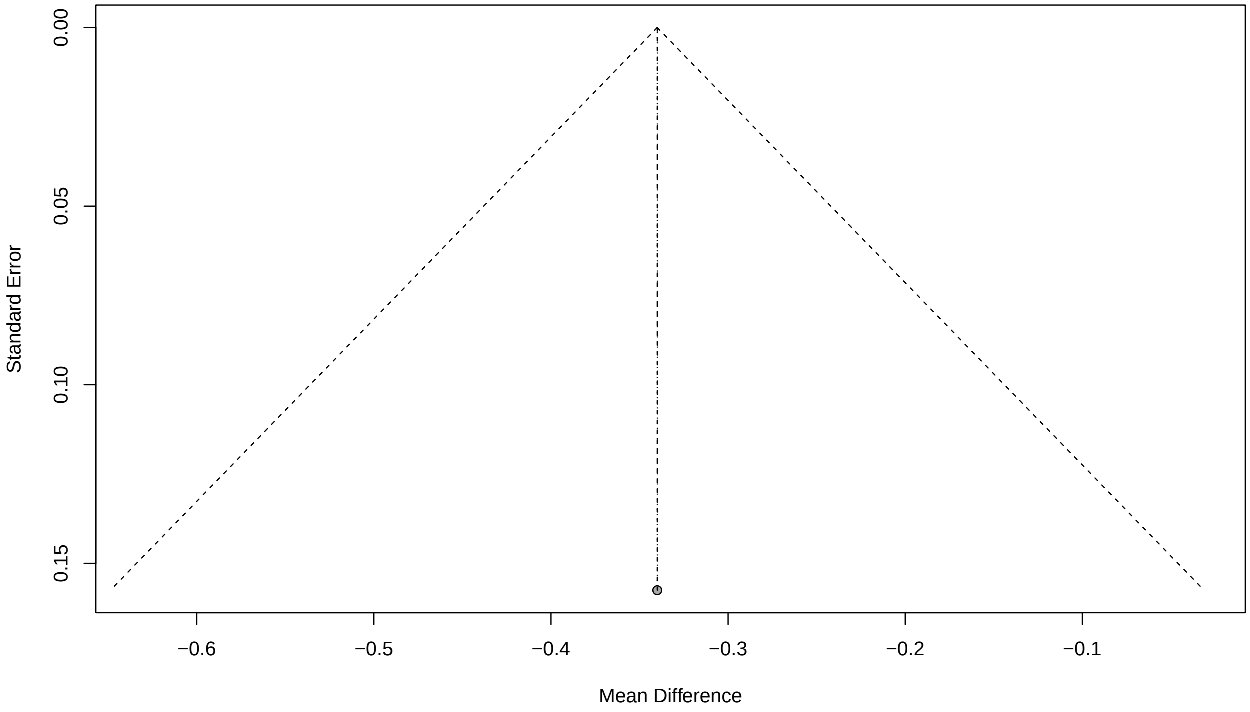

10.5.5 Baujat

```
baujat(vas_metacont, pos = 1)
```

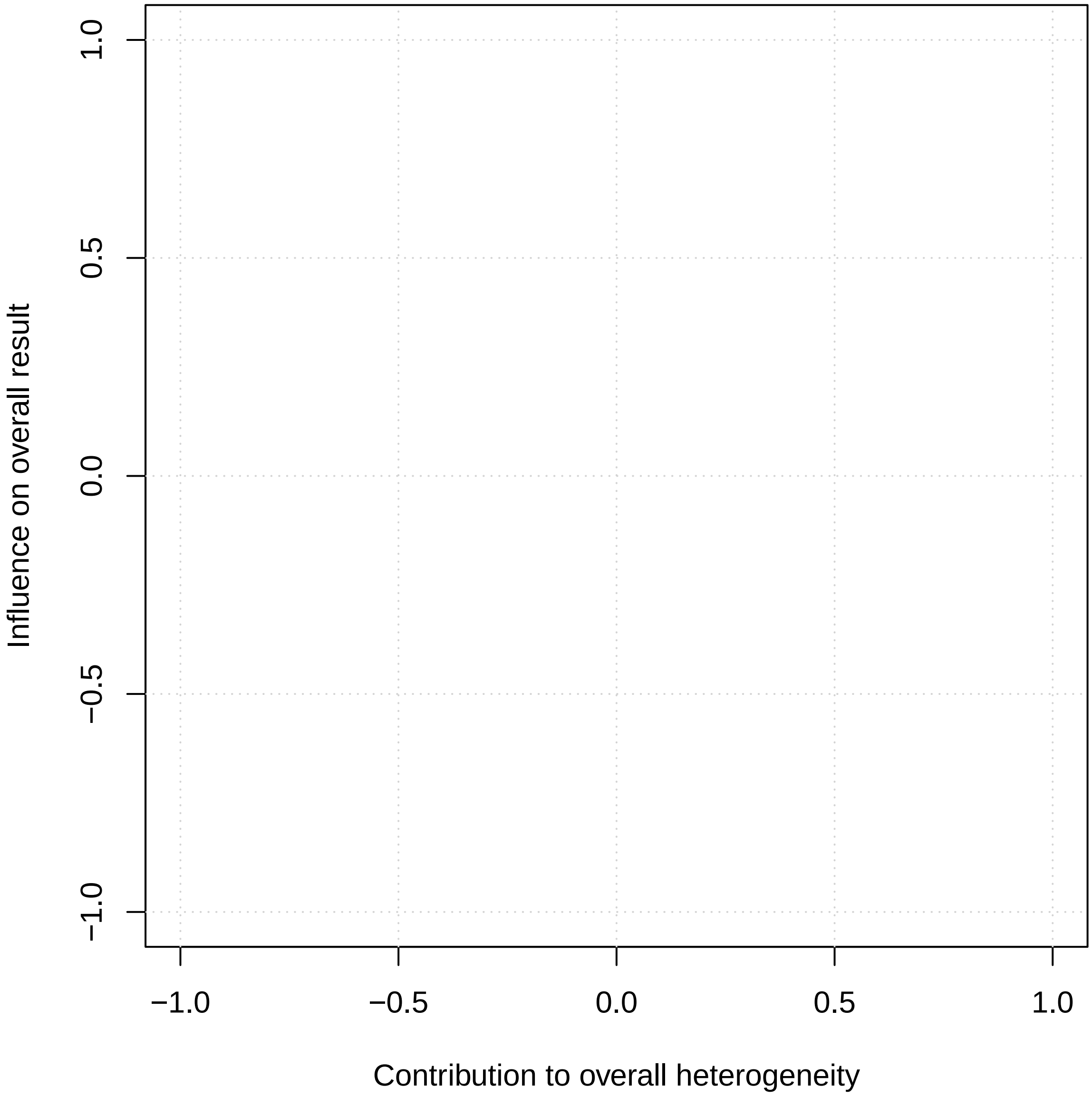

10.5.6 Leave one out

```
metainf(vas_metacont)
```

```
## Influential analysis (common effect model)
##
##                              MD             95%-CI p-value tau^2 tau I^2
## Omitting Sur 2022     -0.3400 [-0.6488; -0.0312]  0.0309
## Omitting Zhu 2018     -0.3400 [-0.6488; -0.0312]  0.0309
## Omitting Zhang 2021   -0.3400 [-0.6488; -0.0312]  0.0309
## Omitting Huang 2023   -0.3400 [-0.6488; -0.0312]  0.0309
## Omitting Ding 2023    -0.3400 [-0.6488; -0.0312]  0.0309
## Omitting Qian 2022    -0.3400 [-0.6488; -0.0312]  0.0309
## Omitting Zhang 2022
##
## Pooled estimate       -0.3400 [-0.6488; -0.0312]  0.0309
##
## Details on meta-analytical method:
## - Inverse variance method
## - Restricted maximum-likelihood estimator for tau^2
```

10.6 LoS

10.6.1 Meta-analysis

```
los_metacont <- metacont(data = flexiurs_only,
                             mean.c = los_days_mean_control,
                             sd.c = los_days_sd_control,
                             mean.e = los_days_mean_suction,
                             sd.e = los_days_sd_suction,
                             n.e = sample_size_suction,
                             n.c = sample_size_control,
                       studlab = author_year)
los_metacont
```

```
## Number of studies: k = 2
## Number of observations: o = 1084
##
##                              MD             95%-CI     z p-value
## Common effect model  -0.3113 [-0.5488; -0.0738] -2.57  0.0102
## Random effects model -0.4965 [-1.1744;  0.1814] -1.44  0.1512
##
## Quantifying heterogeneity:
##  tau^2 = 0.1901; tau = 0.4360; I^2 = 77.6% [2.1%; 94.9%]; H = 2.11 [1.01; 4.42]
##
## Test of heterogeneity:
##     Q d.f. p-value
##  4.46    1  0.0346
##
## Details on meta-analytical method:
## - Inverse variance method
## - Restricted maximum-likelihood estimator for tau^2
```

10.6.2 Forest plot

```
forest(los_metacont,
       sortvar = TE)
```

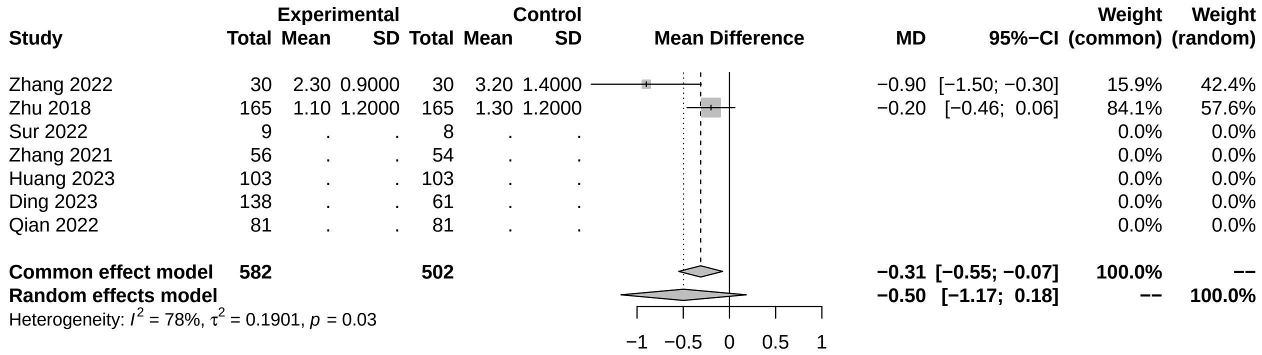

10.6.3 Trim and Fill

```
#trimfill(los_metacont)
#forest(trimfill(los_metacont),
#       sortvar = TE)
```

10.6.4 Funnel plot

```
funnel((los_metacont))
```

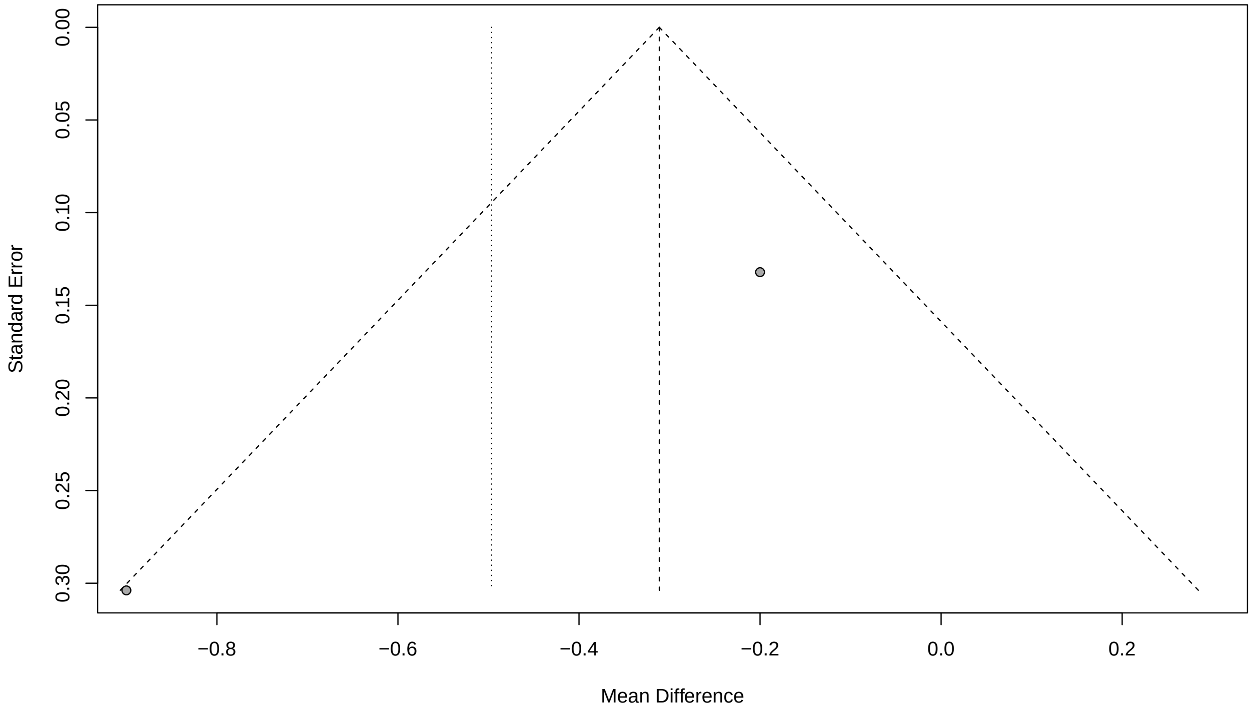

10.6.5 Baujat

```
baujat(los_metacont, pos = 1)
```

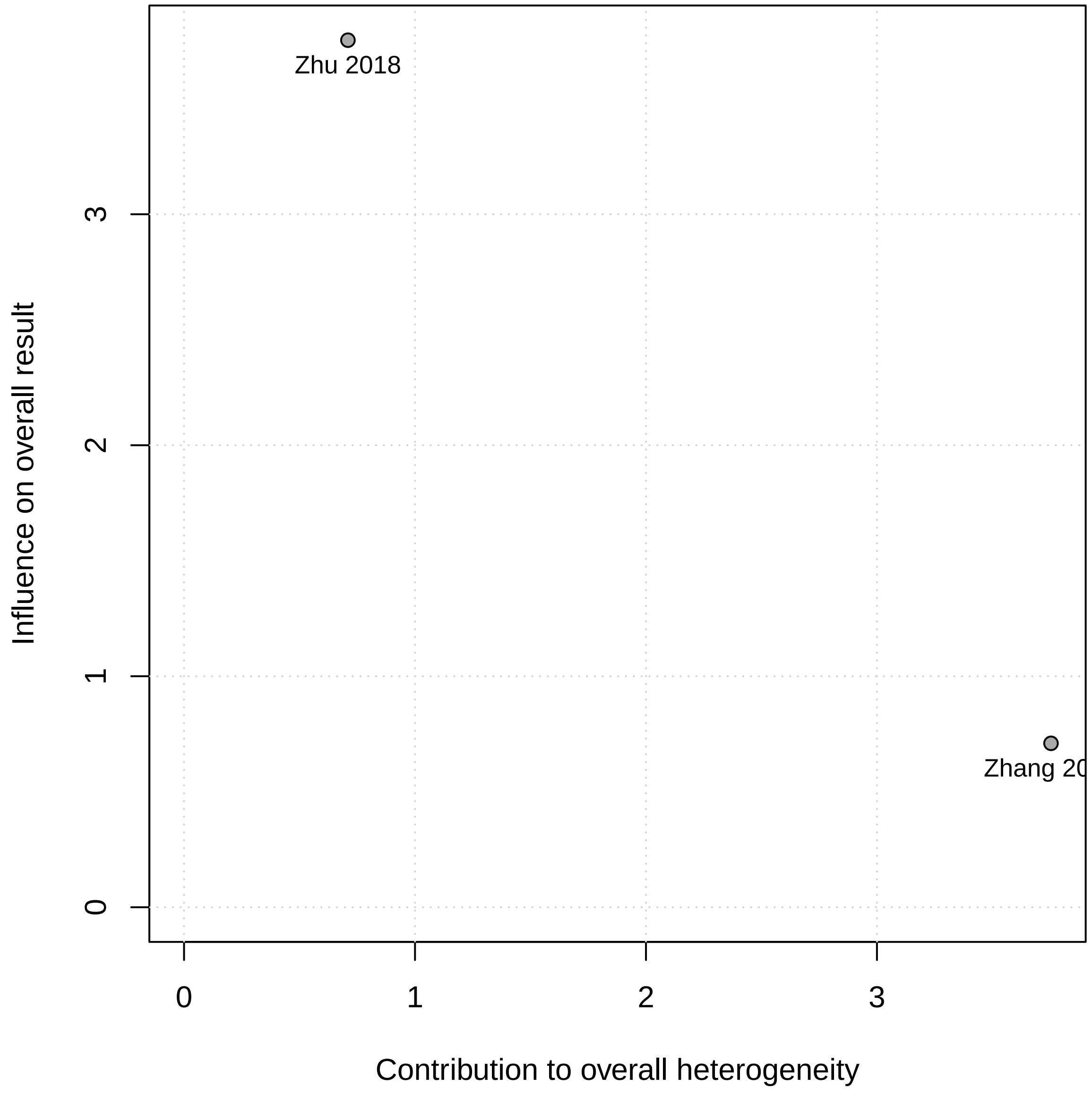

10.6.6 Leave one out

```
metainf(los_metacont)
```

```
## Influential analysis (common effect model)
##
##                              MD             95%-CI p-value   tau^2    tau    I^2
## Omitting Sur 2022     -0.3113 [-0.5488; -0.0738]  0.0102  0.1901  0.4360  77.6%
## Omitting Zhu 2018     -0.9000 [-1.4956; -0.3044]  0.0031
## Omitting Zhang 2021   -0.3113 [-0.5488; -0.0738]  0.0102  0.1901  0.4360  77.6%
## Omitting Huang 2023   -0.3113 [-0.5488; -0.0738]  0.0102  0.1901  0.4360  77.6%
## Omitting Ding 2023    -0.3113 [-0.5488; -0.0738]  0.0102  0.1901  0.4360  77.6%
## Omitting Qian 2022    -0.3113 [-0.5488; -0.0738]  0.0102  0.1901  0.4360  77.6%
## Omitting Zhang 2022   -0.2000 [-0.4589;  0.0589]  0.1301
##
## Pooled estimate       -0.3113 [-0.5488; -0.0738]  0.0102  0.1901  0.4360  77.6%
##
## Details on meta-analytical method:
## - Inverse variance method
## - Restricted maximum-likelihood estimator for tau^2
```

10.7 Complications - All

10.7.1 Meta-analysis

```
comp_metabin <- metabin(data = flexiurs_only,
                          event.c = complications_postop_overall_n_control,
                        n.c = sample_size_control,
                        event.e = complications_postop_overall_n_suction,
                        n.e = sample_size_suction,
                        studlab = author_year)
comp_metabin
```

```
## Number of studies: k = 7
## Number of observations: o = 1084
## Number of events: e = 96
##
##                           RR           95%-CI     z  p-value
## Common effect model  0.4237 [0.2831; 0.6339] -4.18 < 0.0001
## Random effects model 0.4420 [0.2931; 0.6664] -3.90 < 0.0001
##
## Quantifying heterogeneity:
##  tau^2 = 0 [0.0000; 2.2180]; tau = 0 [0.0000; 1.4893]
##  I^2 = 0.0% [0.0%; 70.8%]; H = 1.00 [1.00; 1.85]
##
## Test of heterogeneity:
##     Q d.f. p-value
##  3.54    6  0.7386
##
## Details on meta-analytical method:
## - Mantel-Haenszel method
## - Inverse variance method
## - Restricted maximum-likelihood estimator for tau^2
## - Q-Profile method for confidence interval of tau^2 and tau
## - Continuity correction of 0.5 in studies with zero cell frequencies
```

10.7.2 Forest plot

```r
forest(comp_metabin,
       sortvar = TE)
```

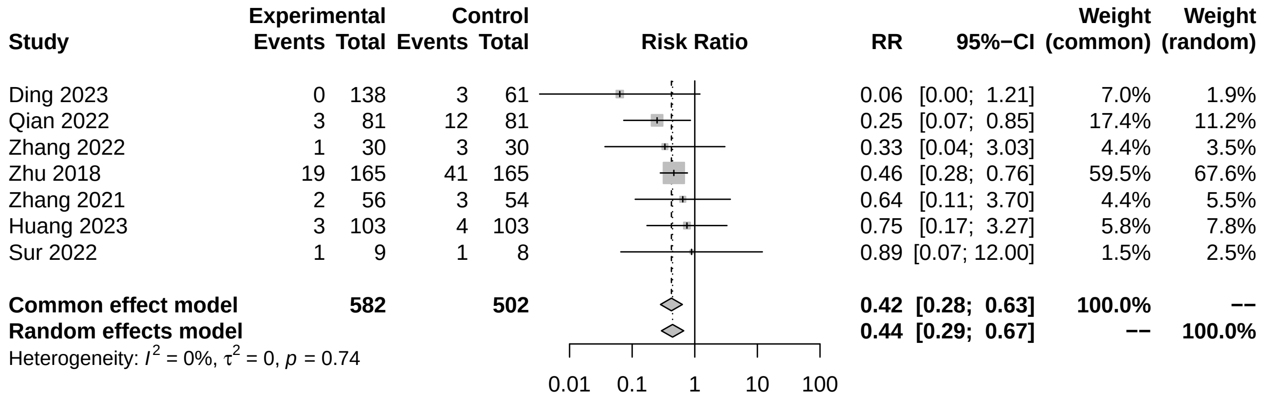

10.7.3 Trim and Fill

```r
trimfill(comp_metabin)
```

```
## Number of studies: k = 7 (with 0 added studies)
## Number of observations: o = 1084
## Number of events: e = 96
##
##                          RR           95%-CI     z  p-value
## Random effects model 0.4420 [0.2931; 0.6664] -3.90 < 0.0001
##
## Quantifying heterogeneity:
##  tau^2 = 0 [0.0000; 2.2180]; tau = 0 [0.0000; 1.4893]
##  I^2 = 0.0% [0.0%; 70.8%]; H = 1.00 [1.00; 1.85]
##
## Test of heterogeneity:
##     Q d.f. p-value
##  3.54    6  0.7386
##
## Details on meta-analytical method:
## - Inverse variance method
## - Restricted maximum-likelihood estimator for tau^2
## - Q-Profile method for confidence interval of tau^2 and tau
## - Trim-and-fill method to adjust for funnel plot asymmetry (L-estimator)
```

```r
forest(trimfill(comp_metabin),
       sortvar = TE)
```

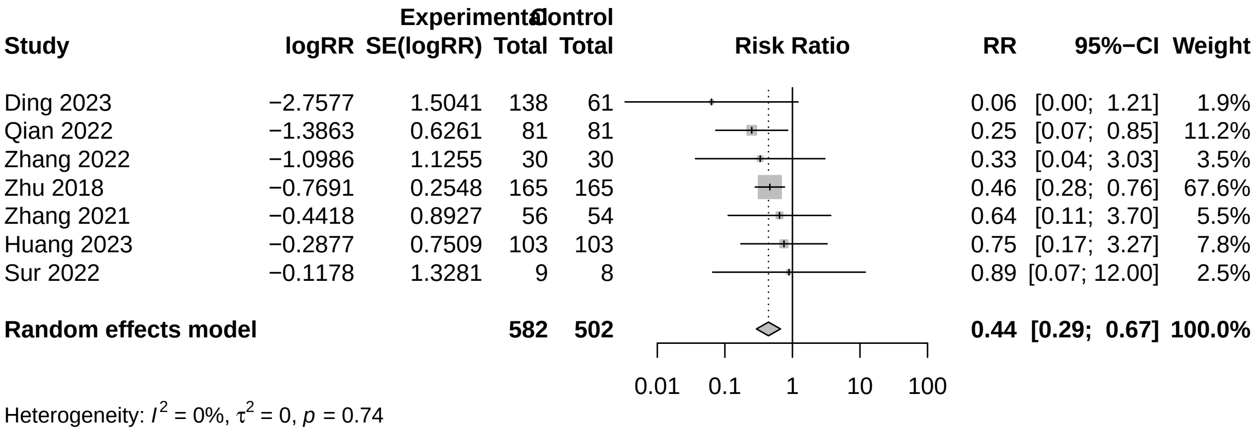

10.7.4 Funnel plot

```
funnel(trimfill(comp_metabin))
```

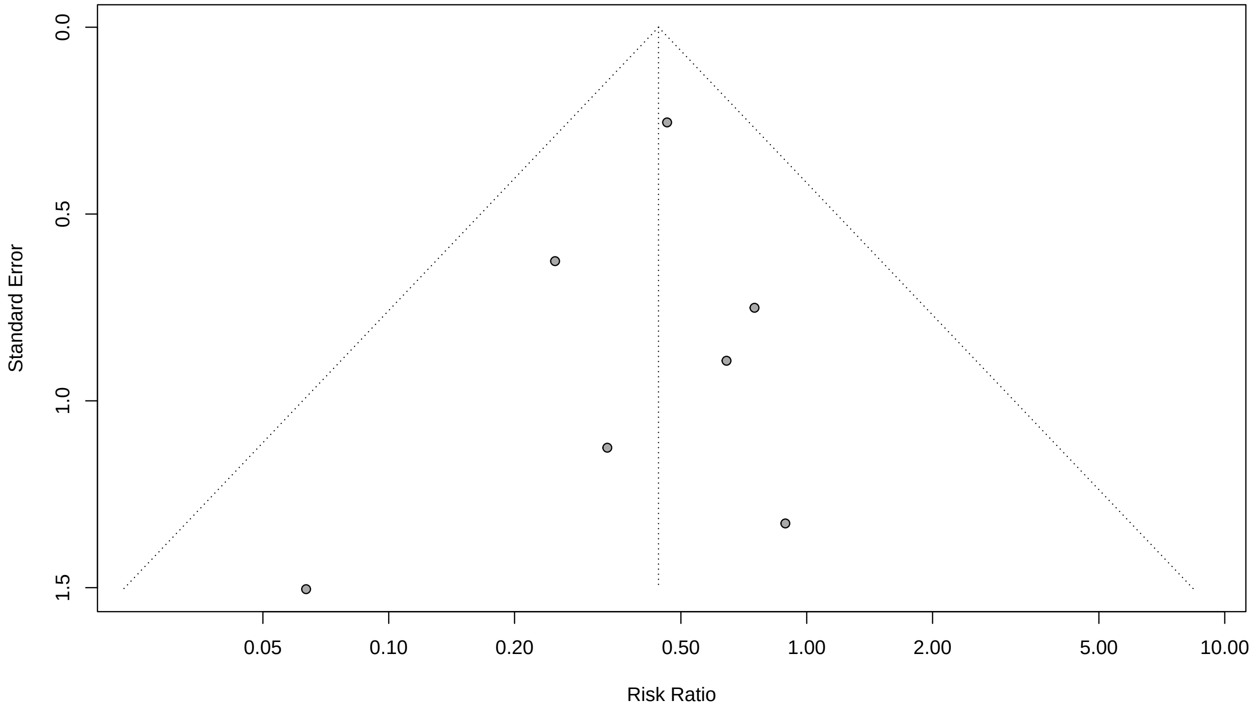

10.7.5 Baujat

```
baujat(comp_metabin, pos = 1)
```

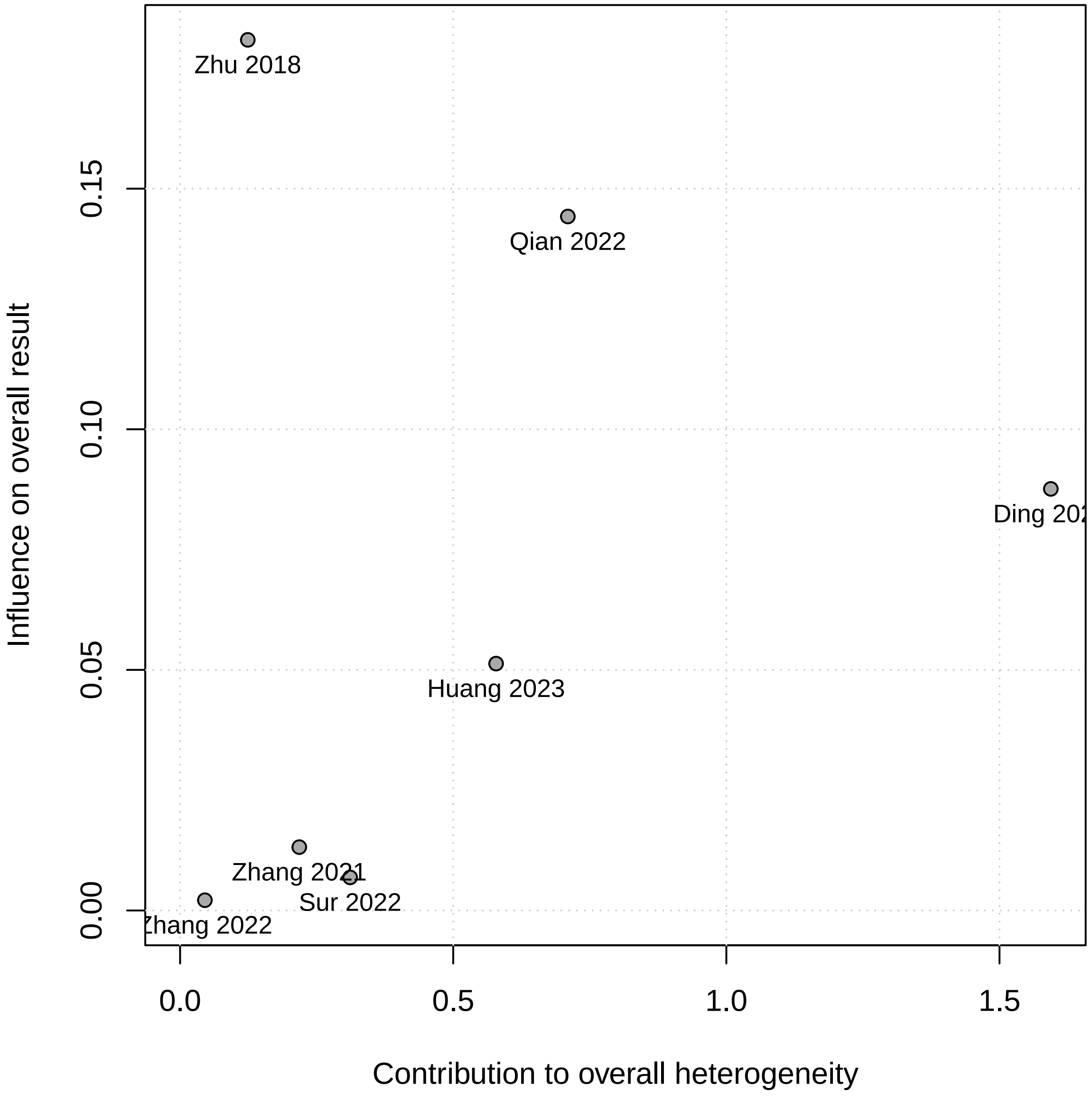

10.7.6 Leave one out

```
metainf(comp_metabin)
```

```
## Influential analysis (common effect model)
##
##                            RR           95%-CI  p-value   tau^2    tau   I^2
## Omitting Sur 2022     0.4164 [0.2768; 0.6265] < 0.0001  0.0000  0.0000  0.0%
## Omitting Zhu 2018     0.3654 [0.1847; 0.7228]   0.0038  0.0000  0.0000  0.0%
## Omitting Zhang 2021   0.4135 [0.2731; 0.6260] < 0.0001  0.0000  0.0000  0.0%
## Omitting Huang 2023   0.4036 [0.2650; 0.6145] < 0.0001  0.0000  0.0000  0.0%
## Omitting Ding 2023    0.4508 [0.2987; 0.6805]   0.0001  0.0000  0.0000  0.0%
## Omitting Qian 2022    0.4603 [0.3001; 0.7058]   0.0004  0.0000  0.0000  0.0%
## Omitting Zhang 2022   0.4278 [0.2839; 0.6445] < 0.0001  0.0000  0.0015  0.0%
##
## Pooled estimate       0.4237 [0.2831; 0.6339] < 0.0001  0.0000  0.0000  0.0%
##
## Details on meta-analytical method:
## - Mantel-Haenszel method
## - Restricted maximum-likelihood estimator for tau^2
```

10.8 Fever

10.8.1 Meta-analysis

```
fever_metabin <- metabin(data = flexiurs_only,
                          event.c = fever_n_control,
                        n.c = sample_size_control,
                        event.e = fever_n_suction,
                        n.e = sample_size_suction,
                        studlab = author_year)
fever_metabin
```

```
## Number of studies: k = 6
## Number of observations: o = 1084
## Number of events: e = 66
##
##                            RR           95%-CI     z p-value
## Common effect model  0.3662 [0.2194; 0.6113] -3.84  0.0001
## Random effects model 0.3830 [0.2261; 0.6487] -3.57  0.0004
##
## Quantifying heterogeneity:
##  tau^2 = 0 [0.0000; 3.1138]; tau = 0 [0.0000; 1.7646]
##  I^2 = 0.0% [0.0%; 74.6%]; H = 1.00 [1.00; 1.99]
##
## Test of heterogeneity:
##     Q d.f. p-value
##  3.05    5  0.6924
##
## Details on meta-analytical method:
## - Mantel-Haenszel method
## - Inverse variance method
## - Restricted maximum-likelihood estimator for tau^2
## - Q-Profile method for confidence interval of tau^2 and tau
## - Continuity correction of 0.5 in studies with zero cell frequencies
```

10.8.2 Forest plot

```r
forest(fever_metabin,
       sortvar = TE)
```

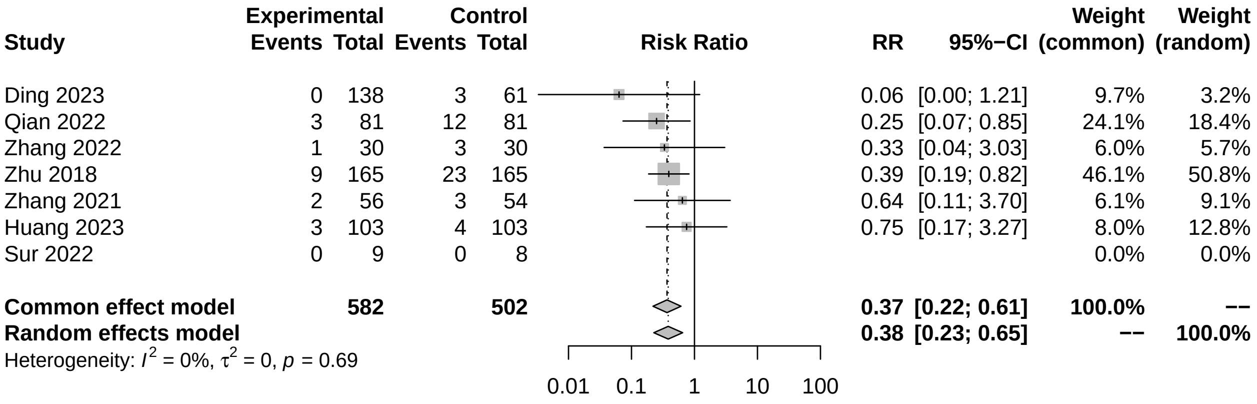

10.8.3 Trim and Fill

```
trimfill(fever_metabin)
```

```
## Warning in trimfill.meta(fever_metabin): 1 observation(s) dropped due to
## missing values

## Number of studies: k = 6 (with 0 added studies)
## Number of observations: o = 1067
## Number of events: e = 66
##
##                            RR           95%-CI     z p-value
## Random effects model 0.3830 [0.2261; 0.6487] -3.57  0.0004
##
## Quantifying heterogeneity:
##  tau^2 = 0 [0.0000; 3.1138]; tau = 0 [0.0000; 1.7646]
##  I^2 = 0.0% [0.0%; 74.6%]; H = 1.00 [1.00; 1.99]
##
## Test of heterogeneity:
##     Q d.f. p-value
##  3.05    5  0.6924
##
## Details on meta-analytical method:
## - Inverse variance method
## - Restricted maximum-likelihood estimator for tau^2
## - Q-Profile method for confidence interval of tau^2 and tau
## - Trim-and-fill method to adjust for funnel plot asymmetry (L-estimator)
```

```
forest(trimfill(fever_metabin),
       sortvar = TE)
```

```
## Warning in trimfill.meta(fever_metabin): 1 observation(s) dropped due to
## missing values
```

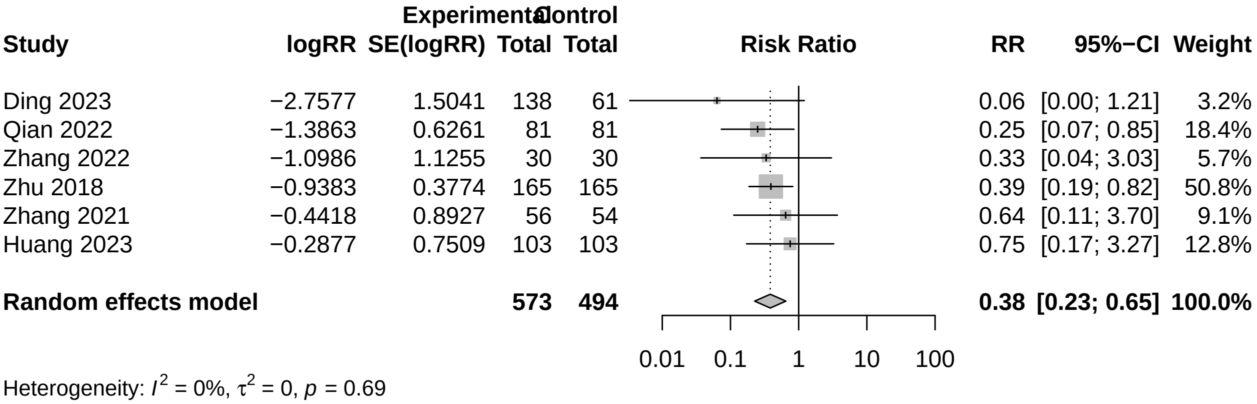

10.8.4 Funnel plot

```
funnel(trimfill(fever_metabin))
```

```
## Warning in trimfill.meta(fever_metabin): 1 observation(s) dropped due to
## missing values
```

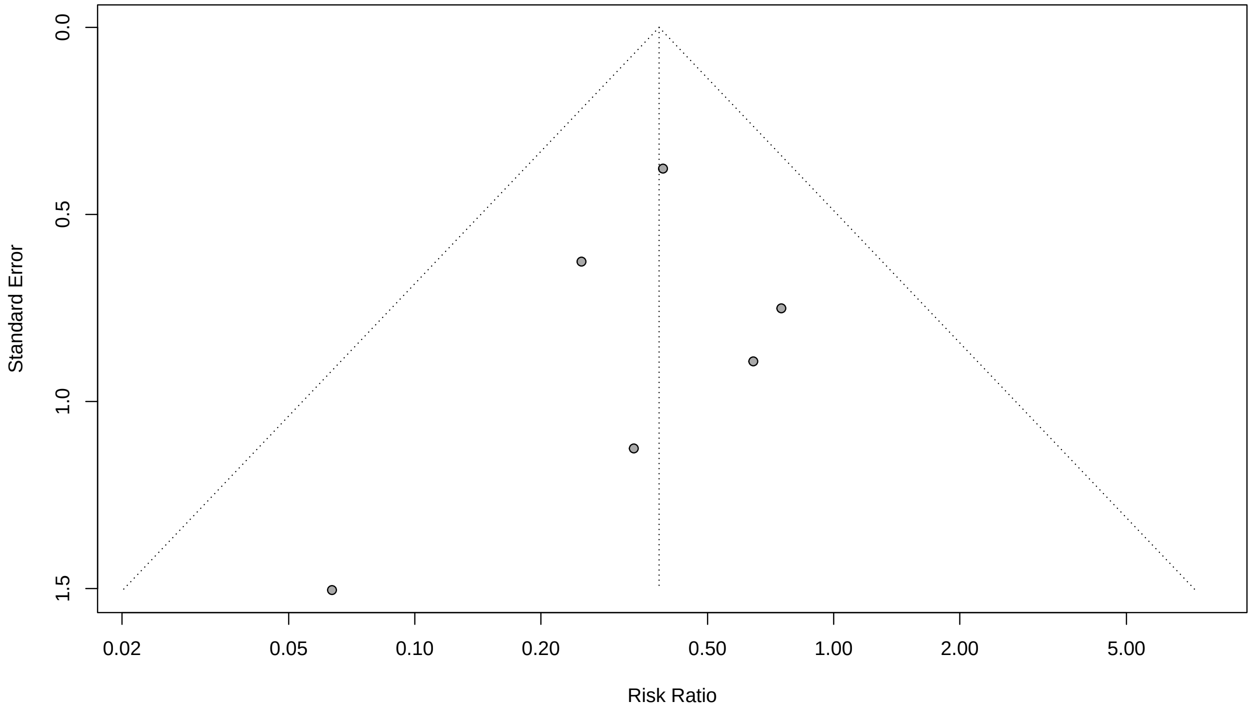

10.8.5 Baujat

```
baujat(fever_metabin, pos = 1)
```

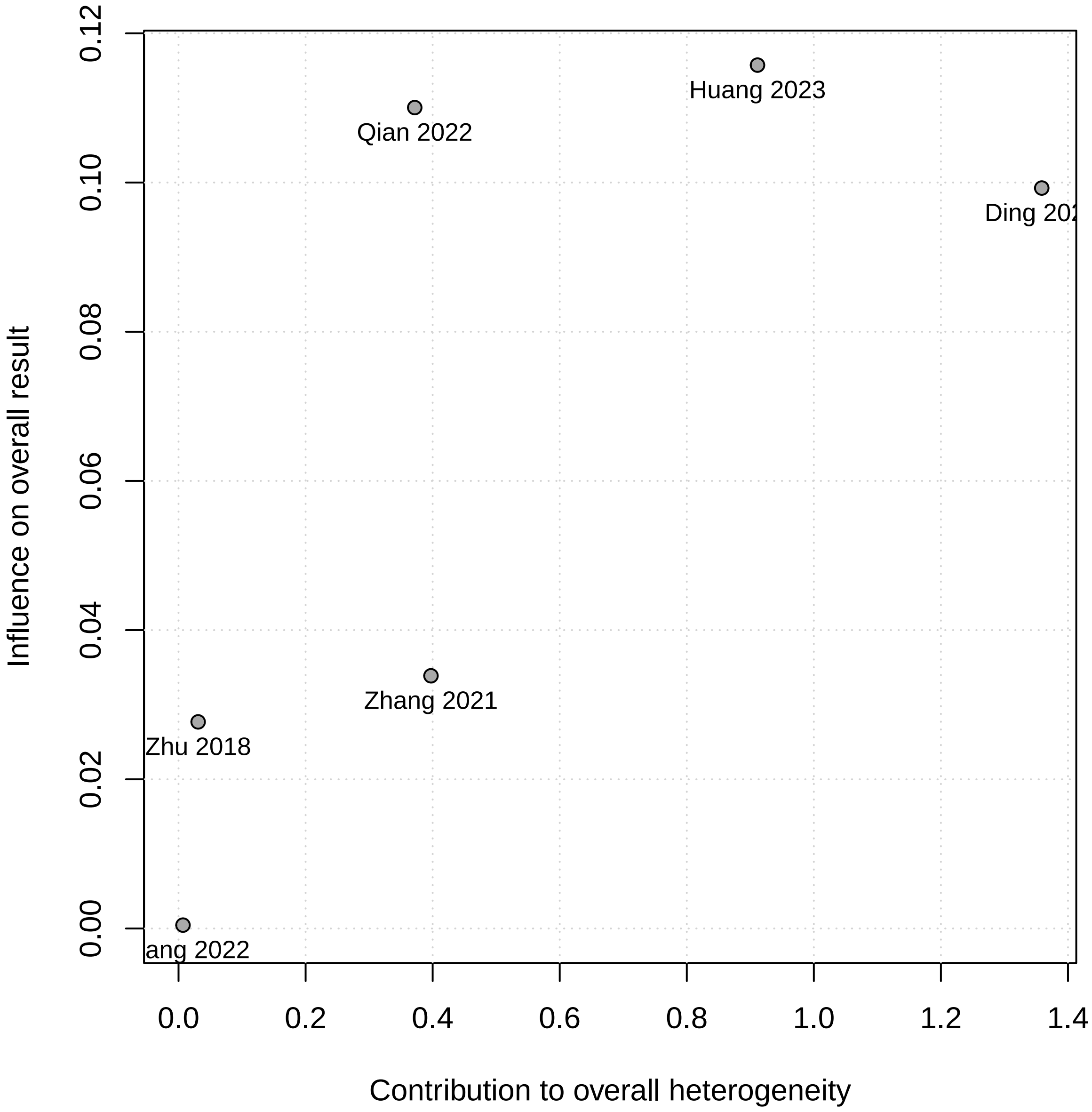

10.8.6 Leave one out

```r
metainf(fever_metabin)
```

```
## Influential analysis (common effect model)
##
##                            RR           95%-CI  p-value   tau^2    tau   I^2
## Omitting Sur 2022      0.3662 [0.2194; 0.6113]   0.0001  0.0000 0.0000  0.0%
## Omitting Zhu 2018      0.3447 [0.1693; 0.7021]   0.0033  0.0000 0.0000  0.0%
## Omitting Zhang 2021    0.3482 [0.2033; 0.5962]   0.0001  0.0000 0.0000  0.0%
## Omitting Huang 2023    0.3328 [0.1916; 0.5778] < 0.0001  0.0000 0.0000  0.0%
## Omitting Ding 2023     0.3987 [0.2349; 0.6767]   0.0007  0.0000 0.0000  0.0%
## Omitting Qian 2022     0.4030 [0.2289; 0.7096]   0.0016  0.0000 0.0000  0.0%
## Omitting Zhang 2022    0.3683 [0.2175; 0.6238]   0.0002  0.0000 0.0007  0.0%
##
## Pooled estimate        0.3662 [0.2194; 0.6113]   0.0001  0.0000 0.0000  0.0%
##
## Details on meta-analytical method:
## - Mantel-Haenszel method
## - Restricted maximum-likelihood estimator for tau^2
```

10.9 Infections

10.9.1 Meta-analysis

```
infection_metabin <- metabin(data = flexiurs_only,
                          event.c = infection_n_control,
                        n.c = sample_size_control,
                        event.e = infection_n_suction,
                        n.e = sample_size_suction,
                        studlab = author_year)
infection_metabin
```

```
## Number of studies: k = 7
## Number of observations: o = 1084
## Number of events: e = 67
##
##                           RR           95%-CI     z  p-value
## Common effect model  0.3642 [0.2197; 0.6037] -3.92 < 0.0001
## Random effects model 0.3802 [0.2262; 0.6392] -3.65   0.0003
##
## Quantifying heterogeneity:
##  tau^2 = 0 [0.0000; 1.6283]; tau = 0 [0.0000; 1.2760]
##  I^2 = 0.0% [0.0%; 70.8%]; H = 1.00 [1.00; 1.85]
##
## Test of heterogeneity:
##     Q d.f. p-value
##  3.07    6  0.7995
##
## Details on meta-analytical method:
## - Mantel-Haenszel method
## - Inverse variance method
## - Restricted maximum-likelihood estimator for tau^2
## - Q-Profile method for confidence interval of tau^2 and tau
## - Continuity correction of 0.5 in studies with zero cell frequencies
```

10.9.2 Forest plot

```
forest(infection_metabin,
       sortvar = TE)
```

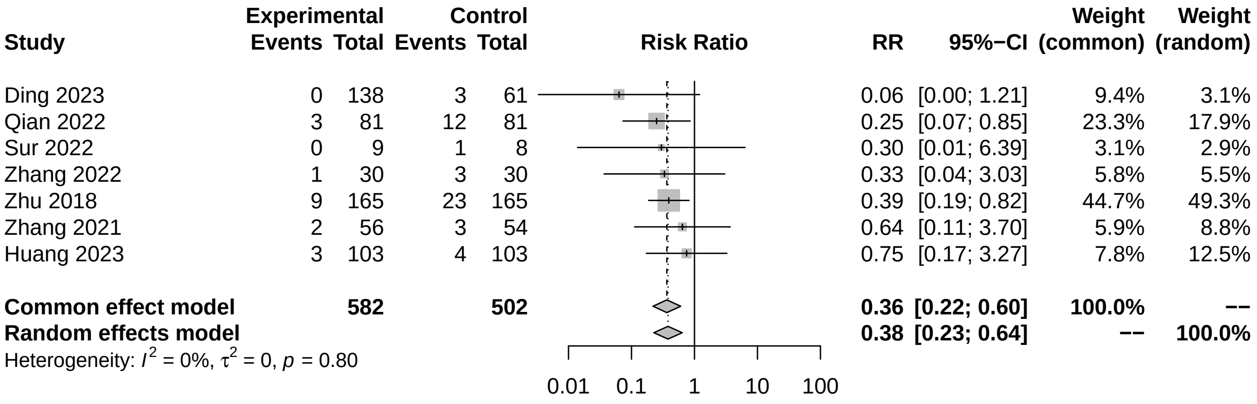

10.9.3 Trim and Fill

```r
trimfill(infection_metabin)
```

```
## Number of studies: k = 8 (with 1 added studies)
## Number of observations: o = 1283
## Number of events: e = 70
##
##                           RR           95%-CI     z p-value
## Random effects model 0.4027 [0.2414; 0.6716] -3.49  0.0005
##
## Quantifying heterogeneity:
##  tau^2 = 0 [0.0000; 2.6099]; tau = 0 [0.0000; 1.6155]
##  I^2 = 0.0% [0.0%; 67.6%]; H = 1.00 [1.00; 1.76]
##
## Test of heterogeneity:
##     Q d.f. p-value
##  4.63    7  0.7049
##
## Details on meta-analytical method:
## - Inverse variance method
## - Restricted maximum-likelihood estimator for tau^2
## - Q-Profile method for confidence interval of tau^2 and tau
## - Trim-and-fill method to adjust for funnel plot asymmetry (L-estimator)
```

```r
forest(trimfill(infection_metabin),
       sortvar = TE)
```

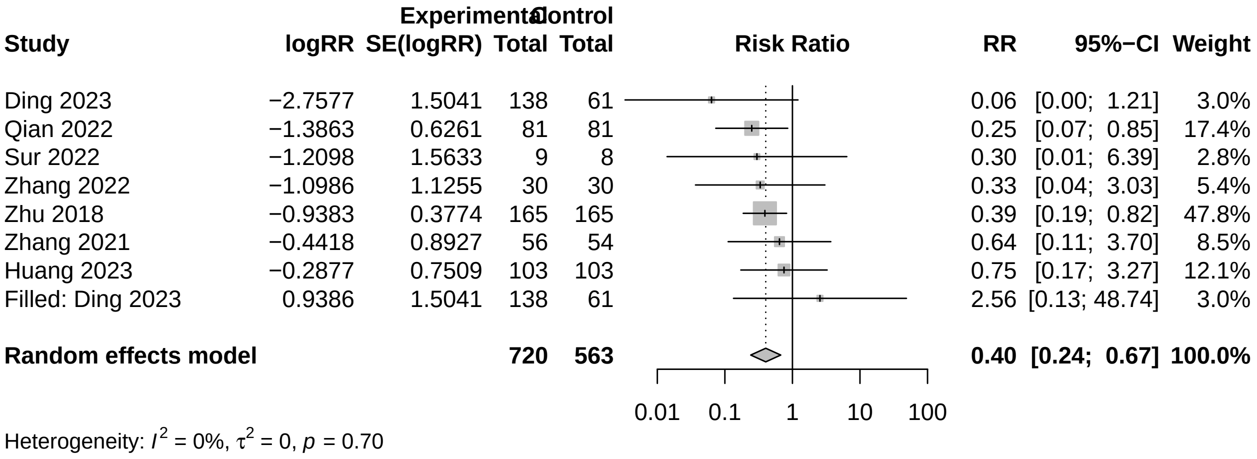

10.9.4 Funnel plot

```
funnel(trimfill(infection_metabin))
```

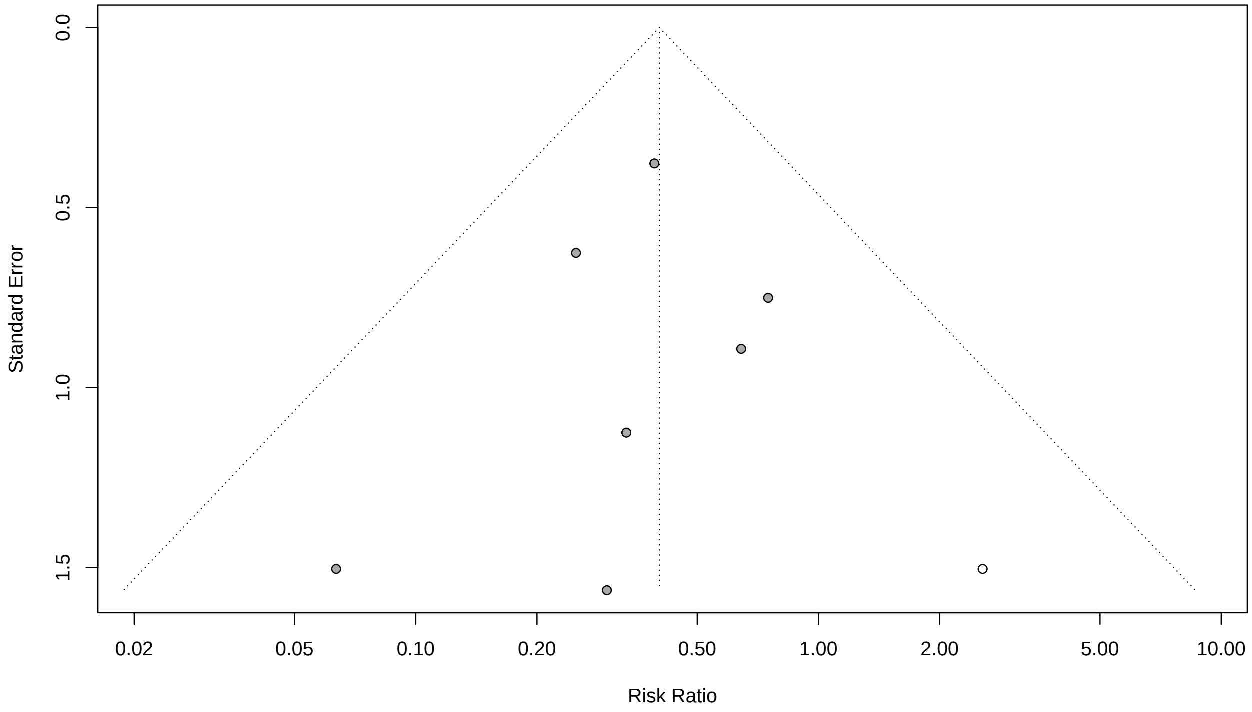

10.9.5 Baujat

```
baujat(infection_metabin, pos = 1)
```

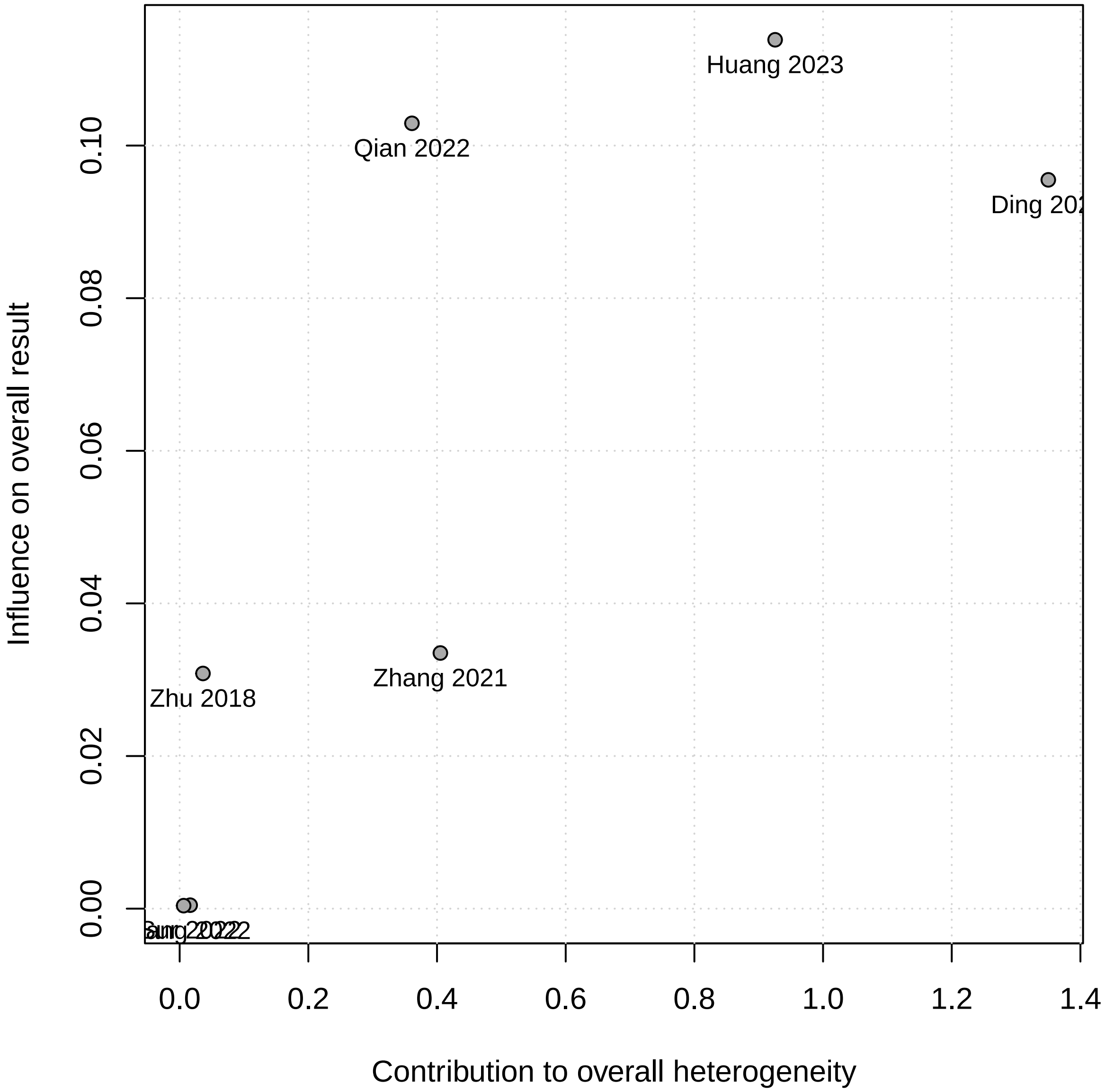

10.9.6 Leave one out

```
metainf(infection_metabin)
```

```
## Influential analysis (common effect model)
##
##                          RR           95%-CI  p-value   tau^2    tau   I^2
## Omitting Sur 2022    0.3662 [0.2194; 0.6113]   0.0001  0.0000 0.0000  0.0%
## Omitting Zhu 2018    0.3423 [0.1712; 0.6843]   0.0024  0.0000 0.0000  0.0%
## Omitting Zhang 2021  0.3466 [0.2040; 0.5888] < 0.0001  0.0000 0.0000  0.0%
## Omitting Huang 2023  0.3317 [0.1927; 0.5709] < 0.0001  0.0000 0.0000  0.0%
## Omitting Ding 2023   0.3954 [0.2348; 0.6658]   0.0005  0.0000 0.0000  0.0%
## Omitting Qian 2022   0.3989 [0.2287; 0.6957]   0.0012  0.0000 0.0000  0.0%
## Omitting Zhang 2022  0.3661 [0.2178; 0.6153]   0.0001  0.0000 0.0000  0.0%
##
## Pooled estimate      0.3642 [0.2197; 0.6037] < 0.0001  0.0000 0.0000  0.0%
##
## Details on meta-analytical method:
## - Mantel-Haenszel method
## - Restricted maximum-likelihood estimator for tau^2
```

10.10 Sepsis

10.10.1 Meta-analysis

```
sepsis_metabin <- metabin(data = flexiurs_only,
                          event.c = sepsis_n_control,
                        n.c = sample_size_control,
                        event.e = sepsis_n_suction,
                        n.e = sample_size_suction,
                        studlab = author_year)
sepsis_metabin
```

```
## Number of studies: k = 1
## Number of observations: o = 1084
## Number of events: e = 14
##
##                          RR           95%-CI     z p-value
## Common effect model  0.2727 [0.0775; 0.9598] -2.02  0.0430
## Random effects model 0.2727 [0.0775; 0.9598] -2.02  0.0430
##
## Quantifying heterogeneity:
##  tau^2 = NA; tau = NA; I^2 = NA; H = NA
##
## Details on meta-analytical method:
## - Mantel-Haenszel method
## - Inverse variance method
## - Restricted maximum-likelihood estimator for tau^2
```

10.10.2 Forest plot

```r
forest(sepsis_metabin,
       sortvar = TE)
```

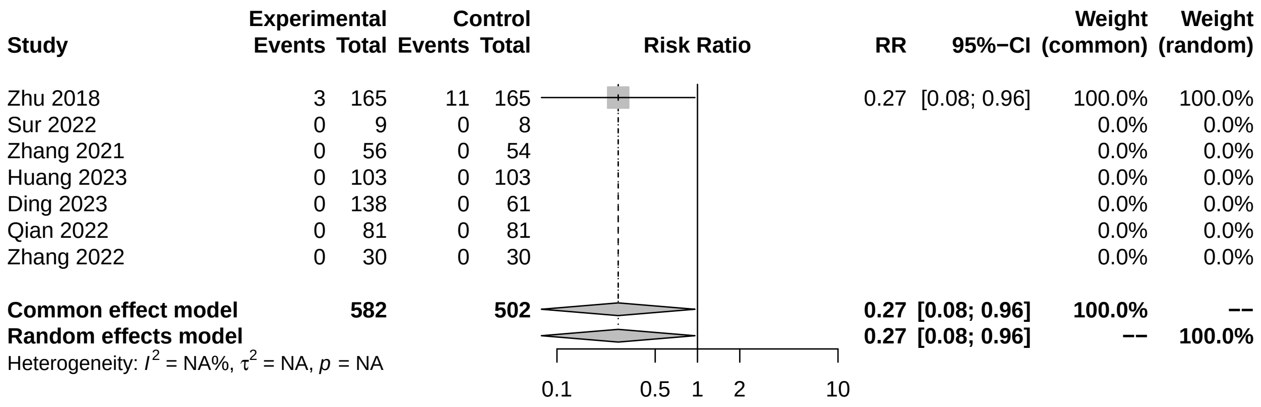

10.10.3 Trim and Fill

```
#trimfill(sepsis_metabin)
#forest(trimfill(sepsis_metabin),
#       sortvar = TE)
```

10.10.4 Funnel plot

```
funnel((sepsis_metabin))
```

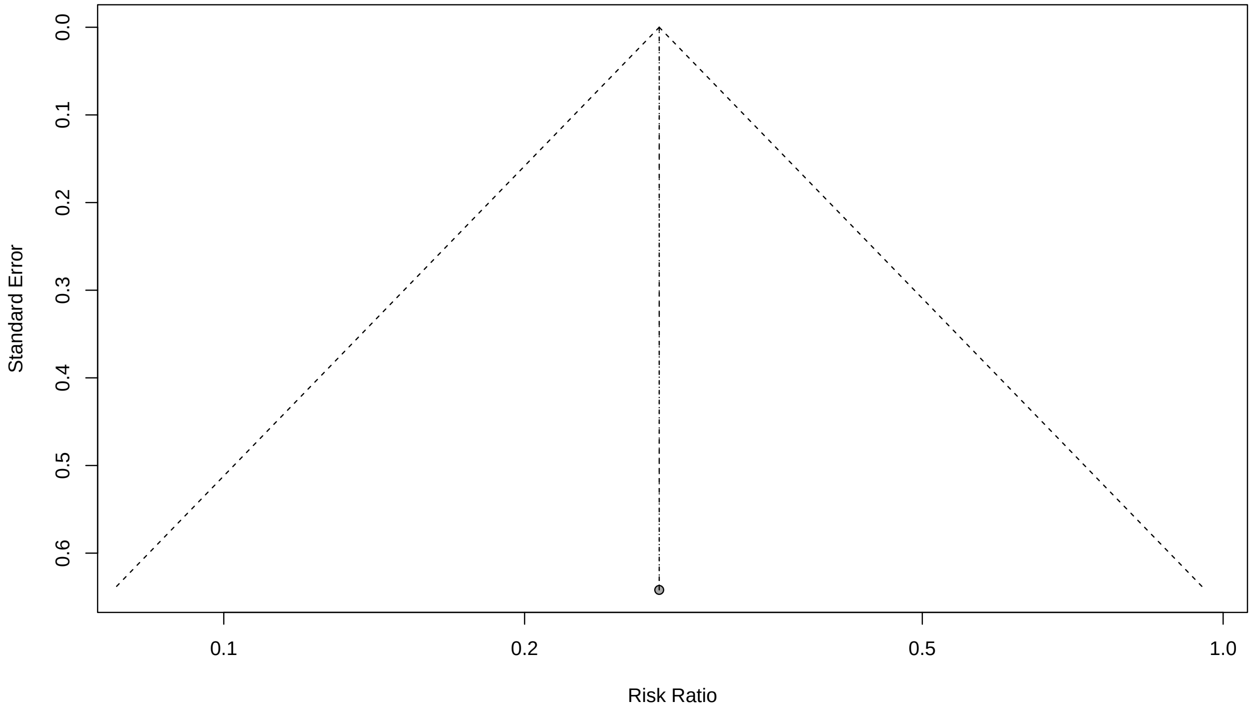

10.10.5 Baujat

```r
baujat(sepsis_metabin, pos = 1)
```

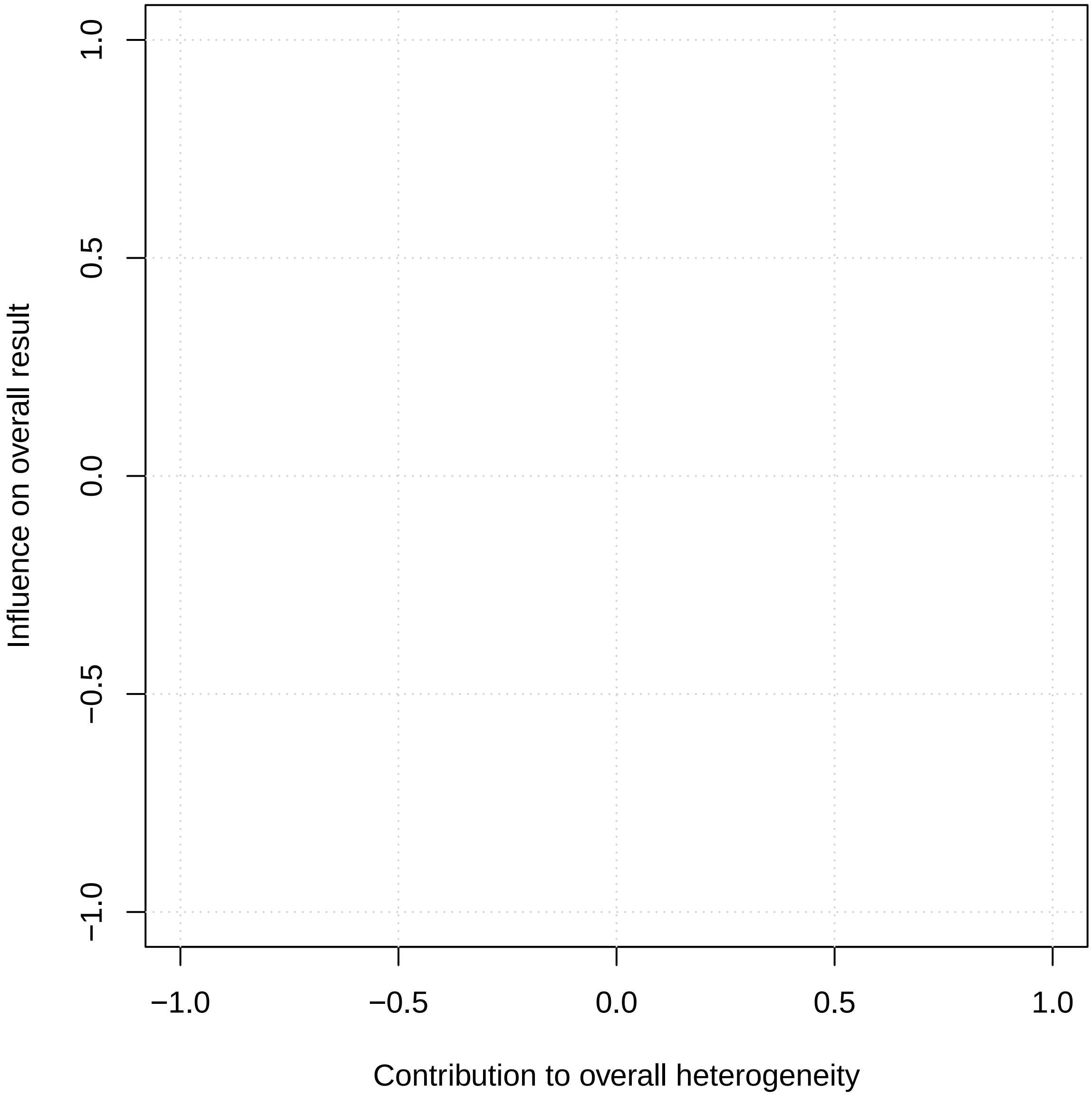

10.10.6 Leave one out

```r
metainf(sepsis_metabin)
```

```
## Influential analysis (common effect model)
##
##                           RR           95%-CI p-value tau^2 tau I^2
## Omitting Sur 2022     0.2727 [0.0775; 0.9598]  0.0430
## Omitting Zhu 2018
## Omitting Zhang 2021   0.2727 [0.0775; 0.9598]  0.0430
## Omitting Huang 2023   0.2727 [0.0775; 0.9598]  0.0430
## Omitting Ding 2023    0.2727 [0.0775; 0.9598]  0.0430
## Omitting Qian 2022    0.2727 [0.0775; 0.9598]  0.0430
## Omitting Zhang 2022   0.2727 [0.0775; 0.9598]  0.0430
##
## Pooled estimate       0.2727 [0.0775; 0.9598]  0.0430
##
## Details on meta-analytical method:
## - Mantel-Haenszel method
## - Restricted maximum-likelihood estimator for tau^2
```

10.11 Abscess

10.11.1 Meta-analysis

```
abscess_metabin <- metabin(data = flexiurs_only,
                             event.c = abscess_n_control,
                           n.c = sample_size_control,
                           event.e = abscess_n_suction,
                           n.e = sample_size_suction,
                           studlab = author_year)
abscess_metabin
```

```
## Number of studies: k = 0
## Number of observations: o = 1084
## Number of events: e = 0
##
##                       RR 95%-CI  z p-value
## Common effect model  NA        --      --
## Random effects model NA        --      --
##
## Quantifying heterogeneity:
##  tau^2 = NA; tau = NA; I^2 = NA; H = NA
##
## Details on meta-analytical method:
## - Mantel-Haenszel method
## - Inverse variance method
## - Restricted maximum-likelihood estimator for tau^2
```

10.11.2 Forest plot

```
forest(abscess_metabin,
       sortvar = TE)
```

Study	Experimental Events	Experimental Total	Control Events	Control Total	Risk Ratio	RR	95%−CI	Weight (common)	Weight (random)
Sur 2022	0	9	0	8				0.0%	0.0%
Zhu 2018	0	165	0	165				0.0%	0.0%
Zhang 2021	0	56	0	54				0.0%	0.0%
Huang 2023	0	103	0	103				0.0%	0.0%
Ding 2023	0	138	0	61				0.0%	0.0%
Qian 2022	0	81	0	81				0.0%	0.0%
Zhang 2022	0	30	0	30				0.0%	0.0%
**Common effect model**		**582**		**502**				**100.0%**	**−−**
**Random effects model**								**−−**	**100.0%**

Heterogeneity: I^2 = NA%, τ^2 = NA, p = NA

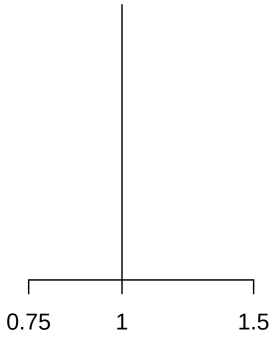

10.11.3 Trim and Fill

```
#trimfill(abscess_metabin)
#forest(trimfill(abscess_metabin),
#        sortvar = TE)
```

10.11.4 Funnel plot

```
#funnel((abscess_metabin))
```

10.11.5 Baujat

```r
#baujat(abscess_metabin, pos = 1)
```

10.11.6 Leave one out

```
#metainf(abscess_metabin)
```

10.12 Haematoma

10.12.1 Meta-analysis

```
haematoma_metabin <- metabin(data = flexiurs_only,
                         event.c = hematoma_n_control,
                       n.c = sample_size_control,
                       event.e = hematoma_n_suction,
                       n.e = sample_size_suction,
                       studlab = author_year)
haematoma_metabin
```

```
## Number of studies: k = 0
## Number of observations: o = 1084
## Number of events: e = 0
##
##                       RR 95%-CI  z p-value
## Common effect model  NA        --      --
## Random effects model NA        --      --
##
## Quantifying heterogeneity:
##  tau^2 = NA; tau = NA; I^2 = NA; H = NA
##
## Details on meta-analytical method:
## - Mantel-Haenszel method
## - Inverse variance method
## - Restricted maximum-likelihood estimator for tau^2
```

10.12.2 Forest plot

```
forest(haematoma_metabin,
       sortvar = TE)
```

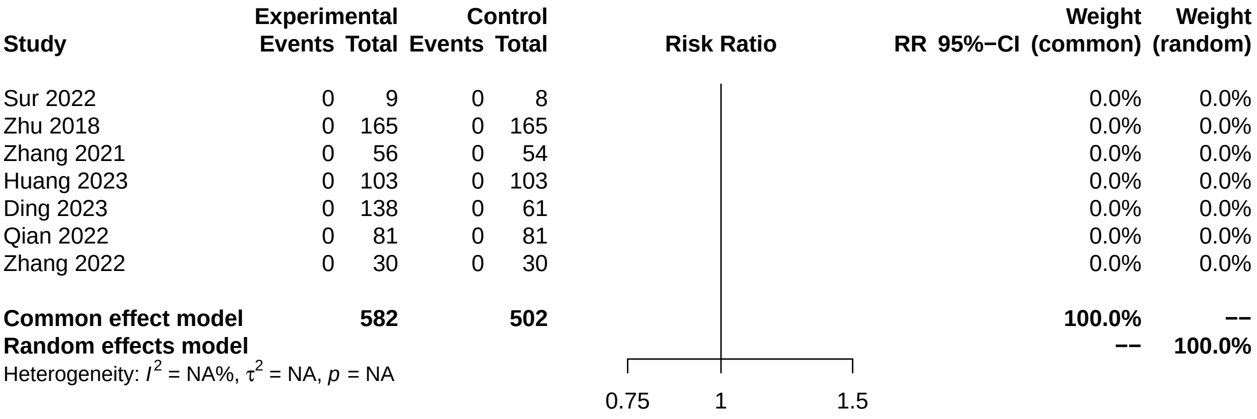

10.12.3 Trim and Fill

```
#trimfill(haematoma_metabin)
#forest(trimfill(haematoma_metabin),
#       sortvar = TE)
```

10.12.4 Funnel plot

```
#funnel((haematoma_metabin))
```

10.12.5 Baujat

```
#baujat(haematoma_metabin, pos = 1)
```

10.12.6 Leave one out

```
#metainf(haematoma_metabin)
```

10.13 Pain

10.13.1 Meta-analysis

```
pain_metabin <- metabin(data = flexiurs_only,
                         event.c = pain_n_control,
                       n.c = sample_size_control,
                       event.e = pain_n_suction,
                       n.e = sample_size_suction,
                       studlab = author_year)
pain_metabin
```

```
## Number of studies: k = 0
## Number of observations: o = 1084
## Number of events: e = 0
##
##                      RR 95%-CI  z p-value
## Common effect model  NA         --      --
## Random effects model NA         --      --
##
## Quantifying heterogeneity:
##  tau^2 = NA; tau = NA; I^2 = NA; H = NA
##
## Details on meta-analytical method:
## - Mantel-Haenszel method
## - Inverse variance method
## - Restricted maximum-likelihood estimator for tau^2
```

10.13.2 Forest plot

```r
forest(pain_metabin,
       sortvar = TE)
```

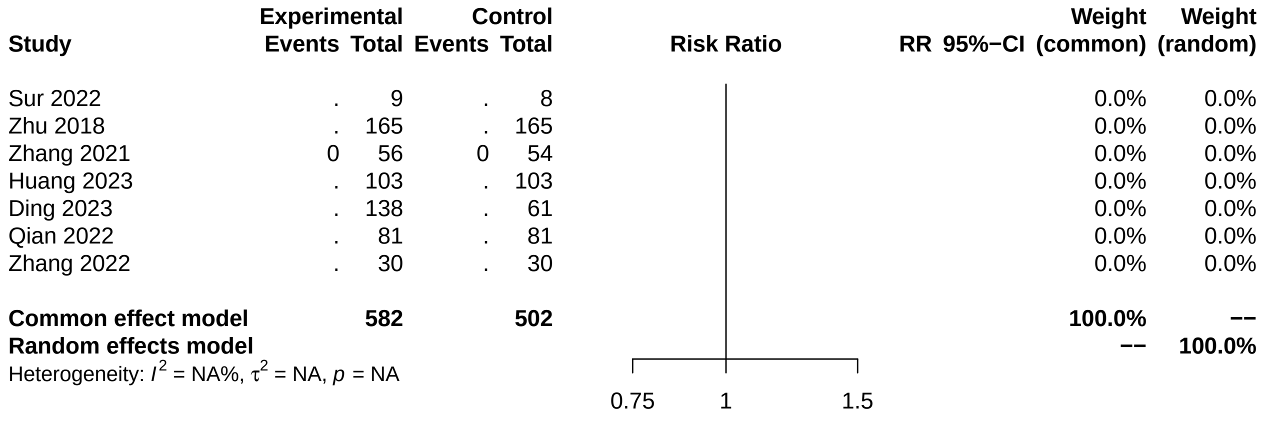

10.13.3 Trim and Fill

```
#trimfill(pain_metabin)
#forest(trimfill(pain_metabin),
 #       sortvar = TE)
```

10.13.4 Funnel plot

```
#funnel(trimfill(pain_metabin))
```

10.13.5 Baujat

```r
#baujat(pain_metabin, pos = 1)
```

10.13.6 Leave one out

```r
#metainf(pain_metabin, pos = 1)
```

10.14 Stricture

10.14.1 Meta-analysis

```
stricture_metabin <- metabin(data = flexiurs_only,
                              event.c = stricture_n_control,
                            n.c = sample_size_control,
                            event.e = stricture_n_suction,
                            n.e = sample_size_suction,
                            studlab = author_year)
stricture_metabin
```

```
## Number of studies: k = 1
## Number of observations: o = 1084
## Number of events: e = 1
##
##                             RR            95%-CI    z p-value
## Common effect model  3.0000 [0.1231; 73.1112] 0.67  0.5001
## Random effects model 3.0000 [0.1231; 73.1096] 0.67  0.5001
##
## Quantifying heterogeneity:
##  tau^2 = NA; tau = NA; I^2 = NA; H = NA
##
## Details on meta-analytical method:
## - Mantel-Haenszel method
## - Inverse variance method
## - Restricted maximum-likelihood estimator for tau^2
## - Continuity correction of 0.5 in studies with zero cell frequencies
```

10.14.2 Forest plot

```r
forest(stricture_metabin,
       sortvar = TE)
```

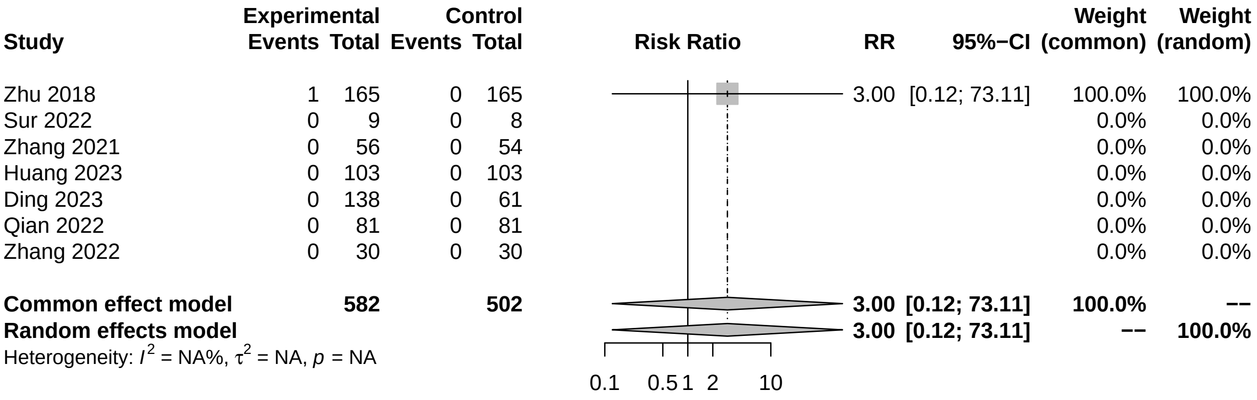

10.14.3 Trim and Fill

```
#trimfill(stricture_metabin)
#forest(trimfill(stricture_metabin),
#       sortvar = TE)
```

10.14.4 Funnel plot

```
funnel((stricture_metabin))
```

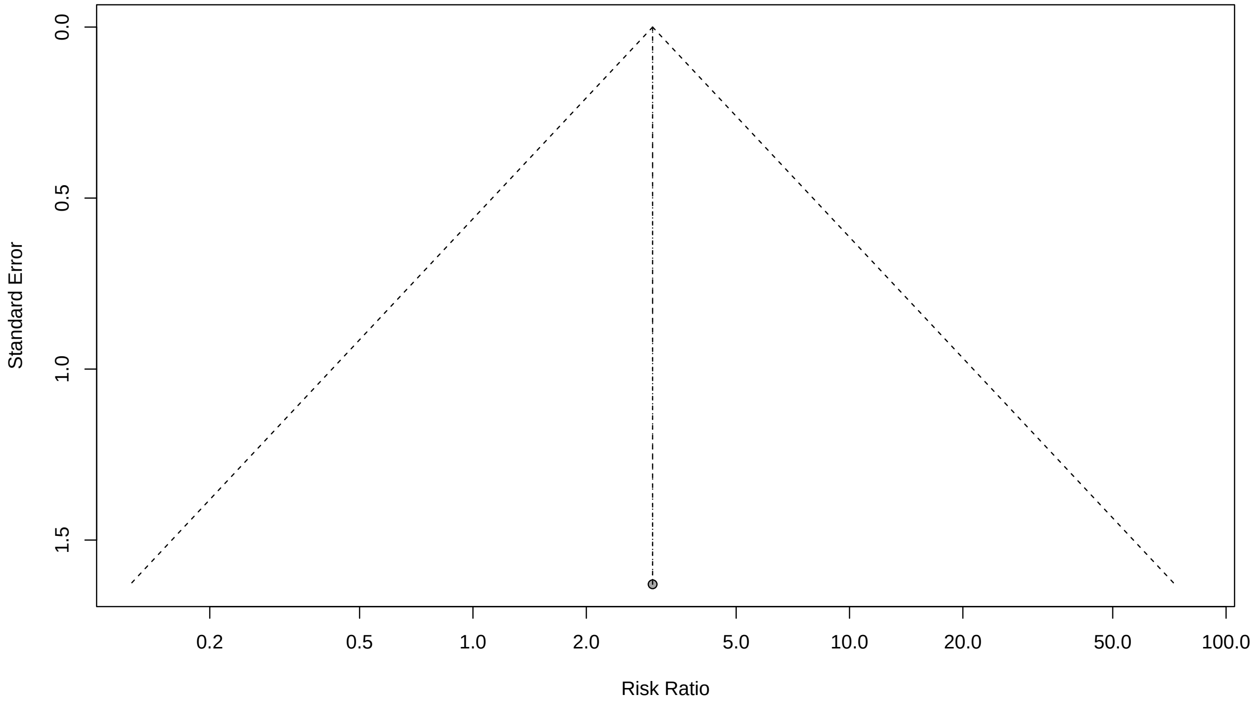

10.14.5 Baujat

```r
baujat(stricture_metabin, pos = 1)
```

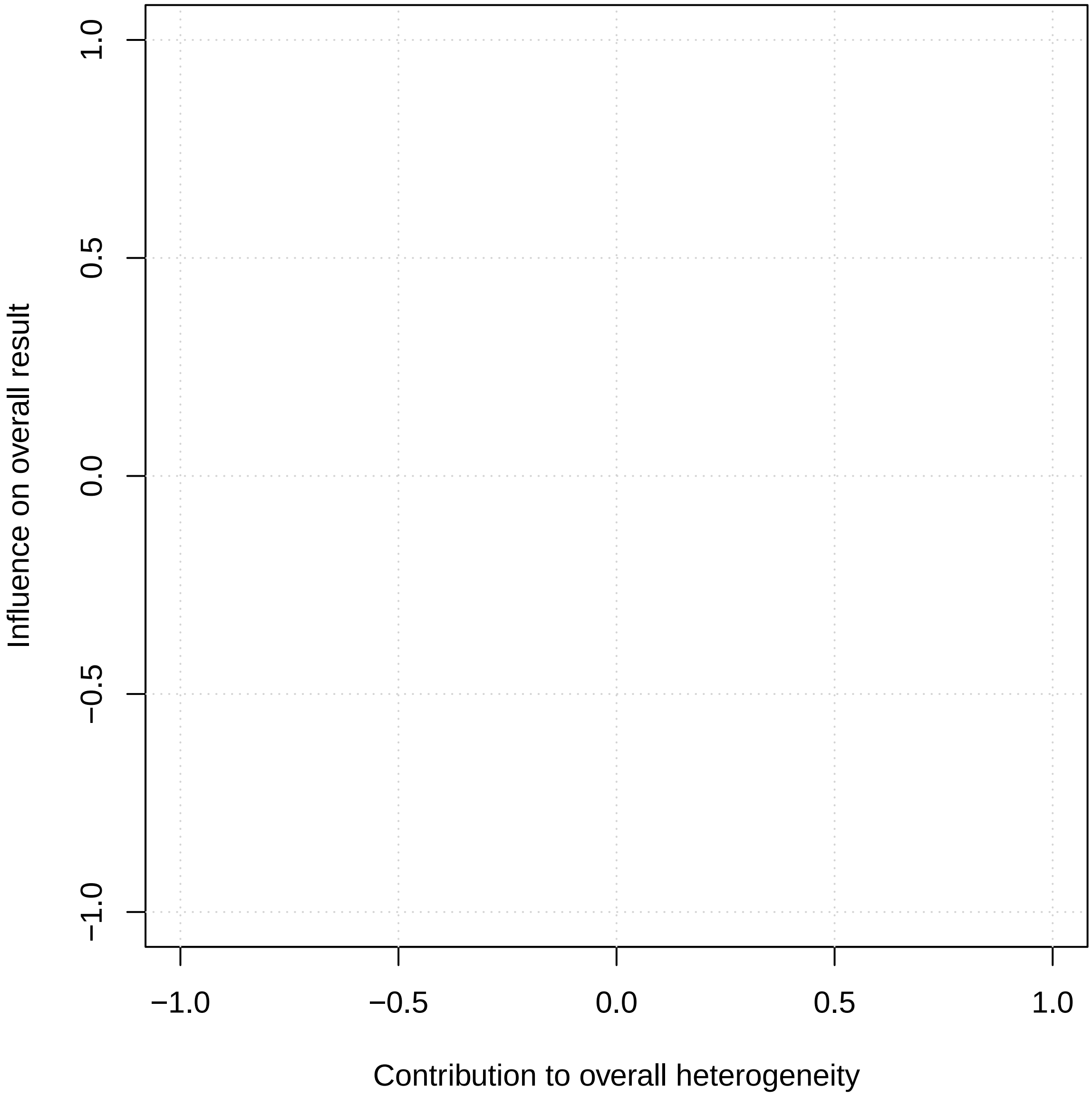

10.14.6 Leave one out

```r
metainf(stricture_metabin)
```

```
## Influential analysis (common effect model)
##
##                              RR            95%-CI p-value tau^2 tau I^2
## Omitting Sur 2022      3.0000 [0.1231; 73.1112]  0.5001
## Omitting Zhu 2018
## Omitting Zhang 2021    3.0000 [0.1231; 73.1112]  0.5001
## Omitting Huang 2023    3.0000 [0.1231; 73.1112]  0.5001
## Omitting Ding 2023     3.0000 [0.1231; 73.1112]  0.5001
## Omitting Qian 2022     3.0000 [0.1231; 73.1112]  0.5001
## Omitting Zhang 2022    3.0000 [0.1231; 73.1112]  0.5001
##
## Pooled estimate        3.0000 [0.1231; 73.1112]  0.5001
##
## Details on meta-analytical method:
## - Mantel-Haenszel method
## - Restricted maximum-likelihood estimator for tau^2
```

10.15 Embolisation required

10.15.1 Meta-analysis

```
ir_embolisation_metabin <- metabin(data = flexiurs_only,
                         event.c = embolism_ir_intervention_n_control,
                       n.c = sample_size_control,
                       event.e = embolism_ir_intervention_n_suction,
                       n.e = sample_size_suction,
                       studlab = author_year)
ir_embolisation_metabin
```

```
## Number of studies: k = 0
## Number of observations: o = 1084
## Number of events: e = 0
##
##                      RR 95%-CI  z p-value
## Common effect model  NA        --      --
## Random effects model NA        --      --
##
## Quantifying heterogeneity:
##  tau^2 = NA; tau = NA; I^2 = NA; H = NA
##
## Details on meta-analytical method:
## - Mantel-Haenszel method
## - Inverse variance method
## - Restricted maximum-likelihood estimator for tau^2
```

10.15.2 Forest plot

```r
forest(ir_embolisation_metabin,
       sortvar = TE)
```

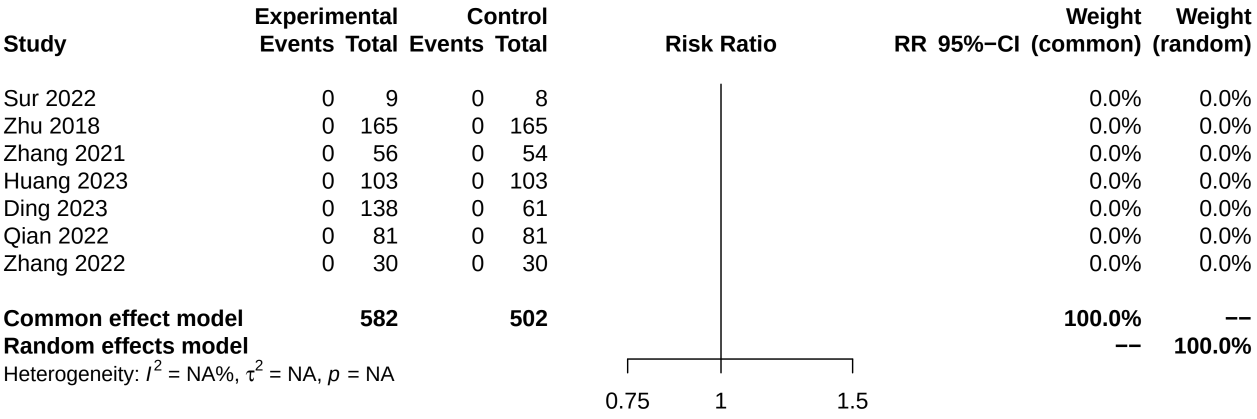

10.15.3 Trim and Fill

```
#trimfill(ir_embolisation_metabin)
#forest(trimfill(ir_embolisation_metabin),
#       sortvar = TE)
```

10.15.4 Funnel plot

```
#funnel((ir_embolisation_metabin))
```

10.15.5 Baujat

```
#baujat(ir_embolisation_metabin, pos = 1)
```

10.15.6 Leave one out

```
#metainf(ir_embolisation_metabin)
```

10.16 Transfusion

10.16.1 Meta-analysis

```
transfusion_metabin <- metabin(data = flexiurs_only,
                        event.c = transfusion_n_control,
                      n.c = sample_size_control,
                      event.e = transfusion_n_suction,
                      n.e = sample_size_suction,
                      studlab = author_year)
transfusion_metabin
```

```
## Number of studies: k = 0
## Number of observations: o = 1084
## Number of events: e = 0
##
##                        RR 95%-CI  z p-value
## Common effect model  NA        --      --
## Random effects model NA        --      --
##
## Quantifying heterogeneity:
##  tau^2 = NA; tau = NA; I^2 = NA; H = NA
##
## Details on meta-analytical method:
## - Mantel-Haenszel method
## - Inverse variance method
## - Restricted maximum-likelihood estimator for tau^2
```

10.16.2 Forest plot

```r
forest(transfusion_metabin,
       sortvar = TE)
```

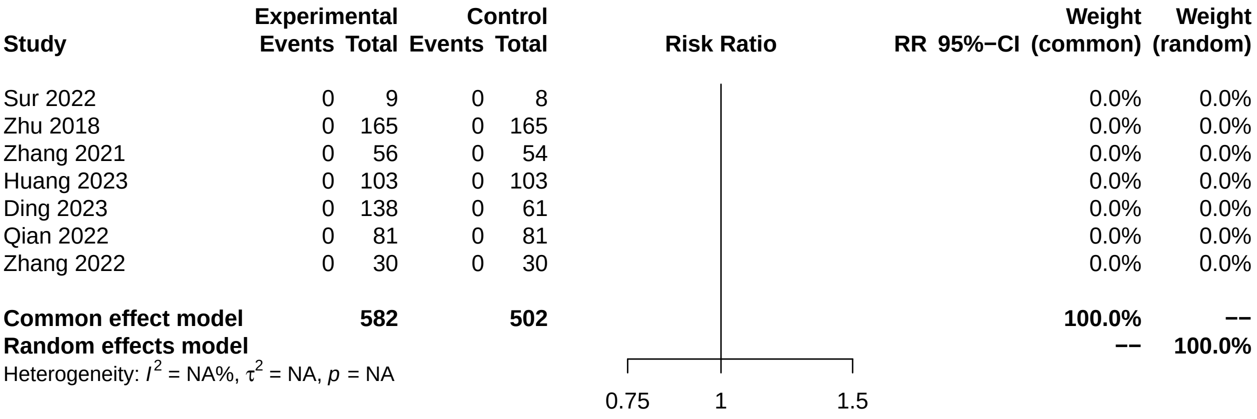

10.16.3 Trim and Fill

```
#trimfill(transfusion_metabin)
#forest(trimfill(transfusion_metabin),
#       sortvar = TE)
```

10.16.4 Funnel plot

```r
#funnel(trimfill(transfusion_metabin))
```

10.16.5 Baujat

```
#baujat(transfusion_metabin, pos = 1)
```

10.16.6 Leave one out

```
#metainf(transfusion_metabin)
```

10.17 Clavien I

10.17.1 Meta-analysis

```
clav_i_metabin <- metabin(data = flexiurs_only,
                           event.c = clavien_i_n_control,
                         n.c = sample_size_control,
                         event.e = clavien_i_n_suction,
                         n.e = sample_size_suction,
                         studlab = author_year)
clav_i_metabin
```

```
## Number of studies: k = 3
## Number of observations: o = 1084
## Number of events: e = 46
##
##                            RR           95%-CI     z p-value
## Common effect model  0.5877 [0.3352; 1.0304] -1.86  0.0635
## Random effects model 0.5798 [0.3284; 1.0236] -1.88  0.0602
##
## Quantifying heterogeneity:
##  tau^2 = 0 [0.0000; 28.9209]; tau = 0 [0.0000; 5.3778]
##  I^2 = 0.0% [0.0%; 89.6%]; H = 1.00 [1.00; 3.10]
##
## Test of heterogeneity:
##     Q d.f. p-value
##  1.03    2  0.5974
##
## Details on meta-analytical method:
## - Mantel-Haenszel method
## - Inverse variance method
## - Restricted maximum-likelihood estimator for tau^2
## - Q-Profile method for confidence interval of tau^2 and tau
## - Continuity correction of 0.5 in studies with zero cell frequencies
```

10.17.2 Forest plot

```r
forest(clav_i_metabin,
       sortvar = TE)
```

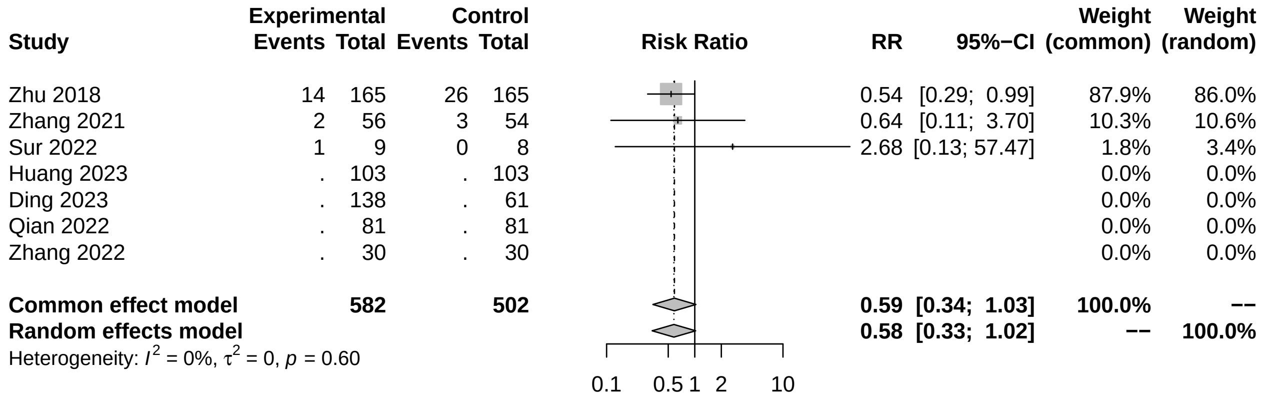

10.17.3 Trim and Fill

```r
trimfill(clav_i_metabin)
```

```
## Warning in trimfill.meta(clav_i_metabin): 4 observation(s) dropped due to
## missing values
```

```
## Number of studies: k = 5 (with 2 added studies)
## Number of observations: o = 584
## Number of events: e = 52
##
##                          RR           95%-CI     z p-value
## Random effects model 0.5385 [0.3162; 0.9169] -2.28  0.0227
##
## Quantifying heterogeneity:
##  tau^2 = 0 [0.0000; 8.3636]; tau = 0 [0.0000; 2.8920]
##  I^2 = 0.0% [0.0%; 79.2%]; H = 1.00 [1.00; 2.19]
##
## Test of heterogeneity:
##     Q d.f. p-value
##  2.19    4  0.7007
##
## Details on meta-analytical method:
## - Inverse variance method
## - Restricted maximum-likelihood estimator for tau^2
## - Q-Profile method for confidence interval of tau^2 and tau
## - Trim-and-fill method to adjust for funnel plot asymmetry (L-estimator)
```

```r
forest(trimfill(clav_i_metabin),
       sortvar = TE)
```

```
## Warning in trimfill.meta(clav_i_metabin): 4 observation(s) dropped due to
## missing values
```

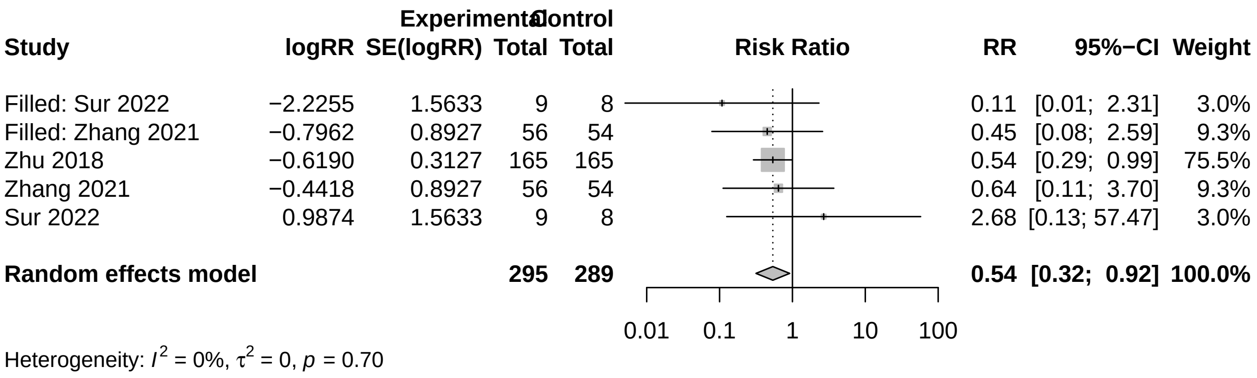

10.17.4 Funnel plot

```
funnel(trimfill(clav_i_metabin))
```

```
## Warning in trimfill.meta(clav_i_metabin): 4 observation(s) dropped due to
## missing values
```

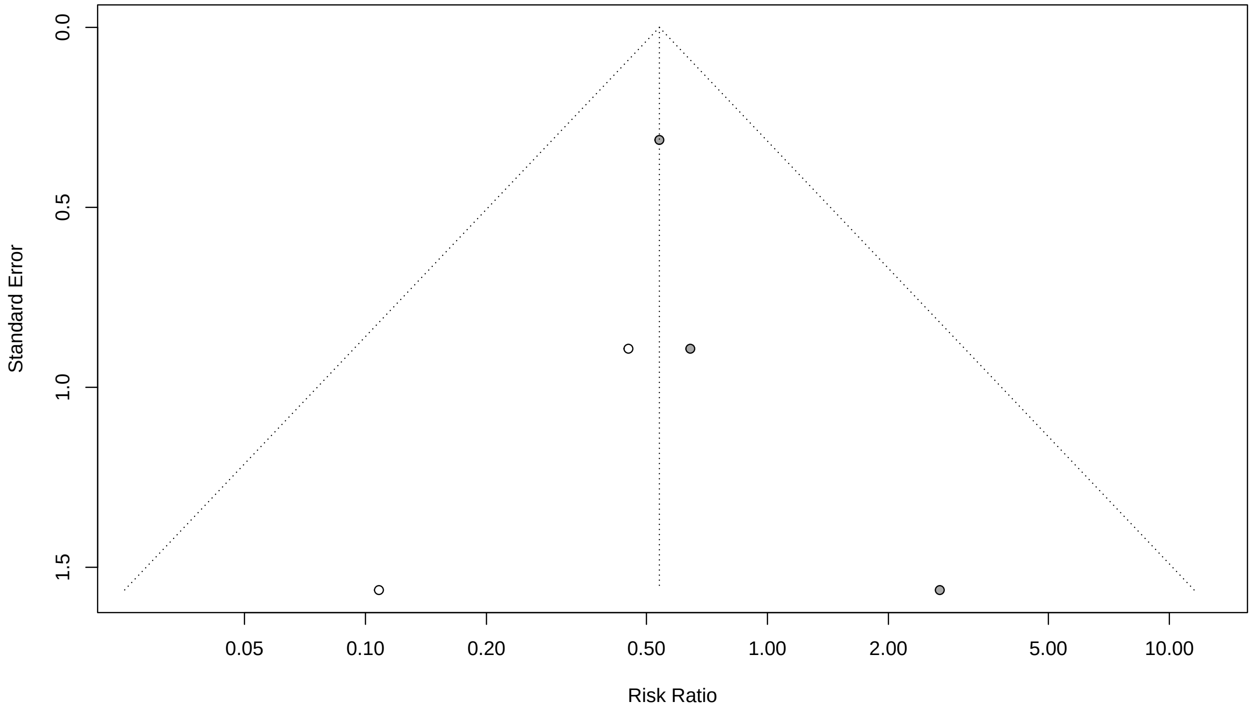

10.17.5 Baujat

```
baujat(clav_i_metabin, pos = 1)
```

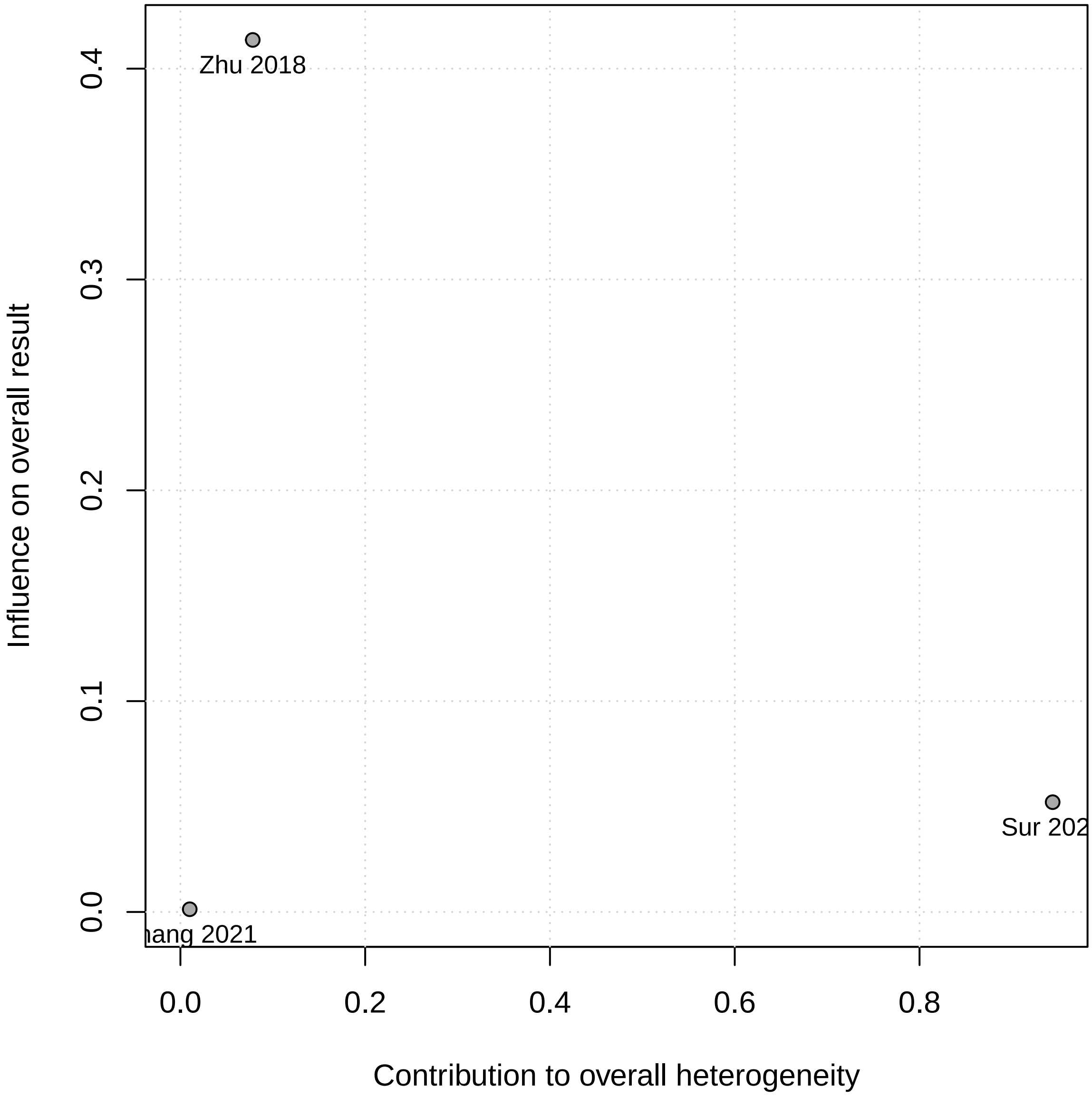

10.17.6 Leave one out

```
metainf(clav_i_metabin)
```

```
## Influential analysis (common effect model)
##
##                           RR           95%-CI p-value   tau^2    tau   I^2
## Omitting Sur 2022     0.5494 [0.3082; 0.9796]  0.0424  0.0000 0.0000  0.0%
## Omitting Zhu 2018     0.9452 [0.2221; 4.0222]  0.9392  0.0000 0.0000  0.0%
## Omitting Zhang 2021   0.5813 [0.3213; 1.0517]  0.0729  0.0195 0.1398  1.5%
## Omitting Huang 2023   0.5877 [0.3352; 1.0304]  0.0635  0.0000 0.0000  0.0%
## Omitting Ding 2023    0.5877 [0.3352; 1.0304]  0.0635  0.0000 0.0000  0.0%
## Omitting Qian 2022    0.5877 [0.3352; 1.0304]  0.0635  0.0000 0.0000  0.0%
## Omitting Zhang 2022   0.5877 [0.3352; 1.0304]  0.0635  0.0000 0.0000  0.0%
##
## Pooled estimate       0.5877 [0.3352; 1.0304]  0.0635  0.0000 0.0000  0.0%
##
## Details on meta-analytical method:
## - Mantel-Haenszel method
## - Restricted maximum-likelihood estimator for tau^2
```

10.18 Clavien II

10.18.1 Meta-analysis

```
clav_ii_metabin <- metabin(data = flexiurs_only,
                           event.c = clavien_ii_n_control,
                         n.c = sample_size_control,
                         event.e = clavien_ii_n_suction,
                         n.e = sample_size_suction,
                         studlab = author_year)
clav_ii_metabin
```

```
## Number of studies: k = 2
## Number of observations: o = 1084
## Number of events: e = 15
##
##                          RR           95%-CI     z p-value
## Common effect model  0.2762 [0.0861; 0.8853] -2.16  0.0304
## Random effects model 0.2763 [0.0863; 0.8847] -2.17  0.0303
##
## Quantifying heterogeneity:
##  tau^2 = 0; tau = 0; I^2 = 0.0%; H = 1.00
##
## Test of heterogeneity:
##     Q d.f. p-value
##  0.00    1  0.9578
##
## Details on meta-analytical method:
## - Mantel-Haenszel method
## - Inverse variance method
## - Restricted maximum-likelihood estimator for tau^2
## - Continuity correction of 0.5 in studies with zero cell frequencies
```

10.18.2 Forest plot

```r
forest(clav_ii_metabin,
       sortvar = TE)
```

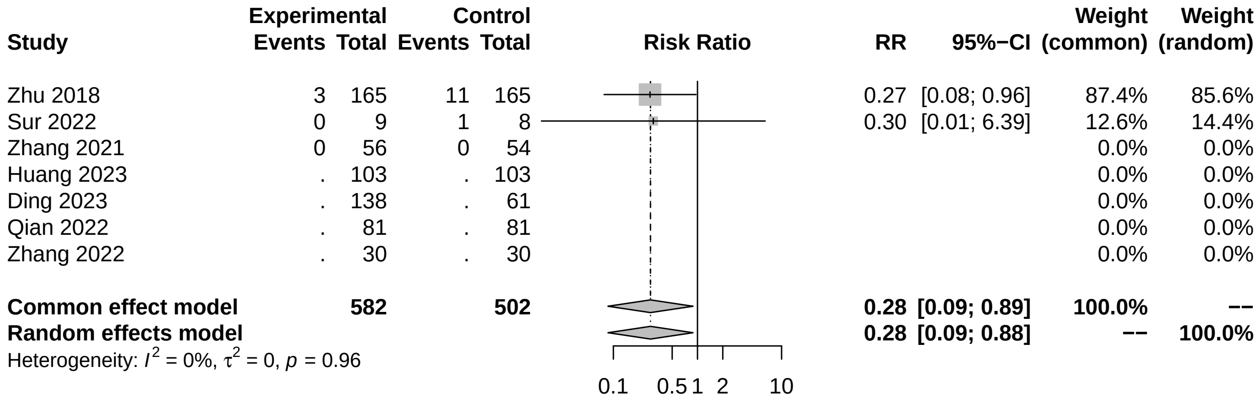

10.18.3 Trim and Fill

```r
#trimfill(clav_ii_metabin)
#forest(trimfill(clav_ii_metabin),
#       sortvar = TE)
```

10.18.4 Funnel plot

```
funnel((clav_ii_metabin))
```

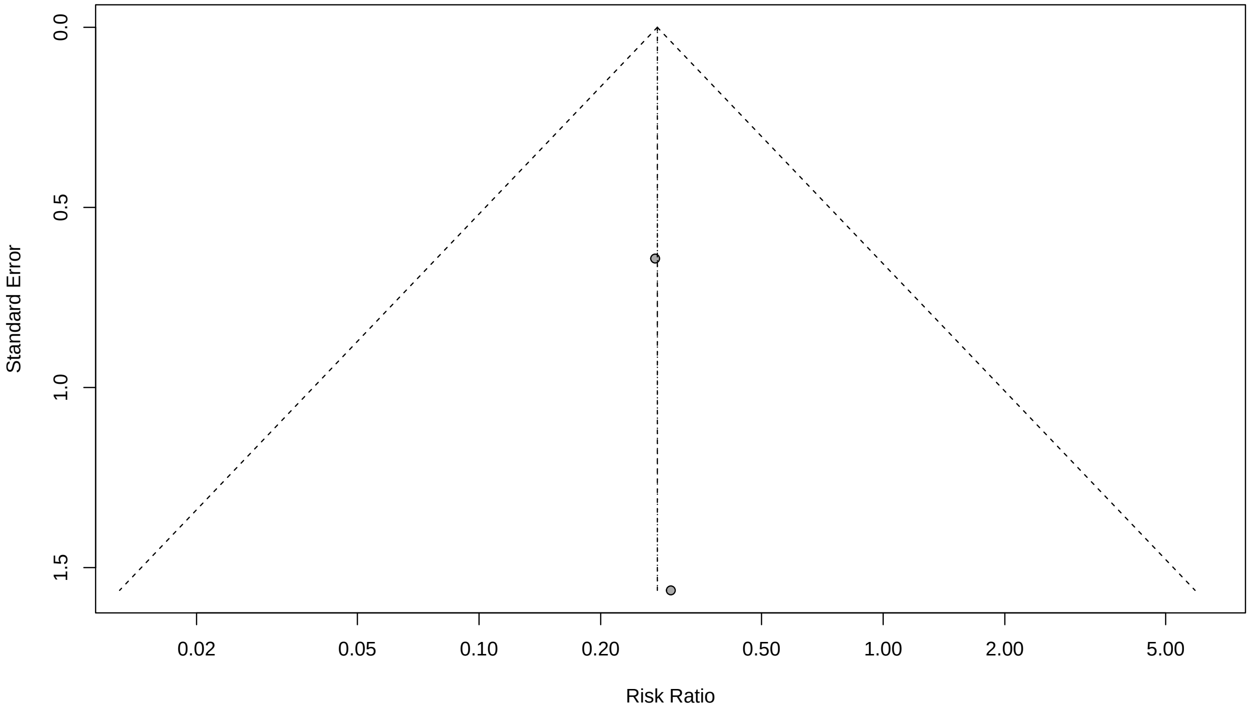

10.18.5 Baujat

```
baujat(clav_ii_metabin, pos = 1)
```

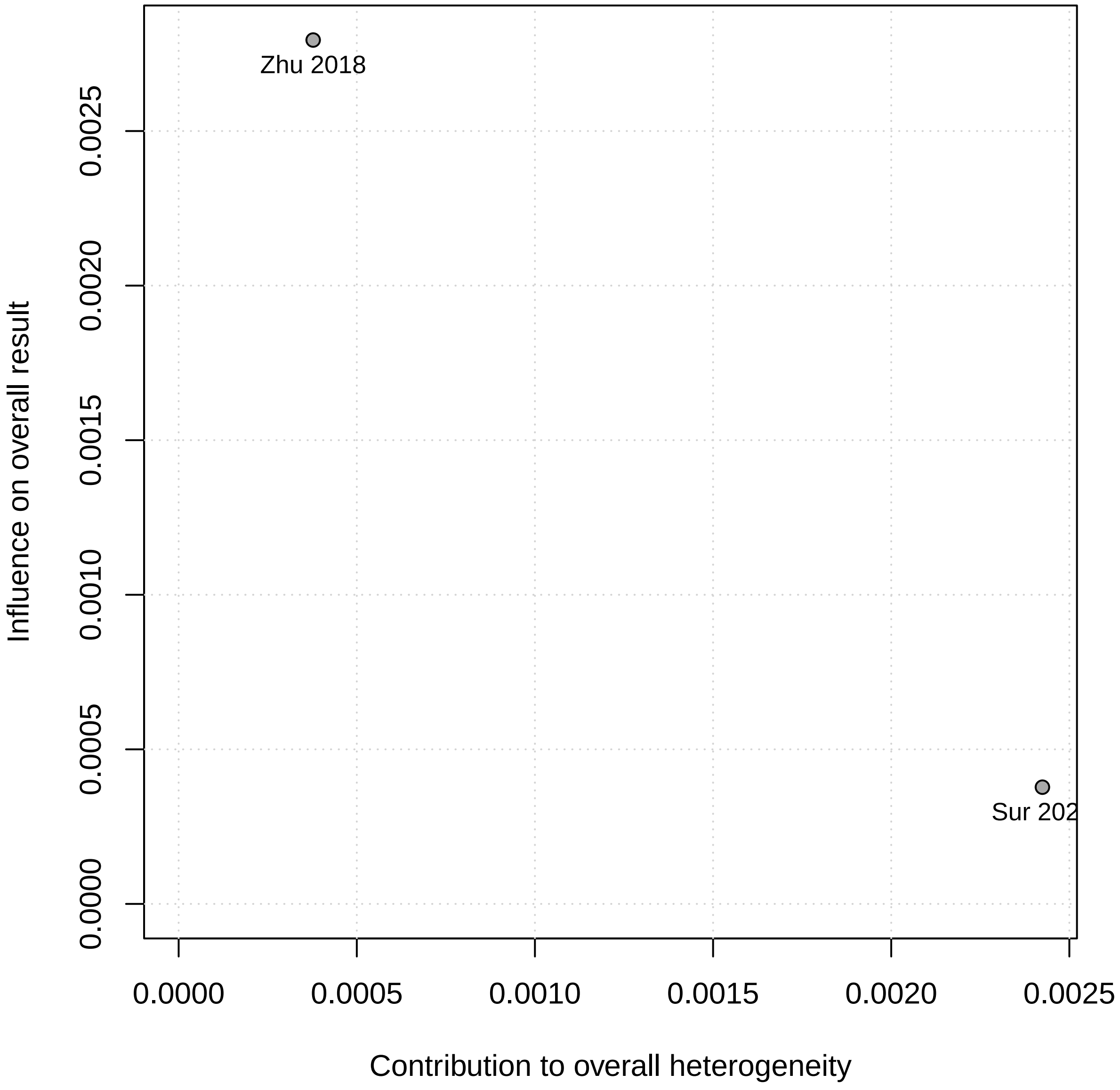

10.18.6 Leave one out

```
metainf(clav_ii_metabin)
```

```
## Influential analysis (common effect model)
##
##                            RR           95%-CI p-value   tau^2     tau   I^2
## Omitting Sur 2022      0.2727 [0.0775; 0.9598]  0.0430
## Omitting Zhu 2018      0.3000 [0.0139; 6.4710]  0.4423
## Omitting Zhang 2021    0.2762 [0.0861; 0.8853]  0.0304  0.0000  0.0000  0.0%
## Omitting Huang 2023    0.2762 [0.0861; 0.8853]  0.0304  0.0000  0.0000  0.0%
## Omitting Ding 2023     0.2762 [0.0861; 0.8853]  0.0304  0.0000  0.0000  0.0%
## Omitting Qian 2022     0.2762 [0.0861; 0.8853]  0.0304  0.0000  0.0000  0.0%
## Omitting Zhang 2022    0.2762 [0.0861; 0.8853]  0.0304  0.0000  0.0000  0.0%
##
## Pooled estimate        0.2762 [0.0861; 0.8853]  0.0304  0.0000  0.0000  0.0%
##
## Details on meta-analytical method:
## - Mantel-Haenszel method
## - Restricted maximum-likelihood estimator for tau^2
```

10.19 Clavien III

10.19.1 Meta-analysis

```
clav_iii_metabin <- metabin(data = flexiurs_only,
                         event.c = clavien_iii_n_control,
                       n.c = sample_size_control,
                       event.e = clavien_iii_n_suction,
                       n.e = sample_size_suction,
                       studlab = author_year)
clav_iii_metabin
```

```
## Number of studies: k = 1
## Number of observations: o = 1084
## Number of events: e = 2
##
##                              RR            95%-CI    z p-value
## Common effect model  1.0000 [0.0631; 15.8536] 0.00  1.0000
## Random effects model 1.0000 [0.0631; 15.8536] 0.00  1.0000
##
## Quantifying heterogeneity:
##  tau^2 = NA; tau = NA; I^2 = NA; H = NA
##
## Details on meta-analytical method:
## - Mantel-Haenszel method
## - Inverse variance method
## - Restricted maximum-likelihood estimator for tau^2
```

10.19.2 Forest plot

```r
forest(clav_iii_metabin,
       sortvar = TE)
```

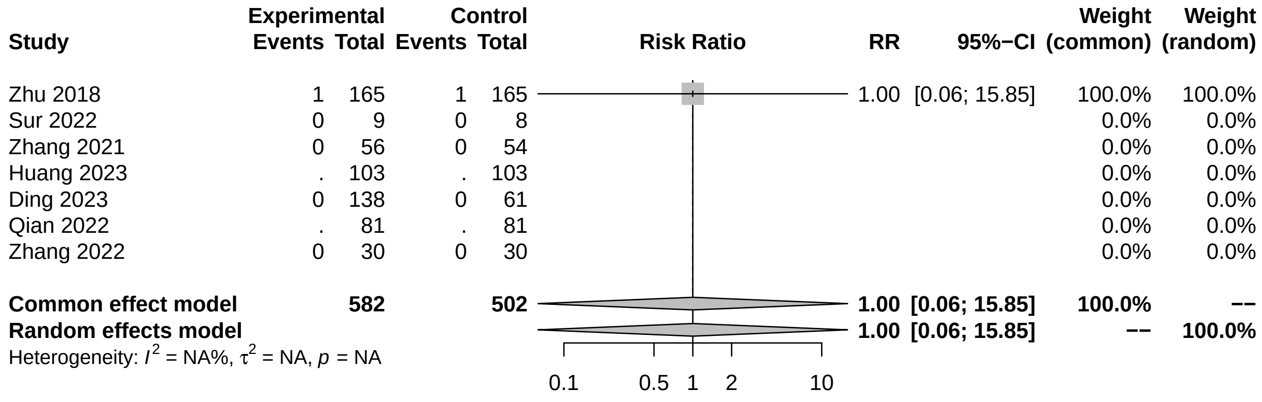

10.19.3 Trim and Fill

```
#trimfill(clav_iii_metabin)
#forest(trimfill(clav_iii_metabin),
#       sortvar = TE)
```

10.19.4 Funnel plot

```
#funnel(trimfill(clav_iii_metabin))
```

10.19.5 Baujat

```
#baujat(clav_iii_metabin, pos = 1)
```

10.19.6 Leave one out

```
#metainf(clav_iii_metabin)
```

10.20 Clavien IV

10.20.1 Meta-analysis

```
clav_iv_metabin <- metabin(data = flexiurs_only,
                         event.c = clavien_iv_n_control,
                       n.c = sample_size_control,
                       event.e = clavien_iv_n_suction,
                       n.e = sample_size_suction,
                       studlab = author_year)
clav_iv_metabin
```

```
## Number of studies: k = 1
## Number of observations: o = 1084
## Number of events: e = 4
##
##                           RR           95%-CI     z p-value
## Common effect model  0.3333 [0.0350; 3.1716] -0.96  0.3392
## Random effects model 0.3333 [0.0350; 3.1716] -0.96  0.3392
##
## Quantifying heterogeneity:
##  tau^2 = NA; tau = NA; I^2 = NA; H = NA
##
## Details on meta-analytical method:
## - Mantel-Haenszel method
## - Inverse variance method
## - Restricted maximum-likelihood estimator for tau^2
```

10.20.2 Forest plot

```r
forest(clav_iv_metabin,
       sortvar = TE)
```

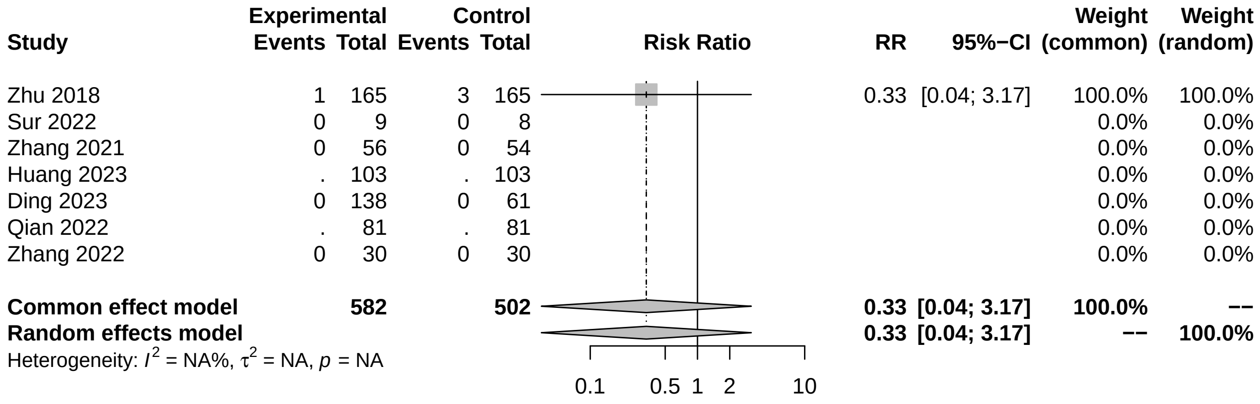

10.20.3 Trim and Fill

```r
#trimfill(clav_iv_metabin)
#forest(trimfill(clav_iv_metabin),
#       sortvar = TE)
```

10.20.4 Funnel plot

```
funnel((clav_iv_metabin))
```

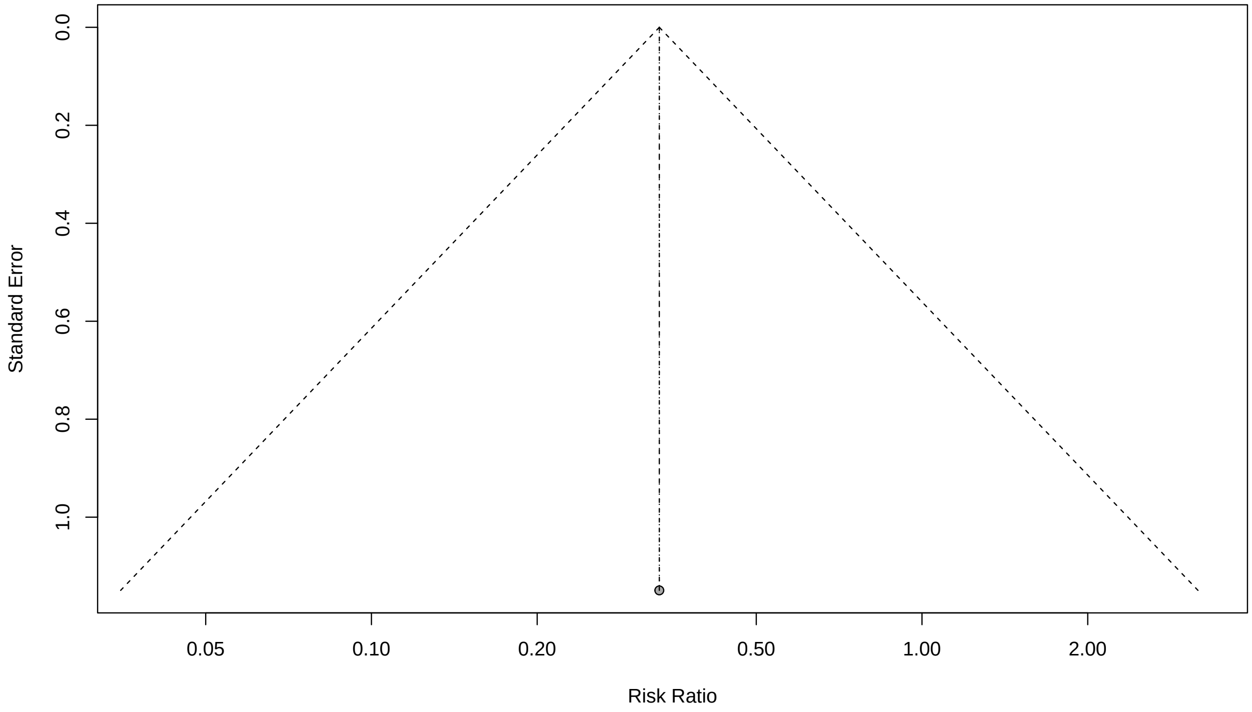

10.20.5 Baujat

```
baujat(clav_iv_metabin, pos = 1)
```

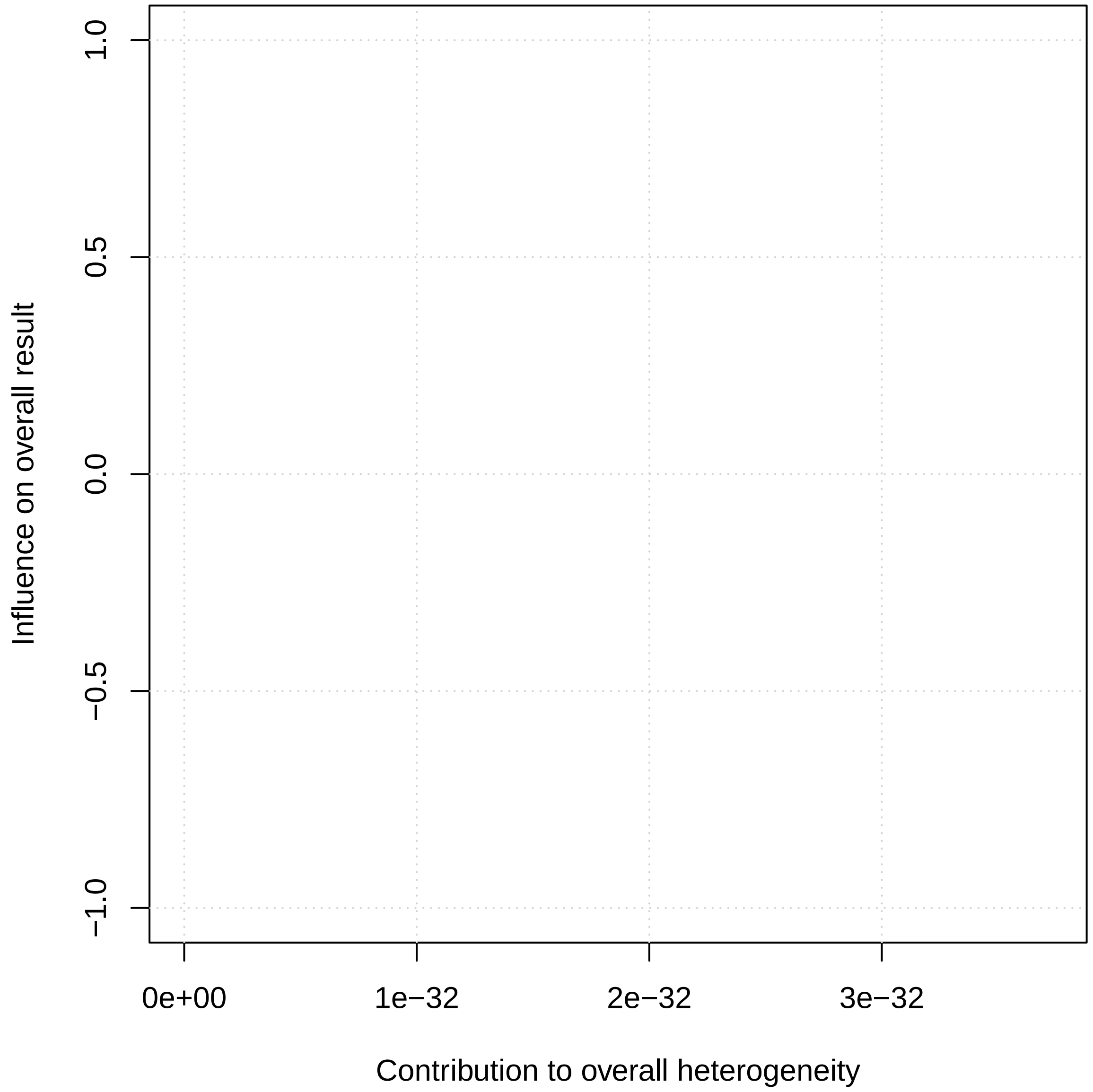

10.20.6 Leave one out

```r
metainf(clav_iv_metabin)
```

```
## Influential analysis (common effect model)
##
##                             RR           95%-CI p-value tau^2 tau I^2
## Omitting Sur 2022     0.3333 [0.0350; 3.1716]  0.3392
## Omitting Zhu 2018
## Omitting Zhang 2021   0.3333 [0.0350; 3.1716]  0.3392
## Omitting Huang 2023   0.3333 [0.0350; 3.1716]  0.3392
## Omitting Ding 2023    0.3333 [0.0350; 3.1716]  0.3392
## Omitting Qian 2022    0.3333 [0.0350; 3.1716]  0.3392
## Omitting Zhang 2022   0.3333 [0.0350; 3.1716]  0.3392
##
## Pooled estimate       0.3333 [0.0350; 3.1716]  0.3392
##
## Details on meta-analytical method:
## - Mantel-Haenszel method
## - Restricted maximum-likelihood estimator for tau^2
```

10.21 Clavien V

10.21.1 Meta-analysis

```
clav_v_metabin <- metabin(data = flexiurs_only,
                         event.c = clavien_v_n_control,
                       n.c = sample_size_control,
                       event.e = clavien_v_n_suction,
                       n.e = sample_size_suction,
                       studlab = author_year)
clav_v_metabin
```

```
## Number of studies: k = 0
## Number of observations: o = 1084
## Number of events: e = 0
##
##                        RR 95%-CI  z p-value
## Common effect model  NA         --      --
## Random effects model NA         --      --
##
## Quantifying heterogeneity:
##  tau^2 = NA; tau = NA; I^2 = NA; H = NA
##
## Details on meta-analytical method:
## - Mantel-Haenszel method
## - Inverse variance method
## - Restricted maximum-likelihood estimator for tau^2
```

10.21.2 Forest plot

```
#forest(clav_v_metabin,
#       sortvar = TE)
```

10.21.3 Trim and Fill

```
#trimfill(clav_v_metabin)
#forest(trimfill(clav_v_metabin),
#       sortvar = TE)
```

10.21.4 Funnel plot

```
#funnel(trimfill(clav_v_metabin))
```

10.21.5 Baujat

```
#baujat(clav_v_metabin, pos = 1)
```

10.21.6 Leave one out

```
#metainf(clav_v_metabin)
```

10.22 Clavien I-II

10.22.1 Meta-analysis

```
clav_i_ii_metabin <- metabin(data = flexiurs_only,
                         event.c = clav_i_ii_n_control,
                       n.c = sample_size_control,
                       event.e = clav_i_ii_n_suction,
                       n.e = sample_size_suction,
                       studlab = author_year)
clav_i_ii_metabin
```

```
## Number of studies: k = 3
## Number of observations: o = 1084
## Number of events: e = 61
##
##                           RR           95%-CI     z p-value
## Common effect model  0.4841 [0.2941; 0.7969] -2.85  0.0043
## Random effects model 0.4838 [0.2936; 0.7975] -2.85  0.0044
##
## Quantifying heterogeneity:
##  tau^2 = 0 [0.0000; 3.4477]; tau = 0 [0.0000; 1.8568]
##  I^2 = 0.0% [0.0%; 89.6%]; H = 1.00 [1.00; 3.10]
##
## Test of heterogeneity:
##     Q d.f. p-value
##  0.35    2  0.8406
##
## Details on meta-analytical method:
## - Mantel-Haenszel method
## - Inverse variance method
## - Restricted maximum-likelihood estimator for tau^2
## - Q-Profile method for confidence interval of tau^2 and tau
```

10.22.2 Forest plot

```r
forest(clav_i_ii_metabin,
       sortvar = TE)
```

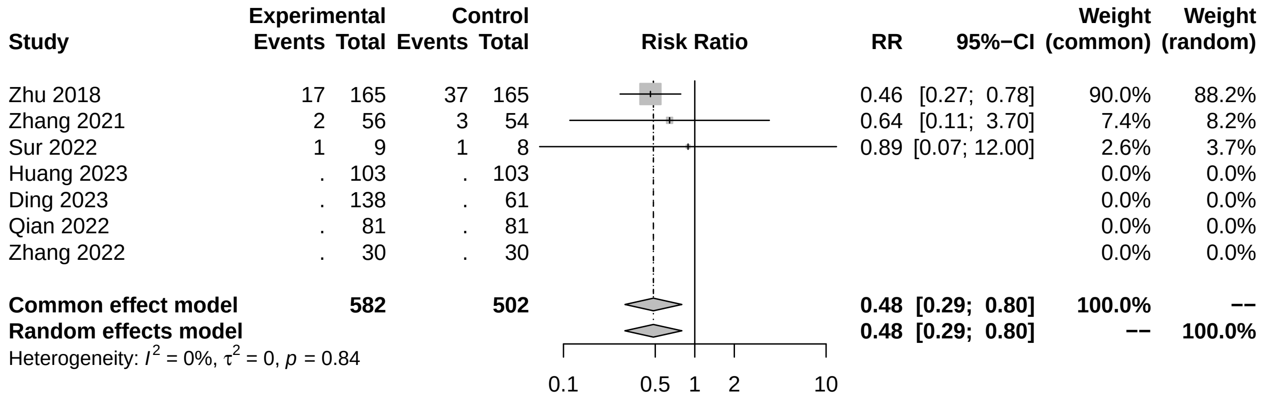

10.22.3 Trim and Fill

```r
trimfill(clav_i_ii_metabin)
```

```
## Warning in trimfill.meta(clav_i_ii_metabin): 4 observation(s) dropped due to
## missing values

## Number of studies: k = 5 (with 2 added studies)
## Number of observations: o = 584
## Number of events: e = 68
##
##                             RR           95%-CI     z p-value
## Random effects model 0.4595 [0.2864; 0.7370] -3.23  0.0013
##
## Quantifying heterogeneity:
##  tau^2 = 0 [0.0000; 0.7846]; tau = 0 [0.0000; 0.8858]
##  I^2 = 0.0% [0.0%; 79.2%]; H = 1.00 [1.00; 2.19]
##
## Test of heterogeneity:
##     Q d.f. p-value
##  0.78    4  0.9415
##
## Details on meta-analytical method:
## - Inverse variance method
## - Restricted maximum-likelihood estimator for tau^2
## - Q-Profile method for confidence interval of tau^2 and tau
## - Trim-and-fill method to adjust for funnel plot asymmetry (L-estimator)
```

```r
forest(trimfill(clav_i_ii_metabin),
       sortvar = TE)
```

```
## Warning in trimfill.meta(clav_i_ii_metabin): 4 observation(s) dropped due to
## missing values
```

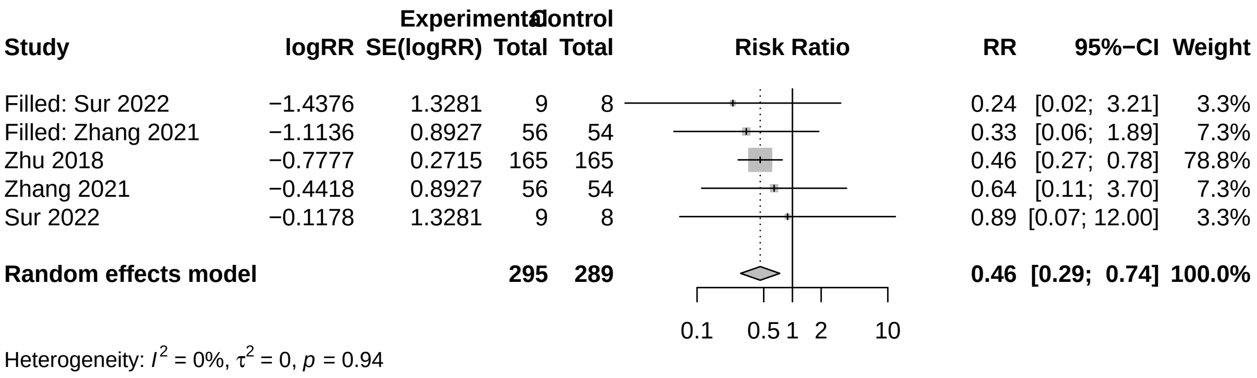

10.22.4 Funnel plot

```
funnel(trimfill(clav_i_ii_metabin))
```

```
## Warning in trimfill.meta(clav_i_ii_metabin): 4 observation(s) dropped due to
## missing values
```

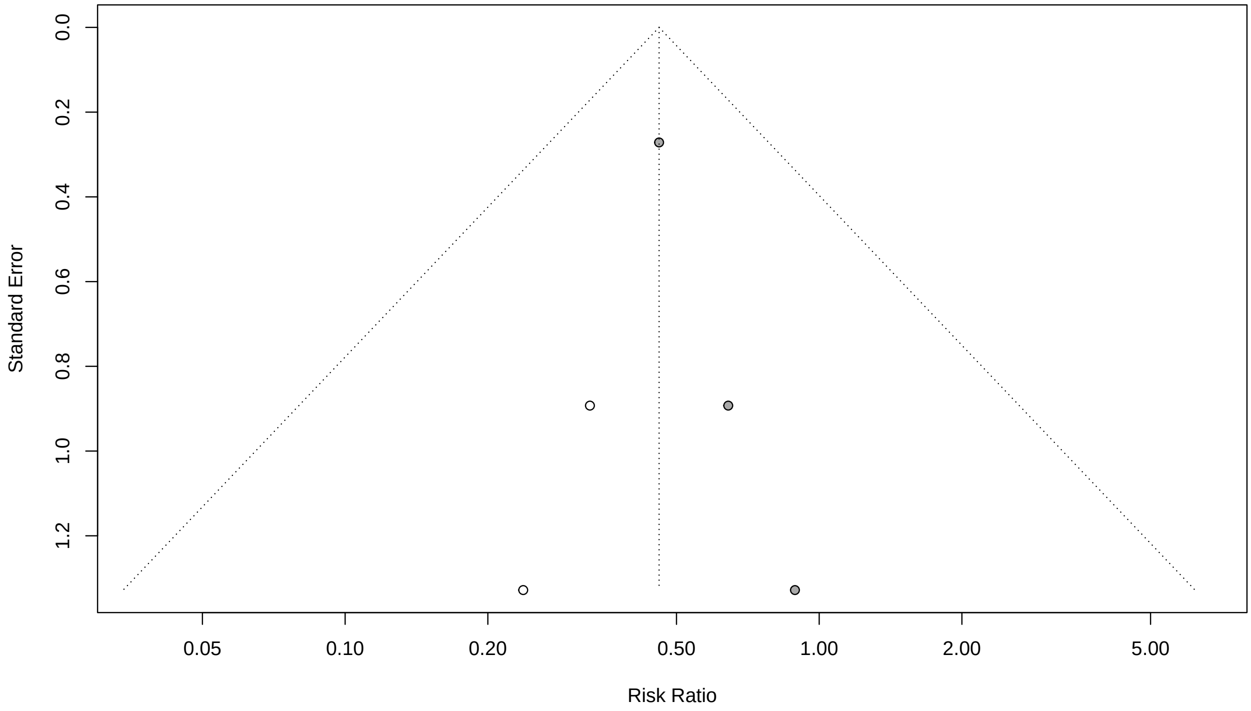

10.22.5 Baujat

```
baujat(clav_i_ii_metabin, pos = 1)
```

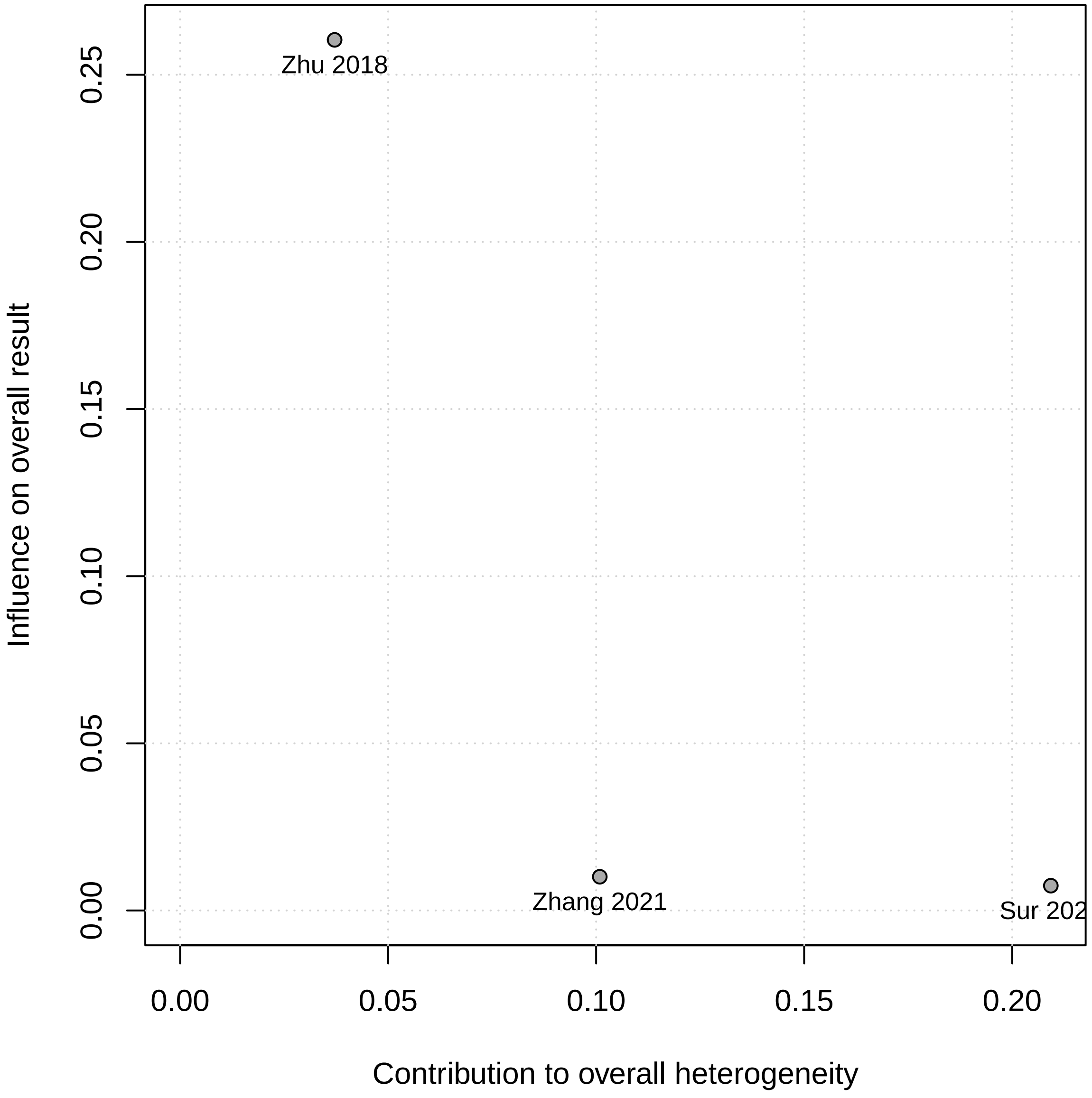

10.22.6 Leave one out

```
metainf(clav_i_ii_metabin)
```

```
## Influential analysis (common effect model)
##
##                              RR           95%-CI p-value   tau^2    tau   I^2
## Omitting Sur 2022       0.4734 [0.2847; 0.7874]  0.0040  0.0000 0.0000  0.0%
## Omitting Zhu 2018       0.7062 [0.1657; 3.0100]  0.6382  0.0000 0.0000  0.0%
## Omitting Zhang 2021     0.4714 [0.2802; 0.7932]  0.0046  0.0000 0.0000  0.0%
## Omitting Huang 2023     0.4841 [0.2941; 0.7969]  0.0043  0.0000 0.0000  0.0%
## Omitting Ding 2023      0.4841 [0.2941; 0.7969]  0.0043  0.0000 0.0000  0.0%
## Omitting Qian 2022      0.4841 [0.2941; 0.7969]  0.0043  0.0000 0.0000  0.0%
## Omitting Zhang 2022     0.4841 [0.2941; 0.7969]  0.0043  0.0000 0.0000  0.0%
##
## Pooled estimate         0.4841 [0.2941; 0.7969]  0.0043  0.0000 0.0000  0.0%
##
## Details on meta-analytical method:
## - Mantel-Haenszel method
## - Restricted maximum-likelihood estimator for tau^2
```

10.23 Clavien III-V

10.23.1 Meta-analysis

```
clav_iii_v_metabin <- metabin(data = flexiurs_only,
                          event.c = clav_iii_v_n_control,
                        n.c = sample_size_control,
                        event.e = clav_iii_v_n_suction,
                        n.e = sample_size_suction,
                        studlab = author_year)
clav_iii_v_metabin
```

```
## Number of studies: k = 1
## Number of observations: o = 1084
## Number of events: e = 6
##
##                             RR           95%-CI     z p-value
## Common effect model  0.5000 [0.0929; 2.6925] -0.81  0.4197
## Random effects model 0.5000 [0.0929; 2.6925] -0.81  0.4197
##
## Quantifying heterogeneity:
##  tau^2 = NA; tau = NA; I^2 = NA; H = NA
##
## Details on meta-analytical method:
## - Mantel-Haenszel method
## - Inverse variance method
## - Restricted maximum-likelihood estimator for tau^2
```

10.23.2 Forest plot

```r
forest(clav_iii_v_metabin,
       sortvar = TE)
```

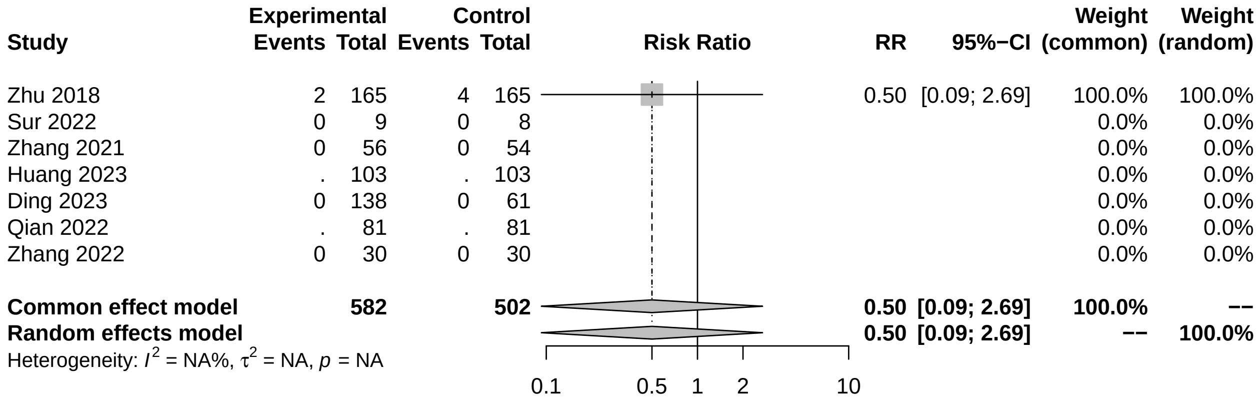

10.23.3 Trim and Fill

```
#trimfill(clav_iii_v_metabin)
#forest(trimfill(clav_iii_v_metabin),
#       sortvar = TE)
```

10.23.4 Funnel plot

```
funnel((clav_iii_v_metabin))
```

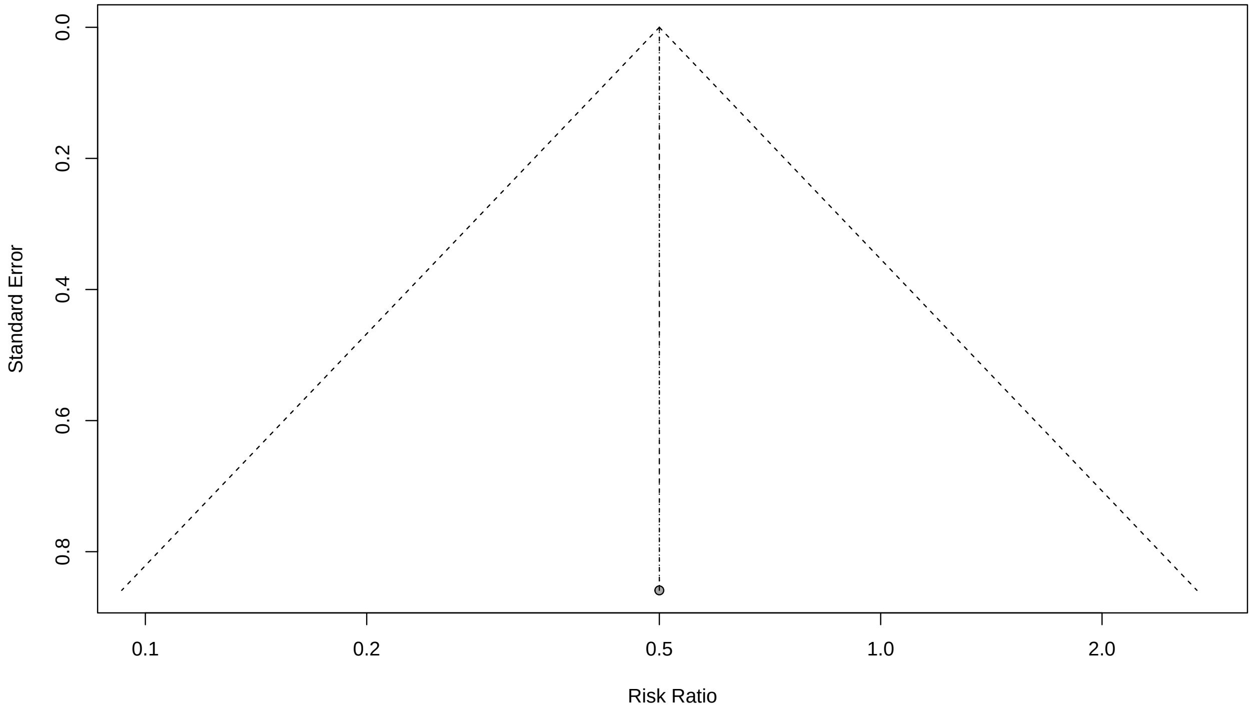

10.23.5 Baujat

```
baujat(clav_iii_v_metabin, pos = 1)
```

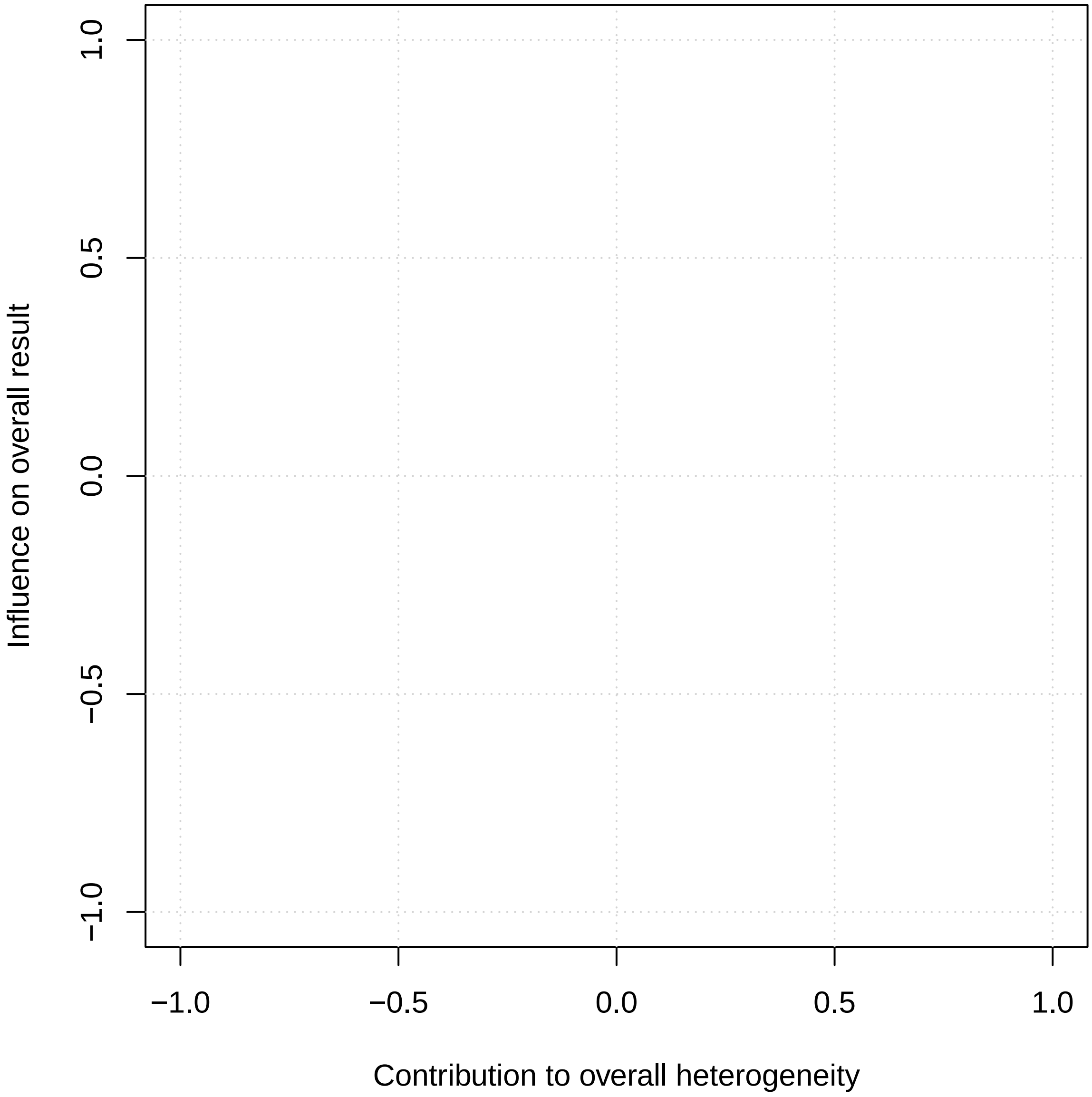

10.23.6 Leave one out

```r
metainf(clav_iii_v_metabin)
```

```
## Influential analysis (common effect model)
##
##                           RR           95%-CI p-value tau^2 tau I^2
## Omitting Sur 2022     0.5000 [0.0929; 2.6925]  0.4197
## Omitting Zhu 2018
## Omitting Zhang 2021   0.5000 [0.0929; 2.6925]  0.4197
## Omitting Huang 2023   0.5000 [0.0929; 2.6925]  0.4197
## Omitting Ding 2023    0.5000 [0.0929; 2.6925]  0.4197
## Omitting Qian 2022    0.5000 [0.0929; 2.6925]  0.4197
## Omitting Zhang 2022   0.5000 [0.0929; 2.6925]  0.4197
##
## Pooled estimate       0.5000 [0.0929; 2.6925]  0.4197
##
## Details on meta-analytical method:
## - Mantel-Haenszel method
## - Restricted maximum-likelihood estimator for tau^2
```

11 Flexi URS only Summary Forest plots

```
overall <- cbind(
  "Outcome" = c(
    "Immediate SFR",
    "Final SFR",
    "Auxiliary Treatment",
    "Overall Complications",
    "Fever",
    "Infection",
    "Sepsis",
    "Abscess",
    "Haematoma",
    "Pain",
    "Stricture",
    "Embolisation",
    "Transfusion",
    "CD I",
    "CD II",
    "CD III",
    "CD IV",
    "CD I-II",
    "CD III-V",
    "Operative time",
    "VAS",
    "Length of Stay"
  ),
  "type" = c(
    "binary",
    "binary",
    "binary",
    "binary",
    "binary",
    "binary",
    "binary",
    "binary",
    "binary",
    "binary",
    "binary",
    "binary",
    "binary",
    "binary",
    "binary",
    "binary",
    "binary",
    "binary",
    "binary",
    "cont",
    "cont",
    "cont"
  ),
  "n_studies" = c(
    immediate_sfr_metabin$k,
```

```
  sfr_metabin$k,
aux_rx_metabin$k,
comp_metabin$k,
fever_metabin$k,
infection_metabin$k,
sepsis_metabin$k,
abscess_metabin$k,
haematoma_metabin$k,
pain_metabin$k,
stricture_metabin$k,
ir_embolisation_metabin$k,
transfusion_metabin$k,
clav_i_metabin$k,
clav_ii_metabin$k,
clav_iii_metabin$k,
clav_iv_metabin$k,
clav_i_ii_metabin$k,
clav_iii_v_metabin$k,
or_time_metacont$k,
vas_metacont$k,
los_metacont$k),
"es" = c(
  immediate_sfr_metabin$TE.random %>% exp(),
  sfr_metabin$TE.random %>% exp(),
aux_rx_metabin$TE.random %>% exp(),
comp_metabin$TE.random %>% exp(),
fever_metabin$TE.random %>% exp(),
infection_metabin$TE.random %>% exp(),
sepsis_metabin$TE.random %>% exp(),
abscess_metabin$TE.random %>% exp(),
haematoma_metabin$TE.random %>% exp(),
pain_metabin$TE.random %>% exp(),
stricture_metabin$TE.random %>% exp(),
ir_embolisation_metabin$TE.random %>% exp(),
transfusion_metabin$TE.random %>% exp(),
clav_i_metabin$TE.random %>% exp(),
clav_ii_metabin$TE.random %>% exp(),
clav_iii_metabin$TE.random %>% exp(),
clav_iv_metabin$TE.random %>% exp(),
clav_i_ii_metabin$TE.random %>% exp(),
clav_iii_v_metabin$TE.random %>% exp(),
or_time_metacont$TE.random,
vas_metacont$TE.random,
los_metacont$TE.random),
"lower_ci" = c(
  immediate_sfr_metabin$lower.random %>% exp(),
  sfr_metabin$lower.random %>% exp(),
aux_rx_metabin$lower.random %>% exp(),
comp_metabin$lower.random %>% exp(),
fever_metabin$lower.random %>% exp(),
infection_metabin$lower.random %>% exp(),
sepsis_metabin$lower.random %>% exp(),
abscess_metabin$lower.random %>% exp(),
```

```
haematoma_metabin$lower.random %>% exp(),
pain_metabin$lower.random %>% exp(),
stricture_metabin$lower.random %>% exp(),
ir_embolisation_metabin$lower.random %>% exp(),
transfusion_metabin$lower.random %>% exp(),
clav_i_metabin$lower.random %>% exp(),
clav_ii_metabin$lower.random %>% exp(),
clav_iii_metabin$lower.random %>% exp(),
clav_iv_metabin$lower.random %>% exp(),
clav_i_ii_metabin$lower.random %>% exp(),
clav_iii_v_metabin$lower.random %>% exp(),
or_time_metacont$lower.random,
vas_metacont$lower.random,
los_metacont$lower.random),
"upper_ci" = c(immediate_sfr_metabin$upper.random %>% exp(),
               sfr_metabin$upper.random %>% exp(),
aux_rx_metabin$upper.random %>% exp(),
comp_metabin$upper.random %>% exp(),
fever_metabin$upper.random %>% exp(),
infection_metabin$upper.random %>% exp(),
sepsis_metabin$upper.random %>% exp(),
abscess_metabin$upper.random %>% exp(),
haematoma_metabin$upper.random %>% exp(),
pain_metabin$upper.random %>% exp(),
stricture_metabin$upper.random %>% exp(),
ir_embolisation_metabin$upper.random %>% exp(),
transfusion_metabin$upper.random %>% exp(),
clav_i_metabin$upper.random %>% exp(),
clav_ii_metabin$upper.random %>% exp(),
clav_iii_metabin$upper.random %>% exp(),
clav_iv_metabin$upper.random %>% exp(),
clav_i_ii_metabin$upper.random %>% exp(),
clav_iii_v_metabin$upper.random %>% exp(),
or_time_metacont$upper.random,
vas_metacont$upper.random,
los_metacont$upper.random),
"p" = c(
  immediate_sfr_metabin$pval.random,
  sfr_metabin$pval.random,
aux_rx_metabin$pval.random,
comp_metabin$pval.random,
fever_metabin$pval.random,
infection_metabin$pval.random,
sepsis_metabin$pval.random,
abscess_metabin$pval.random,
haematoma_metabin$pval.random,
pain_metabin$pval.random,
stricture_metabin$pval.random,
ir_embolisation_metabin$pval.random,
transfusion_metabin$pval.random,
clav_i_metabin$pval.random,
clav_ii_metabin$pval.random,
clav_iii_metabin$pval.random,
```

```
clav_iv_metabin$pval.random,
clav_i_ii_metabin$pval.random,
clav_iii_v_metabin$pval.random,
or_time_metacont$pval.random,
vas_metacont$pval.random,
los_metacont$pval.random)) %>% as_tibble() %>% drop_na(es)

overall$es <- as.numeric(overall$es)
overall$es <- round(overall$es, digits = 2)

overall$lower_ci <- as.numeric(overall$lower_ci)
overall$lower_ci <- round(overall$lower_ci, digits = 2)

overall$upper_ci <- as.numeric(overall$upper_ci)
overall$upper_ci <- round(overall$upper_ci, digits = 2)

overall$p <- as.numeric(overall$p)
overall$p <- round(overall$p, digits = 2)
overall$p <- ifelse(overall$p<0.001, "<0.001", overall$p)
```

11.1 Summary Table of number of studies for each outcome included in meta-analysis

```
overall %>% subset(select = c(Outcome, n_studies)) %>% gt() %>% tab_header(title = "Summary table of Nu
```

Summary table of Number of Studies for Each Outcome Meta-Analyis

Outcome	Studies, n
Immediate SFR	9
Final SFR	7
Auxiliary Treatment	1
Overall Complications	7
Fever	6
Infection	7
Sepsis	1
Stricture	1
CD I	3
CD II	2
CD III	1
CD IV	1
CD I-II	3
CD III-V	1
Operative time	6
VAS	1
Length of Stay	2

11.2 Summary Forest plot of Continuous outcomes

11.2.1 Continuous Outcomes Table

md = mean difference lb = lower bound of 95% confidence interval ub = upper bound of 95% confidence interval tf = trim and fill

```
overall_continuous <-
  overall %>% subset(type ==  "cont") %>% subset(select = c(Outcome,
                                                             n_studies,
                                                             es,
                                                             lower_ci,
                                                             upper_ci,
                                                             p)) %>% as_tibble()
overall_continuous %>% gt() %>% tab_header(title = "Summary Table for Continuous Outcomes") %>% cols_me
```

Summary Table for Continuous Outcomes

Outcome	Studies, n	MD (95% CI)	p
Operative time	6	-4.31 (-8.61-0.00)	0.05
VAS	1	-0.34 (-0.65–0.03)	0.03
Length of Stay	2	-0.50 (-1.17-0.18)	0.15

11.2.2 Continuous Forest plot

```
continuous_plot <- overall_continuous %>%
  forestplot(
    mean = es,
    lower = lower_ci,
    upper = upper_ci,
    labeltext = c(Outcome, n_studies, es, lower_ci, upper_ci, p),
  zero = 0,
  vertices = TRUE,
  title = "A. Forest plot of MA Outcomes for Continuous Outcomes",
  clip = c(-4, 2),
  xlab = "MD [95% CI]",
  graph.pos = 3
) %>% fp_set_style(
    box = c("black"),
    line = "black",
    txt_gp = fpTxtGp(
      ticks = gpar(fontfamily = "", cex = 1),
      xlab  = gpar(fontfamily = "", cex = 1)
    )) %>% fp_add_lines("steelblue") %>%
  fp_add_header("Outcome",
                "Studies, n",
                  "MD",
                "[ 95%", " CI ]",
                "P-Value")  %>% fp_decorate_graph(box = TRUE) %>% fp_set_zebra_style("#EFEFEF")
continuous_plot
```

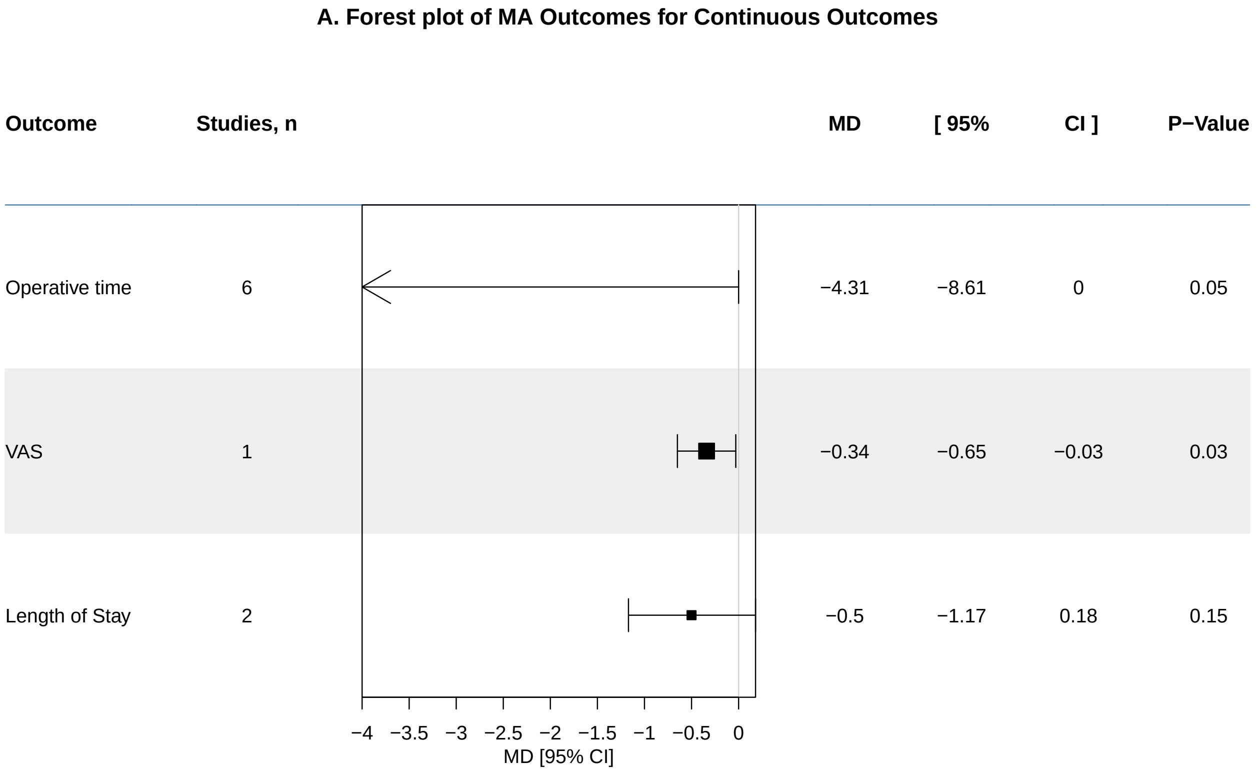

11.3 Summary Forest plot of Binary outcomes

11.3.1 Binary Outcomes Table

md = mean difference lb = lower bound of 95% confidence interval ub = upper bound of 95% confidence interval tf = trim and fill

```
overall_binary <-
  overall %>% subset(type ==  "binary") %>% subset(select = c(Outcome,
                                                            n_studies,
                                                            es,
                                                            lower_ci,
                                                            upper_ci,
                                                            p)) %>% as_tibble()
overall_binary %>% gt() %>% tab_header(title = "Summary Table for Binary Outcomes") %>% cols_merge(colu
```

Summary Table for Binary Outcomes

Outcome	Studies, n	RR (95% CI)	p
Immediate SFR	9	1.15 (0.99-1.34)	0.08
Final SFR	7	1.20 (1.10-1.30)	<0.001
Auxiliary Treatment	1	0.33 (0.01-8.12)	0.5
Overall Complications	7	0.44 (0.29-0.67)	<0.001
Fever	6	0.38 (0.23-0.65)	<0.001
Infection	7	0.38 (0.23-0.64)	<0.001
Sepsis	1	0.27 (0.08-0.96)	0.04
Stricture	1	3.00 (0.12-73.11)	0.5
CD I	3	0.58 (0.33-1.02)	0.06
CD II	2	0.28 (0.09-0.88)	0.03
CD III	1	1.00 (0.06-15.85)	1
CD IV	1	0.33 (0.04-3.17)	0.34
CD I-II	3	0.48 (0.29-0.80)	<0.001
CD III-V	1	0.50 (0.09-2.69)	0.42

11.3.2 Binary Forest plot - Meta-Analysis

Reference = No Suction

```
binary_plot <- overall_binary %>%
  forestplot(
    mean = es,
    lower = lower_ci,
    upper = upper_ci,
    labeltext = c(Outcome, n_studies, es, lower_ci, upper_ci, p),
  zero = 1,
  vertices = TRUE,
  title = "A. Forest plot of MA Outcomes for Binary Outcomes",
  clip = c(-1.5, 1.5),
  xlab = "RR [95% CI]",
  graph.pos = 3
) %>% fp_set_style(
    box = c("black"),
    line = "black",
    txt_gp = fpTxtGp(
      ticks = gpar(fontfamily = "", cex = 1),
      xlab  = gpar(fontfamily = "", cex = 1)
    )) %>% fp_add_lines("steelblue") %>%
  fp_add_header("Outcome",
                "Studies, n",
                  "RR",
                "[ 95%", " CI ]",
                "P-Value")  %>% fp_decorate_graph(box = TRUE) %>% fp_set_zebra_style("#EFEFEF")
binary_plot
```

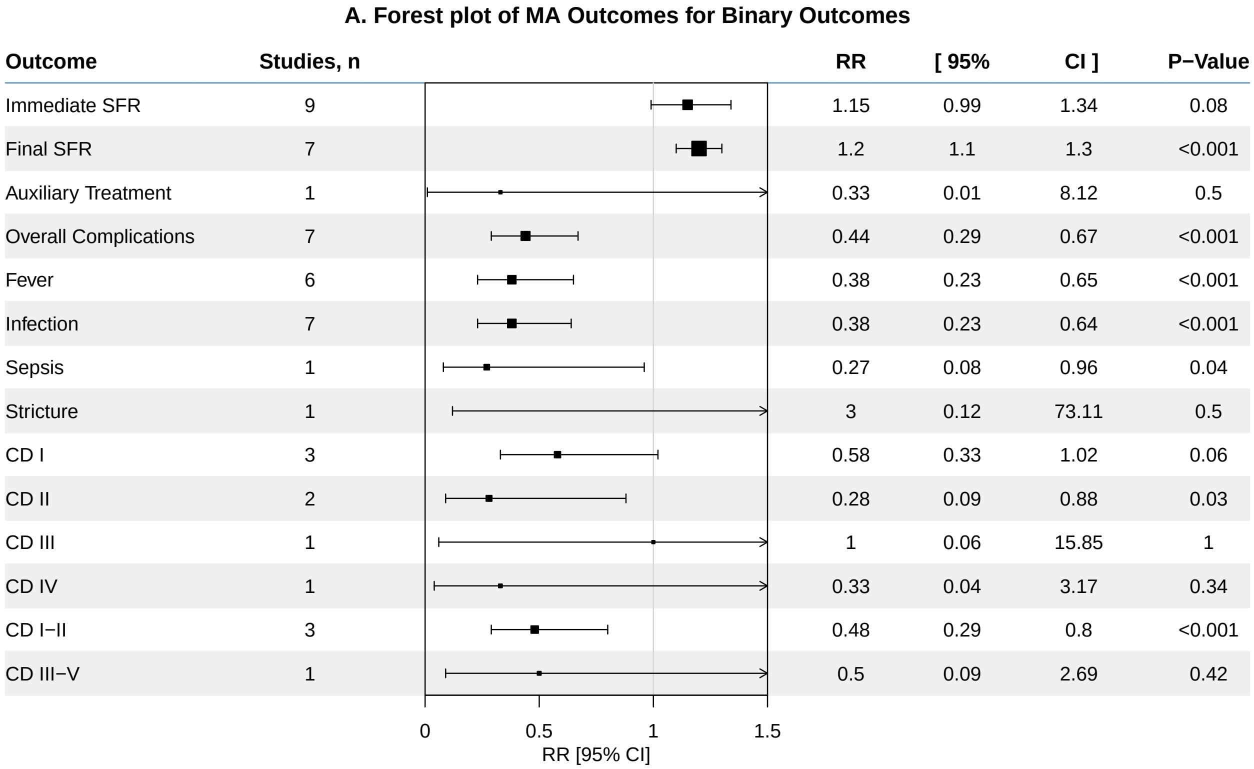

12 Mini-PCNL vs Flexi URS with Suction Meta-Analysis Outcomes

```
minipcnl_only <- suction_data %>% subset(control_group == "mini-PCNL ")
```

12.1 Immediate SFR

12.1.1 Meta-analysis

```
immediate_sfr_metabin <- metabin(data = suction_data,
                         event.c = sfr_immediate_n_control,
                       n.c = sample_size_control,
                       event.e = sfr_immediate_n_suction,
                       n.e = sample_size_suction,
                       studlab = author_year)
immediate_sfr_metabin
```

```
## Number of studies: k = 9
## Number of observations: o = 2311
## Number of events: e = 979
##
##                          RR           95%-CI    z  p-value
## Common effect model  1.1581 [1.0834; 1.2380] 4.31 < 0.0001
## Random effects model 1.1478 [0.9854; 1.3370] 1.77   0.0765
##
## Quantifying heterogeneity:
##  tau^2 = 0.0365 [0.0088; 0.2318]; tau = 0.1910 [0.0939; 0.4814]
##  I^2 = 74.6% [50.9%; 86.9%]; H = 1.99 [1.43; 2.76]
##
## Test of heterogeneity:
##      Q d.f. p-value
##  31.55    8  0.0001
##
## Details on meta-analytical method:
## - Mantel-Haenszel method
## - Inverse variance method
## - Restricted maximum-likelihood estimator for tau^2
## - Q-Profile method for confidence interval of tau^2 and tau
```

12.1.2 Forest plot

```r
forest(immediate_sfr_metabin,
       sortvar = TE)
```

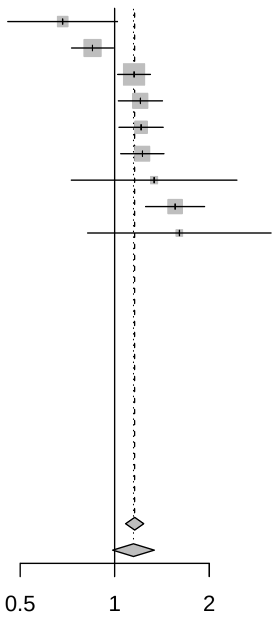

Study	Experimental Events	Experimental Total	Control Events	Control Total	RR	95%−CI	Weight (common)	Weight (random)
Lai 2020	14	28	41	56	0.68	[0.46; 1.02]	6.1%	7.7%
Tang 2023	63	86	75	87	0.85	[0.73; 0.99]	16.6%	14.2%
Zhu 2018	136	165	118	165	1.15	[1.02; 1.30]	26.3%	15.1%
Qian 2022	70	81	58	81	1.21	[1.03; 1.42]	12.9%	14.0%
AlSmadi 2019	40	43	46	60	1.21	[1.03; 1.43]	8.6%	14.0%
Wu 2022	67	76	59	82	1.23	[1.05; 1.44]	12.6%	14.1%
Zhang 2021	18	56	13	54	1.34	[0.73; 2.45]	2.9%	4.6%
Huang 2023	81	103	52	103	1.56	[1.25; 1.93]	11.6%	12.5%
Zhang 2021	18	56	10	50	1.61	[0.82; 3.15]	2.4%	3.9%
Sur 2022	.	9	.	8			0.0%	0.0%
Chen 2019	.	44	.	45			0.0%	0.0%
Du 2019	.	62	.	60			0.0%	0.0%
Lechevallier 2003	.	11	.	14			0.0%	0.0%
Ding 2023	.	138	.	61			0.0%	0.0%
Zhai 2023	.	60	.	60			0.0%	0.0%
Zhai 2023	.	60	.	60			0.0%	0.0%
Zhang 2022	.	30	.	30			0.0%	0.0%
Deng 2022	.	57	.	70			0.0%	0.0%
**Common effect model**		**1165**		**1146**	**1.16**	**[1.08; 1.24]**	**100.0%**	**--**
**Random effects model**					**1.15**	**[0.99; 1.34]**	**--**	**100.0%**

Heterogeneity: $I^2 = 75\%$, $\tau^2 = 0.0365$, $p < 0.01$

12.1.3 Trim and Fill

```r
trimfill(immediate_sfr_metabin)
```

```
## Warning in trimfill.meta(immediate_sfr_metabin): 9 observation(s) dropped due
## to missing values

## Number of studies: k = 11 (with 2 added studies)
## Number of observations: o = 1744
## Number of events: e = 1140
##
##                            RR           95%-CI     z p-value
## Random effects model 1.0855 [0.9289; 1.2685] 1.03  0.3019
##
## Quantifying heterogeneity:
##  tau^2 = 0.0471 [0.0138; 0.2196]; tau = 0.2170 [0.1173; 0.4686]
##  I^2 = 76.9% [58.8%; 87.1%]; H = 2.08 [1.56; 2.78]
##
## Test of heterogeneity:
##      Q d.f.  p-value
##  43.33   10 < 0.0001
##
## Details on meta-analytical method:
## - Inverse variance method
## - Restricted maximum-likelihood estimator for tau^2
## - Q-Profile method for confidence interval of tau^2 and tau
## - Trim-and-fill method to adjust for funnel plot asymmetry (L-estimator)
```

```r
forest(trimfill(immediate_sfr_metabin),
       sortvar = TE)
```

```
## Warning in trimfill.meta(immediate_sfr_metabin): 9 observation(s) dropped due
## to missing values
```

Study	logRR	SE(logRR)	Experimental Total	Control Total	RR	95%−CI	Weight
Lai 2020	−0.3814	0.2055	28	56	0.68	[0.46; 1.02]	7.1%
Filled: Zhang 2021	−0.2721	0.3431	56	50	0.76	[0.39; 1.49]	3.8%
Filled: Huang 2023	−0.2408	0.1103	103	103	0.79	[0.63; 0.98]	10.7%
Tang 2023	−0.1628	0.0780	86	87	0.85	[0.73; 0.99]	11.9%
Zhu 2018	0.1420	0.0609	165	165	1.15	[1.02; 1.30]	12.4%
Qian 2022	0.1881	0.0827	81	81	1.21	[1.03; 1.42]	11.7%
AlSmadi 2019	0.1934	0.0826	43	60	1.21	[1.03; 1.43]	11.7%
Wu 2022	0.2031	0.0808	76	82	1.23	[1.05; 1.44]	11.8%
Zhang 2021	0.2891	0.3100	56	54	1.34	[0.73; 2.45]	4.4%
Huang 2023	0.4432	0.1103	103	103	1.56	[1.25; 1.93]	10.7%
Zhang 2021	0.4745	0.3431	56	50	1.61	[0.82; 3.15]	3.8%
**Random effects model**			**853**	**891**	**1.09**	**[0.93; 1.27]**	**100.0%**

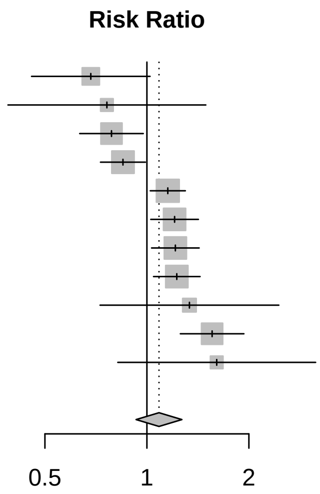

Heterogeneity: $I^2 = 77\%$, $\tau^2 = 0.0471$, $p < 0.01$

12.1.4 Funnel plot

```
funnel(trimfill(immediate_sfr_metabin))
```

```
## Warning in trimfill.meta(immediate_sfr_metabin): 9 observation(s) dropped due
## to missing values
```

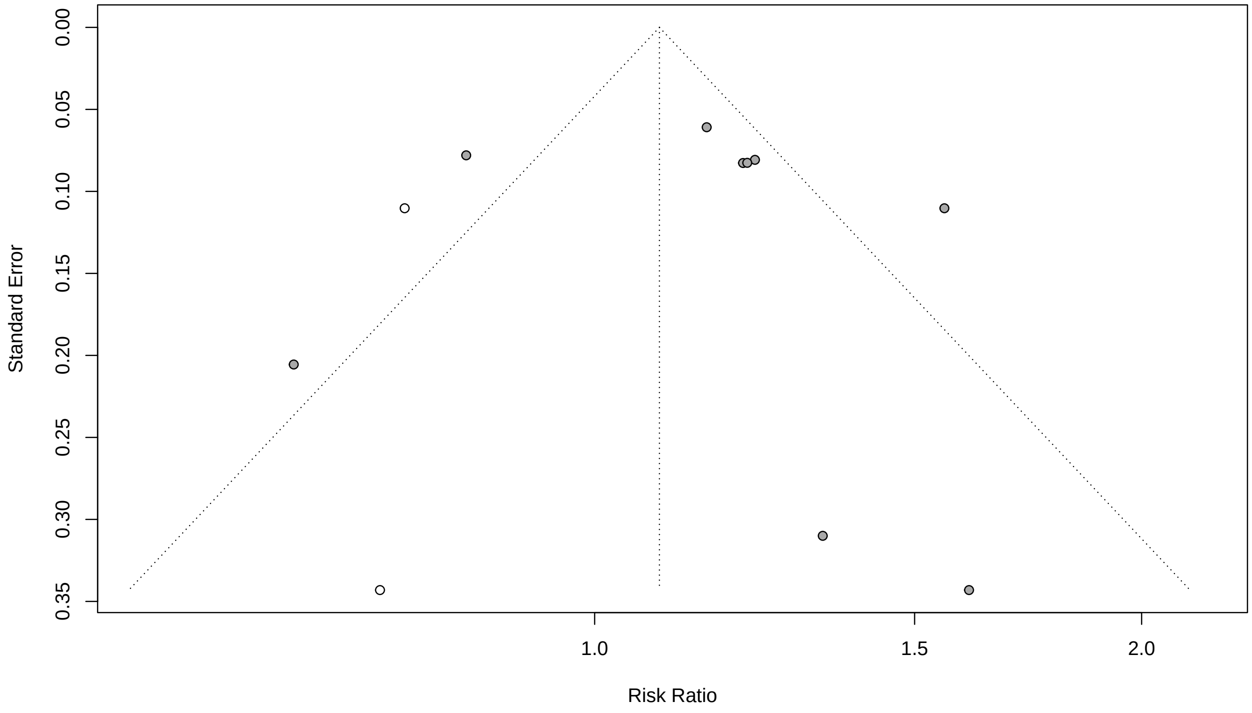

12.1.5 Baujat

```
baujat(immediate_sfr_metabin, pos = 1)
```

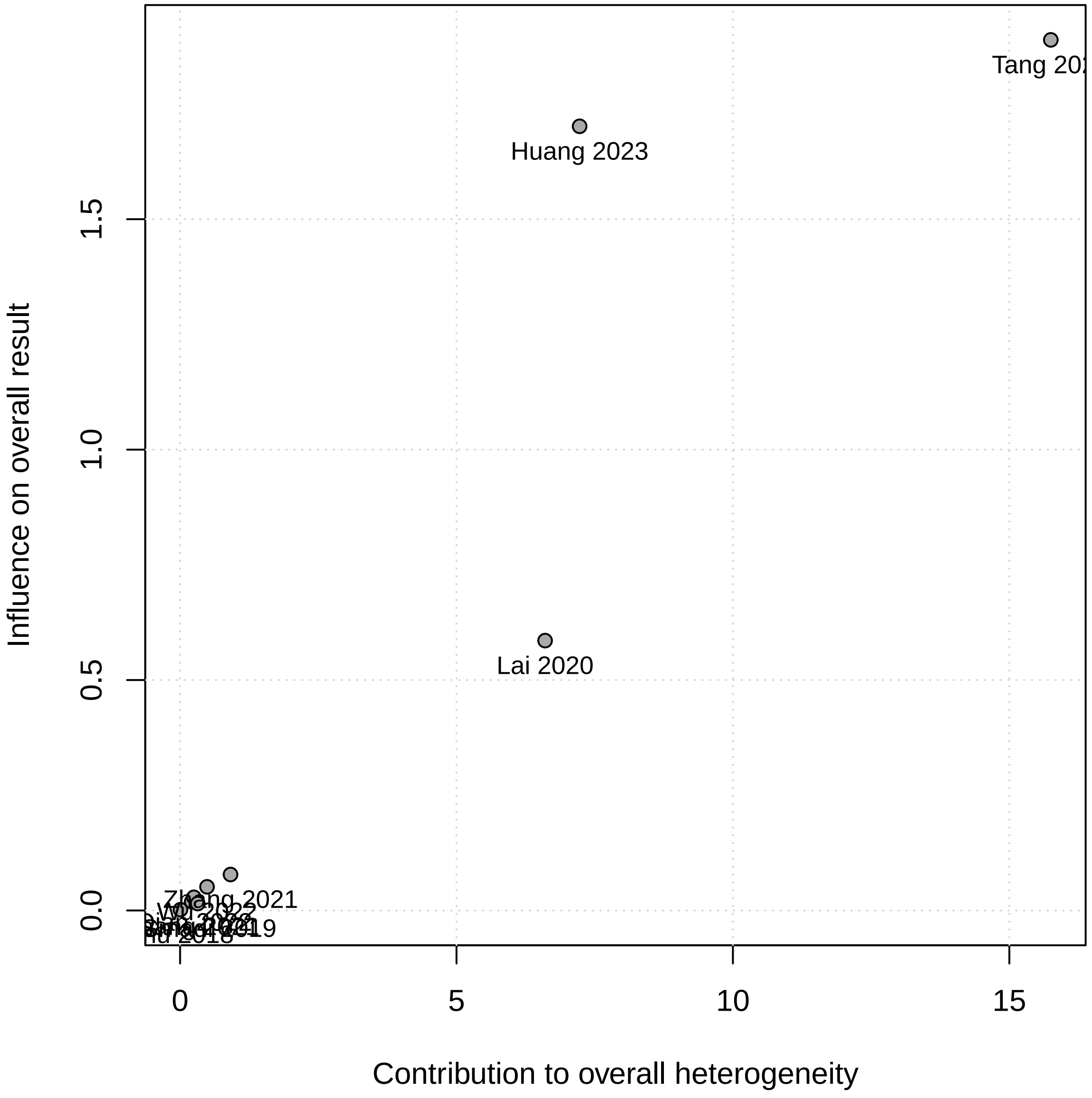

12.1.6 Leave one out

```r
metainf(immediate_sfr_metabin)
```

```
## Influential analysis (common effect model)
##
##                                  RR           95%-CI  p-value   tau^2     tau
## Omitting Sur 2022            1.1581 [1.0834; 1.2380] < 0.0001  0.0365  0.1910
## Omitting Tang 2023           1.2195 [1.1329; 1.3128] < 0.0001  0.0076  0.0873
## Omitting Chen 2019           1.1581 [1.0834; 1.2380] < 0.0001  0.0365  0.1910
## Omitting Du 2019             1.1581 [1.0834; 1.2380] < 0.0001  0.0365  0.1910
## Omitting Lai 2020            1.1889 [1.1116; 1.2716] < 0.0001  0.0258  0.1607
## Omitting Zhu 2018            1.1601 [1.0710; 1.2565]   0.0003  0.0482  0.2196
## Omitting Zhang 2021          1.1473 [1.0741; 1.2254] < 0.0001  0.0376  0.1938
## Omitting Zhang 2021          1.1527 [1.0791; 1.2313] < 0.0001  0.0396  0.1990
## Omitting Lechevallier 2003   1.1581 [1.0834; 1.2380] < 0.0001  0.0365  0.1910
## Omitting Huang 2023          1.1057 [1.0315; 1.1853]   0.0046  0.0246  0.1568
## Omitting Wu 2022             1.1484 [1.0676; 1.2353]   0.0002  0.0464  0.2153
## Omitting Ding 2023           1.1581 [1.0834; 1.2380] < 0.0001  0.0365  0.1910
## Omitting Zhai 2023           1.1581 [1.0834; 1.2380] < 0.0001  0.0365  0.1910
## Omitting Zhai 2023           1.1581 [1.0834; 1.2380] < 0.0001  0.0365  0.1910
## Omitting Qian 2022           1.1508 [1.0700; 1.2377]   0.0002  0.0468  0.2164
## Omitting Zhang 2022          1.1581 [1.0834; 1.2380] < 0.0001  0.0365  0.1910
## Omitting Deng 2022           1.1581 [1.0834; 1.2380] < 0.0001  0.0365  0.1910
## Omitting AlSmadi 2019        1.1529 [1.0734; 1.2383] < 0.0001  0.0467  0.2160
##
## Pooled estimate              1.1581 [1.0834; 1.2380] < 0.0001  0.0365  0.1910
##                                 I^2
## Omitting Sur 2022             74.6%
## Omitting Tang 2023            51.5%
## Omitting Chen 2019            74.6%
## Omitting Du 2019              74.6%
## Omitting Lai 2020             72.2%
## Omitting Zhu 2018             77.8%
## Omitting Zhang 2021           77.1%
## Omitting Zhang 2021           77.6%
## Omitting Lechevallier 2003    74.6%
## Omitting Huang 2023           69.4%
## Omitting Wu 2022              77.2%
## Omitting Ding 2023            74.6%
## Omitting Zhai 2023            74.6%
## Omitting Zhai 2023            74.6%
## Omitting Qian 2022            77.4%
## Omitting Zhang 2022           74.6%
## Omitting Deng 2022            74.6%
## Omitting AlSmadi 2019         77.4%
##
## Pooled estimate               74.6%
##
## Details on meta-analytical method:
## - Mantel-Haenszel method
## - Restricted maximum-likelihood estimator for tau^2
```

12.2 Final SFR

12.2.1 Meta-analysis

```
sfr_metabin <- metabin(data = minipcnl_only,
                         event.c = sfr_final_n_control,
                       n.c = sample_size_control,
                       event.e = sfr_final_n_suction,
                       n.e = sample_size_suction,
                       studlab = author_year)
sfr_metabin
```

```
## Number of studies: k = 4
## Number of observations: o = 473
## Number of events: e = 443
##
##                          RR           95%-CI     z p-value
## Common effect model  0.9669 [0.9210; 1.0151] -1.36  0.1754
## Random effects model 0.9694 [0.9243; 1.0166] -1.28  0.2003
##
## Quantifying heterogeneity:
##  tau^2 = 0 [0.0000; 0.0023]; tau = 0 [0.0000; 0.0482]
##  I^2 = 0.0% [0.0%; 84.7%]; H = 1.00 [1.00; 2.56]
##
## Test of heterogeneity:
##     Q d.f. p-value
##  0.50    3  0.9182
##
## Details on meta-analytical method:
## - Mantel-Haenszel method
## - Inverse variance method
## - Restricted maximum-likelihood estimator for tau^2
## - Q-Profile method for confidence interval of tau^2 and tau
```

12.2.2 Forest plot

```
forest(sfr_metabin,
       sortvar = TE)
```

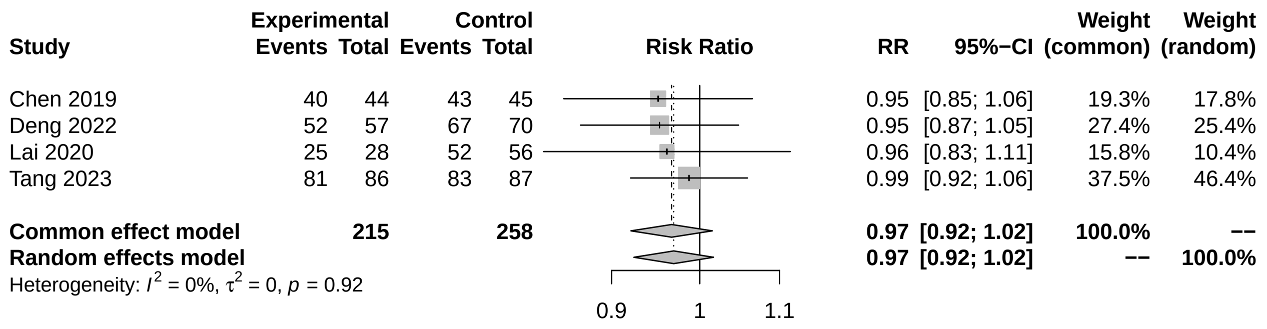

12.2.3 Trim and Fill

```r
trimfill(sfr_metabin)
```

```
## Number of studies: k = 6 (with 2 added studies)
## Number of observations: o = 689
## Number of events: e = 645
##
##                          RR           95%-CI     z p-value
## Random effects model 0.9825 [0.9442; 1.0224] -0.87  0.3839
##
## Quantifying heterogeneity:
##  tau^2 = 0 [0.0000; 0.0023]; tau = 0 [0.0000; 0.0476]
##  I^2 = 0.0% [0.0%; 74.6%]; H = 1.00 [1.00; 1.99]
##
## Test of heterogeneity:
##     Q d.f. p-value
##  1.52    5  0.9110
##
## Details on meta-analytical method:
## - Inverse variance method
## - Restricted maximum-likelihood estimator for tau^2
## - Q-Profile method for confidence interval of tau^2 and tau
## - Trim-and-fill method to adjust for funnel plot asymmetry (L-estimator)
```

```r
forest(trimfill(sfr_metabin),
       sortvar = TE)
```

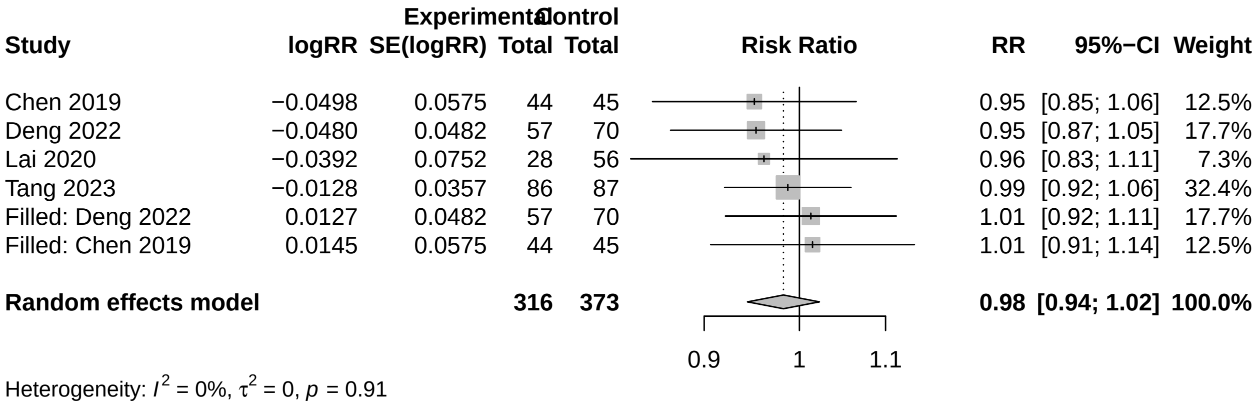

12.2.4 Funnel plot

```
funnel(trimfill(sfr_metabin))
```

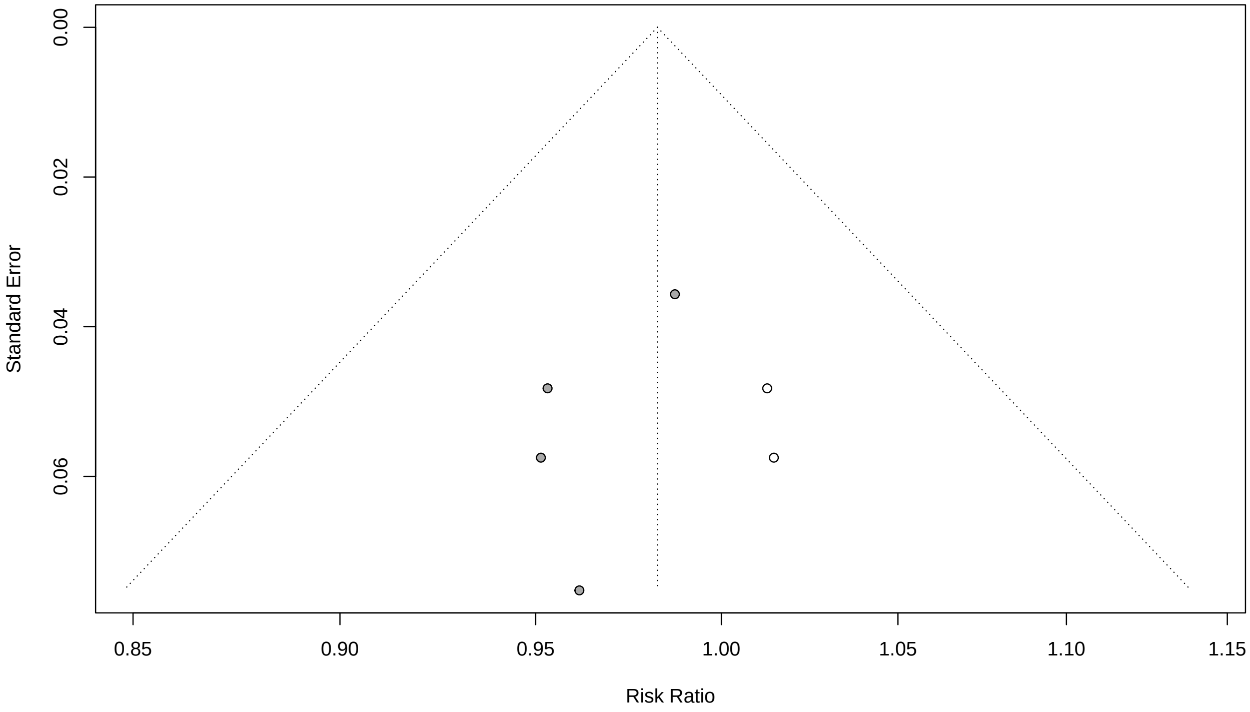

12.2.5 Baujat

```
baujat(sfr_metabin, pos = 1)
```

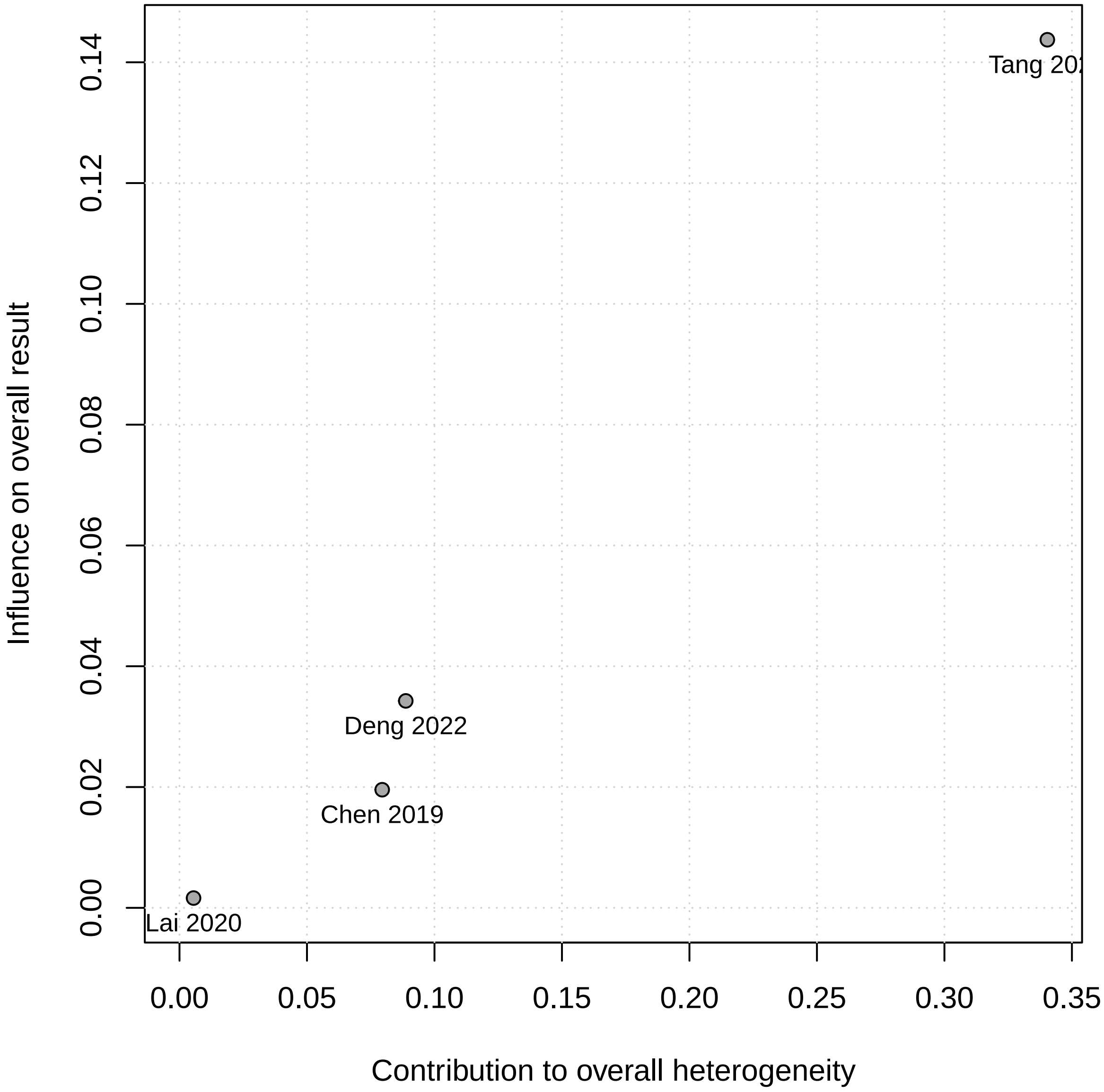

12.2.6 Leave one out

```r
metainf(sfr_metabin)
```

```
## Influential analysis (common effect model)
##
##                          RR           95%-CI p-value   tau^2    tau   I^2
## Omitting Tang 2023   0.9547 [0.8940; 1.0196]  0.1669  0.0000  0.0000  0.0%
## Omitting Chen 2019   0.9707 [0.9197; 1.0244]  0.2790  0.0000  0.0000  0.0%
## Omitting Lai 2020    0.9679 [0.9200; 1.0183]  0.2081  0.0000  0.0000  0.0%
## Omitting Deng 2022   0.9721 [0.9185; 1.0289]  0.3286  0.0000  0.0000  0.0%
##
## Pooled estimate      0.9669 [0.9210; 1.0151]  0.1754  0.0000  0.0000  0.0%
##
## Details on meta-analytical method:
## - Mantel-Haenszel method
## - Restricted maximum-likelihood estimator for tau^2
```

12.3 OR time

12.3.1 Meta-analysis

```
or_time_metacont <- metacont(data = minipcnl_only,
                             mean.c = or_time_min_mean_control,
                             sd.c = or_time_min_sd_control,
                             mean.e = or_time_min_mean_suction,
                             sd.e = or_time_min_sd_suction,
                             n.e = sample_size_suction,
                             n.c = sample_size_control,
                       studlab = author_year)
or_time_metacont
```

```
## Number of studies: k = 4
## Number of observations: o = 473
##
##                          MD            95%-CI    z p-value
## Common effect model  2.1825 [0.6472;  3.7178] 2.79  0.0053
## Random effects model 8.1512 [0.0481; 16.2544] 1.97  0.0487
##
## Quantifying heterogeneity:
##  tau^2 = 52.8557 [9.6858; >528.5572]; tau = 7.2702 [3.1122; >22.9904]
##  I^2 = 87.6% [70.5%; 94.8%]; H = 2.84 [1.84; 4.38]
##
## Test of heterogeneity:
##      Q d.f.  p-value
##  24.16    3 < 0.0001
##
## Details on meta-analytical method:
## - Inverse variance method
## - Restricted maximum-likelihood estimator for tau^2
## - Q-Profile method for confidence interval of tau^2 and tau
```

12.3.2 Forest plot

```r
forest(or_time_metacont,
       sortvar = TE)
```

Study	Experimental Total	Experimental Mean	Experimental SD	Control Total	Control Mean	Control SD	Mean Difference	MD	95%−CI	Weight (common)	Weight (random)
Tang 2023	86	61.40	5.2000	87	60.30	5.6000		1.10	[−0.51; 2.71]	90.9%	31.9%
Lai 2020	28	72.40	21.3000	56	67.40	25.0000		5.00	[−5.25; 15.25]	2.2%	21.3%
Chen 2019	44	65.60	22.5000	45	56.20	28.4000		9.40	[−1.23; 20.03]	2.1%	20.8%
Deng 2022	57	61.80	21.1000	70	43.40	18.9000		18.40	[11.36; 25.44]	4.8%	26.0%
**Common effect model**	**215**			**258**				**2.18**	**[0.65; 3.72]**	**100.0%**	**--**
**Random effects model**								**8.15**	**[0.05; 16.25]**	**--**	**100.0%**

Heterogeneity: $I^2 = 88\%$, $\tau^2 = 52.8557$, $p < 0.01$

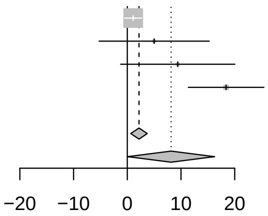

12.3.3 Trim and Fill

```
trimfill(or_time_metacont)
```

```
## Number of studies: k = 6 (with 2 added studies)
## Number of observations: o = 689
##
##                          MD              95%-CI    z p-value
## Random effects model 1.7739 [-8.1053; 11.6530] 0.35  0.7249
##
## Quantifying heterogeneity:
##  tau^2 = 134.4351 [42.0770; 872.9574]; tau = 11.5946 [6.4867; 29.5459]
##  I^2 = 90.2% [81.4%; 94.8%]; H = 3.19 [2.32; 4.40]
##
## Test of heterogeneity:
##      Q d.f.  p-value
##  50.97    5 < 0.0001
##
## Details on meta-analytical method:
## - Inverse variance method
## - Restricted maximum-likelihood estimator for tau^2
## - Q-Profile method for confidence interval of tau^2 and tau
## - Trim-and-fill method to adjust for funnel plot asymmetry (L-estimator)
```

```
forest(trimfill(or_time_metacont),
       sortvar = TE)
```

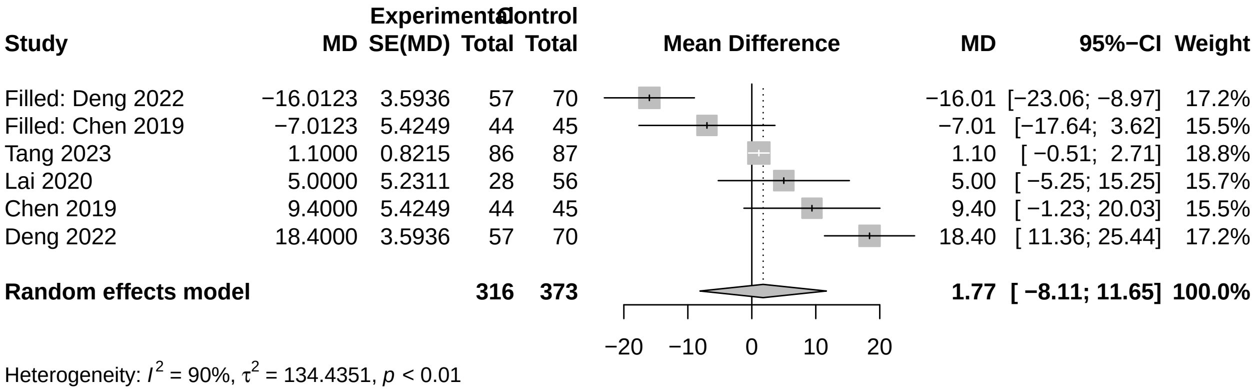

12.3.4 Funnel plot

```
funnel(trimfill(or_time_metacont))
```

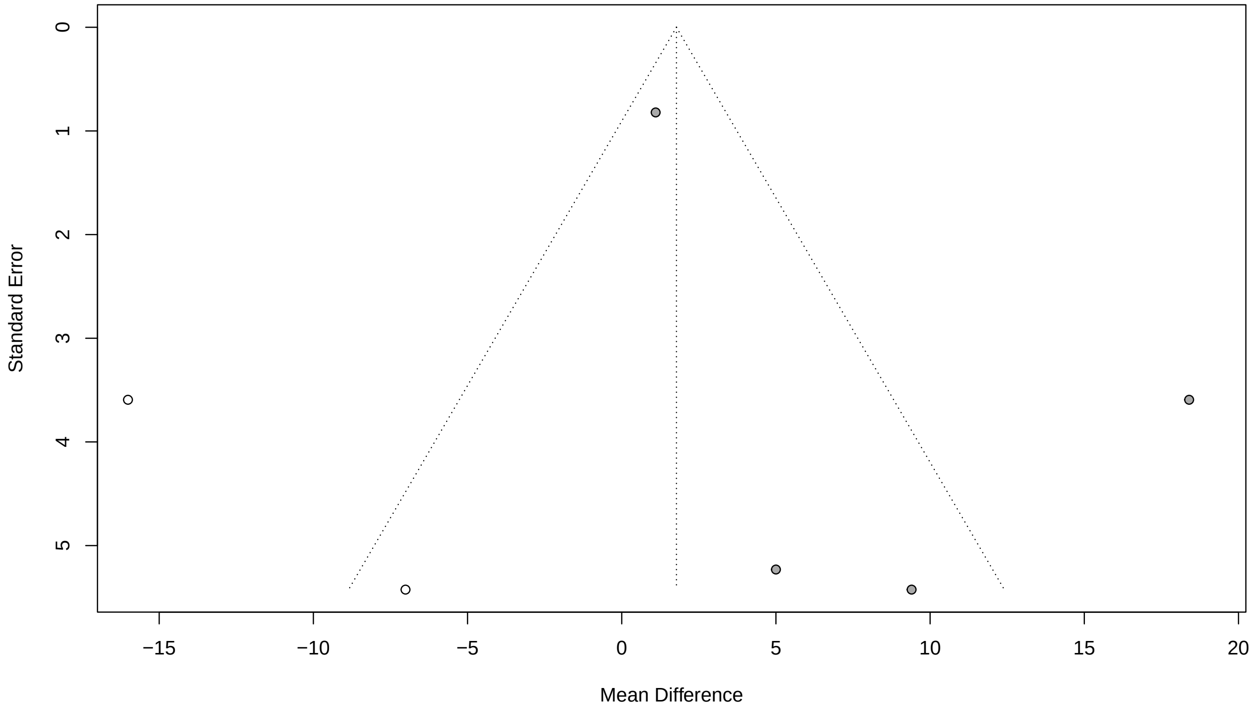

12.3.5 Baujat

```
baujat(or_time_metacont, pos = 1)
```

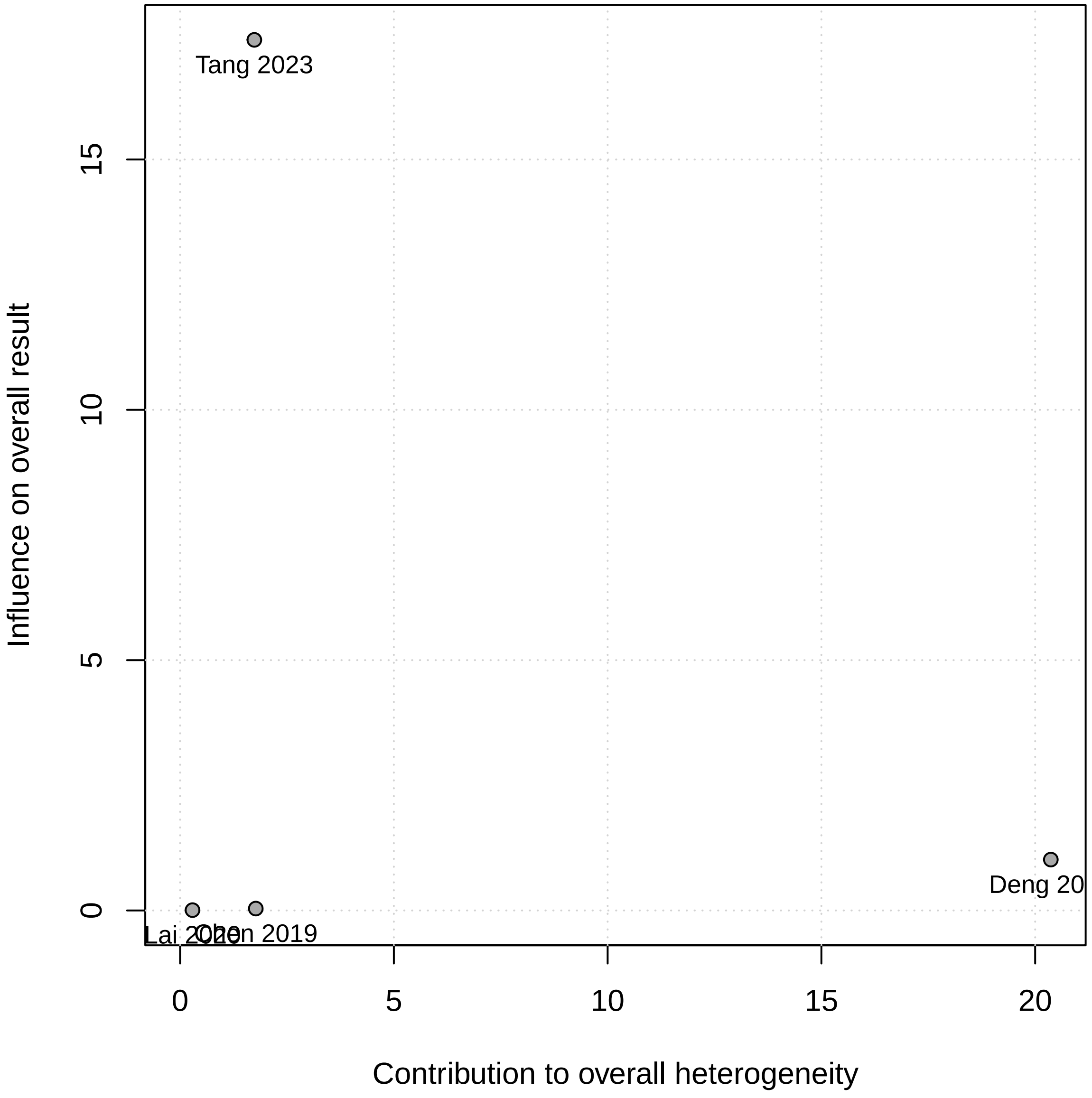

12.3.6 Leave one out

```
metainf(or_time_metacont)
```

```
## Influential analysis (common effect model)
##
##                          MD             95%-CI  p-value   tau^2    tau
## Omitting Tang 2023   13.0235 [ 7.9282; 18.1189] < 0.0001  31.8643  5.6448
## Omitting Chen 2019    2.0288 [ 0.4773;  3.5804]   0.0104  76.4762  8.7451
## Omitting Lai 2020     2.1179 [ 0.5651;  3.6707]   0.0075  74.4036  8.6258
## Omitting Deng 2022    1.3735 [-0.1996;  2.9466]   0.0870   7.8020  2.7932
##
## Pooled estimate       2.1825 [ 0.6472;  3.7178]   0.0053  52.8557  7.2702
##                        I^2
## Omitting Tang 2023    60.3%
## Omitting Chen 2019    91.1%
## Omitting Lai 2020     91.6%
## Omitting Deng 2022    28.1%
##
## Pooled estimate       87.6%
##
## Details on meta-analytical method:
## - Inverse variance method
## - Restricted maximum-likelihood estimator for tau^2
```

12.4 Auxiliary Treatments

12.4.1 Meta-analysis

```
aux_rx_metabin <- metabin(data = minipcnl_only,
                         event.c = auxillary_tx_n_control,
                       n.c = sample_size_control,
                       event.e = auxillary_tx_n_suction,
                       n.e = sample_size_suction,
                       studlab = author_year)
aux_rx_metabin
```

```
## Number of studies: k = 2
## Number of observations: o = 473
## Number of events: e = 34
##
##                          RR           95%-CI    z p-value
## Common effect model  1.7887 [1.0028; 3.1907] 1.97  0.0489
## Random effects model 1.7661 [1.0000; 3.1189] 1.96  0.0500
##
## Quantifying heterogeneity:
##  tau^2 = 0; tau = 0; I^2 = 0.0%; H = 1.00
##
## Test of heterogeneity:
##     Q d.f. p-value
##  0.05    1  0.8194
##
## Details on meta-analytical method:
## - Mantel-Haenszel method
## - Inverse variance method
## - Restricted maximum-likelihood estimator for tau^2
```

12.4.2 Forest plot

```r
forest(aux_rx_metabin,
       sortvar = TE)
```

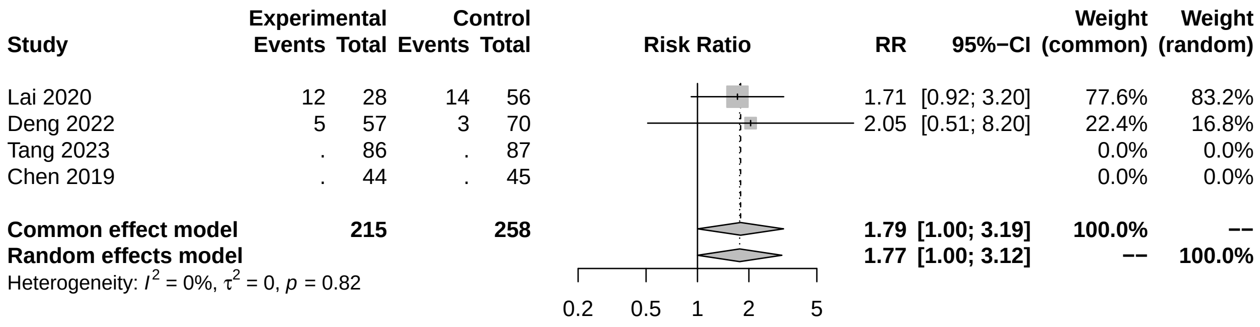

12.4.3 Trim and Fill

```
#trimfill(aux_rx_metabin)
#forest(trimfill(aux_rx_metabin),
#       sortvar = TE)
```

12.4.4 Funnel plot

```r
funnel((aux_rx_metabin))
```

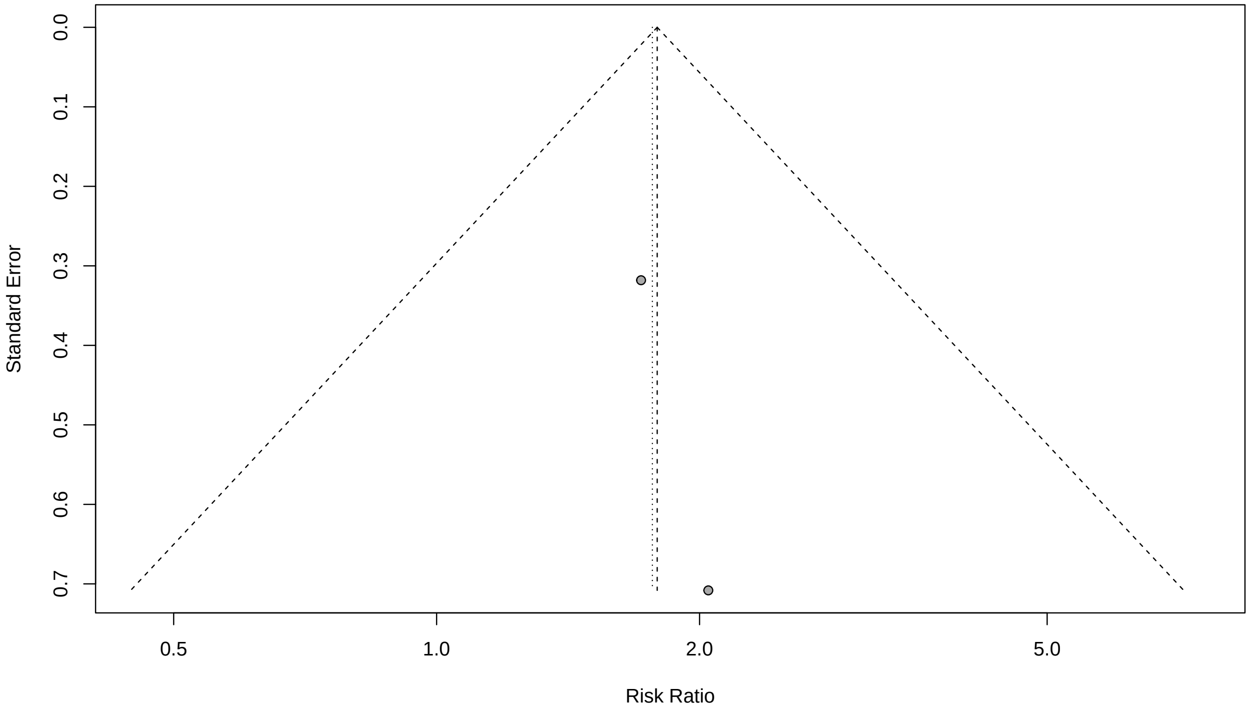

12.4.5 Baujat

```
baujat(aux_rx_metabin, pos = 1)
```

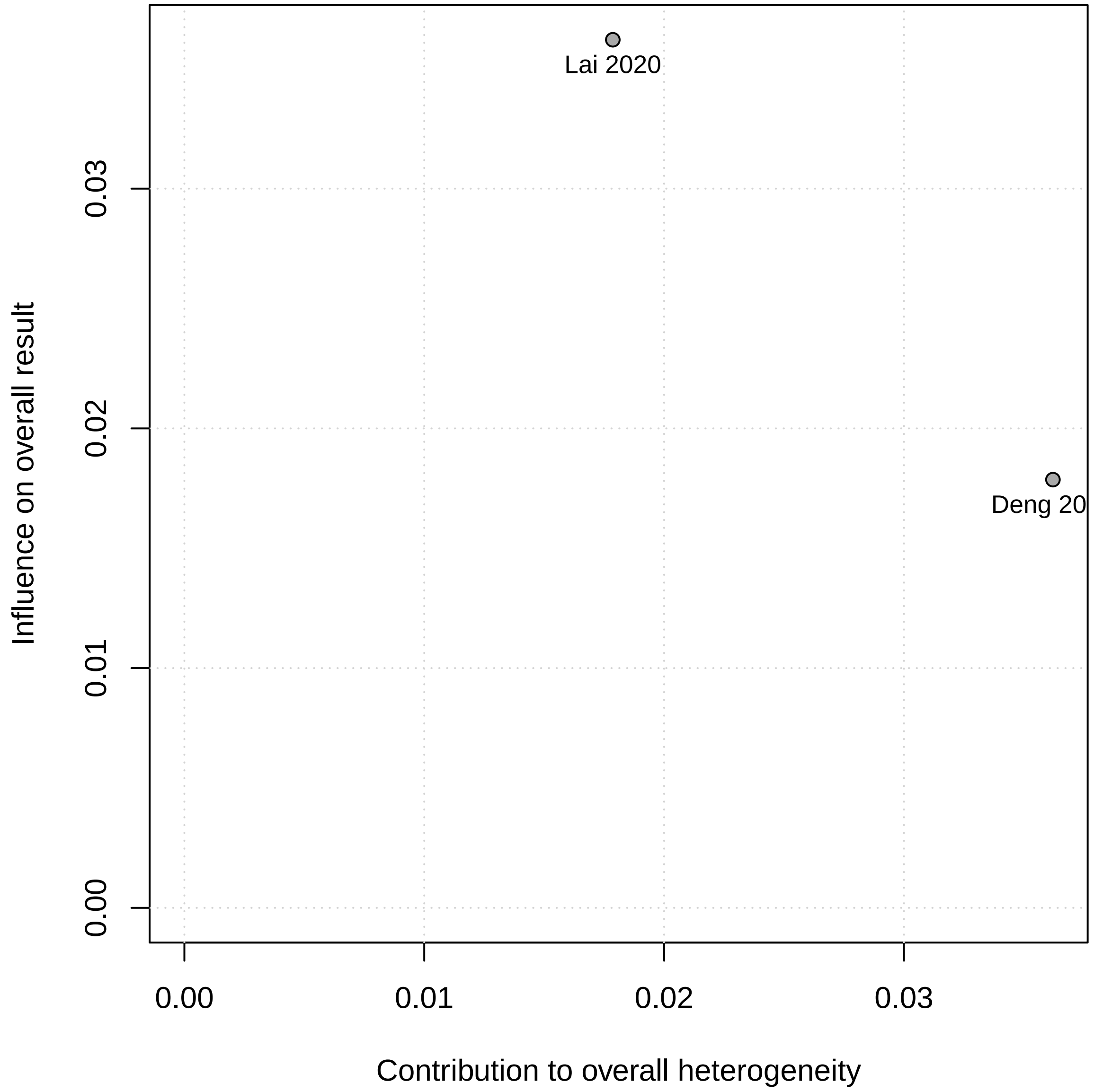

12.4.6 Leave one out

```
metainf(aux_rx_metabin)
```

```
## Influential analysis (common effect model)
##
##                          RR           95%-CI p-value   tau^2    tau   I^2
## Omitting Tang 2023   1.7887 [1.0028; 3.1907]  0.0489  0.0000 0.0000  0.0%
## Omitting Chen 2019   1.7887 [1.0028; 3.1907]  0.0489  0.0000 0.0000  0.0%
## Omitting Lai 2020    2.0468 [0.5108; 8.2010]  0.3118
## Omitting Deng 2022   1.7143 [0.9190; 3.1978]  0.0902
##
## Pooled estimate      1.7887 [1.0028; 3.1907]  0.0489  0.0000 0.0000  0.0%
##
## Details on meta-analytical method:
## - Mantel-Haenszel method
## - Restricted maximum-likelihood estimator for tau^2
```

12.5 VAS

12.5.1 Meta-analysis

```
vas_metacont <- metacont(data = minipcnl_only,
                             mean.c = vas_score_mean_control,
                             sd.c = vas_score_sd_control,
                             mean.e = vas_score_mean_suction,
                             sd.e = vas_score_sd_suction,
                             n.e = sample_size_suction,
                             n.c = sample_size_control,
                       studlab = author_year)
vas_metacont
```

```
## Number of studies: k = 1
## Number of observations: o = 473
##
##                             MD             95%-CI     z  p-value
## Common effect model  -2.1000 [-2.6611; -1.5389] -7.34 < 0.0001
## Random effects model -2.1000 [-2.6611; -1.5389] -7.34 < 0.0001
##
## Quantifying heterogeneity:
##  tau^2 = NA; tau = NA; I^2 = NA; H = NA
##
## Details on meta-analytical method:
## - Inverse variance method
## - Restricted maximum-likelihood estimator for tau^2
```

12.5.2 Forest plot

```r
forest(vas_metacont,
       sortvar = TE)
```

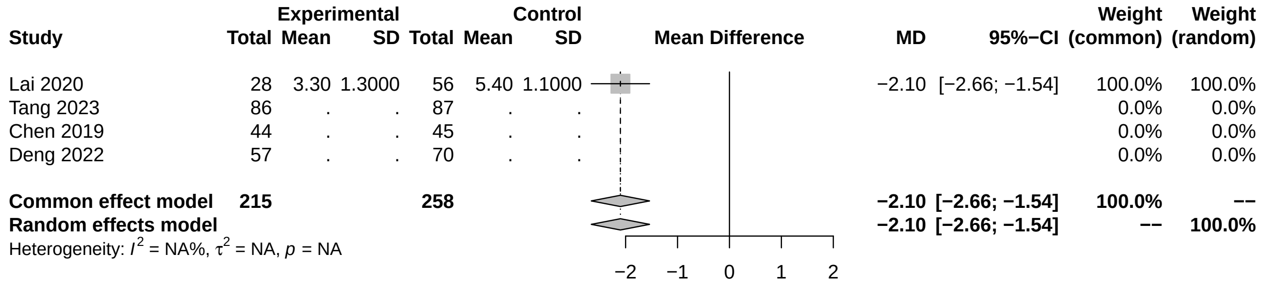

12.5.3 Trim and Fill

```r
#trimfill(vas_metacont)
#forest(trimfill(vas_metacont))
```

12.5.4 Funnel plot

```
funnel((vas_metacont))
```

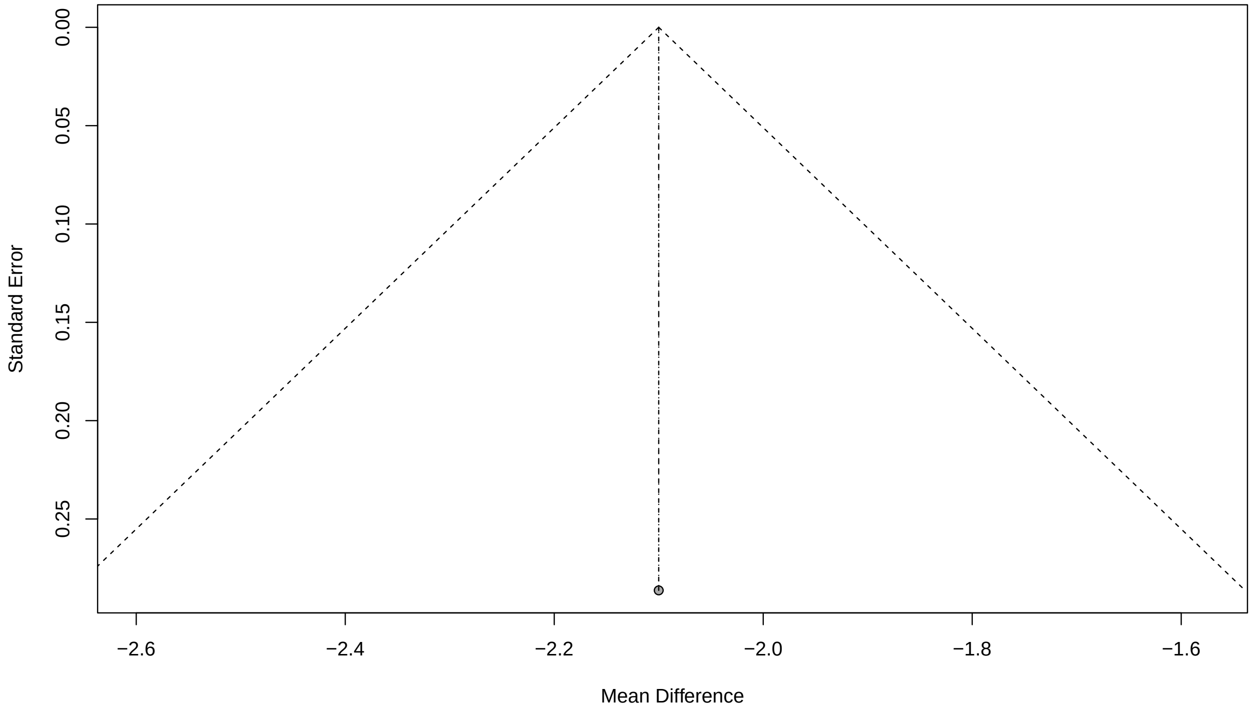

12.5.5 Baujat

```
baujat(vas_metacont, pos = 1)
```

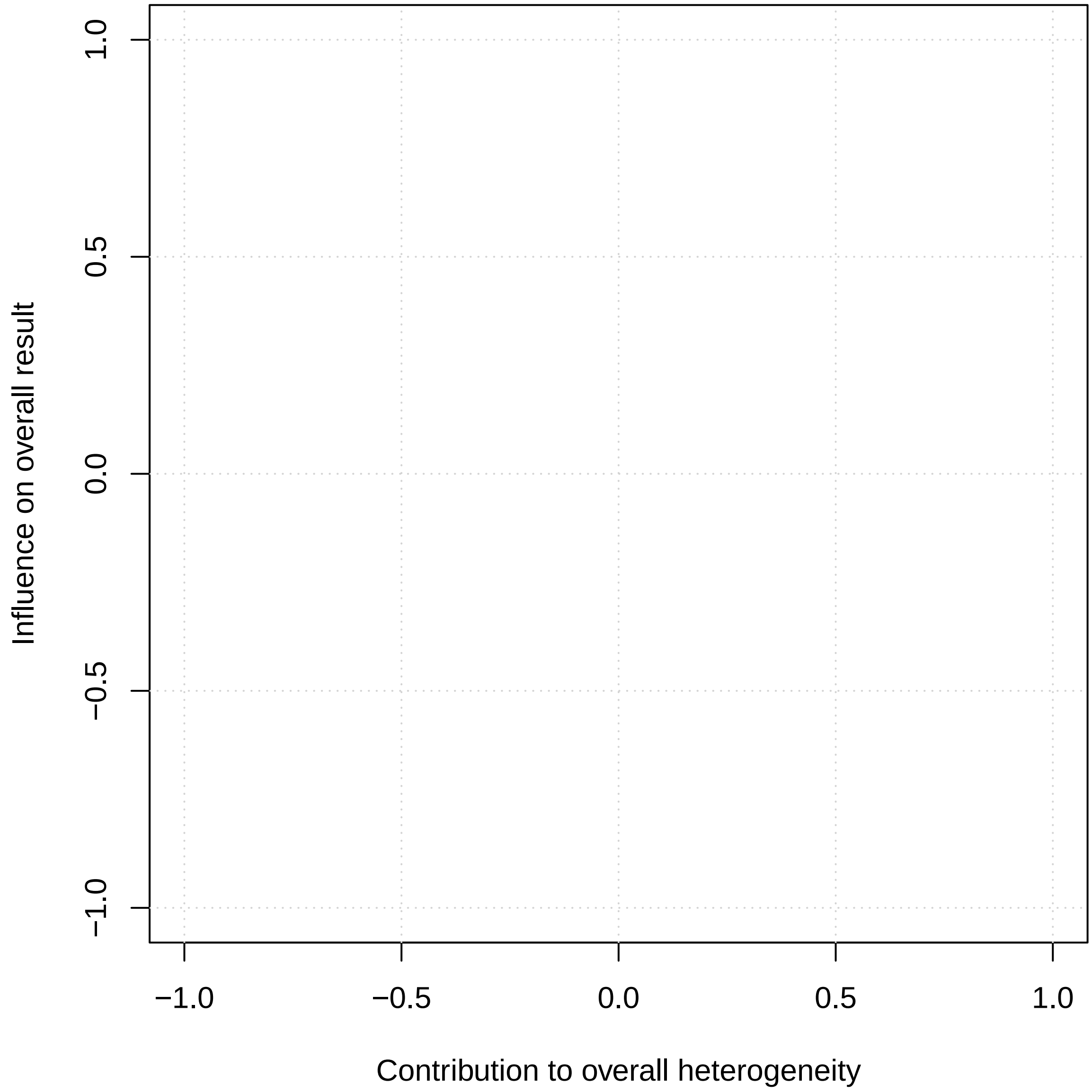

12.5.6 Leave one out

```r
metainf(vas_metacont)
```

```
## Influential analysis (common effect model)
##
##                            MD             95%-CI  p-value tau^2 tau I^2
## Omitting Tang 2023   -2.1000 [-2.6611; -1.5389] < 0.0001
## Omitting Chen 2019   -2.1000 [-2.6611; -1.5389] < 0.0001
## Omitting Lai 2020
## Omitting Deng 2022   -2.1000 [-2.6611; -1.5389] < 0.0001
##
## Pooled estimate      -2.1000 [-2.6611; -1.5389] < 0.0001
##
## Details on meta-analytical method:
## - Inverse variance method
## - Restricted maximum-likelihood estimator for tau^2
```

12.6 LoS

12.6.1 Meta-analysis

```
los_metacont <- metacont(data = minipcnl_only,
                             mean.c = los_days_mean_control,
                             sd.c = los_days_sd_control,
                             mean.e = los_days_mean_suction,
                             sd.e = los_days_sd_suction,
                             n.e = sample_size_suction,
                             n.c = sample_size_control,
                       studlab = author_year)
los_metacont
```

```
## Number of studies: k = 3
## Number of observations: o = 473
##
##                           MD             95%-CI      z  p-value
## Common effect model  -2.9428 [-3.4716; -2.4139] -10.91 < 0.0001
## Random effects model -2.8894 [-3.5491; -2.2296]  -8.58 < 0.0001
##
## Quantifying heterogeneity:
##  tau^2 = 0.1088 [0.0000; 24.5107]; tau = 0.3299 [0.0000; 4.9508]
##  I^2 = 41.9% [0.0%; 82.4%]; H = 1.31 [1.00; 2.38]
##
## Test of heterogeneity:
##     Q d.f. p-value
##  3.44    2  0.1787
##
## Details on meta-analytical method:
## - Inverse variance method
## - Restricted maximum-likelihood estimator for tau^2
## - Q-Profile method for confidence interval of tau^2 and tau
```

12.6.2 Forest plot

```r
forest(los_metacont,
       sortvar = TE)
```

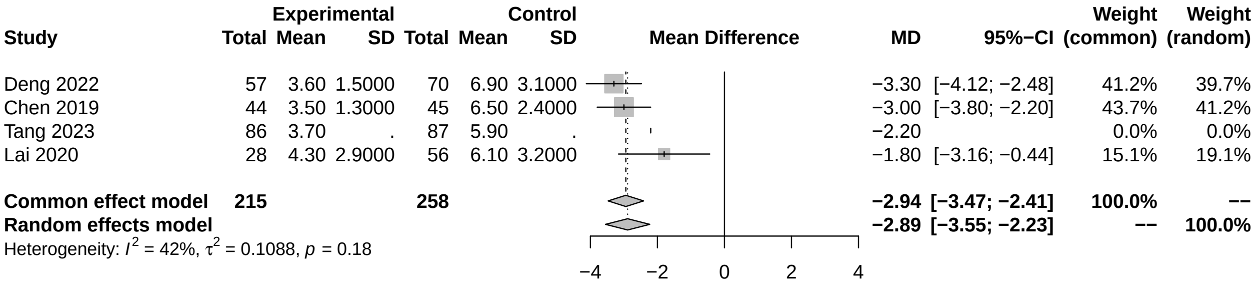

12.6.3 Trim and Fill

```r
trimfill(los_metacont)
```

```
## Warning in trimfill.meta(los_metacont): 1 observation(s) dropped due to missing
## values
```

```
## Number of studies: k = 3 (with 0 added studies)
## Number of observations: o = 300
##
##                           MD              95%-CI     z  p-value
## Random effects model -2.8894 [-3.5491; -2.2296] -8.58 < 0.0001
##
## Quantifying heterogeneity:
##  tau^2 = 0.1088 [0.0000; 24.5107]; tau = 0.3299 [0.0000; 4.9508]
##  I^2 = 41.9% [0.0%; 82.4%]; H = 1.31 [1.00; 2.38]
##
## Test of heterogeneity:
##     Q d.f. p-value
##  3.44    2  0.1787
##
## Details on meta-analytical method:
## - Inverse variance method
## - Restricted maximum-likelihood estimator for tau^2
## - Q-Profile method for confidence interval of tau^2 and tau
## - Trim-and-fill method to adjust for funnel plot asymmetry (L-estimator)
```

```r
forest(trimfill(los_metacont),
       sortvar = TE)
```

```
## Warning in trimfill.meta(los_metacont): 1 observation(s) dropped due to missing
## values
```

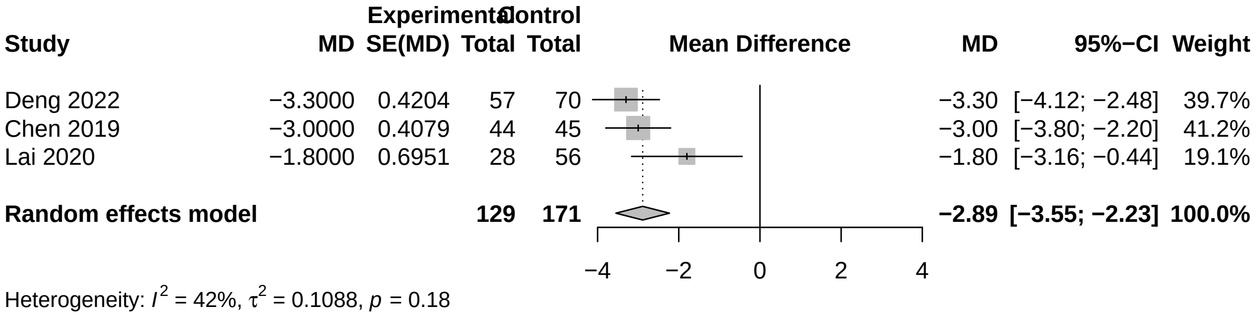

12.6.4 Funnel plot

```
funnel(trimfill(los_metacont))
```

```
## Warning in trimfill.meta(los_metacont): 1 observation(s) dropped due to missing
## values
```

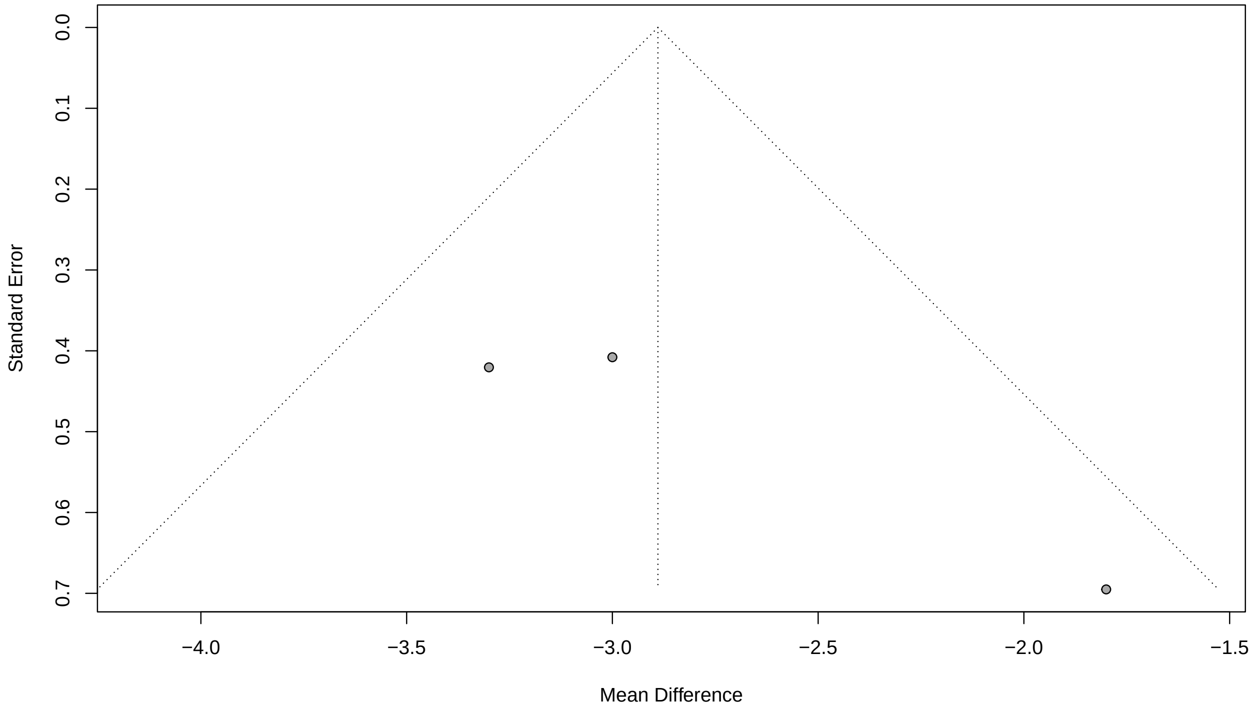

12.6.5 Baujat

```
baujat(los_metacont, pos = 1)
```

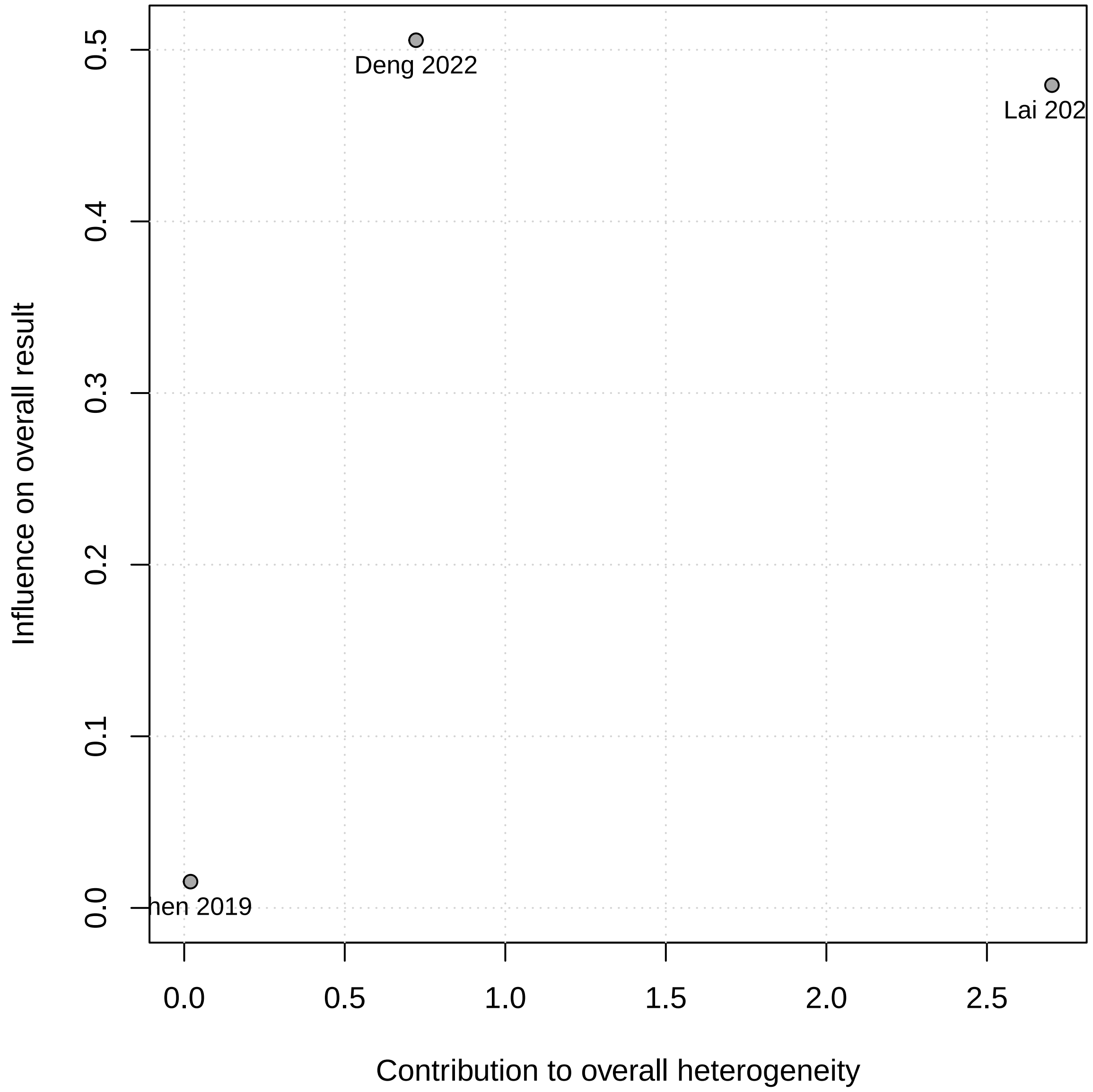

12.6.6 Leave one out

```
metainf(los_metacont)
```

```
## Influential analysis (common effect model)
##
##                           MD             95%-CI  p-value   tau^2     tau    I^2
## Omitting Tang 2023   -2.9428 [-3.4716; -2.4139] < 0.0001  0.1088  0.3299  41.9%
## Omitting Chen 2019   -2.8983 [-3.6034; -2.1932] < 0.0001  0.7950  0.8916  70.7%
## Omitting Lai 2020    -3.1455 [-3.7193; -2.5717] < 0.0001  0.0000  0.0000   0.0%
## Omitting Deng 2022   -2.6926 [-3.3822; -2.0030] < 0.0001  0.3952  0.6286  54.9%
##
## Pooled estimate      -2.9428 [-3.4716; -2.4139] < 0.0001  0.1088  0.3299  41.9%
##
## Details on meta-analytical method:
## - Inverse variance method
## - Restricted maximum-likelihood estimator for tau^2
```

12.7 Complications - All

12.7.1 Meta-analysis

```
comp_metabin <- metabin(data = minipcnl_only,
                          event.c = complications_postop_overall_n_control,
                        n.c = sample_size_control,
                        event.e = complications_postop_overall_n_suction,
                        n.e = sample_size_suction,
                        studlab = author_year)
comp_metabin
```

```
## Number of studies: k = 4
## Number of observations: o = 473
## Number of events: e = 97
##
##                           RR           95%-CI     z  p-value
## Common effect model  0.3823 [0.2508; 0.5825] -4.47 < 0.0001
## Random effects model 0.4151 [0.2121; 0.8121] -2.57   0.0102
##
## Quantifying heterogeneity:
##  tau^2 = 0.2715 [0.0000; 6.4062]; tau = 0.5211 [0.0000; 2.5310]
##  I^2 = 57.8% [0.0%; 86.0%]; H = 1.54 [1.00; 2.67]
##
## Test of heterogeneity:
##     Q d.f. p-value
##  7.10    3  0.0687
##
## Details on meta-analytical method:
## - Mantel-Haenszel method
## - Inverse variance method
## - Restricted maximum-likelihood estimator for tau^2
## - Q-Profile method for confidence interval of tau^2 and tau
```

12.7.2 Forest plot

```r
forest(comp_metabin,
       sortvar = TE)
```

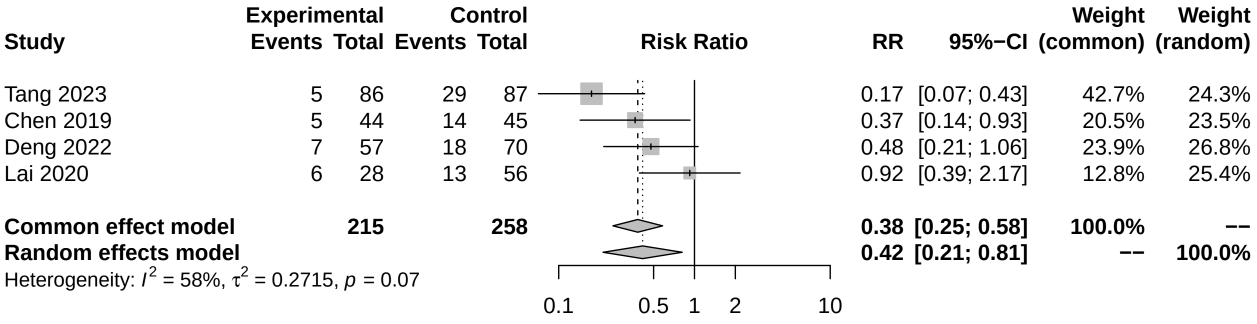

12.7.3 Trim and Fill

```
trimfill(comp_metabin)
```

```
## Number of studies: k = 5 (with 1 added studies)
## Number of observations: o = 646
## Number of events: e = 131
##
##                            RR           95%-CI     z p-value
## Random effects model 0.5489 [0.2557; 1.1786] -1.54  0.1239
##
## Quantifying heterogeneity:
##  tau^2 = 0.5593 [0.0697; 6.2184]; tau = 0.7479 [0.2639; 2.4937]
##  I^2 = 73.1% [32.6%; 89.2%]; H = 1.93 [1.22; 3.05]
##
## Test of heterogeneity:
##      Q d.f. p-value
##  14.85    4  0.0050
##
## Details on meta-analytical method:
## - Inverse variance method
## - Restricted maximum-likelihood estimator for tau^2
## - Q-Profile method for confidence interval of tau^2 and tau
## - Trim-and-fill method to adjust for funnel plot asymmetry (L-estimator)
```

```
forest(trimfill(comp_metabin),
       sortvar = TE)
```

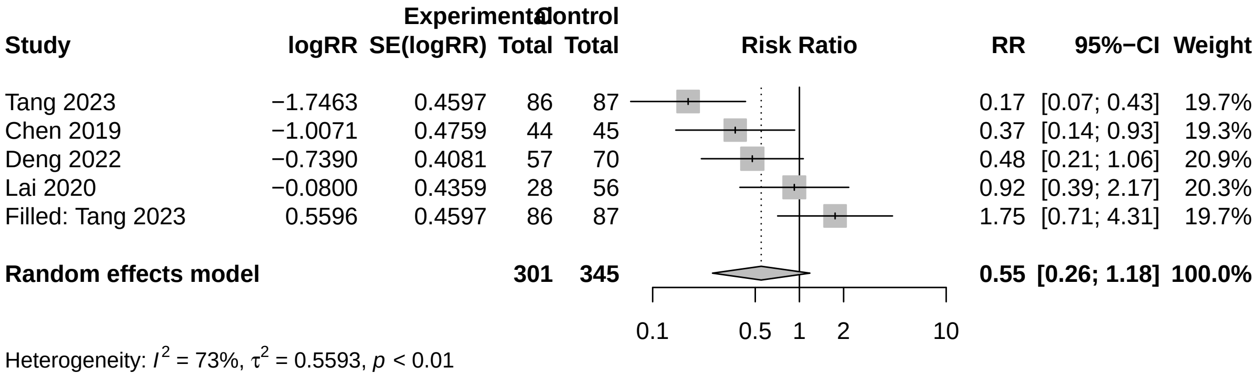

12.7.4 Funnel plot

```r
funnel(trimfill(comp_metabin))
```

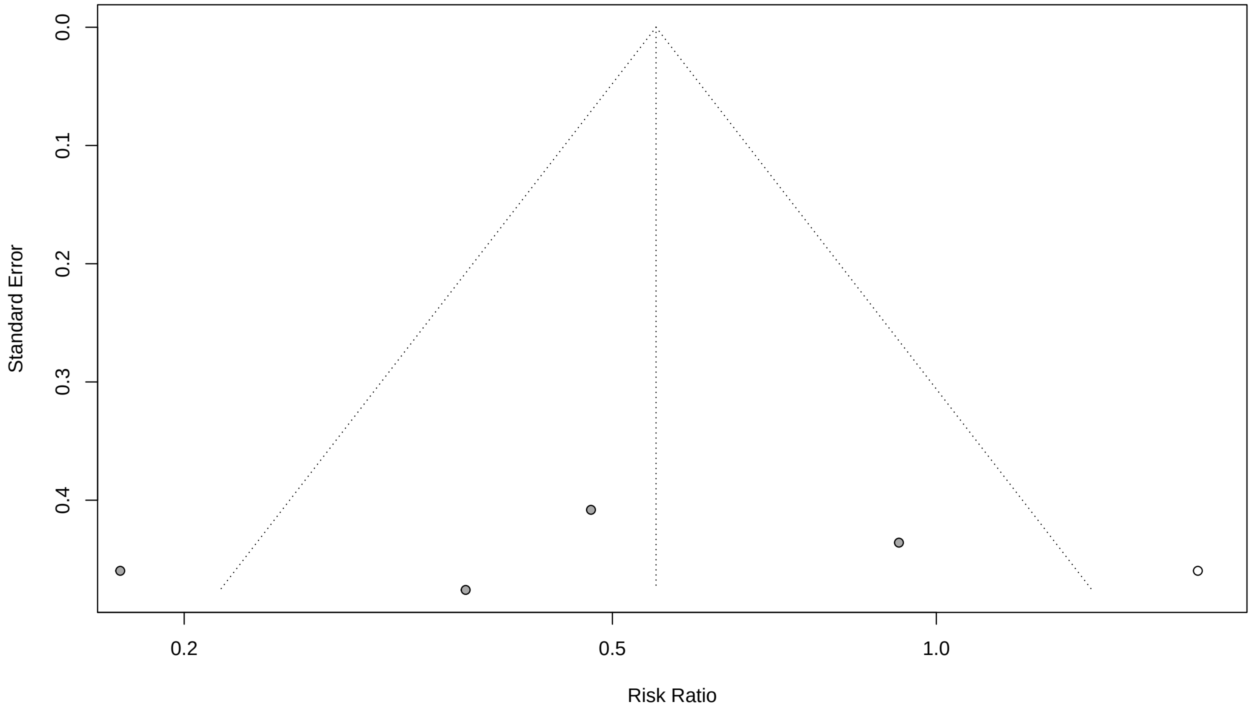

12.7.5 Baujat

```r
baujat(comp_metabin, pos = 1)
```

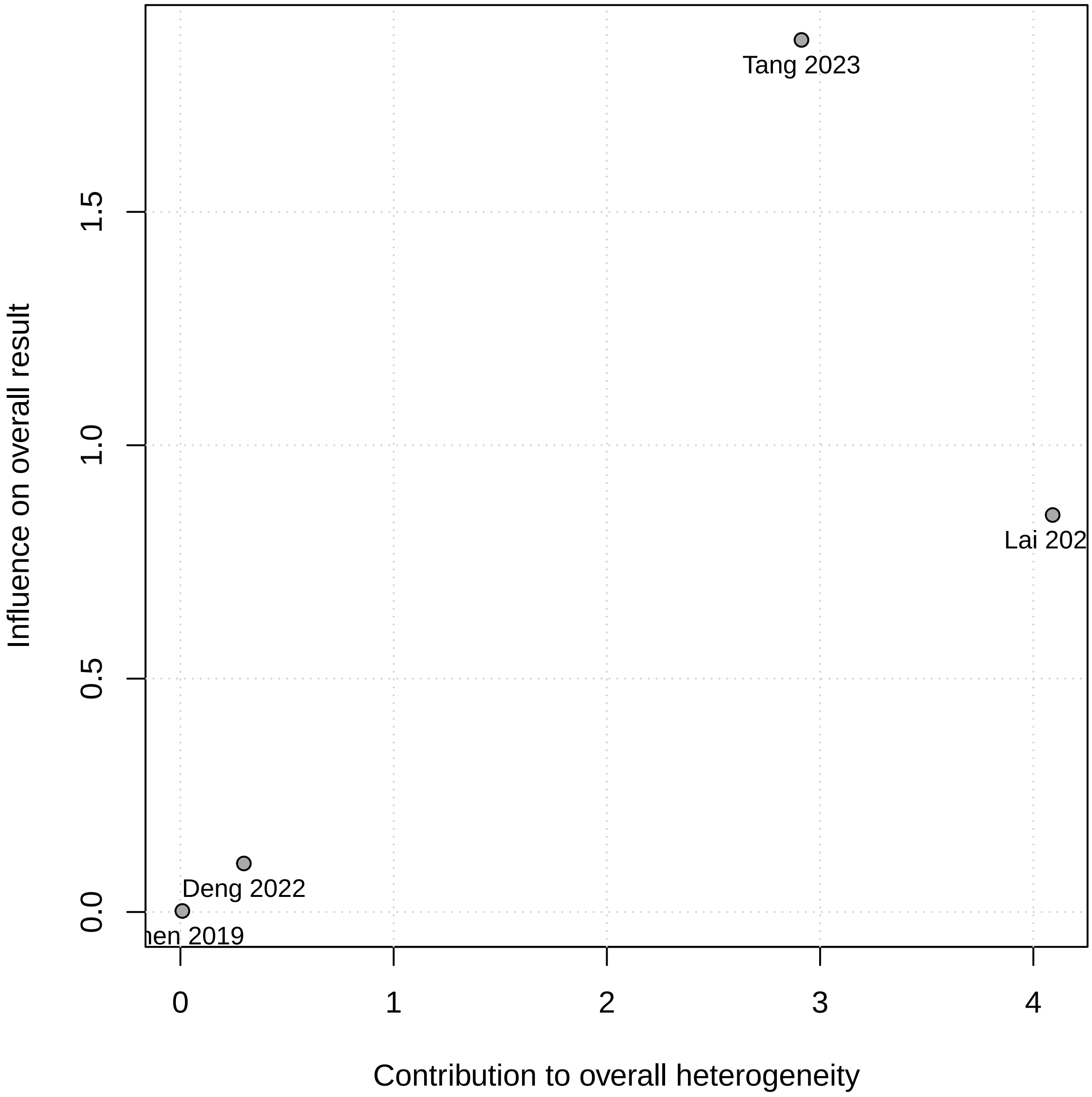

12.7.6 Leave one out

```r
metainf(comp_metabin)
```

```
## Influential analysis (common effect model)
##
##                           RR           95%-CI  p-value   tau^2     tau    I^2
## Omitting Tang 2023   0.5372 [0.3298; 0.8752]   0.0126  0.0203  0.1423  11.9%
## Omitting Chen 2019   0.3866 [0.2411; 0.6199] < 0.0001  0.4894  0.6996  71.3%
## Omitting Lai 2020    0.3026 [0.1841; 0.4972] < 0.0001  0.0812  0.2849  28.1%
## Omitting Deng 2022   0.3522 [0.2143; 0.5789] < 0.0001  0.5062  0.7115  71.3%
##
## Pooled estimate      0.3823 [0.2508; 0.5825] < 0.0001  0.2715  0.5211  57.8%
##
## Details on meta-analytical method:
## - Mantel-Haenszel method
## - Restricted maximum-likelihood estimator for tau^2
```

12.8 Fever

12.8.1 Meta-analysis

```
fever_metabin <- metabin(data = minipcnl_only,
                          event.c = fever_n_control,
                        n.c = sample_size_control,
                        event.e = fever_n_suction,
                        n.e = sample_size_suction,
                        studlab = author_year)
fever_metabin
```

```
## Number of studies: k = 4
## Number of observations: o = 473
## Number of events: e = 35
##
##                          RR           95%-CI     z p-value
## Common effect model  0.6409 [0.3280; 1.2522] -1.30  0.1930
## Random effects model 0.7017 [0.3485; 1.4129] -0.99  0.3212
##
## Quantifying heterogeneity:
##  tau^2 = 0 [0.0000; 6.7656]; tau = 0 [0.0000; 2.6011]
##  I^2 = 0.0% [0.0%; 84.7%]; H = 1.00 [1.00; 2.56]
##
## Test of heterogeneity:
##     Q d.f. p-value
##  2.88    3  0.4099
##
## Details on meta-analytical method:
## - Mantel-Haenszel method
## - Inverse variance method
## - Restricted maximum-likelihood estimator for tau^2
## - Q-Profile method for confidence interval of tau^2 and tau
```

12.8.2 Forest plot

```r
forest(fever_metabin,
       sortvar = TE)
```

Study	Experimental Events	Experimental Total	Control Events	Control Total	Risk Ratio	RR	95%−CI	Weight (common)	Weight (random)
Tang 2023	2	86	9	87		0.22	[0.05; 1.01]	43.4%	21.7%
Lai 2020	2	28	5	56		0.80	[0.17; 3.87]	16.2%	19.7%
Chen 2019	3	44	3	45		1.02	[0.22; 4.80]	14.4%	20.5%
Deng 2022	5	57	6	70		1.02	[0.33; 3.18]	26.1%	38.1%
**Common effect model**		**215**		**258**		**0.64**	**[0.33; 1.25]**	**100.0%**	**−−**
**Random effects model**						**0.70**	**[0.35; 1.41]**	**−−**	**100.0%**

Heterogeneity: $I^2 = 0\%$, $\tau^2 = 0$, $p = 0.41$

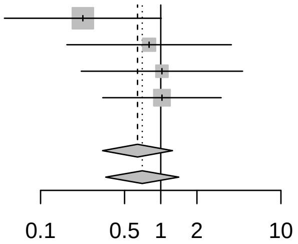

12.8.3 Trim and Fill

```r
trimfill(fever_metabin)
```

```
## Number of studies: k = 4 (with 0 added studies)
## Number of observations: o = 473
## Number of events: e = 35
##
##                          RR           95%-CI     z p-value
## Random effects model 0.7017 [0.3485; 1.4129] -0.99  0.3212
##
## Quantifying heterogeneity:
##  tau^2 = 0 [0.0000; 6.7656]; tau = 0 [0.0000; 2.6011]
##  I^2 = 0.0% [0.0%; 84.7%]; H = 1.00 [1.00; 2.56]
##
## Test of heterogeneity:
##     Q d.f. p-value
##  2.88    3  0.4099
##
## Details on meta-analytical method:
## - Inverse variance method
## - Restricted maximum-likelihood estimator for tau^2
## - Q-Profile method for confidence interval of tau^2 and tau
## - Trim-and-fill method to adjust for funnel plot asymmetry (L-estimator)
```

```r
forest(trimfill(fever_metabin),
       sortvar = TE)
```

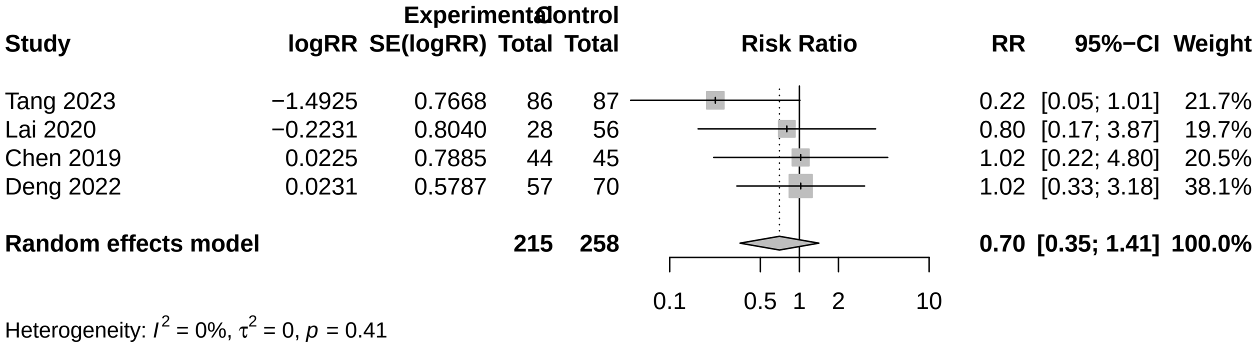

12.8.4 Funnel plot

```
funnel(trimfill(fever_metabin))
```

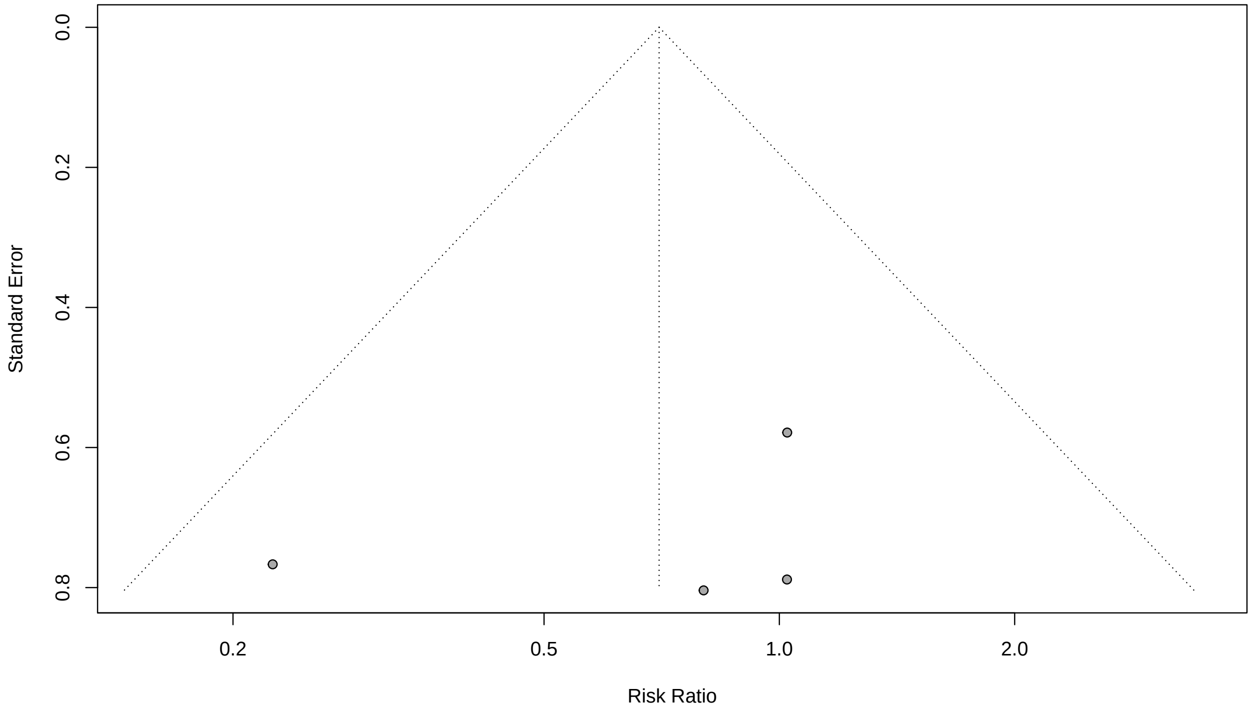

12.8.5 Baujat

```
baujat(fever_metabin, pos = 1)
```

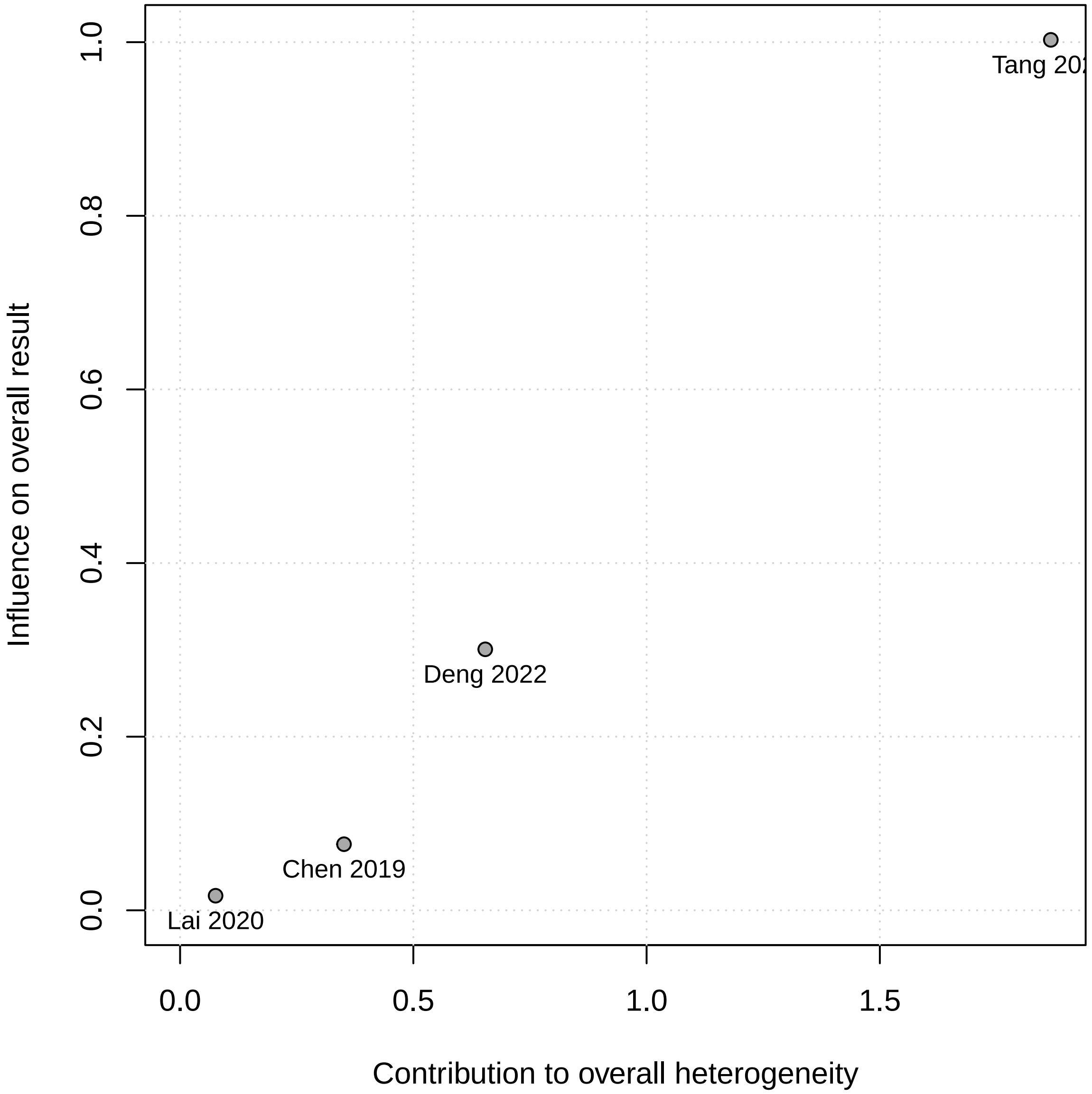

12.8.6 Leave one out

```
metainf(fever_metabin)
```

```
## Influential analysis (common effect model)
##
##                           RR           95%-CI p-value   tau^2    tau    I^2
## Omitting Tang 2023   0.9595 [0.4355; 2.1139]  0.9183  0.0000 0.0000   0.0%
## Omitting Chen 2019   0.5768 [0.2728; 1.2194]  0.1497  0.1571 0.3963  23.0%
## Omitting Lai 2020    0.6102 [0.2908; 1.2805]  0.1915  0.1959 0.4426  29.8%
## Omitting Deng 2022   0.5058 [0.2170; 1.1790]  0.1144  0.0752 0.2743   9.0%
##
## Pooled estimate      0.6409 [0.3280; 1.2522]  0.1930  0.0000 0.0000   0.0%
##
## Details on meta-analytical method:
## - Mantel-Haenszel method
## - Restricted maximum-likelihood estimator for tau^2
```

12.9 Infections

12.9.1 Meta-analysis

```
infection_metabin <- metabin(data = minipcnl_only,
                           event.c = infection_n_control,
                         n.c = sample_size_control,
                         event.e = infection_n_suction,
                         n.e = sample_size_suction,
                         studlab = author_year)
infection_metabin
```

```
## Number of studies: k = 4
## Number of observations: o = 473
## Number of events: e = 31
##
##                          RR           95%-CI     z p-value
## Common effect model  0.6381 [0.3152; 1.2918] -1.25  0.2119
## Random effects model 0.7003 [0.3191; 1.5367] -0.89  0.3743
##
## Quantifying heterogeneity:
##  tau^2 = 0.0588 [0.0000; 7.2737]; tau = 0.2426 [0.0000; 2.6970]
##  I^2 = 0.0% [0.0%; 84.7%]; H = 1.00 [1.00; 2.56]
##
## Test of heterogeneity:
##     Q d.f. p-value
##  2.94    3  0.4004
##
## Details on meta-analytical method:
## - Mantel-Haenszel method
## - Inverse variance method
## - Restricted maximum-likelihood estimator for tau^2
## - Q-Profile method for confidence interval of tau^2 and tau
```

12.9.2 Forest plot

```r
forest(infection_metabin,
       sortvar = TE)
```

Study	Experimental Events	Experimental Total	Control Events	Control Total	RR	95%−CI	Weight (common)	Weight (random)
Tang 2023	2	86	9	87	0.22	[0.05; 1.01]	48.0%	24.9%
Lai 2020	1	28	2	56	1.00	[0.09; 10.56]	7.2%	10.7%
Chen 2019	3	44	3	45	1.02	[0.22; 4.80]	15.9%	23.6%
Deng 2022	5	57	6	70	1.02	[0.33; 3.18]	28.9%	40.8%
**Common effect model**		**215**		**258**	**0.64**	**[0.32; 1.29]**	**100.0%**	**−−**
**Random effects model**					**0.70**	**[0.32; 1.54]**	**−−**	**100.0%**

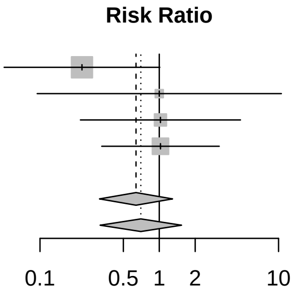

Heterogeneity: $I^2 = 0\%$, $\tau^2 = 0.0588$, $p = 0.40$

12.9.3 Trim and Fill

```r
trimfill(infection_metabin)
```

```
## Number of studies: k = 4 (with 0 added studies)
## Number of observations: o = 473
## Number of events: e = 31
##
##                          RR           95%-CI     z p-value
## Random effects model 0.7003 [0.3191; 1.5367] -0.89  0.3743
##
## Quantifying heterogeneity:
##  tau^2 = 0.0588 [0.0000; 7.2737]; tau = 0.2426 [0.0000; 2.6970]
##  I^2 = 0.0% [0.0%; 84.7%]; H = 1.00 [1.00; 2.56]
##
## Test of heterogeneity:
##     Q d.f. p-value
##  2.94    3  0.4004
##
## Details on meta-analytical method:
## - Inverse variance method
## - Restricted maximum-likelihood estimator for tau^2
## - Q-Profile method for confidence interval of tau^2 and tau
## - Trim-and-fill method to adjust for funnel plot asymmetry (L-estimator)
```

```r
forest(trimfill(infection_metabin),
       sortvar = TE)
```

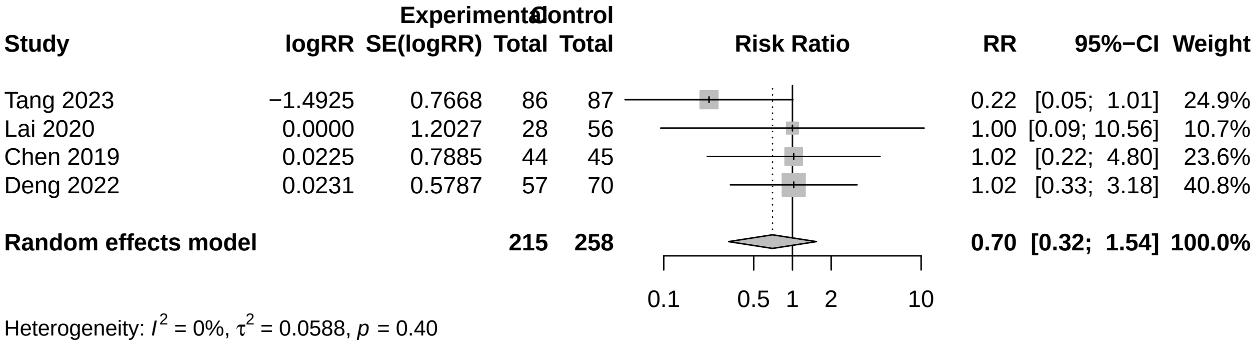

12.9.4 Funnel plot

```
funnel(trimfill(infection_metabin))
```

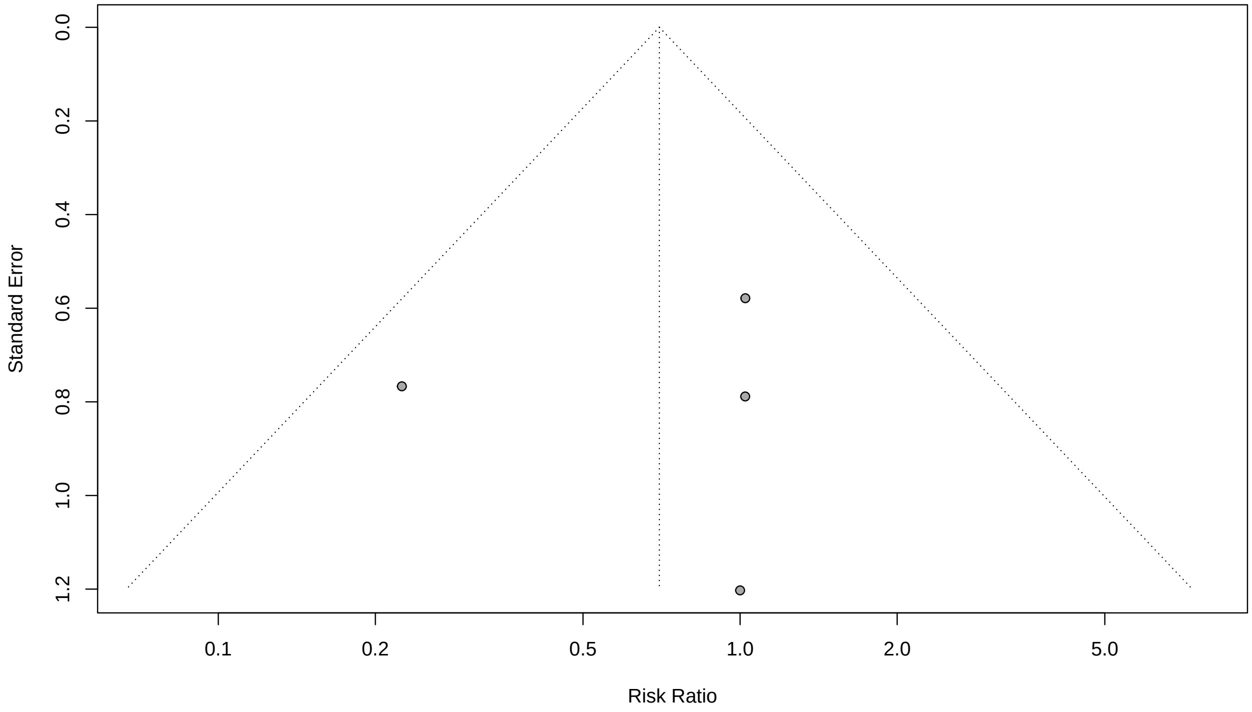

12.9.5 Baujat

```
baujat(infection_metabin, pos = 1)
```

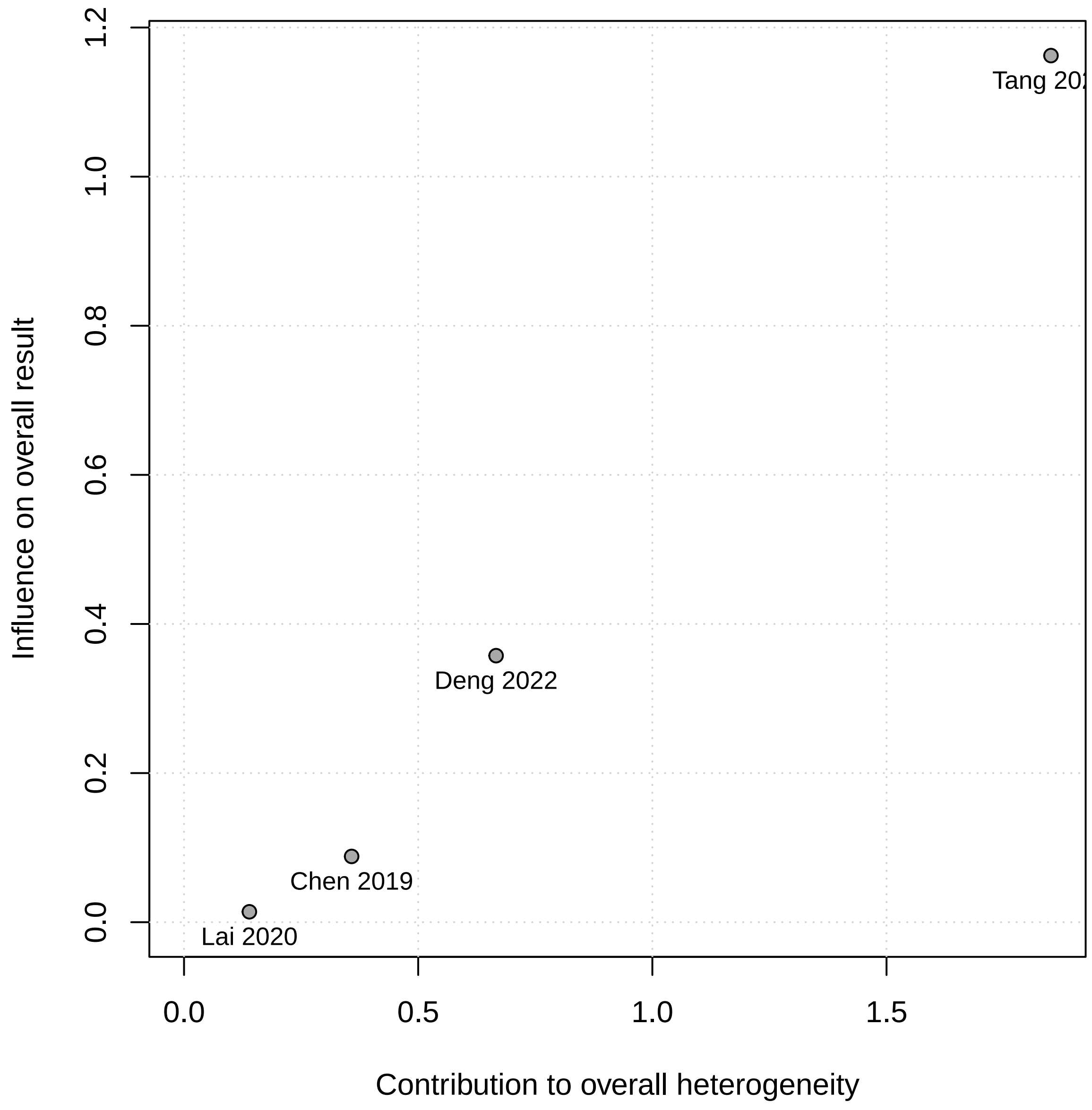

12.9.6 Leave one out

```
metainf(infection_metabin)
```

```
## Influential analysis (common effect model)
##
##                            RR           95%-CI p-value   tau^2    tau    I^2
## Omitting Tang 2023   1.0200 [0.4348; 2.3926]  0.9637  0.0000  0.0000   0.0%
## Omitting Chen 2019   0.5653 [0.2541; 1.2576]  0.1621  0.2938  0.5421  24.7%
## Omitting Lai 2020    0.6102 [0.2908; 1.2805]  0.1915  0.1959  0.4426  29.8%
## Omitting Deng 2022   0.4815 [0.1913; 1.2121]  0.1208  0.2048  0.4526  10.1%
##
## Pooled estimate      0.6381 [0.3152; 1.2918]  0.2119  0.0588  0.2426   0.0%
##
## Details on meta-analytical method:
## - Mantel-Haenszel method
## - Restricted maximum-likelihood estimator for tau^2
```

12.10 Sepsis

12.10.1 Meta-analysis

```
sepsis_metabin <- metabin(data = minipcnl_only,
                          event.c = sepsis_n_control,
                        n.c = sample_size_control,
                        event.e = sepsis_n_suction,
                        n.e = sample_size_suction,
                        studlab = author_year)
sepsis_metabin
```

```
## Number of studies: k = 1
## Number of observations: o = 473
## Number of events: e = 4
##
##                           RR           95%-CI     z p-value
## Common effect model  0.1124 [0.0061; 2.0562] -1.47  0.1405
## Random effects model 0.1124 [0.0061; 2.0562] -1.47  0.1405
##
## Quantifying heterogeneity:
##  tau^2 = NA; tau = NA; I^2 = NA; H = NA
##
## Details on meta-analytical method:
## - Mantel-Haenszel method
## - Inverse variance method
## - Restricted maximum-likelihood estimator for tau^2
## - Continuity correction of 0.5 in studies with zero cell frequencies
```

12.10.2 Forest plot

```r
forest(sepsis_metabin,
       sortvar = TE)
```

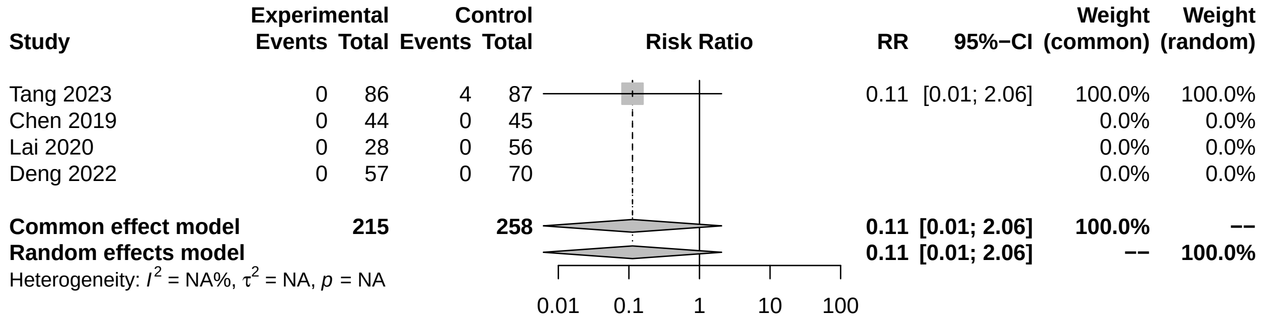

12.10.3 Trim and Fill

```
#trimfill(sepsis_metabin)
#forest(trimfill(sepsis_metabin),
#       sortvar = TE)
```

12.10.4 Funnel plot

```
funnel((sepsis_metabin))
```

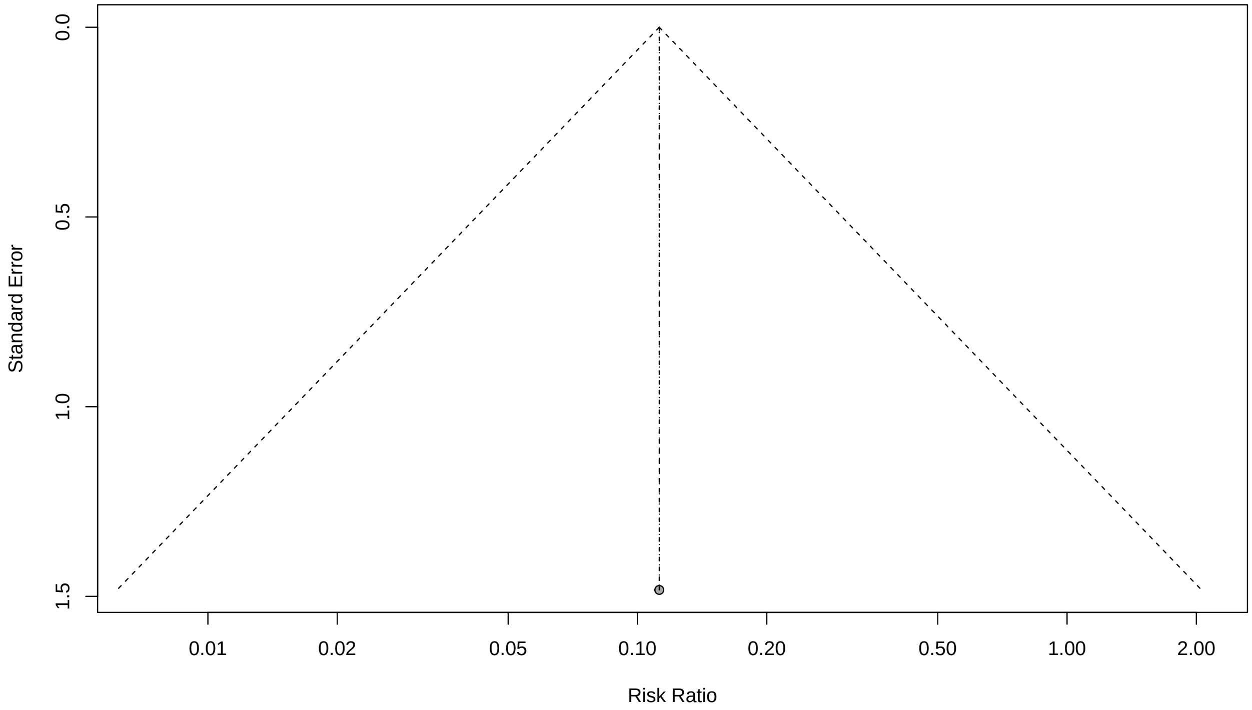

12.10.5 Baujat

```
baujat(sepsis_metabin, pos = 1)
```

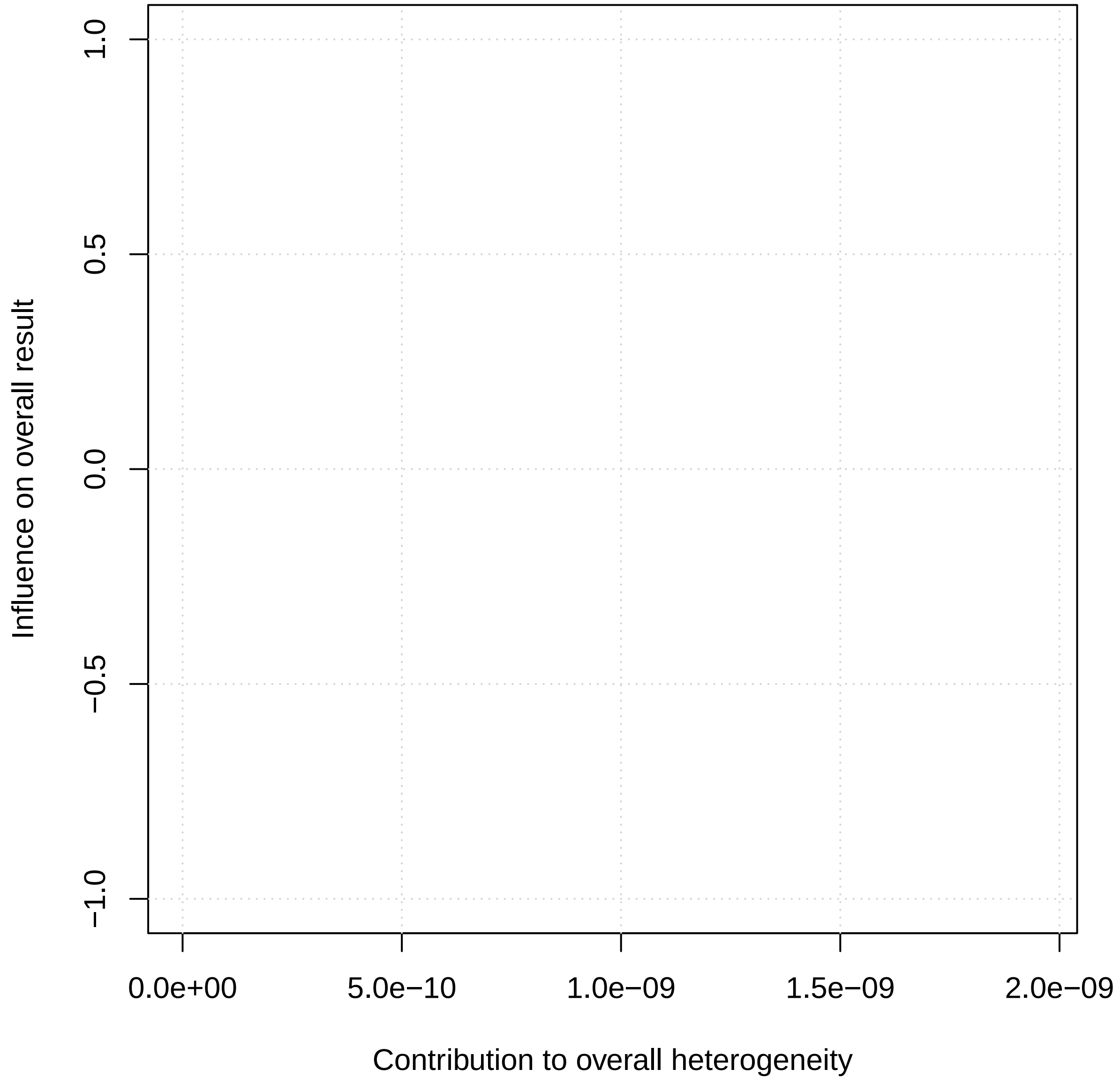

12.10.6 Leave one out

```r
metainf(sepsis_metabin)
```

```
## Influential analysis (common effect model)
##
##                          RR           95%-CI p-value tau^2 tau I^2
## Omitting Tang 2023
## Omitting Chen 2019   0.1124 [0.0061; 2.0562]  0.1405
## Omitting Lai 2020    0.1124 [0.0061; 2.0562]  0.1405
## Omitting Deng 2022   0.1124 [0.0061; 2.0562]  0.1405
##
## Pooled estimate      0.1124 [0.0061; 2.0562]  0.1405
##
## Details on meta-analytical method:
## - Mantel-Haenszel method
## - Restricted maximum-likelihood estimator for tau^2
```

12.11 Abscess

12.11.1 Meta-analysis

```
abscess_metabin <- metabin(data = minipcnl_only,
                         event.c = abscess_n_control,
                       n.c = sample_size_control,
                       event.e = abscess_n_suction,
                       n.e = sample_size_suction,
                       studlab = author_year)
abscess_metabin
```

```
## Number of studies: k = 0
## Number of observations: o = 473
## Number of events: e = 0
##
##                        RR 95%-CI  z p-value
## Common effect model  NA        --      --
## Random effects model NA        --      --
##
## Quantifying heterogeneity:
##  tau^2 = NA; tau = NA; I^2 = NA; H = NA
##
## Details on meta-analytical method:
## - Mantel-Haenszel method
## - Inverse variance method
## - Restricted maximum-likelihood estimator for tau^2
```

12.11.2 Forest plot

```r
forest(abscess_metabin,
       sortvar = TE)
```

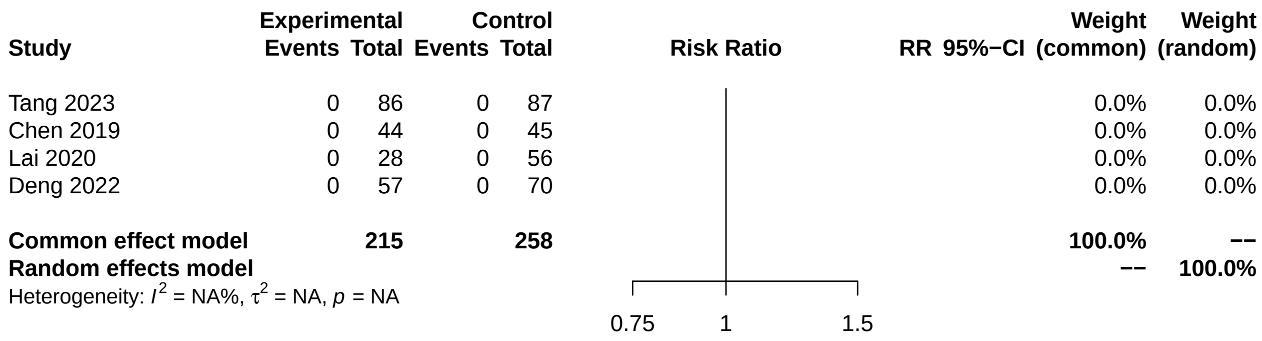

12.11.3 Trim and Fill

```
#trimfill(abscess_metabin)
#forest(trimfill(abscess_metabin),
#       sortvar = TE)
```

12.11.4 Funnel plot

```
#funnel((abscess_metabin))
```

12.11.5 Baujat

```
#baujat(abscess_metabin, pos = 1)
```

12.11.6 Leave one out

```
#metainf(abscess_metabin)
```

12.12 Haematoma

12.12.1 Meta-analysis

```
haematoma_metabin <- metabin(data = minipcnl_only,
                         event.c = hematoma_n_control,
                       n.c = sample_size_control,
                       event.e = hematoma_n_suction,
                       n.e = sample_size_suction,
                       studlab = author_year)
haematoma_metabin
```

```
## Number of studies: k = 1
## Number of observations: o = 473
## Number of events: e = 1
##
##                           RR           95%-CI     z p-value
## Common effect model  0.4080 [0.0169; 9.8294] -0.55  0.5808
## Random effects model 0.4087 [0.0170; 9.8436] -0.55  0.5815
##
## Quantifying heterogeneity:
##  tau^2 = NA; tau = NA; I^2 = NA; H = NA
##
## Details on meta-analytical method:
## - Mantel-Haenszel method
## - Inverse variance method
## - Restricted maximum-likelihood estimator for tau^2
## - Continuity correction of 0.5 in studies with zero cell frequencies
```

12.12.2 Forest plot

```r
forest(haematoma_metabin,
       sortvar = TE)
```

Study	Experimental Events	Experimental Total	Control Events	Control Total	RR	95%−CI	Weight (common)	Weight (random)
Deng 2022	0	57	1	70	0.41	[0.02; 9.84]	100.0%	100.0%
Tang 2023	0	86	0	87			0.0%	0.0%
Chen 2019	0	44	0	45			0.0%	0.0%
Lai 2020	0	28	0	56			0.0%	0.0%
**Common effect model**		**215**		**258**	**0.41**	**[0.02; 9.83]**	**100.0%**	**−−**
**Random effects model**					**0.41**	**[0.02; 9.84]**	**−−**	**100.0%**

Heterogeneity: I^2 = NA%, τ^2 = NA, p = NA

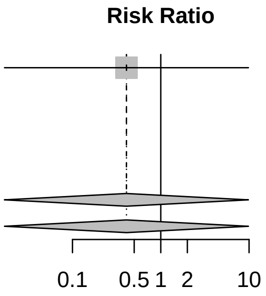

12.12.3 Trim and Fill

```
#trimfill(haematoma_metabin)
#forest(trimfill(haematoma_metabin),
#       sortvar = TE)
```

12.12.4 Funnel plot

```r
funnel((haematoma_metabin))
```

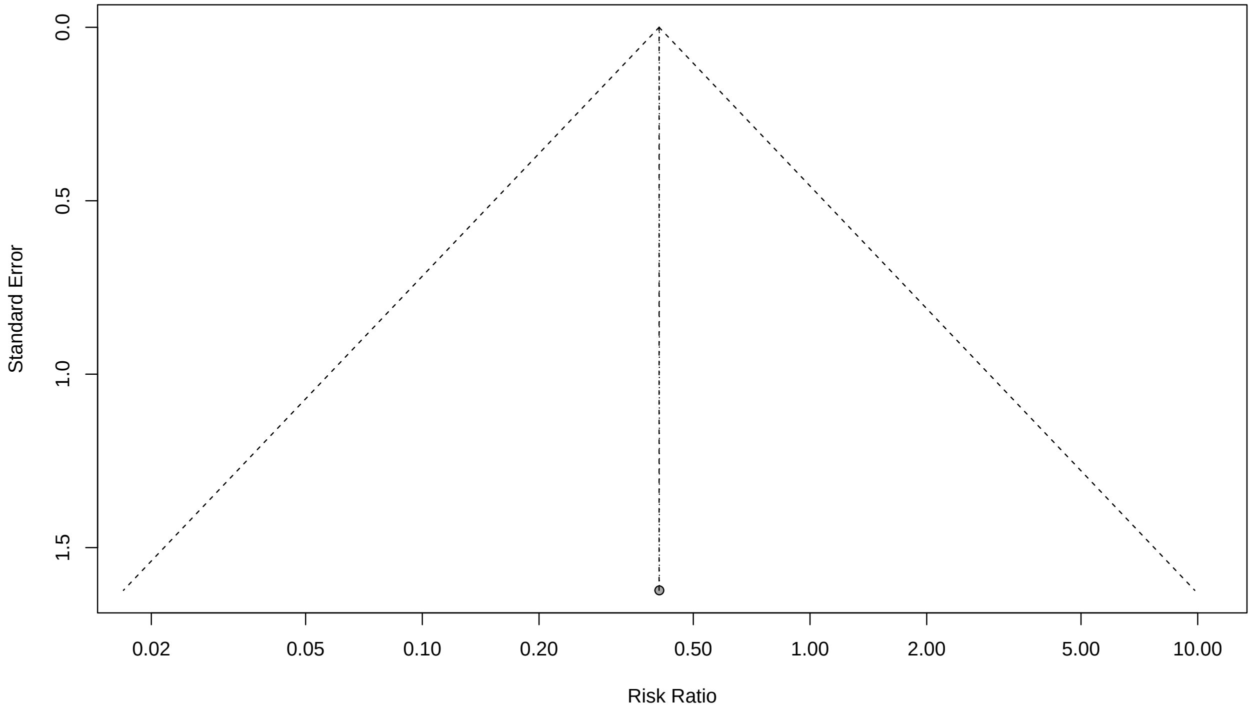

12.12.5 Baujat

```
baujat(haematoma_metabin, pos = 1)
```

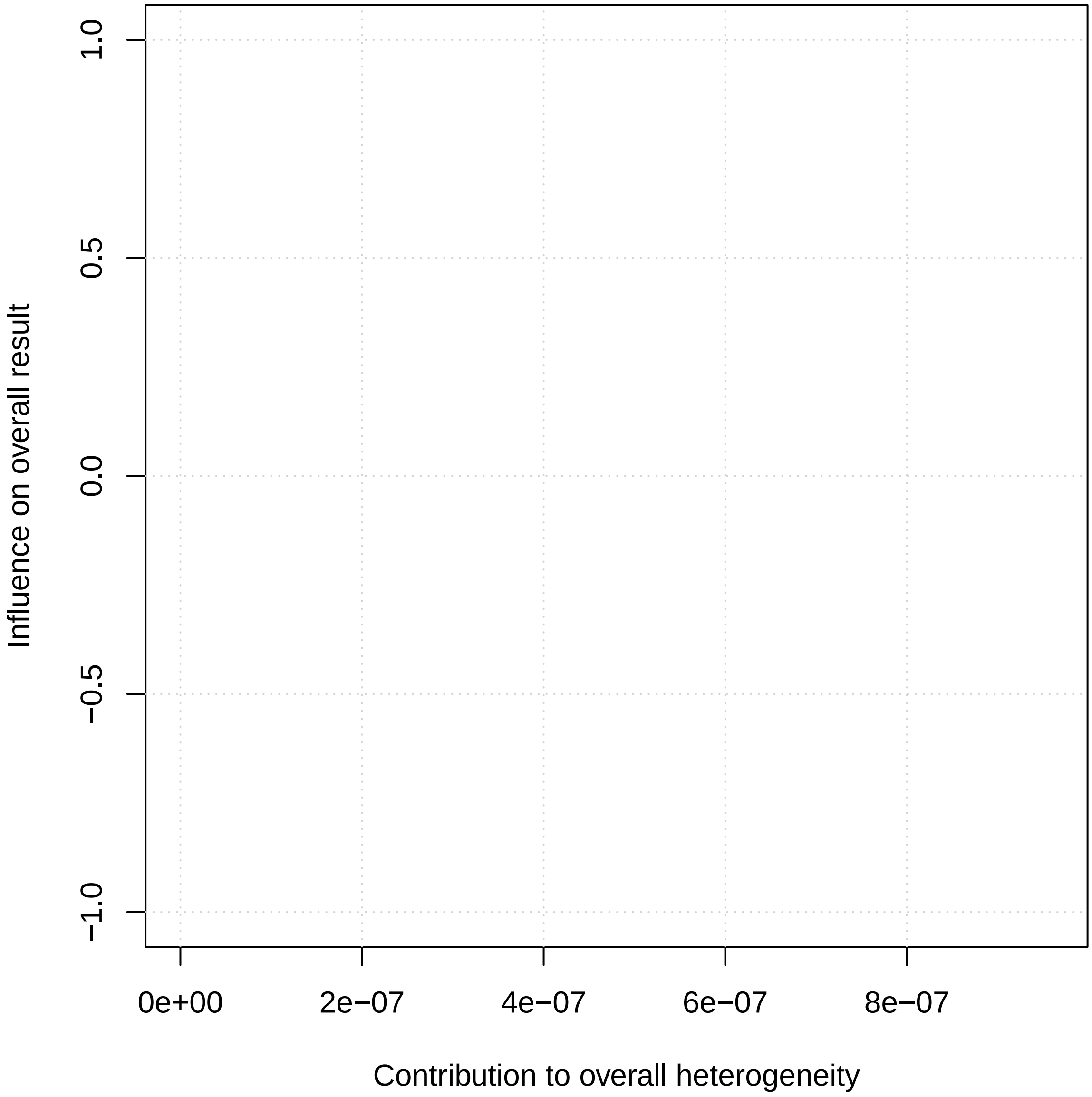

12.12.6 Leave one out

```r
metainf(haematoma_metabin)
```

```
## Influential analysis (common effect model)
##
##                            RR           95%-CI p-value tau^2 tau I^2
## Omitting Tang 2023   0.4080 [0.0169; 9.8294]  0.5808
## Omitting Chen 2019   0.4080 [0.0169; 9.8294]  0.5808
## Omitting Lai 2020    0.4080 [0.0169; 9.8294]  0.5808
## Omitting Deng 2022
##
## Pooled estimate      0.4080 [0.0169; 9.8294]  0.5808
##
## Details on meta-analytical method:
## - Mantel-Haenszel method
## - Restricted maximum-likelihood estimator for tau^2
```

12.13 Pain

12.13.1 Meta-analysis

```
pain_metabin <- metabin(data = minipcnl_only,
                          event.c = pain_n_control,
                        n.c = sample_size_control,
                        event.e = pain_n_suction,
                        n.e = sample_size_suction,
                        studlab = author_year)
pain_metabin
```

```
## Number of studies: k = 2
## Number of observations: o = 473
## Number of events: e = 24
##
##                             RR           95%-CI     z p-value
## Common effect model  0.2033 [0.0715; 0.5781] -2.99  0.0028
## Random effects model 0.2107 [0.0737; 0.6027] -2.90  0.0037
##
## Quantifying heterogeneity:
##  tau^2 = 0; tau = 0; I^2 = 0.0%; H = 1.00
##
## Test of heterogeneity:
##     Q d.f. p-value
##  0.34    1  0.5572
##
## Details on meta-analytical method:
## - Mantel-Haenszel method
## - Inverse variance method
## - Restricted maximum-likelihood estimator for tau^2
```

12.13.2 Forest plot

```r
forest(pain_metabin,
       sortvar = TE)
```

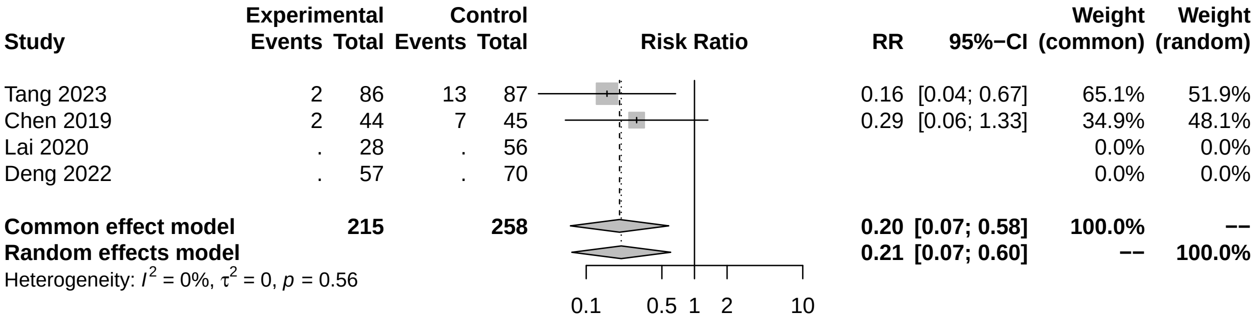

12.13.3 Trim and Fill

```
#trimfill(pain_metabin)
#forest(trimfill(pain_metabin),
#       sortvar = TE)
```

12.13.4 Funnel plot

```
funnel((pain_metabin))
```

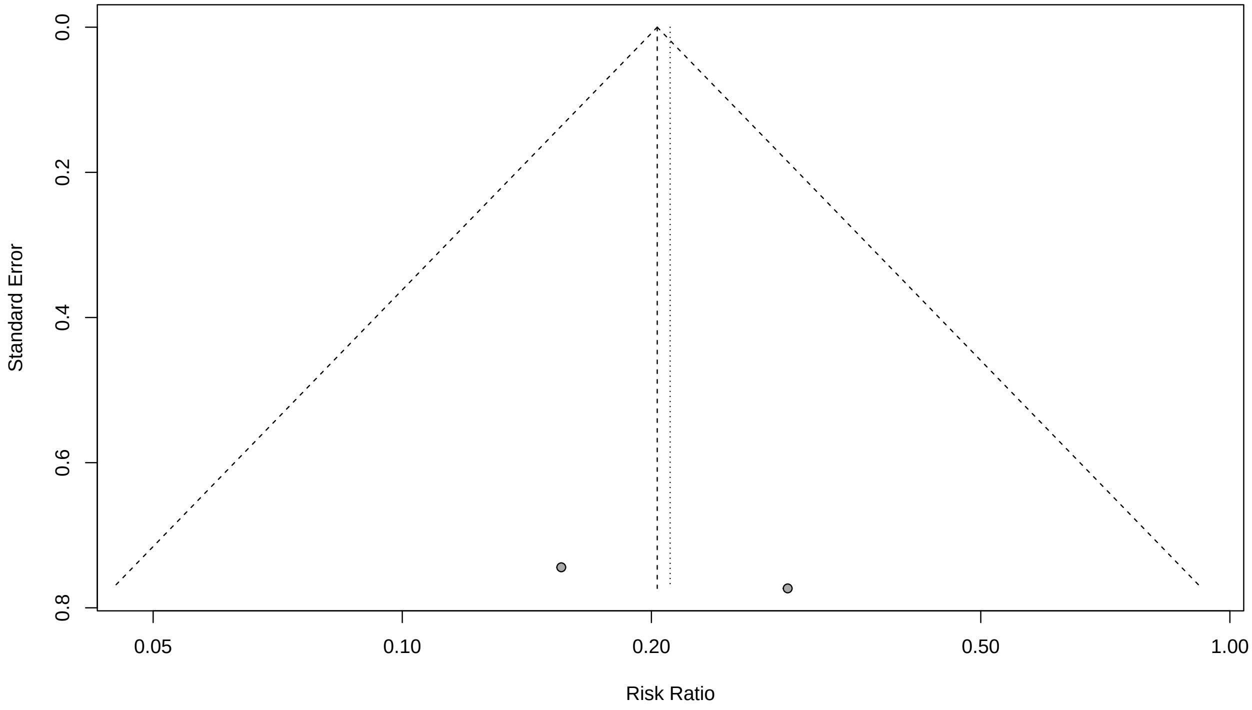

12.13.5 Baujat

```r
baujat(pain_metabin, pos = 1)
```

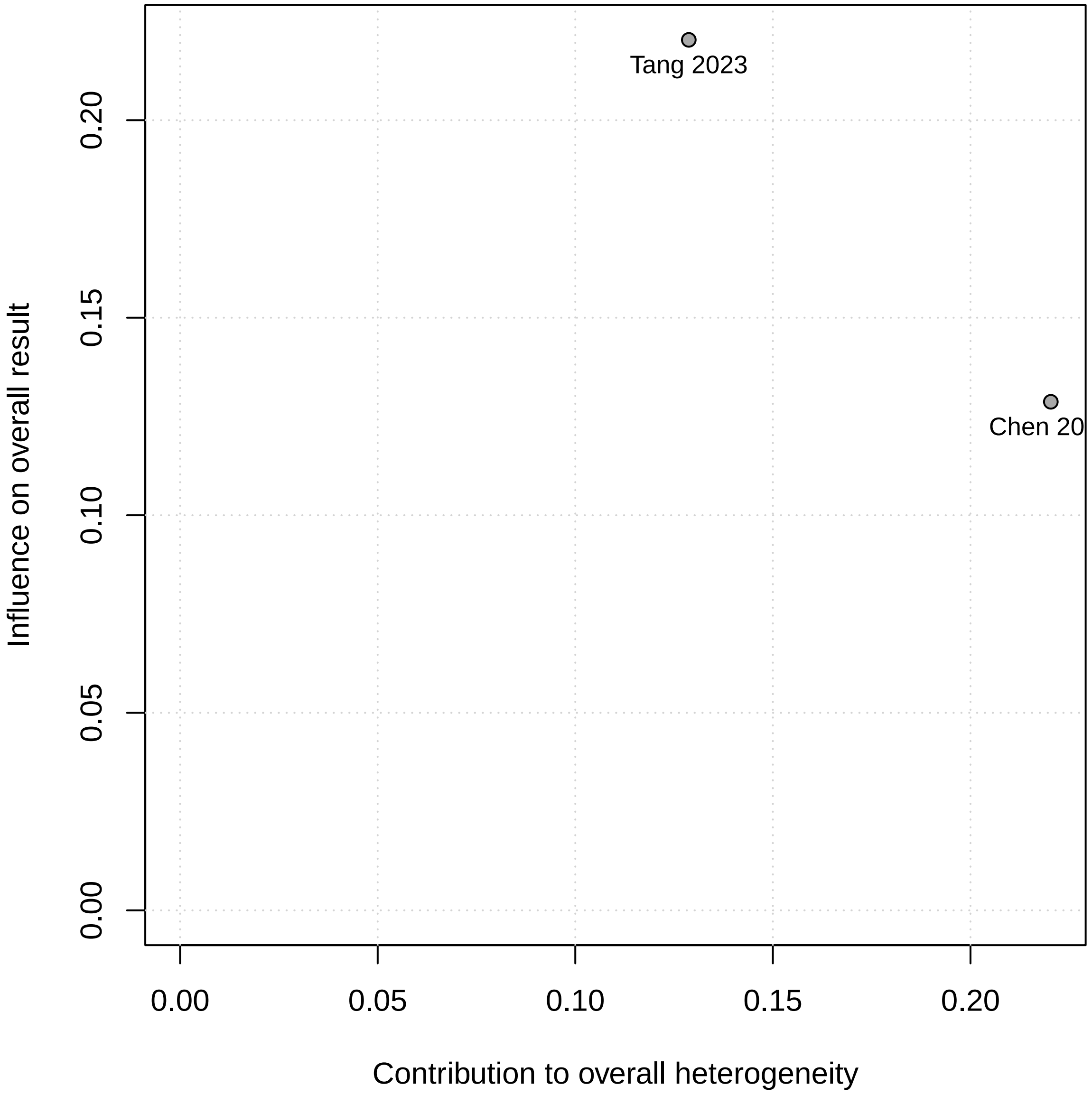

12.13.6 Leave one out

```
metainf(pain_metabin, pos = 1)
```

```
## Influential analysis (common effect model)
##
##                            RR           95%-CI p-value   tau^2     tau   I^2
## Omitting Tang 2023   0.2922 [0.0642; 1.3301]  0.1116
## Omitting Chen 2019   0.1556 [0.0362; 0.6692]  0.0124
## Omitting Lai 2020    0.2033 [0.0715; 0.5781]  0.0028  0.0000  0.0000  0.0%
## Omitting Deng 2022   0.2033 [0.0715; 0.5781]  0.0028  0.0000  0.0000  0.0%
##
## Pooled estimate      0.2033 [0.0715; 0.5781]  0.0028  0.0000  0.0000  0.0%
##
## Details on meta-analytical method:
## - Mantel-Haenszel method
## - Restricted maximum-likelihood estimator for tau^2
```

12.14 Stricture

12.14.1 Meta-analysis

```
stricture_metabin <- metabin(data = minipcnl_only,
                          event.c = stricture_n_control,
                        n.c = sample_size_control,
                        event.e = stricture_n_suction,
                        n.e = sample_size_suction,
                        studlab = author_year)
stricture_metabin
```

```
## Number of studies: k = 0
## Number of observations: o = 473
## Number of events: e = 0
##
##                        RR 95%-CI  z p-value
## Common effect model  NA        --      --
## Random effects model NA        --      --
##
## Quantifying heterogeneity:
##  tau^2 = NA; tau = NA; I^2 = NA; H = NA
##
## Details on meta-analytical method:
## - Mantel-Haenszel method
## - Inverse variance method
## - Restricted maximum-likelihood estimator for tau^2
```

12.14.2 Forest plot

```r
forest(stricture_metabin,
       sortvar = TE)
```

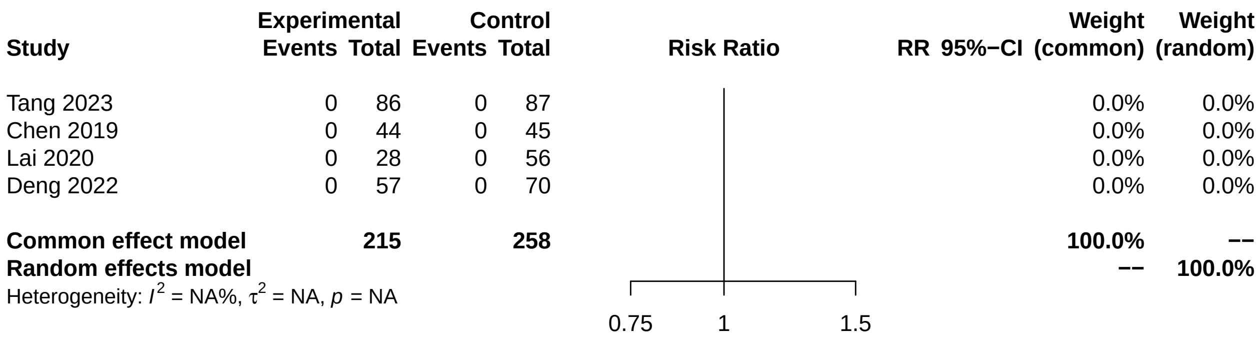

12.14.3 Trim and Fill

```
#trimfill(stricture_metabin)
#forest(trimfill(stricture_metabin),
#       sortvar = TE)
```

12.14.4 Funnel plot

```
#funnel((stricture_metabin))
```

12.14.5 Baujat

```
#baujat(stricture_metabin, pos = 1)
```

12.14.6 Leave one out

```r
#metainf(stricture_metabin)
```

12.15 Embolisation required

12.15.1 Meta-analysis

```
ir_embolisation_metabin <- metabin(data = minipcnl_only,
                         event.c = embolism_ir_intervention_n_control,
                       n.c = sample_size_control,
                       event.e = embolism_ir_intervention_n_suction,
                       n.e = sample_size_suction,
                       studlab = author_year)
ir_embolisation_metabin
```

```
## Number of studies: k = 2
## Number of observations: o = 473
## Number of events: e = 3
##
##                          RR           95%-CI     z p-value
## Common effect model  0.2830 [0.0318; 2.5161] -1.13  0.2575
## Random effects model 0.2867 [0.0322; 2.5524] -1.12  0.2627
##
## Quantifying heterogeneity:
##  tau^2 = 0; tau = 0; I^2 = 0.0%; H = 1.00
##
## Test of heterogeneity:
##     Q d.f. p-value
##  0.02    1  0.8828
##
## Details on meta-analytical method:
## - Mantel-Haenszel method
## - Inverse variance method
## - Restricted maximum-likelihood estimator for tau^2
## - Continuity correction of 0.5 in studies with zero cell frequencies
```

12.15.2 Forest plot

```r
forest(ir_embolisation_metabin,
       sortvar = TE)
```

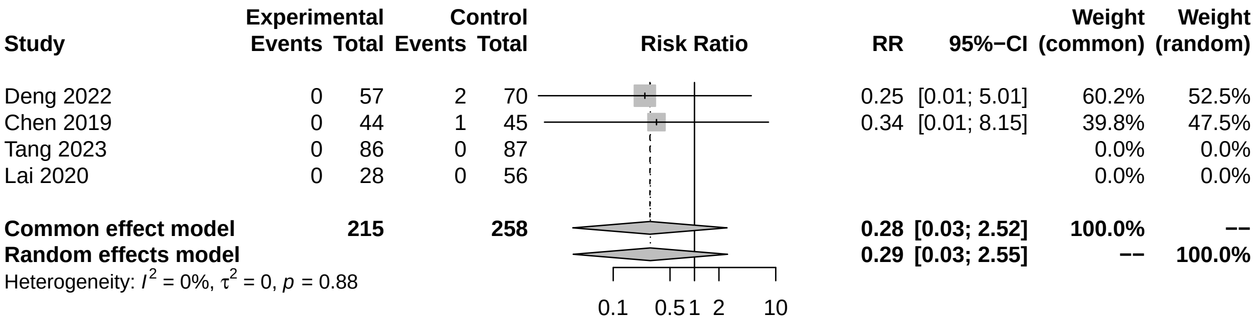

12.15.3 Trim and Fill

```
#trimfill(ir_embolisation_metabin)
#forest(trimfill(ir_embolisation_metabin),
#       sortvar = TE)
```

12.15.4 Funnel plot

```
funnel((ir_embolisation_metabin))
```

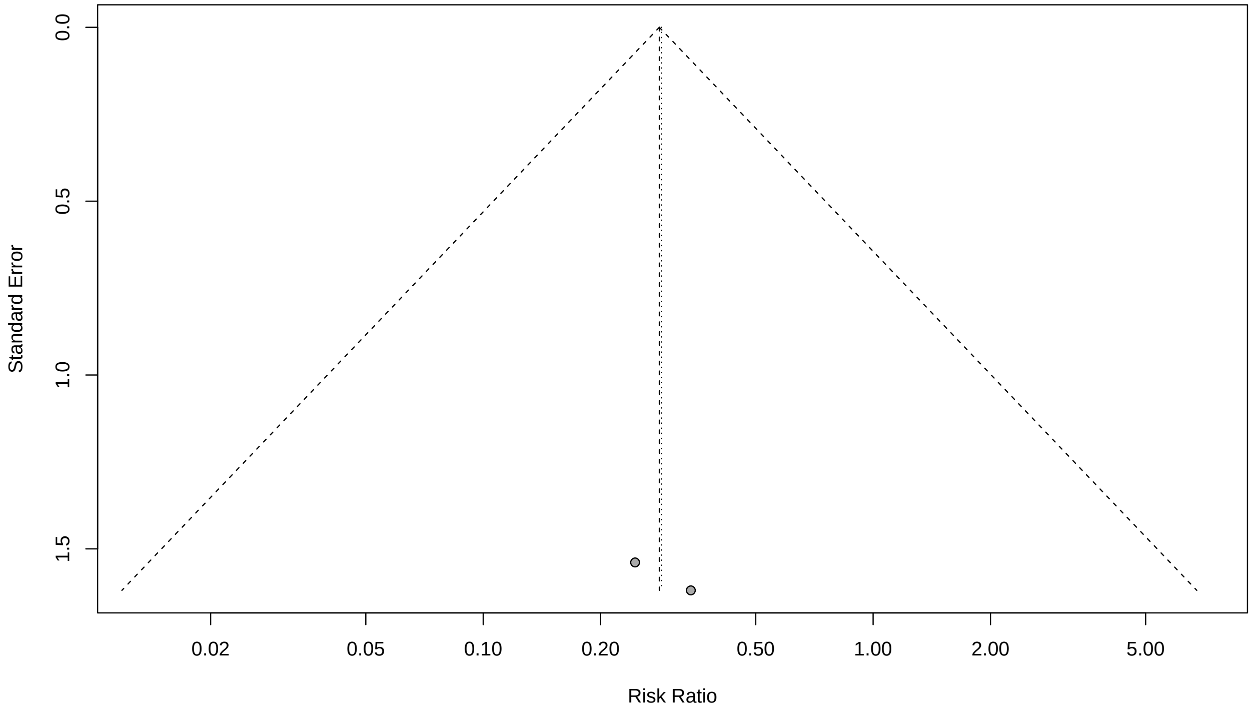

12.15.5 Baujat

```
baujat(ir_embolisation_metabin, pos = 1)
```

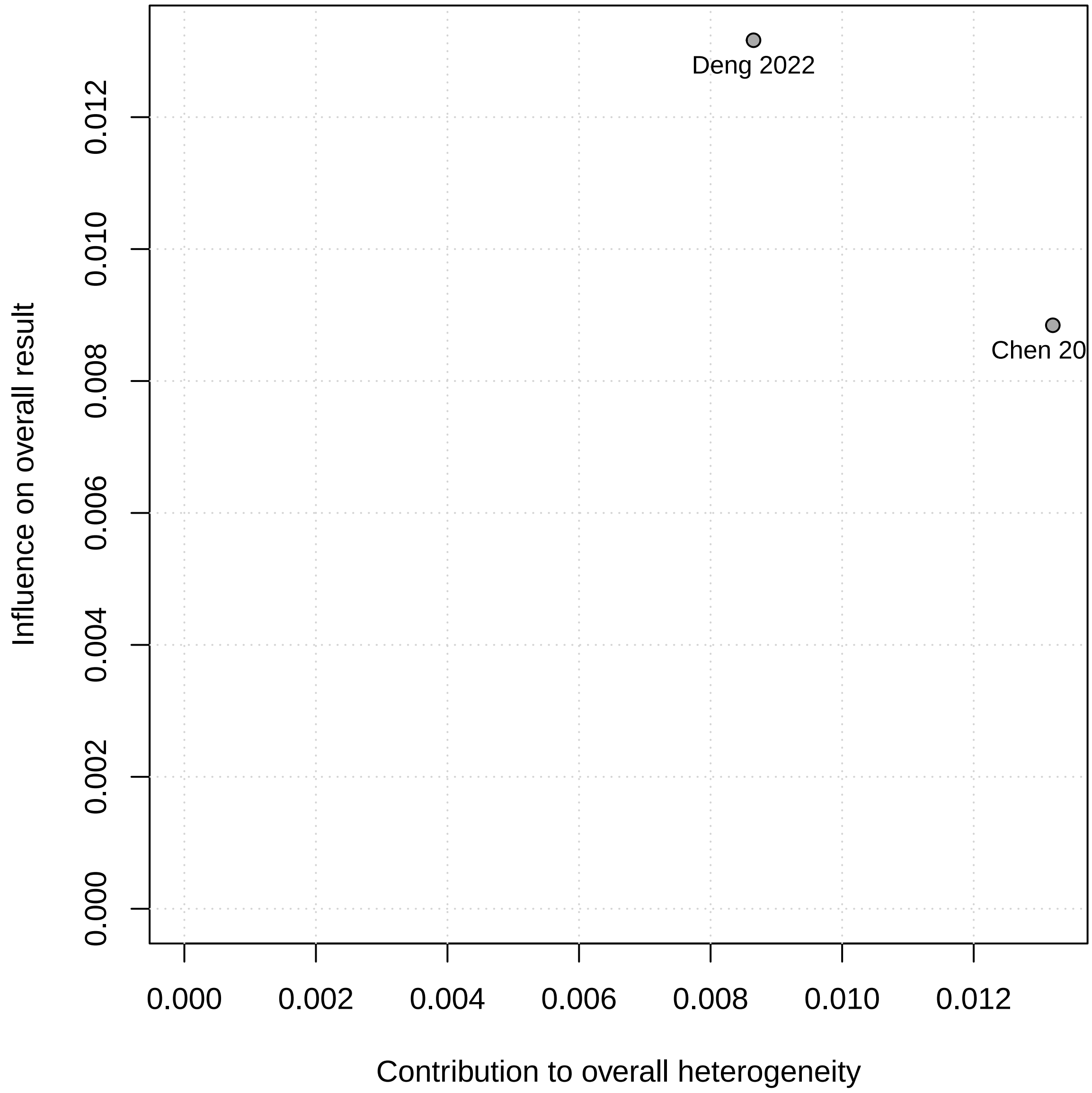

12.15.6 Leave one out

```
metainf(ir_embolisation_metabin)
```

```
## Influential analysis (common effect model)
##
##                             RR           95%-CI p-value   tau^2     tau   I^2
## Omitting Tang 2023   0.2830 [0.0318; 2.5161]  0.2575  0.0000  0.0000  0.0%
## Omitting Chen 2019   0.2448 [0.0120; 4.9992]  0.3605
## Omitting Lai 2020    0.2830 [0.0318; 2.5161]  0.2575  0.0000  0.0000  0.0%
## Omitting Deng 2022   0.3407 [0.0143; 8.1456]  0.5062
##
## Pooled estimate      0.2830 [0.0318; 2.5161]  0.2575  0.0000  0.0000  0.0%
##
## Details on meta-analytical method:
## - Mantel-Haenszel method
## - Restricted maximum-likelihood estimator for tau^2
```

12.16 Transfusion

12.16.1 Meta-analysis

```
transfusion_metabin <- metabin(data = minipcnl_only,
                          event.c = transfusion_n_control,
                        n.c = sample_size_control,
                        event.e = transfusion_n_suction,
                        n.e = sample_size_suction,
                        studlab = author_year)
transfusion_metabin
```

```
## Number of studies: k = 3
## Number of observations: o = 473
## Number of events: e = 12
##
##                           RR           95%-CI     z p-value
## Common effect model  0.1445 [0.0280; 0.7467] -2.31  0.0210
## Random effects model 0.1632 [0.0302; 0.8826] -2.10  0.0353
##
## Quantifying heterogeneity:
##  tau^2 = 0 [0.0000; 22.9527]; tau = 0 [0.0000; 4.7909]
##  I^2 = 0.0% [0.0%; 89.6%]; H = 1.00 [1.00; 3.10]
##
## Test of heterogeneity:
##     Q d.f. p-value
##  0.57    2  0.7525
##
## Details on meta-analytical method:
## - Mantel-Haenszel method
## - Inverse variance method
## - Restricted maximum-likelihood estimator for tau^2
## - Q-Profile method for confidence interval of tau^2 and tau
## - Continuity correction of 0.5 in studies with zero cell frequencies
```

12.16.2 Forest plot

```r
forest(transfusion_metabin,
       sortvar = TE)
```

Study	Experimental Events	Experimental Total	Control Events	Control Total	Risk Ratio	RR	95%−CI	Weight (common)	Weight (random)
Deng 2022	0	57	7	70		0.08	[0.00; 1.40]	56.7%	35.3%
Chen 2019	0	44	3	45		0.15	[0.01; 2.75]	29.1%	33.1%
Lai 2020	0	28	2	56		0.40	[0.02; 7.99]	14.2%	31.6%
Tang 2023	0	86	0	87				0.0%	0.0%
**Common effect model**		**215**		**258**		**0.14**	**[0.03; 0.75]**	**100.0%**	**−−**
**Random effects model**						**0.16**	**[0.03; 0.88]**	**−−**	**100.0%**

Heterogeneity: $I^2 = 0\%$, $\tau^2 = 0$, $p = 0.75$

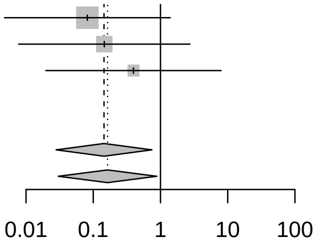

12.16.3 Trim and Fill

```r
trimfill(transfusion_metabin)
```

```
## Warning in trimfill.meta(transfusion_metabin): 1 observation(s) dropped due to
## missing values
```

```
## Number of studies: k = 3 (with 0 added studies)
## Number of observations: o = 300
## Number of events: e = 12
##
##                             RR           95%-CI     z p-value
## Random effects model 0.1632 [0.0302; 0.8826] -2.10  0.0353
##
## Quantifying heterogeneity:
##  tau^2 = 0 [0.0000; 22.9527]; tau = 0 [0.0000; 4.7909]
##  I^2 = 0.0% [0.0%; 89.6%]; H = 1.00 [1.00; 3.10]
##
## Test of heterogeneity:
##     Q d.f. p-value
##  0.57    2  0.7525
##
## Details on meta-analytical method:
## - Inverse variance method
## - Restricted maximum-likelihood estimator for tau^2
## - Q-Profile method for confidence interval of tau^2 and tau
## - Trim-and-fill method to adjust for funnel plot asymmetry (L-estimator)
```

```r
forest(trimfill(transfusion_metabin),
       sortvar = TE)
```

```
## Warning in trimfill.meta(transfusion_metabin): 1 observation(s) dropped due to
## missing values
```

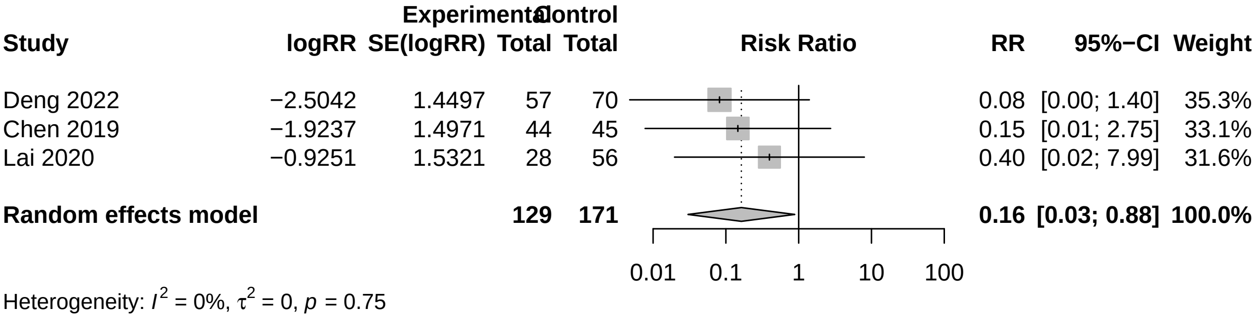

12.16.4 Funnel plot

```r
funnel(trimfill(transfusion_metabin))
```

```
## Warning in trimfill.meta(transfusion_metabin): 1 observation(s) dropped due to
## missing values
```

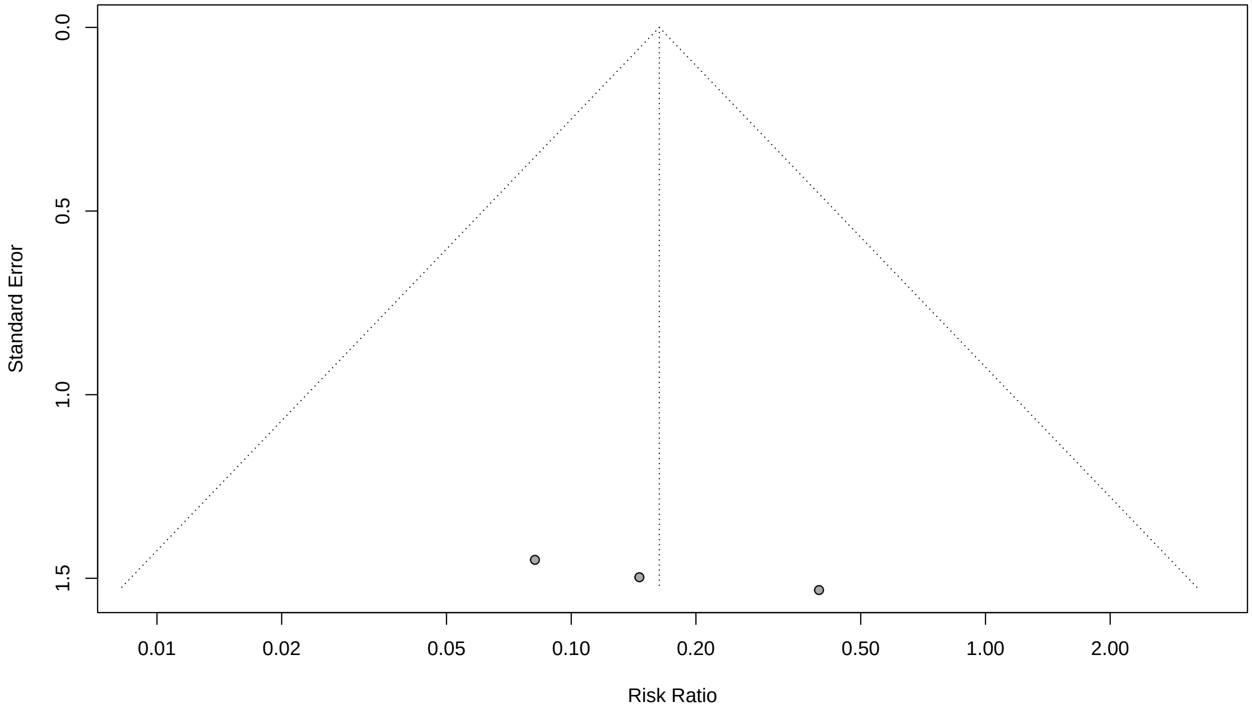

12.16.5 Baujat

```r
baujat(transfusion_metabin, pos = 1)
```

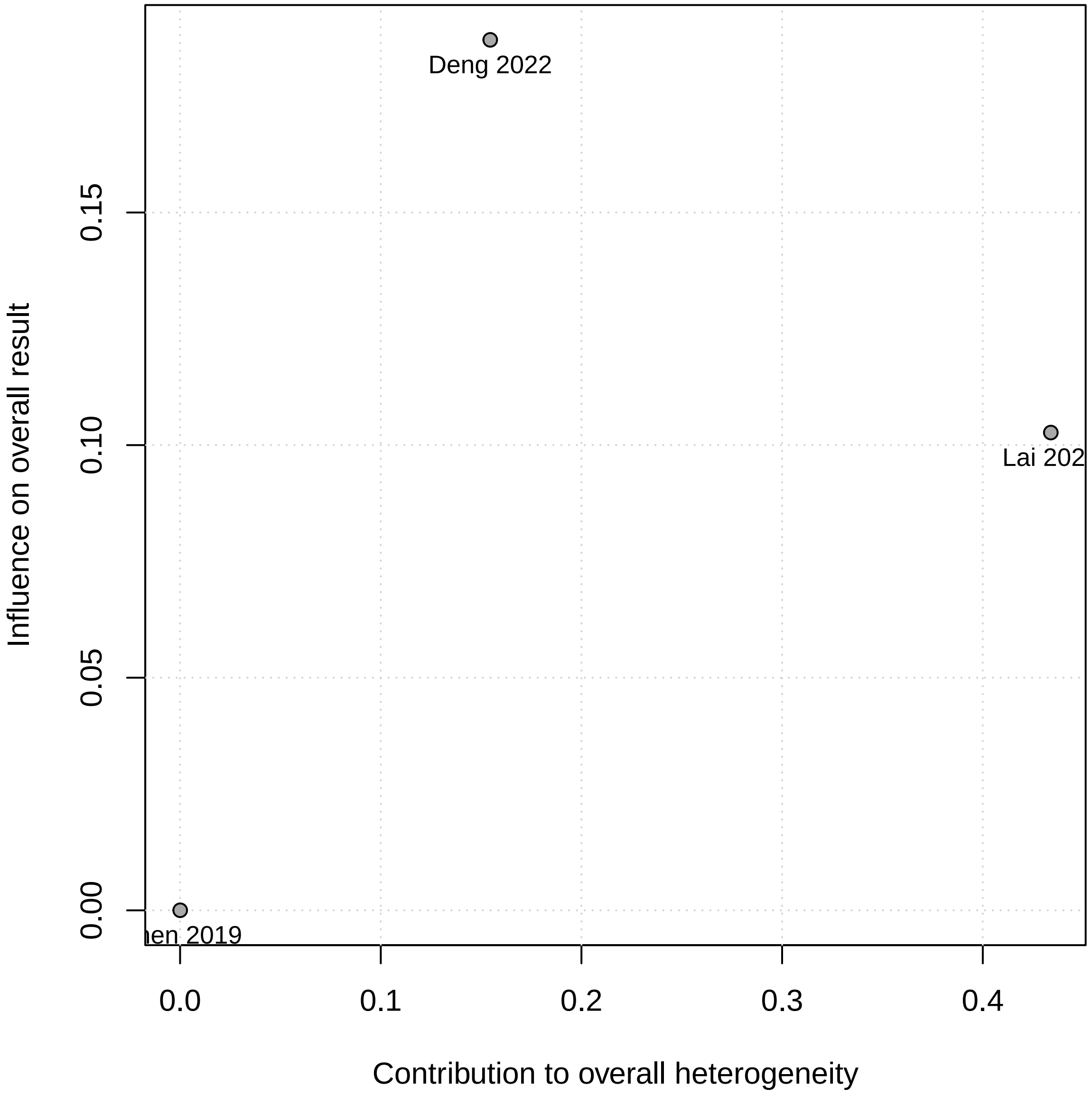

12.16.6 Leave one out

```r
metainf(transfusion_metabin)
```

```
## Influential analysis (common effect model)
##
##                           RR           95%-CI p-value   tau^2    tau   I^2
## Omitting Tang 2023   0.1445 [0.0280; 0.7467]  0.0210  0.0000  0.0000  0.0%
## Omitting Chen 2019   0.1439 [0.0199; 1.0412]  0.0549  0.0000  0.0000  0.0%
## Omitting Lai 2020    0.1035 [0.0134; 0.7992]  0.0296  0.0000  0.0000  0.0%
## Omitting Deng 2022   0.2270 [0.0294; 1.7540]  0.1552  0.0000  0.0000  0.0%
##
## Pooled estimate      0.1445 [0.0280; 0.7467]  0.0210  0.0000  0.0000  0.0%
##
## Details on meta-analytical method:
## - Mantel-Haenszel method
## - Restricted maximum-likelihood estimator for tau^2
```

12.17 Clavien I

12.17.1 Meta-analysis

```
clav_i_metabin <- metabin(data = minipcnl_only,
                           event.c = clavien_i_n_control,
                         n.c = sample_size_control,
                         event.e = clavien_i_n_suction,
                         n.e = sample_size_suction,
                         studlab = author_year)
clav_i_metabin
```

```
## Number of studies: k = 2
## Number of observations: o = 473
## Number of events: e = 26
##
##                           RR           95%-CI     z p-value
## Common effect model  0.5950 [0.2749; 1.2876] -1.32  0.1874
## Random effects model 0.5930 [0.2731; 1.2874] -1.32  0.1864
##
## Quantifying heterogeneity:
##  tau^2 = 0; tau = 0; I^2 = 0.0%; H = 1.00
##
## Test of heterogeneity:
##     Q d.f. p-value
##  0.22    1  0.6373
##
## Details on meta-analytical method:
## - Mantel-Haenszel method
## - Inverse variance method
## - Restricted maximum-likelihood estimator for tau^2
```

12.17.2 Forest plot

```r
forest(clav_i_metabin,
       sortvar = TE)
```

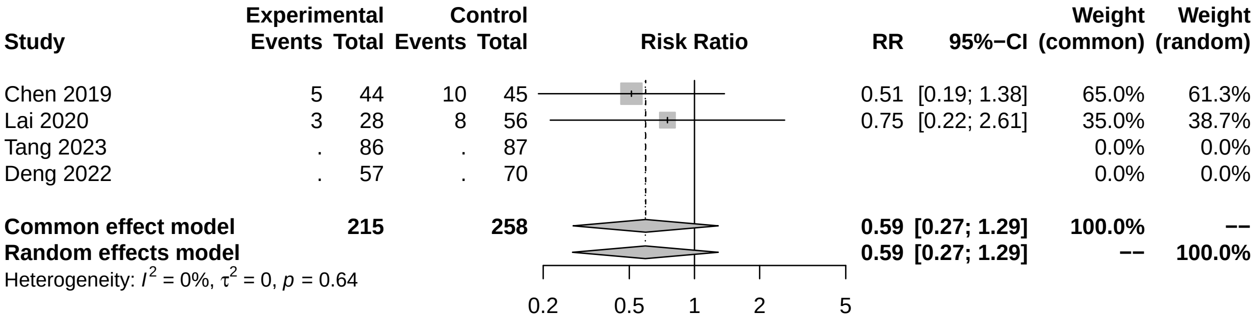

12.17.3 Trim and Fill

```
#trimfill(clav_i_metabin)
#forest(trimfill(clav_i_metabin),
#       sortvar = TE)
```

12.17.4 Funnel plot

```r
funnel((clav_i_metabin))
```

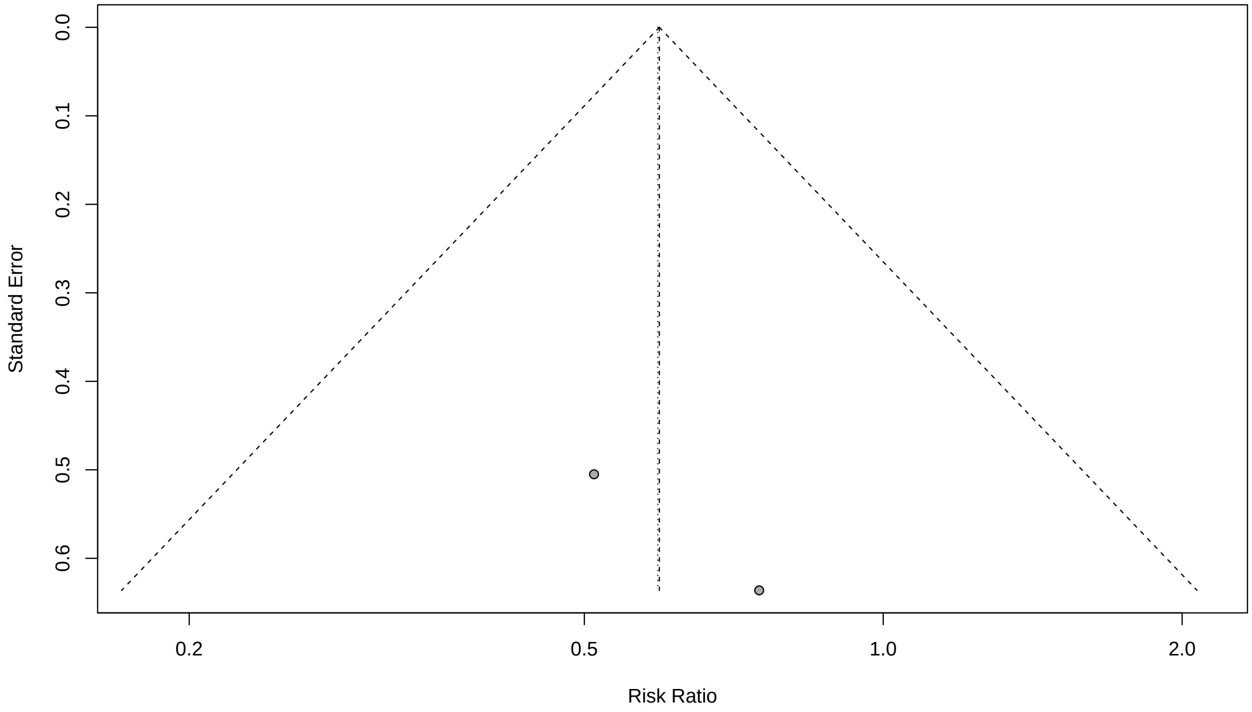

12.17.5 Baujat

```r
baujat(clav_i_metabin, pos = 1)
```

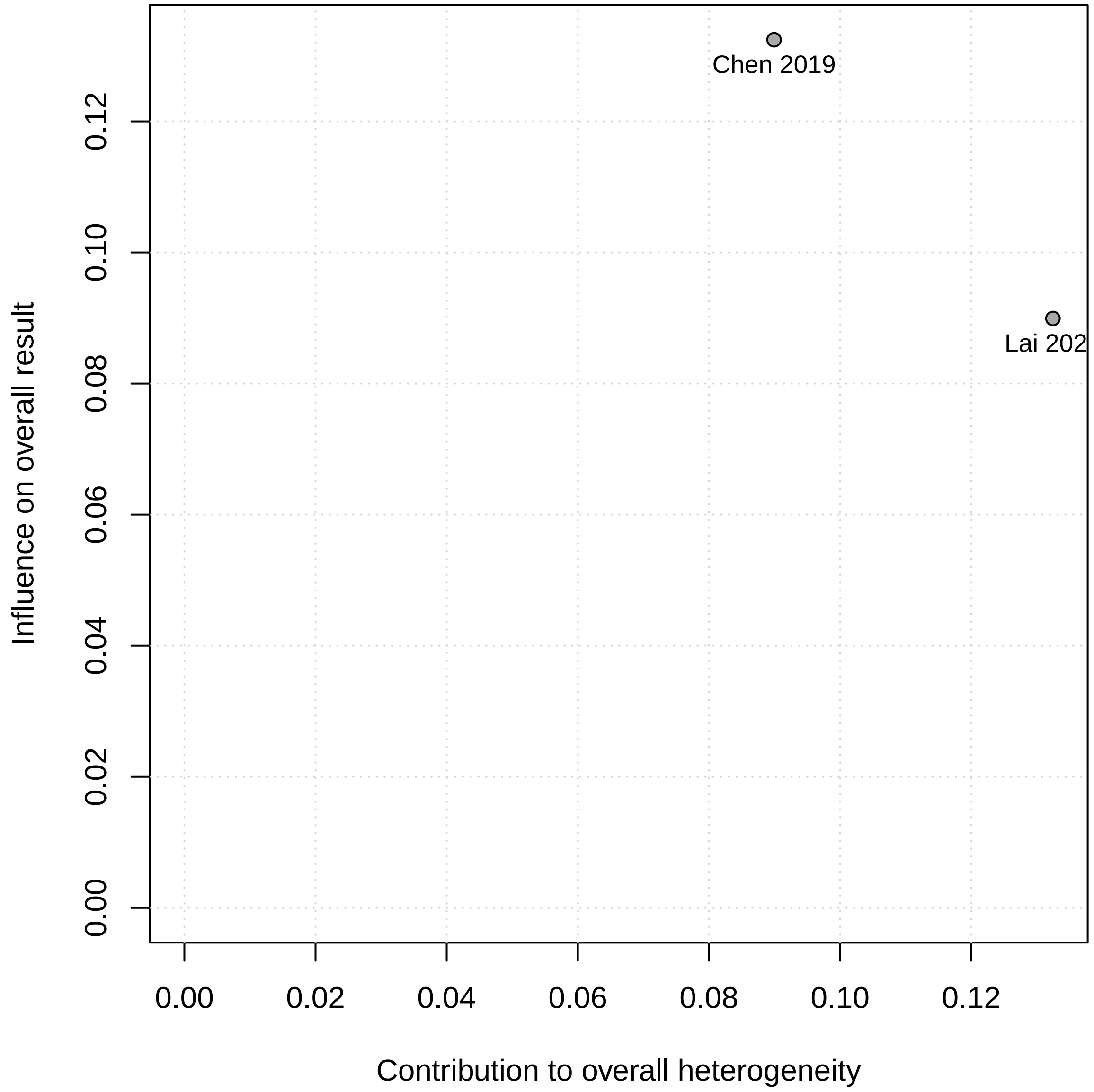

12.17.6 Leave one out

```r
metainf(clav_i_metabin)
```

```
## Influential analysis (common effect model)
##
##                          RR           95%-CI p-value   tau^2     tau   I^2
## Omitting Tang 2023   0.5950 [0.2749; 1.2876]  0.1874  0.0000  0.0000  0.0%
## Omitting Chen 2019   0.7500 [0.2155; 2.6098]  0.6511
## Omitting Lai 2020    0.5114 [0.1900; 1.3760]  0.1842
## Omitting Deng 2022   0.5950 [0.2749; 1.2876]  0.1874  0.0000  0.0000  0.0%
##
## Pooled estimate      0.5950 [0.2749; 1.2876]  0.1874  0.0000  0.0000  0.0%
##
## Details on meta-analytical method:
## - Mantel-Haenszel method
## - Restricted maximum-likelihood estimator for tau^2
```

12.18 Clavien II

12.18.1 Meta-analysis

```
clav_ii_metabin <- metabin(data = minipcnl_only,
                             event.c = clavien_ii_n_control,
                           n.c = sample_size_control,
                           event.e = clavien_ii_n_suction,
                           n.e = sample_size_suction,
                           studlab = author_year)
clav_ii_metabin
```

```
## Number of studies: k = 2
## Number of observations: o = 473
## Number of events: e = 8
##
##                          RR           95%-CI     z p-value
## Common effect model  0.3001 [0.0565; 1.5932] -1.41  0.1576
## Random effects model 0.3258 [0.0577; 1.8397] -1.27  0.2042
##
## Quantifying heterogeneity:
##  tau^2 = 0; tau = 0; I^2 = 0.0%; H = 1.00
##
## Test of heterogeneity:
##     Q d.f. p-value
##  0.44    1  0.5069
##
## Details on meta-analytical method:
## - Mantel-Haenszel method
## - Inverse variance method
## - Restricted maximum-likelihood estimator for tau^2
## - Continuity correction of 0.5 in studies with zero cell frequencies
```

12.18.2 Forest plot

```r
forest(clav_ii_metabin,
       sortvar = TE)
```

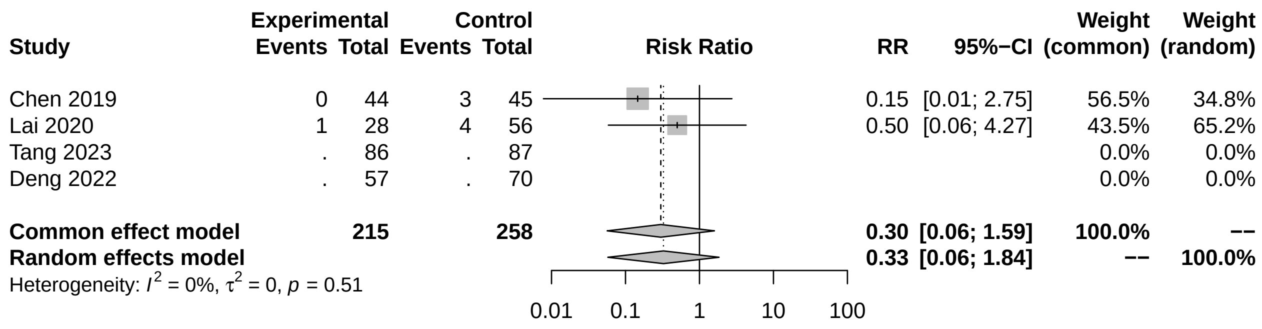

12.18.3 Trim and Fill

```
#trimfill(clav_ii_metabin)
#forest(trimfill(clav_ii_metabin),
#       sortvar = TE)
```

12.18.4 Funnel plot

```
funnel((clav_ii_metabin))
```

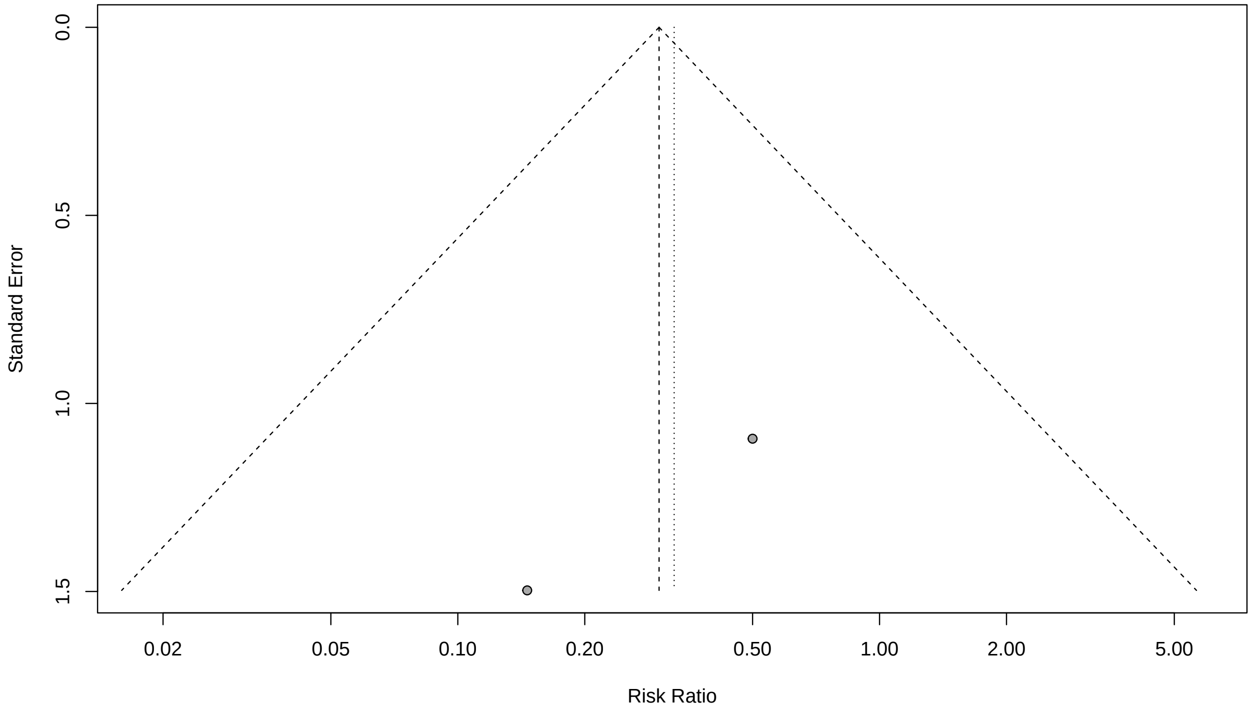

12.18.5 Baujat

```r
baujat(clav_ii_metabin, pos = 1)
```

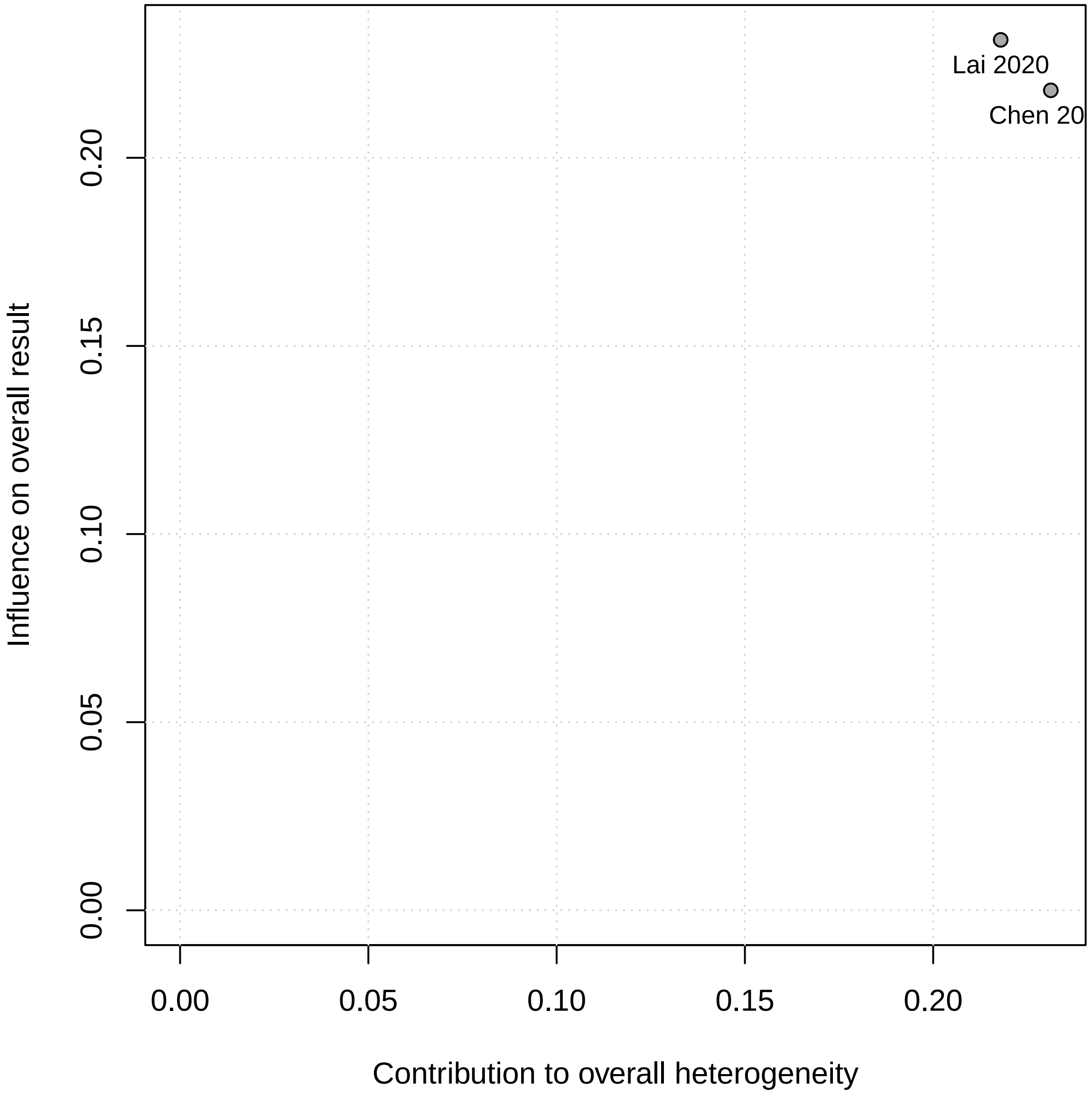

12.18.6 Leave one out

```
metainf(clav_ii_metabin)
```

```
## Influential analysis (common effect model)
##
##                          RR           95%-CI p-value   tau^2    tau   I^2
## Omitting Tang 2023   0.3001 [0.0565; 1.5932]  0.1576  0.0000  0.0000  0.0%
## Omitting Chen 2019   0.5000 [0.0586; 4.2661]  0.5263
## Omitting Lai 2020    0.1460 [0.0078; 2.7473]  0.1988
## Omitting Deng 2022   0.3001 [0.0565; 1.5932]  0.1576  0.0000  0.0000  0.0%
##
## Pooled estimate      0.3001 [0.0565; 1.5932]  0.1576  0.0000  0.0000  0.0%
##
## Details on meta-analytical method:
## - Mantel-Haenszel method
## - Restricted maximum-likelihood estimator for tau^2
```

12.19 Clavien III

12.19.1 Meta-analysis

```
clav_iii_metabin <- metabin(data = minipcnl_only,
                         event.c = clavien_iii_n_control,
                       n.c = sample_size_control,
                       event.e = clavien_iii_n_suction,
                       n.e = sample_size_suction,
                       studlab = author_year)
clav_iii_metabin
```

```
## Number of studies: k = 2
## Number of observations: o = 473
## Number of events: e = 4
##
##                             RR           95%-CI     z p-value
## Common effect model  0.6528 [0.1042; 4.0882] -0.46  0.6487
## Random effects model 0.6820 [0.1028; 4.5254] -0.40  0.6919
##
## Quantifying heterogeneity:
##  tau^2 = 0; tau = 0; I^2 = 0.0%; H = 1.00
##
## Test of heterogeneity:
##     Q d.f. p-value
##  0.28    1  0.5936
##
## Details on meta-analytical method:
## - Mantel-Haenszel method
## - Inverse variance method
## - Restricted maximum-likelihood estimator for tau^2
## - Continuity correction of 0.5 in studies with zero cell frequencies
```

12.19.2 Forest plot

```r
forest(clav_iii_metabin,
       sortvar = TE)
```

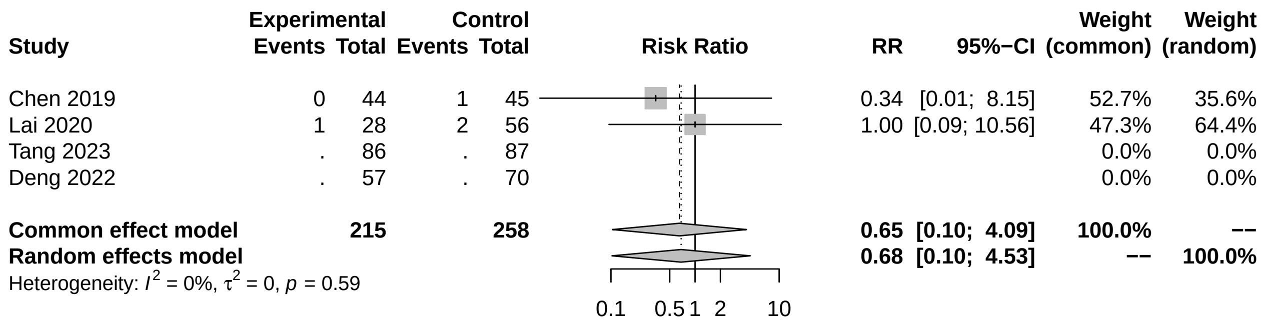

12.19.3 Trim and Fill

```
#trimfill(clav_iii_metabin)
#forest(trimfill(clav_iii_metabin),
#       sortvar = TE)
```

12.19.4 Funnel plot

```
funnel((clav_iii_metabin))
```

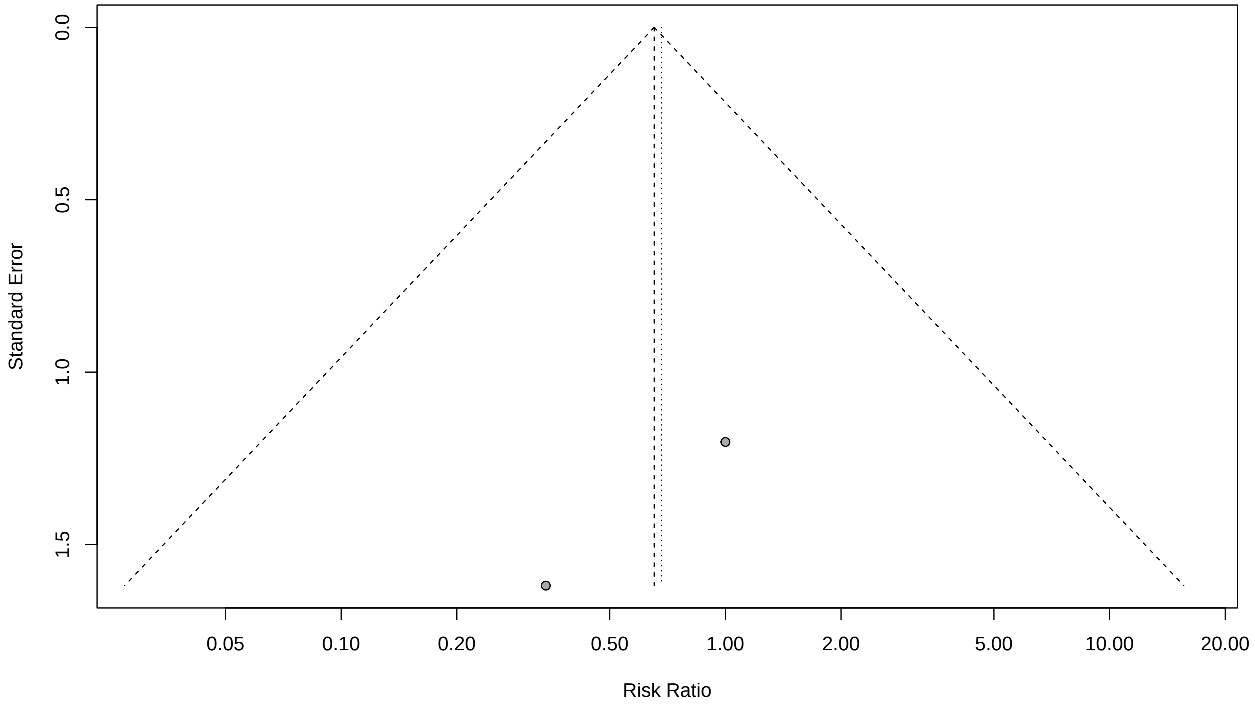

12.19.5 Baujat

```
baujat(clav_iii_metabin, pos = 1)
```

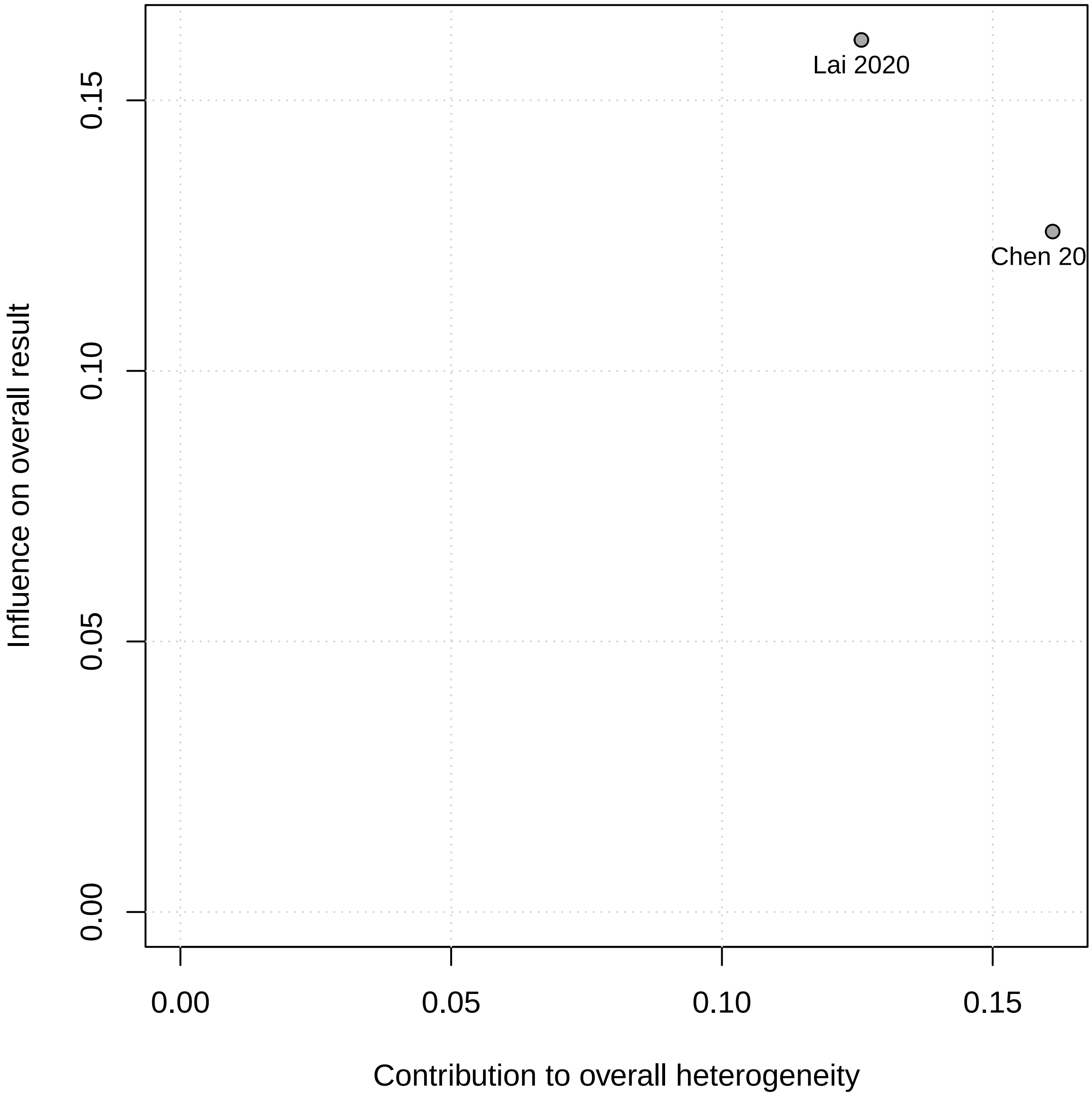

12.19.6 Leave one out

```
metainf(clav_iii_metabin)
```

```
## Influential analysis (common effect model)
##
##                              RR            95%-CI p-value   tau^2     tau   I^2
## Omitting Tang 2023   0.6528 [0.1042;  4.0882]  0.6487  0.0000  0.0000  0.0%
## Omitting Chen 2019   1.0000 [0.0947; 10.5613]  1.0000
## Omitting Lai 2020    0.3407 [0.0143;  8.1456]  0.5062
## Omitting Deng 2022   0.6528 [0.1042;  4.0882]  0.6487  0.0000  0.0000  0.0%
##
## Pooled estimate      0.6528 [0.1042;  4.0882]  0.6487  0.0000  0.0000  0.0%
##
## Details on meta-analytical method:
## - Mantel-Haenszel method
## - Restricted maximum-likelihood estimator for tau^2
```

12.20 Clavien IV

12.20.1 Meta-analysis

```
clav_iv_metabin <- metabin(data = minipcnl_only,
                         event.c = clavien_iv_n_control,
                       n.c = sample_size_control,
                       event.e = clavien_iv_n_suction,
                       n.e = sample_size_suction,
                       studlab = author_year)
clav_iv_metabin
```

```
## Number of studies: k = 0
## Number of observations: o = 473
## Number of events: e = 0
##
##                        RR 95%-CI  z p-value
## Common effect model  NA        --      --
## Random effects model NA        --      --
##
## Quantifying heterogeneity:
##  tau^2 = NA; tau = NA; I^2 = NA; H = NA
##
## Details on meta-analytical method:
## - Mantel-Haenszel method
## - Inverse variance method
## - Restricted maximum-likelihood estimator for tau^2
```

12.20.2 Forest plot

```r
forest(clav_iv_metabin,
       sortvar = TE)
```

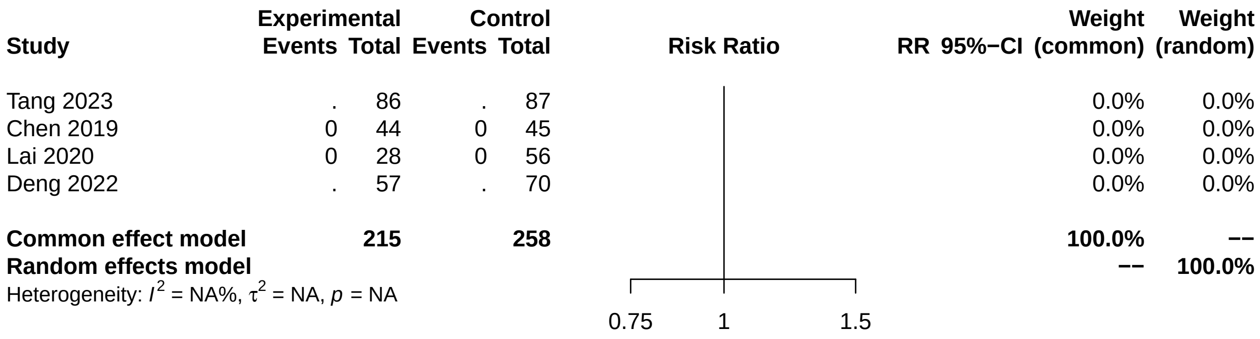

12.20.3 Trim and Fill

```
#trimfill(clav_iv_metabin)
#forest(trimfill(clav_iv_metabin),
#       sortvar = TE)
```

12.20.4 Funnel plot

```
#funnel((clav_iv_metabin))
```

12.20.5 Baujat

```
#baujat(clav_iv_metabin, pos = 1)
```

12.20.6 Leave one out

```
#metainf(clav_iv_metabin)
```

12.21 Clavien V

12.21.1 Meta-analysis

```
clav_v_metabin <- metabin(data = minipcnl_only,
                         event.c = clavien_v_n_control,
                       n.c = sample_size_control,
                       event.e = clavien_v_n_suction,
                       n.e = sample_size_suction,
                       studlab = author_year)
clav_v_metabin
```

```
## Number of studies: k = 0
## Number of observations: o = 473
## Number of events: e = 0
##
##                        RR 95%-CI  z p-value
## Common effect model  NA        --      --
## Random effects model NA        --      --
##
## Quantifying heterogeneity:
##  tau^2 = NA; tau = NA; I^2 = NA; H = NA
##
## Details on meta-analytical method:
## - Mantel-Haenszel method
## - Inverse variance method
## - Restricted maximum-likelihood estimator for tau^2
```

12.21.2 Forest plot

```
#forest(clav_v_metabin,
#       sortvar = TE)
```

12.21.3 Trim and Fill

```
#trimfill(clav_v_metabin)
#forest(trimfill(clav_v_metabin),
#       sortvar = TE)
```

12.21.4 Funnel plot

```
#funnel(trimfill(clav_v_metabin))
```

12.21.5 Baujat

```
#baujat(clav_v_metabin, pos = 1)
```

12.21.6 Leave one out

```
#metainf(clav_v_metabin)
```

12.22 Clavien I-II

12.22.1 Meta-analysis

```
clav_i_ii_metabin <- metabin(data = minipcnl_only,
                             event.c = clav_i_ii_n_control,
                           n.c = sample_size_control,
                           event.e = clav_i_ii_n_suction,
                           n.e = sample_size_suction,
                           studlab = author_year)
clav_i_ii_metabin
```

```
## Number of studies: k = 2
## Number of observations: o = 473
## Number of events: e = 34
##
##                            RR           95%-CI     z p-value
## Common effect model  0.4982 [0.2494; 0.9953] -1.97  0.0485
## Random effects model 0.4996 [0.2486; 1.0041] -1.95  0.0514
##
## Quantifying heterogeneity:
##  tau^2 = 0; tau = 0; I^2 = 0.0%; H = 1.00
##
## Test of heterogeneity:
##     Q d.f. p-value
##  0.54    1  0.4608
##
## Details on meta-analytical method:
## - Mantel-Haenszel method
## - Inverse variance method
## - Restricted maximum-likelihood estimator for tau^2
```

12.22.2 Forest plot

```r
forest(clav_i_ii_metabin,
       sortvar = TE)
```

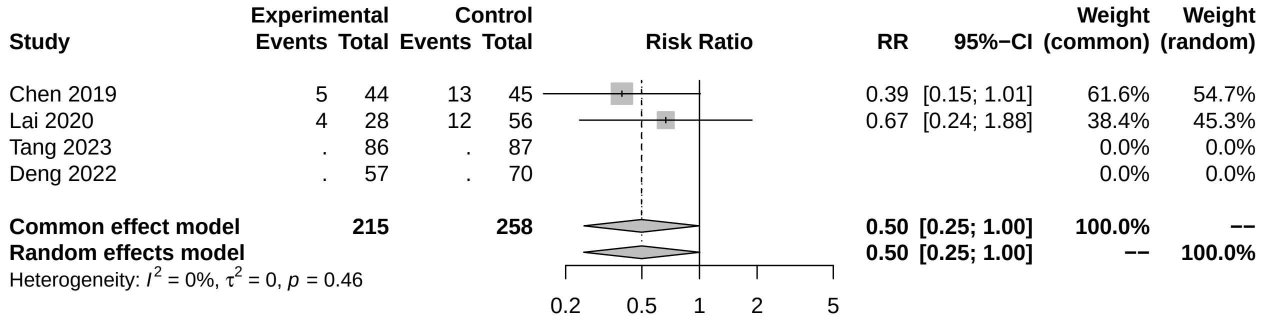

12.22.3 Trim and Fill

```
#trimfill(clav_i_ii_metabin)
#forest(trimfill(clav_i_ii_metabin),
#       sortvar = TE)
```

12.22.4 Funnel plot

```r
funnel((clav_i_ii_metabin))
```

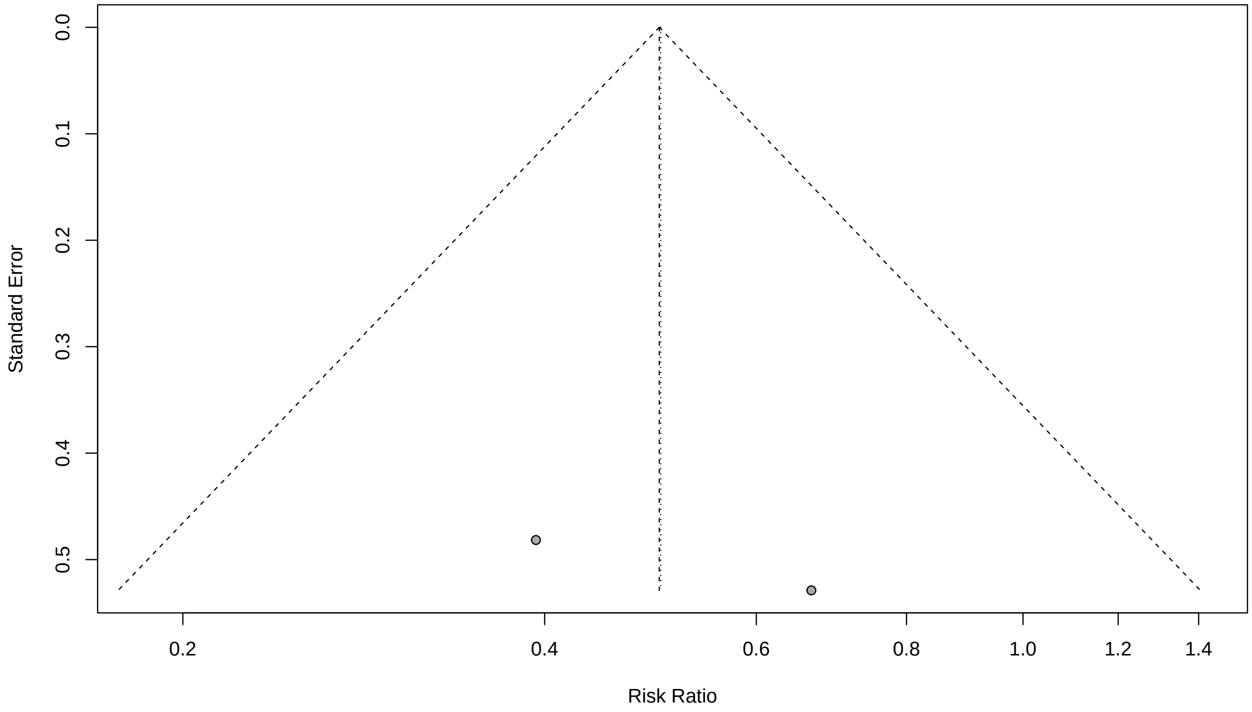

12.22.5 Baujat

```
baujat(clav_i_ii_metabin, pos = 1)
```

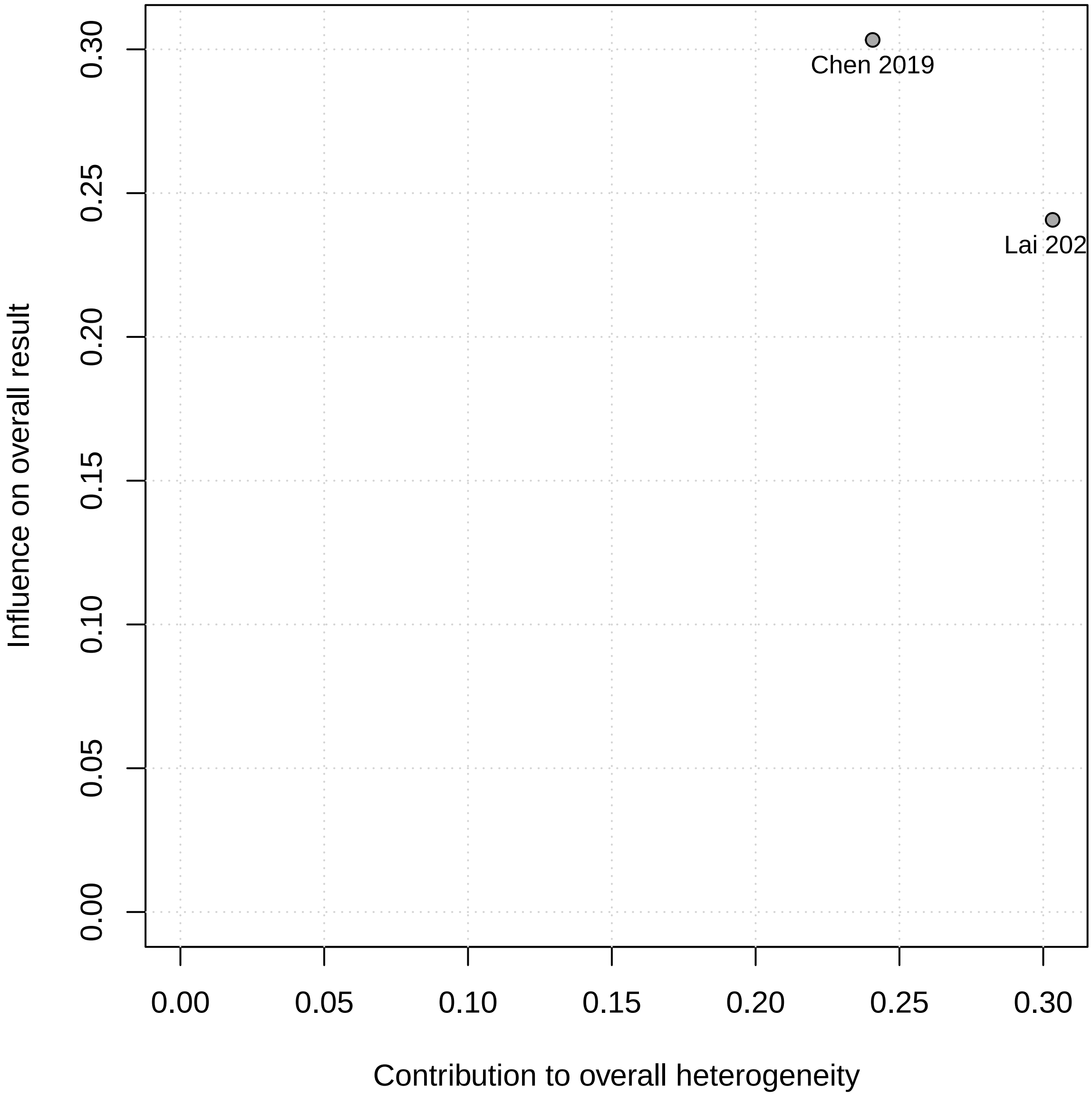

12.22.6 Leave one out

```
metainf(clav_i_ii_metabin)
```

```
## Influential analysis (common effect model)
##
##                             RR           95%-CI p-value   tau^2     tau   I^2
## Omitting Tang 2023   0.4982 [0.2494; 0.9953]  0.0485  0.0000  0.0000  0.0%
## Omitting Chen 2019   0.6667 [0.2364; 1.8799]  0.4433
## Omitting Lai 2020    0.3934 [0.1530; 1.0110]  0.0527
## Omitting Deng 2022   0.4982 [0.2494; 0.9953]  0.0485  0.0000  0.0000  0.0%
##
## Pooled estimate      0.4982 [0.2494; 0.9953]  0.0485  0.0000  0.0000  0.0%
##
## Details on meta-analytical method:
## - Mantel-Haenszel method
## - Restricted maximum-likelihood estimator for tau^2
```

12.23 Clavien III-V

12.23.1 Meta-analysis

```
clav_iii_v_metabin <- metabin(data = minipcnl_only,
                         event.c = clav_iii_v_n_control,
                       n.c = sample_size_control,
                       event.e = clav_iii_v_n_suction,
                       n.e = sample_size_suction,
                       studlab = author_year)
clav_iii_v_metabin
```

```
## Number of studies: k = 2
## Number of observations: o = 473
## Number of events: e = 4
##
##                          RR           95%-CI     z p-value
## Common effect model  0.6528 [0.1042; 4.0882] -0.46  0.6487
## Random effects model 0.6820 [0.1028; 4.5254] -0.40  0.6919
##
## Quantifying heterogeneity:
##  tau^2 = 0; tau = 0; I^2 = 0.0%; H = 1.00
##
## Test of heterogeneity:
##     Q d.f. p-value
##  0.28    1  0.5936
##
## Details on meta-analytical method:
## - Mantel-Haenszel method
## - Inverse variance method
## - Restricted maximum-likelihood estimator for tau^2
## - Continuity correction of 0.5 in studies with zero cell frequencies
```

12.23.2 Forest plot

```
forest(clav_iii_v_metabin,
       sortvar = TE)
```

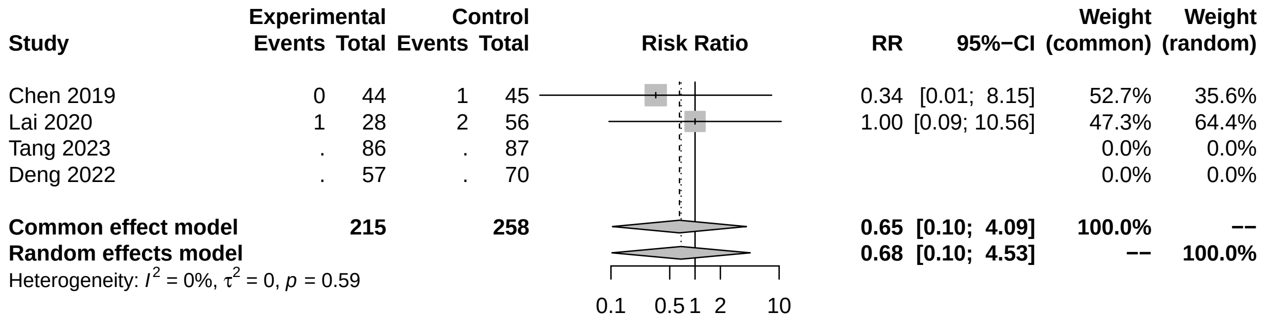

12.23.3 Trim and Fill

```
#trimfill(clav_iii_v_metabin)
#forest(trimfill(clav_iii_v_metabin),
#       sortvar = TE)
```

12.23.4 Funnel plot

```
funnel((clav_iii_v_metabin))
```

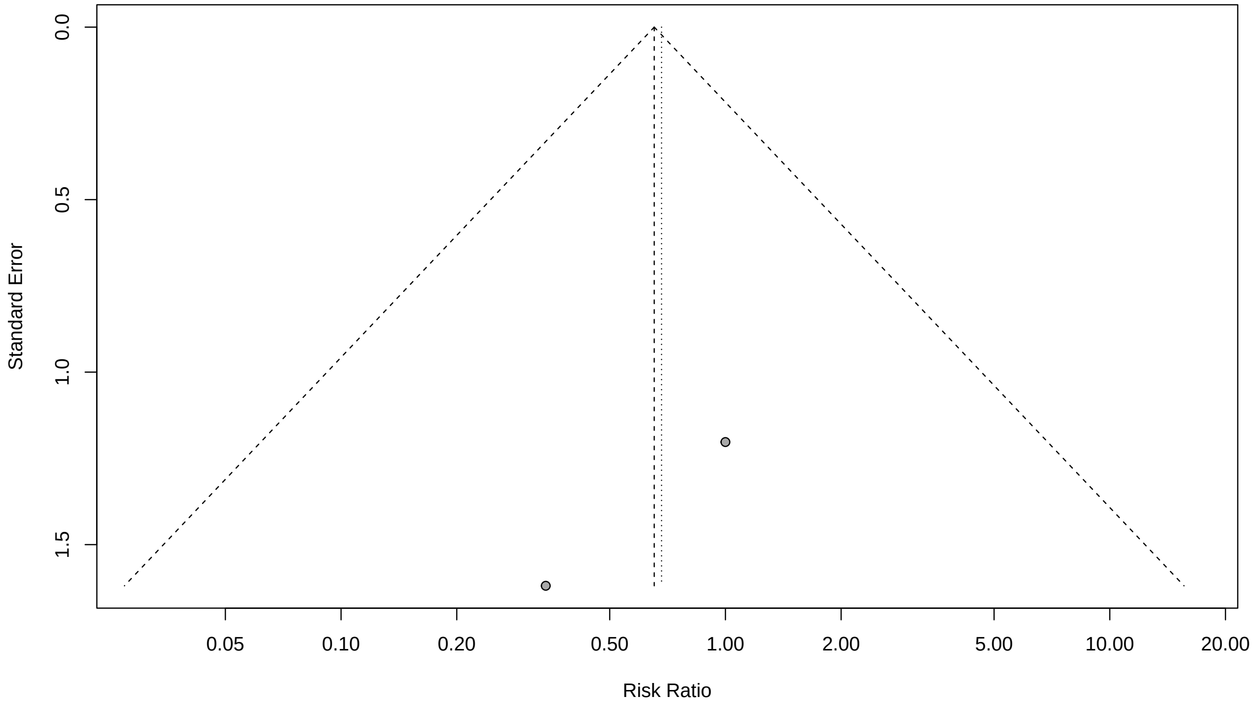

12.23.5 Baujat

```
baujat(clav_iii_v_metabin, pos = 1)
```

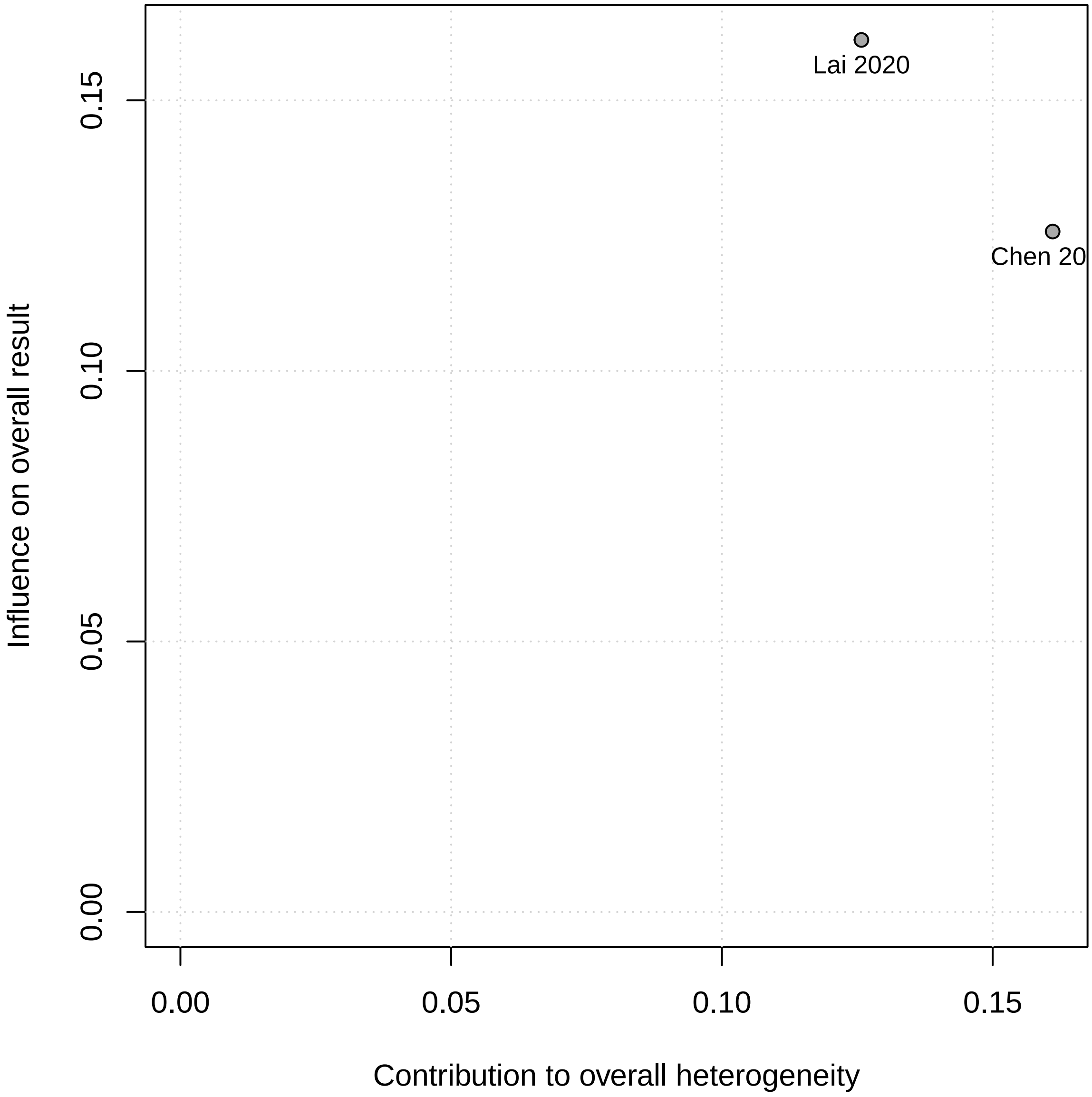

12.23.6 Leave one out

```r
metainf(clav_iii_v_metabin)
```

```
## Influential analysis (common effect model)
##
##                          RR            95%-CI p-value   tau^2    tau   I^2
## Omitting Tang 2023   0.6528 [0.1042;  4.0882]  0.6487  0.0000  0.0000  0.0%
## Omitting Chen 2019   1.0000 [0.0947; 10.5613]  1.0000
## Omitting Lai 2020    0.3407 [0.0143;  8.1456]  0.5062
## Omitting Deng 2022   0.6528 [0.1042;  4.0882]  0.6487  0.0000  0.0000  0.0%
##
## Pooled estimate      0.6528 [0.1042;  4.0882]  0.6487  0.0000  0.0000  0.0%
##
## Details on meta-analytical method:
## - Mantel-Haenszel method
## - Restricted maximum-likelihood estimator for tau^2
```

13 Mini-PCNL vs Flexi URS Summary Forest plots

```
overall <- cbind(
  "Outcome" = c(
    "Immediate SFR",
    "Final SFR",
    "Auxiliary Treatment",
    "Overall Complications",
    "Fever",
    "Infection",
    "Sepsis",
    "Abscess",
    "Haematoma",
    "Pain",
    "Stricture",
    "Embolisation",
    "Transfusion",
    "CD I",
    "CD II",
    "CD III",
    "CD IV",
    "CD I-II",
    "CD III-V",
    "Operative time",
    "VAS",
    "Length of Stay"
  ),
  "type" = c(
    "binary",
    "binary",
    "binary",
    "binary",
    "binary",
    "binary",
    "binary",
    "binary",
    "binary",
    "binary",
    "binary",
    "binary",
    "binary",
    "binary",
    "binary",
    "binary",
    "binary",
    "binary",
    "binary",
    "cont",
    "cont",
    "cont"
  ),
  "n_studies" = c(
    immediate_sfr_metabin$k,
```

```
  sfr_metabin$k,
aux_rx_metabin$k,
comp_metabin$k,
fever_metabin$k,
infection_metabin$k,
sepsis_metabin$k,
abscess_metabin$k,
haematoma_metabin$k,
pain_metabin$k,
stricture_metabin$k,
ir_embolisation_metabin$k,
transfusion_metabin$k,
clav_i_metabin$k,
clav_ii_metabin$k,
clav_iii_metabin$k,
clav_iv_metabin$k,
clav_i_ii_metabin$k,
clav_iii_v_metabin$k,
or_time_metacont$k,
vas_metacont$k,
los_metacont$k),
"es" = c(
  immediate_sfr_metabin$TE.random %>% exp(),
  sfr_metabin$TE.random %>% exp(),
aux_rx_metabin$TE.random %>% exp(),
comp_metabin$TE.random %>% exp(),
fever_metabin$TE.random %>% exp(),
infection_metabin$TE.random %>% exp(),
sepsis_metabin$TE.random %>% exp(),
abscess_metabin$TE.random %>% exp(),
haematoma_metabin$TE.random %>% exp(),
pain_metabin$TE.random %>% exp(),
stricture_metabin$TE.random %>% exp(),
ir_embolisation_metabin$TE.random %>% exp(),
transfusion_metabin$TE.random %>% exp(),
clav_i_metabin$TE.random %>% exp(),
clav_ii_metabin$TE.random %>% exp(),
clav_iii_metabin$TE.random %>% exp(),
clav_iv_metabin$TE.random %>% exp(),
clav_i_ii_metabin$TE.random %>% exp(),
clav_iii_v_metabin$TE.random %>% exp(),
or_time_metacont$TE.random,
vas_metacont$TE.random,
los_metacont$TE.random),
"lower_ci" = c(
  immediate_sfr_metabin$lower.random %>% exp(),
  sfr_metabin$lower.random %>% exp(),
aux_rx_metabin$lower.random %>% exp(),
comp_metabin$lower.random %>% exp(),
fever_metabin$lower.random %>% exp(),
infection_metabin$lower.random %>% exp(),
sepsis_metabin$lower.random %>% exp(),
abscess_metabin$lower.random %>% exp(),
```

```r
haematoma_metabin$lower.random %>% exp(),
pain_metabin$lower.random %>% exp(),
stricture_metabin$lower.random %>% exp(),
ir_embolisation_metabin$lower.random %>% exp(),
transfusion_metabin$lower.random %>% exp(),
clav_i_metabin$lower.random %>% exp(),
clav_ii_metabin$lower.random %>% exp(),
clav_iii_metabin$lower.random %>% exp(),
clav_iv_metabin$lower.random %>% exp(),
clav_i_ii_metabin$lower.random %>% exp(),
clav_iii_v_metabin$lower.random %>% exp(),
or_time_metacont$lower.random,
vas_metacont$lower.random,
los_metacont$lower.random),
"upper_ci" = c(immediate_sfr_metabin$upper.random %>% exp(),
               sfr_metabin$upper.random %>% exp(),
aux_rx_metabin$upper.random %>% exp(),
comp_metabin$upper.random %>% exp(),
fever_metabin$upper.random %>% exp(),
infection_metabin$upper.random %>% exp(),
sepsis_metabin$upper.random %>% exp(),
abscess_metabin$upper.random %>% exp(),
haematoma_metabin$upper.random %>% exp(),
pain_metabin$upper.random %>% exp(),
stricture_metabin$upper.random %>% exp(),
ir_embolisation_metabin$upper.random %>% exp(),
transfusion_metabin$upper.random %>% exp(),
clav_i_metabin$upper.random %>% exp(),
clav_ii_metabin$upper.random %>% exp(),
clav_iii_metabin$upper.random %>% exp(),
clav_iv_metabin$upper.random %>% exp(),
clav_i_ii_metabin$upper.random %>% exp(),
clav_iii_v_metabin$upper.random %>% exp(),
or_time_metacont$upper.random,
vas_metacont$upper.random,
los_metacont$upper.random),
"p" = c(
  immediate_sfr_metabin$pval.random,
  sfr_metabin$pval.random,
aux_rx_metabin$pval.random,
comp_metabin$pval.random,
fever_metabin$pval.random,
infection_metabin$pval.random,
sepsis_metabin$pval.random,
abscess_metabin$pval.random,
haematoma_metabin$pval.random,
pain_metabin$pval.random,
stricture_metabin$pval.random,
ir_embolisation_metabin$pval.random,
transfusion_metabin$pval.random,
clav_i_metabin$pval.random,
clav_ii_metabin$pval.random,
clav_iii_metabin$pval.random,
```

```r
clav_iv_metabin$pval.random,
clav_i_ii_metabin$pval.random,
clav_iii_v_metabin$pval.random,
or_time_metacont$pval.random,
vas_metacont$pval.random,
los_metacont$pval.random)) %>% as_tibble() %>% drop_na(es)

overall$es <- as.numeric(overall$es)
overall$es <- round(overall$es, digits = 2)

overall$lower_ci <- as.numeric(overall$lower_ci)
overall$lower_ci <- round(overall$lower_ci, digits = 2)

overall$upper_ci <- as.numeric(overall$upper_ci)
overall$upper_ci <- round(overall$upper_ci, digits = 2)

overall$p <- as.numeric(overall$p)
overall$p <- round(overall$p, digits = 2)
overall$p <- ifelse(overall$p<0.001, "<0.001", overall$p)
```

13.1 Summary Table of number of studies for each outcome included in meta-analysis

```
overall %>% subset(select = c(Outcome, n_studies)) %>% gt() %>% tab_header(title = "Summary table of Nu
```

Summary table of Number of Studies for Each Outcome Meta-Analyis

Outcome	Studies, n
Immediate SFR	9
Final SFR	4
Auxiliary Treatment	2
Overall Complications	4
Fever	4
Infection	4
Sepsis	1
Haematoma	1
Pain	2
Embolisation	2
Transfusion	3
CD I	2
CD II	2
CD III	2
CD I-II	2
CD III-V	2
Operative time	4
VAS	1
Length of Stay	3

13.2 Summary Forest plot of Continuous outcomes

13.2.1 Continuous Outcomes Table

md = mean difference lb = lower bound of 95% confidence interval ub = upper bound of 95% confidence interval tf = trim and fill

```
overall_continuous <-
  overall %>% subset(type ==  "cont") %>% subset(select = c(Outcome,
                                                            n_studies,
                                                            es,
                                                            lower_ci,
                                                            upper_ci,
                                                            p)) %>% as_tibble()
overall_continuous %>% gt() %>% tab_header(title = "Summary Table for Continuous Outcomes") %>% cols_me
```

Summary Table for Continuous Outcomes

Outcome	Studies, n	MD (95% CI)	p
Operative time	4	8.15 (0.05-16.25)	0.05
VAS	1	-2.10 (-2.66–1.54)	<0.001
Length of Stay	3	-2.89 (-3.55–2.23)	<0.001

13.2.2 Continuous Forest plot

```
continuous_plot <- overall_continuous %>%
  forestplot(
    mean = es,
    lower = lower_ci,
    upper = upper_ci,
    labeltext = c(Outcome, n_studies, es, lower_ci, upper_ci, p),
  zero = 0,
  vertices = TRUE,
  title = "A. Forest plot of MA Outcomes for Continuous Outcomes",
  clip = c(-4, 2),
  xlab = "MD [95% CI]",
  graph.pos = 3
) %>% fp_set_style(
    box = c("black"),
    line = "black",
    txt_gp = fpTxtGp(
      ticks = gpar(fontfamily = "", cex = 1),
      xlab  = gpar(fontfamily = "", cex = 1)
    )) %>% fp_add_lines("steelblue") %>%
  fp_add_header("Outcome",
                "Studies, n",
                  "MD",
                "[ 95%", " CI ]",
                "P-Value")  %>% fp_decorate_graph(box = TRUE) %>% fp_set_zebra_style("#EFEFEF")
continuous_plot
```

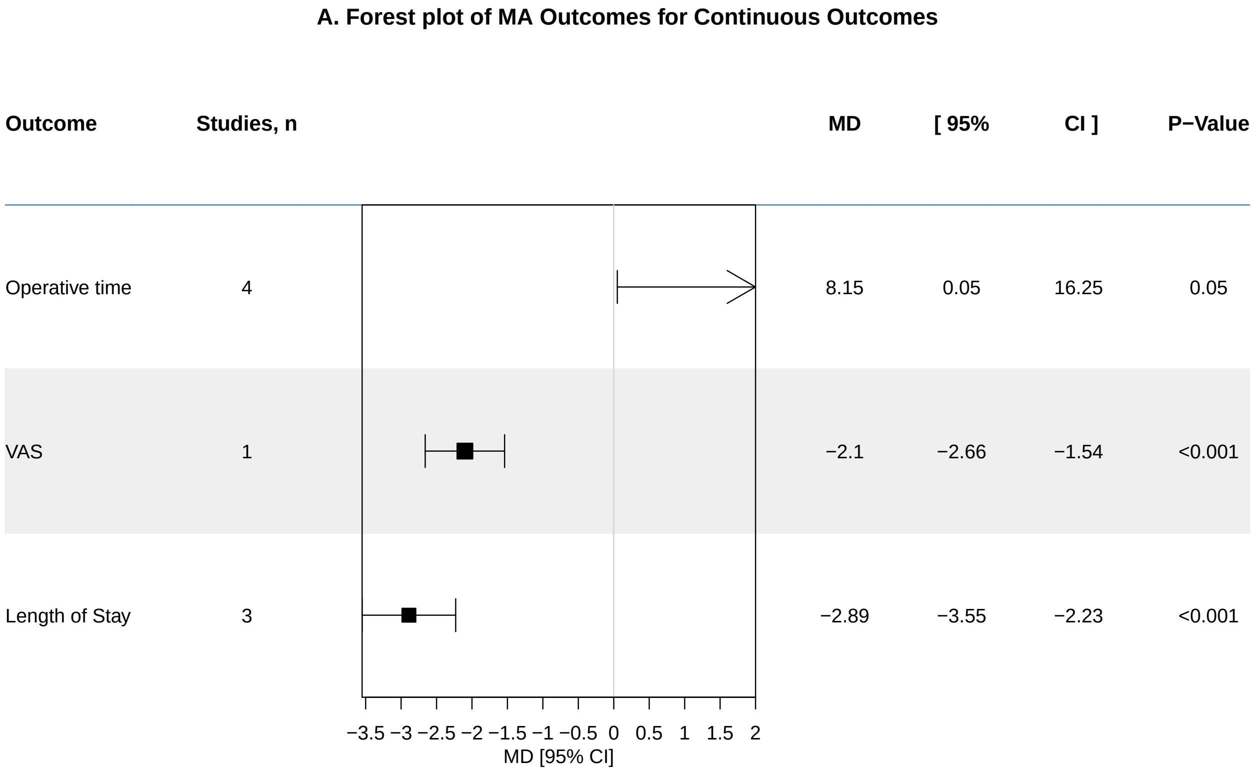

13.3 Summary Forest plot of Binary outcomes

13.3.1 Binary Outcomes Table

md = mean difference lb = lower bound of 95% confidence interval ub = upper bound of 95% confidence interval tf = trim and fill

```
overall_binary <-
  overall %>% subset(type ==  "binary") %>% subset(select = c(Outcome,
                                                             n_studies,
                                                             es,
                                                             lower_ci,
                                                             upper_ci,
                                                             p)) %>% as_tibble()
overall_binary %>% gt() %>% tab_header(title = "Summary Table for Binary Outcomes") %>% cols_merge(colu
```

Summary Table for Binary Outcomes

Outcome	Studies, n	RR (95% CI)	p
Immediate SFR	9	1.15 (0.99-1.34)	0.08
Final SFR	4	0.97 (0.92-1.02)	0.2
Auxiliary Treatment	2	1.77 (1.00-3.12)	0.05
Overall Complications	4	0.42 (0.21-0.81)	0.01
Fever	4	0.70 (0.35-1.41)	0.32
Infection	4	0.70 (0.32-1.54)	0.37
Sepsis	1	0.11 (0.01-2.06)	0.14
Haematoma	1	0.41 (0.02-9.84)	0.58
Pain	2	0.21 (0.07-0.60)	<0.001
Embolisation	2	0.29 (0.03-2.55)	0.26
Transfusion	3	0.16 (0.03-0.88)	0.04
CD I	2	0.59 (0.27-1.29)	0.19
CD II	2	0.33 (0.06-1.84)	0.2
CD III	2	0.68 (0.10-4.53)	0.69
CD I-II	2	0.50 (0.25-1.00)	0.05
CD III-V	2	0.68 (0.10-4.53)	0.69

13.3.2 Binary Forest plot - Meta-Analysis

Reference = No Suction

```
binary_plot <- overall_binary %>%
  forestplot(
    mean = es,
    lower = lower_ci,
    upper = upper_ci,
    labeltext = c(Outcome, n_studies, es, lower_ci, upper_ci, p),
  zero = 1,
  vertices = TRUE,
  title = "A. Forest plot of MA Outcomes for Binary Outcomes",
  clip = c(-1.5, 1.5),
  xlab = "RR [95% CI]",
  graph.pos = 3
) %>% fp_set_style(
    box = c("black"),
    line = "black",
    txt_gp = fpTxtGp(
      ticks = gpar(fontfamily = "", cex = 1),
      xlab  = gpar(fontfamily = "", cex = 1)
    )) %>% fp_add_lines("steelblue") %>%
  fp_add_header("Outcome",
                "Studies, n",
                  "RR",
                "[ 95%", " CI ]",
                "P-Value")  %>% fp_decorate_graph(box = TRUE) %>% fp_set_zebra_style("#EFEFEF")
binary_plot
```

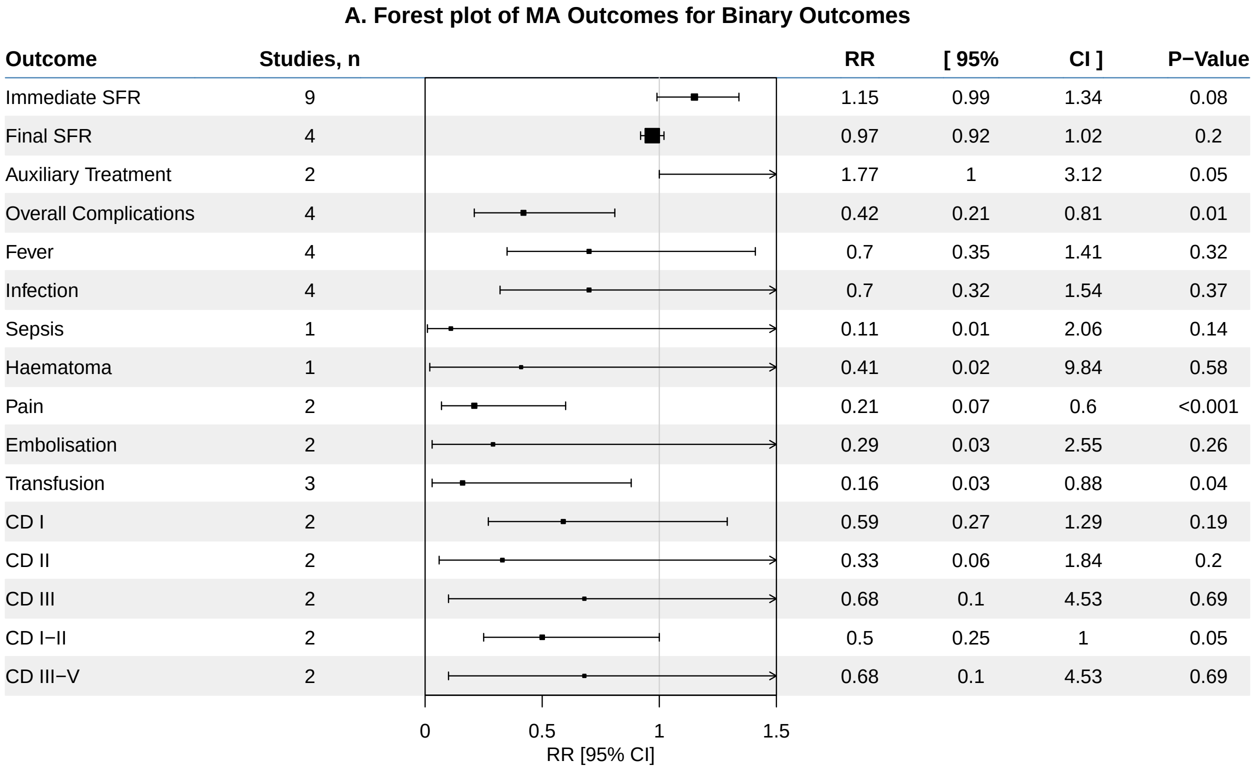

Supplement: Supplementary file 2 — Appendix S2. Supporting Information [file BCO2-5-895-s004.pdf]
