# Supplementary material for: Suction use in ureterorenoscopy: A systematic review and meta‐analysis of comparative studies
Source: BJUI Compass. 2024 Jul 8;5(10):895–912. doi: 10.1002/bco2.408 (PMC11479806; doi:10.1002/bco2.408)
Supplement: Supplementary file 3 — Table S1. Risk of bias for randomized controlled trials (Risk of Bias 2 tool) Table S2. Risk of bias for non‐randomized comparative trials (ROBINS‐I tool) [file BCO2-5-895-s007.docx]

| Author/ Year | Randomization | Deviation from intended interventions | Missing outcome | Measurement of outcome | Selection of reported results | Overall Risk of bias |
| --- | --- | --- | --- | --- | --- | --- |
| Sur 2022 | Some concerns | Low | Low | Low | Low | Some concerns |
| Tang 2023 | Some concerns | Low | Low | Some concerns | Low | Some concerns |
| Du 2019 | Some concerns | High | Low | High | Some concerns | High |
| Levhevallier 2003 | Some concerns | High | Low | High | High | High |
| Zhang 2022 | Some concerns | High | Low | High | Some concerns | High |

**Supplementary Table 1**. Risk of bias for randomized controlled trials (Risk of Bias 2 tool)

| Author/ Year | Confounding | Selection | Measurement of exposure | Departures from exposure | Missing data | Measurement of Outcomes | Reported results | Overall Risk of bias |
| --- | --- | --- | --- | --- | --- | --- | --- | --- |
| Chen 2019 | Moderate | Moderate | Low | Moderate | Low | Serious | Low | Serious |
| Lai 2020 | Moderate | Moderate | Low | Low | Low | Low | Low | Moderate |
| Zhu 2018 | Moderate | Moderate | Low | Low | Low | Moderate | Low | Moderate |
| Zhang 2021 | Moderate | Moderate | Low | Low | Low | Low | Low | Moderate |
| Huang 2023 | Low | Moderate | Low | Low | Low | Low | Low | Moderate |
| Wu 2022 | Moderate | Moderate | Low | Low | Low | Serious | Low | Serious |
| Ding 2023 | Moderate | Moderate | Low | Low | Low | Low | Low | Moderate |
| Zhai 2023 | Moderate | Moderate | Low | Low | Low | Serious | Low | Serious |
| Qian 2022 | Low | Low | Low | Low | Low | Moderate | Moderate | Moderate |
| Deng 2022 | Low | Moderate | Low | Low | Low | Low | Low | Moderate |
| AlSmadi 2019 | Moderate | Moderate | Low | Low | Low | Low | Low | Moderate |

**Supplementary Table 2**. Risk of bias for non-randomized comparative trials (ROBINS-I tool)
