# Supplementary material for: Suction use in ureterorenoscopy: A systematic review and meta‐analysis of comparative studies
Source: BJUI Compass. 2024 Jul 8;5(10):895–912. doi: 10.1002/bco2.408 (PMC11479806; doi:10.1002/bco2.408)
Supplement: Supplementary file 4 — Table S3. Certainty of the evidence for each outcomes based on the GRADE approach [file BCO2-5-895-s003.docx]

| **Outcomes** | **Certainty of the evidence (GRADE)** |
| --- | --- |
| Stone-free rate immediate | ⊝⊝⊝⊝ **Very Low** |
| Stone-free rate final | ⊝⊝⊝⊝ **Very Low** |
| Auxiliary treatments | ⊝⊝⊝⊝ **Very Low** |
| Overall complications | ⊕⊝⊝⊝  **Low** |
| Fever | ⊕⊝⊝⊝  **Low** |
| Infections | ⊕⊝⊝⊝  **Low** |
| Sepsis | ⊝⊝⊝⊝ **Very Low** |
| Pain | ⊕⊝⊝⊝  **Low** |
| Transfusion rates | ⊝⊝⊝⊝ **Very Low** |
| Embolization | ⊝⊝⊝⊝ **Very Low** |
| Ureteral stricture formation | ⊝⊝⊝⊝ **Very Low** |
| Clavien-Dindo complications | ⊕⊝⊝⊝  **Low** |
| Operative time | ⊕⊝⊝⊝  **Low** |
| Length of stay | ⊕⊝⊝⊝  **Low** |

**Supplementary Table 2.** Certainty of the evidence for each outcomes based on the GRADE approach

**GRADE Working Group grades of evidence**

**High certainty:** We are very confident that the true effect lies close to that of the estimate of the effect

**Moderate certainty:** We are moderately confident in the effect estimate; the true effect is likely to be close to the estimate of the effect, but there is a possibility that it is substantially different

**Low certainty:** Our confidence in the effect estimate is limited; the true effect may be substantially different from the estimate of the effect

**Very low certainty:** We have very little confidence in the effect estimate; the true effect is likely to be substantially different from the estimate of effect
