# Supplementary figures and images for: Suction use in ureterorenoscopy: A systematic review and meta‐analysis of comparative studies
Source: BJUI Compass. 2024 Jul 8;5(10):895–912. doi: 10.1002/bco2.408 (PMC11479806; doi:10.1002/bco2.408)

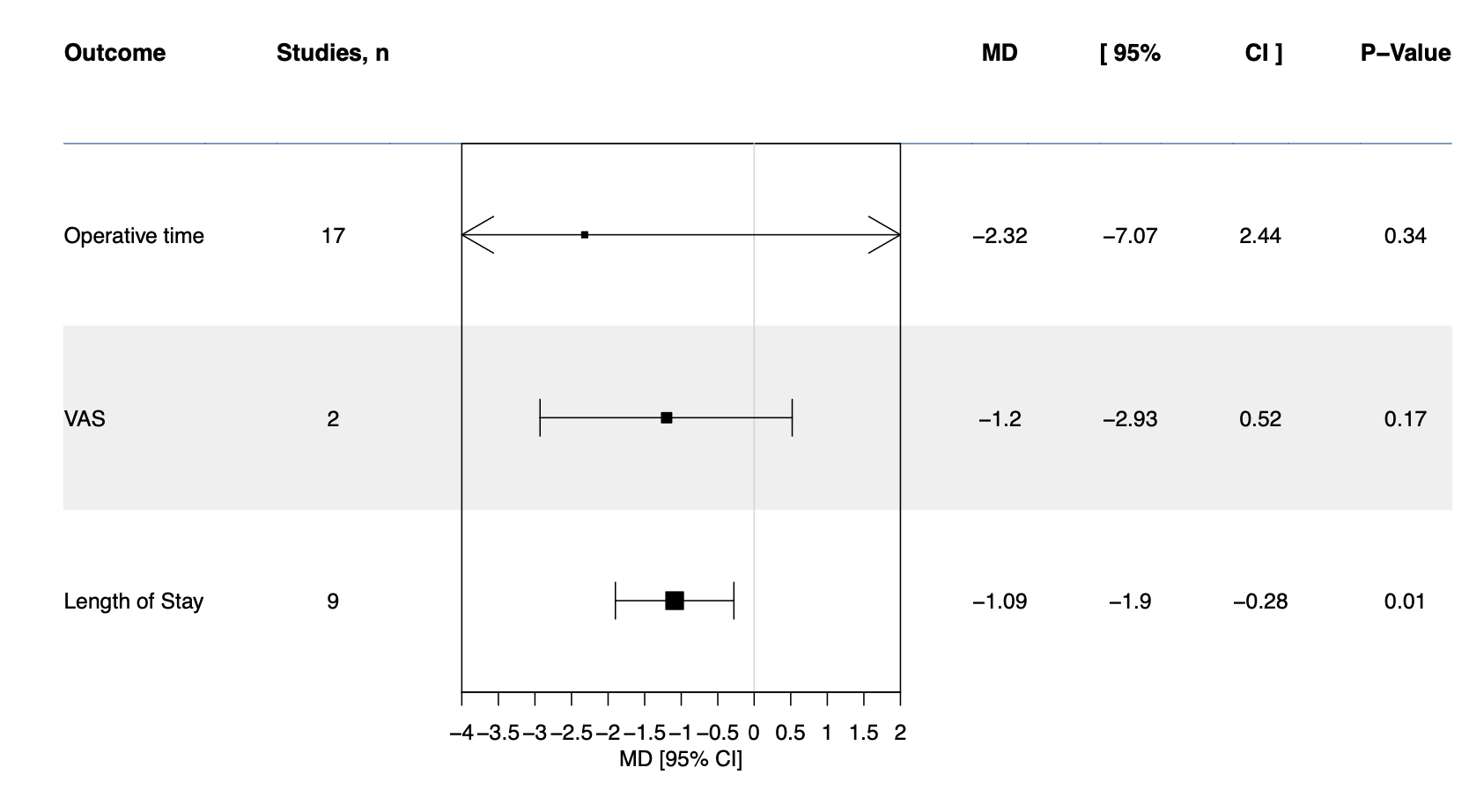

Supplement: Supplementary file 5 — Figure S1. Forest plot for overall comparative analysis of continuous outcomes [file BCO2-5-895-s013.png]

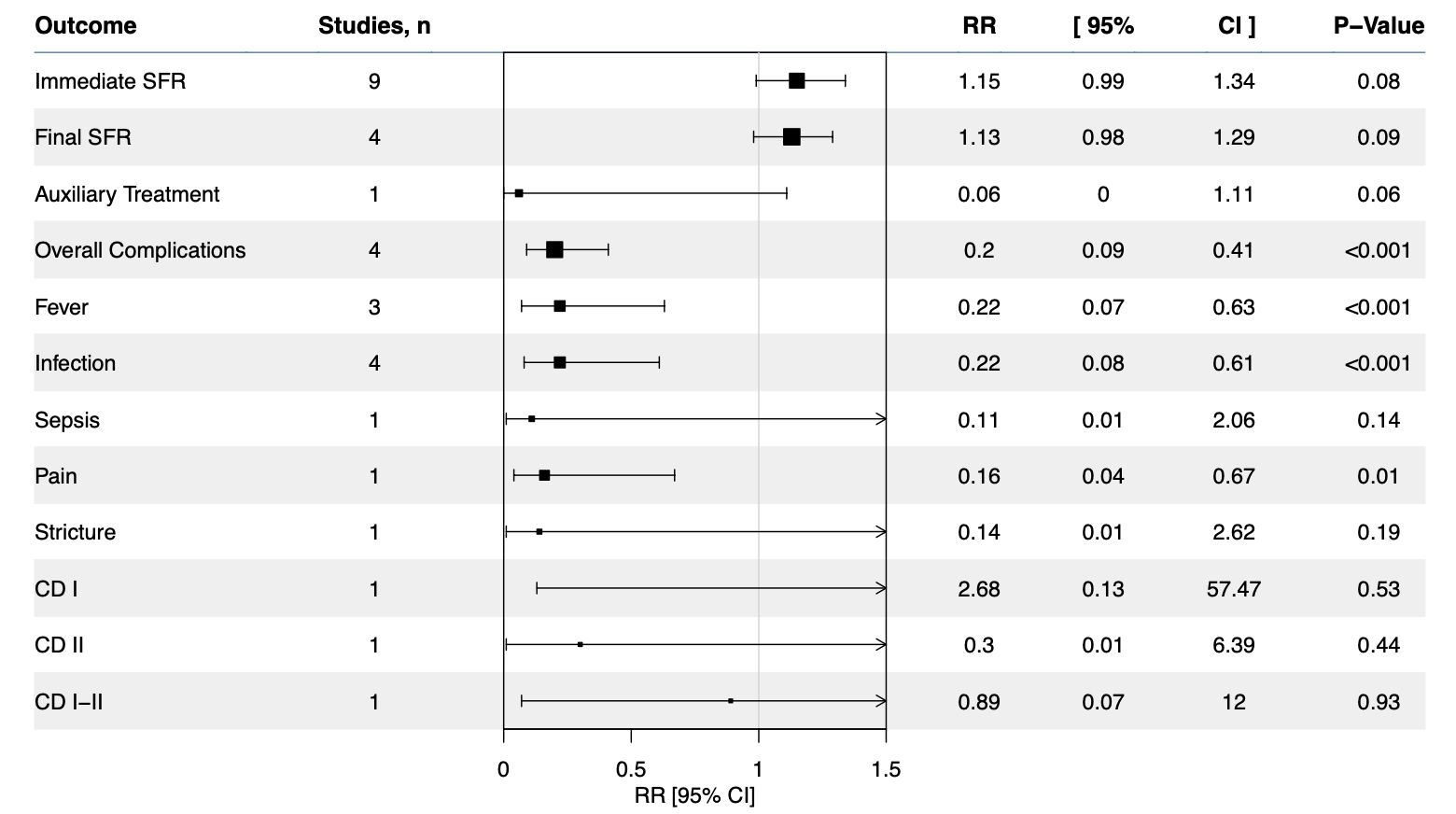

Supplement: Supplementary file 6 — Figure S2. Forest plot for analysis of binary outcomes in RCTs [file BCO2-5-895-s010.png]

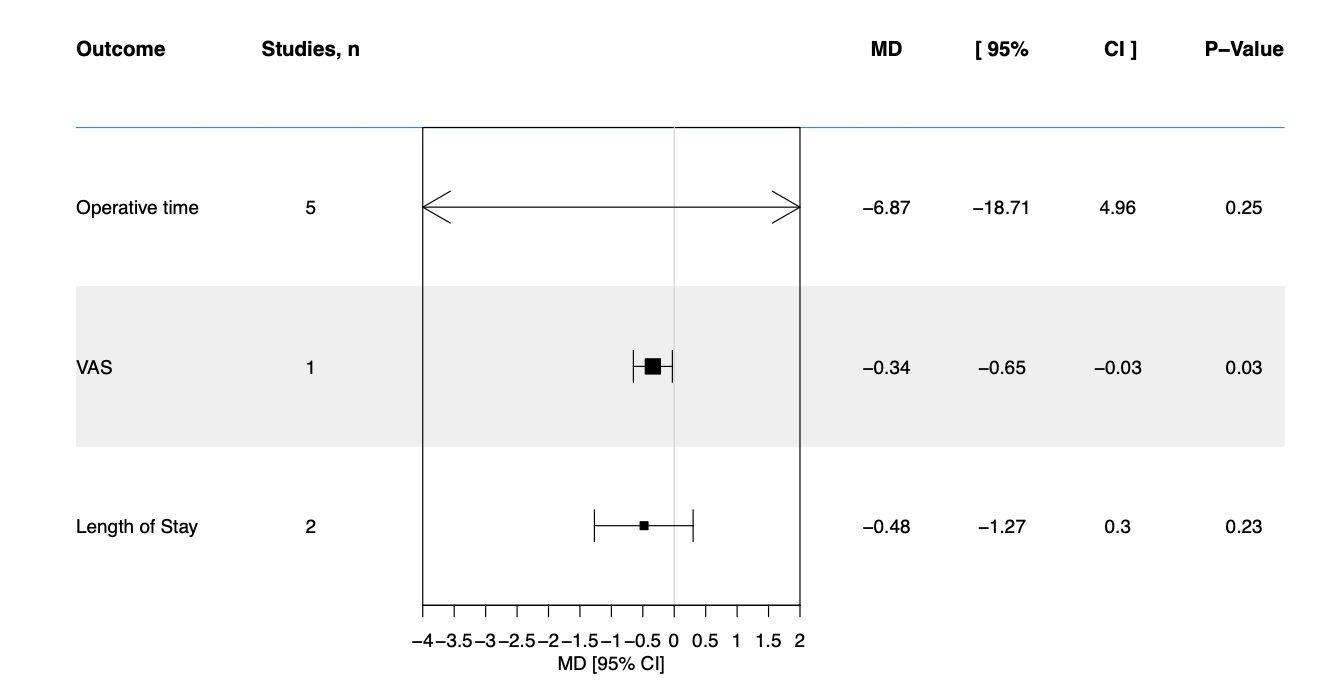

Supplement: Supplementary file 7 — Figure S3. Forest plot for analysis of continuous outcomes in RCTs [file BCO2-5-895-s005.png]

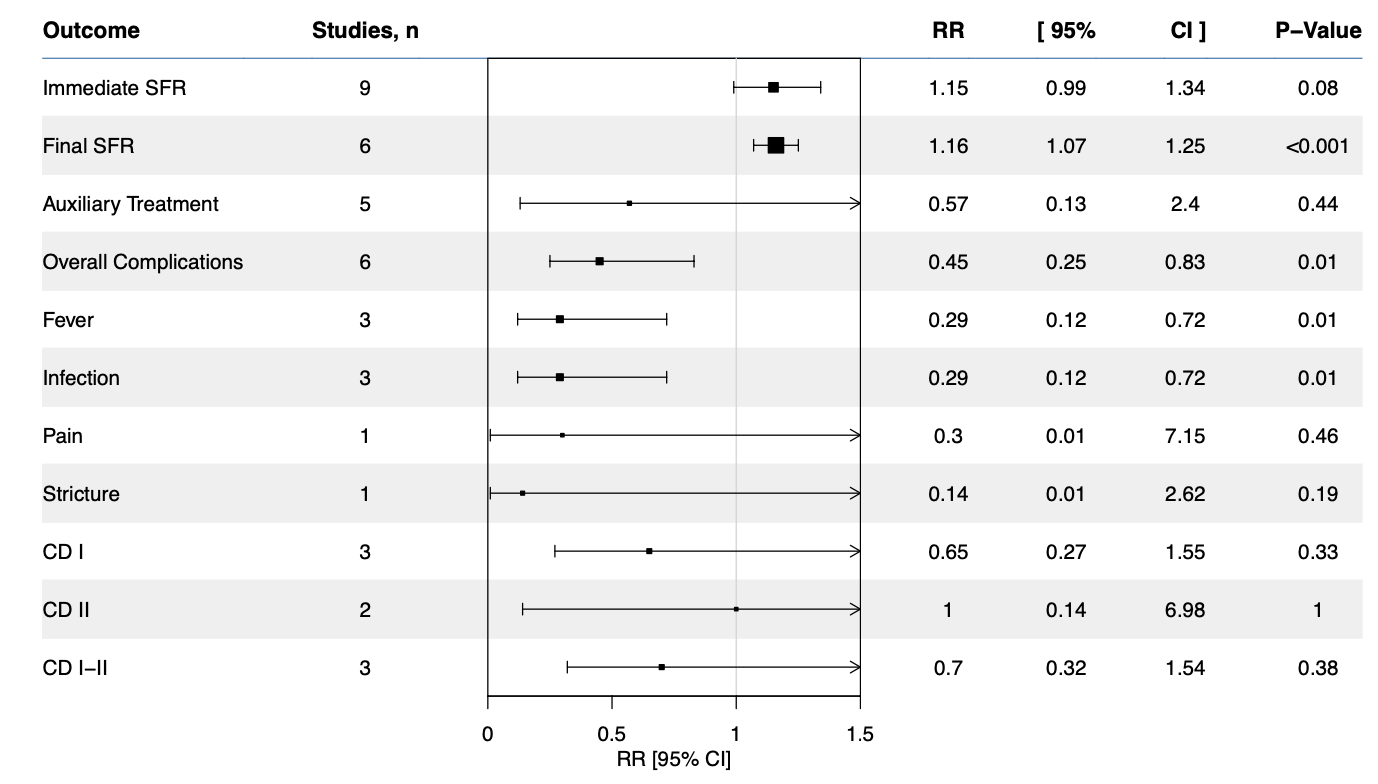

Supplement: Supplementary file 8 — Figure S4. Forest plot for analysis of binary outcomes in semi‐rigid URS [file BCO2-5-895-s012.png]

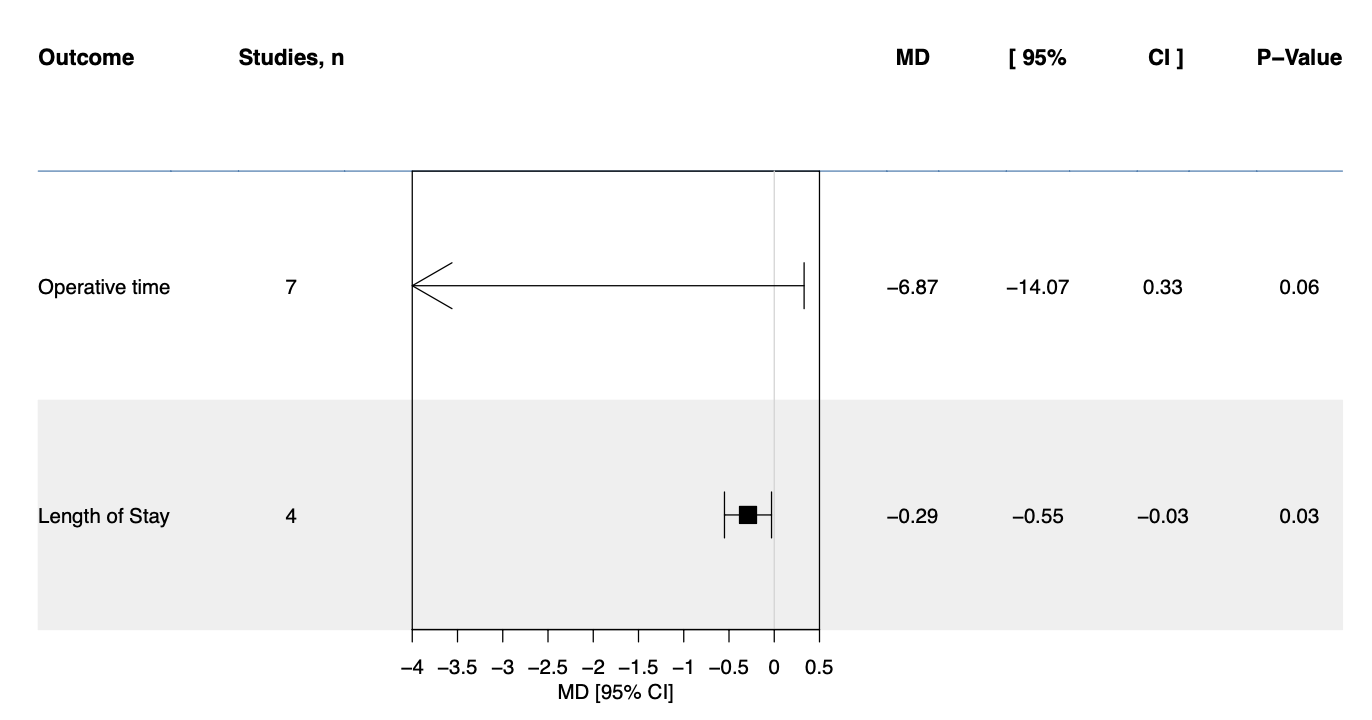

Supplement: Supplementary file 9 — Figure S5. Forest plot for analysis of continuous outcomes in semi‐rigid URS [file BCO2-5-895-s001.png]

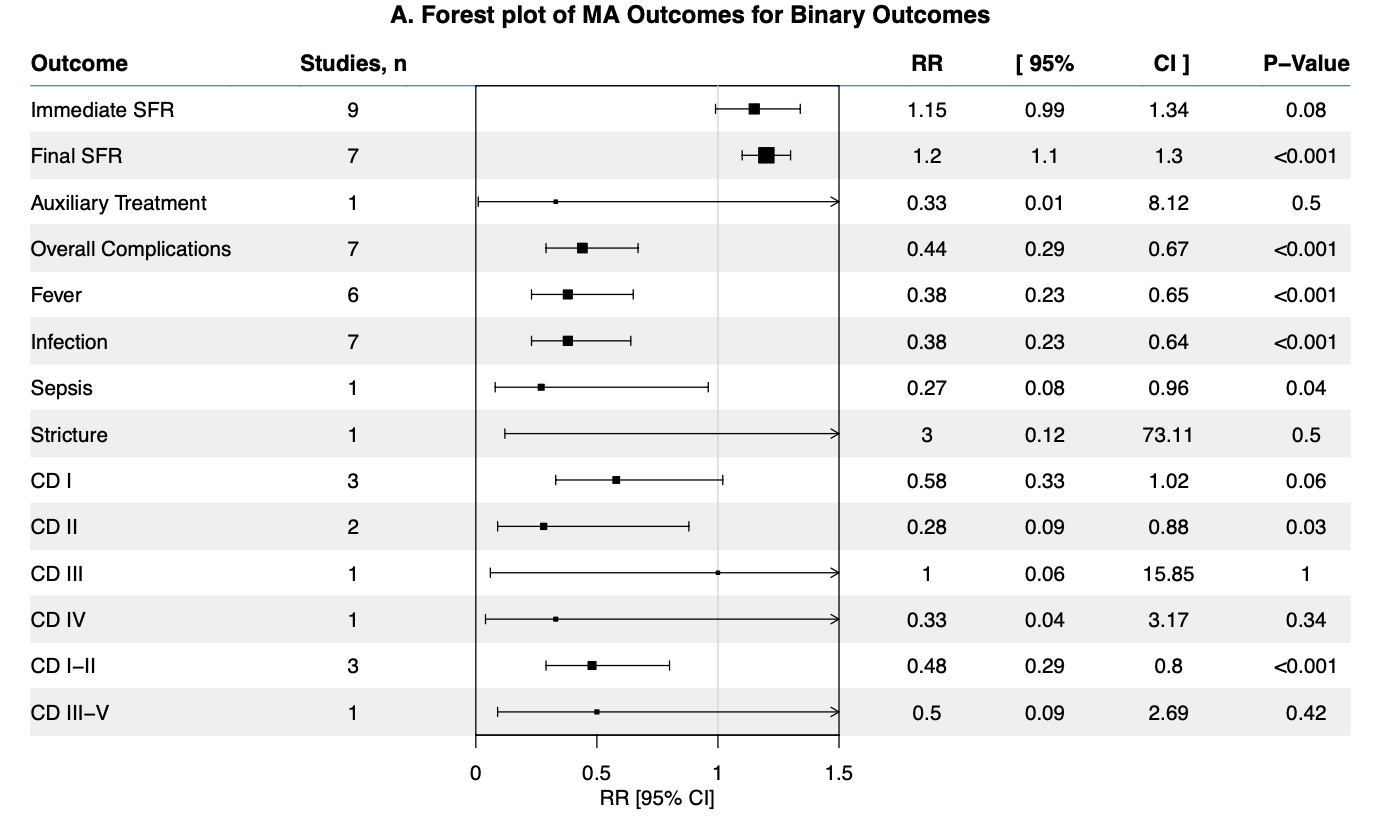

Supplement: Supplementary file 10 — Figure S6. Forest plot for analysis of binary outcomes in flexible URS/RIRS [file BCO2-5-895-s006.png]

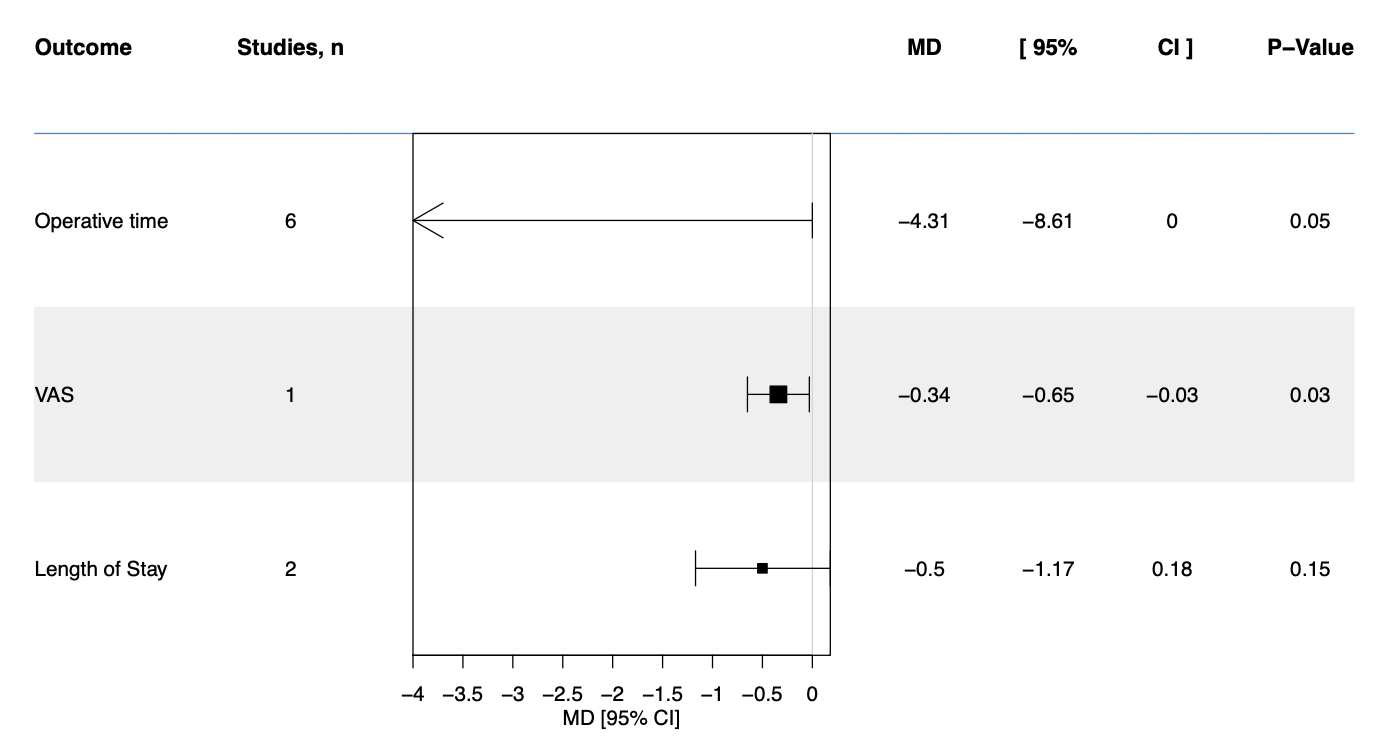

Supplement: Supplementary file 11 — Figure S7. Forest plot for analysis of continuous outcomes in flexible URS/RIRS [file BCO2-5-895-s009.png]

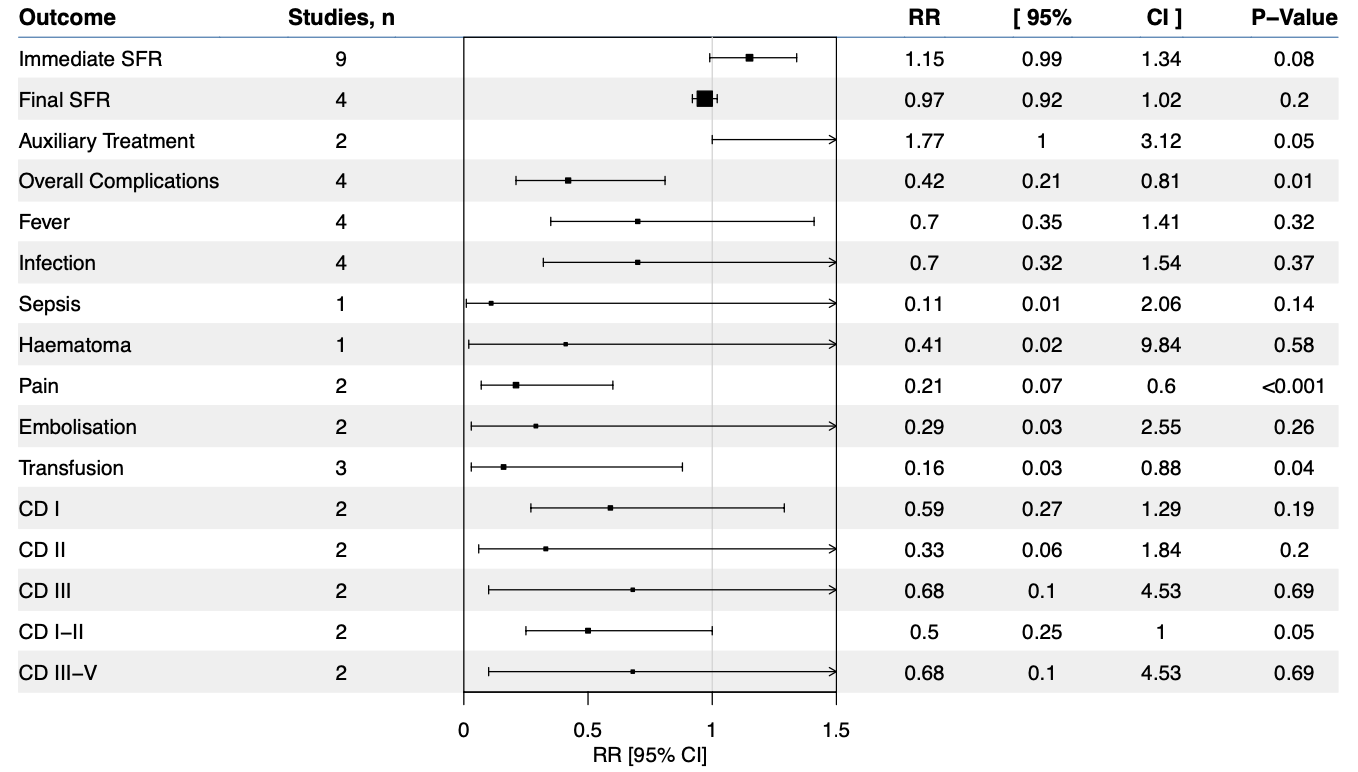

Supplement: Supplementary file 12 — Figure S8. Forest plot for analysis of binary outcomes in flexible URS/RIRS vs mini‐PCNL [file BCO2-5-895-s011.png]

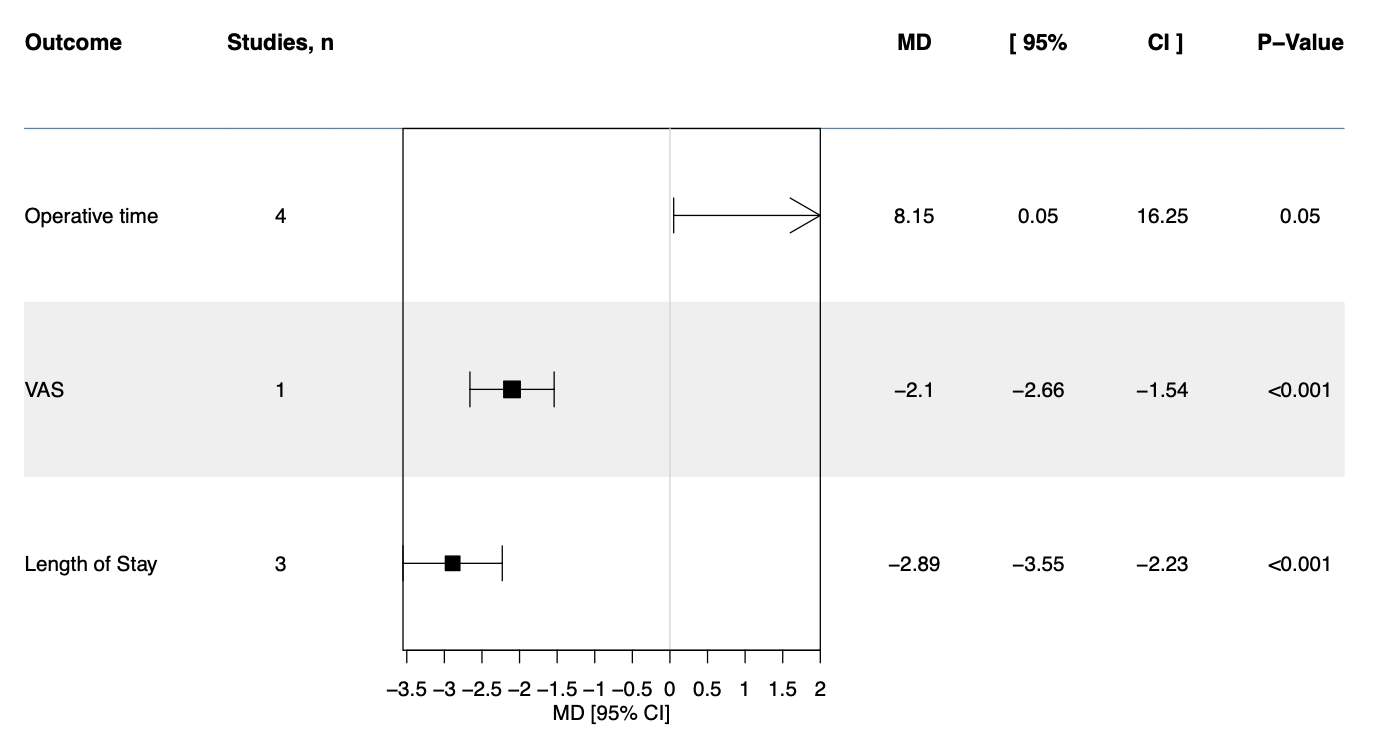

Supplement: Supplementary file 13 — Figure S9. Forest plot for analysis of continuous outcomes in flexible URS/RIRS vs mini‐PCNL [file BCO2-5-895-s008.png]
